# Supplementary material for: Unleashing a novel function of Endonuclease G in mitochondrial genome instability
Source: eLife. 2022 Nov 17;11:e69916. doi: 10.7554/eLife.69916 (PMC9711528; doi:10.7554/eLife.69916)
Supplement: Figure 1—source data 1. [file elife-69916-fig1-data1.zip › Figure 1_Source data1_main/Figure 1E_Gel profile DMS protection assay/Figure 1E_Gel profile DMS protection.pptx]

## Slide 1
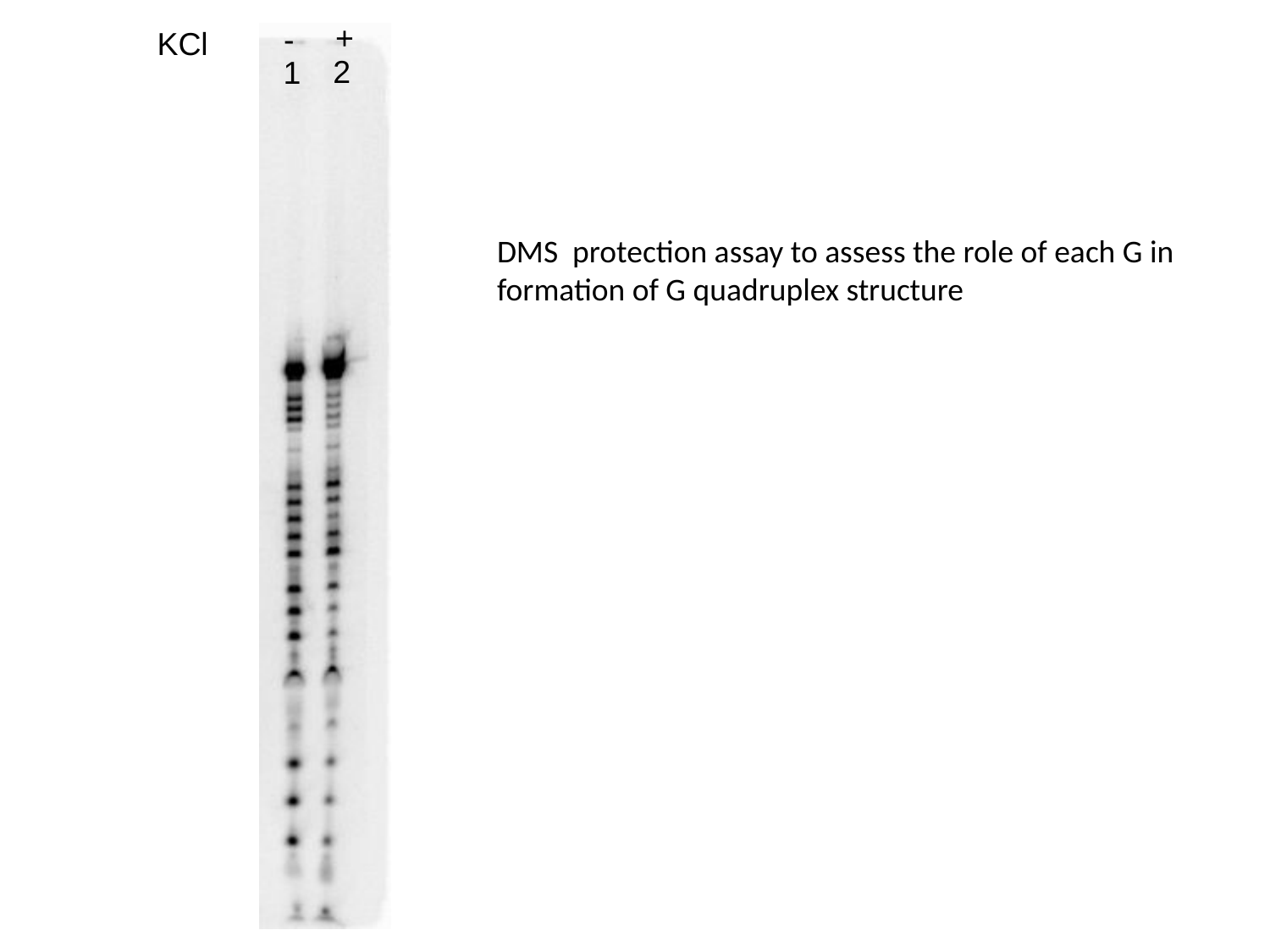

+
-
KCl
2
1
DMS protection assay to assess the role of each G in formation of G quadruplex structure
