# Supplementary material for: Unleashing a novel function of Endonuclease G in mitochondrial genome instability
Source: eLife. 2022 Nov 17;11:e69916. doi: 10.7554/eLife.69916 (PMC9711528; doi:10.7554/eLife.69916)
Supplement: Figure 1—source data 1. [file elife-69916-fig1-data1.zip › Figure 1_Source data1_main/Figure 1C_EMSA Gel profile_Mitochondrial region_presence and absence_KCl/Figure 1C_EMSA Gel profile_Mitochondrial region_presence or absence KCl.pptx]

## Slide 1
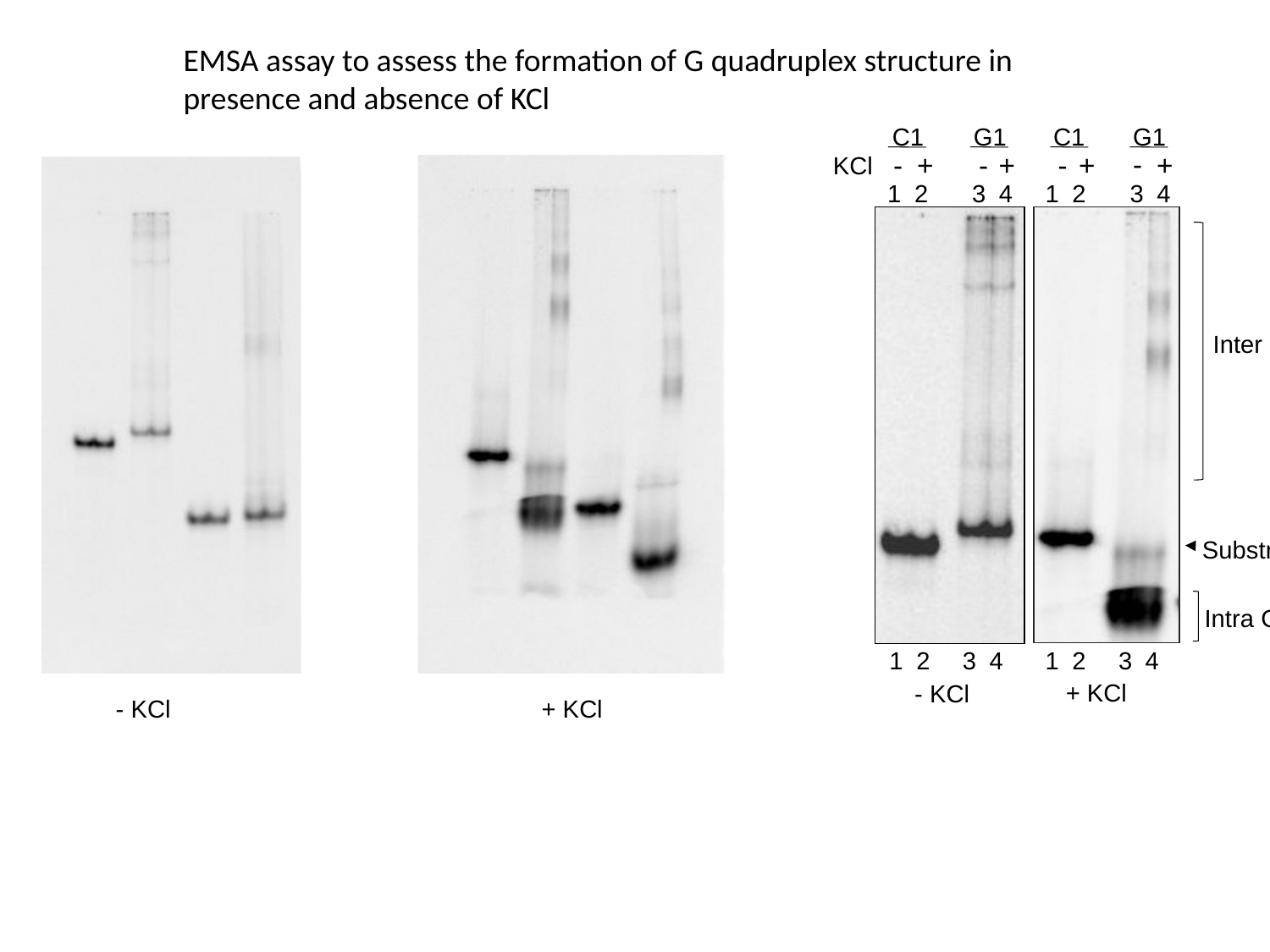

EMSA assay to assess the formation of G quadruplex structure in presence and absence of KCl
C1
G1
C1
G1
 -
 +
 -
 +
 -
 -
 +
 +
KCl
4
4
2
3
2
3
1
1
Inter G
Substrate
Intra G
1
2
3
4
1
2
3
4
+ KCl
- KCl
+ KCl
- KCl
