# Supplementary figures and images for: Unleashing a novel function of Endonuclease G in mitochondrial genome instability (part 1 of 4)
Source: eLife. 2022 Nov 17;11:e69916. doi: 10.7554/eLife.69916 (PMC9711528; doi:10.7554/eLife.69916)

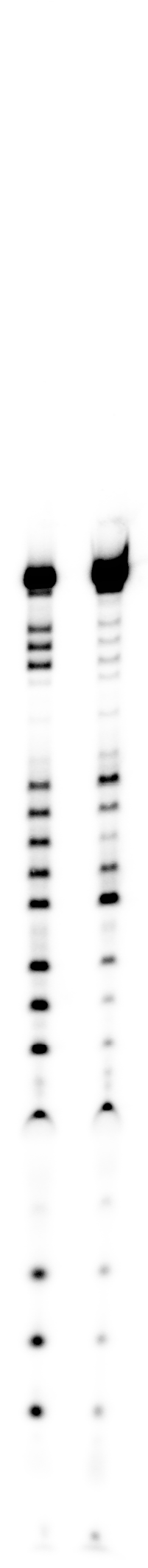

Supplement: Figure 1—source data 1. [file elife-69916-fig1-data1.zip › Figure 1_Source data1_main/Figure 1E_Gel profile DMS protection assay/Figure 1E_Gel profile_DMS protection assay_regI.tif]

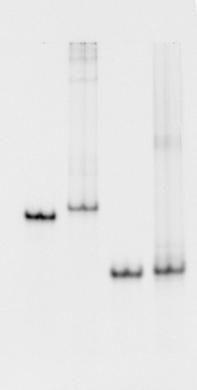

Supplement: Figure 1—source data 1. [file elife-69916-fig1-data1.zip › Figure 1_Source data1_main/Figure 1C_EMSA Gel profile_Mitochondrial region_presence and absence_KCl/Figure 1C_EMSA Gel profile_Mitochondrial region_absence_KCl.tif]

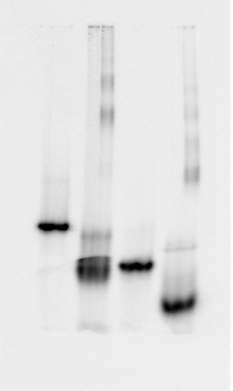

Supplement: Figure 1—source data 1. [file elife-69916-fig1-data1.zip › Figure 1_Source data1_main/Figure 1C_EMSA Gel profile_Mitochondrial region_presence and absence_KCl/Figure 1C_EMSA Gel profile_Mitochondrial region_presence_KCl.tif]

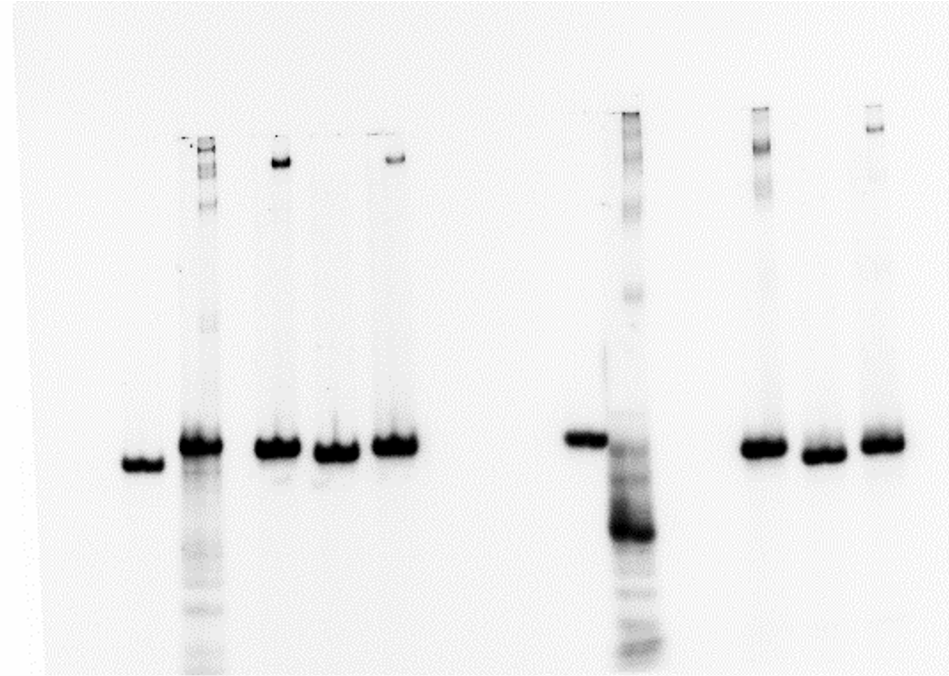

Supplement: Figure 1—source data 2. [file elife-69916-fig1-data2.zip › Figure 1_Source data2_Supplementary/Figure S1C_EMSA gel profile_Mitochondrial region 1 in presence_absence_KCl_with mutants.tif]

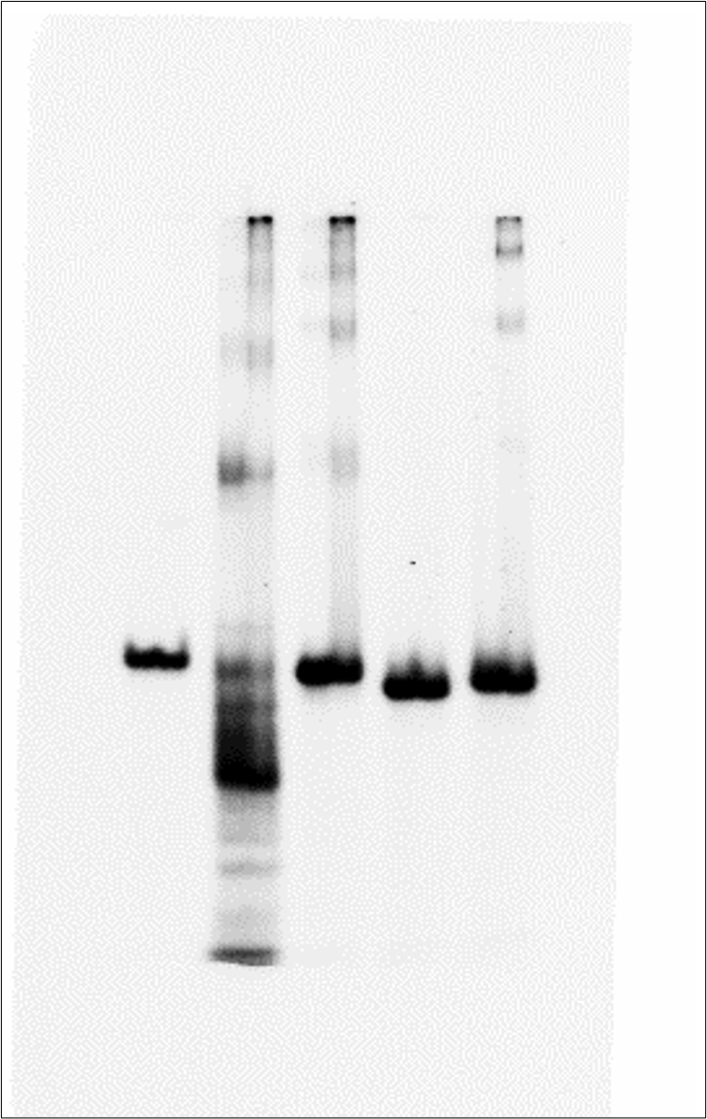

Supplement: Figure 1—source data 2. [file elife-69916-fig1-data2.zip › Figure 1_Source data2_Supplementary/Figure S1C_EMSA gel profile_Mitochondrial region 1 in presence_KCl_with mutants.tif]

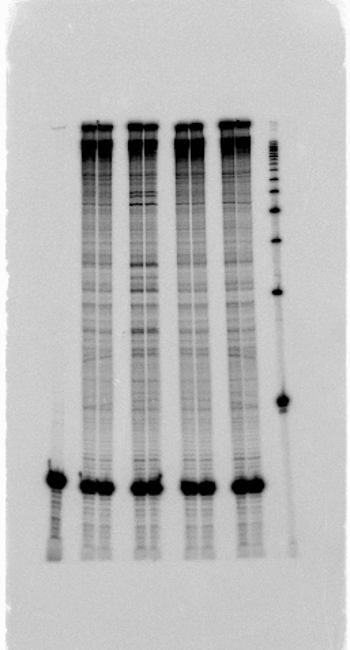

Supplement: Figure 2—source data 1. [file elife-69916-fig2-data1.zip › Figure2_Source data1_main/Figure 2E_Gel profile_Primer extension_Different ions/Figure 2E_Gel profile_Primer extension_Different salts.tif]

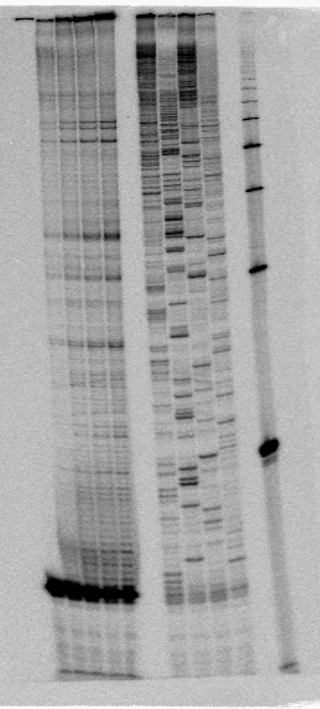

Supplement: Figure 2—source data 1. [file elife-69916-fig2-data1.zip › Figure2_Source data1_main/Figure 2C_Gel profile_Primer extension_potassium increasing concentration/Figure 1C_Gel profile_Primer extension_potassium increasing concentration.tif]

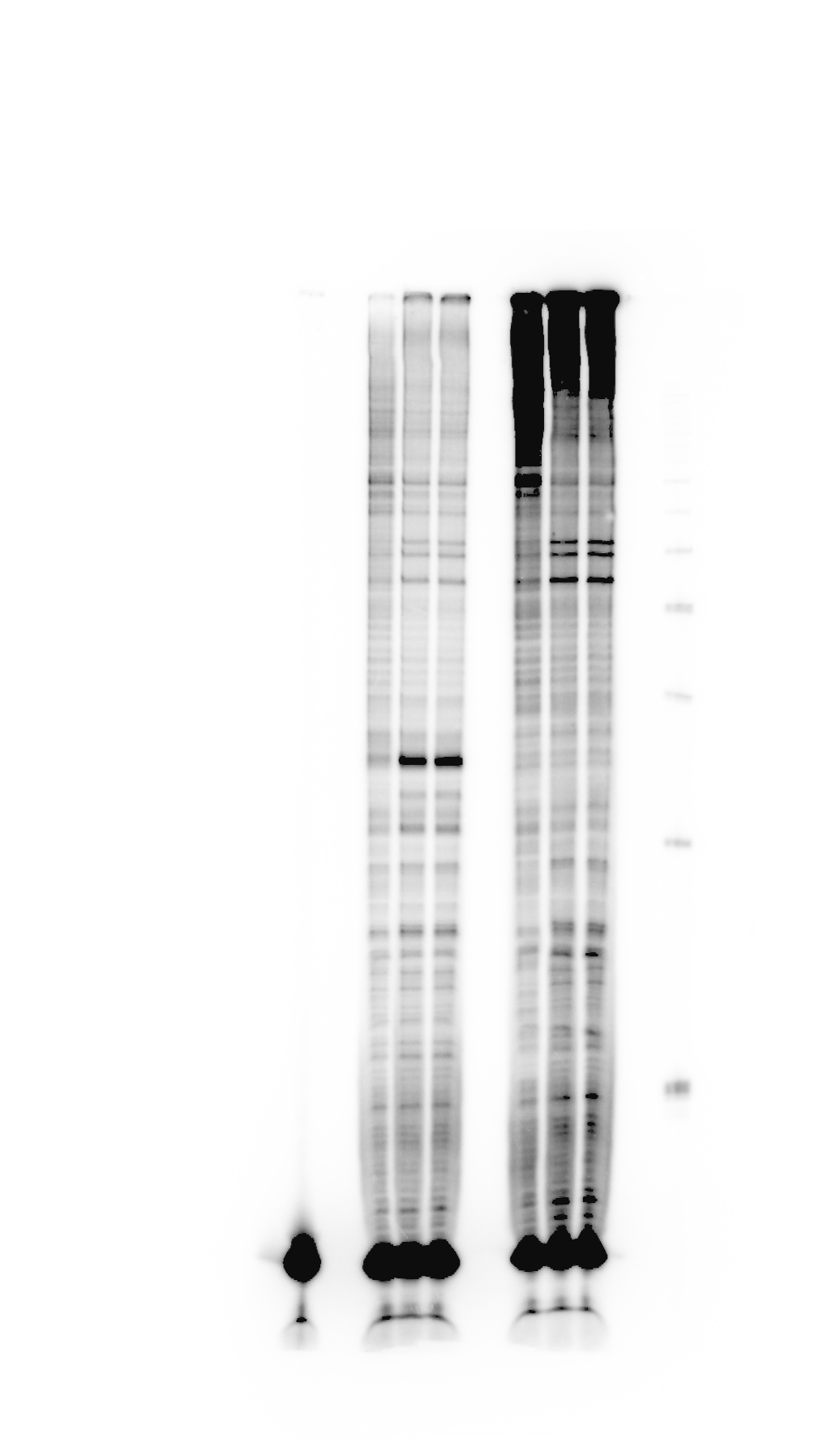

Supplement: Figure 2—source data 1. [file elife-69916-fig2-data1.zip › Figure2_Source data1_main/Figure 2D_Source data_Gel profile/Figure 2D_Gel profile_Primer extension_wild type and mutant plasmid.tif]

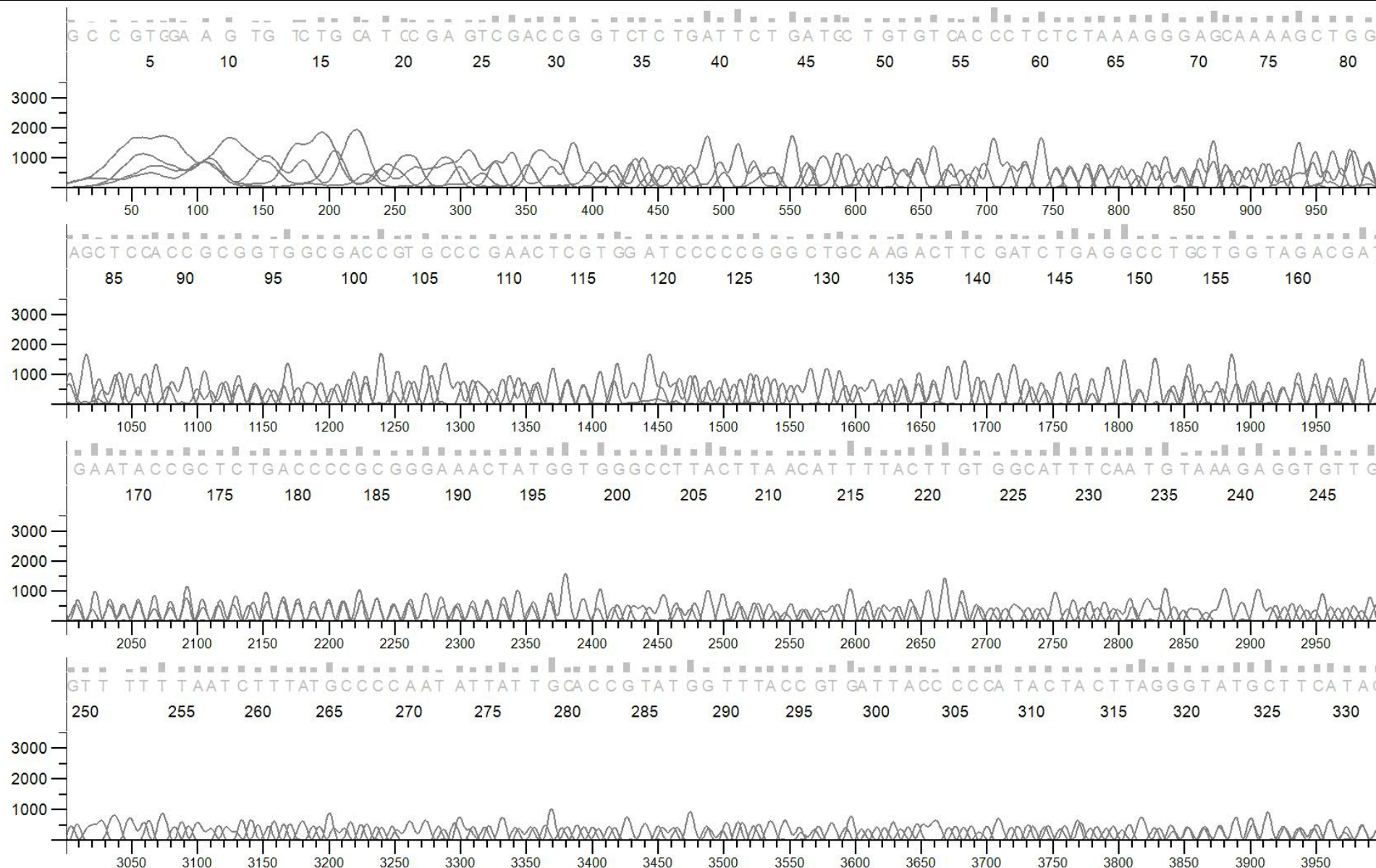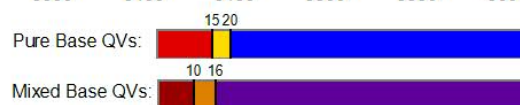

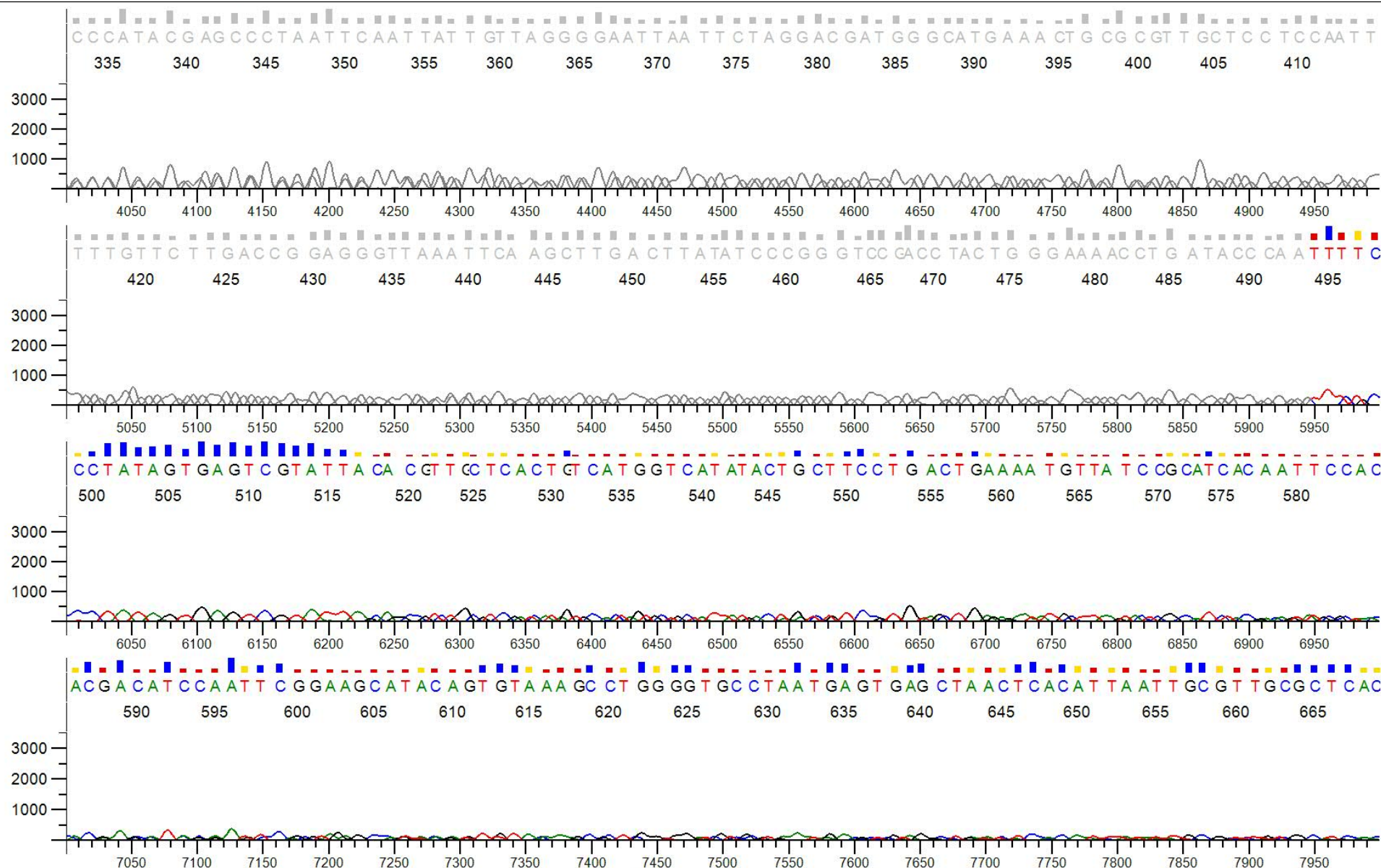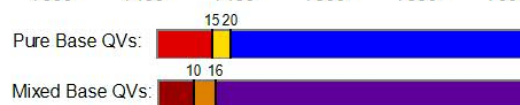

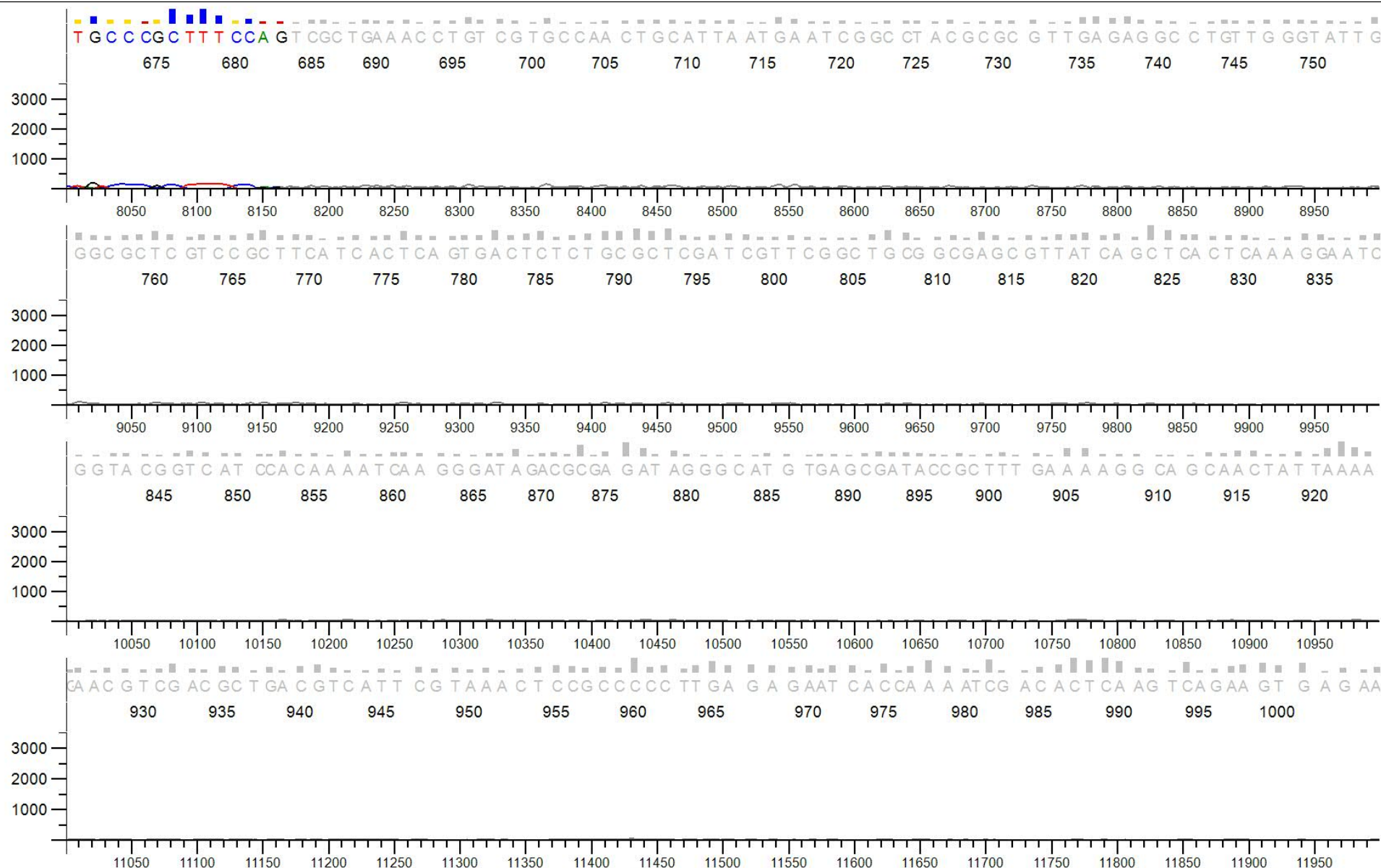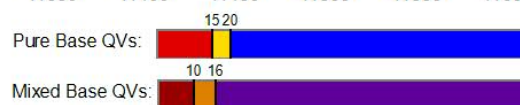

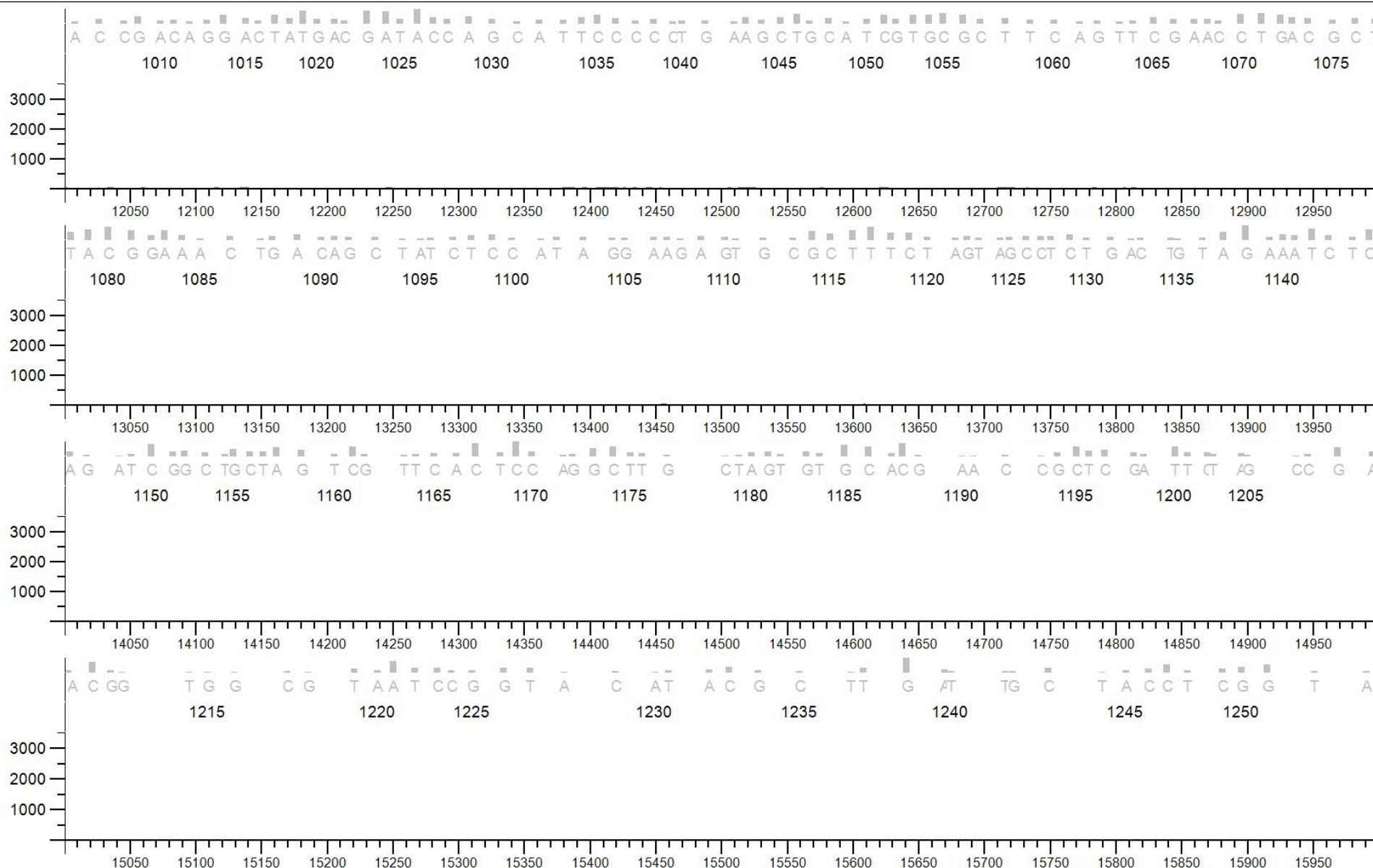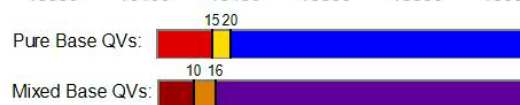

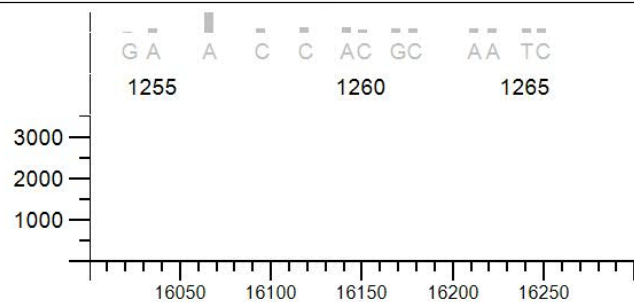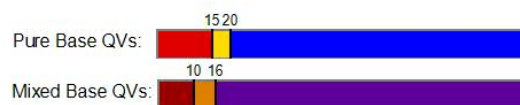

Supplement: Figure 3—source data 1. [file elife-69916-fig3-data1.zip › Figure 3A_Source data2_Bisulphite sequencing data_plasmid/RBK_PDI1_BSF_1.2_T7_FOR.pdf]

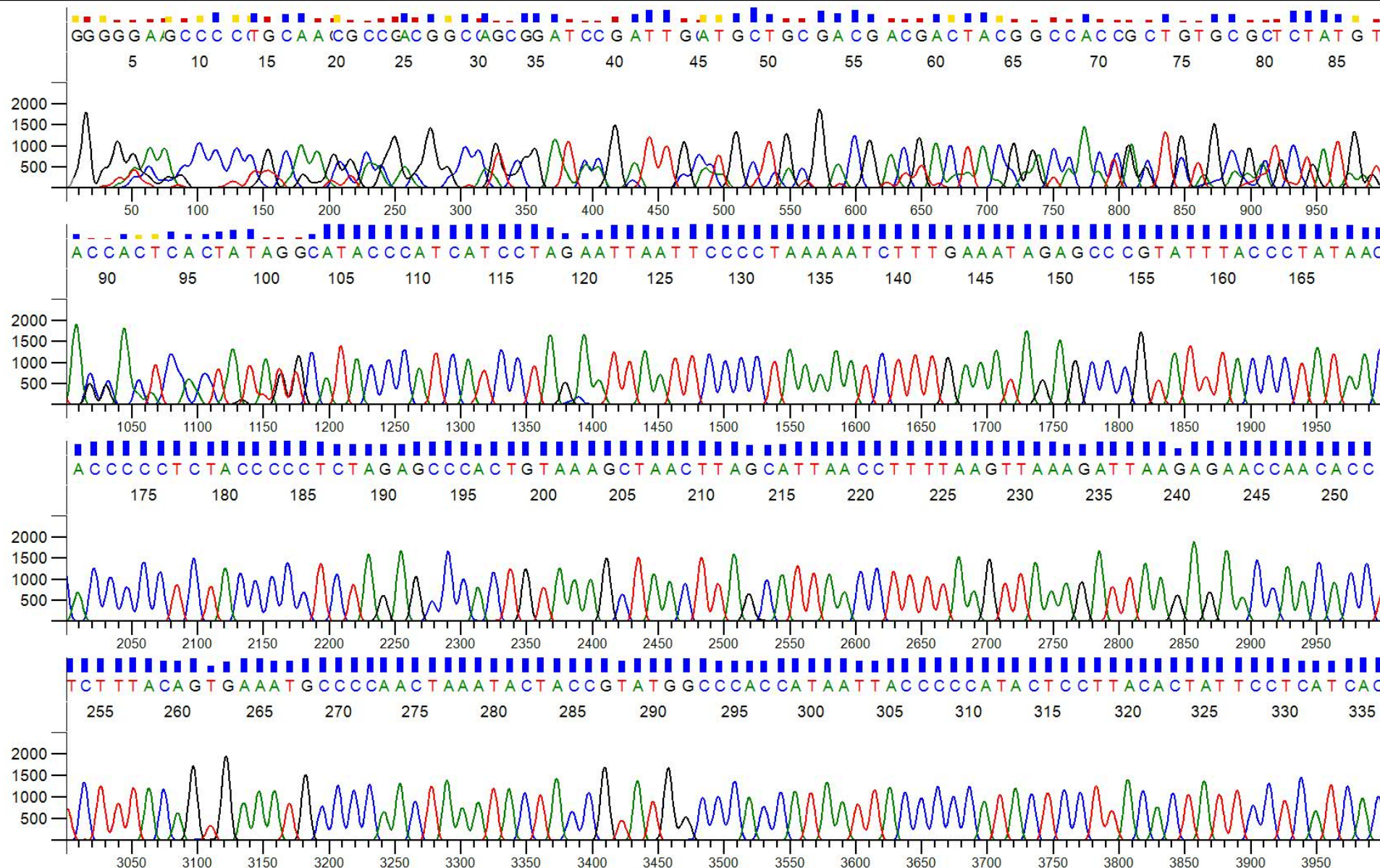

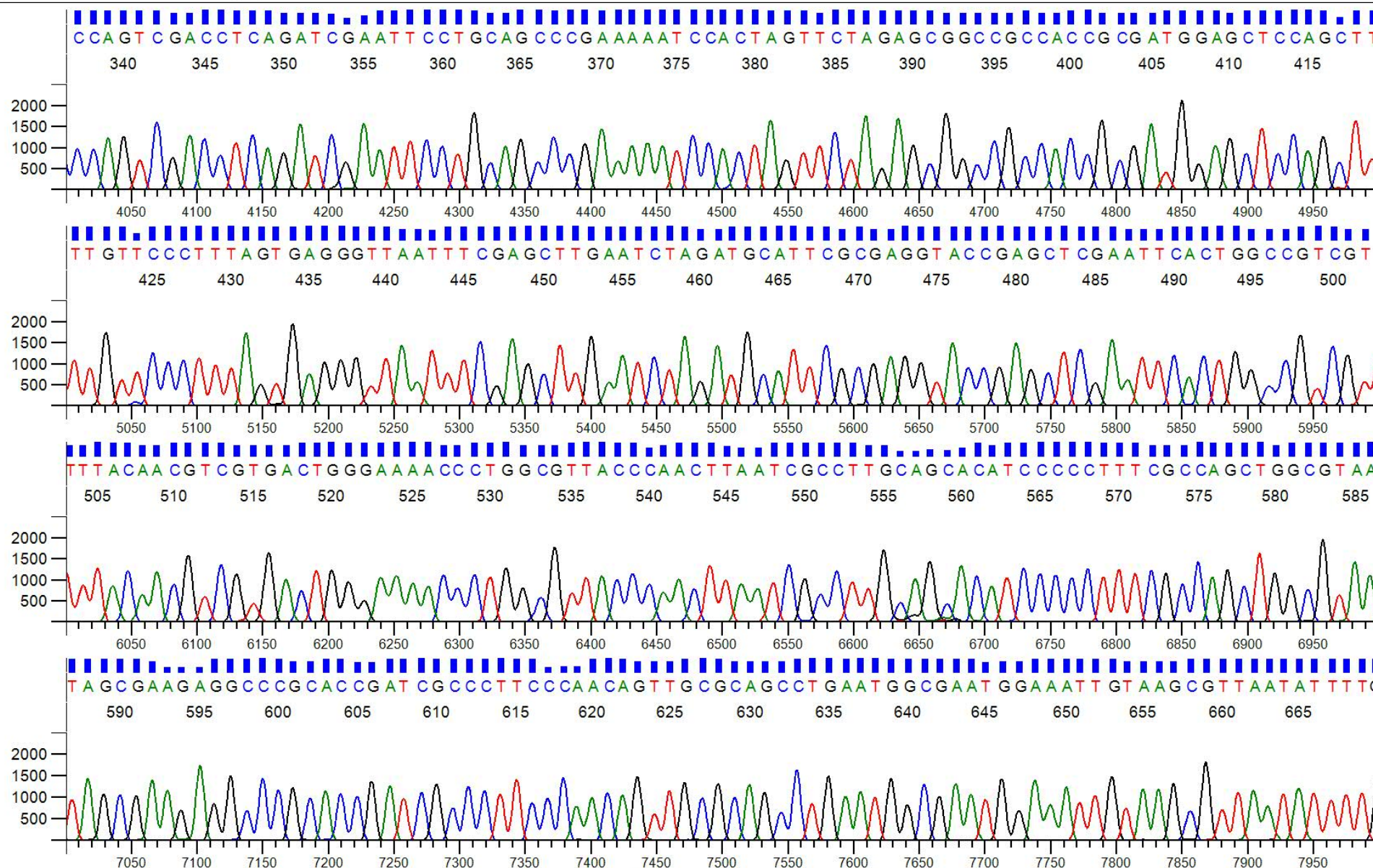

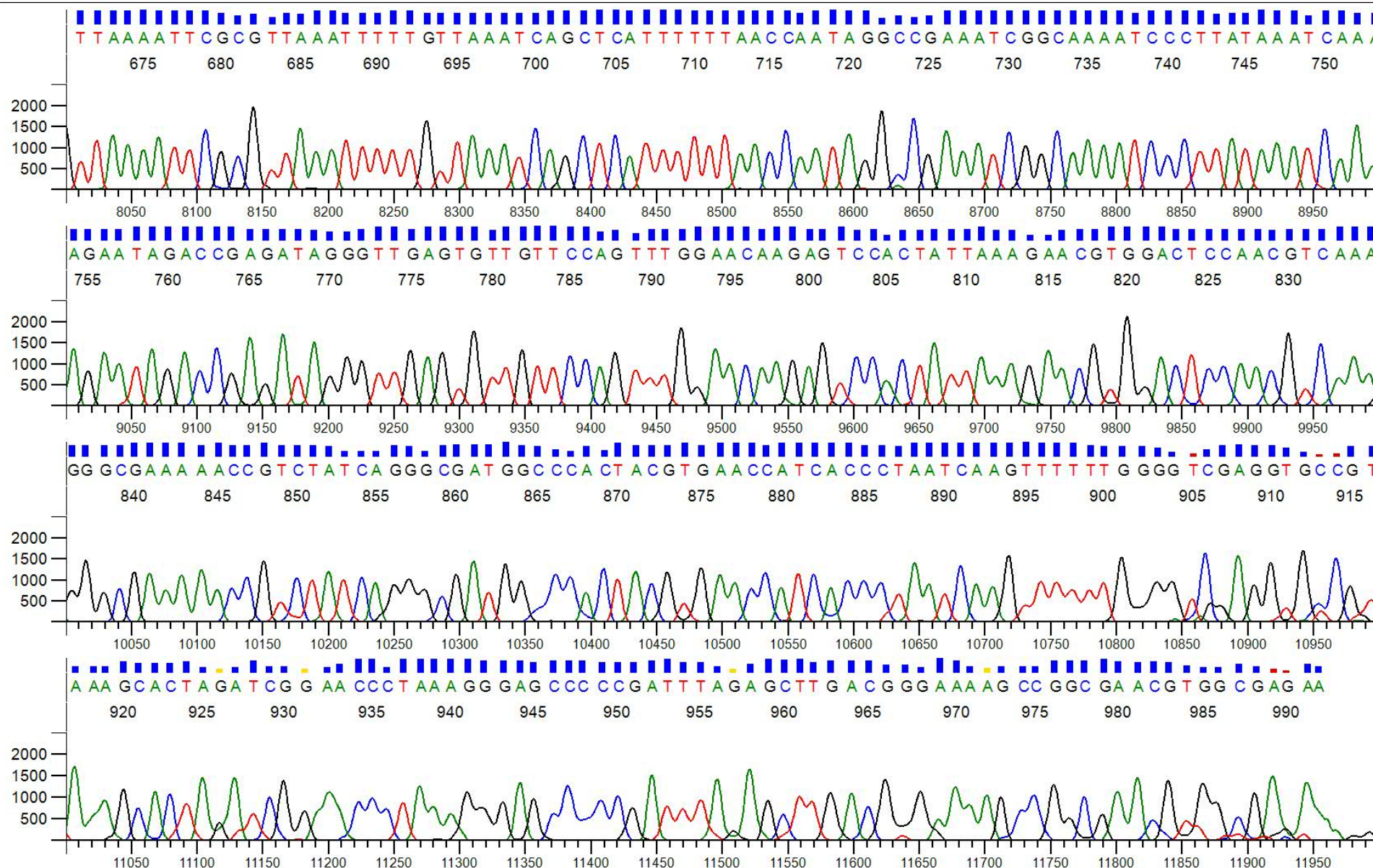

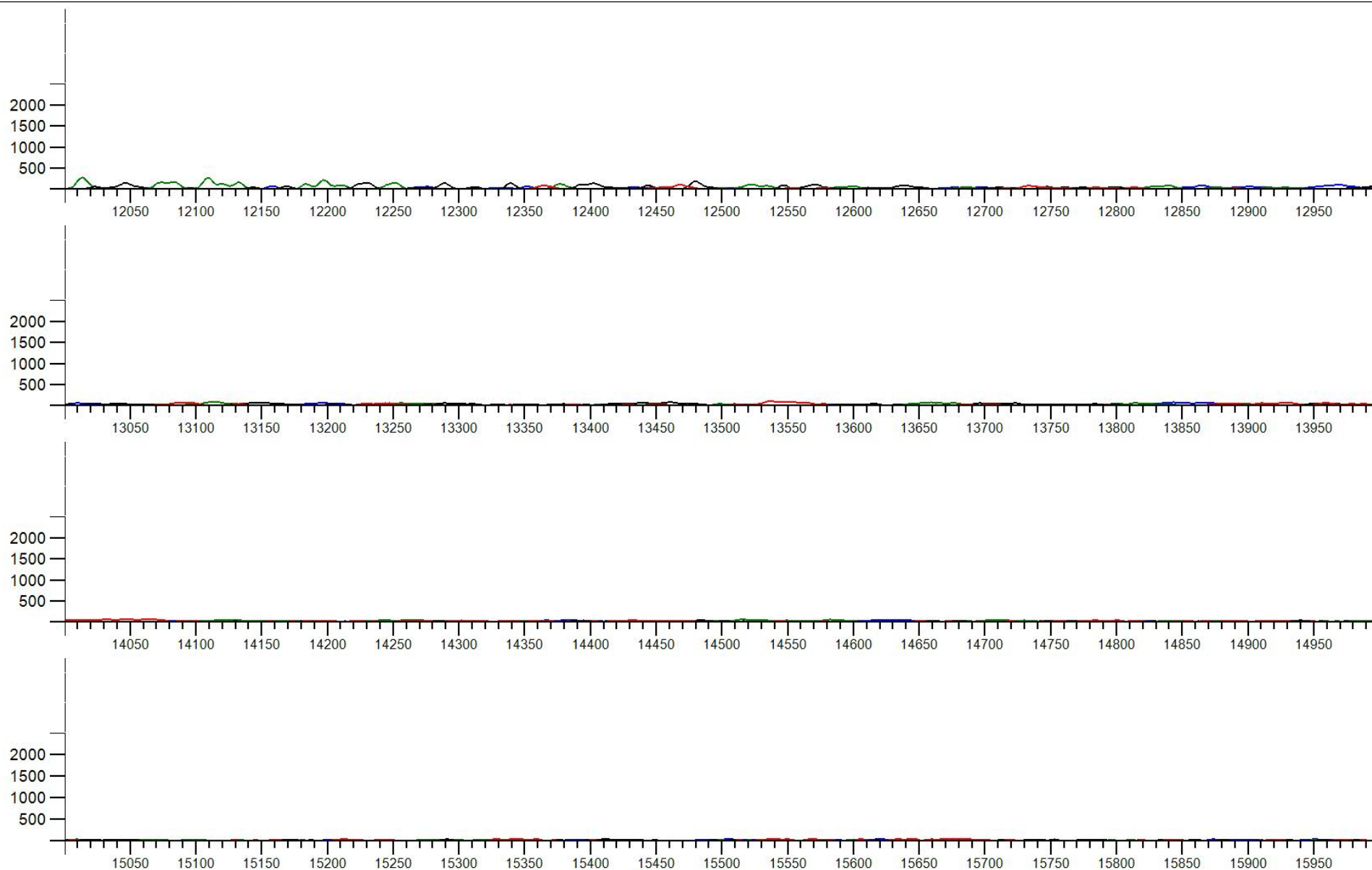

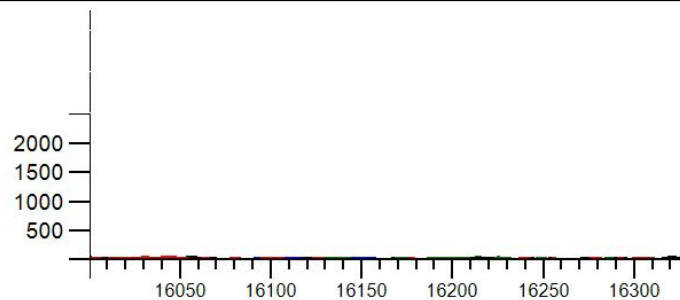

Supplement: Figure 3—source data 1. [file elife-69916-fig3-data1.zip › Figure 3A_Source data2_Bisulphite sequencing data_plasmid/SD-PDT1-BSF-3.17_T7FOR-G11.pdf]

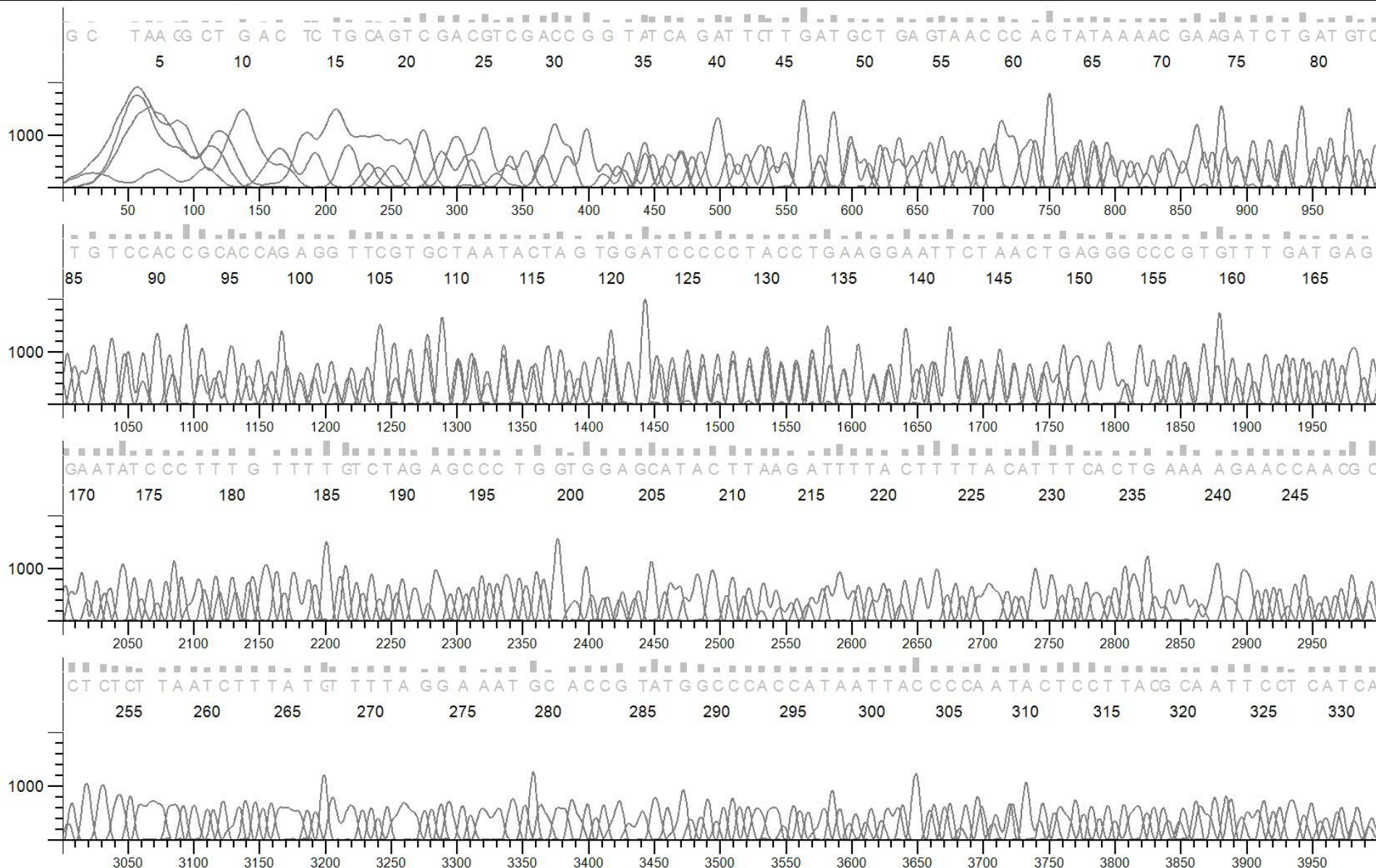

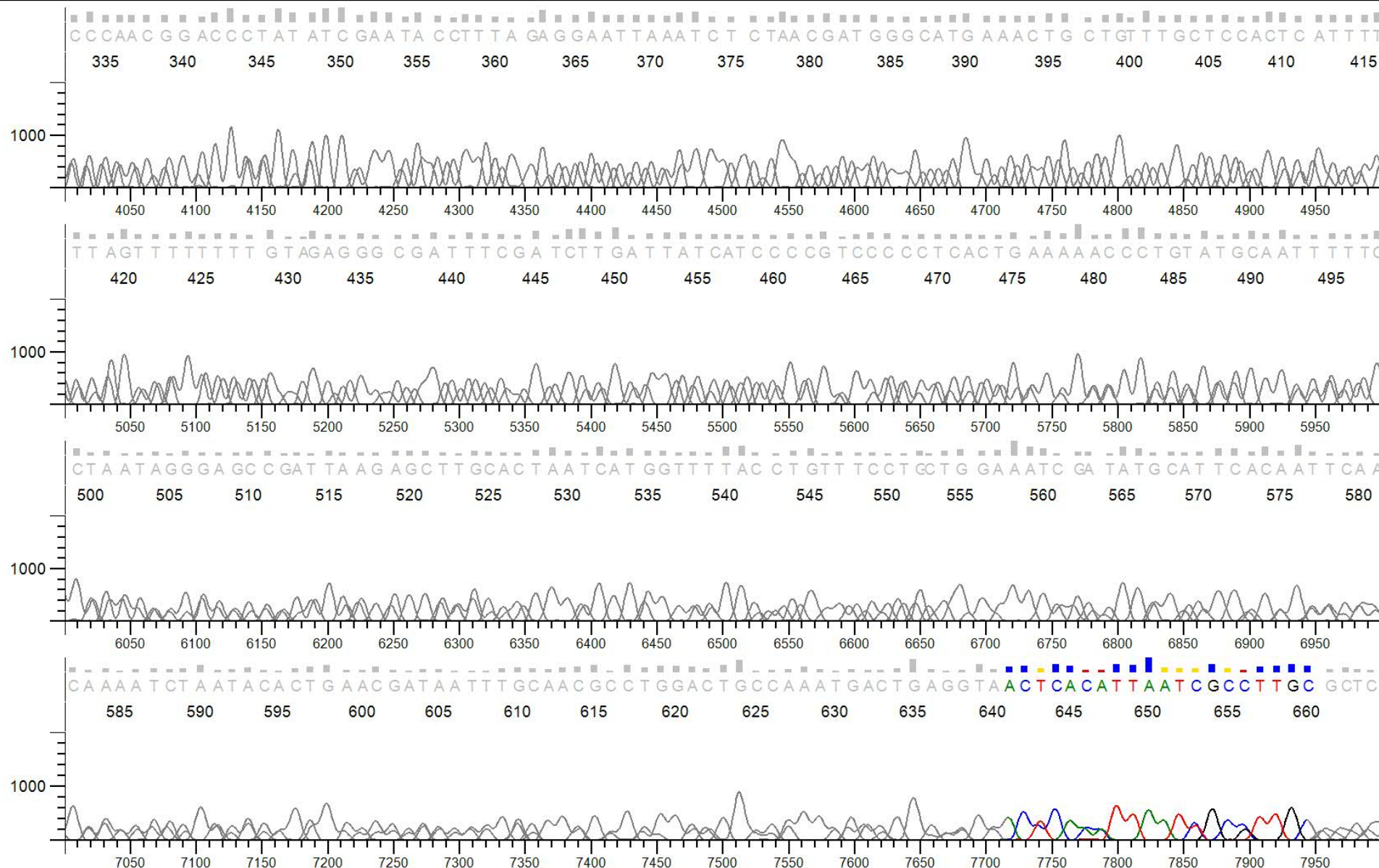

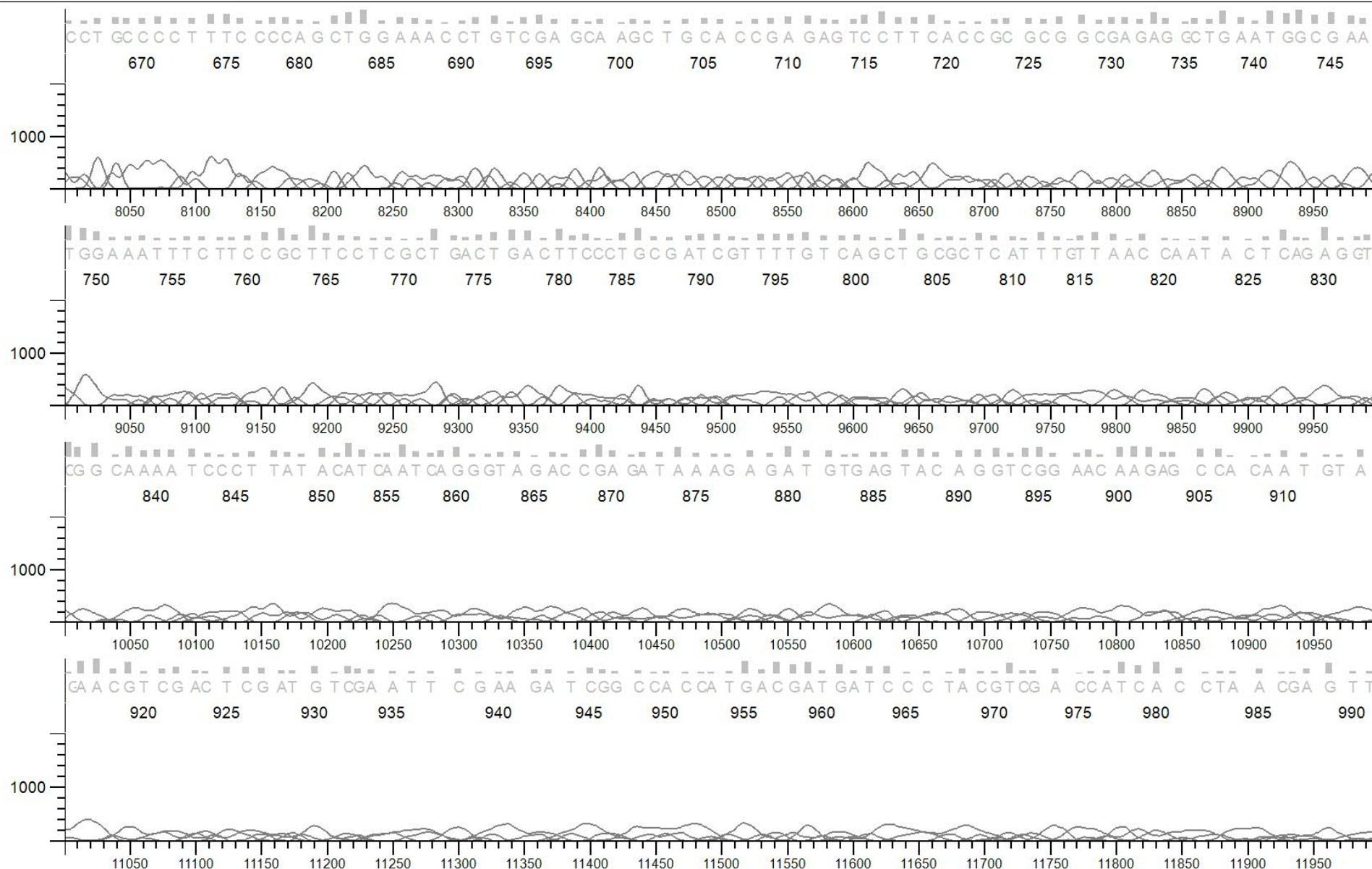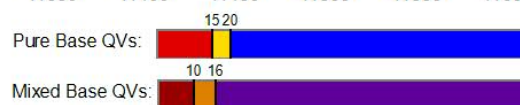

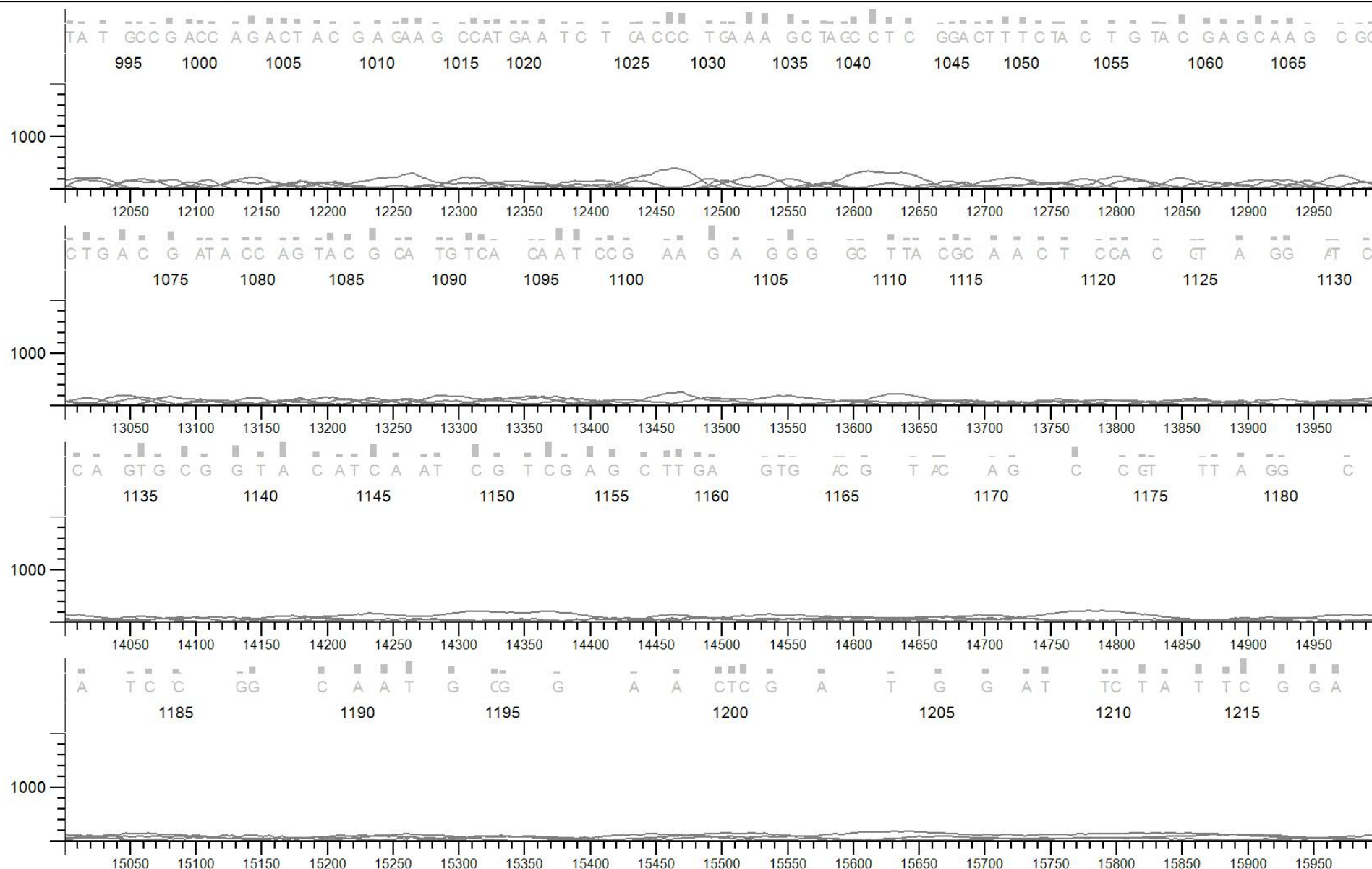

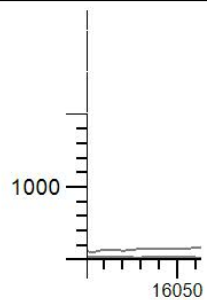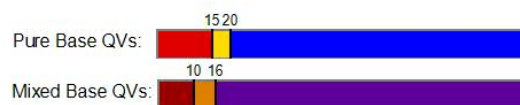

Supplement: Figure 3—source data 1. [file elife-69916-fig3-data1.zip › Figure 3A_Source data2_Bisulphite sequencing data_plasmid/SS4_PDI1_BIS_19_T7FOR-H01.pdf]

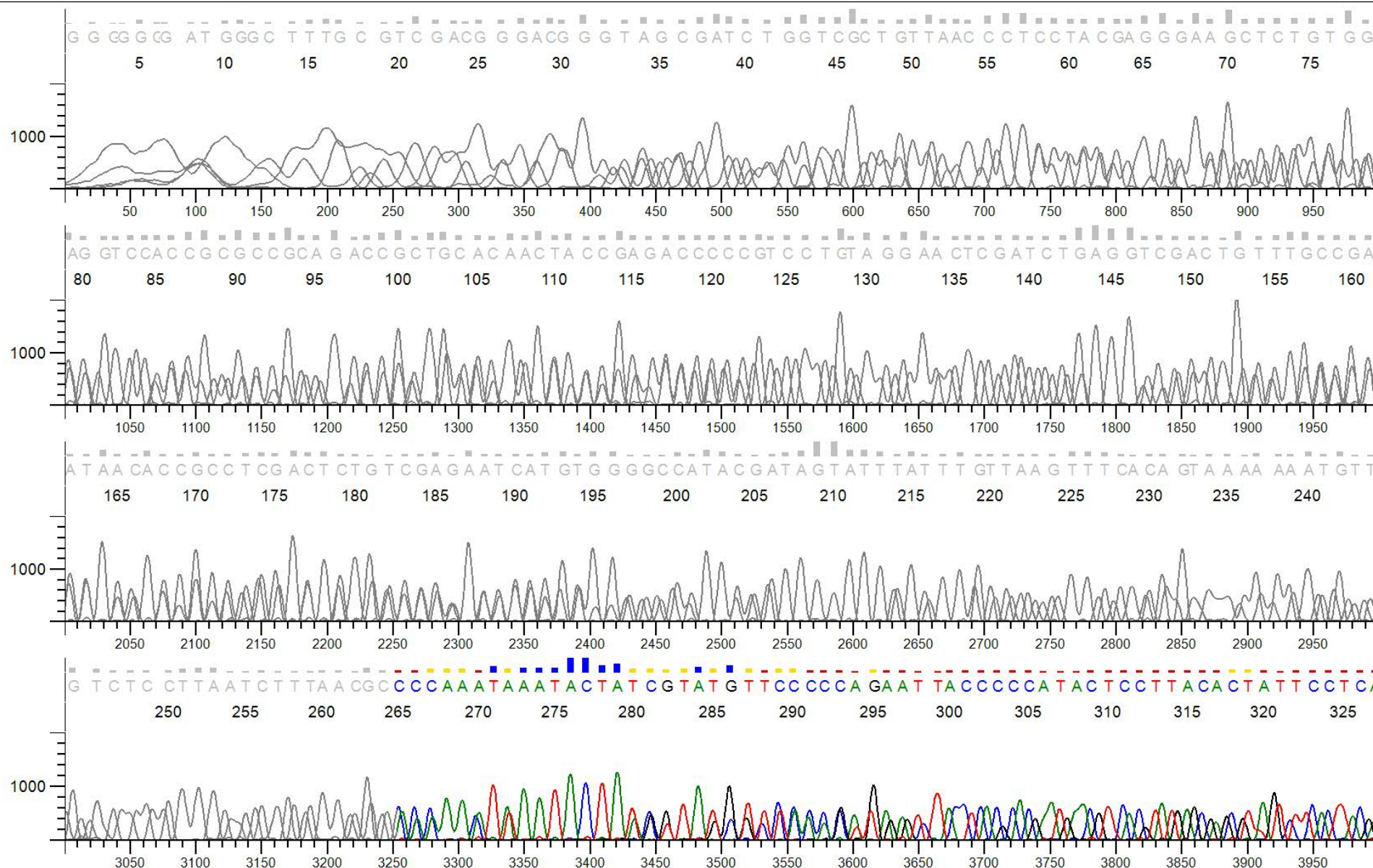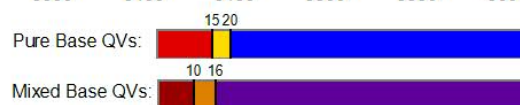

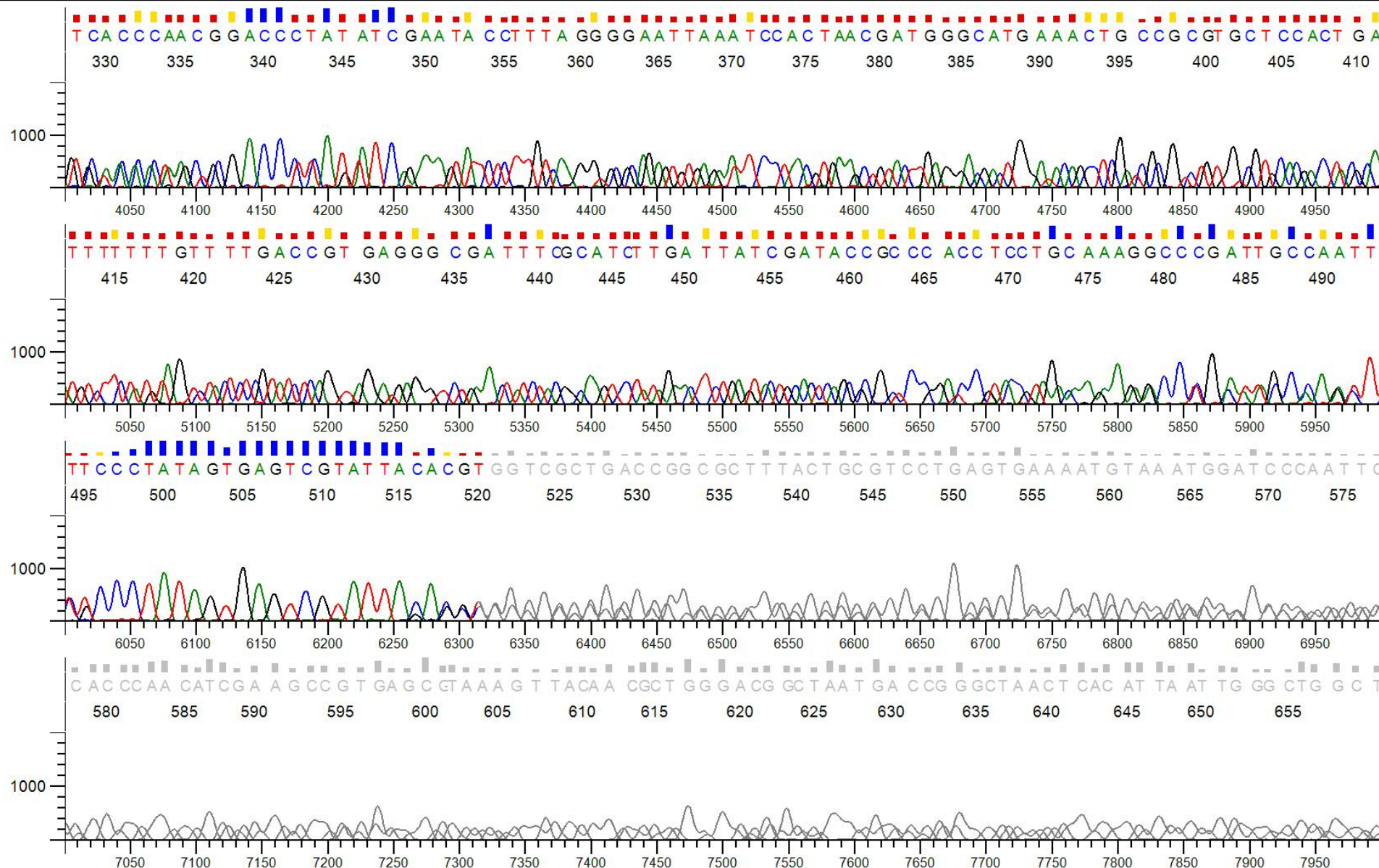

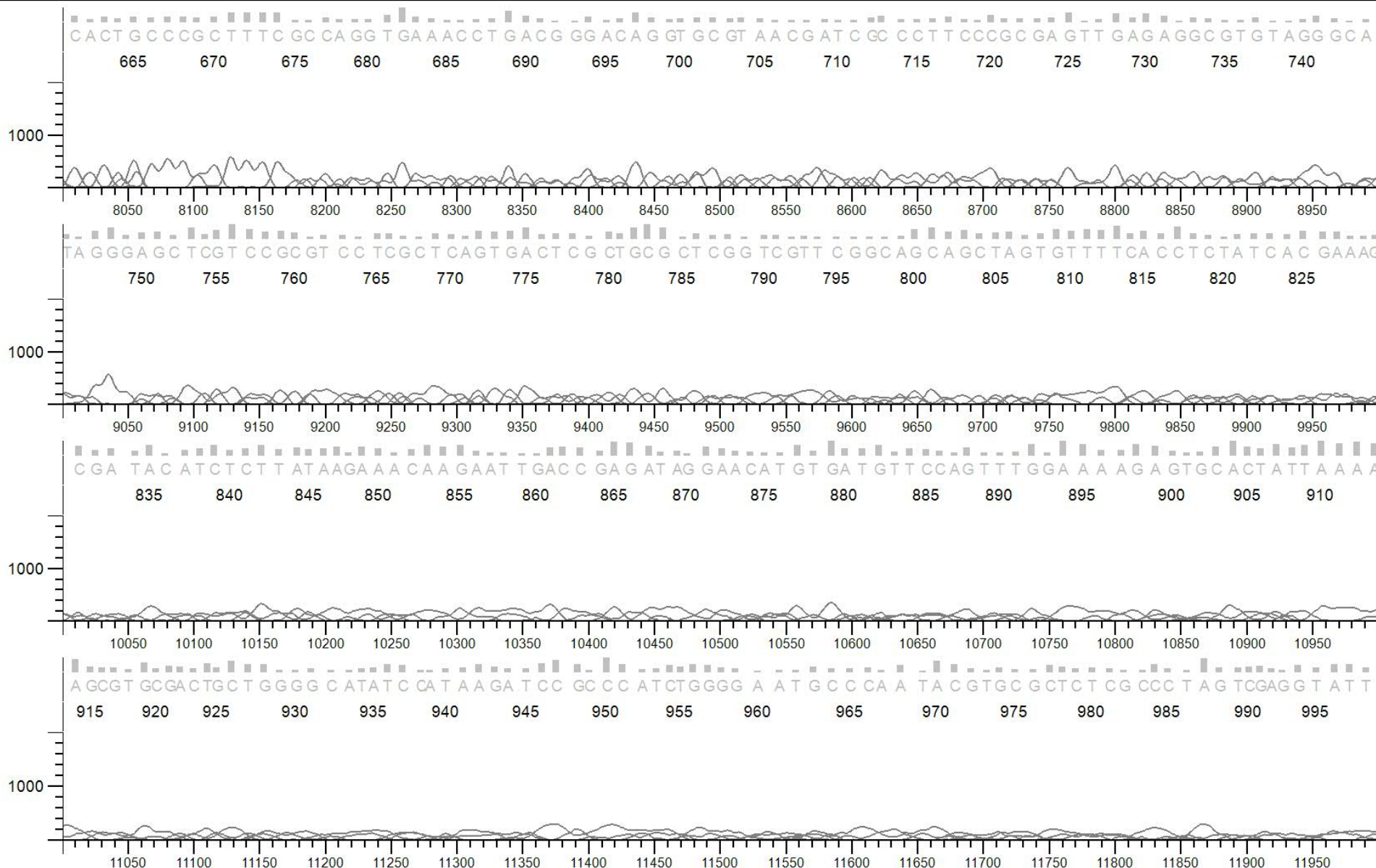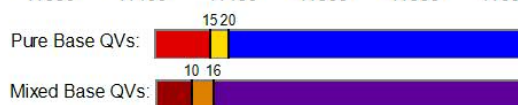

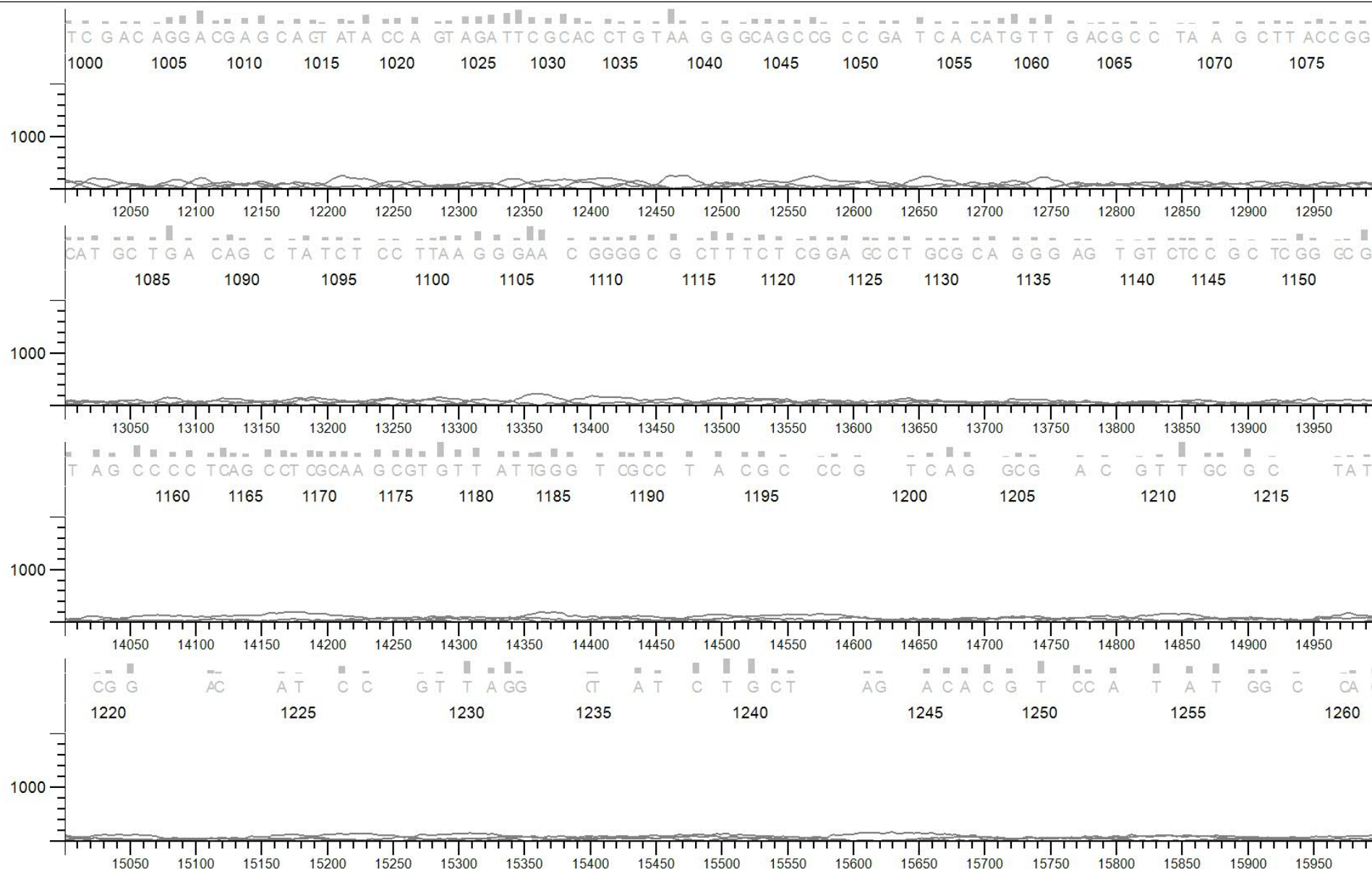

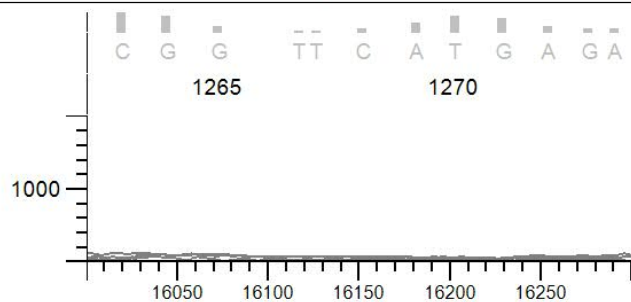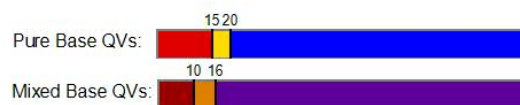

Supplement: Figure 3—source data 1. [file elife-69916-fig3-data1.zip › Figure 3A_Source data2_Bisulphite sequencing data_plasmid/SD_PDI1_BSF_2.3_T7FOR-F02.pdf]

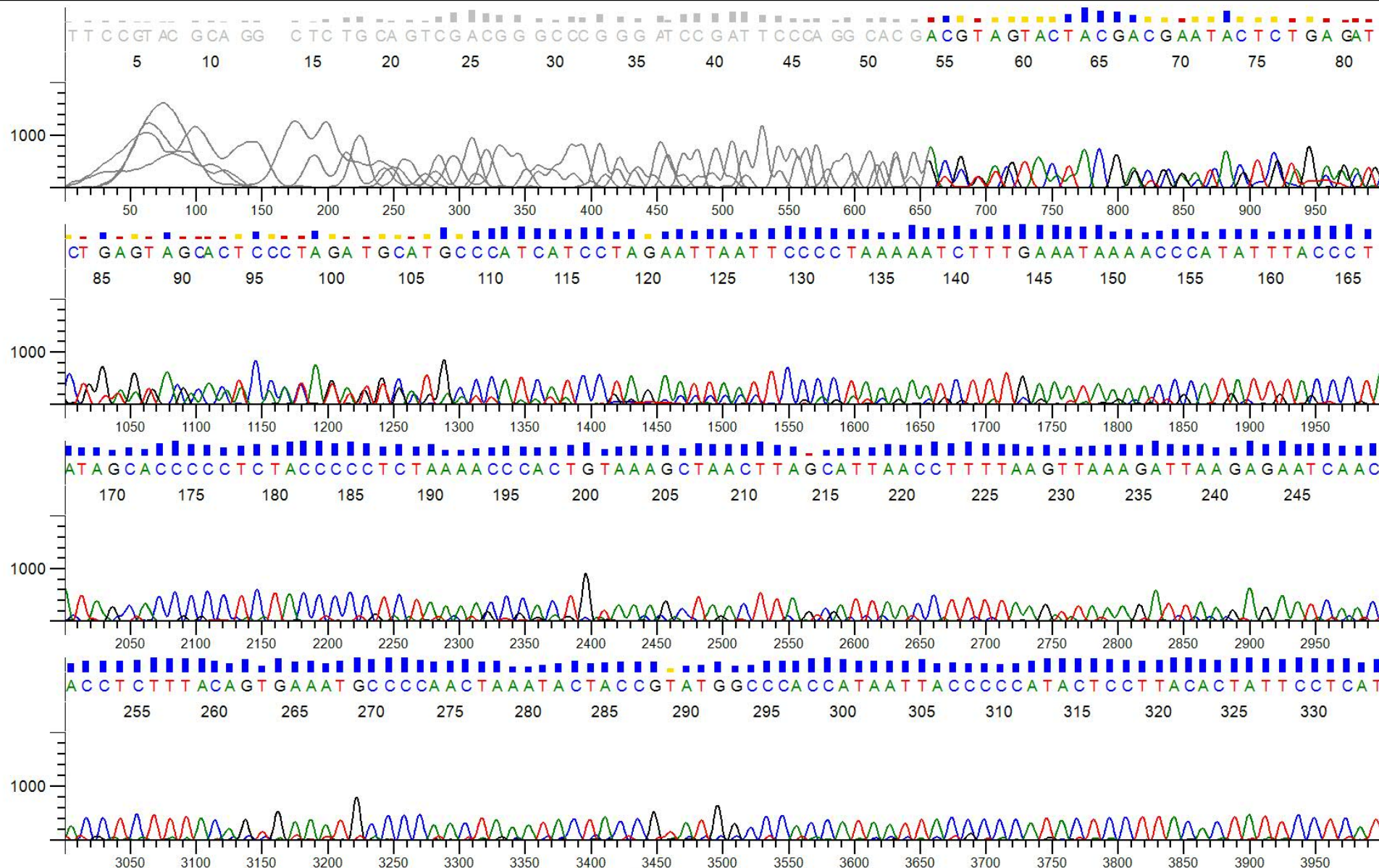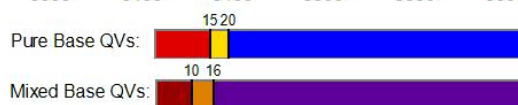

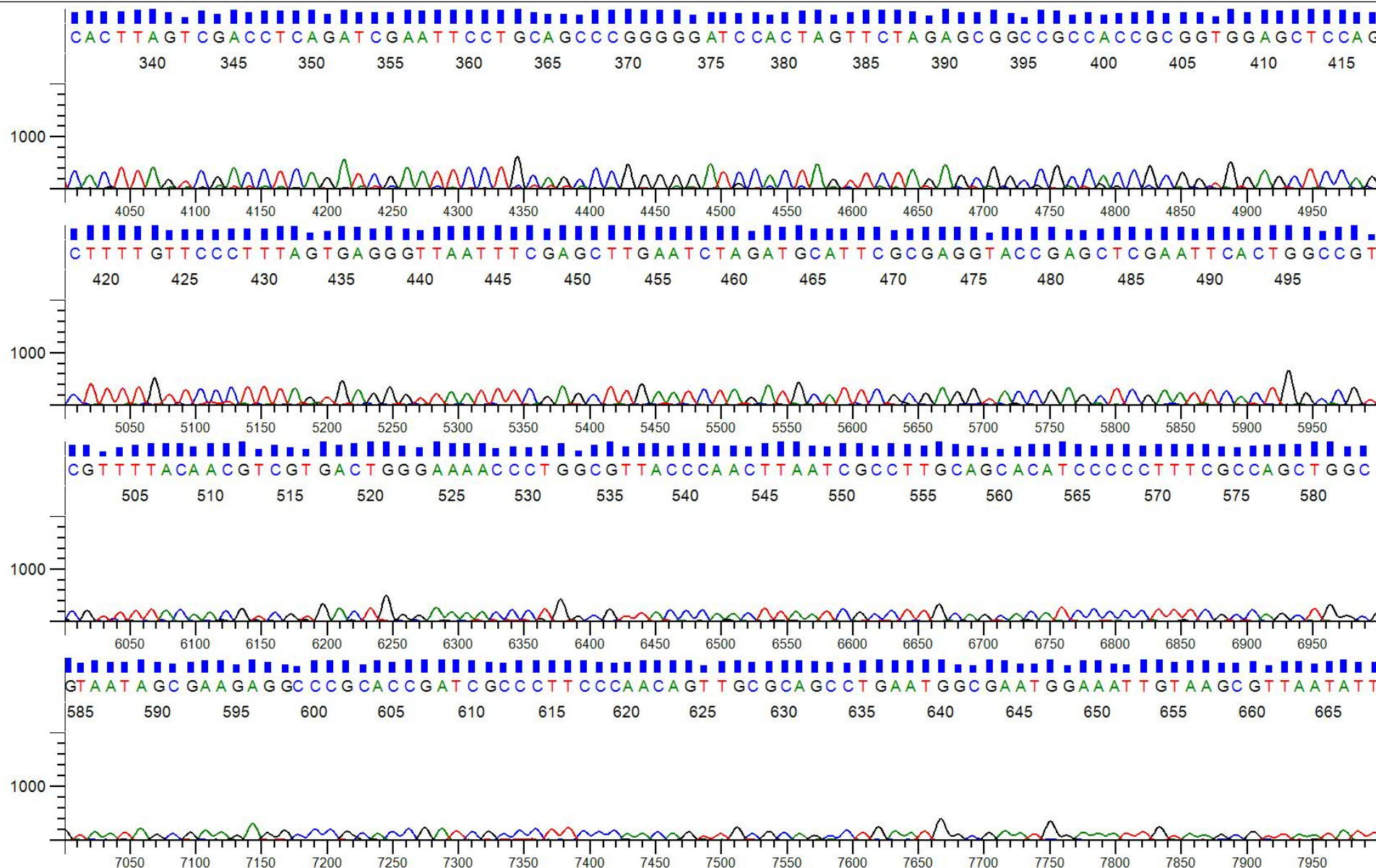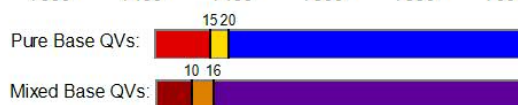

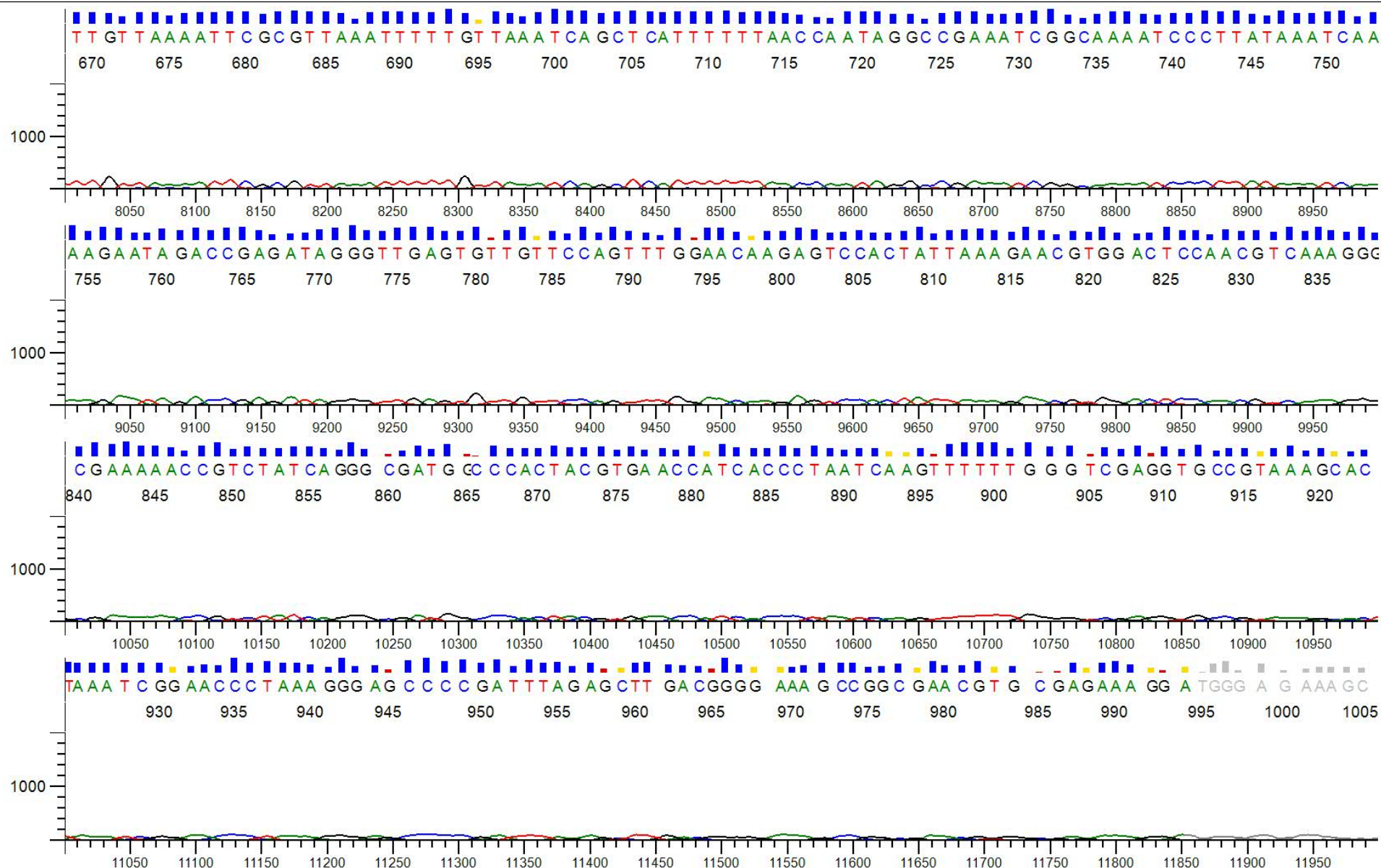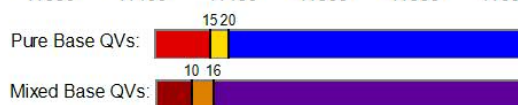

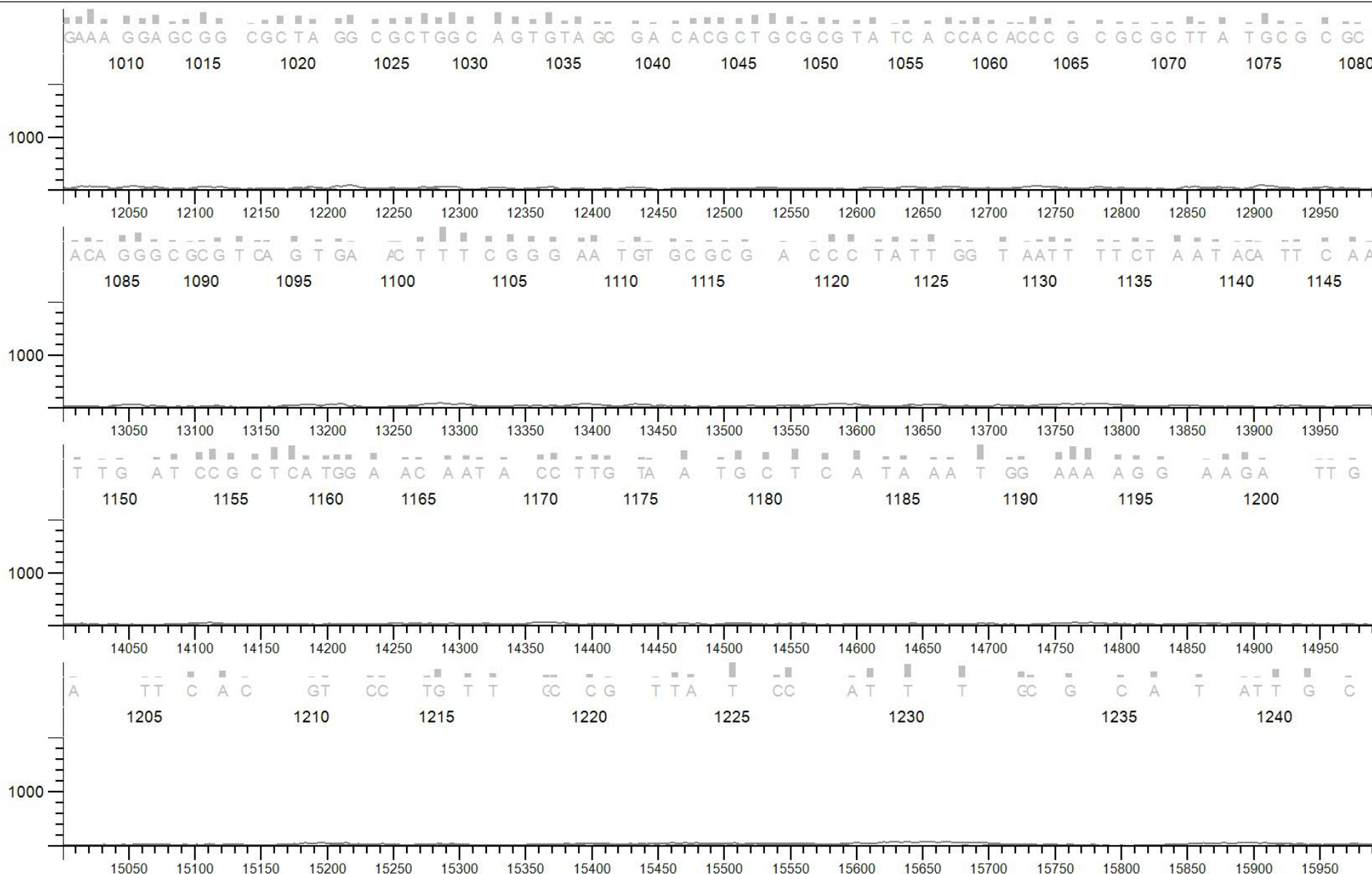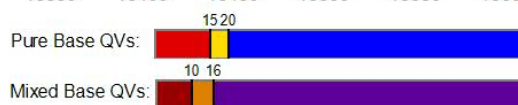

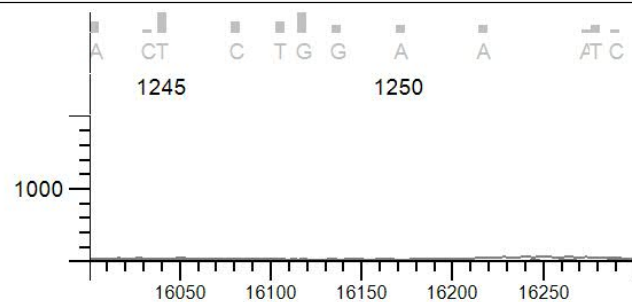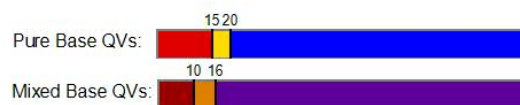

Supplement: Figure 3—source data 1. [file elife-69916-fig3-data1.zip › Figure 3A_Source data2_Bisulphite sequencing data_plasmid/SS4_PDI1_BIS_25_T7FOR-D01.pdf]

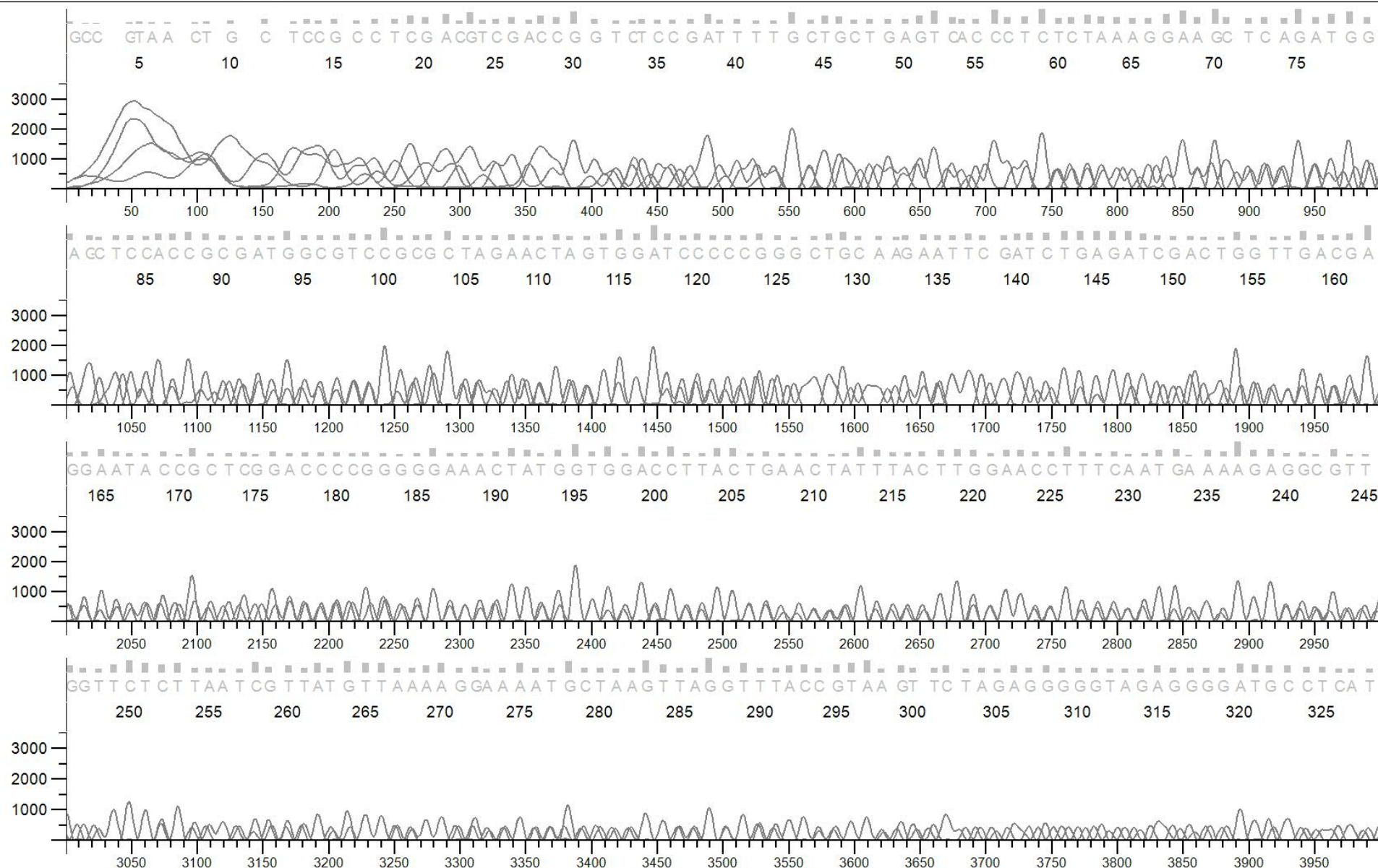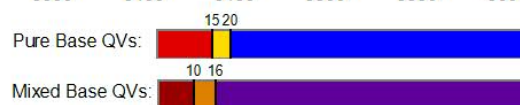

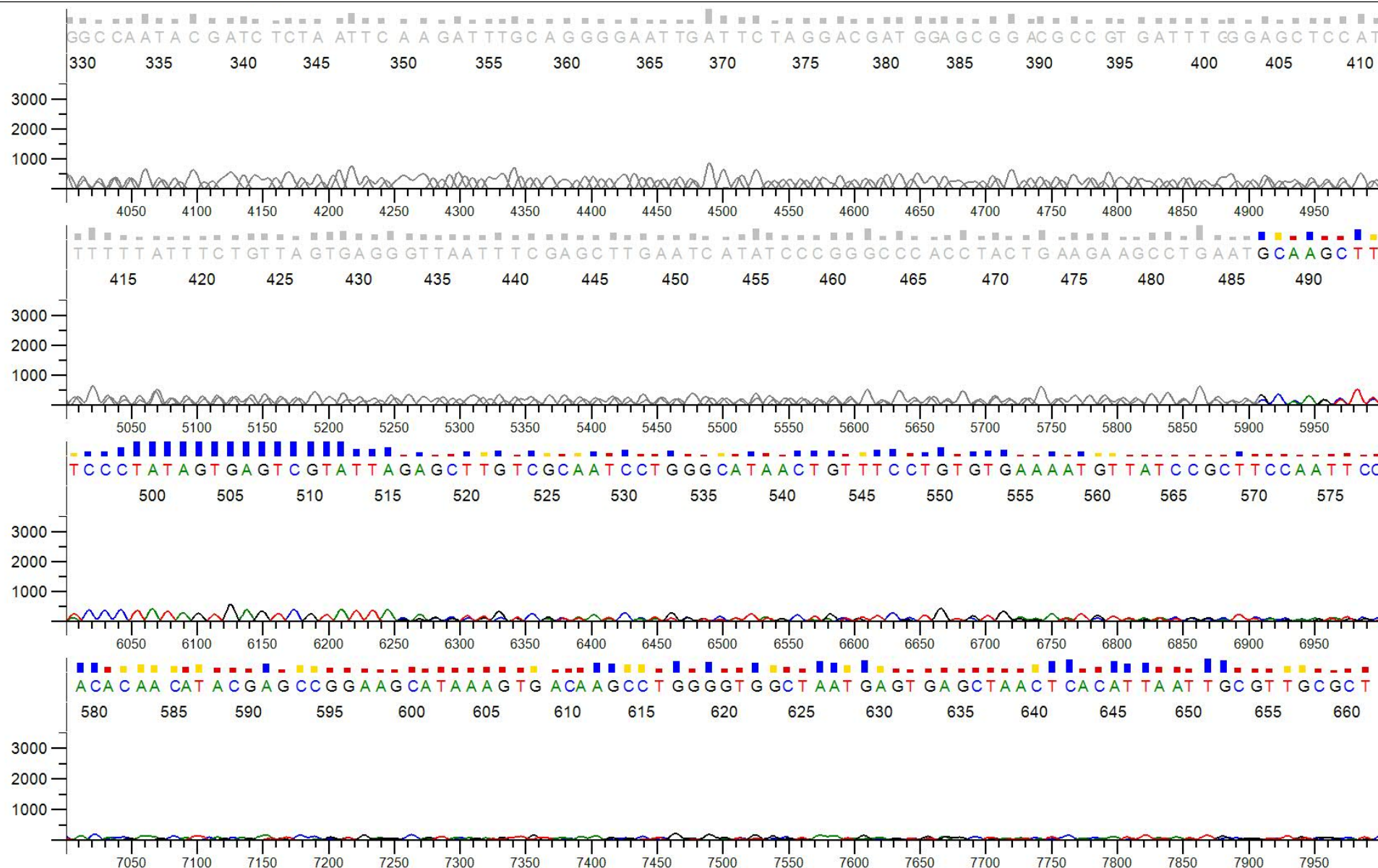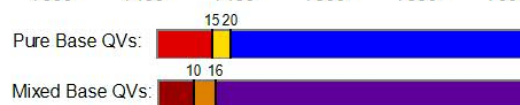

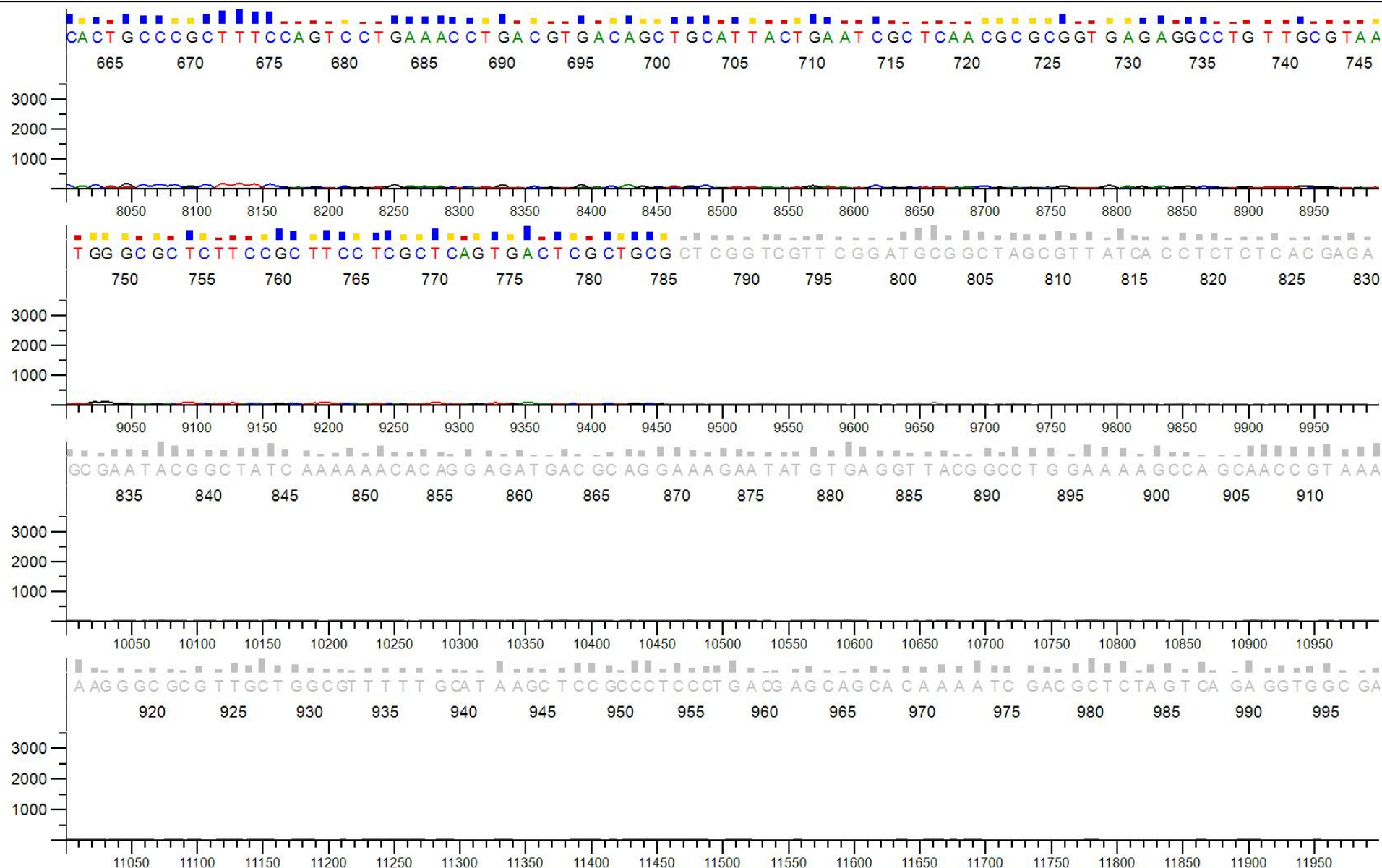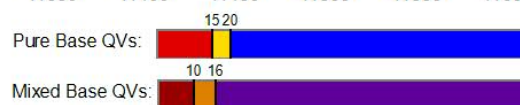

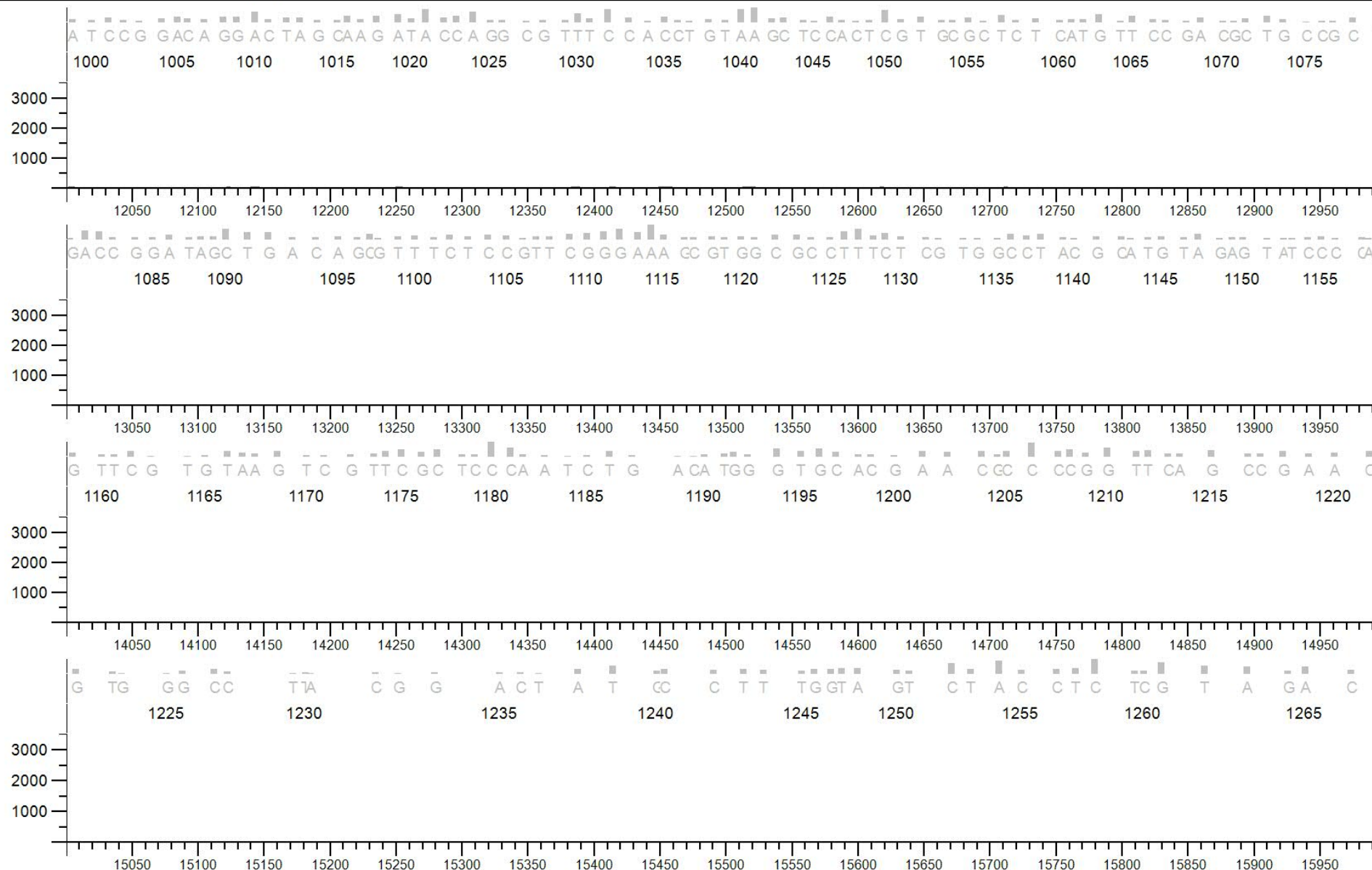

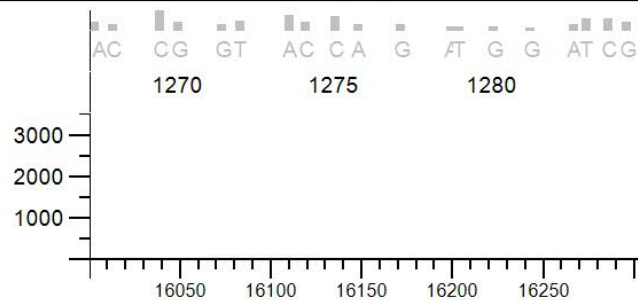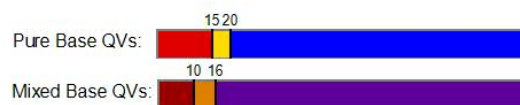

Supplement: Figure 3—source data 1. [file elife-69916-fig3-data1.zip › Figure 3A_Source data2_Bisulphite sequencing data_plasmid/SD-PDI1-BSF-1.2_T7-FOR-B01.pdf]

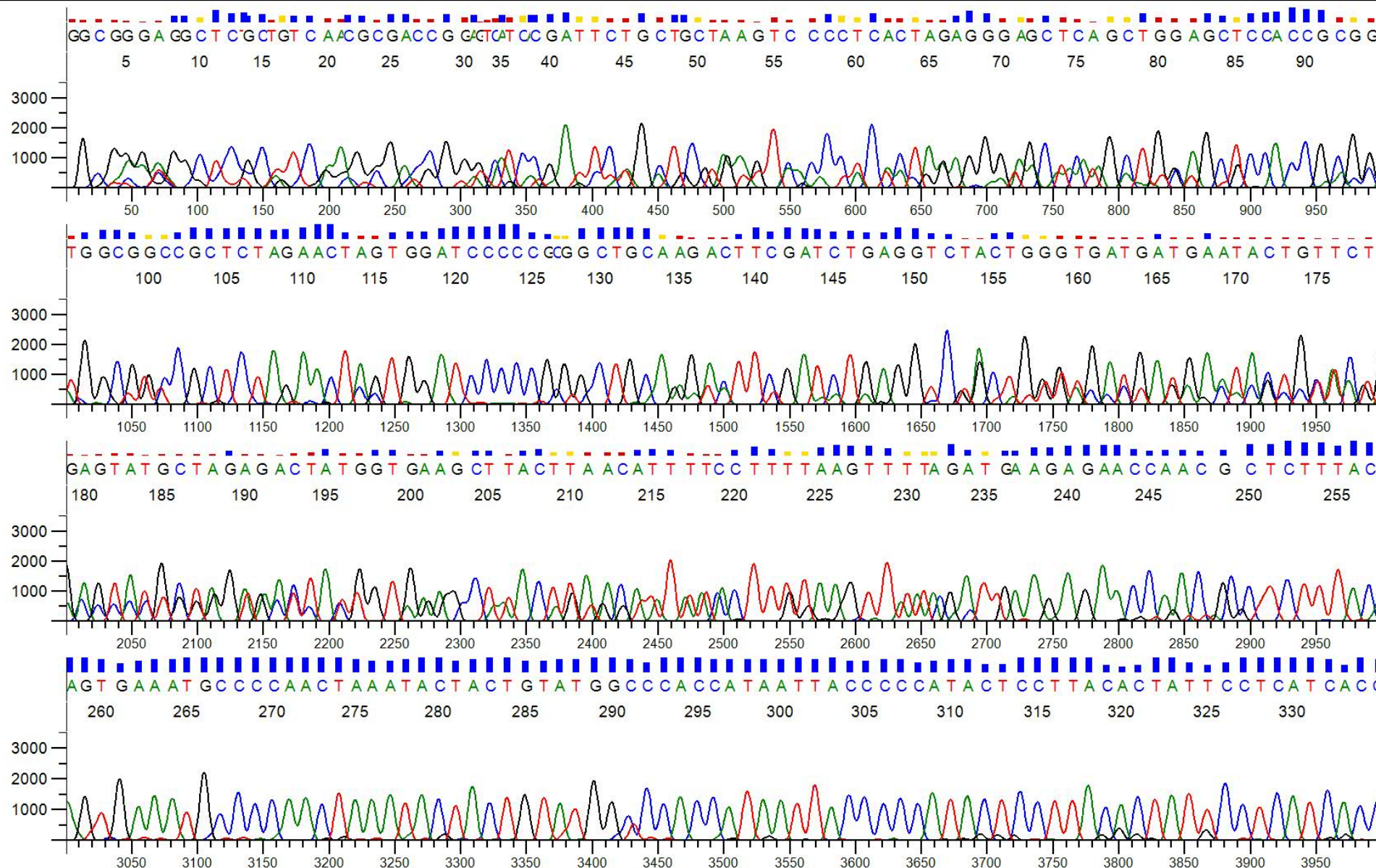

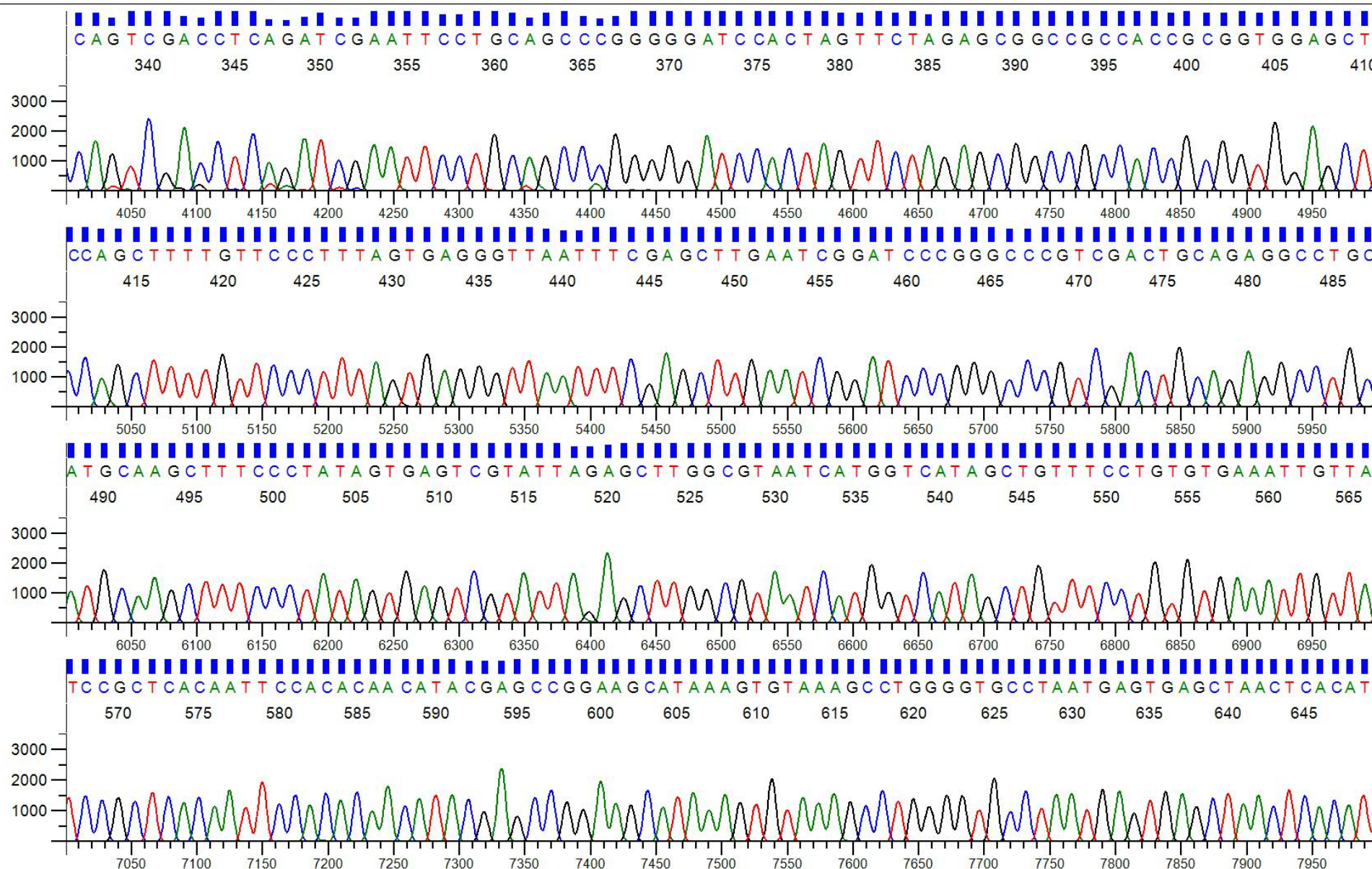

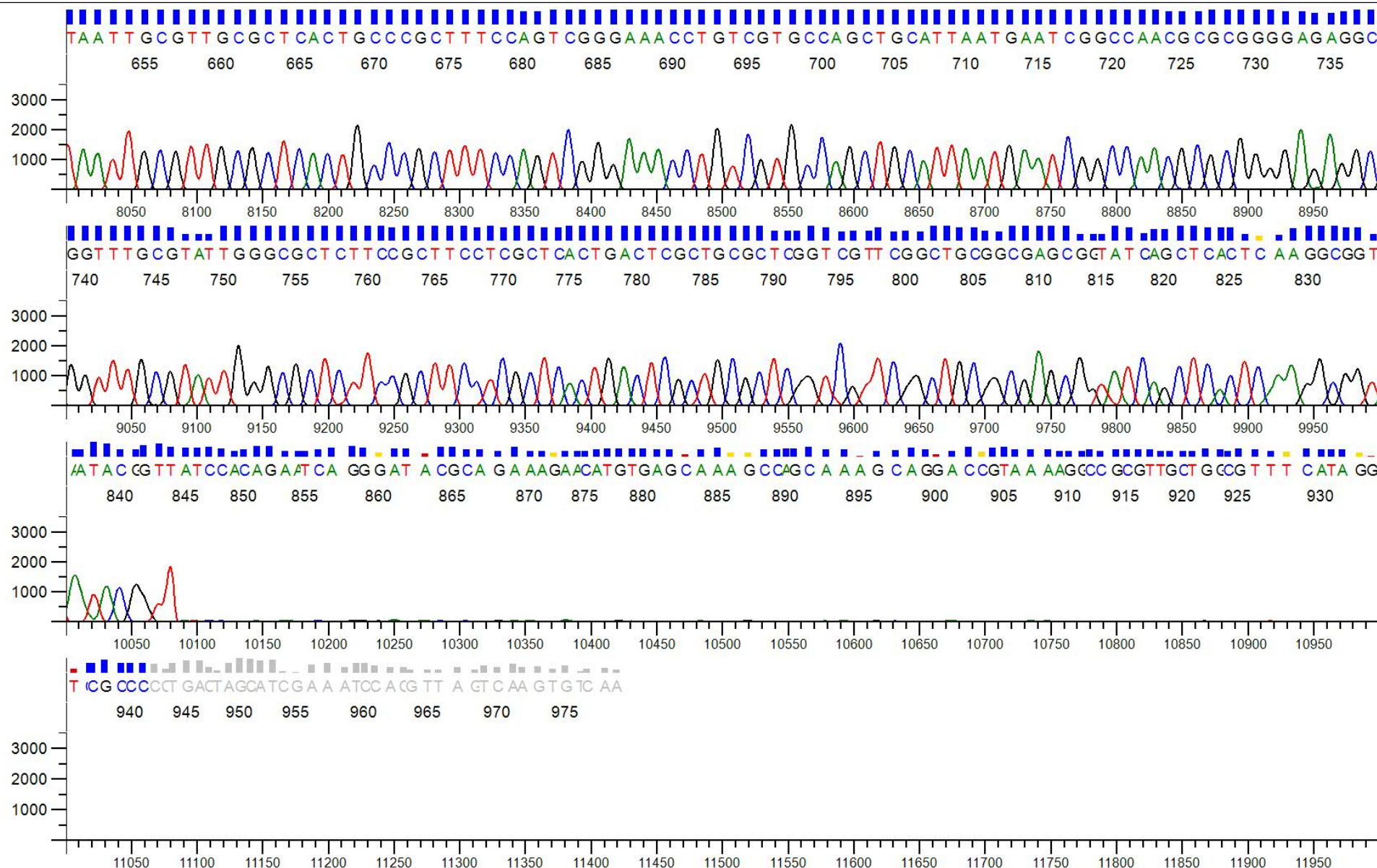

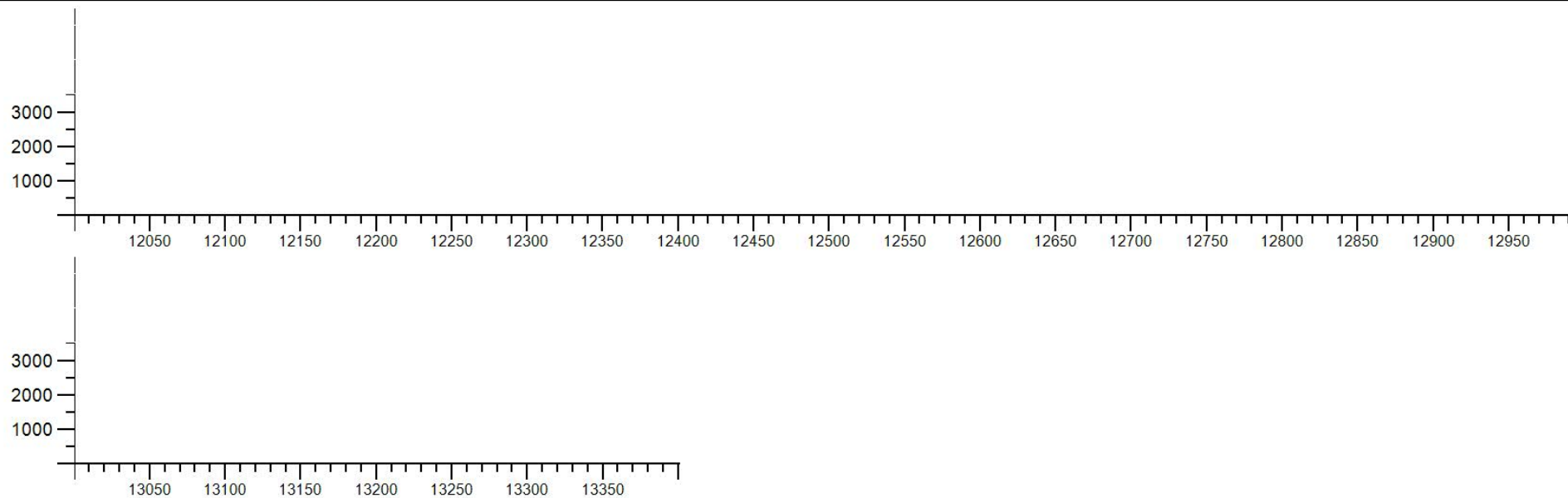

Supplement: Figure 3—source data 1. [file elife-69916-fig3-data1.zip › Figure 3A_Source data2_Bisulphite sequencing data_plasmid/RBK_PDI1-BSF_1.8_T7_FOR.pdf]

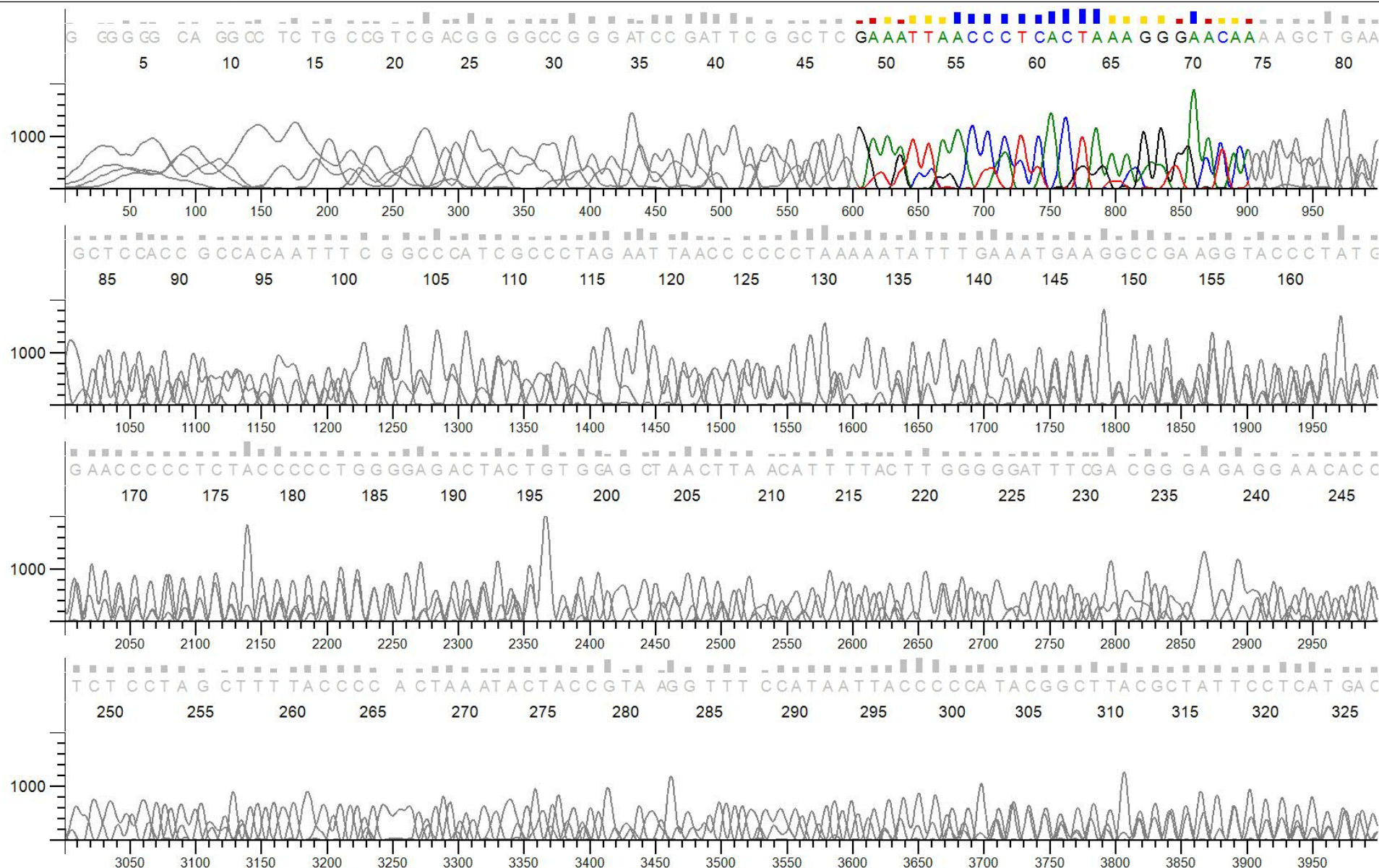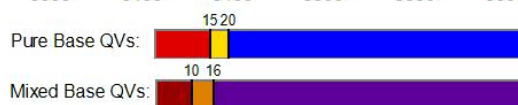

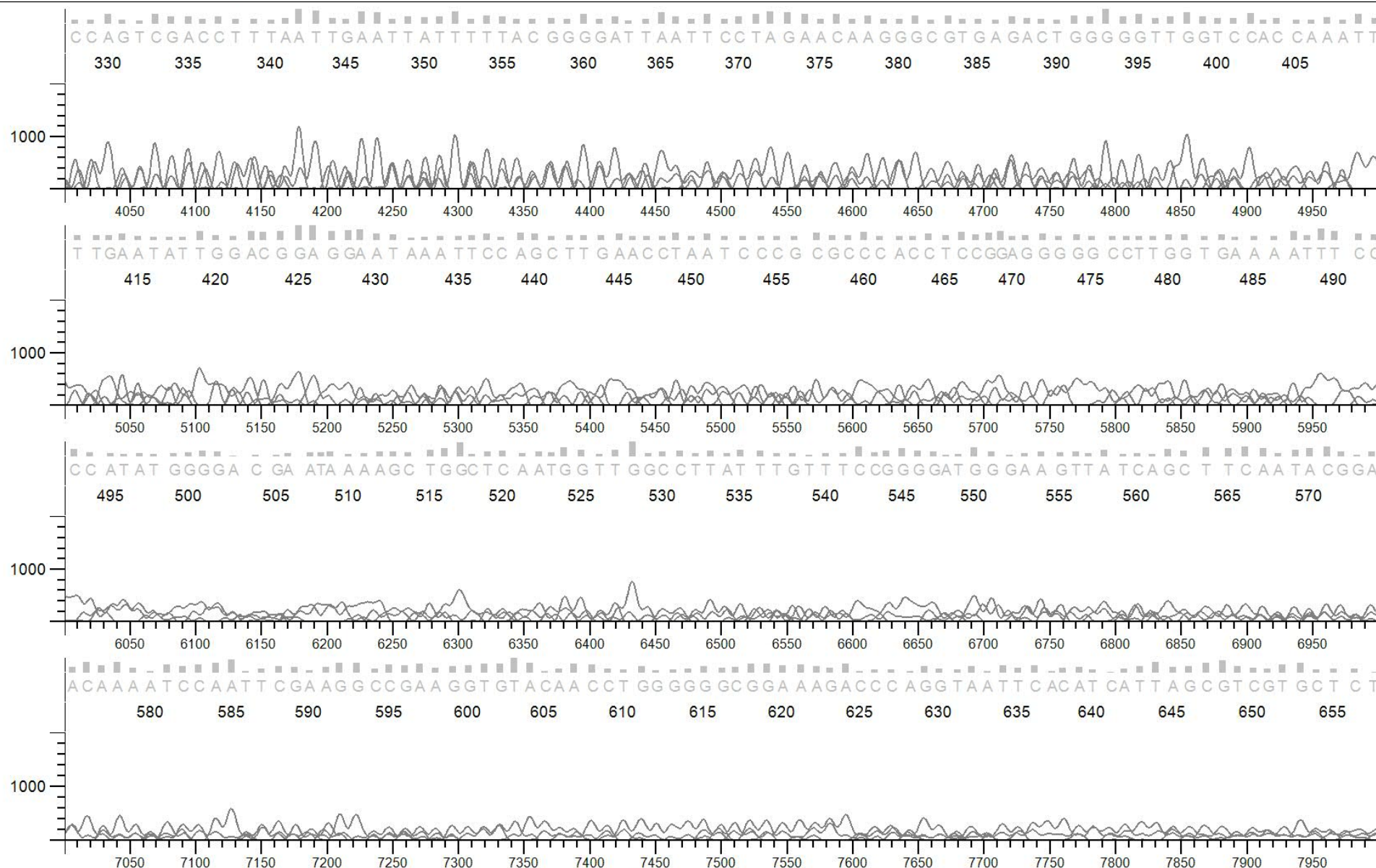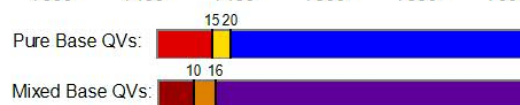

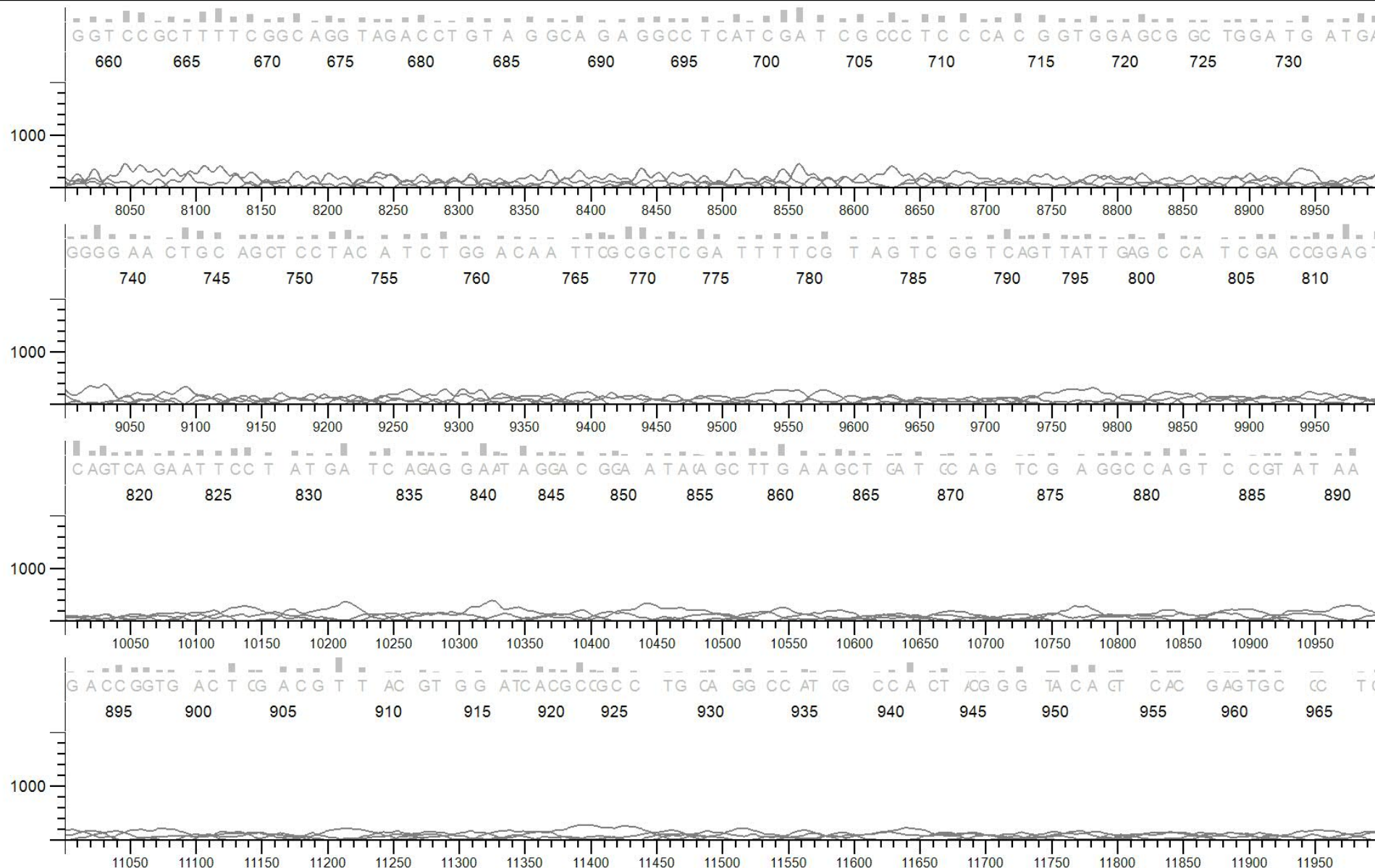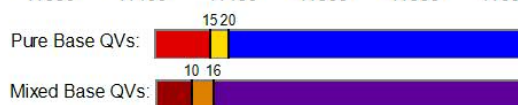

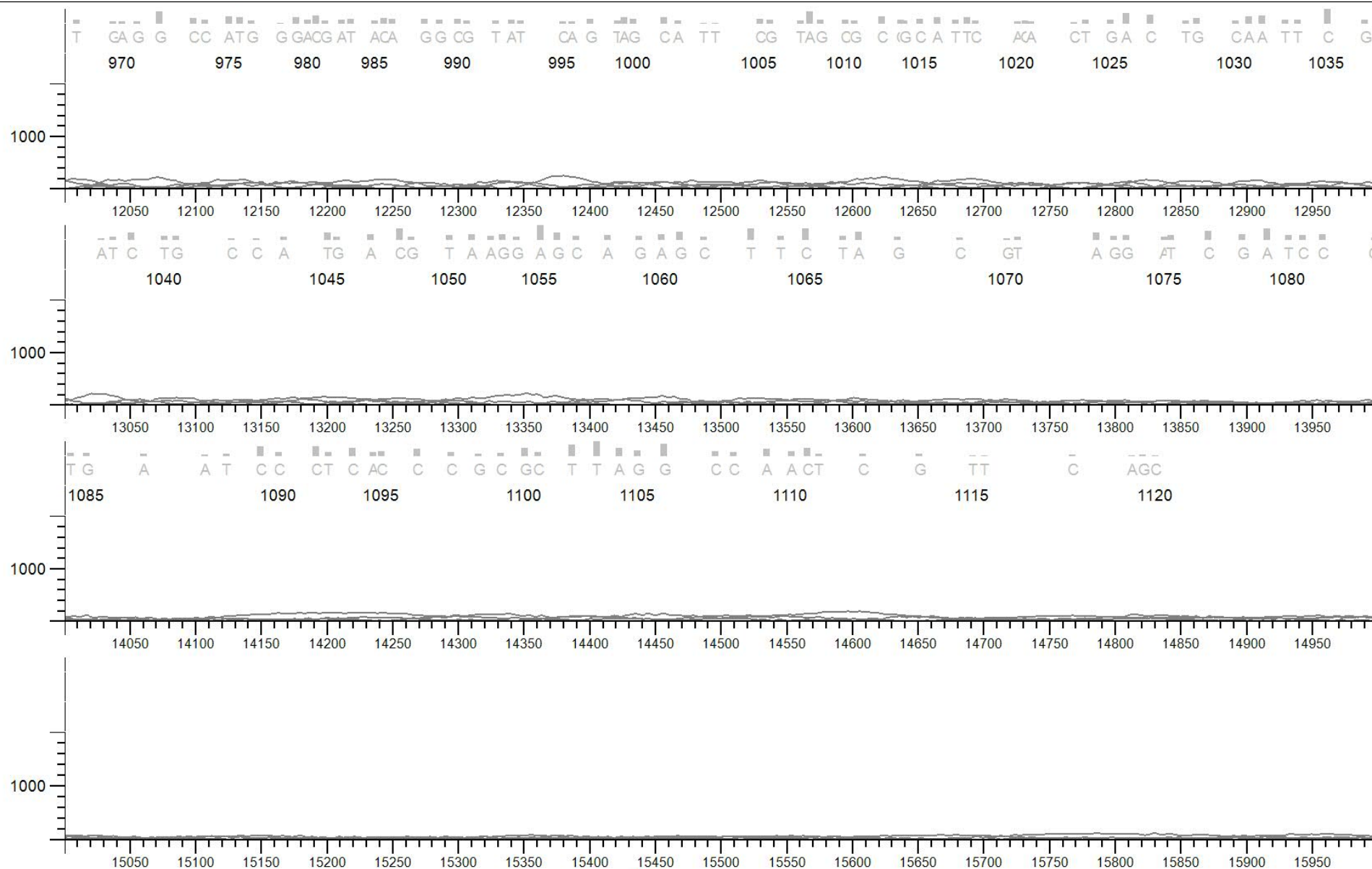

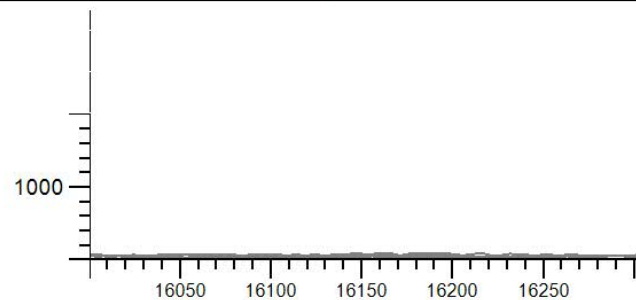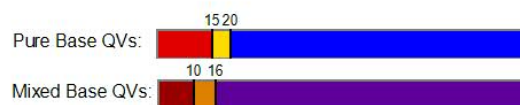

Supplement: Figure 3—source data 1. [file elife-69916-fig3-data1.zip › Figure 3A_Source data2_Bisulphite sequencing data_plasmid/RBK_PDI1_BSF_1.4_T7FOR.pdf]

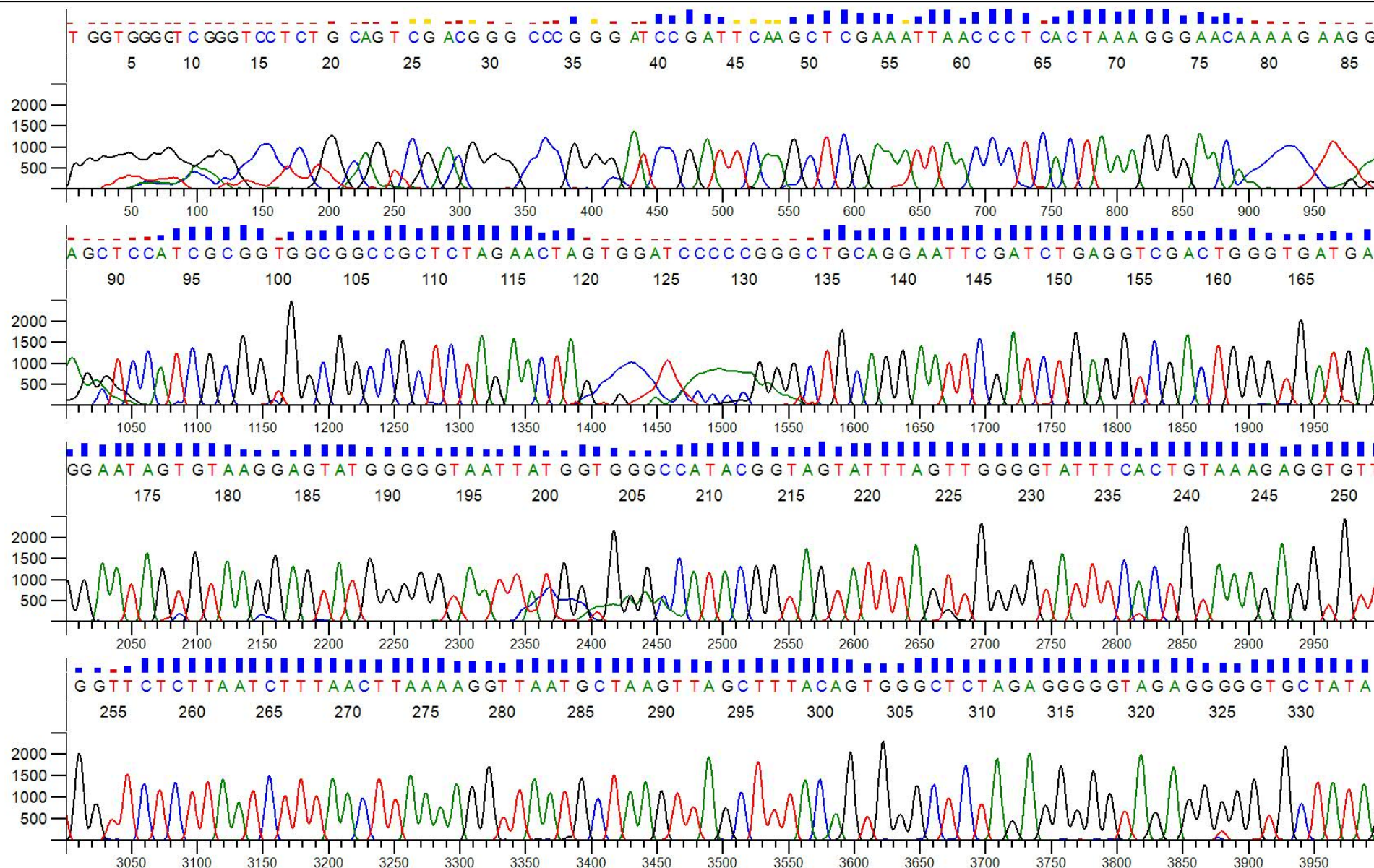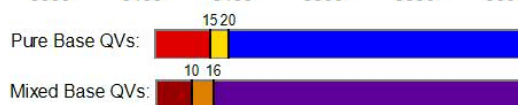

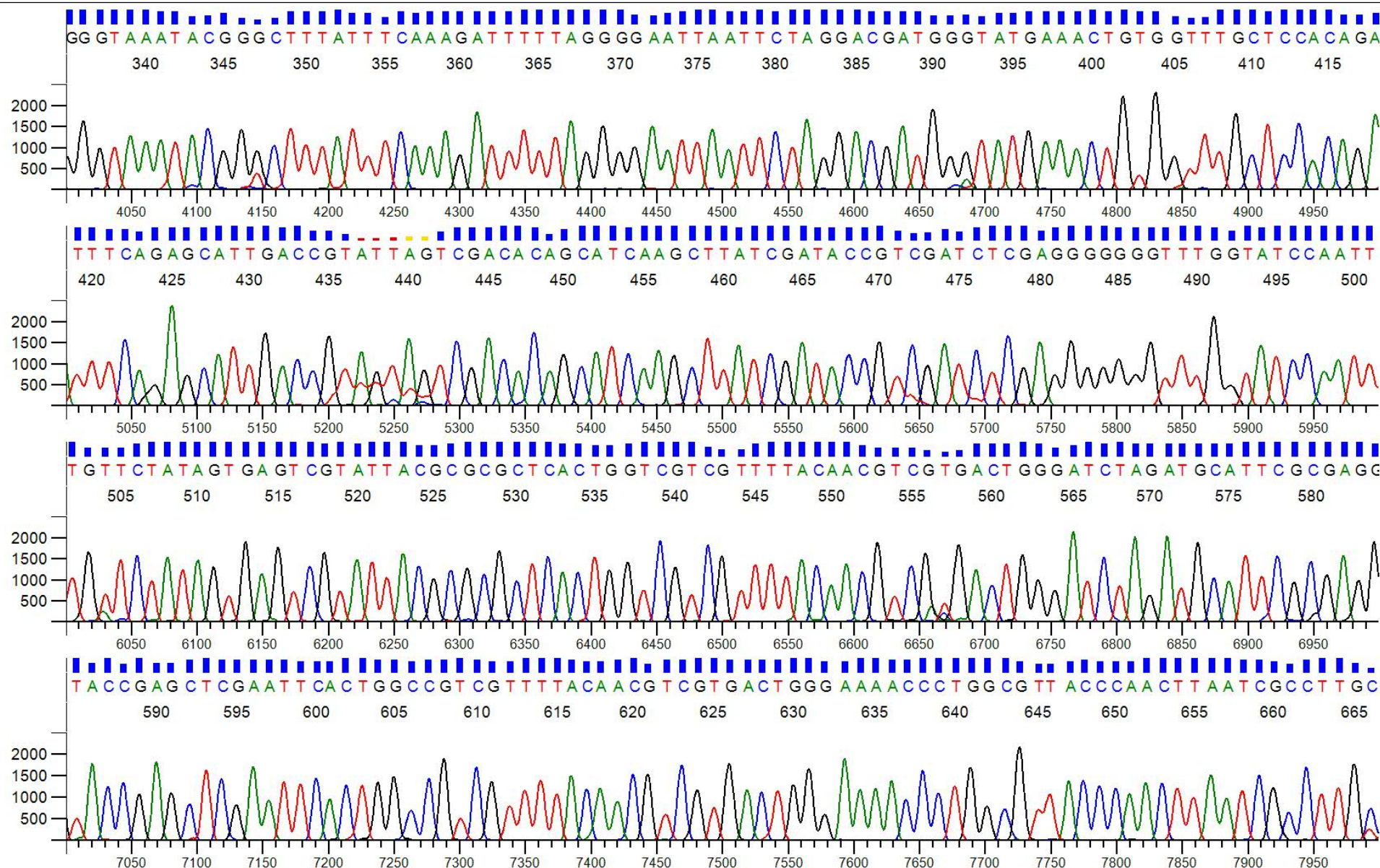

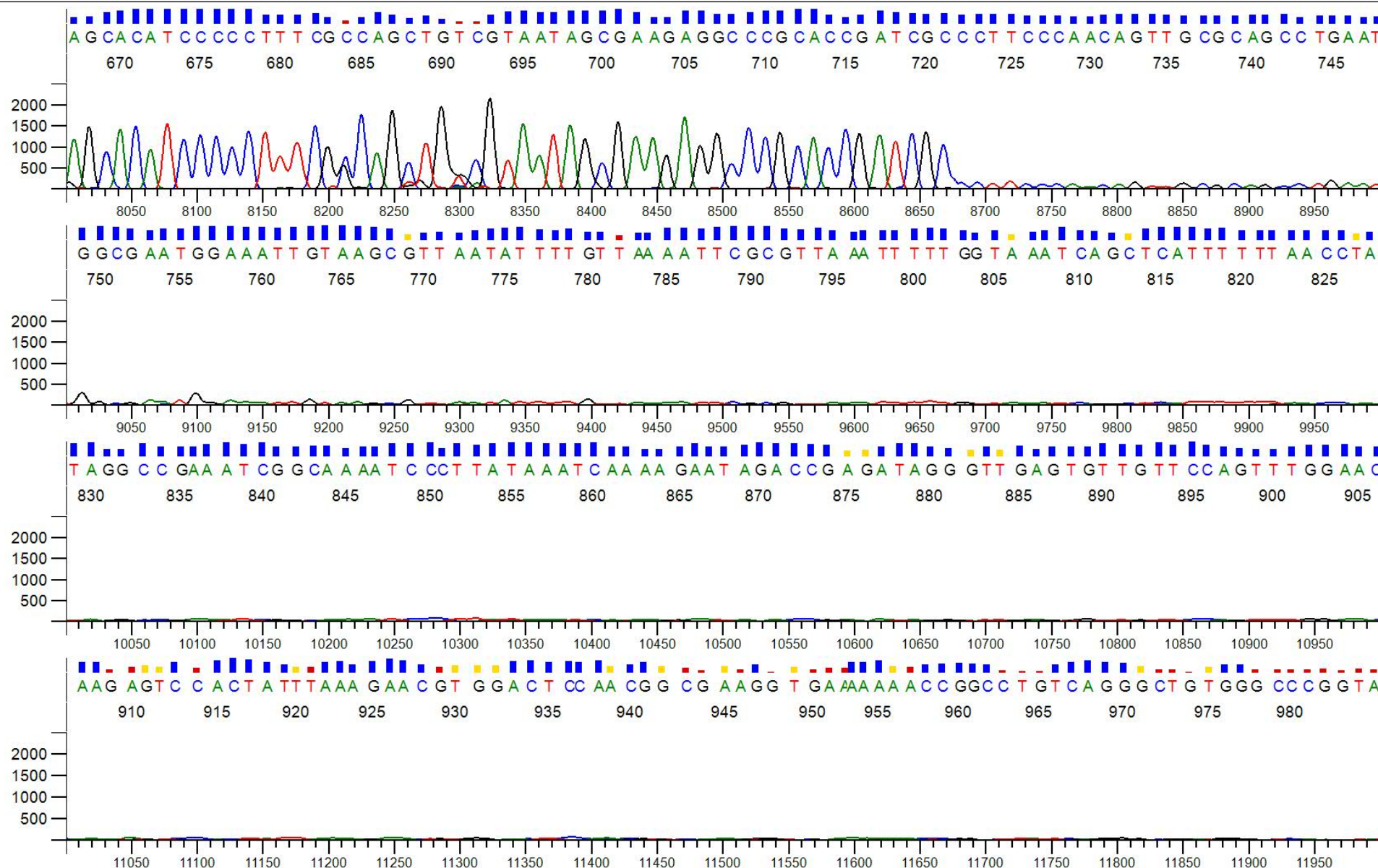

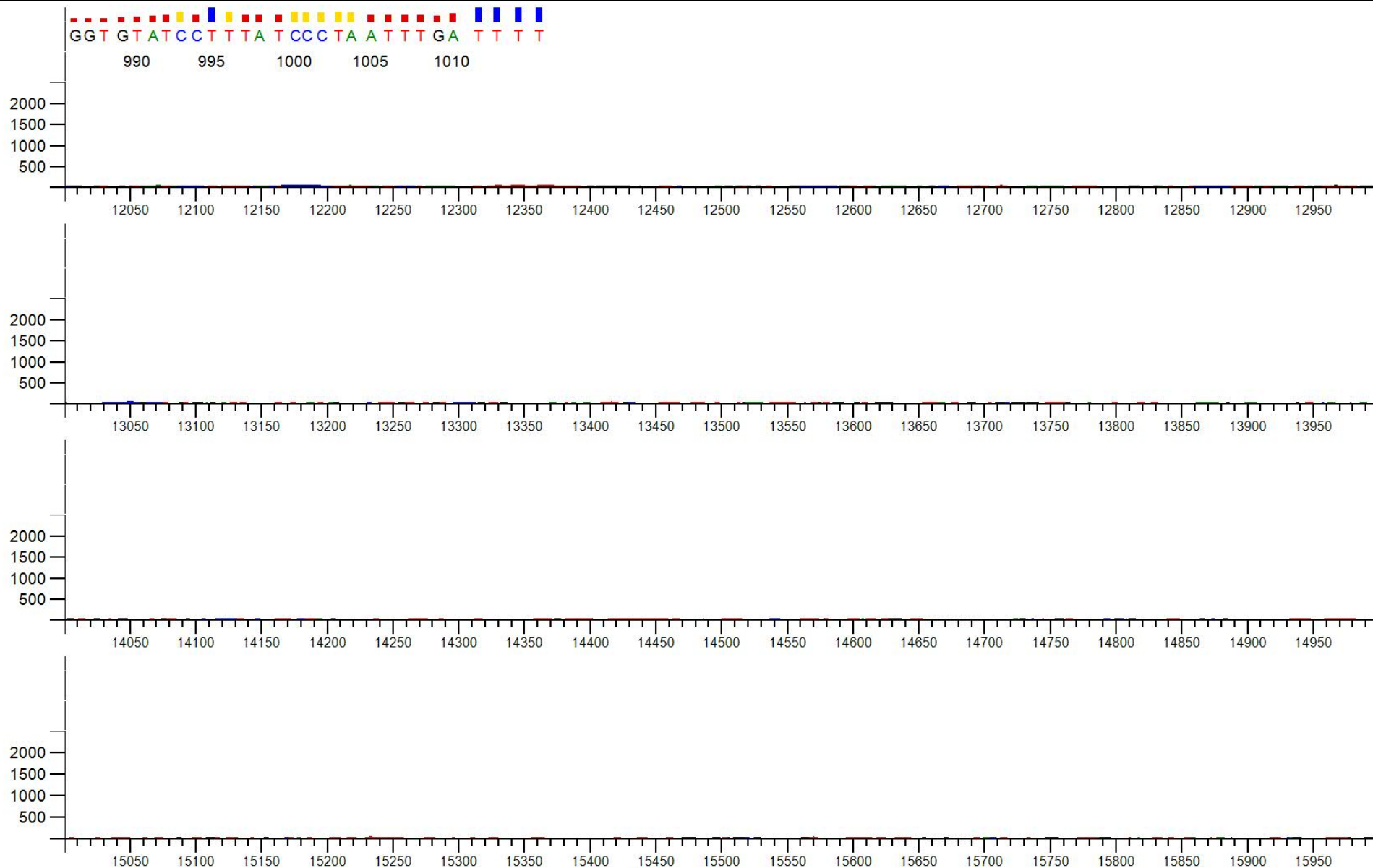

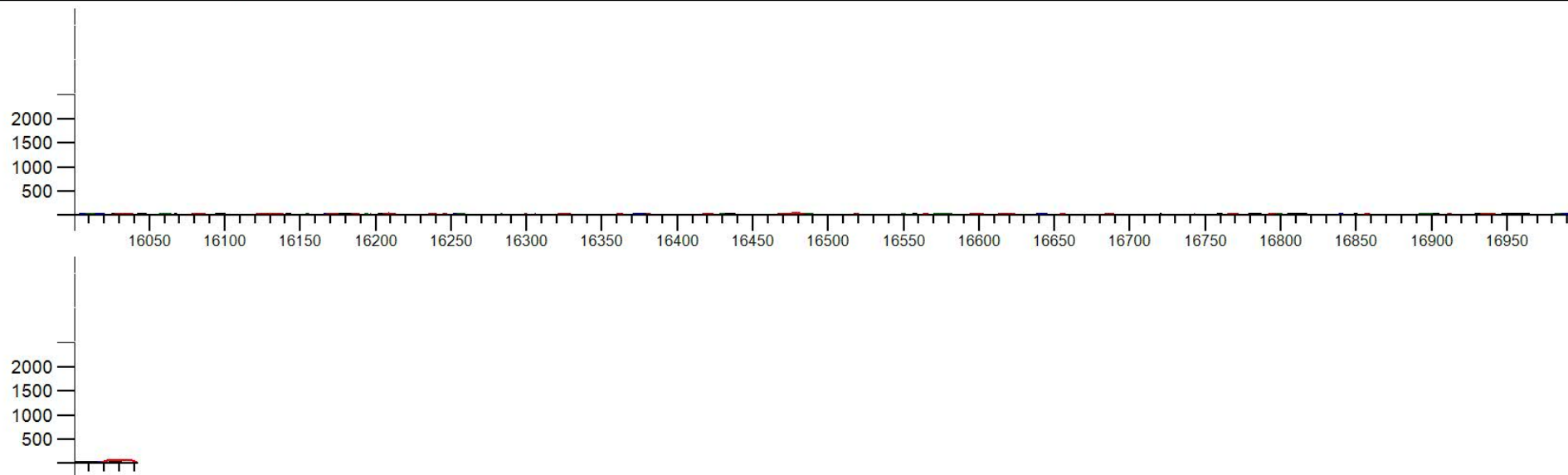

Supplement: Figure 3—source data 1. [file elife-69916-fig3-data1.zip › Figure 3A_Source data2_Bisulphite sequencing data_plasmid/SD-PDT1-BSF-3.18_T7FOR-H11.pdf]

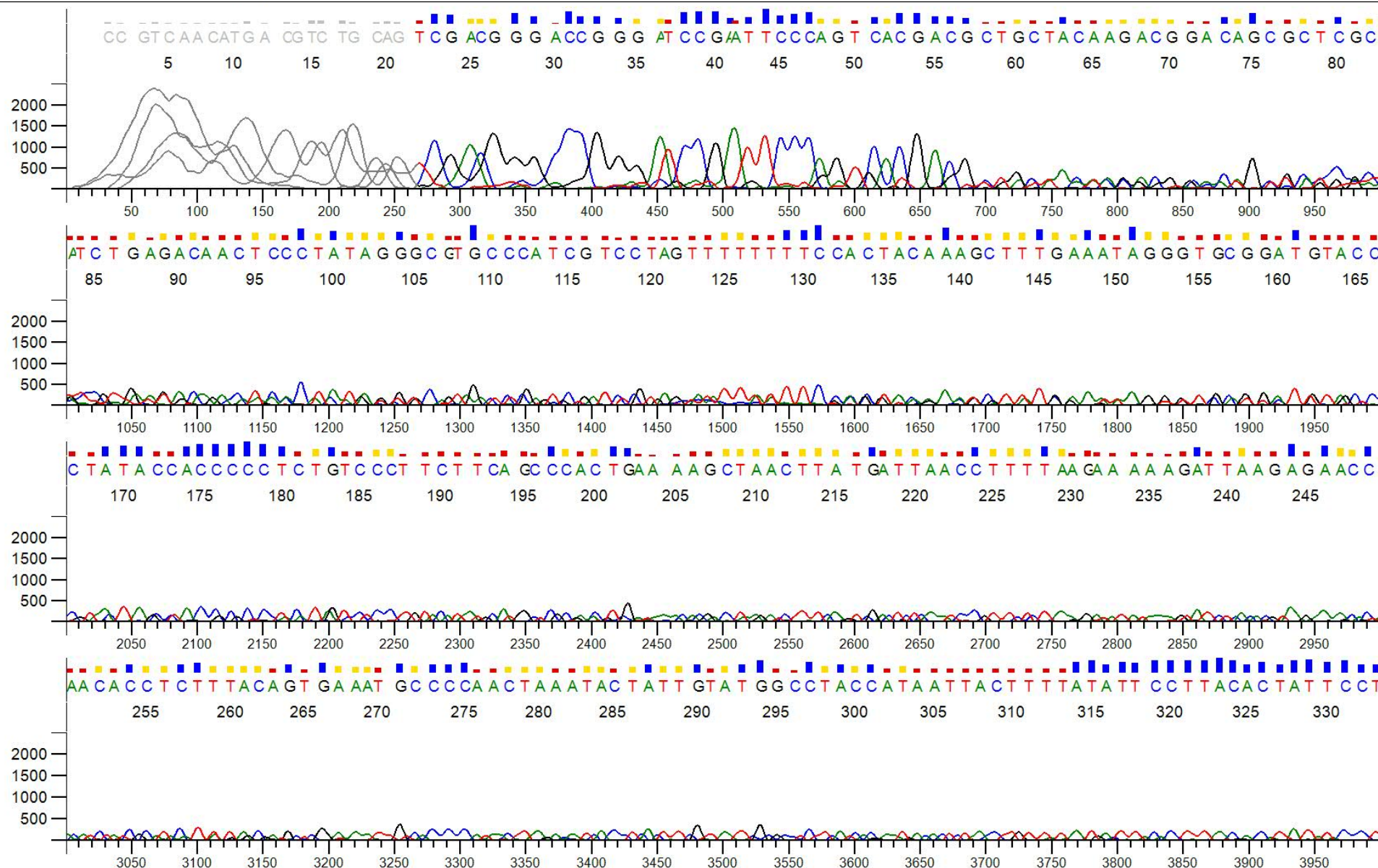

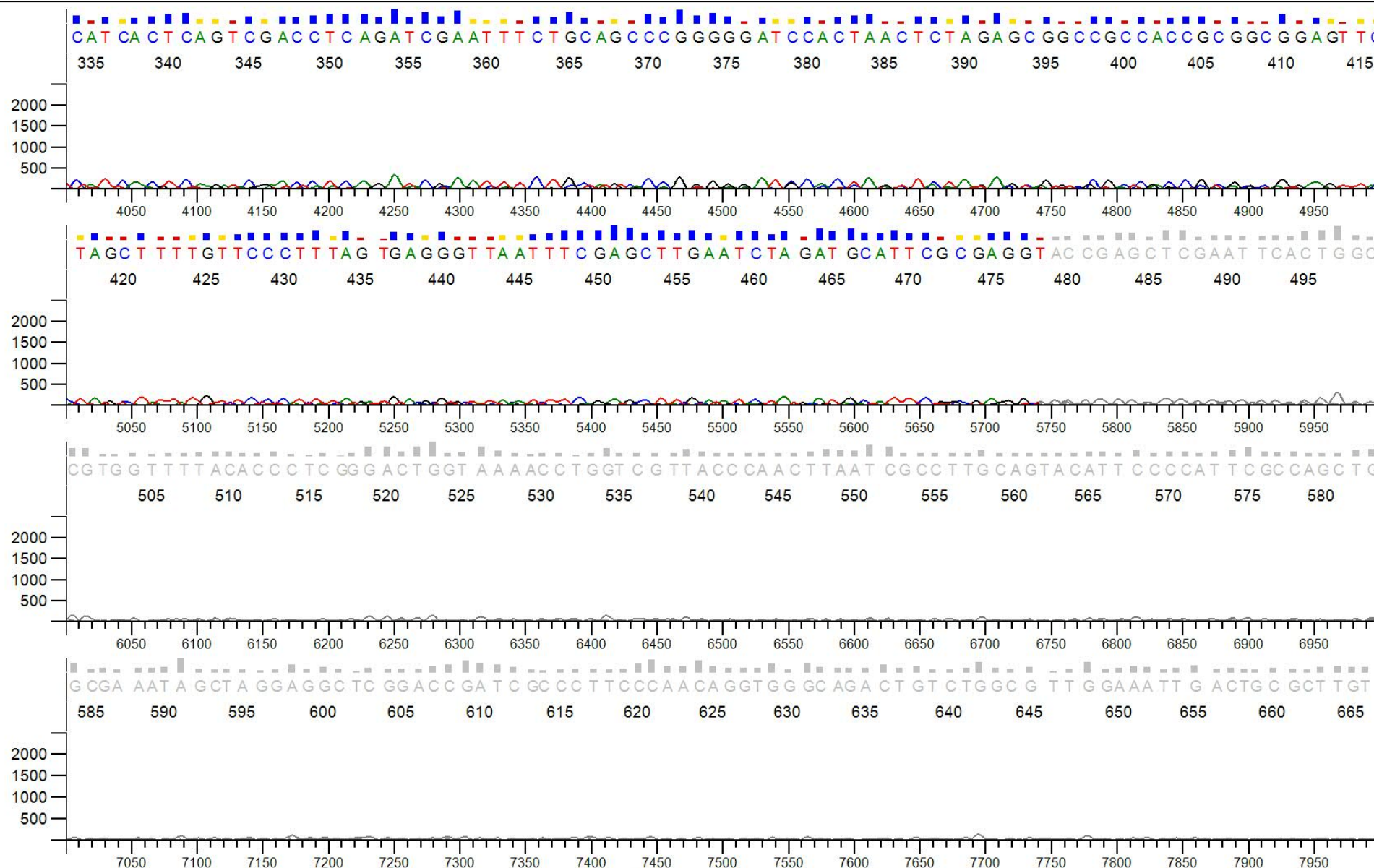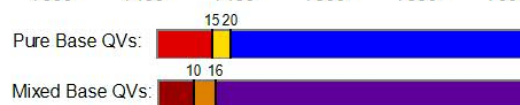

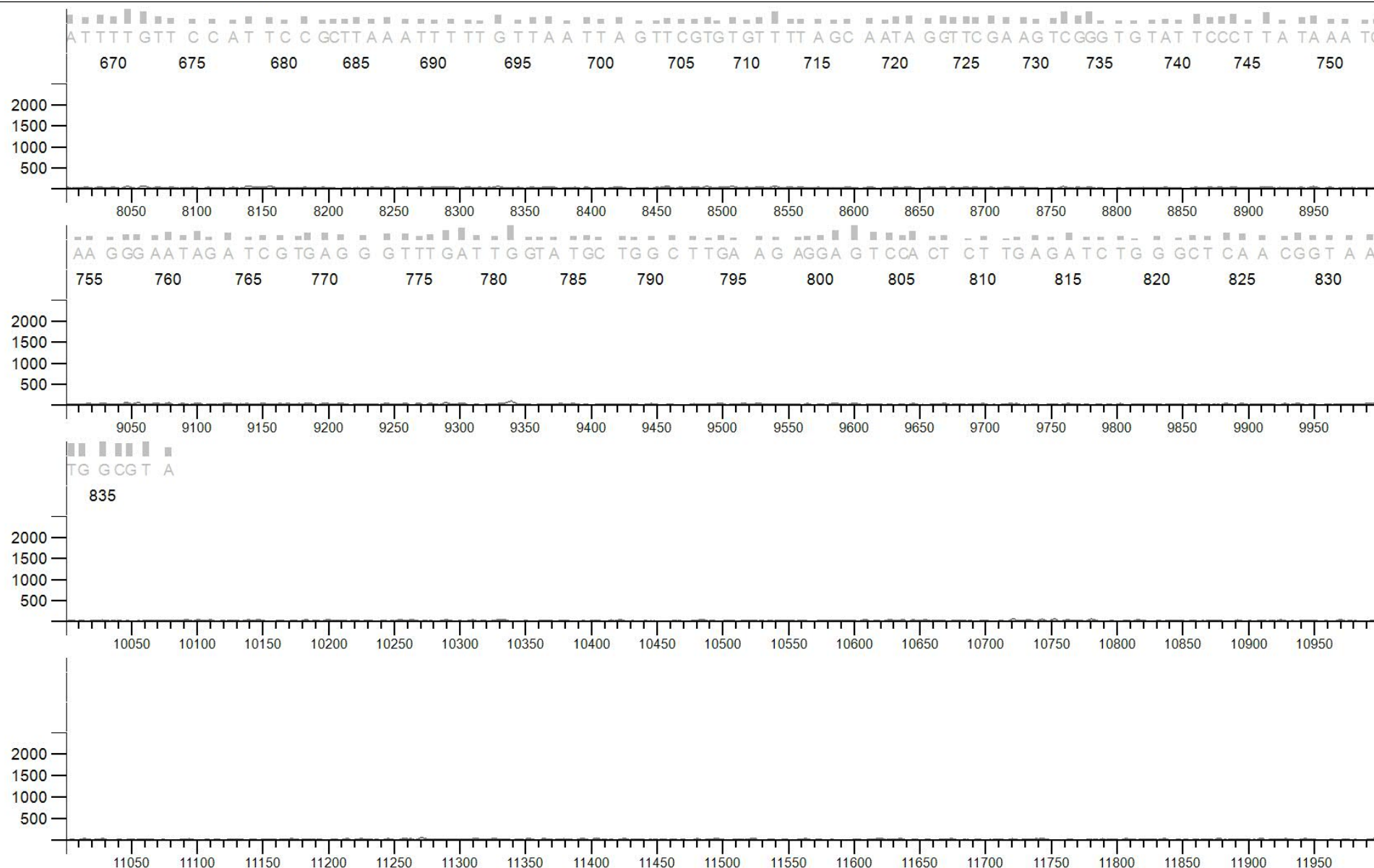

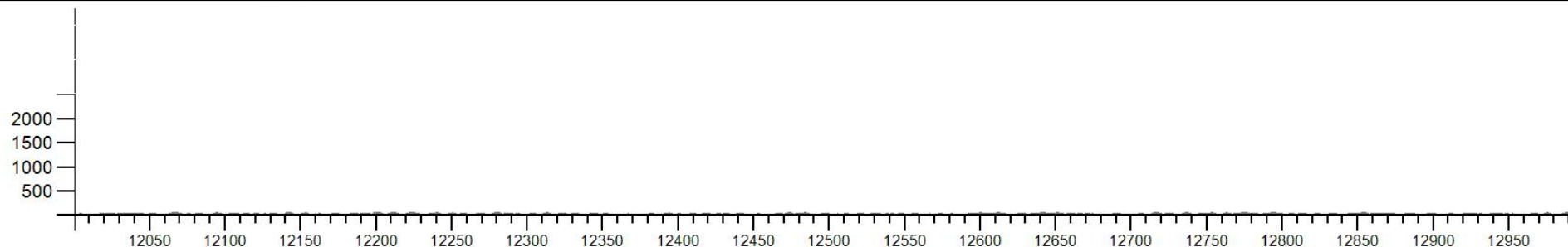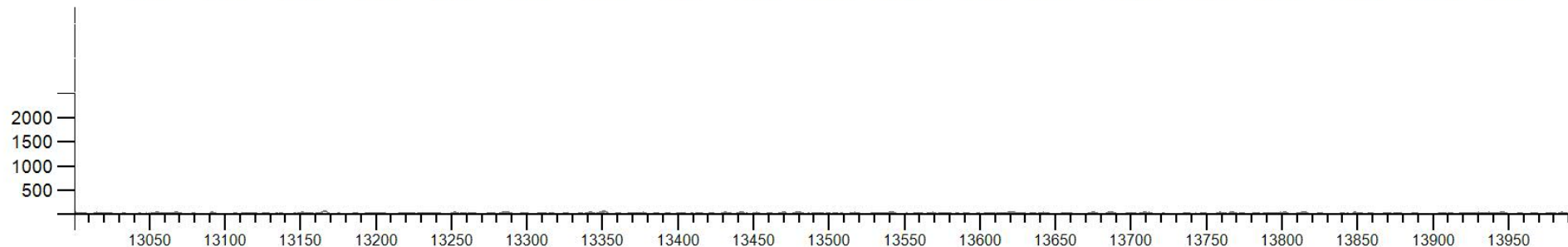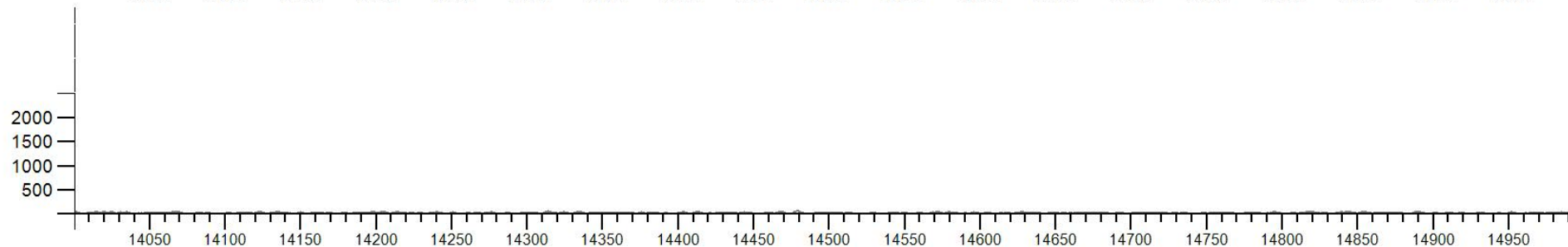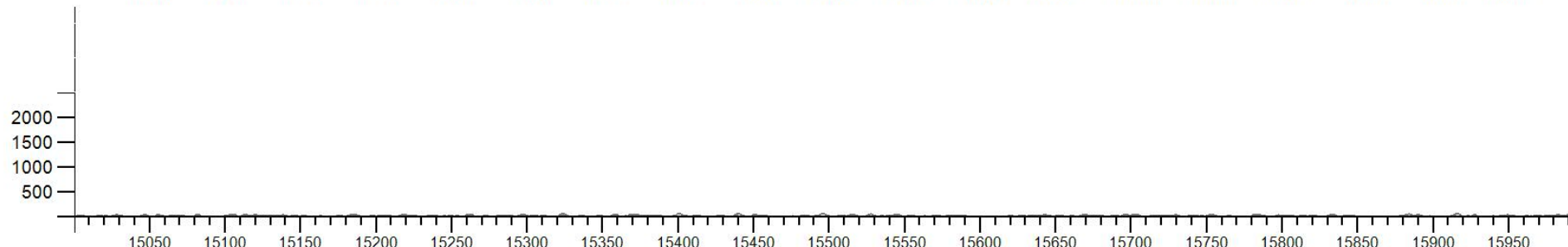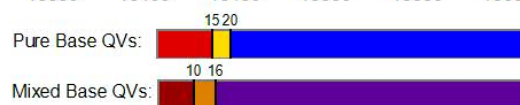

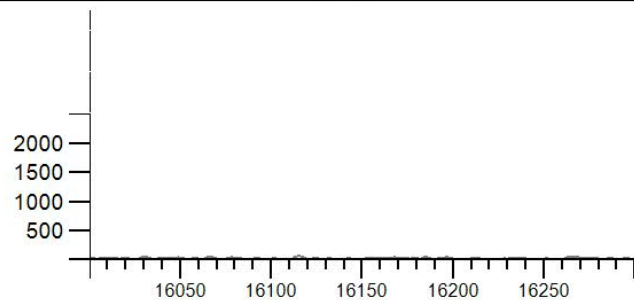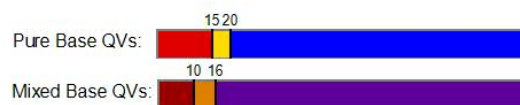

Supplement: Figure 3—source data 1. [file elife-69916-fig3-data1.zip › Figure 3A_Source data2_Bisulphite sequencing data_plasmid/SD-PDT1-BSF-3.6_T7FOR-B11.pdf]

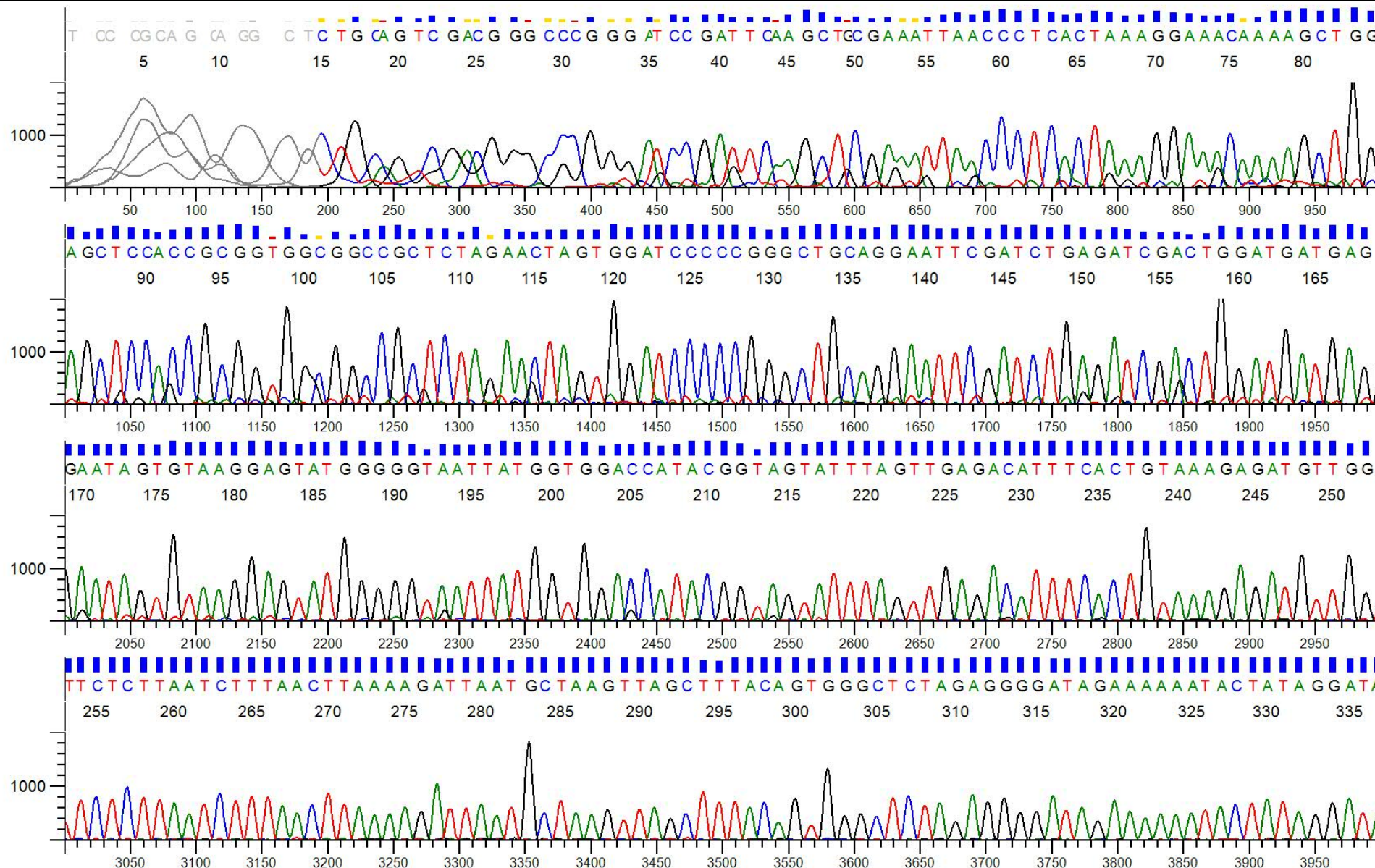

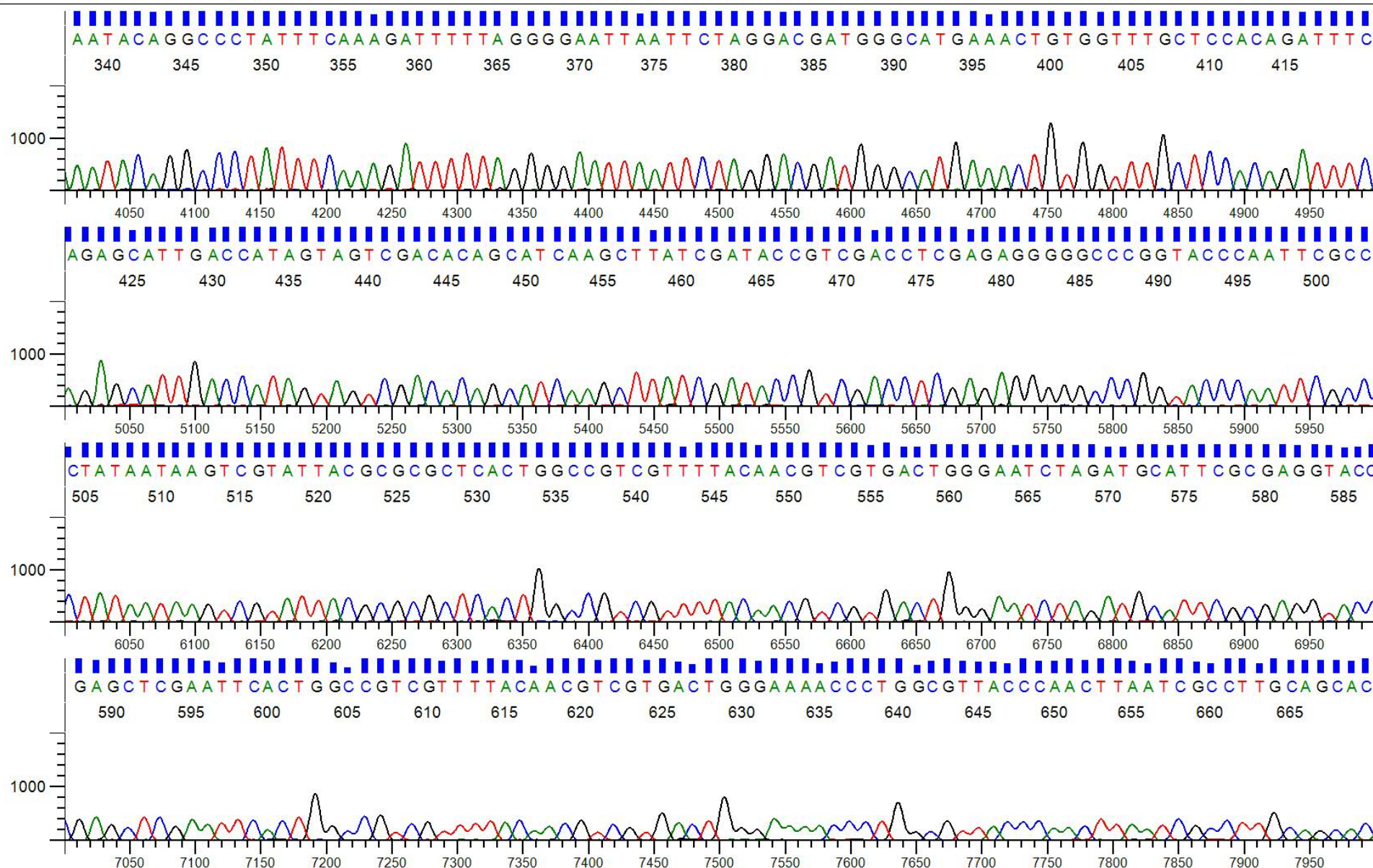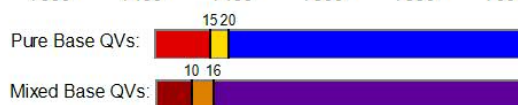

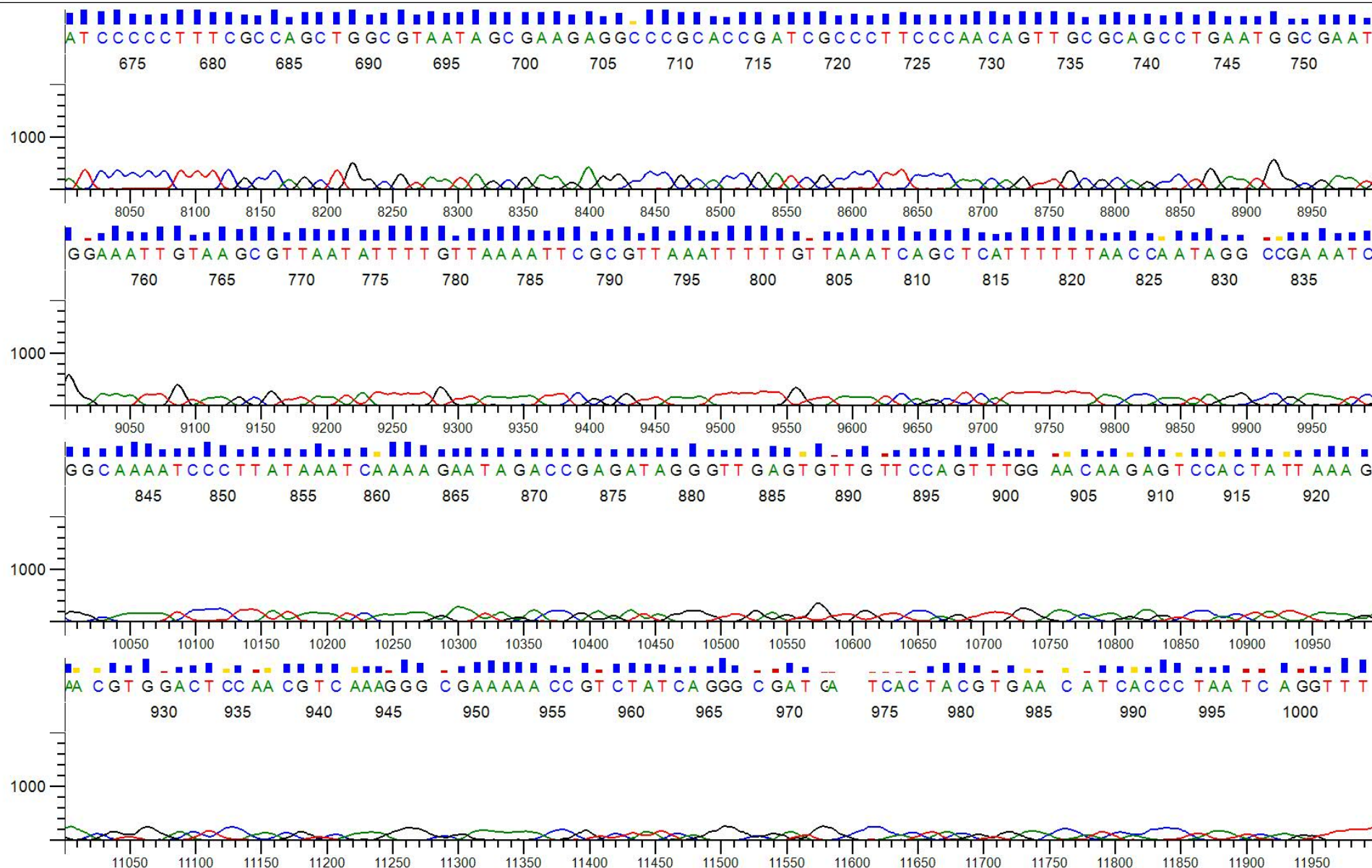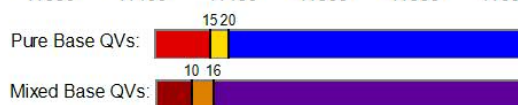

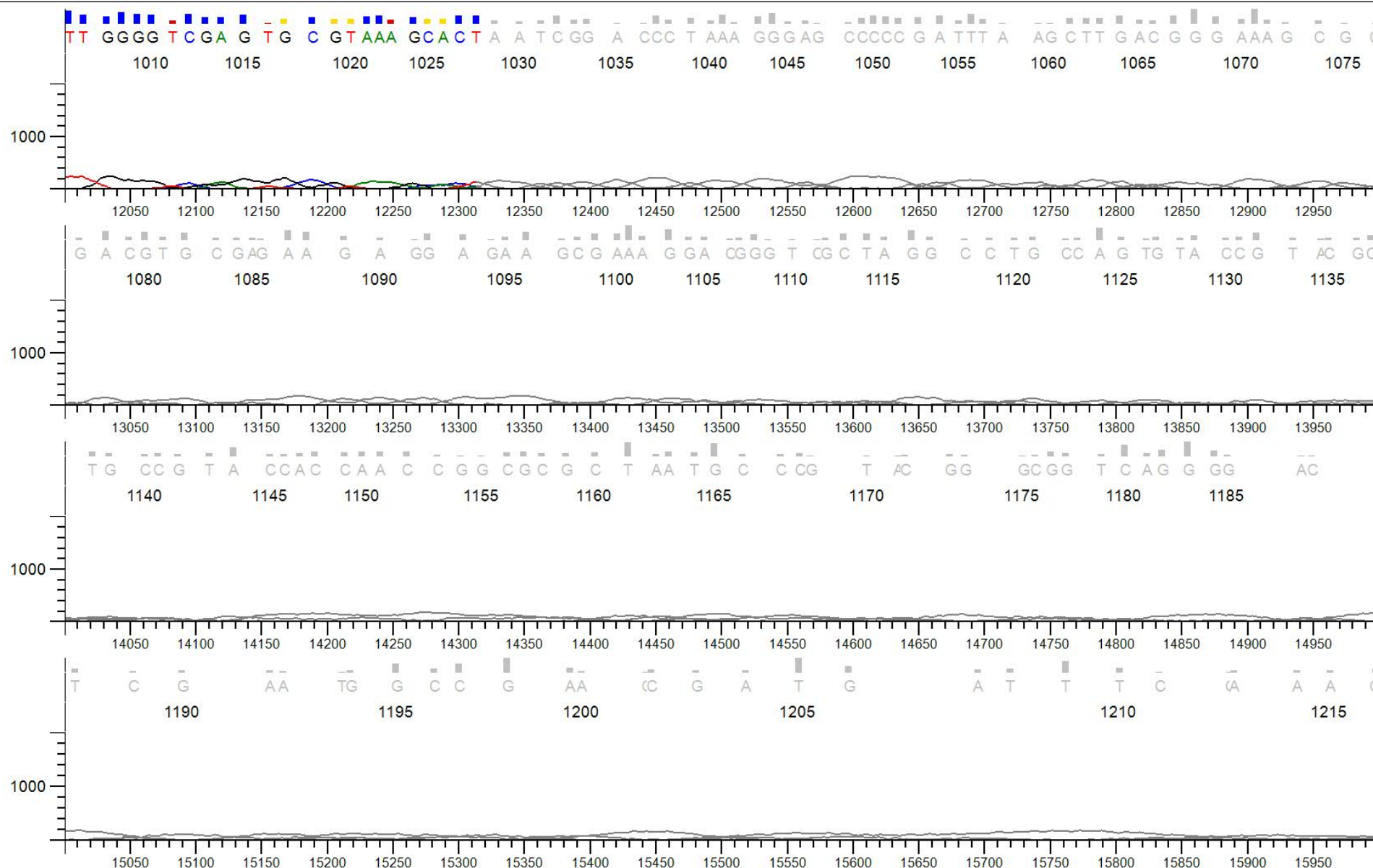

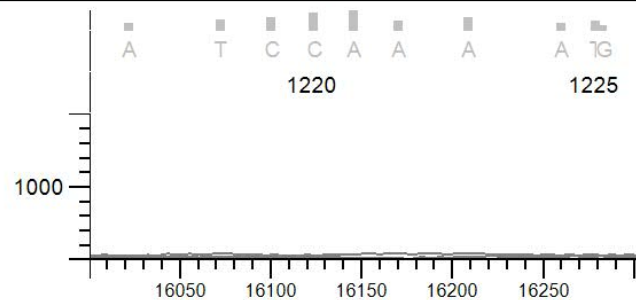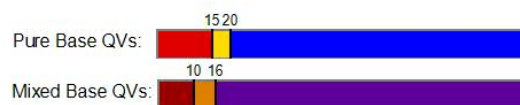

Supplement: Figure 3—source data 1. [file elife-69916-fig3-data1.zip › Figure 3A_Source data2_Bisulphite sequencing data_plasmid/SS4_PDI1_BIS_16_T7FOR-G01.pdf]

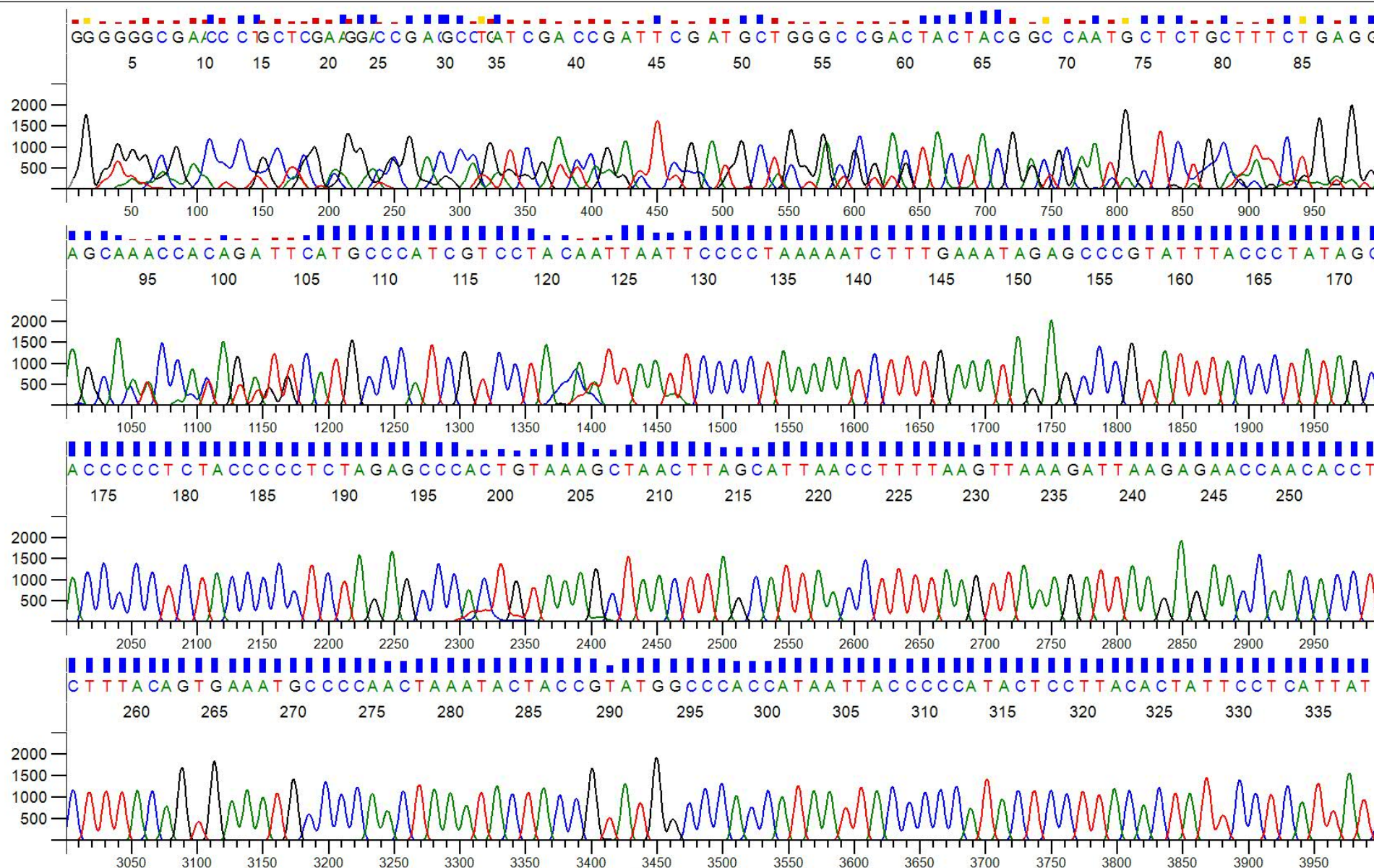

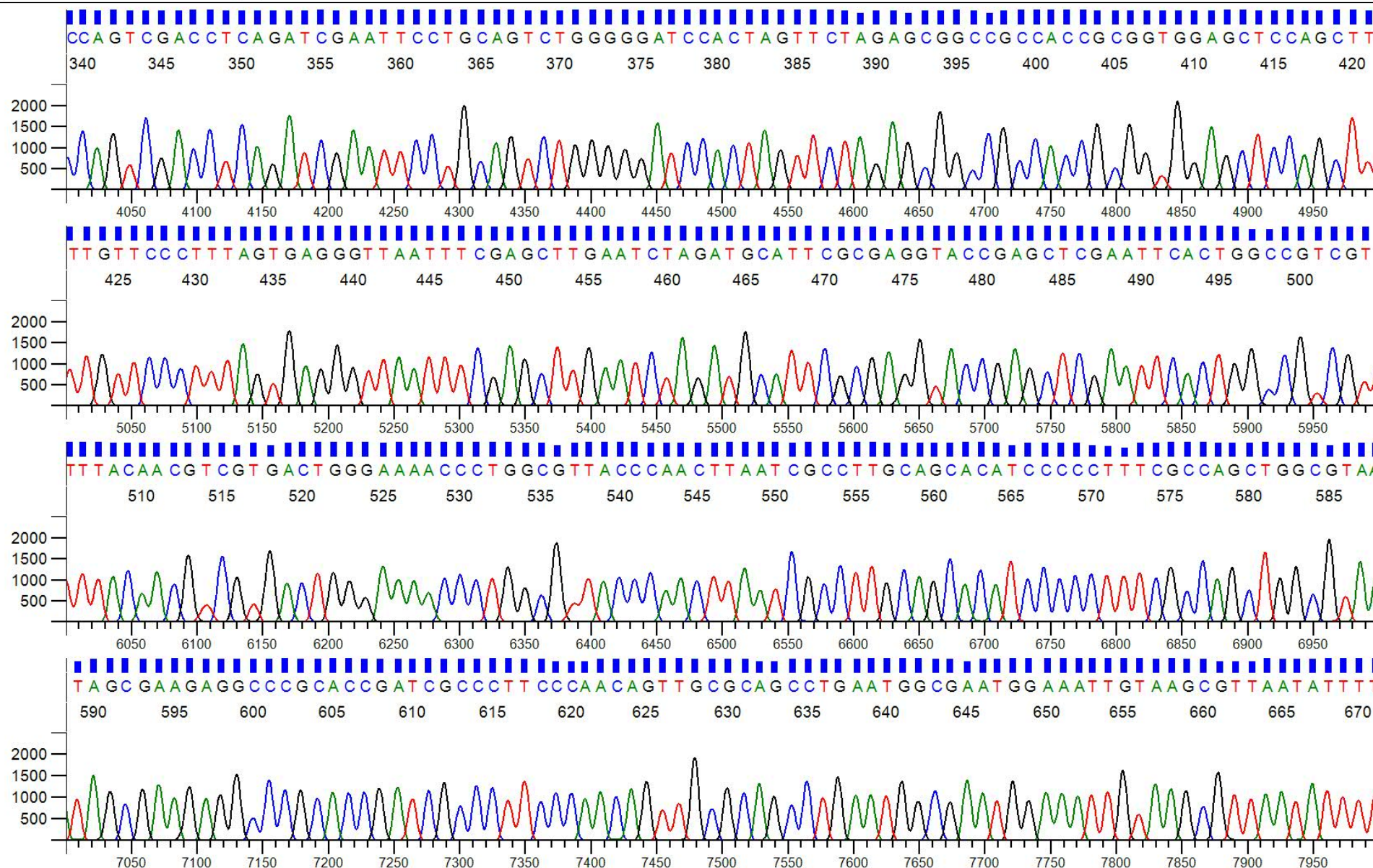

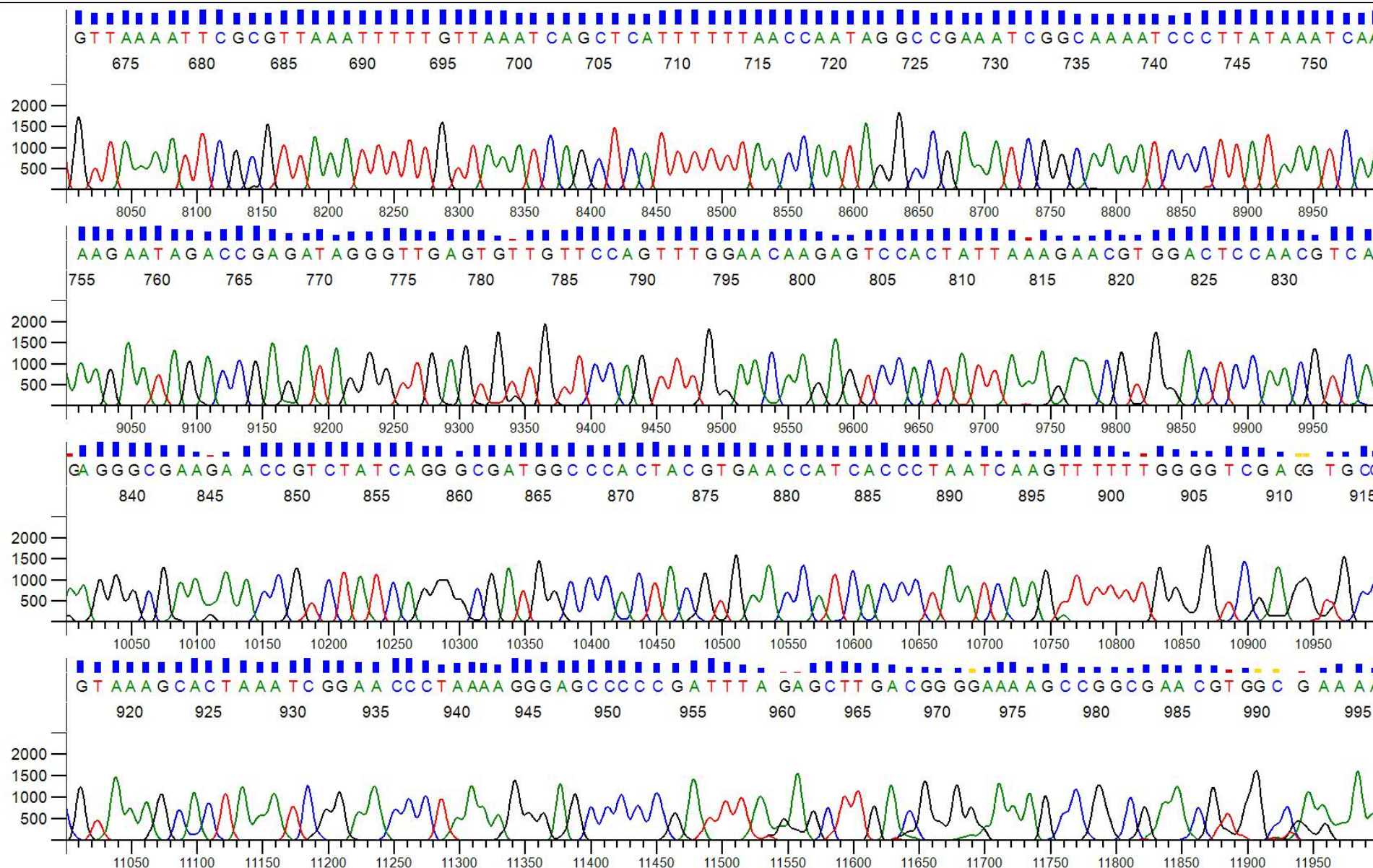

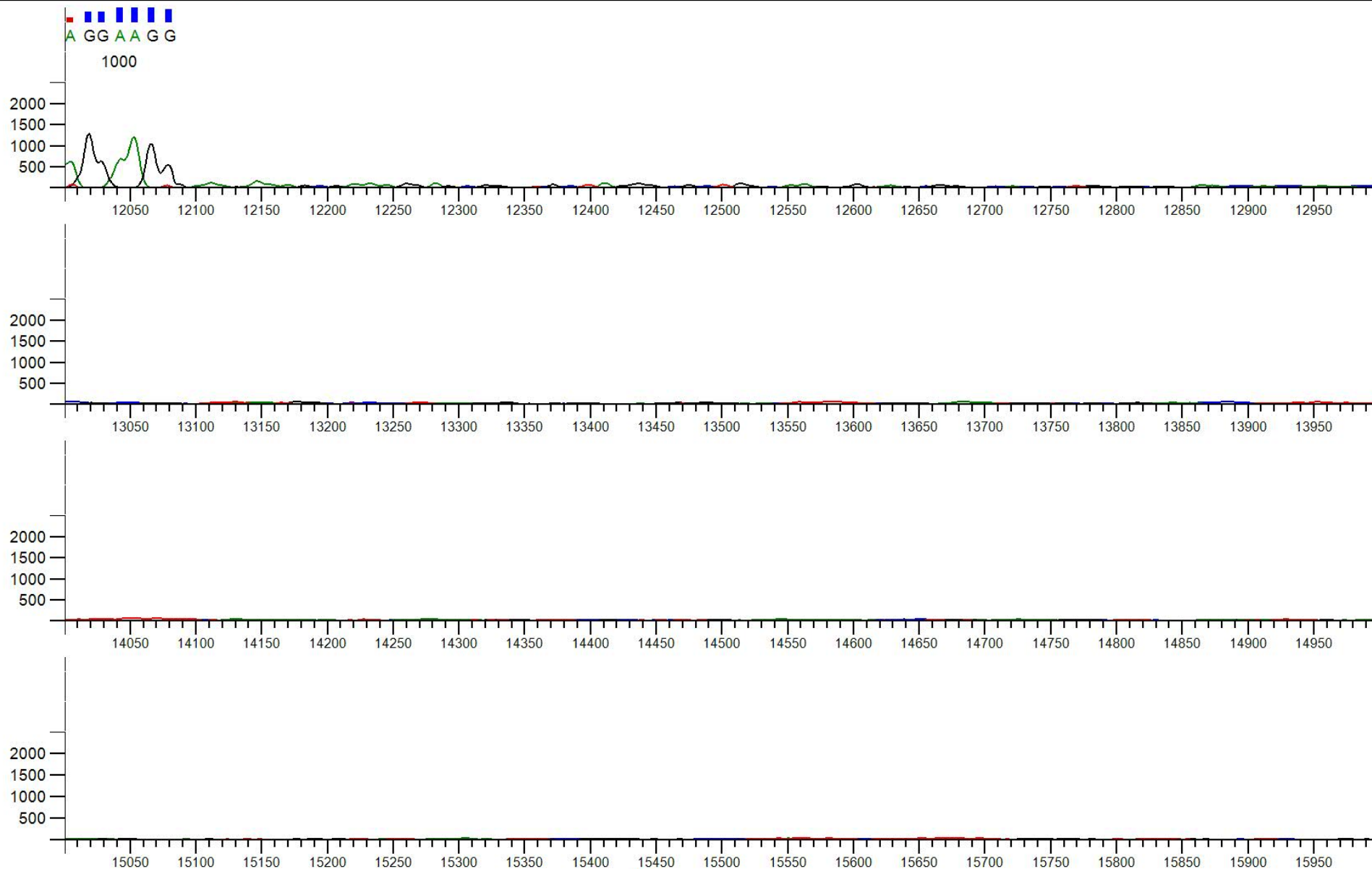

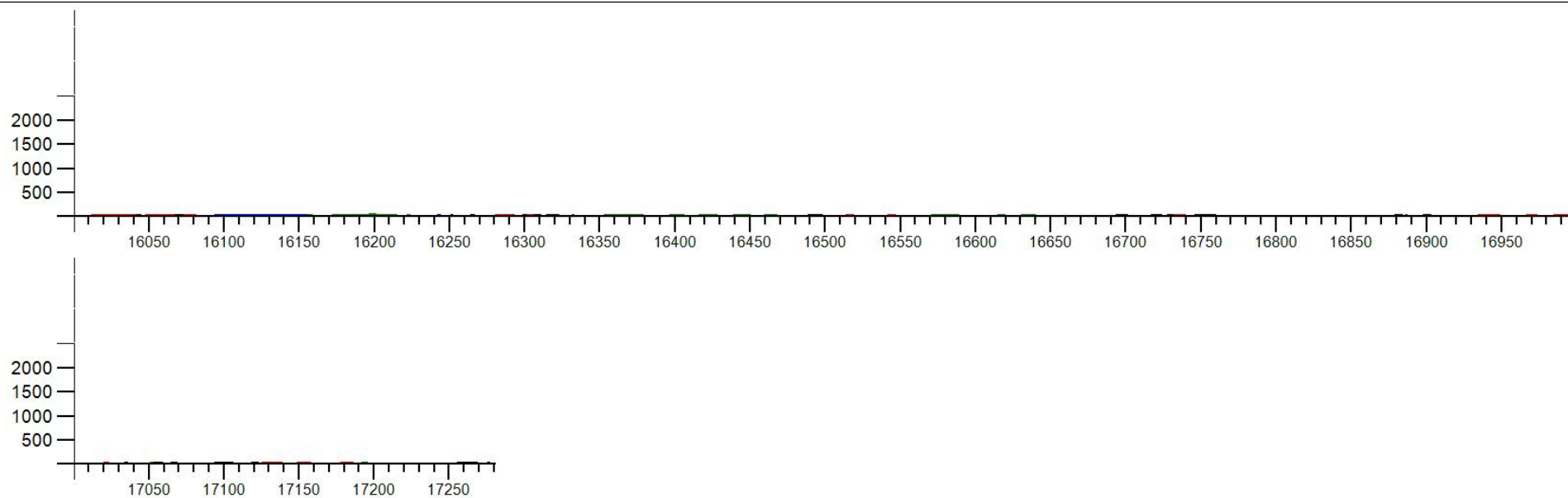

Supplement: Figure 3—source data 1. [file elife-69916-fig3-data1.zip › Figure 3A_Source data2_Bisulphite sequencing data_plasmid/SD-PDT1-BSF-3.4_T7FOR-H10.pdf]

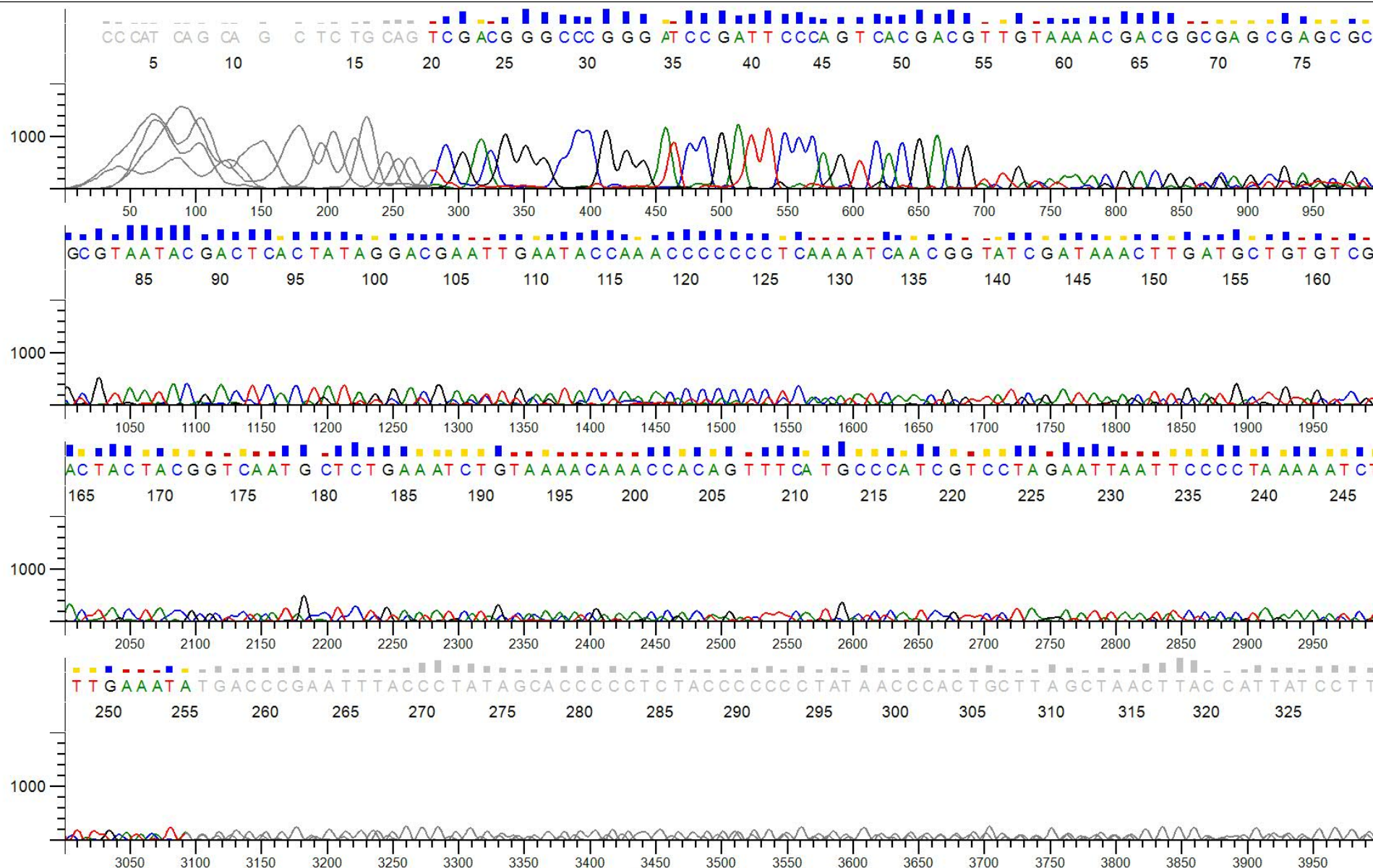

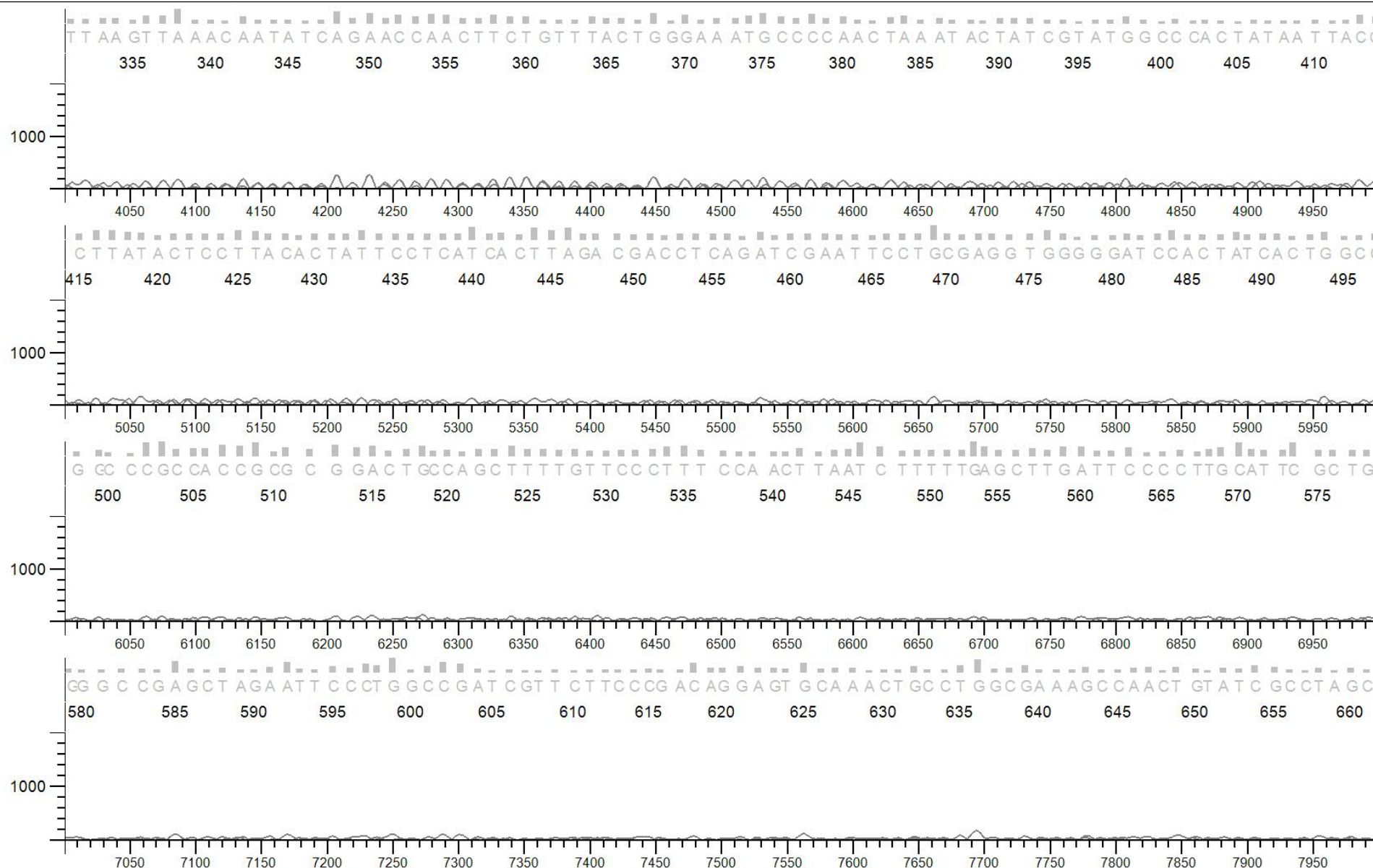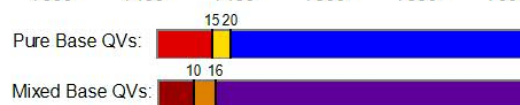

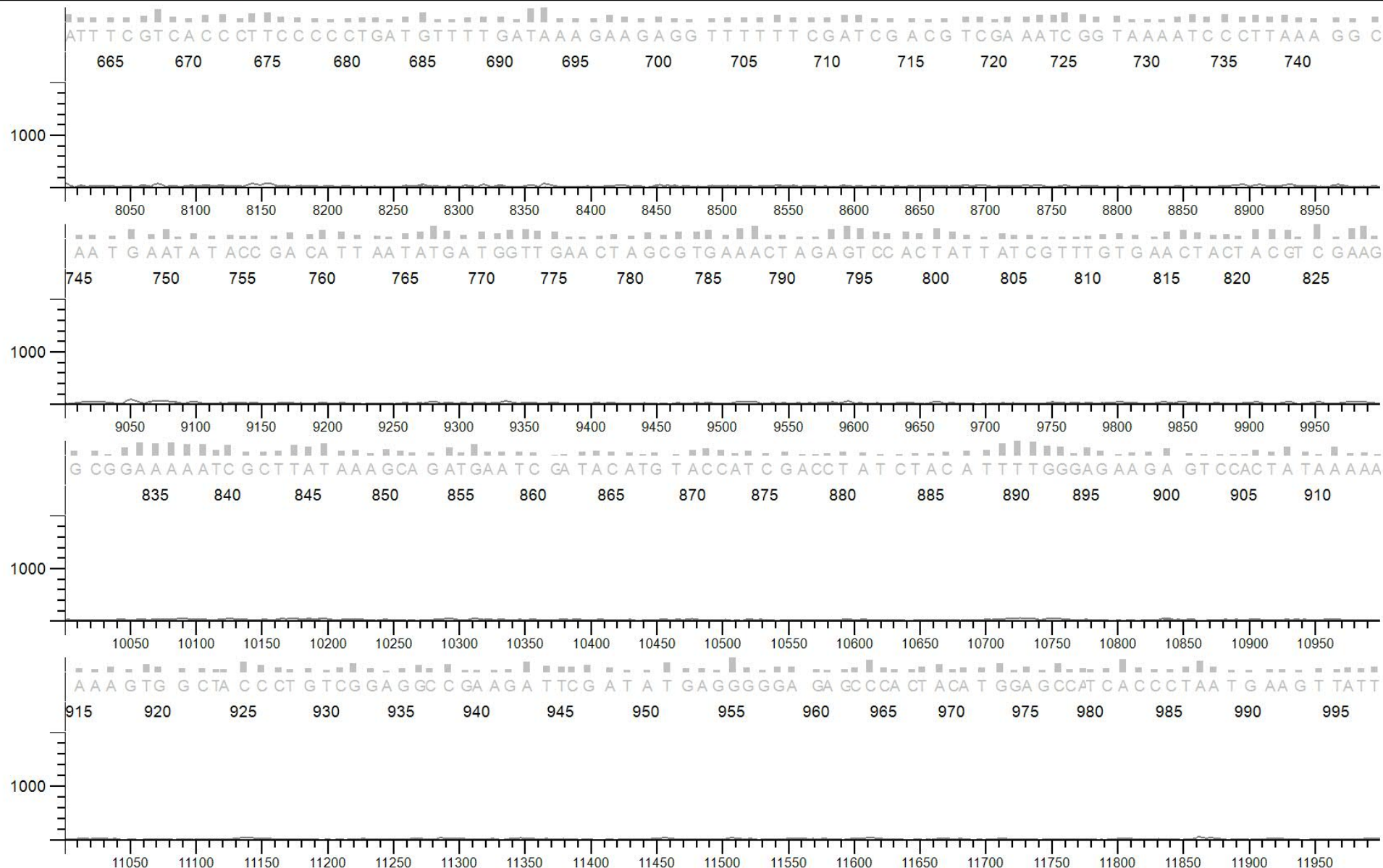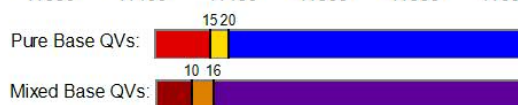

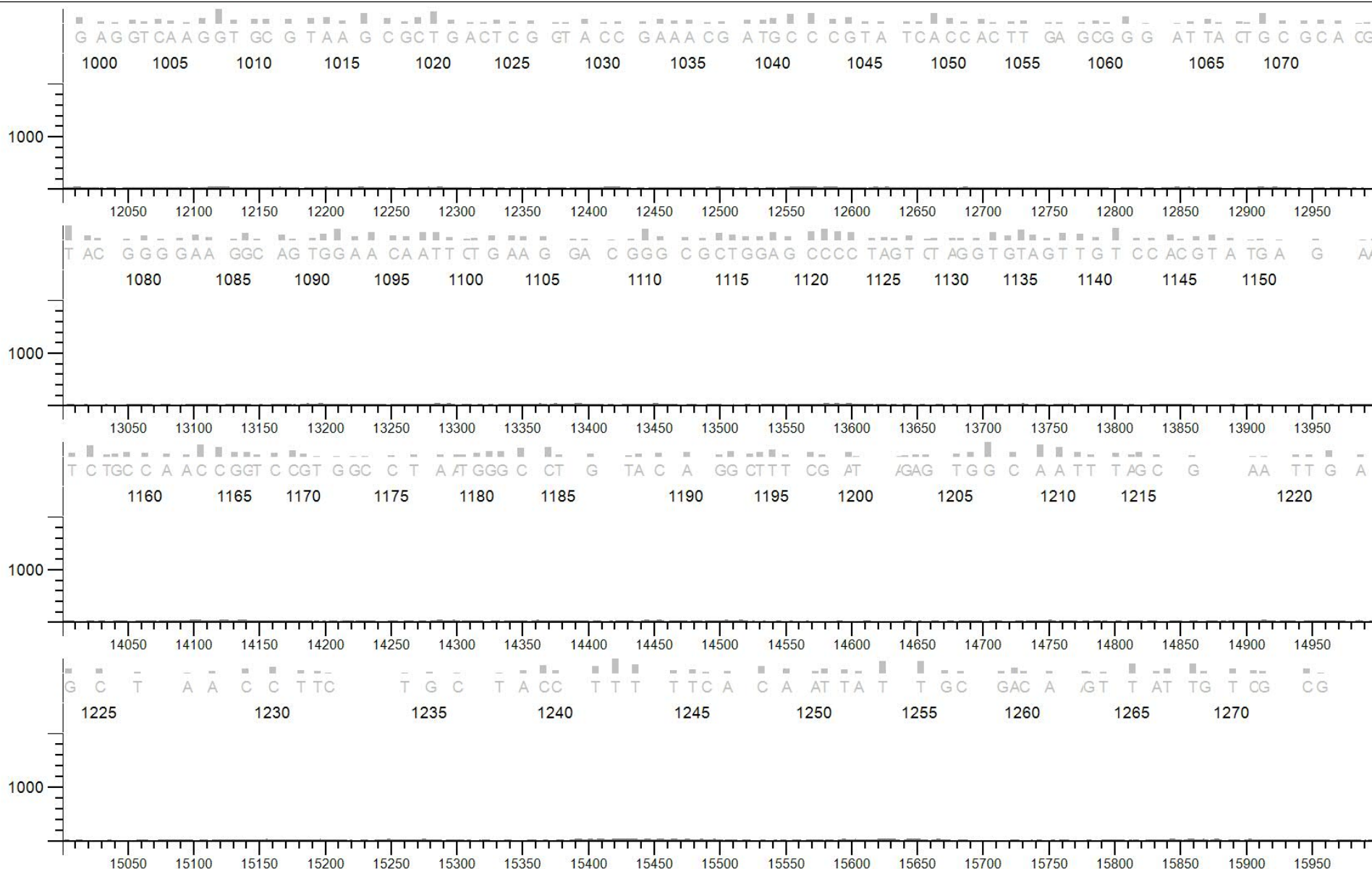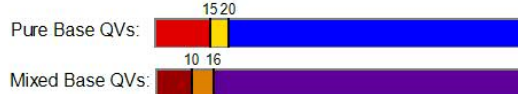

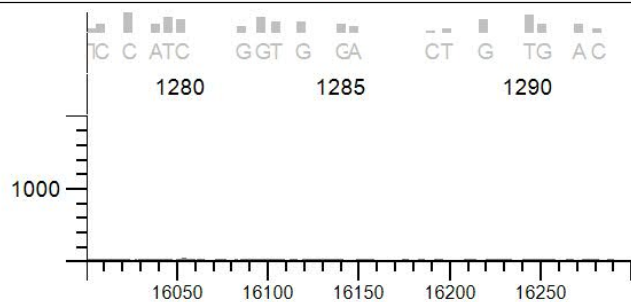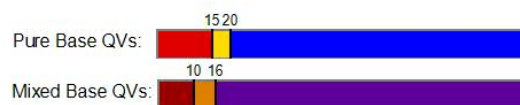

Supplement: Figure 3—source data 1. [file elife-69916-fig3-data1.zip › Figure 3A_Source data2_Bisulphite sequencing data_plasmid/SS4_PDI1_BIS_2_T7FOR-F01.pdf]

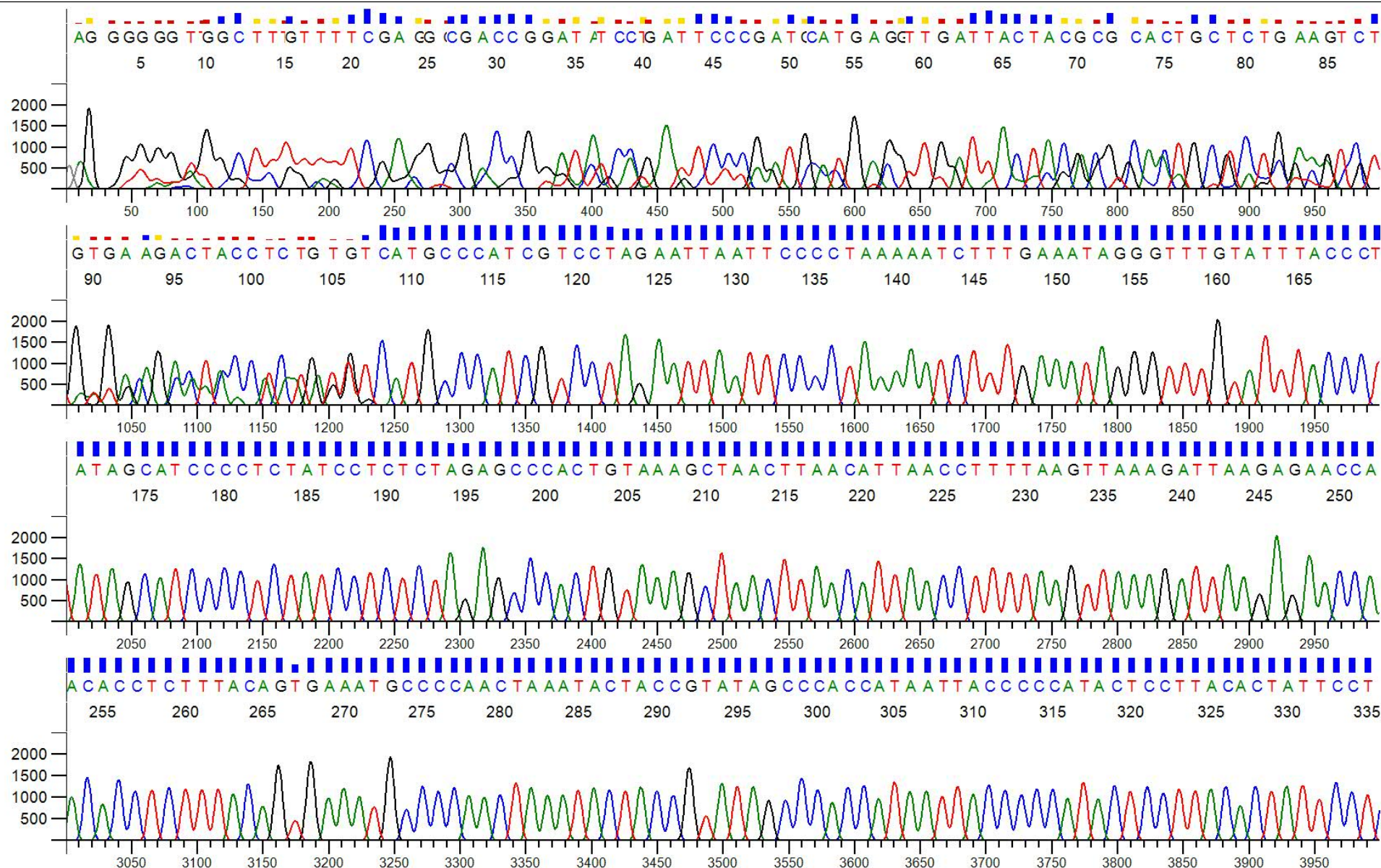

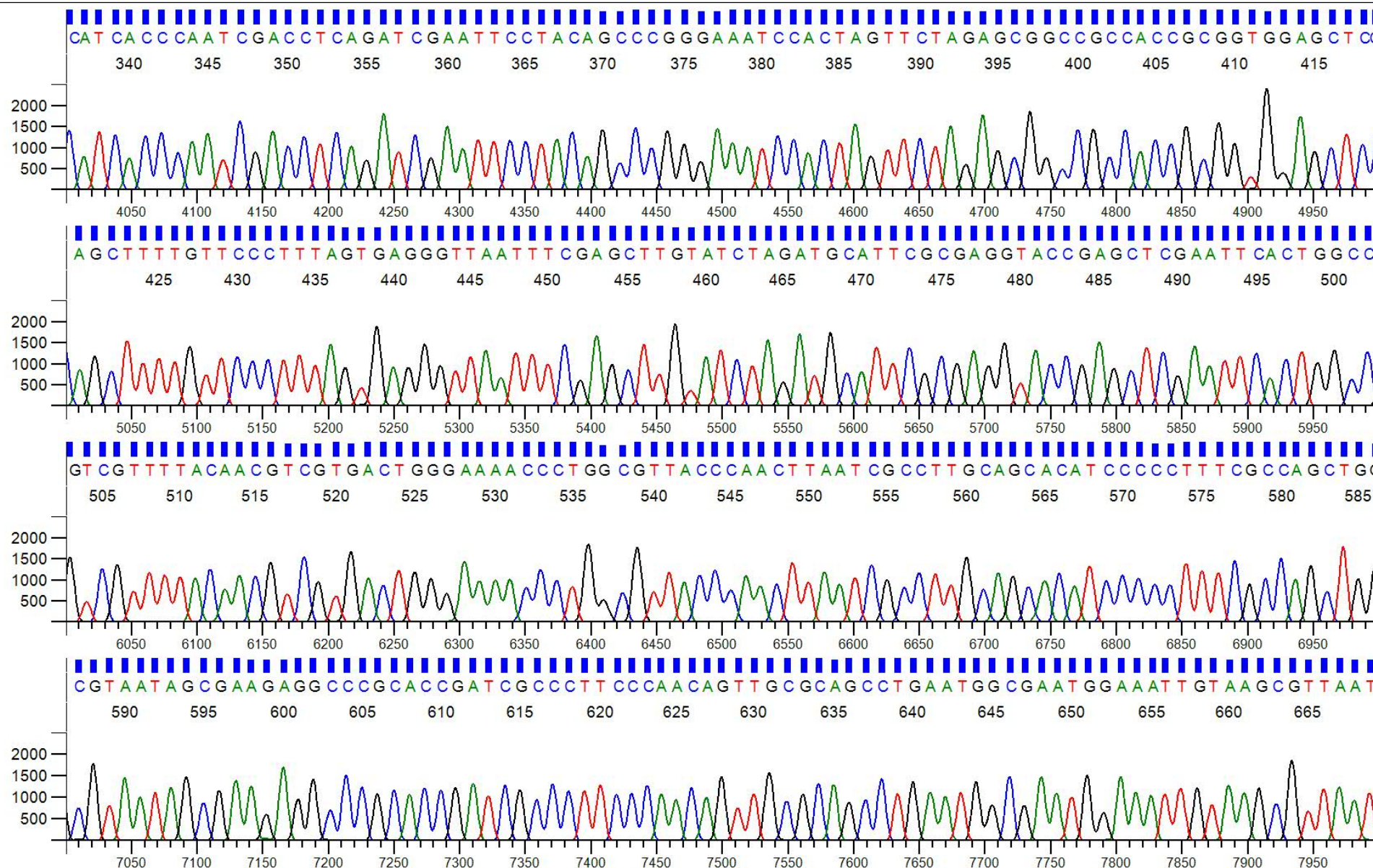

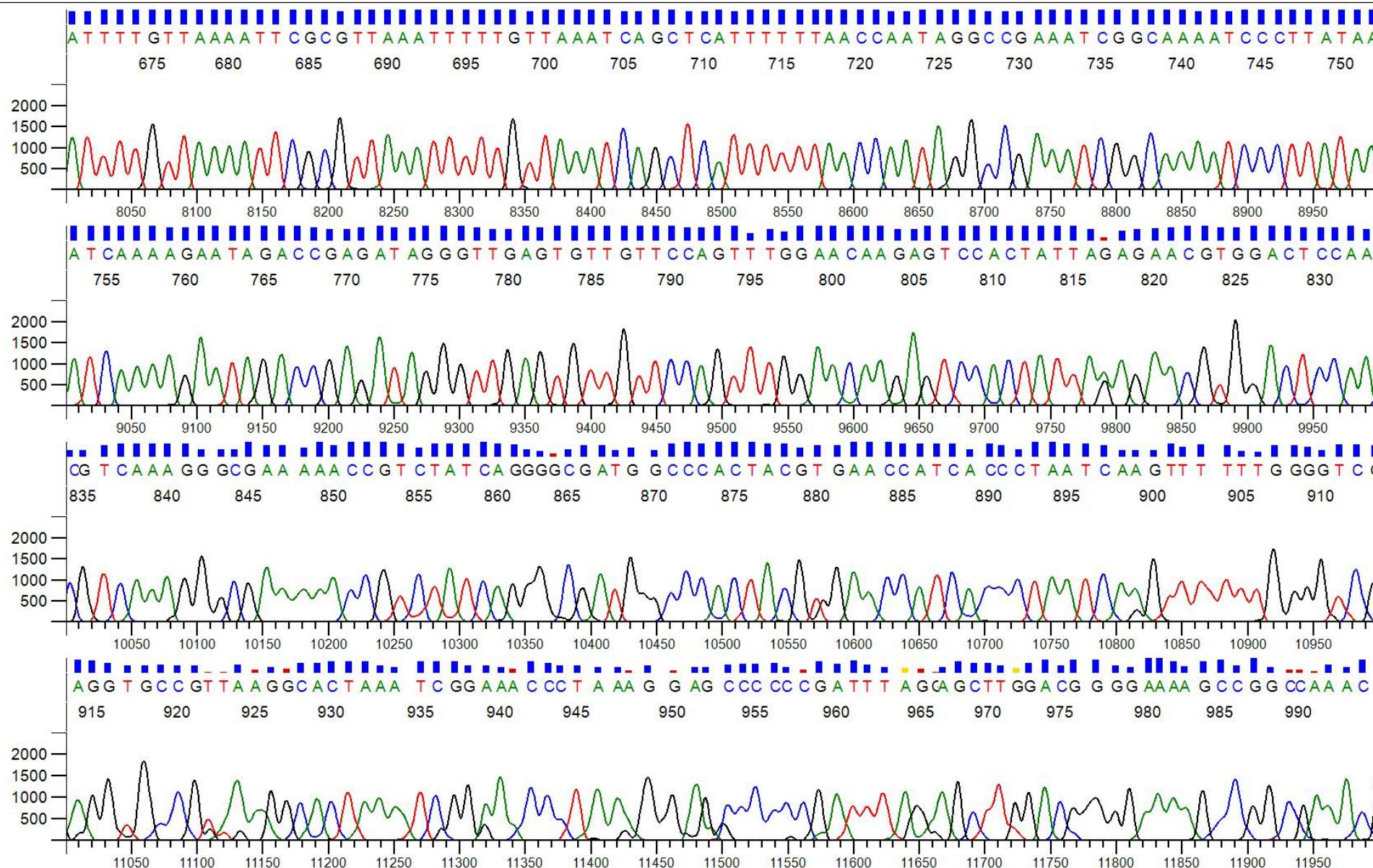

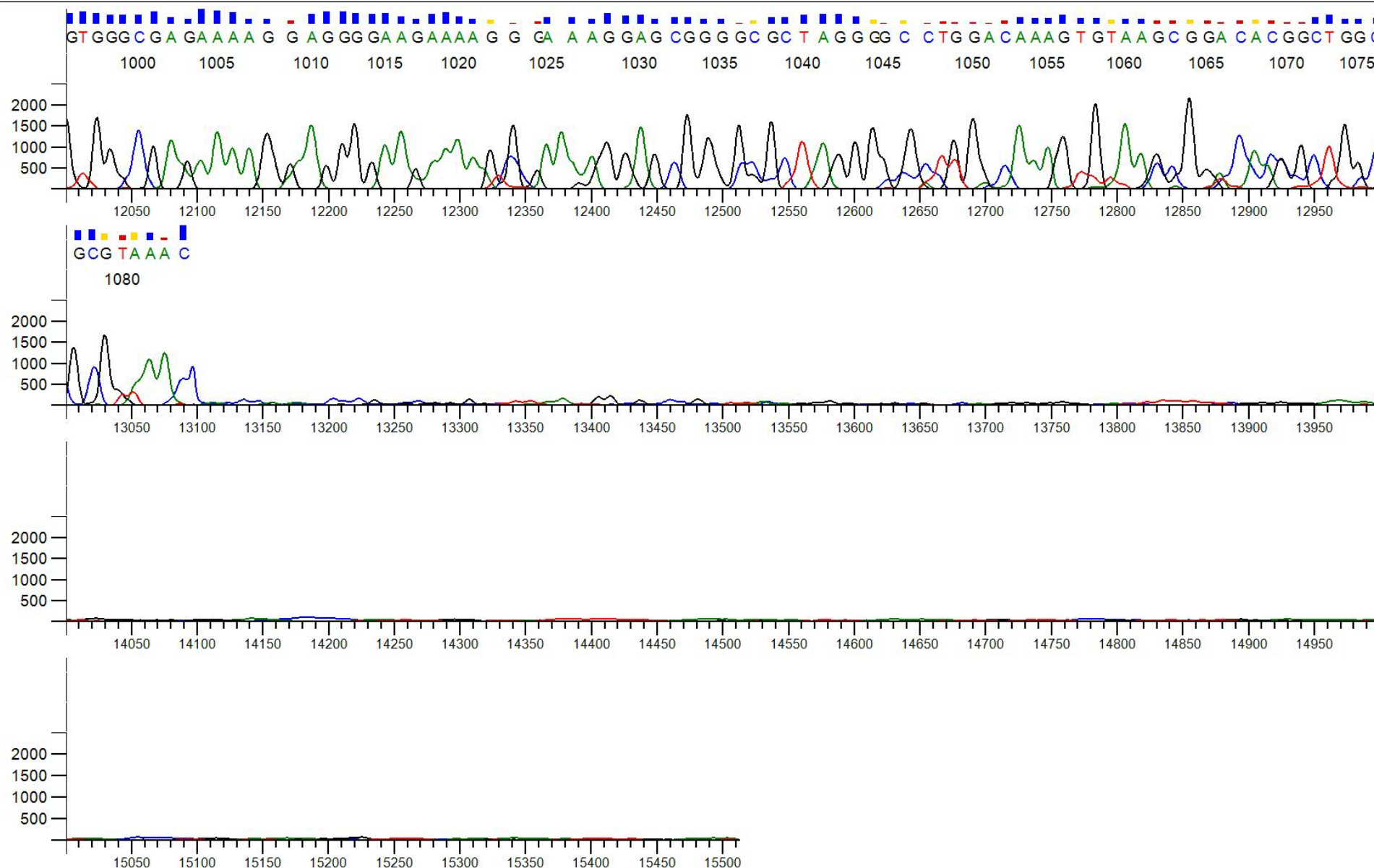

Supplement: Figure 3—source data 1. [file elife-69916-fig3-data1.zip › Figure 3A_Source data2_Bisulphite sequencing data_plasmid/SD-PDT1-BSF-3.19_T7FOR-A12.pdf]

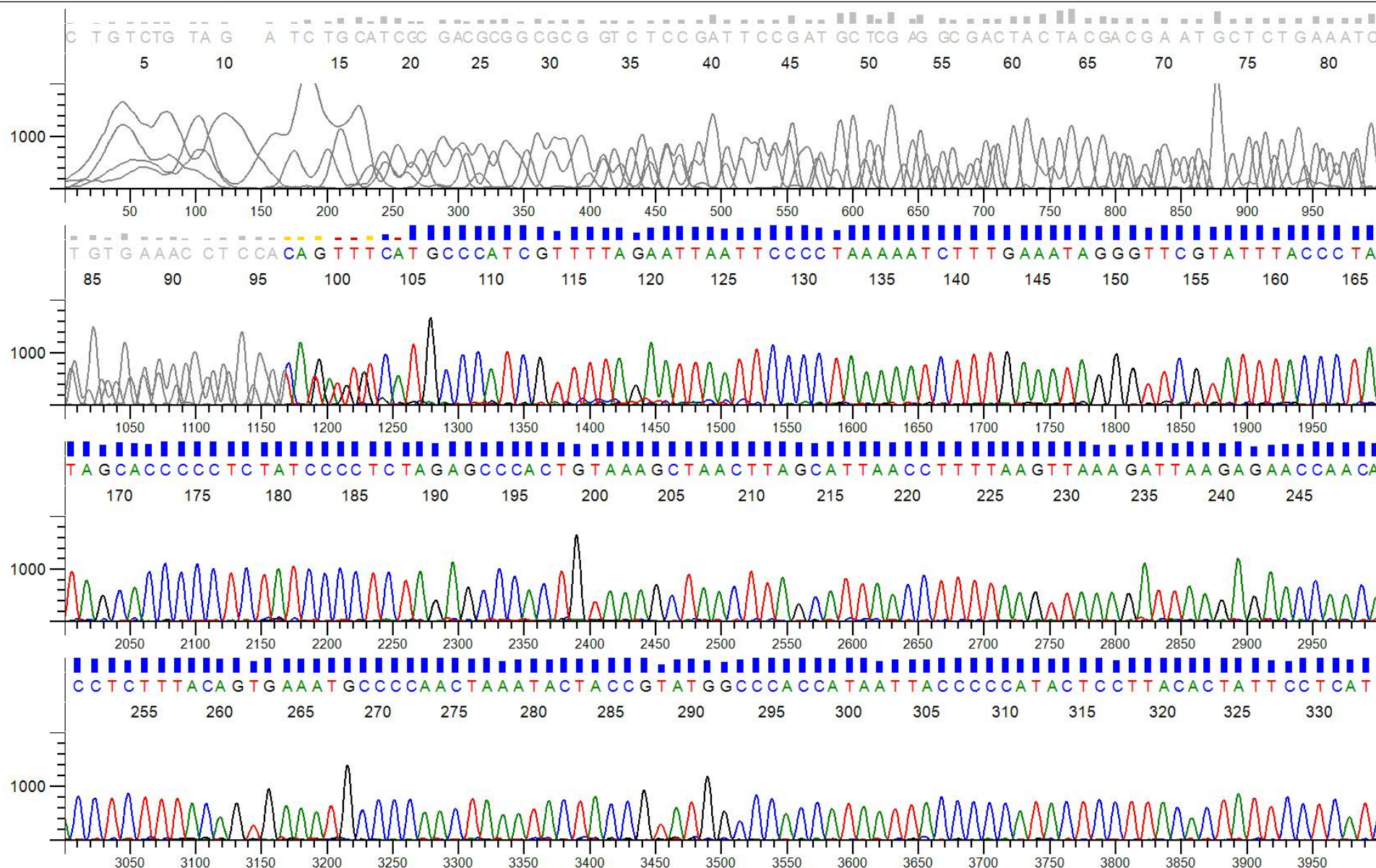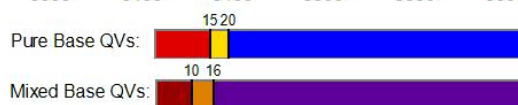

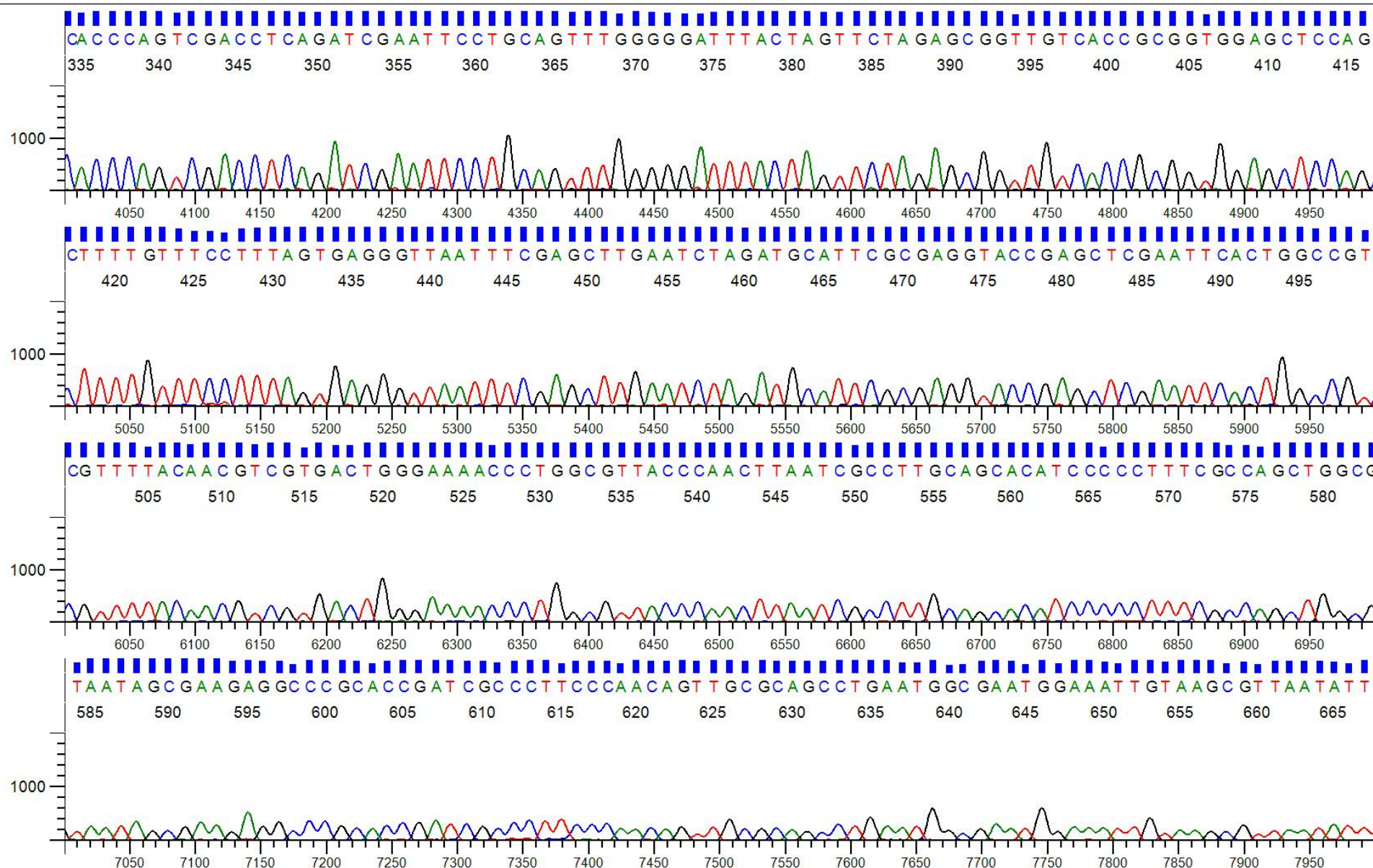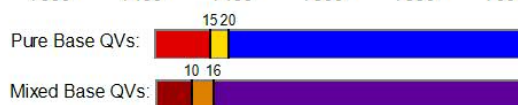

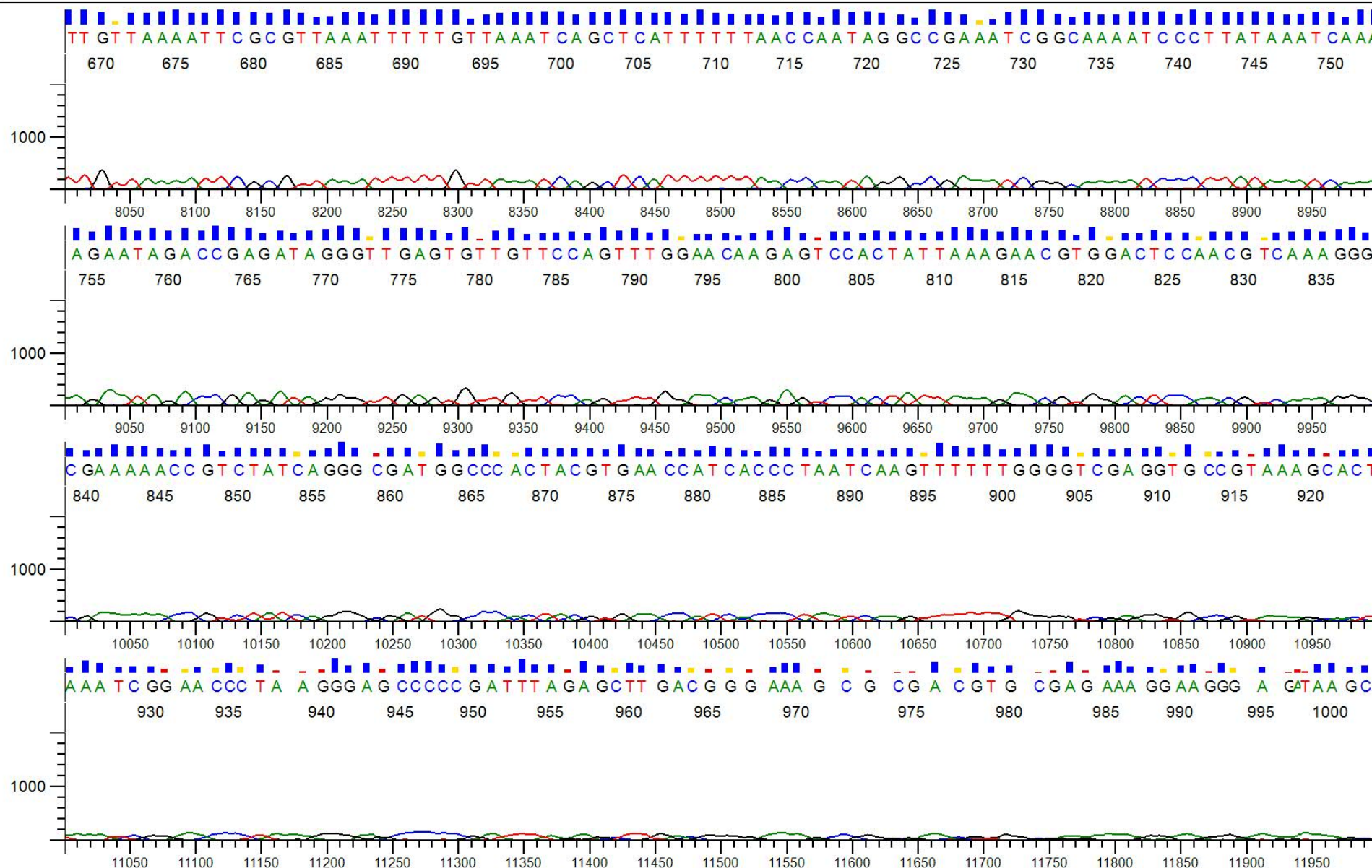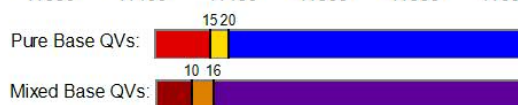

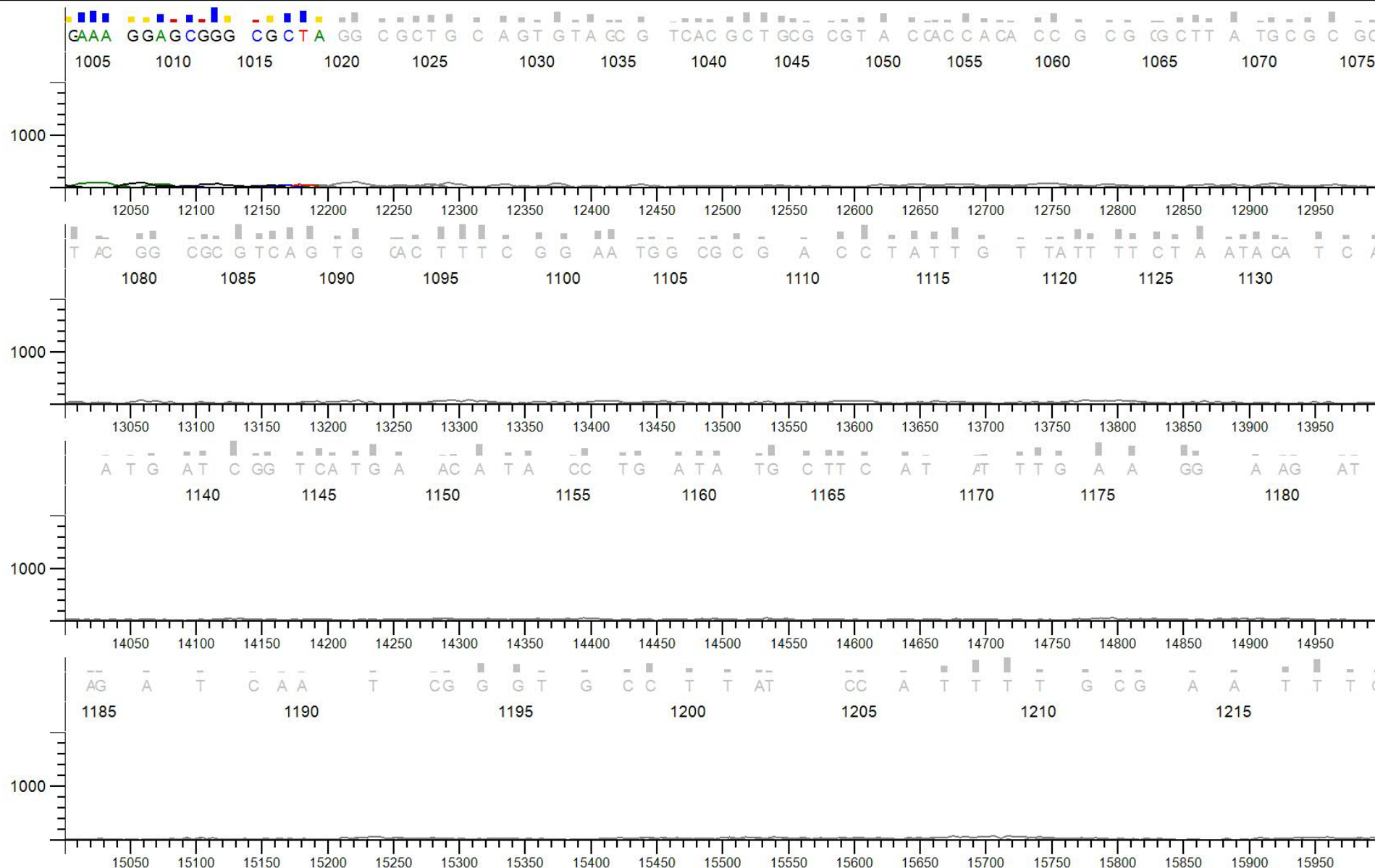

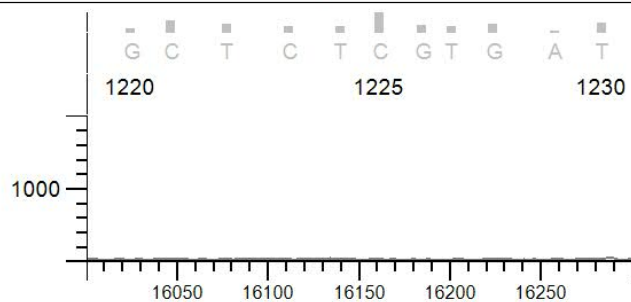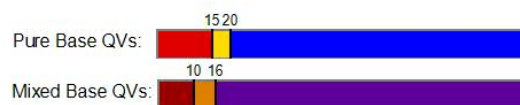

Supplement: Figure 3—source data 1. [file elife-69916-fig3-data1.zip › Figure 3A_Source data2_Bisulphite sequencing data_plasmid/RBK_PDI1_BSF_1.5_T7FOR.pdf]

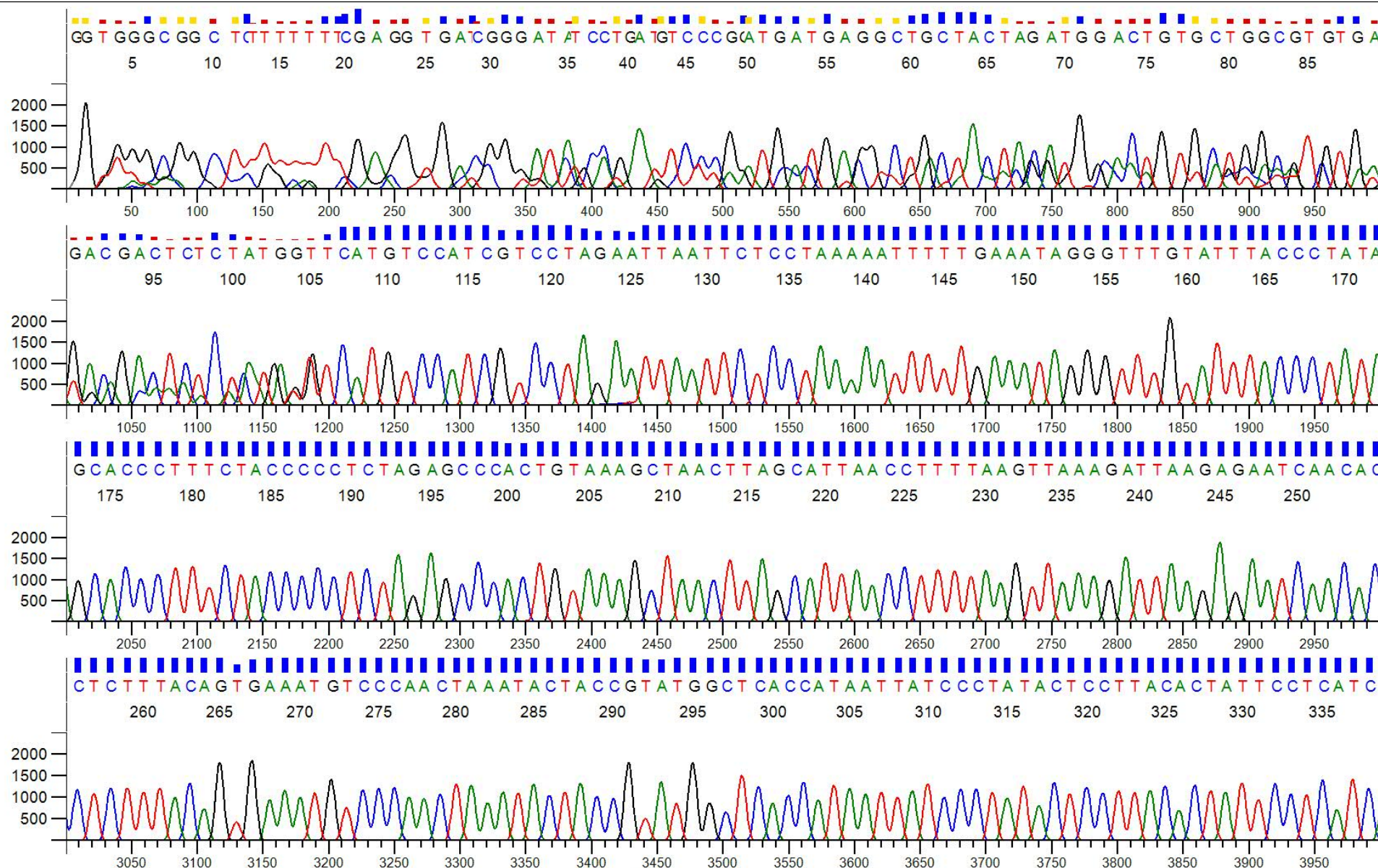

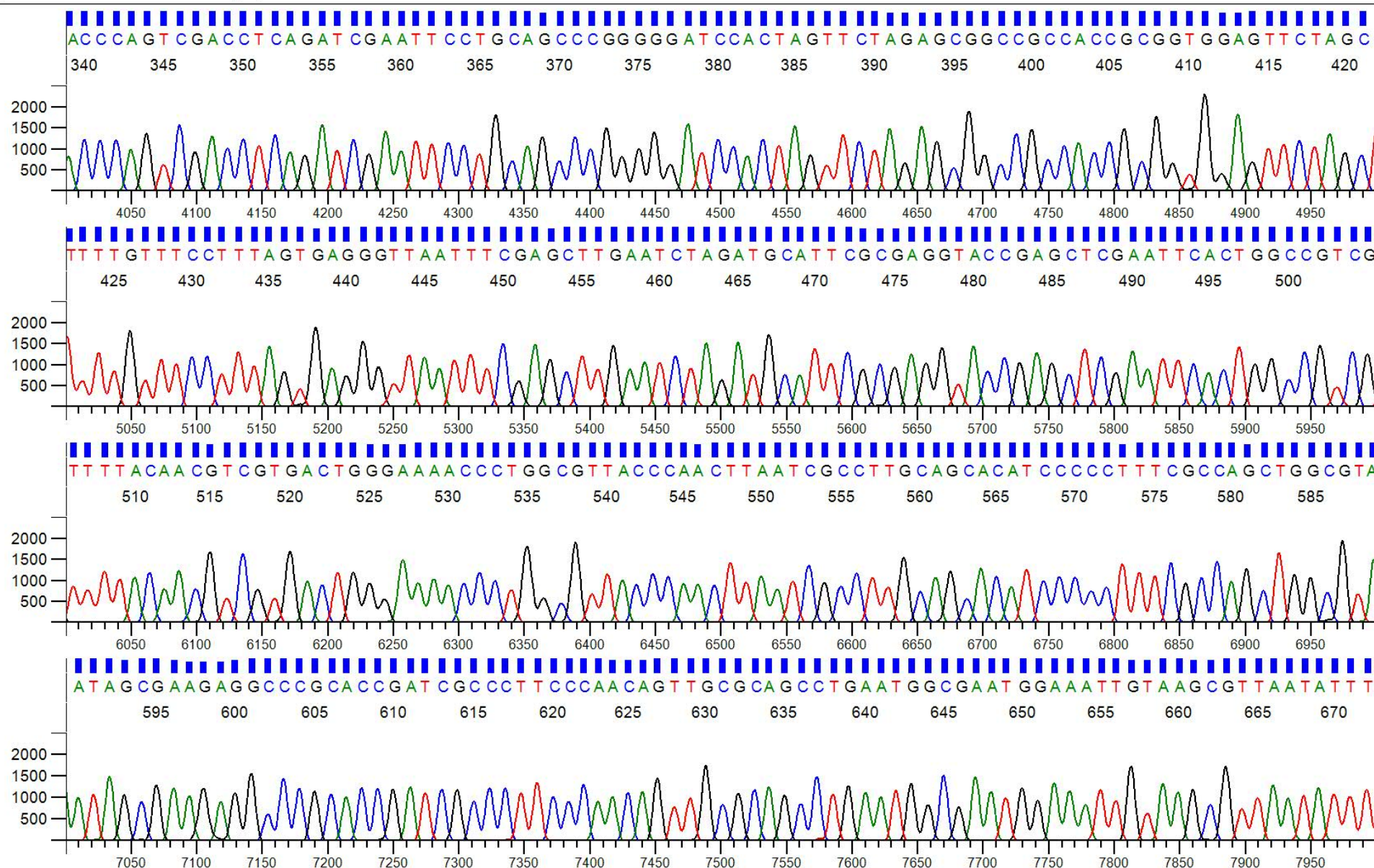

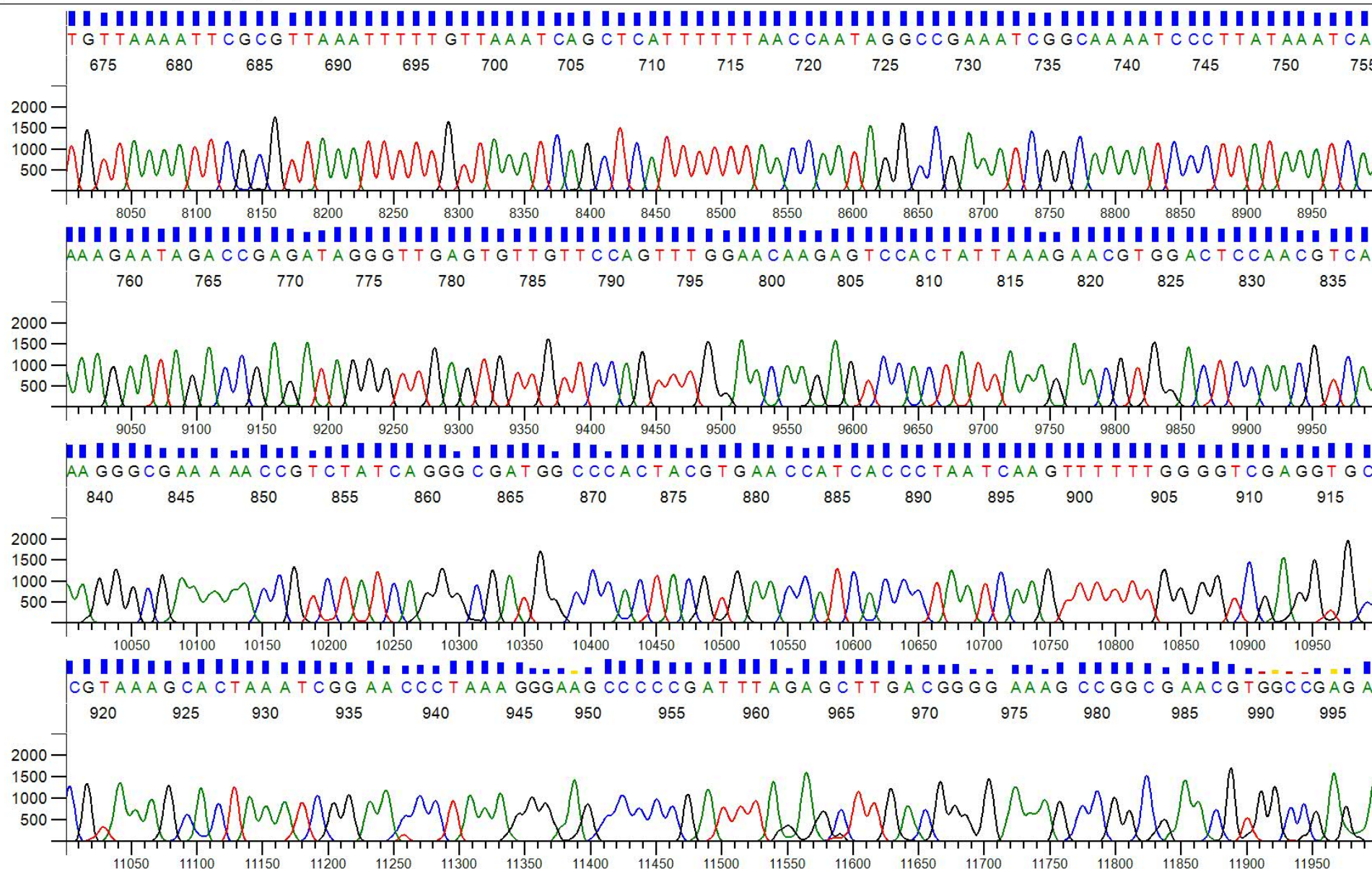

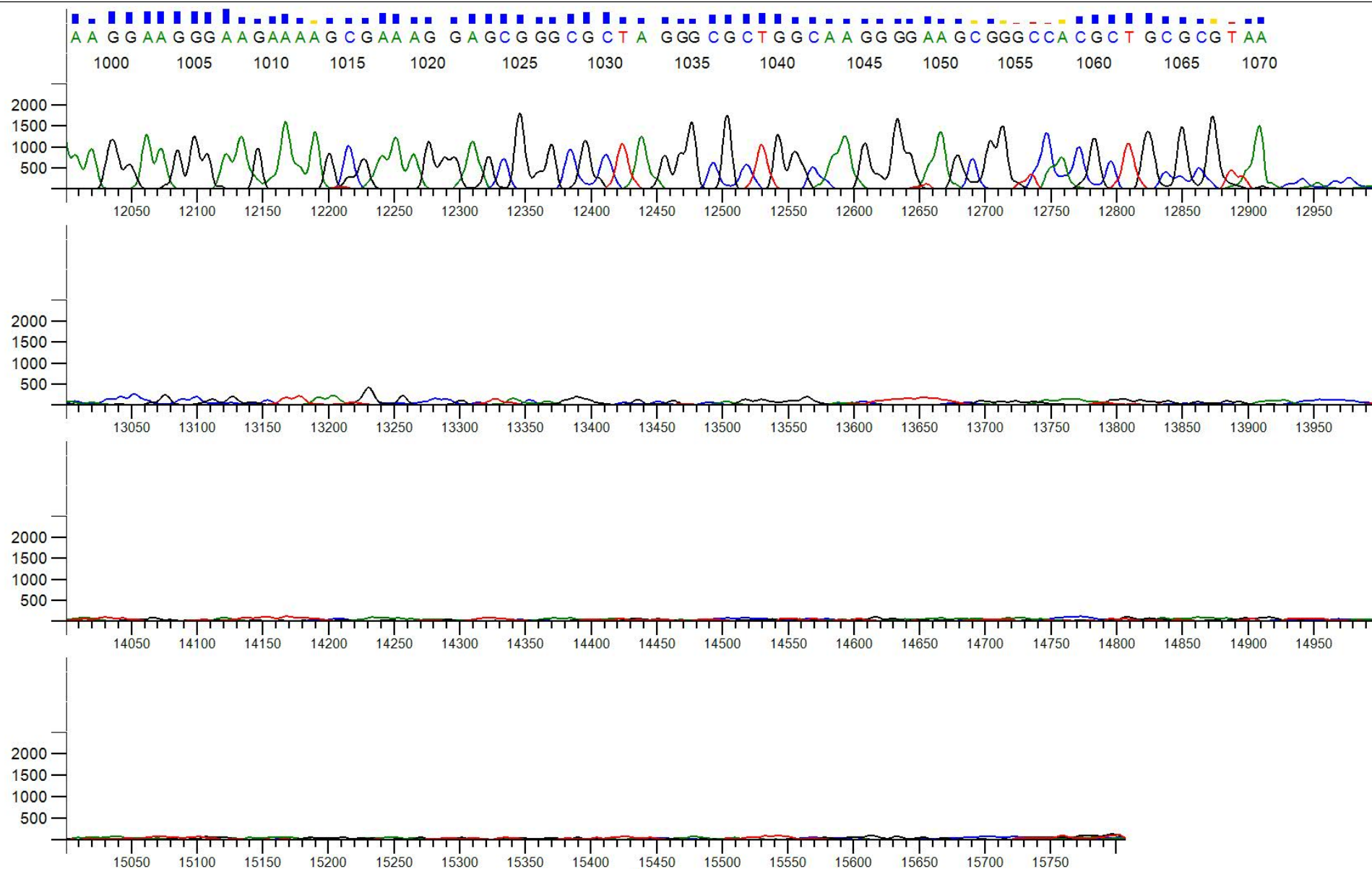

Supplement: Figure 3—source data 1. [file elife-69916-fig3-data1.zip › Figure 3A_Source data2_Bisulphite sequencing data_plasmid/SD-PDT1-BSF-3.5_T7FOR-A11.pdf]

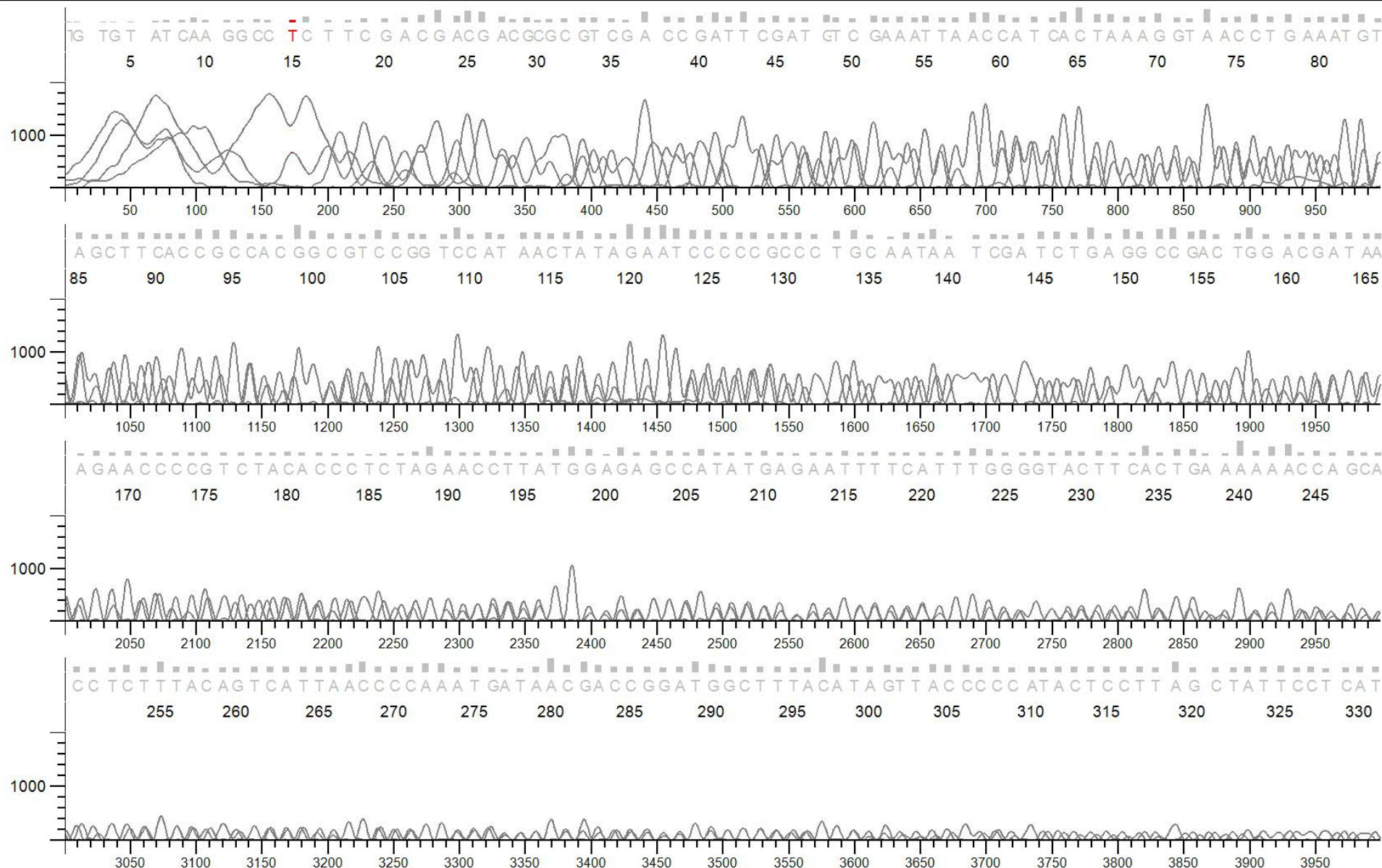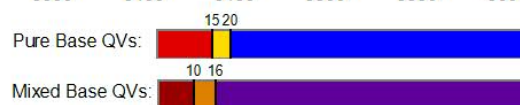

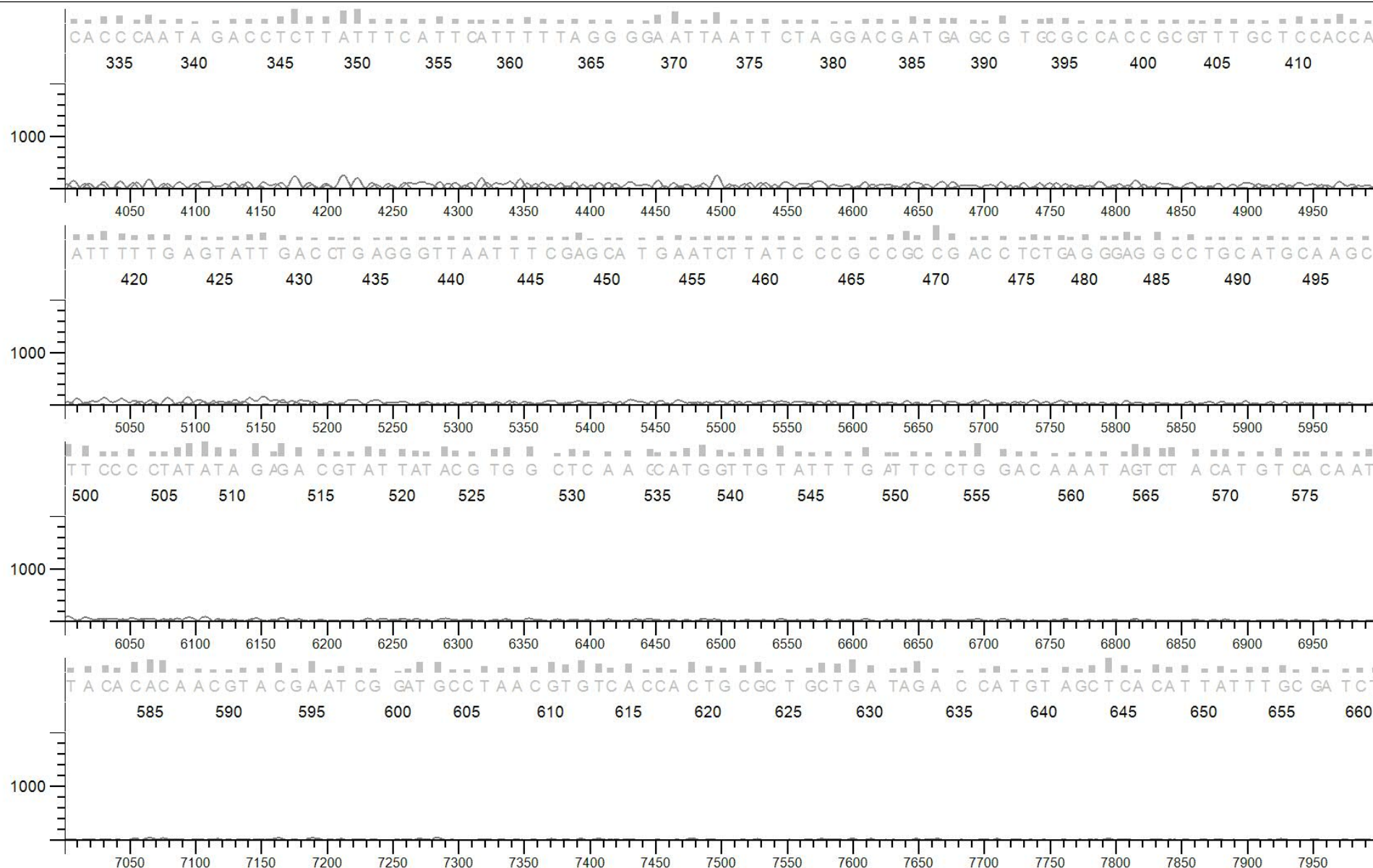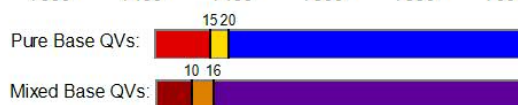

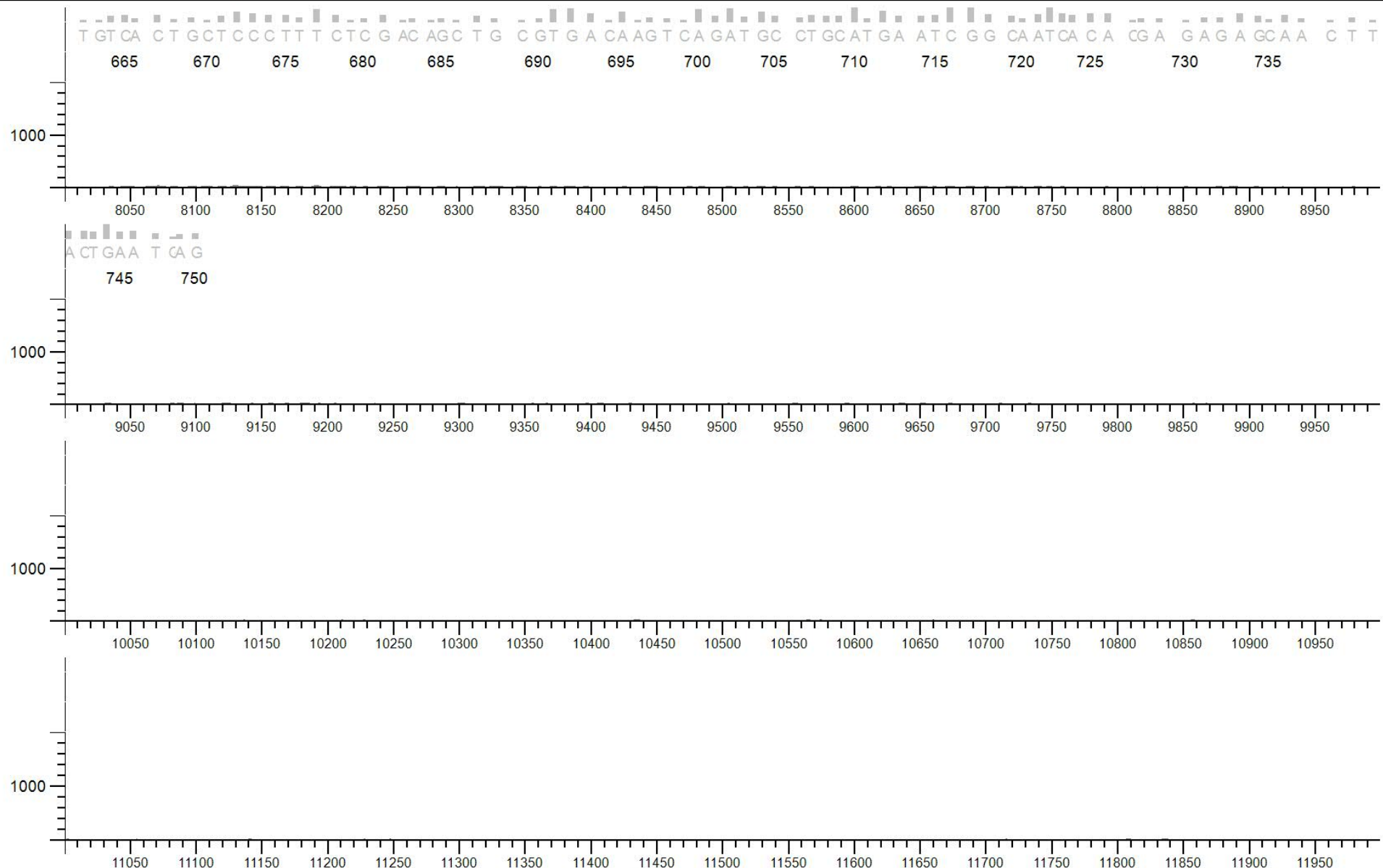

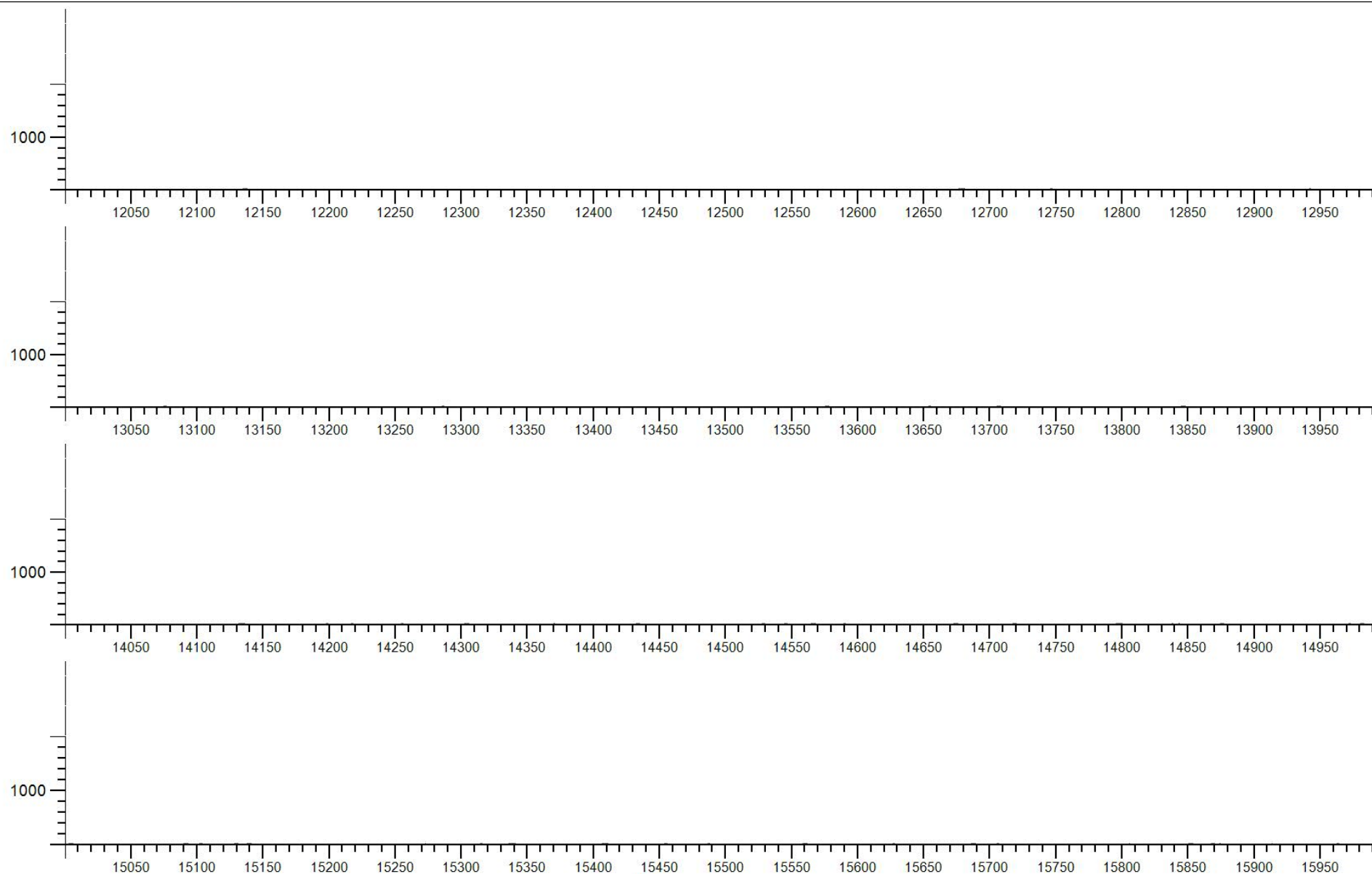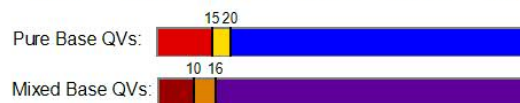

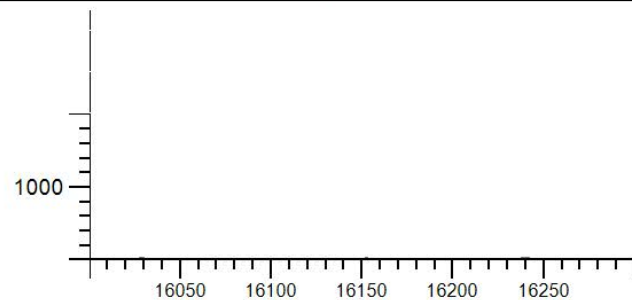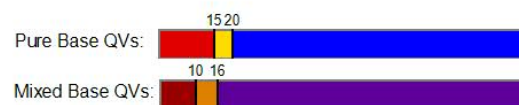

Supplement: Figure 3—source data 1. [file elife-69916-fig3-data1.zip › Figure 3A_Source data2_Bisulphite sequencing data_plasmid/SD_PDI1_BSF_2.4_T7FOR-G02.pdf]

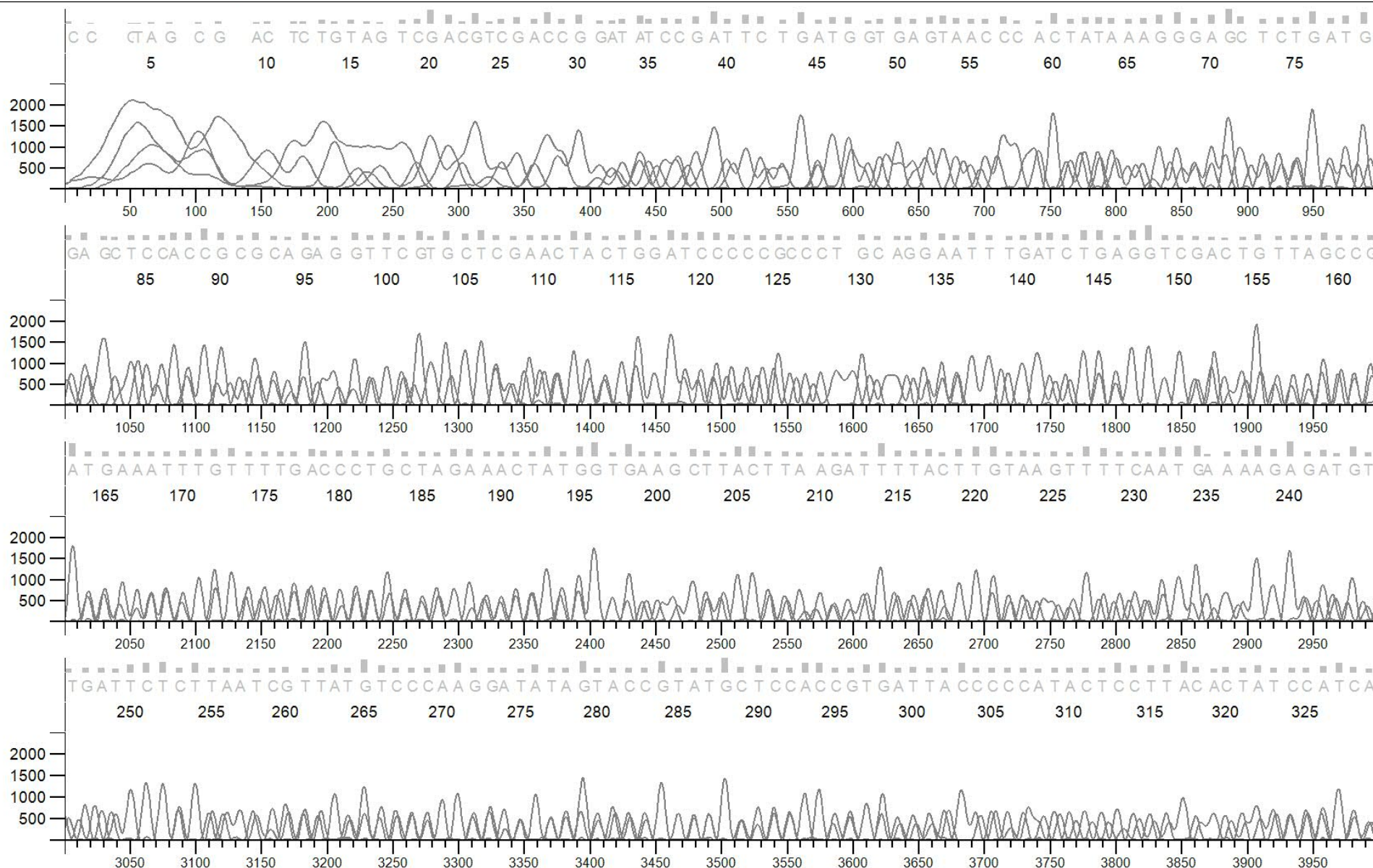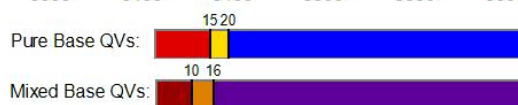

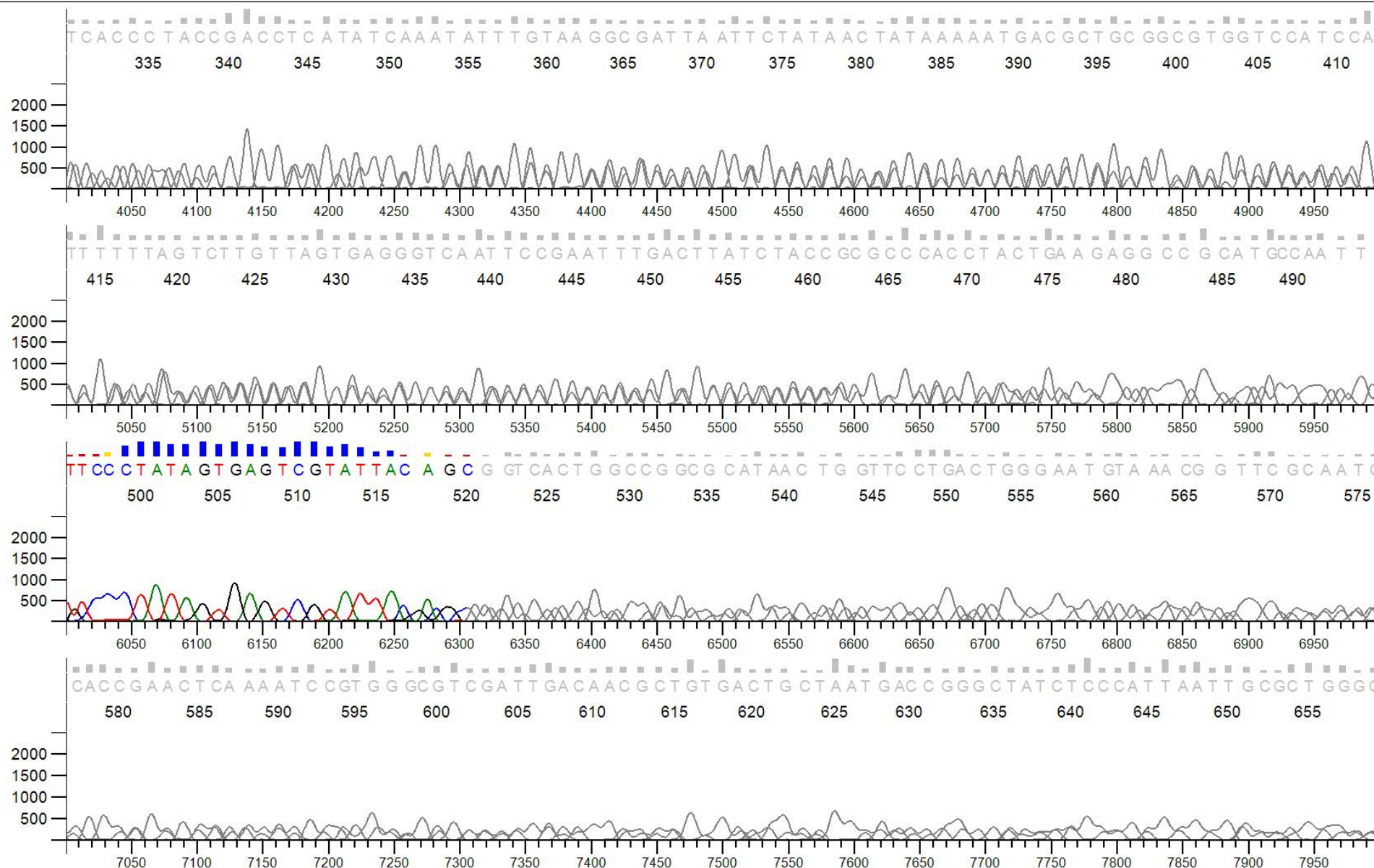

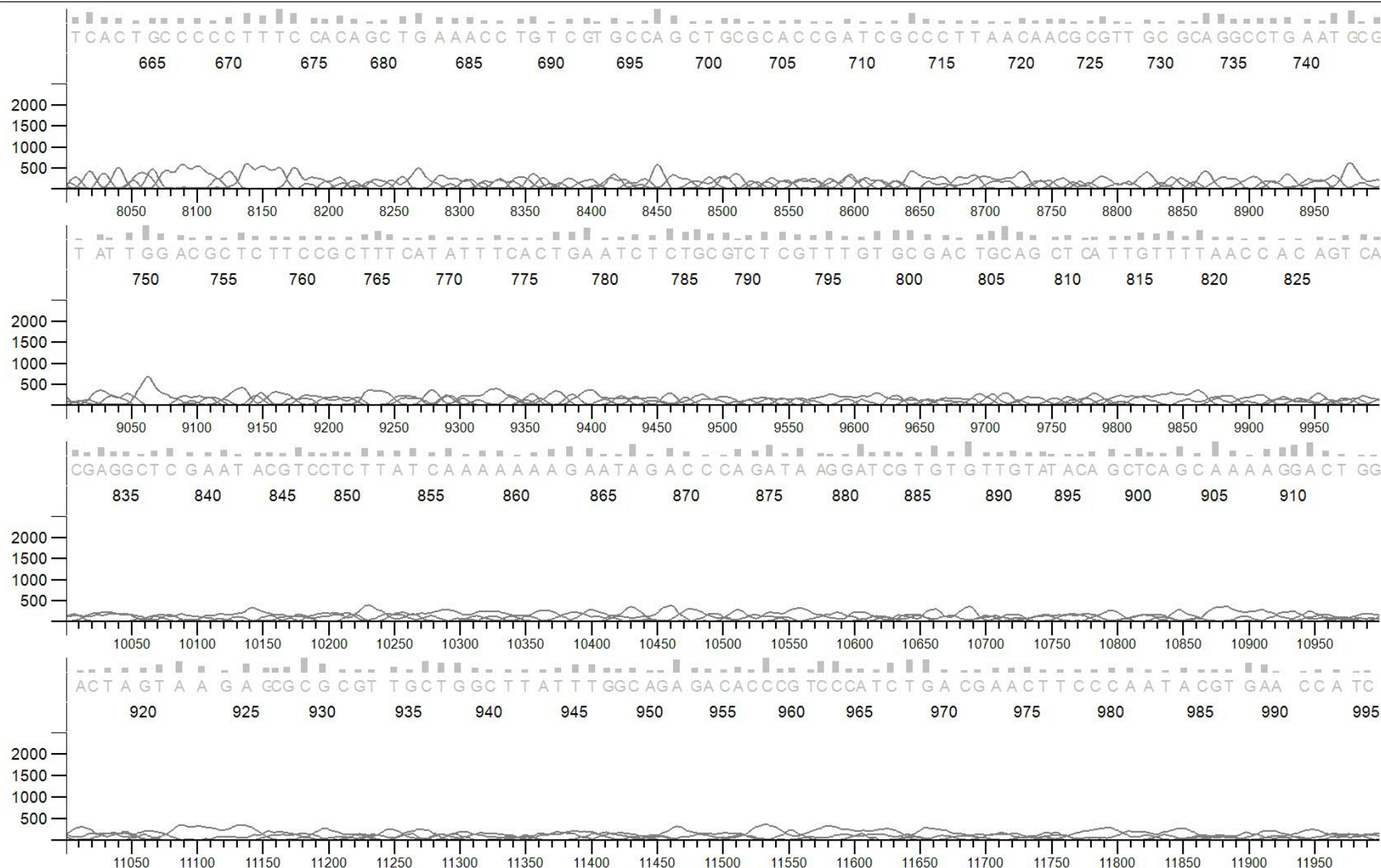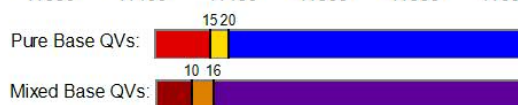

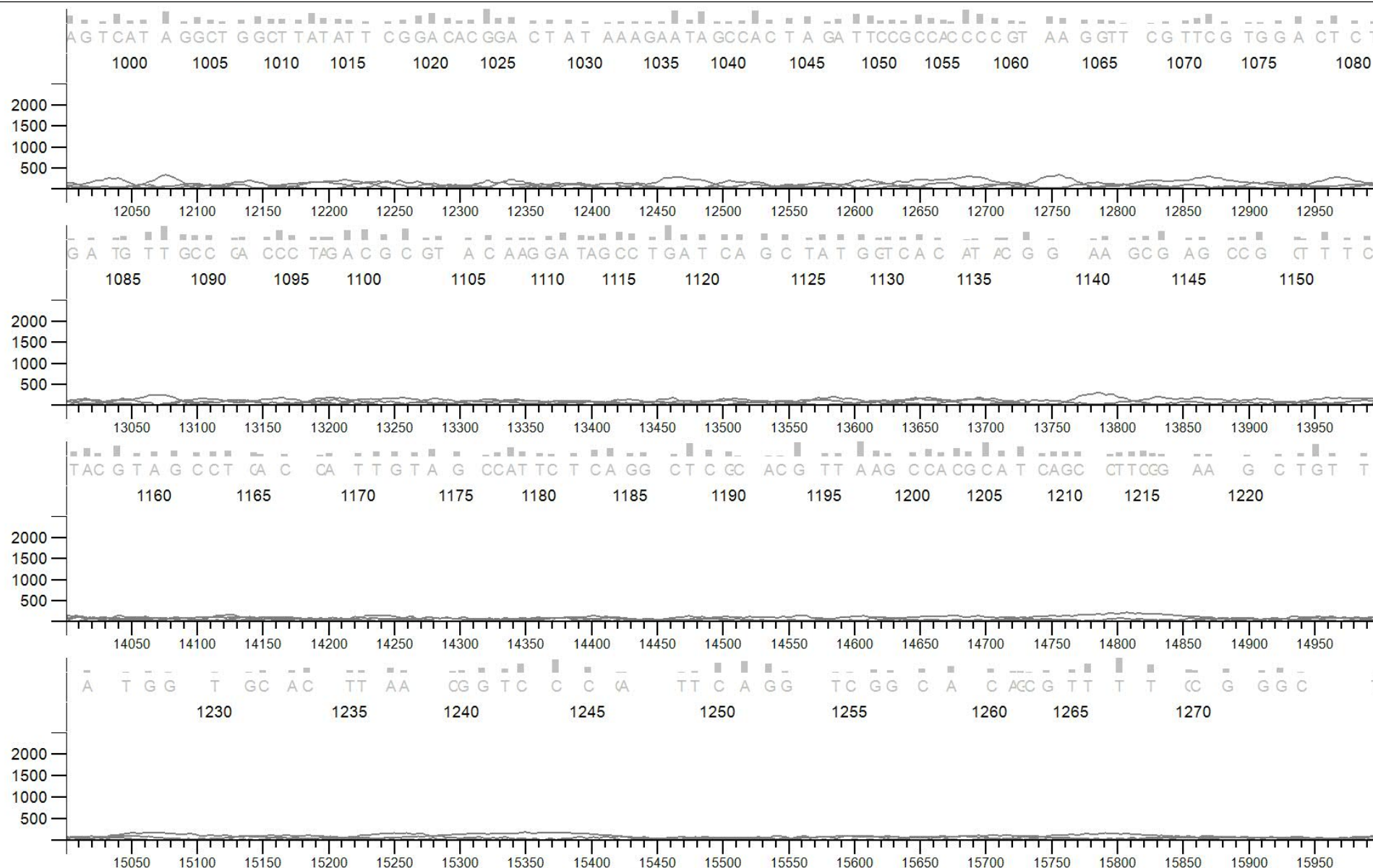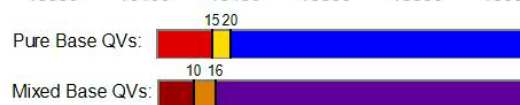

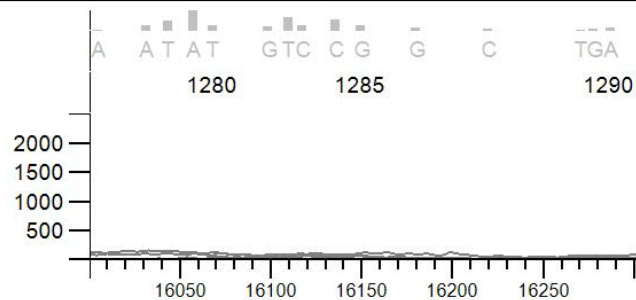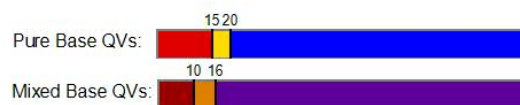

Supplement: Figure 3—source data 1. [file elife-69916-fig3-data1.zip › Figure 3A_Source data2_Bisulphite sequencing data_plasmid/SD-PDT1-BSF-3.10_T7FOR-F11.pdf]

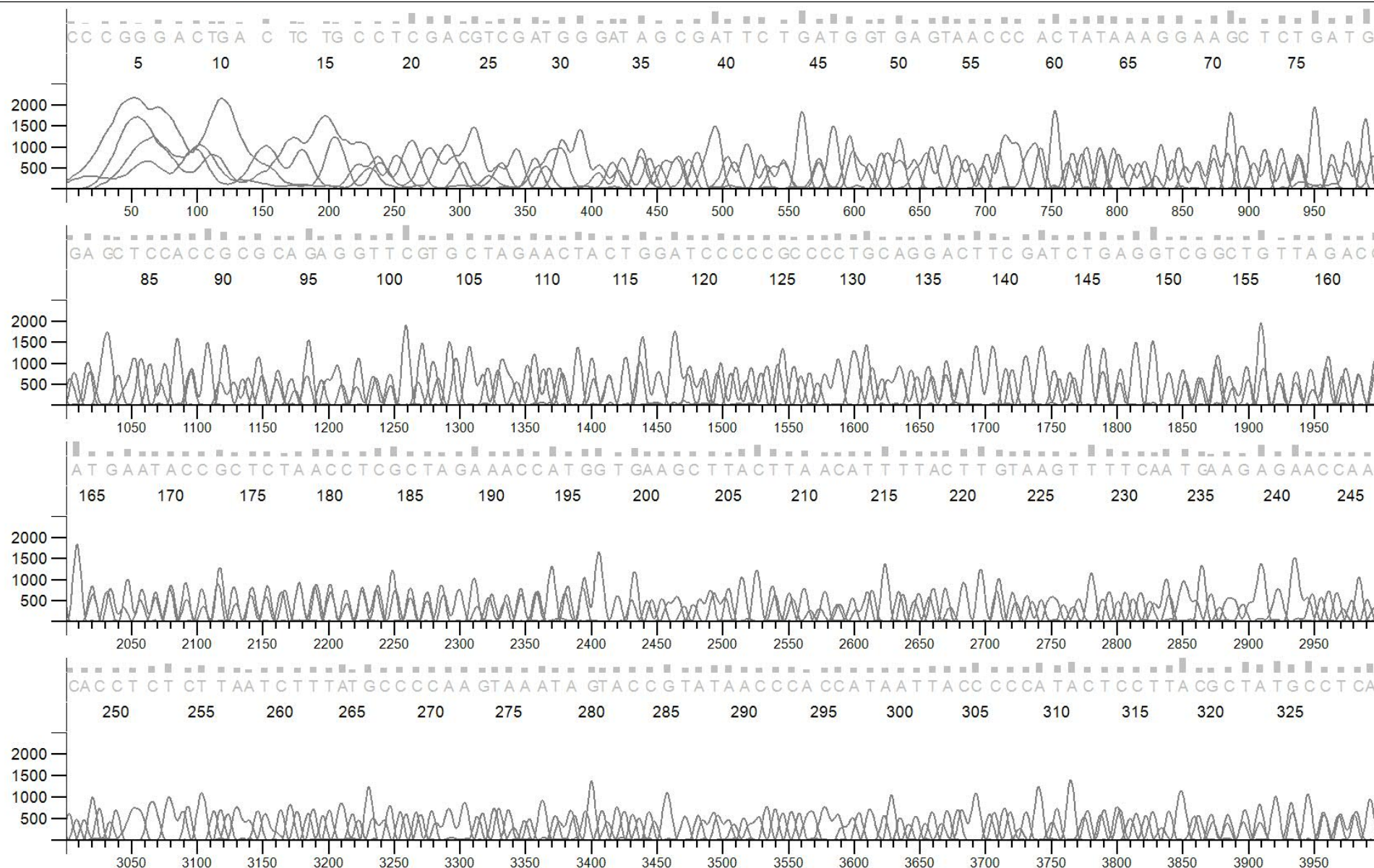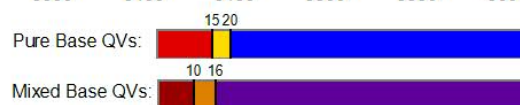

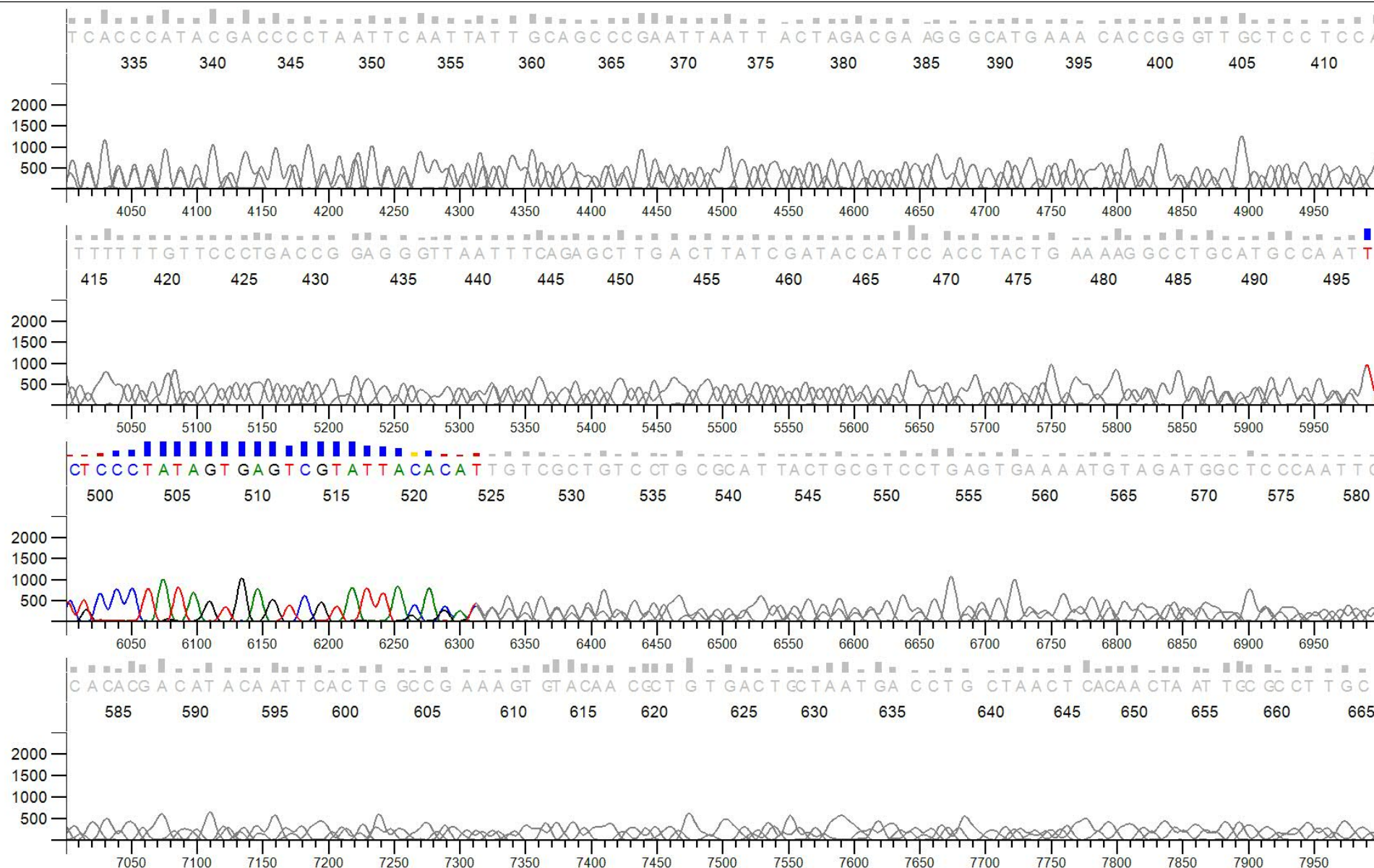

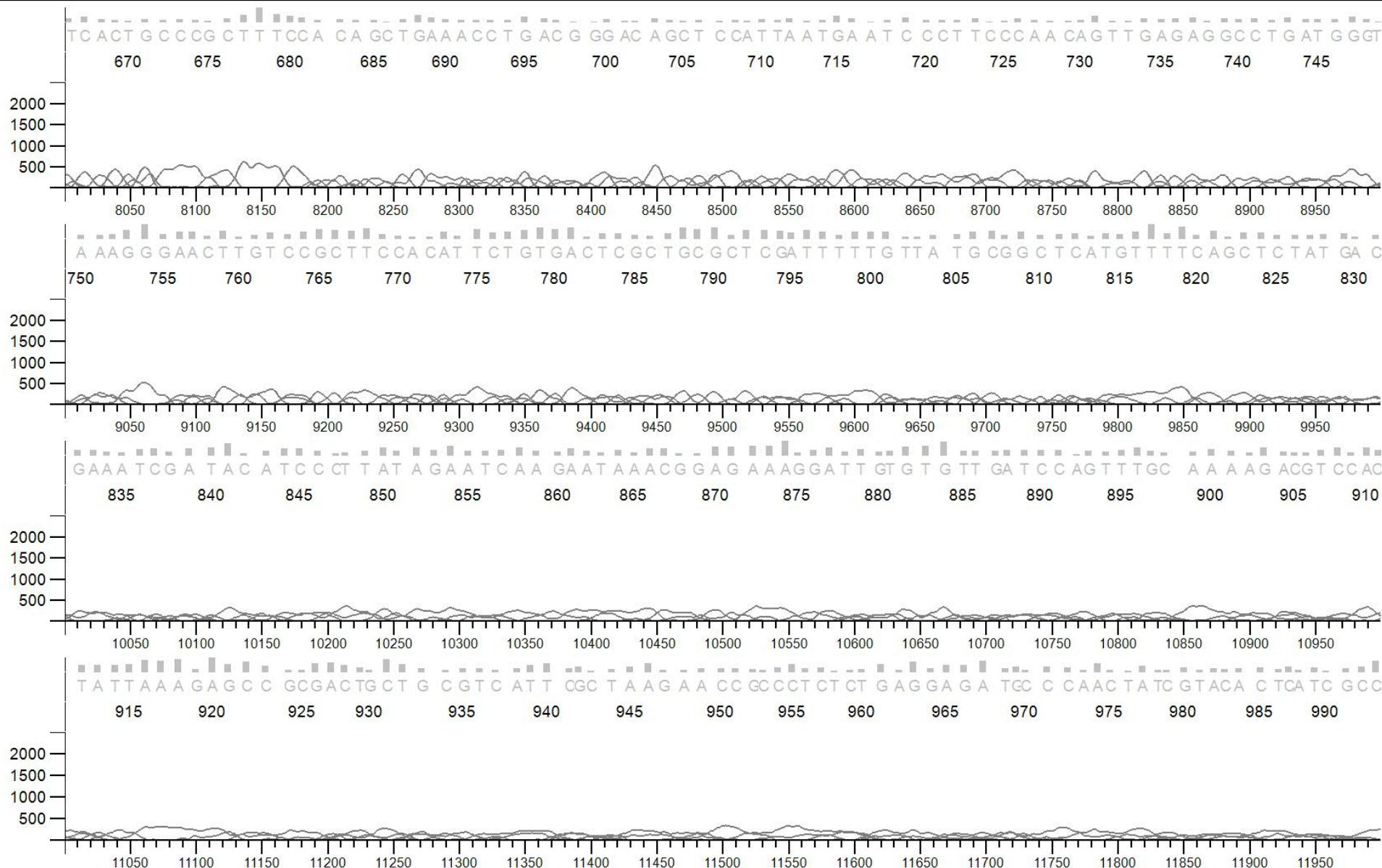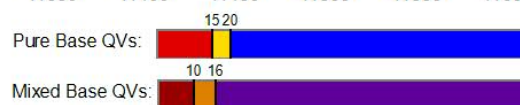

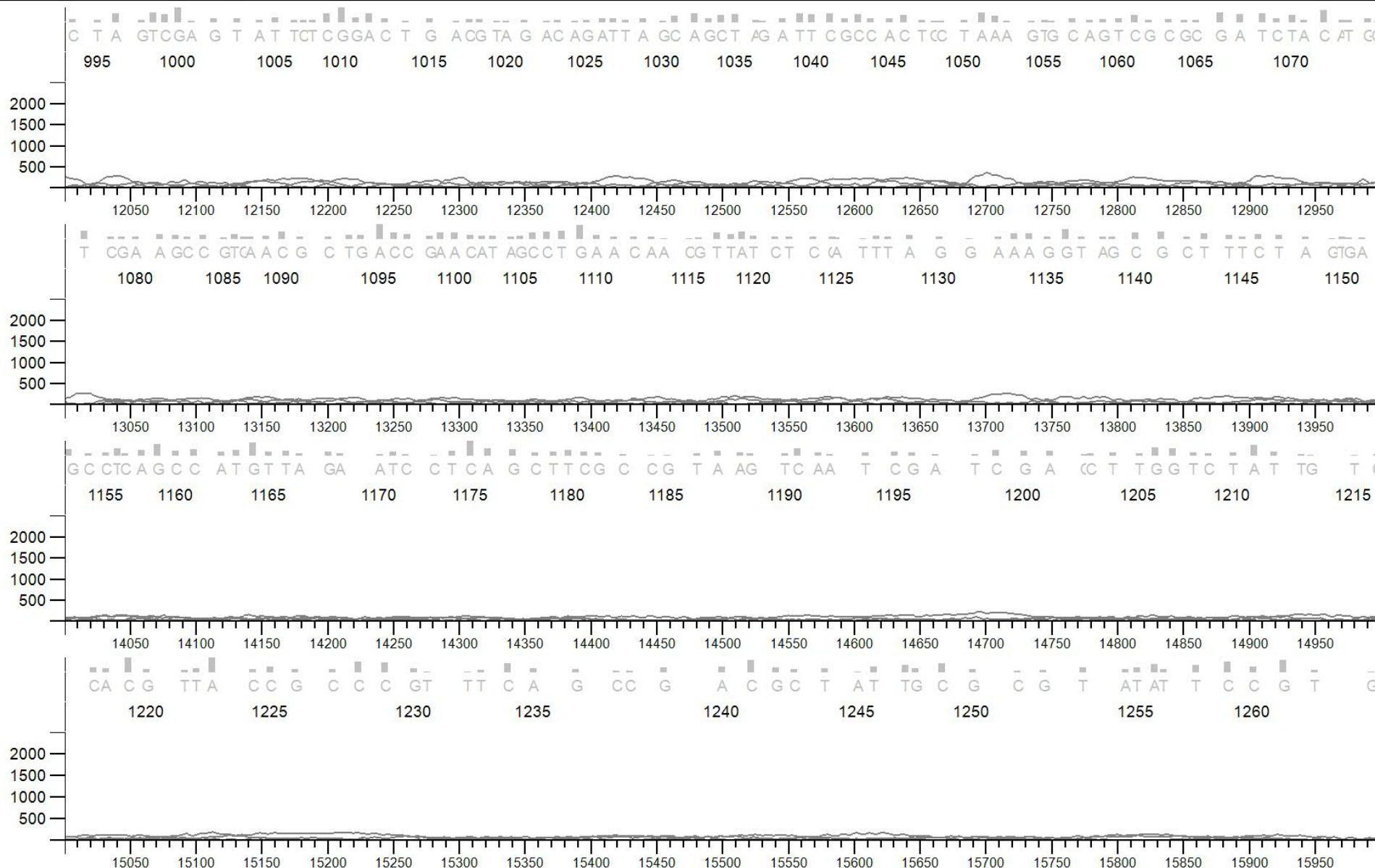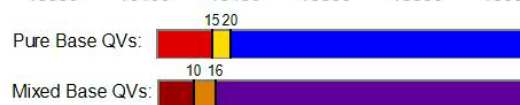

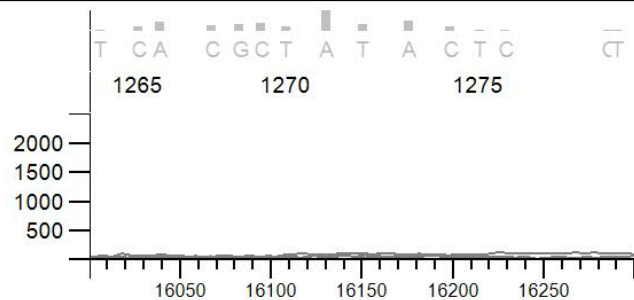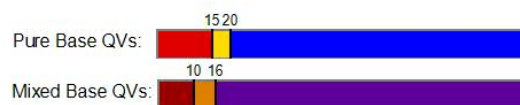

Supplement: Figure 3—source data 1. [file elife-69916-fig3-data1.zip › Figure 3A_Source data2_Bisulphite sequencing data_plasmid/SD-PDT1-BSF-3.8_T7FOR-D11.pdf]

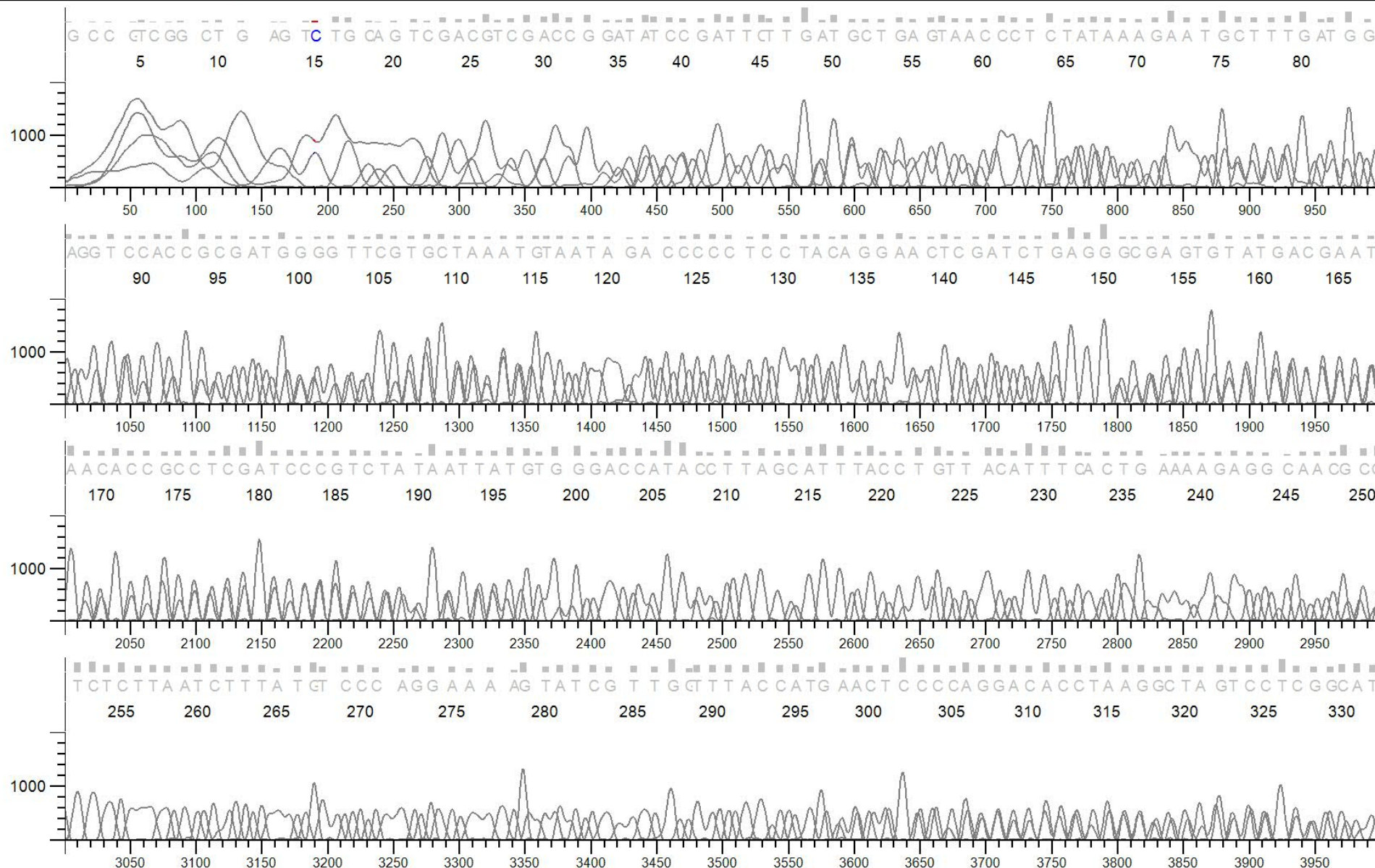

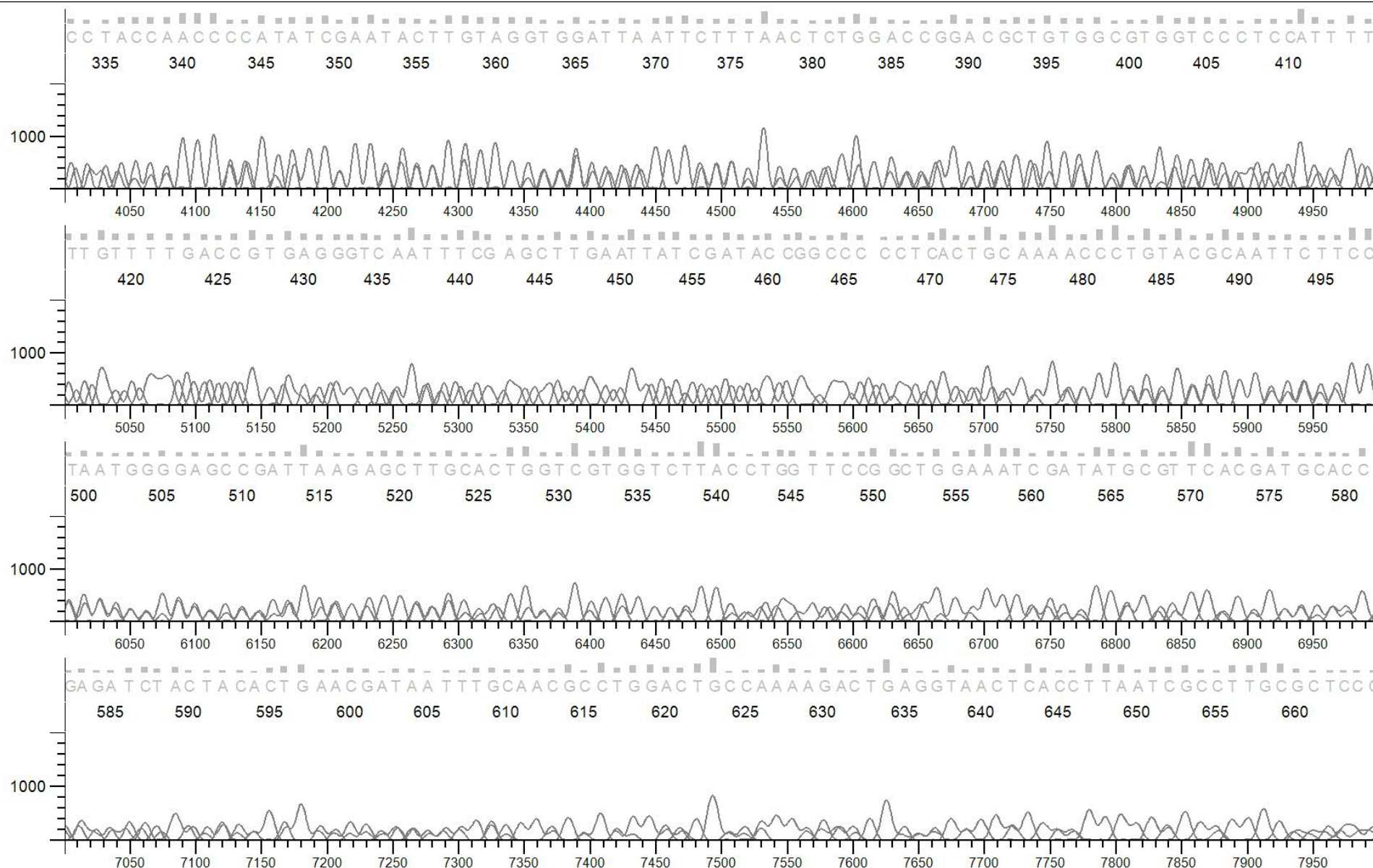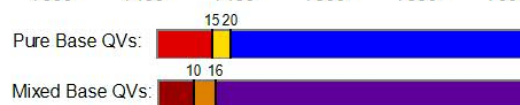

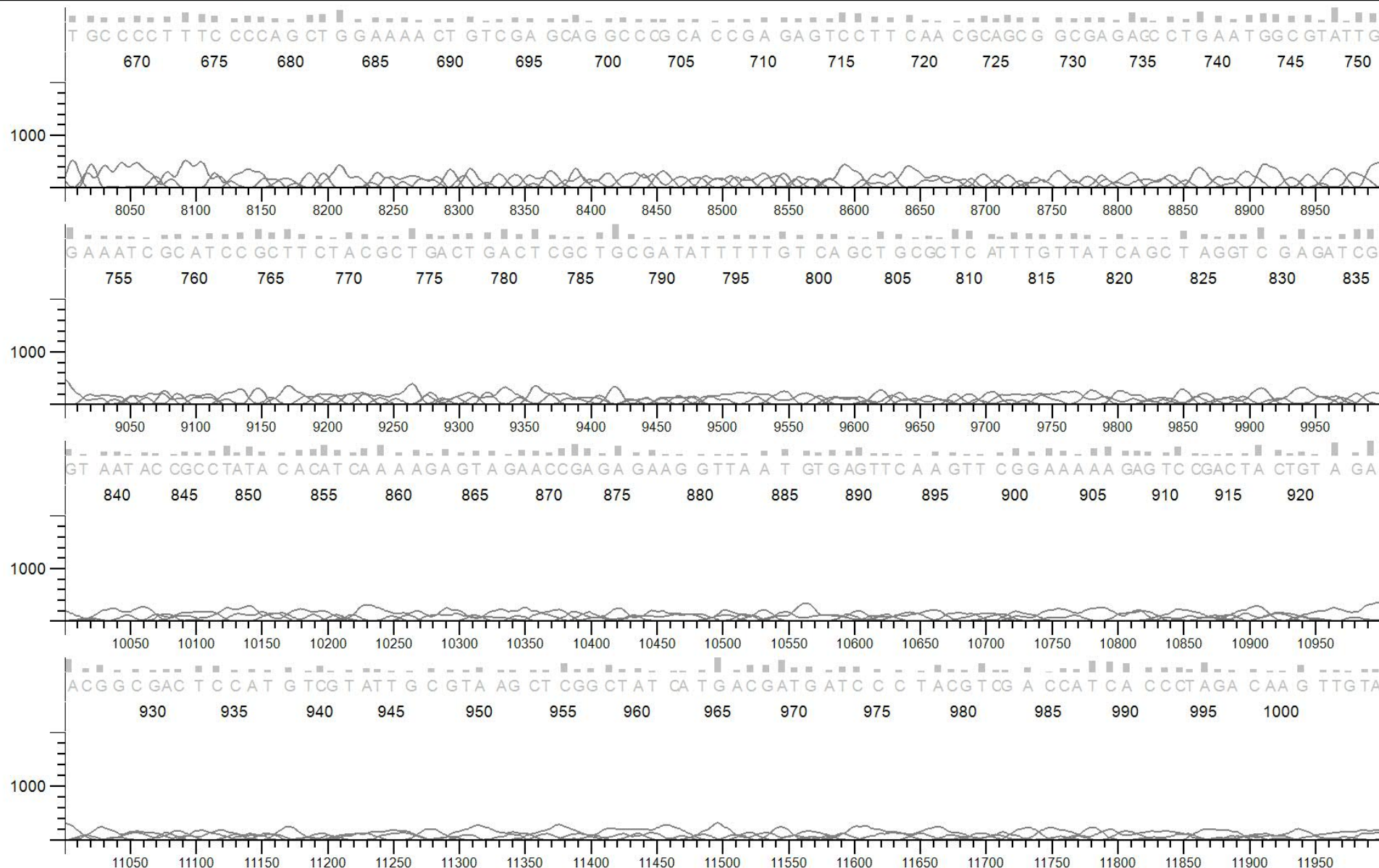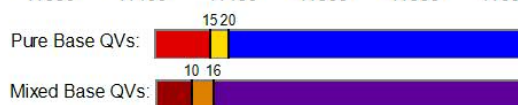

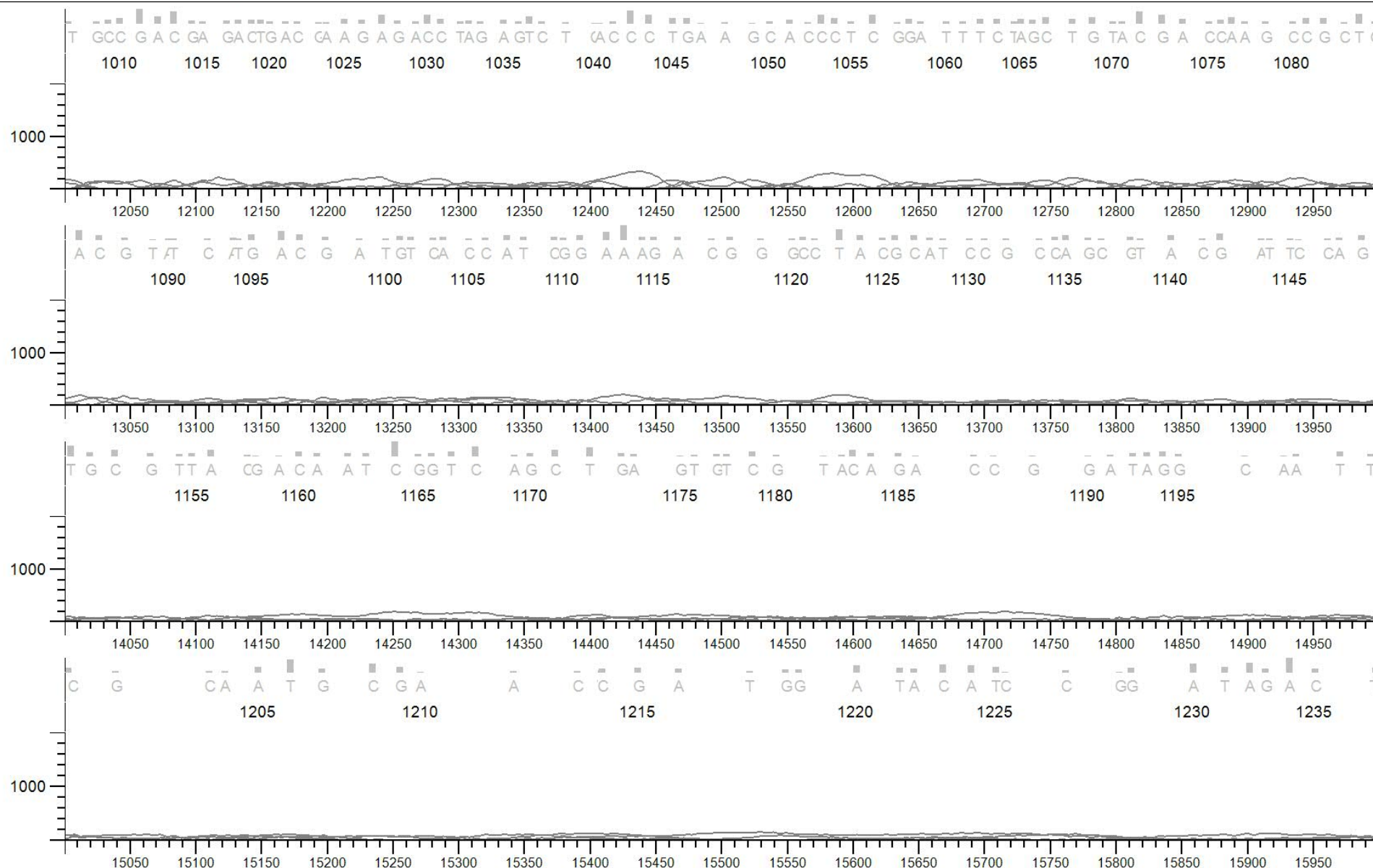

Signal: G:404 A:813 T:660 C:751 AvgSig: 657

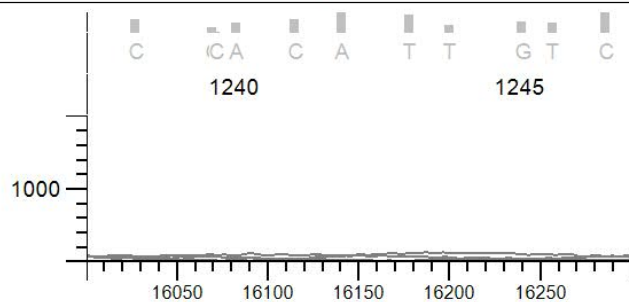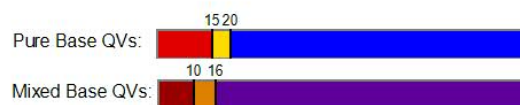

Supplement: Figure 3—source data 1. [file elife-69916-fig3-data1.zip › Figure 3A_Source data2_Bisulphite sequencing data_plasmid/SS4_PDI1_BIS_7_T7FOR-E01.pdf]

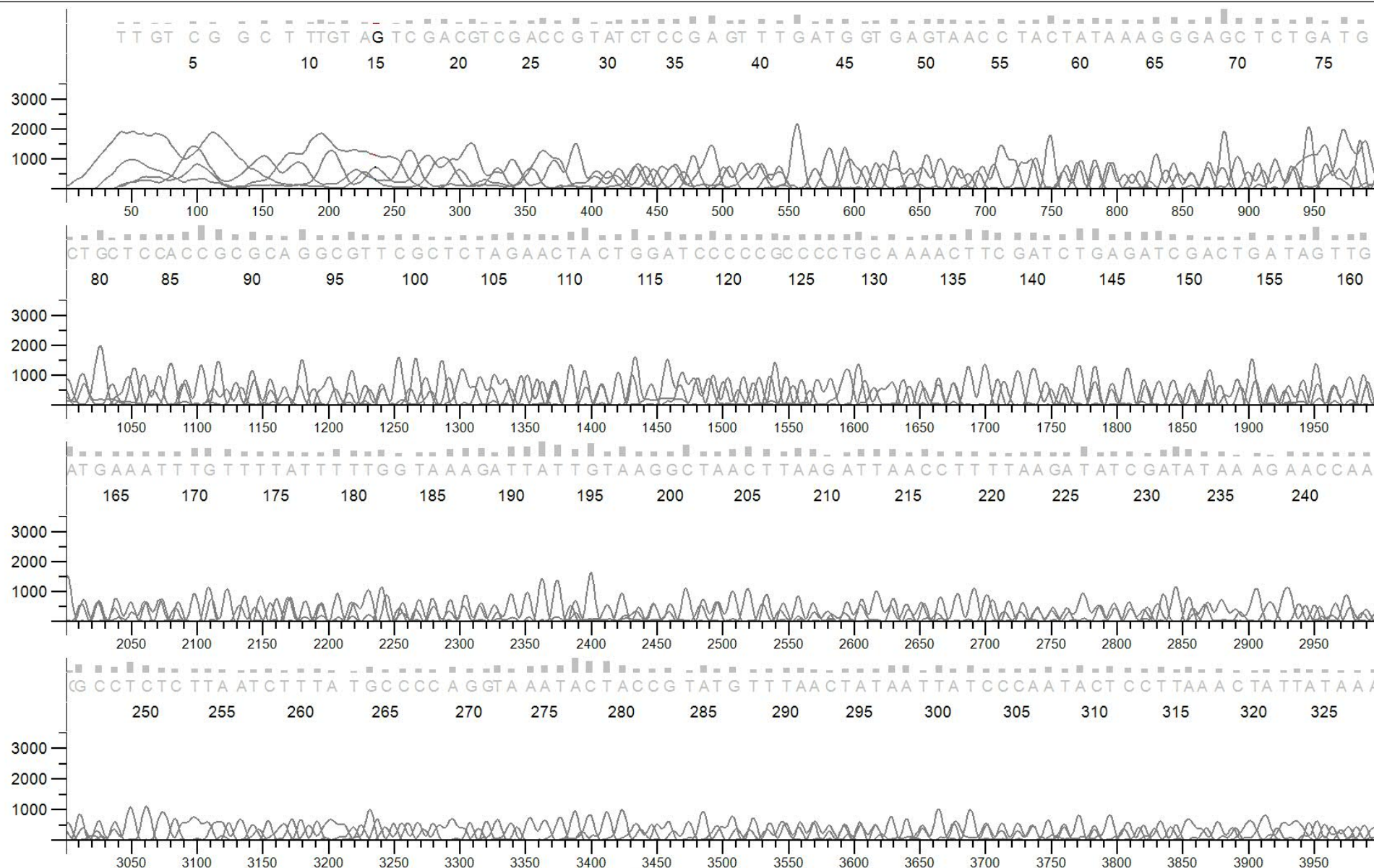

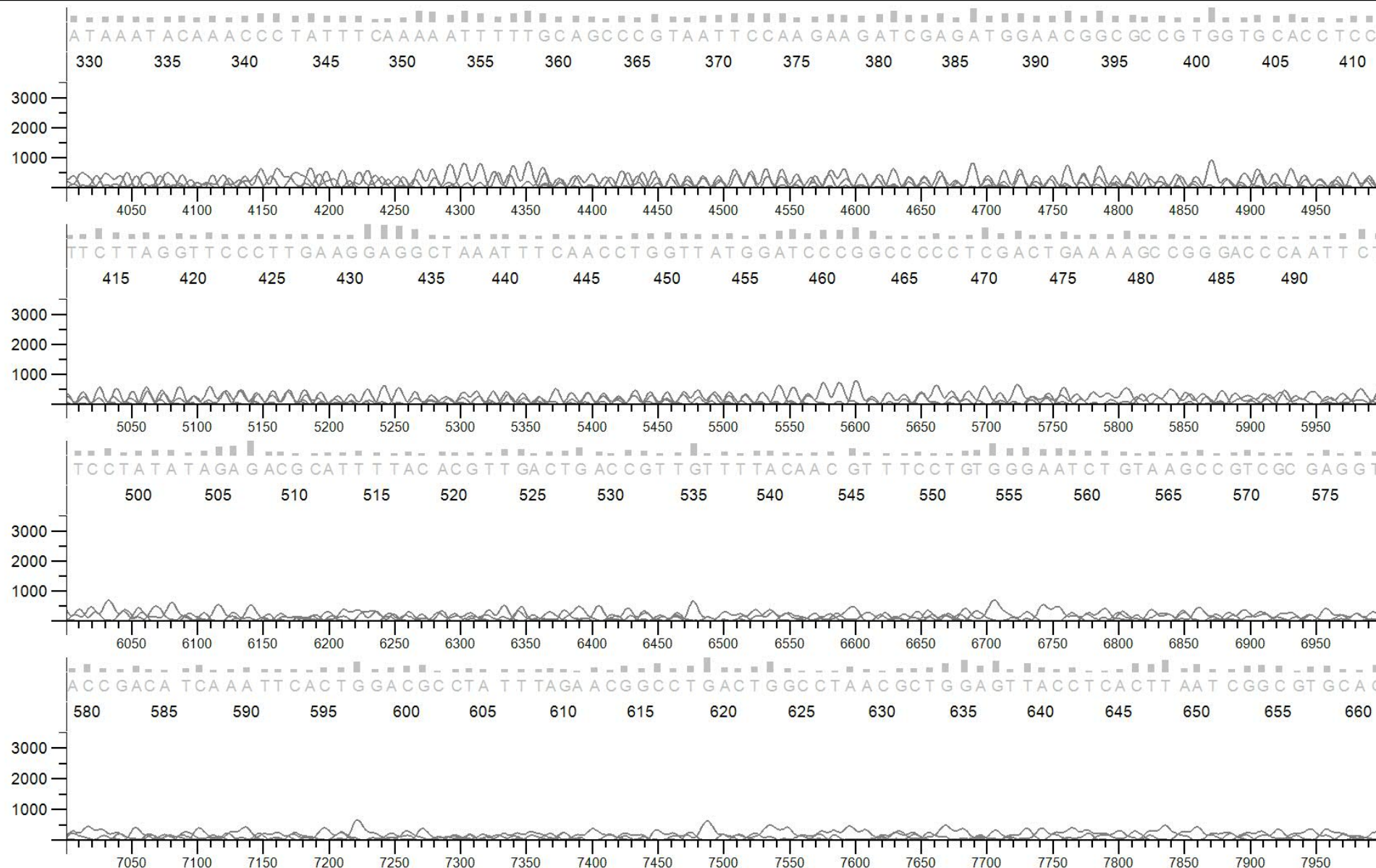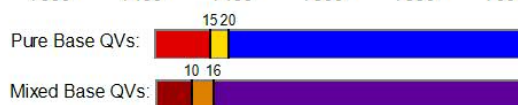

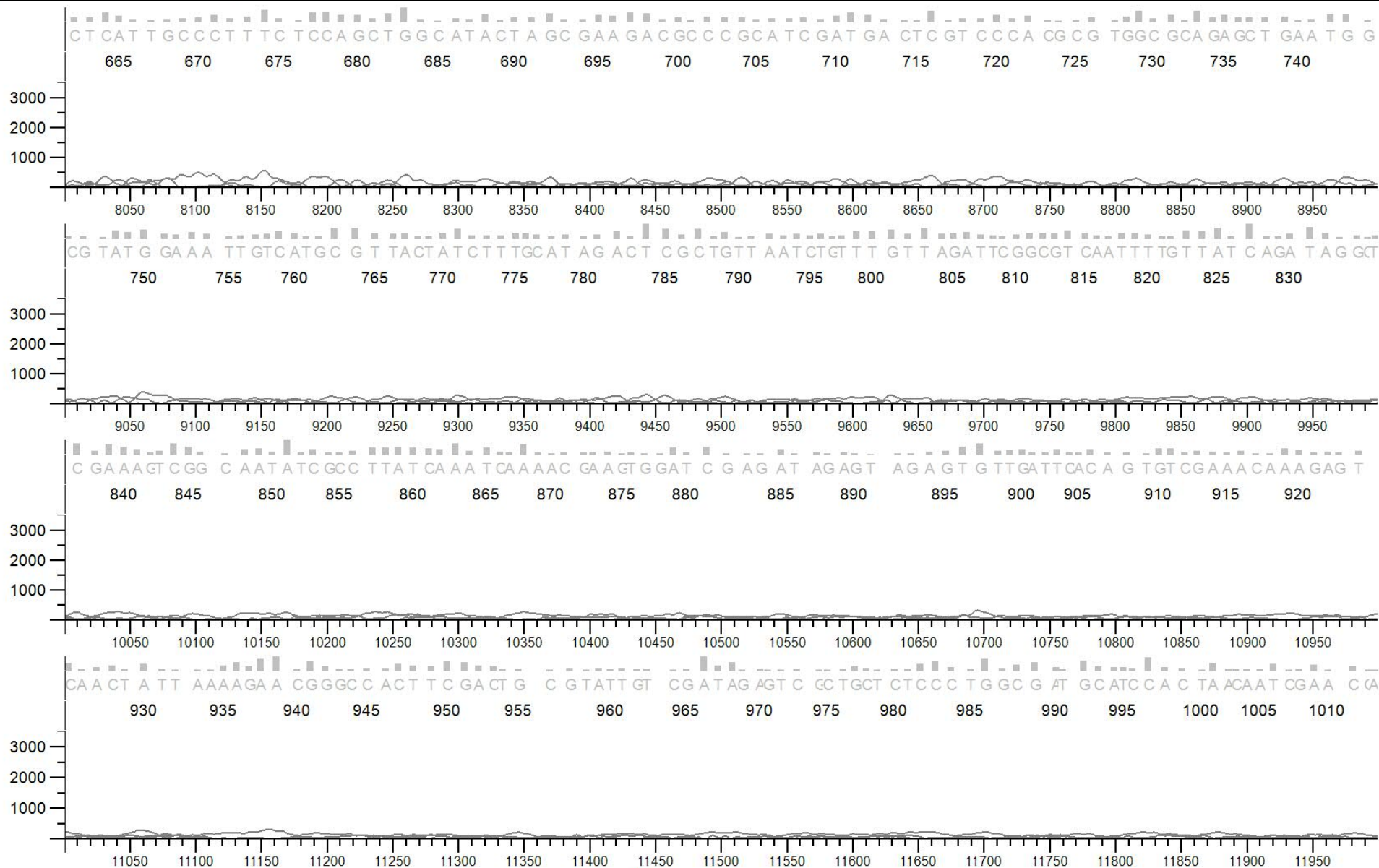

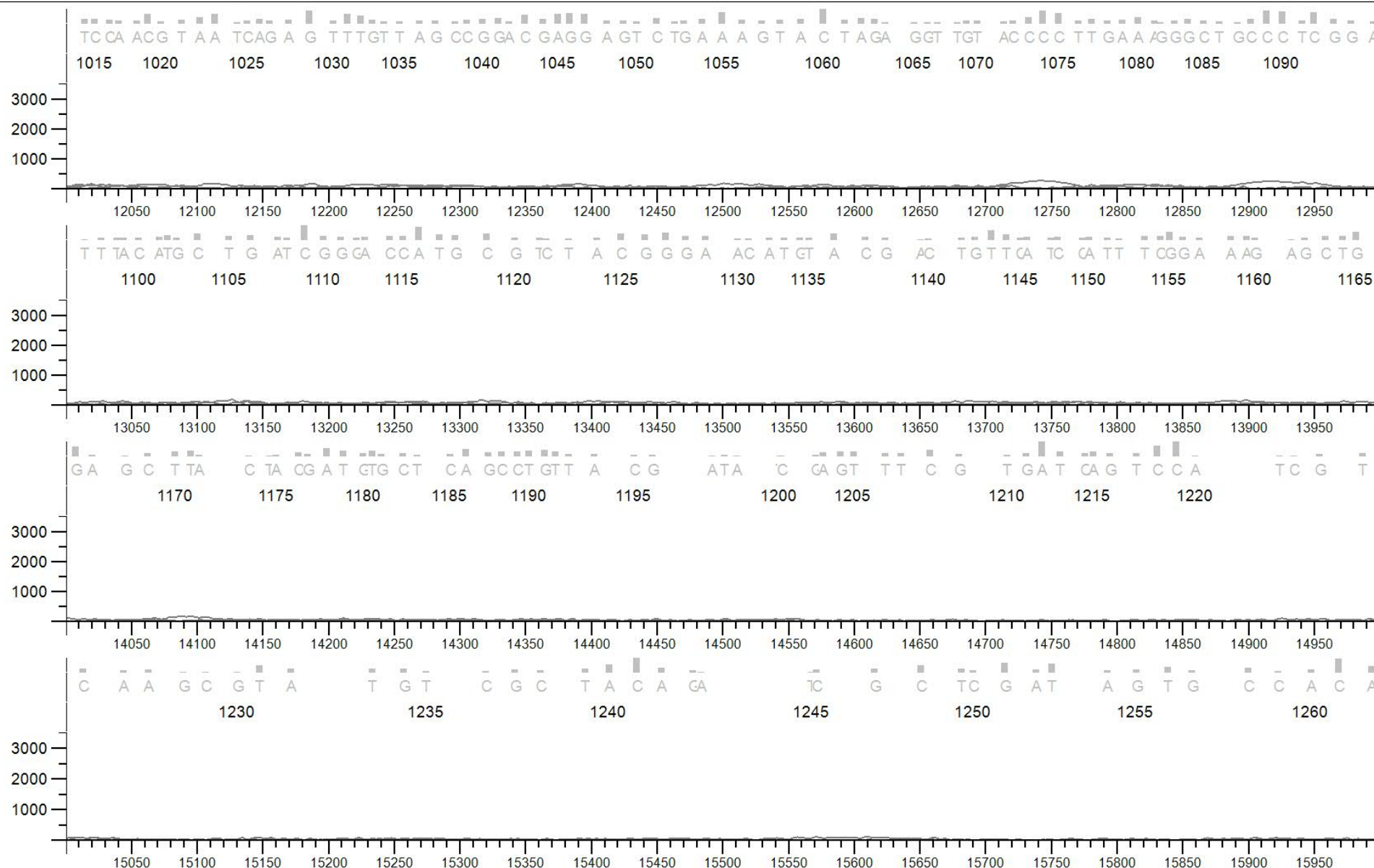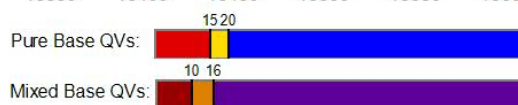

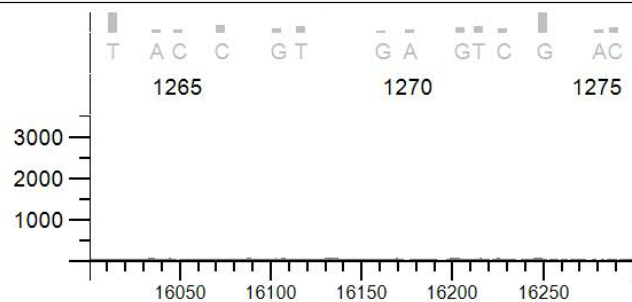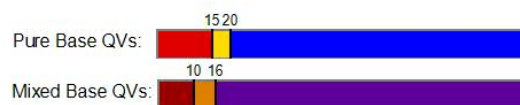

Supplement: Figure 3—source data 1. [file elife-69916-fig3-data1.zip › Figure 3A_Source data2_Bisulphite sequencing data_plasmid/SD-PDI1-BSF-3.16_T7-FOR-F04.pdf]

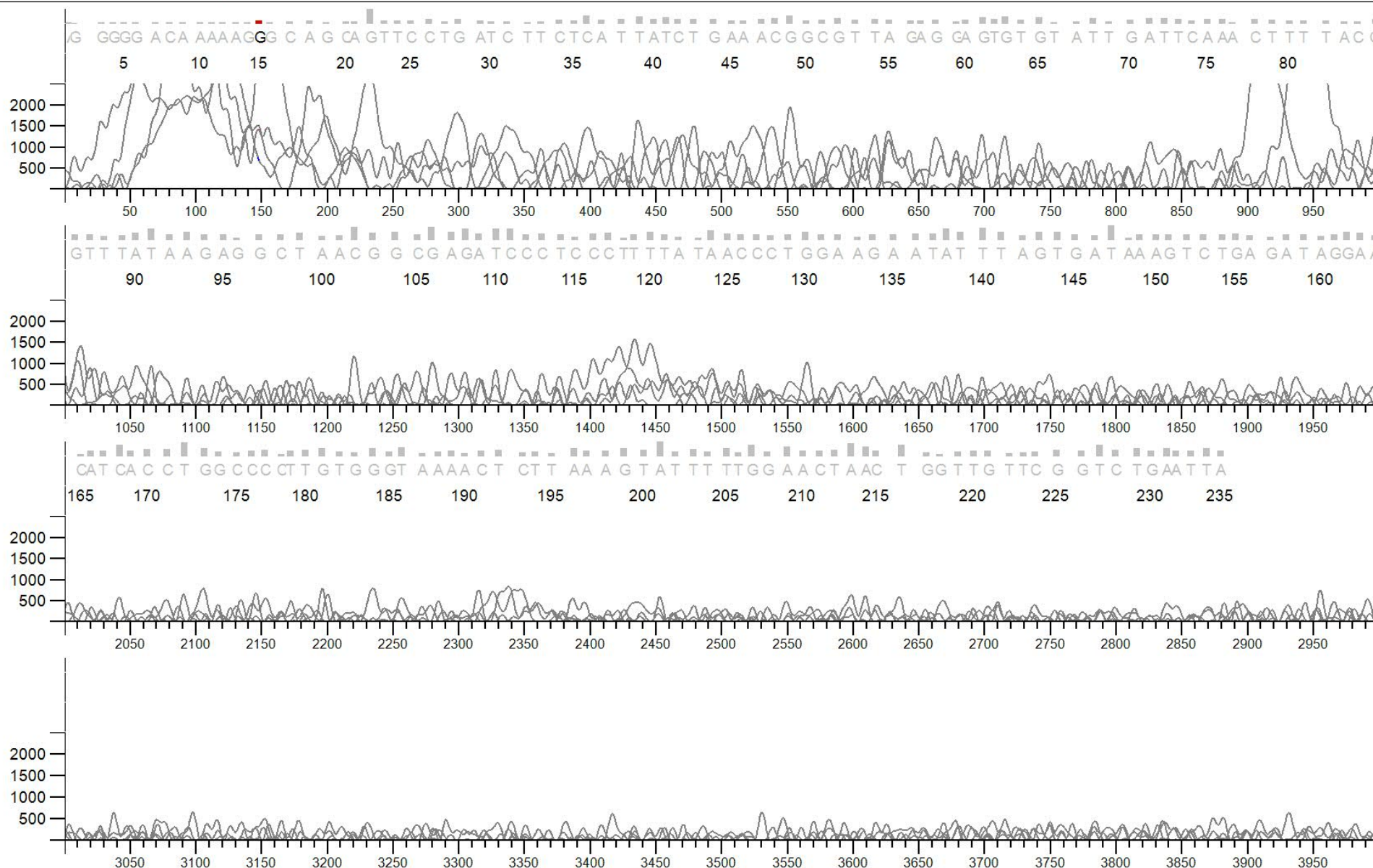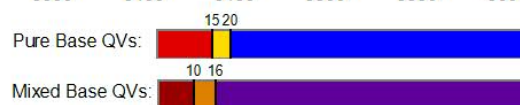

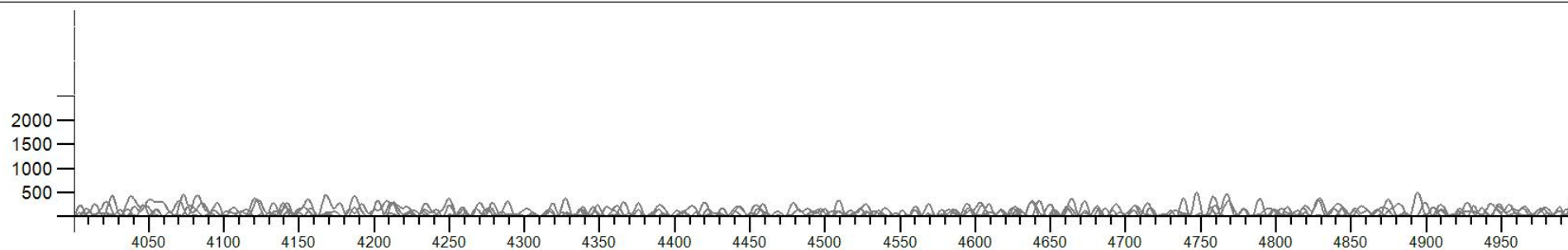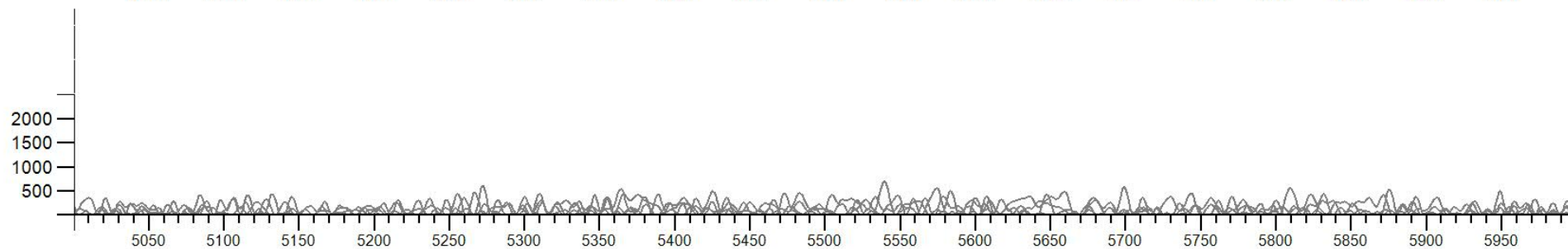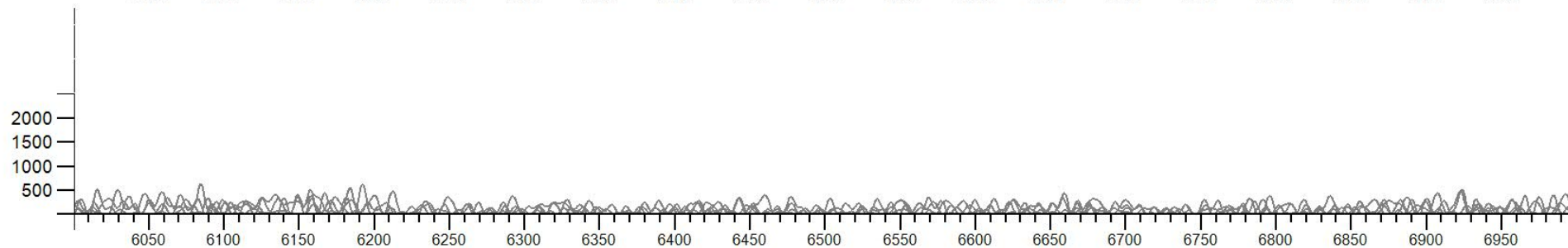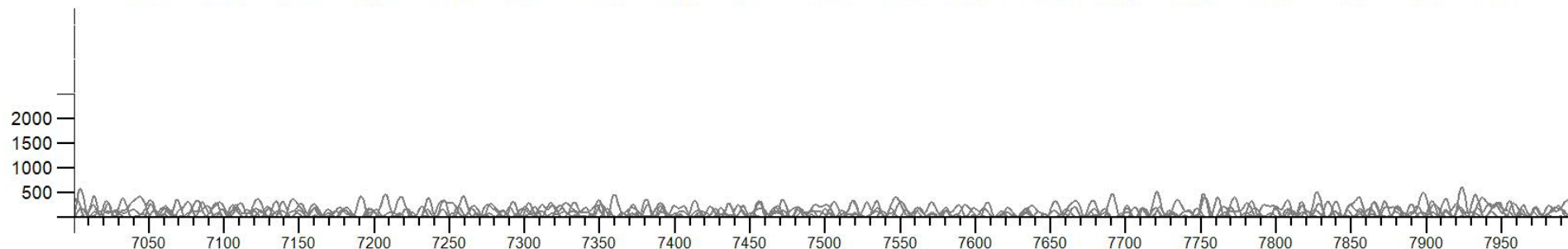

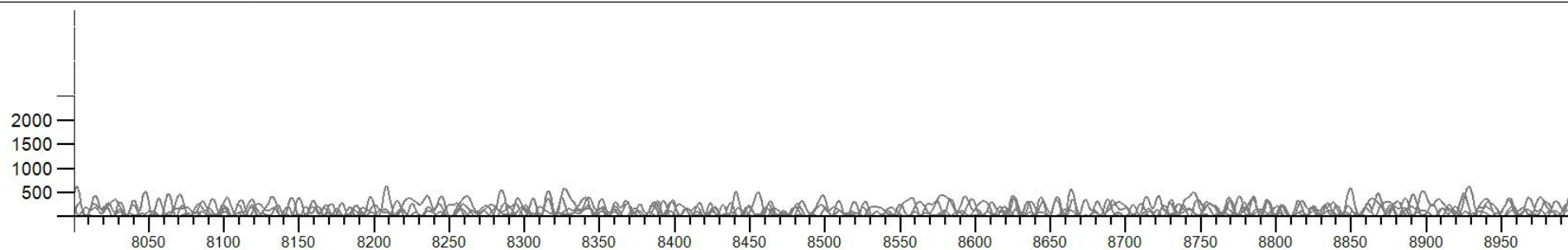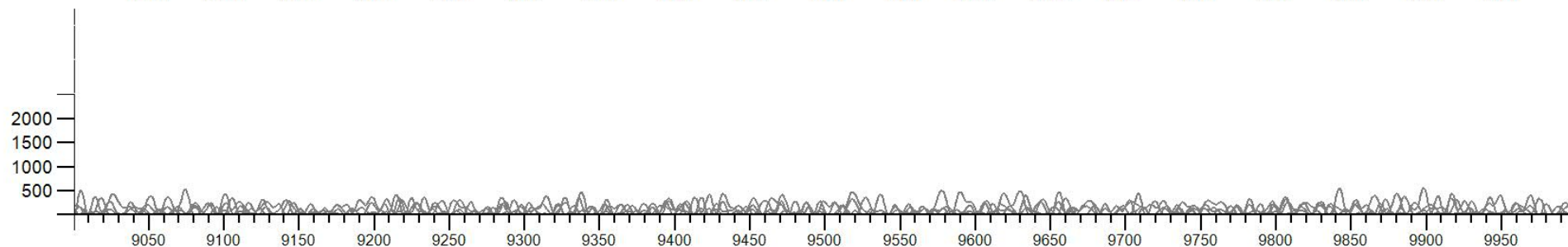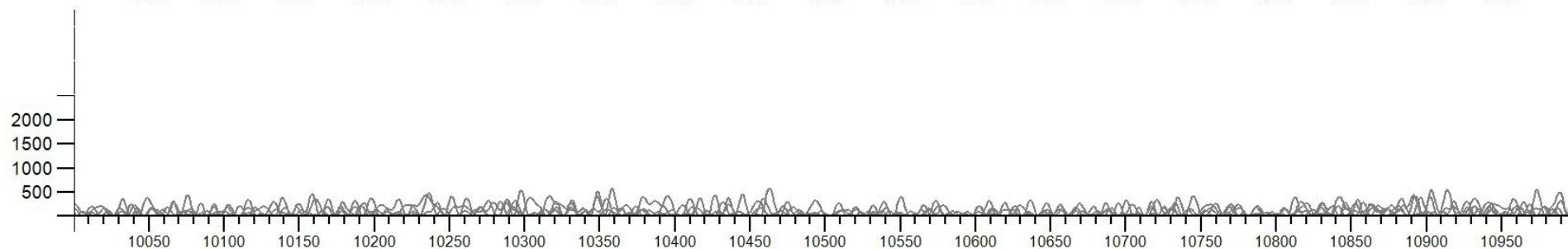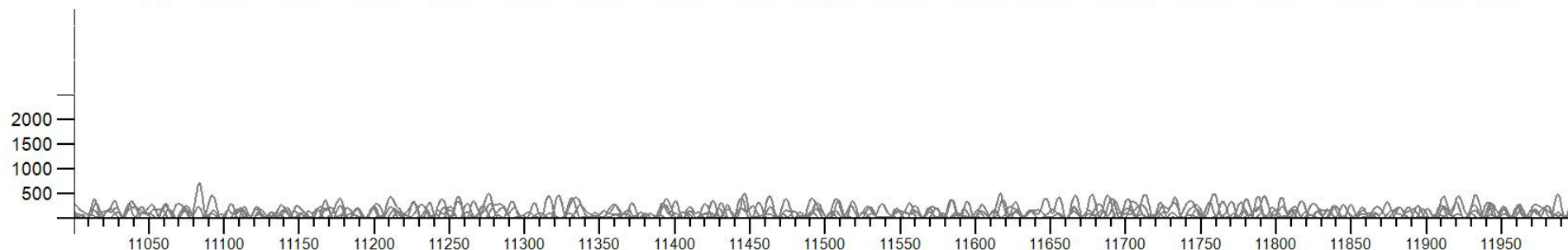

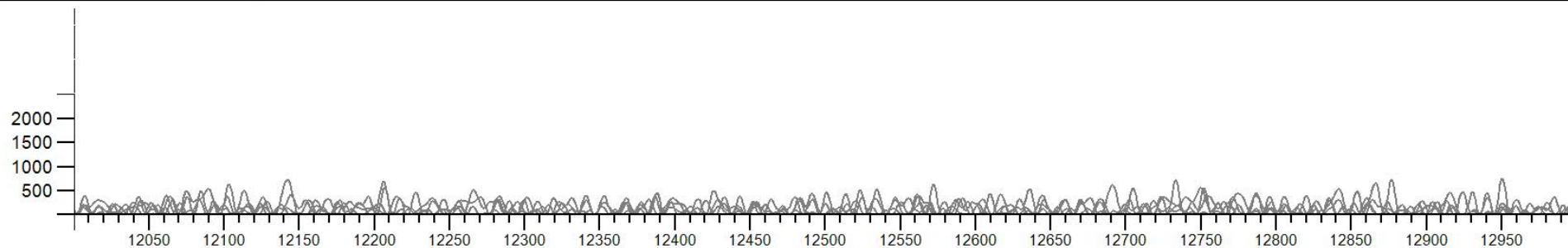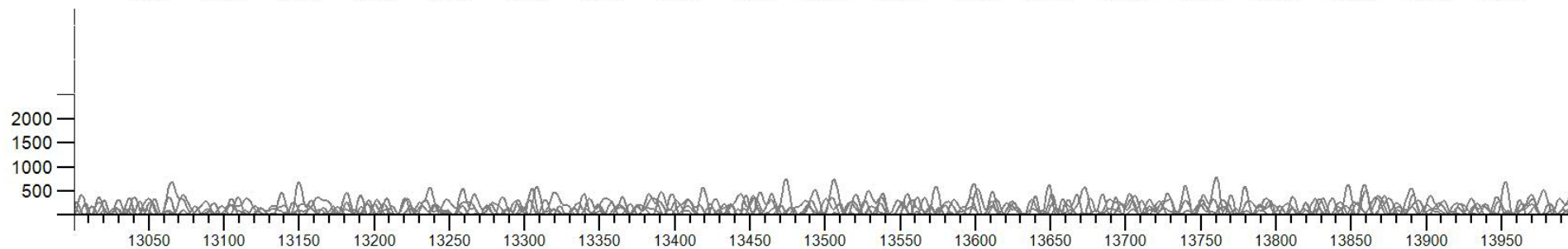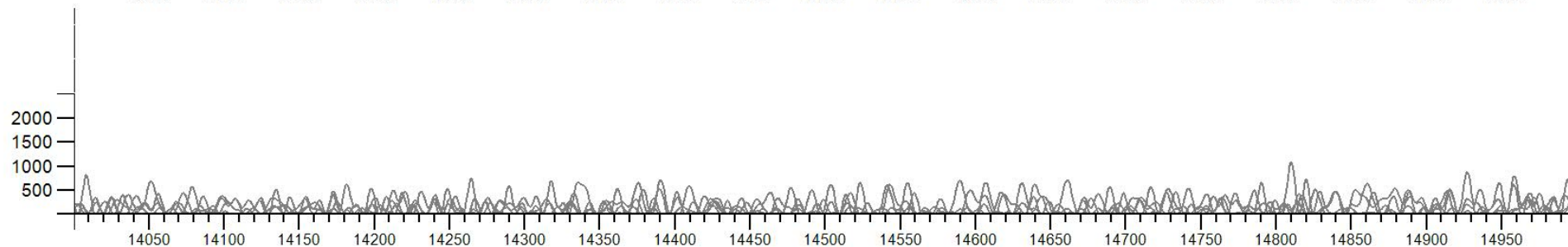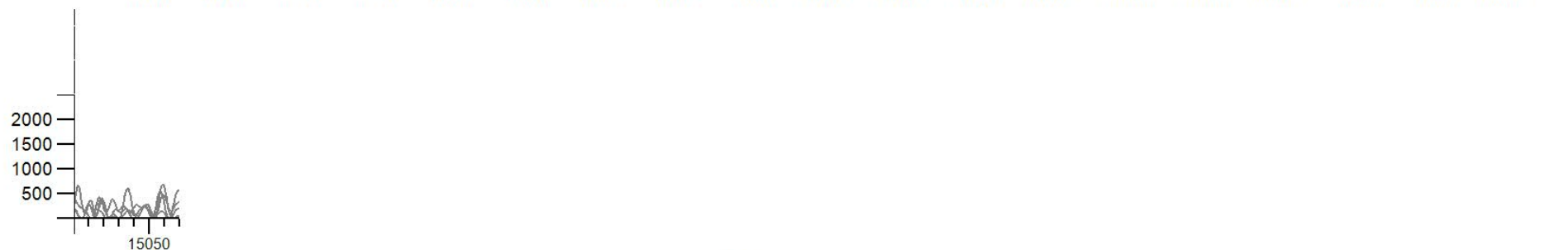

Supplement: Figure 3—source data 1. [file elife-69916-fig3-data1.zip › Figure 3A_Source data2_Bisulphite sequencing data_plasmid/SD-PDT1-BSF-3.7_T7FOR-C11.pdf]

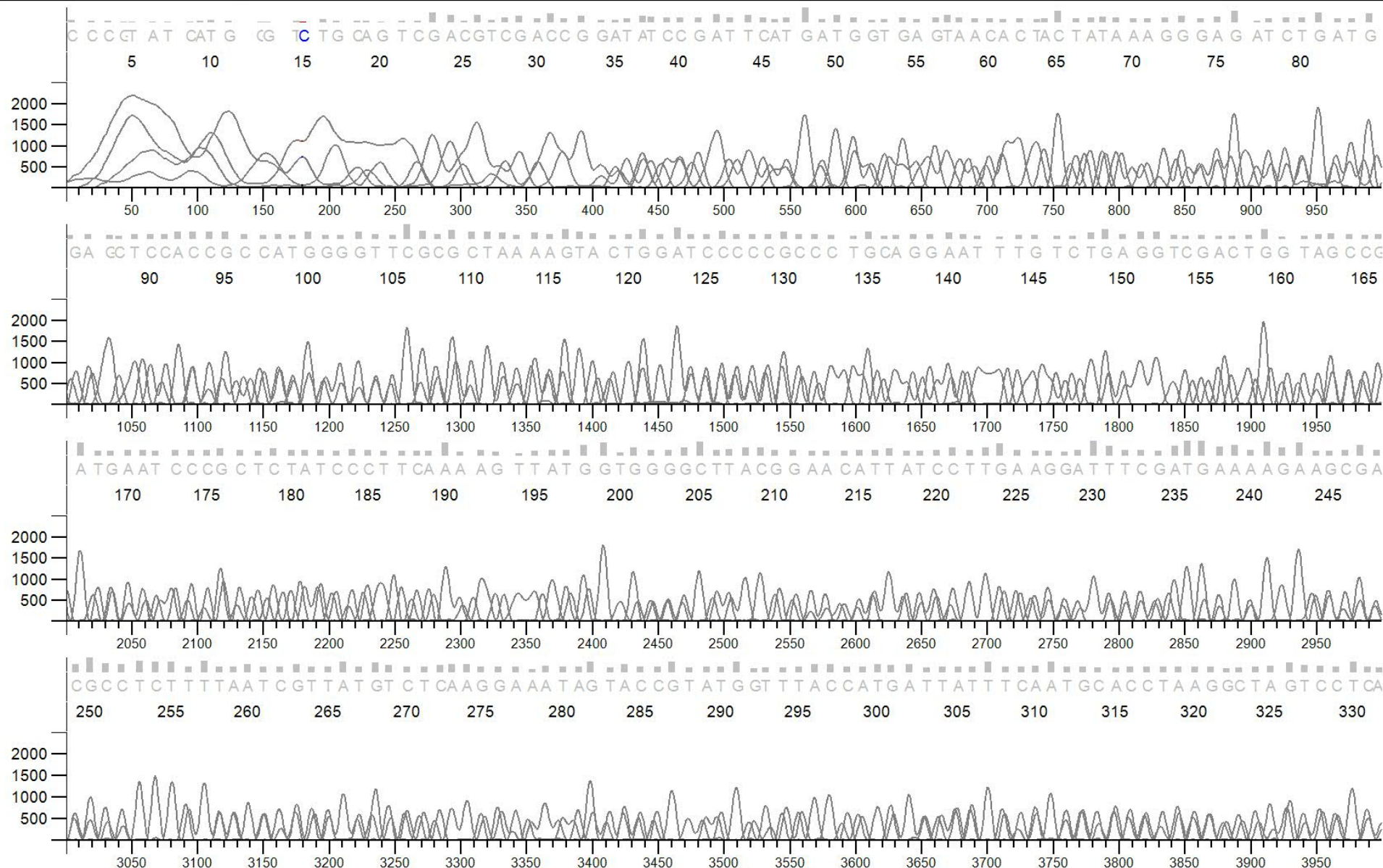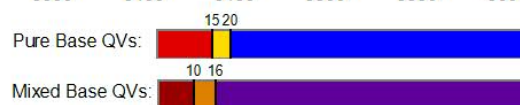

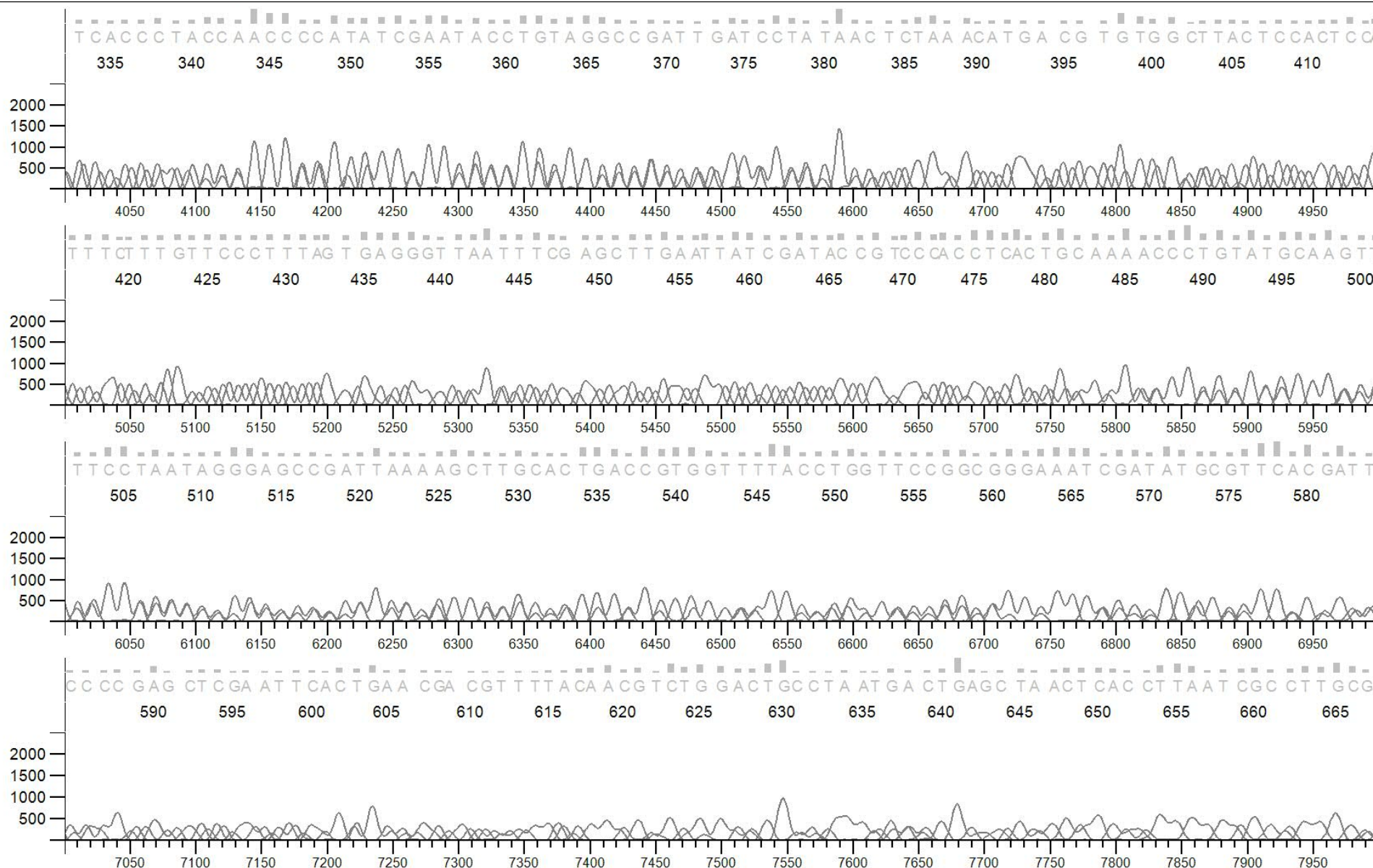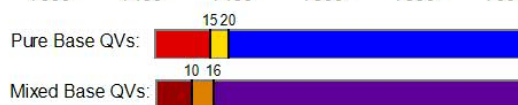

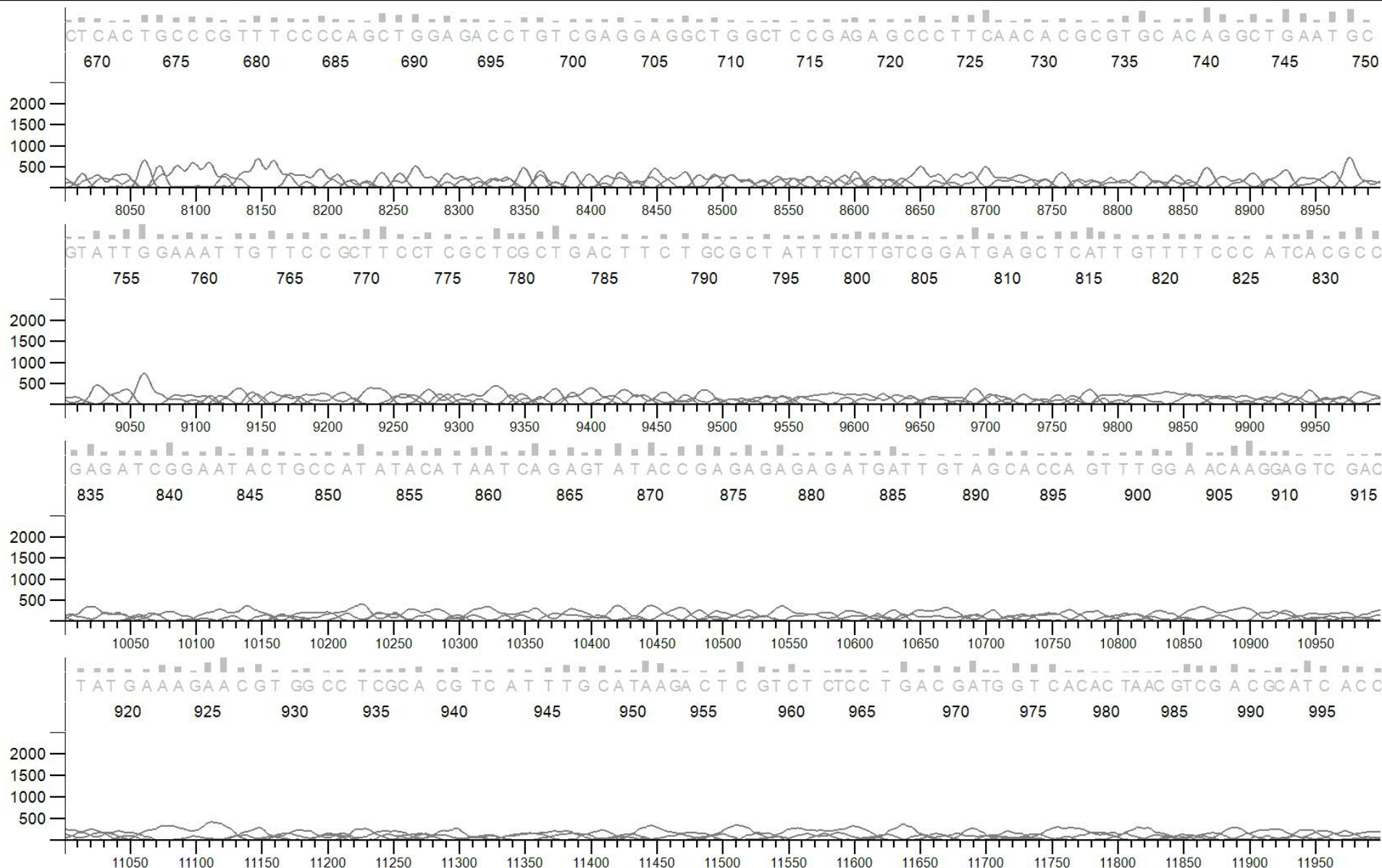

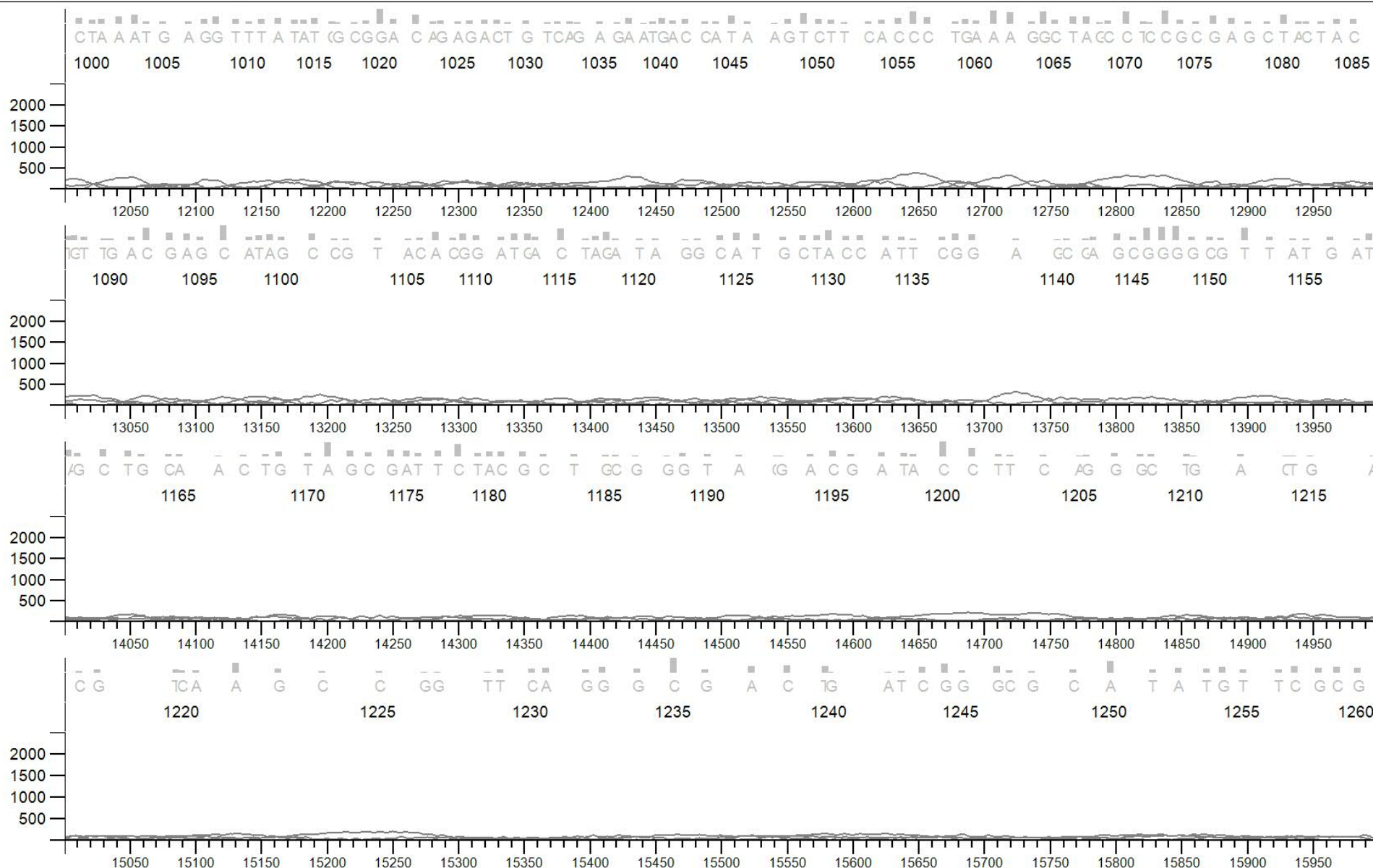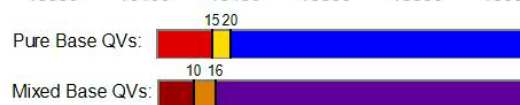

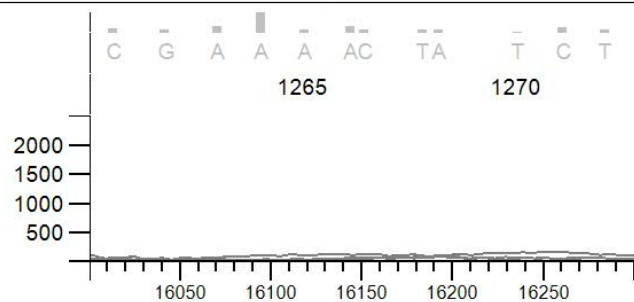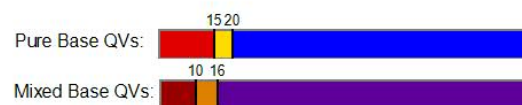

Supplement: Figure 3—source data 1. [file elife-69916-fig3-data1.zip › Figure 3A_Source data2_Bisulphite sequencing data_plasmid/SD-PDT1-BSF-3.9_T7FOR-E11.pdf]

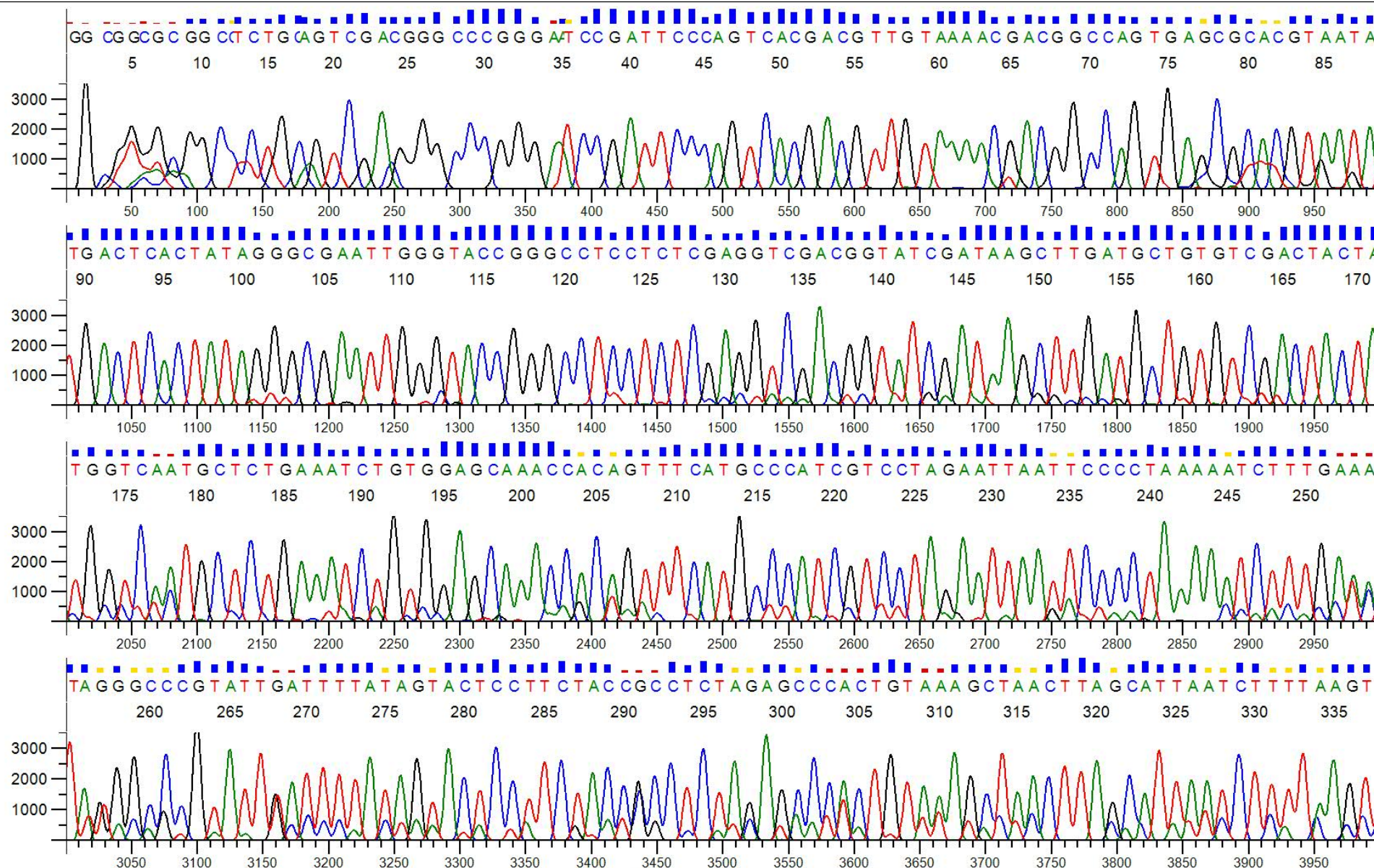

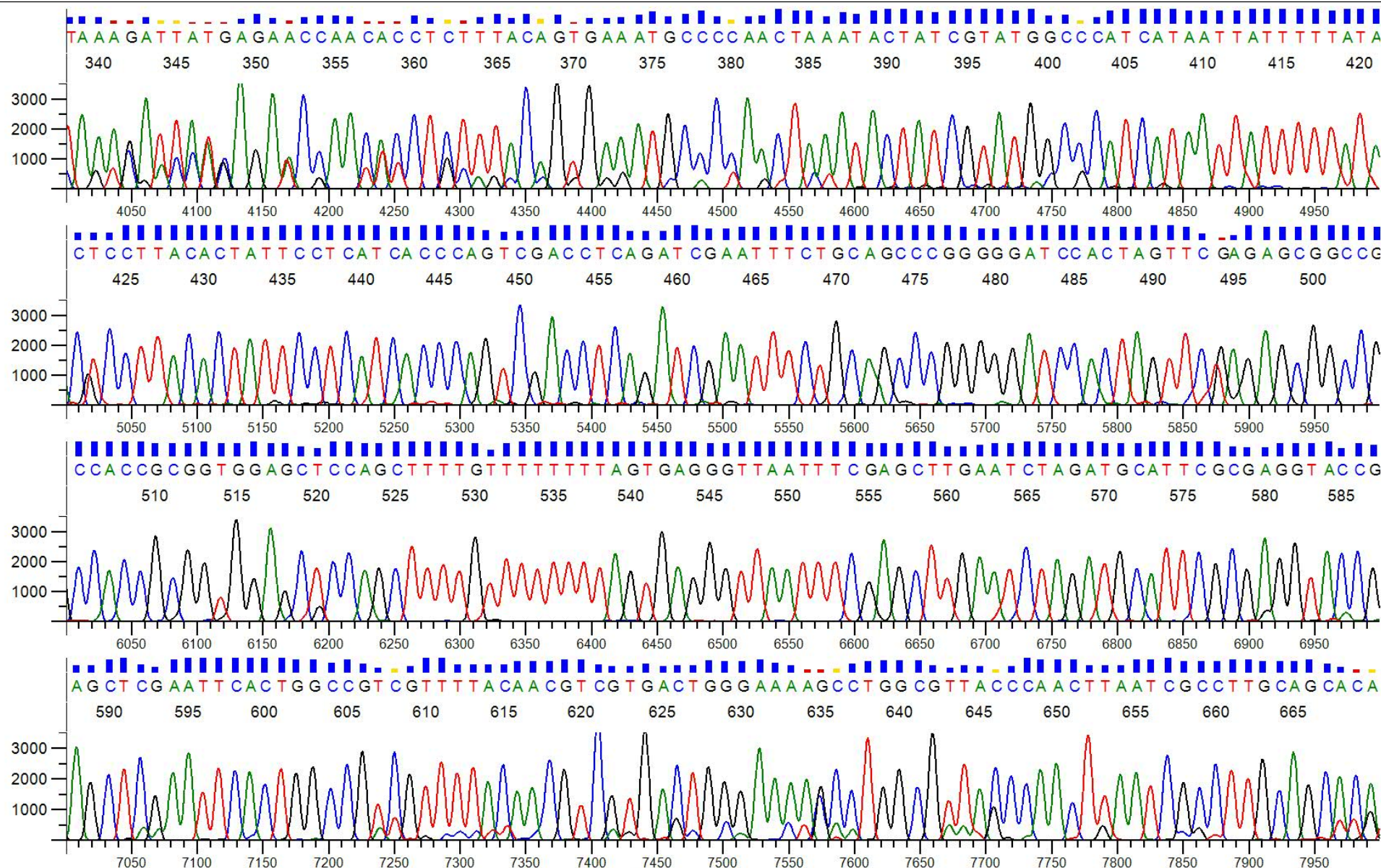

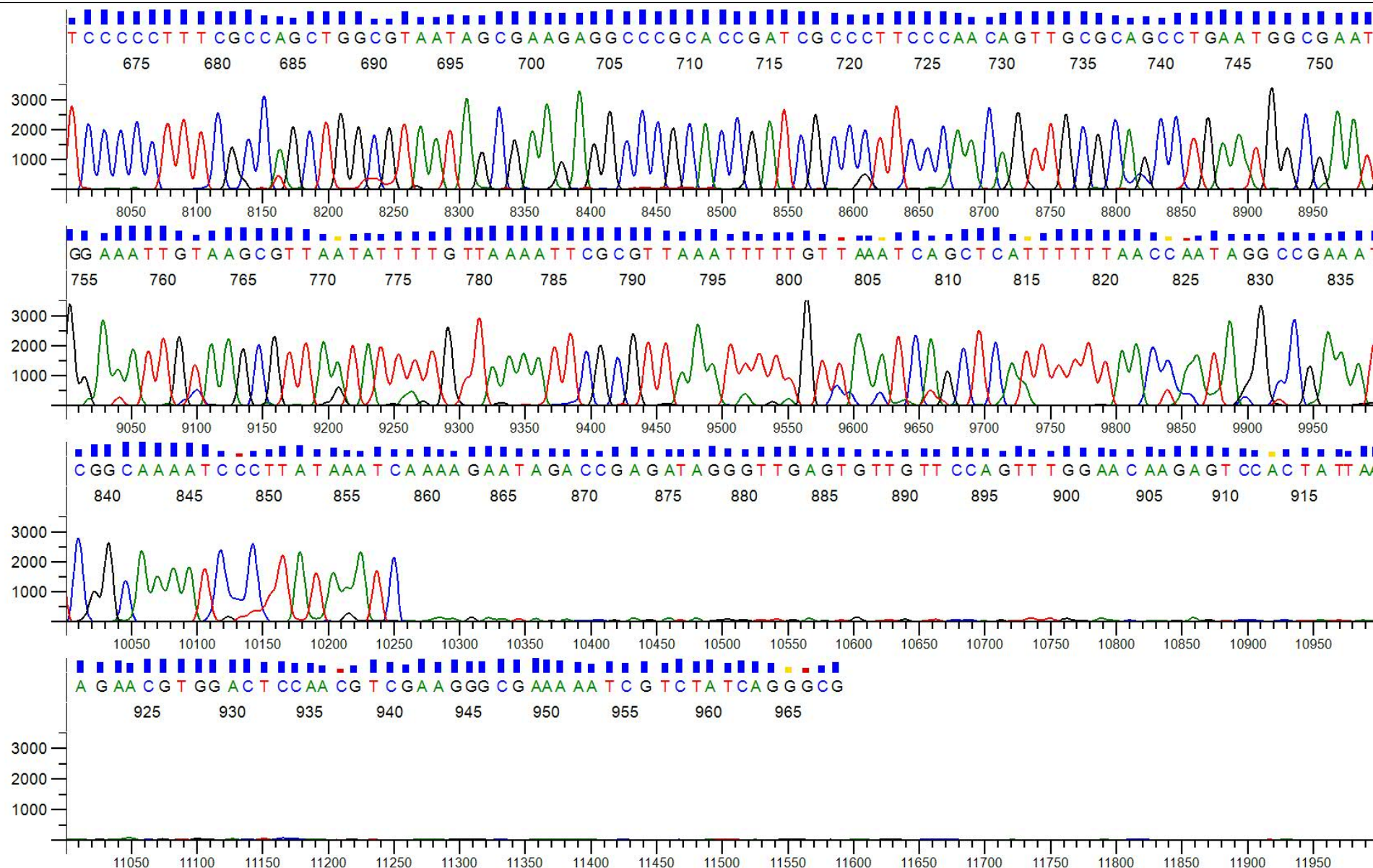

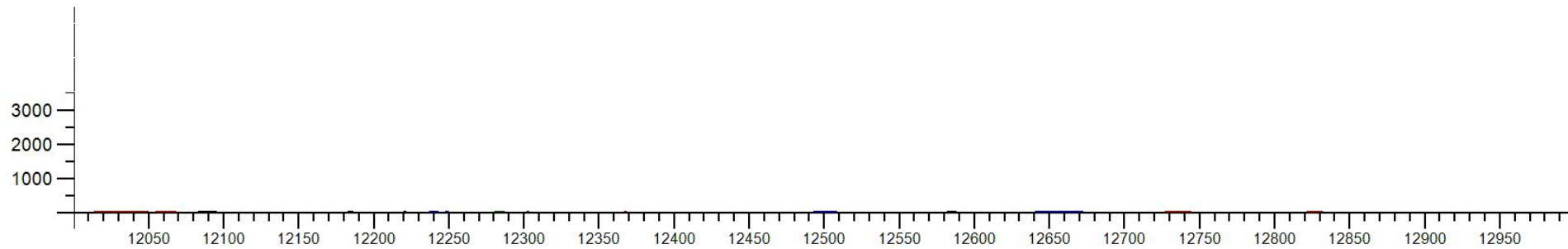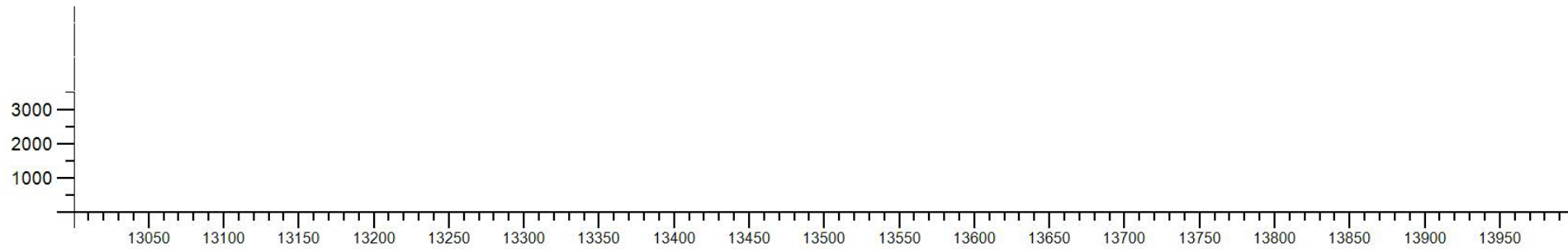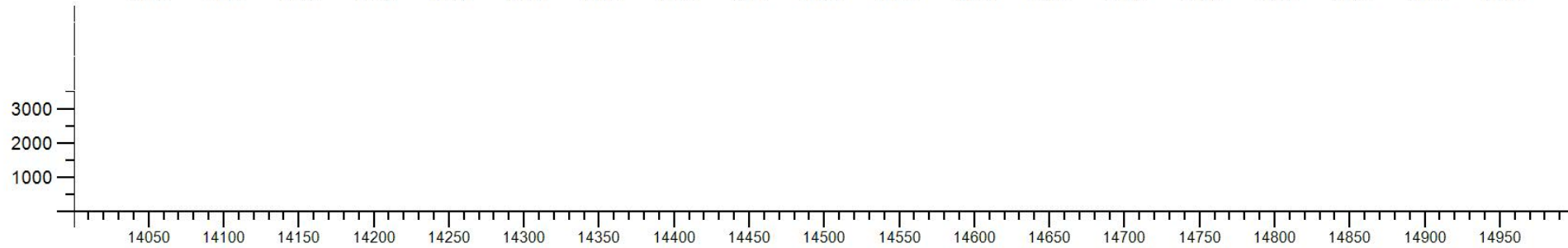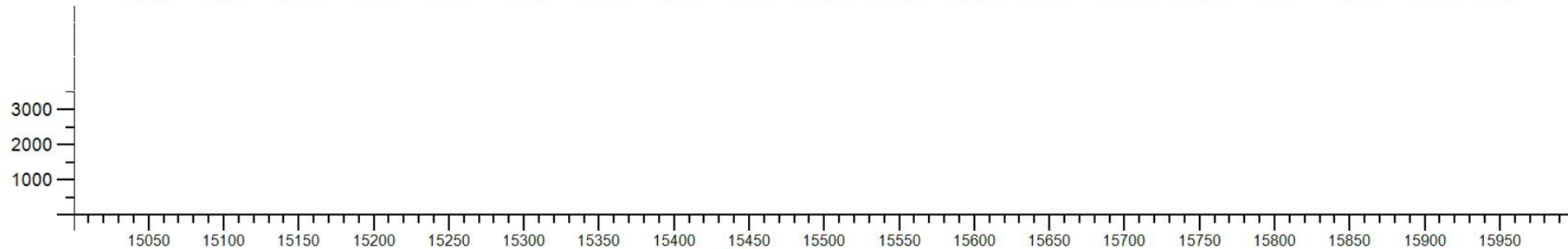

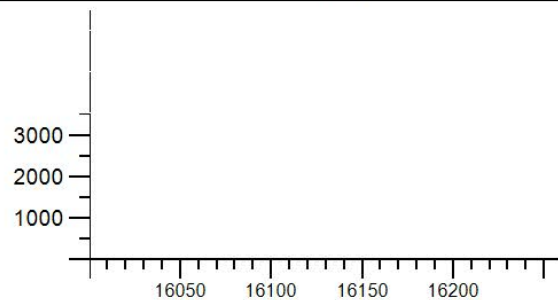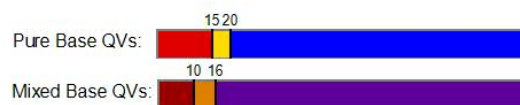

Supplement: Figure 3—source data 1. [file elife-69916-fig3-data1.zip › Figure 3A_Source data2_Bisulphite sequencing data_plasmid/SD-PDI1-BSF-2.1_T7-FOR-C01.pdf]

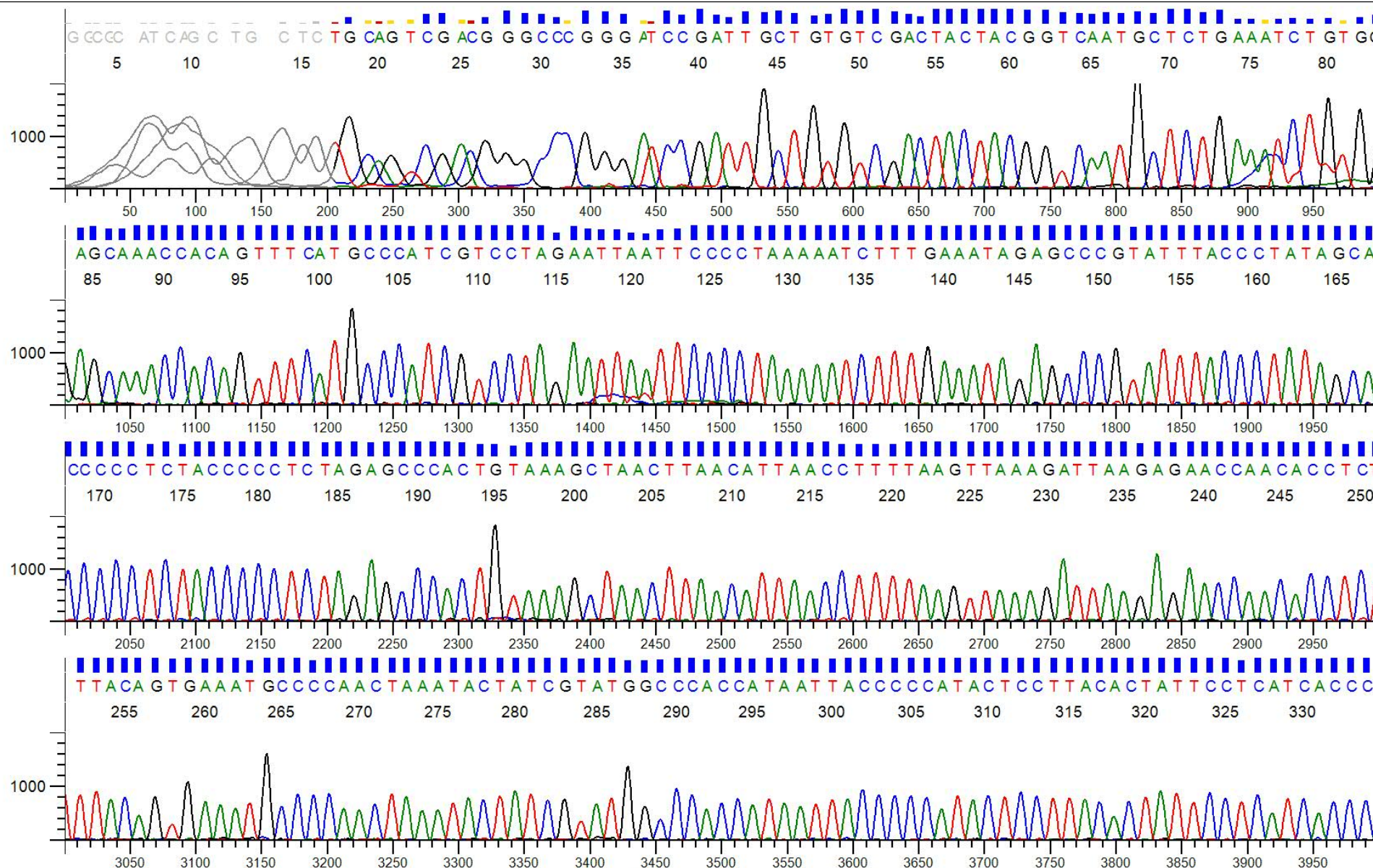

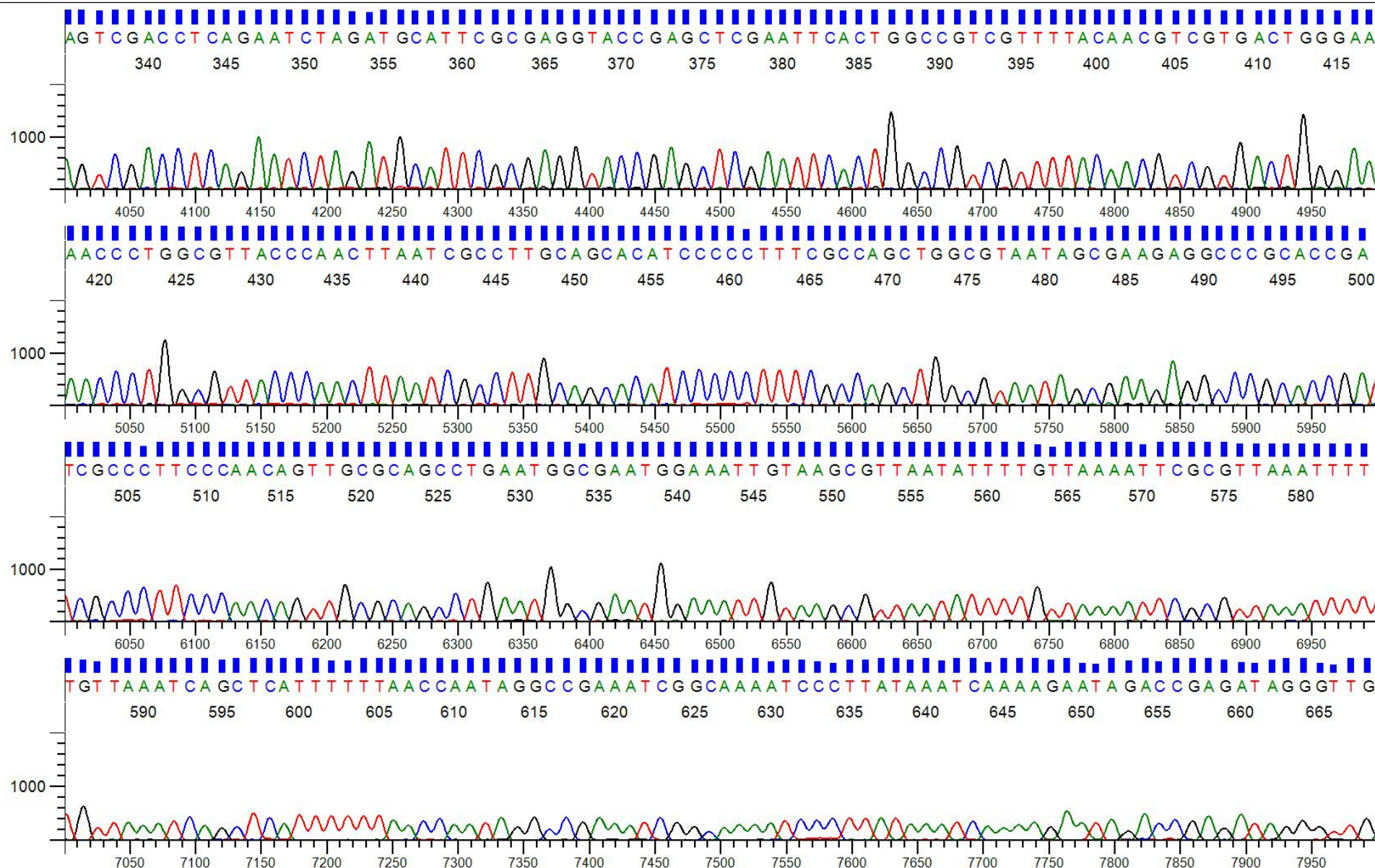

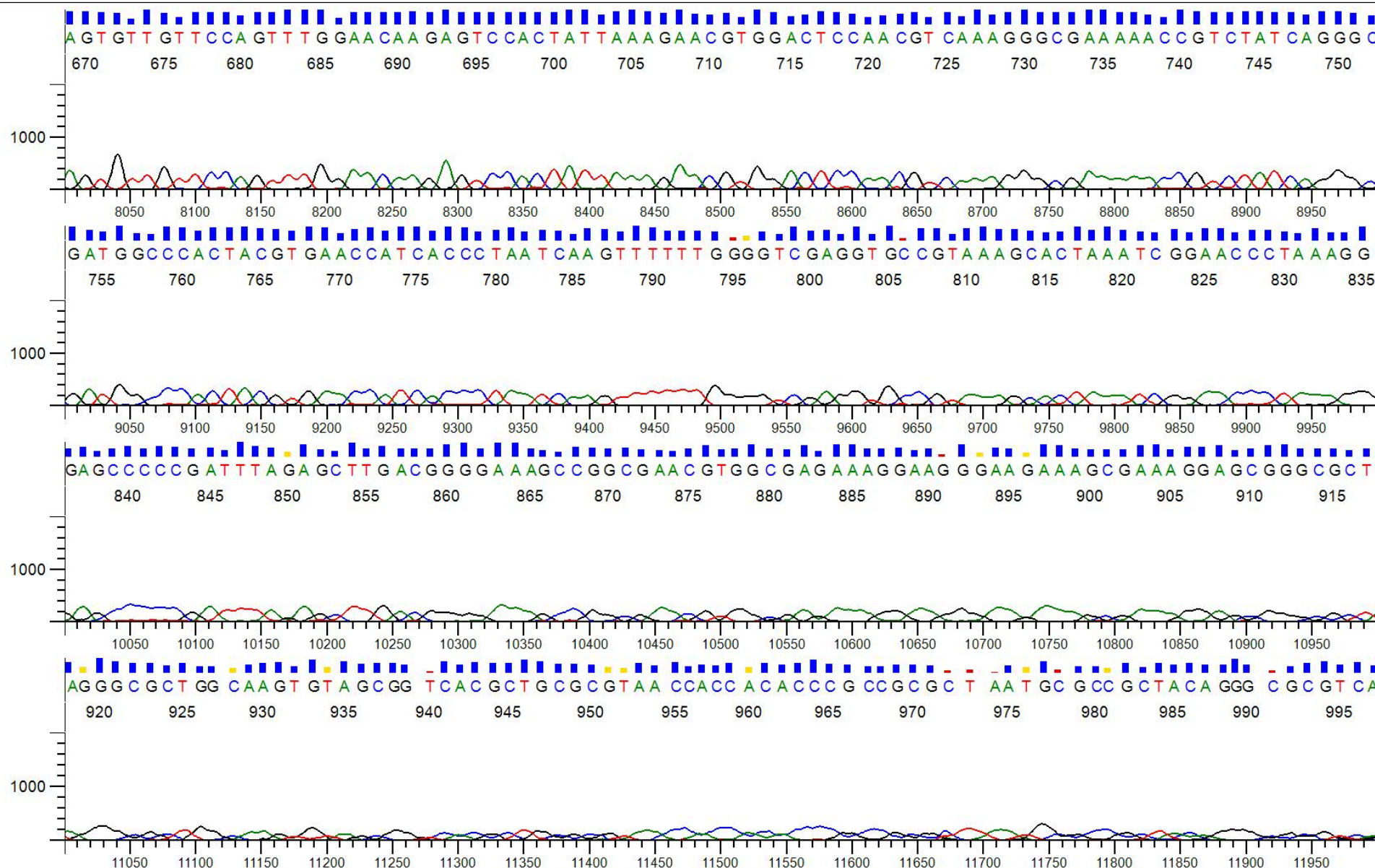

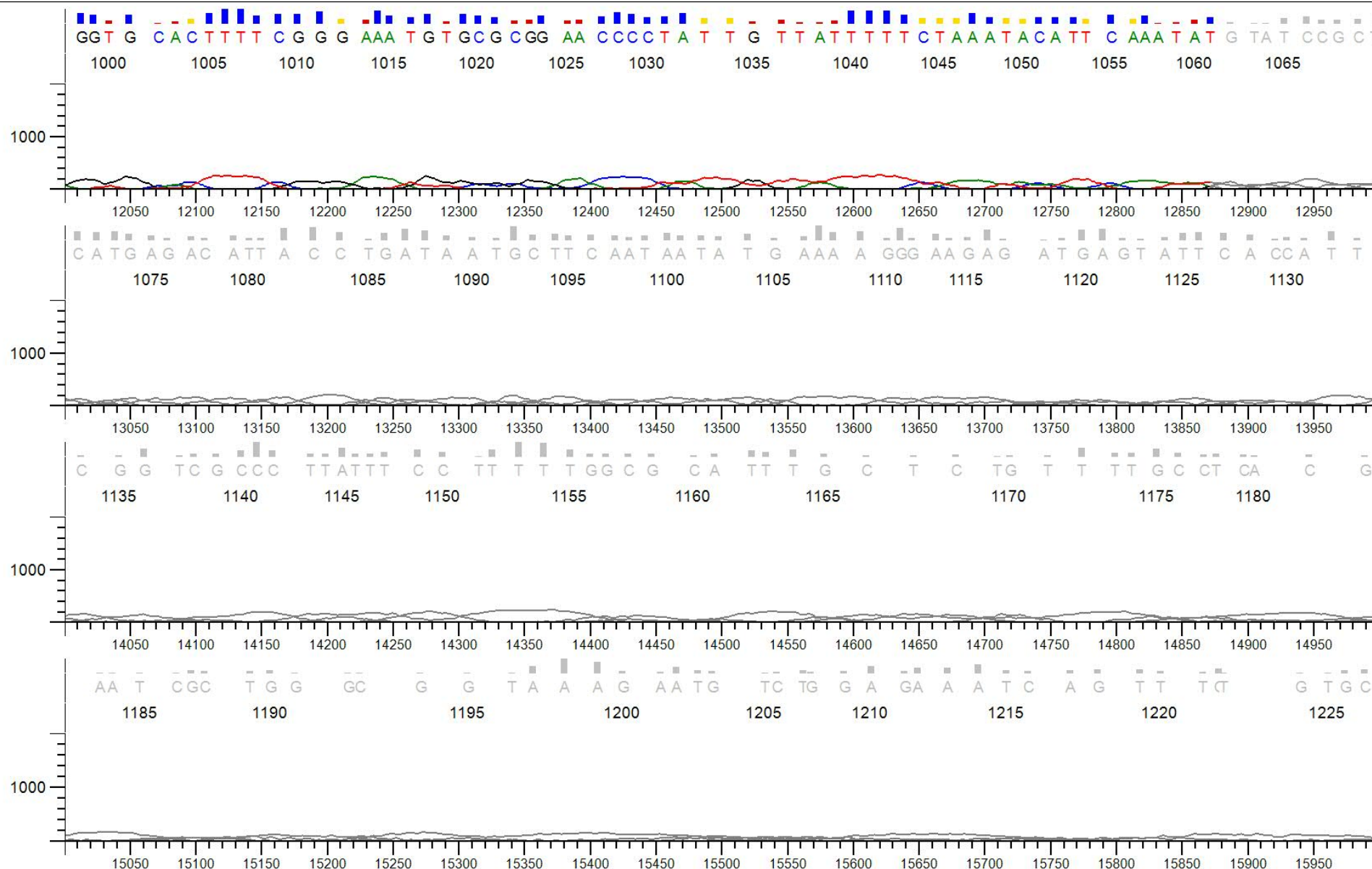

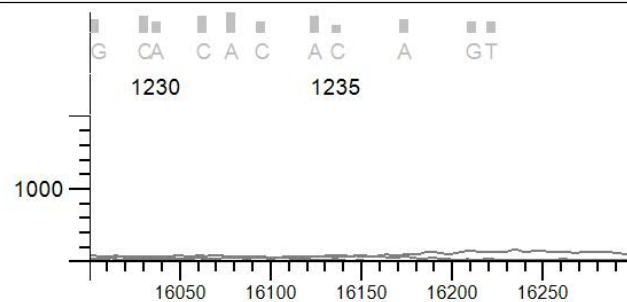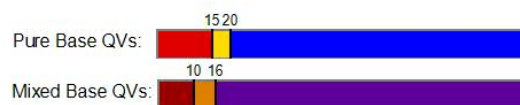

Supplement: Figure 3—source data 2. [file elife-69916-fig3-data2.zip › Figure 3B.C_Source data3_Bisulphite sequencing_mtDNA/SS4-MT-BIS-24_T7FOR-D06.pdf]

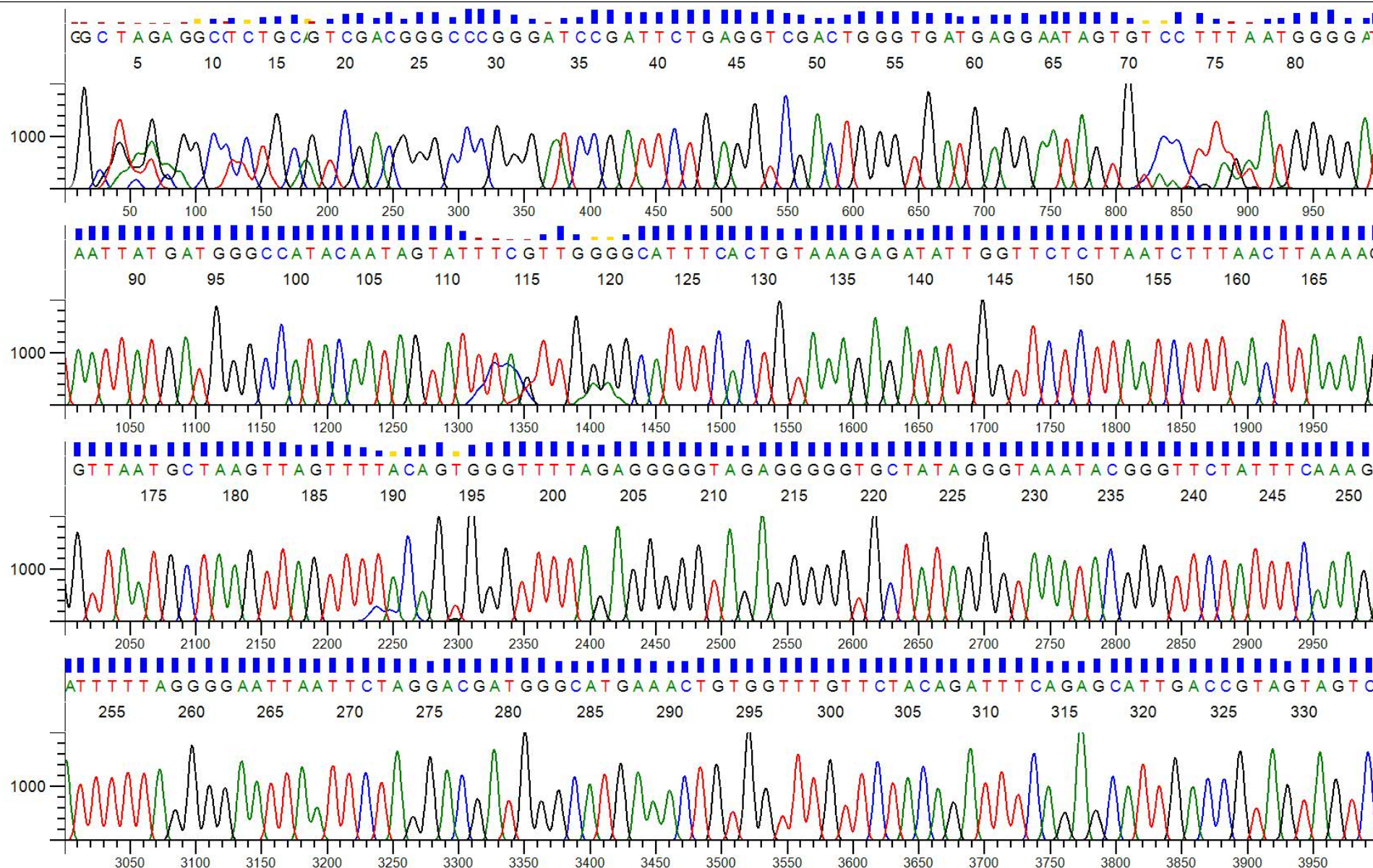

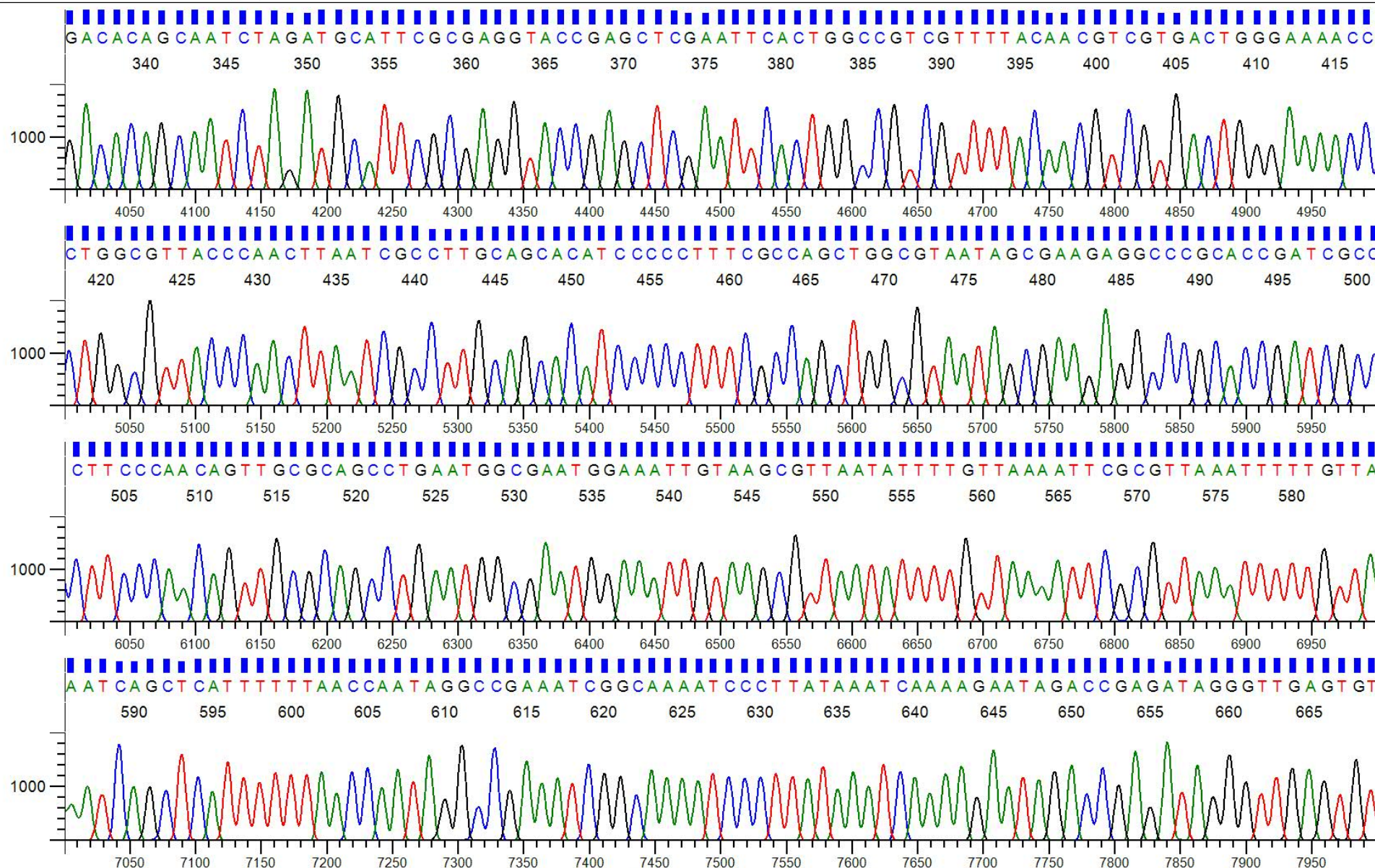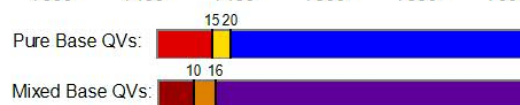

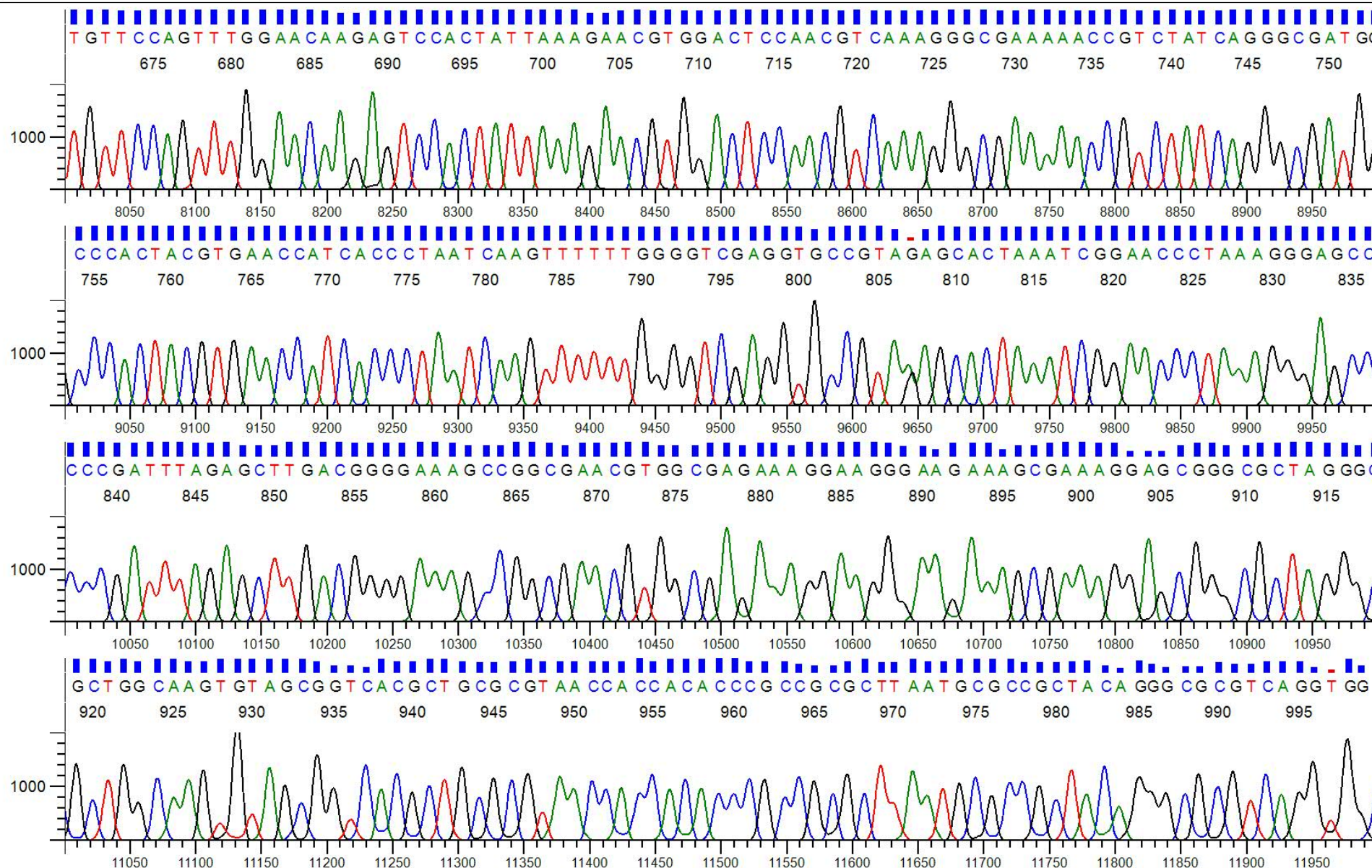

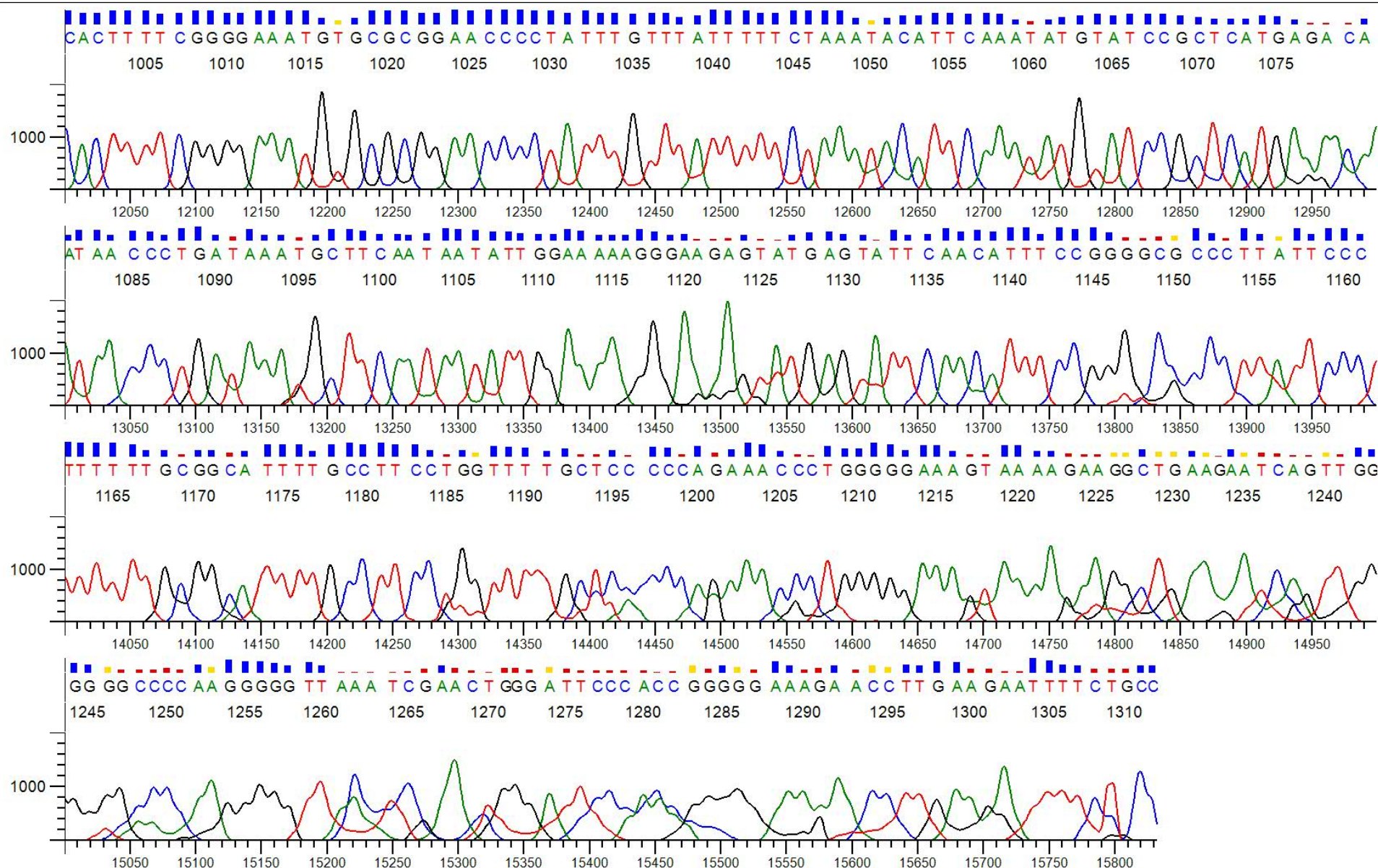

Supplement: Figure 3—source data 2. [file elife-69916-fig3-data2.zip › Figure 3B.C_Source data3_Bisulphite sequencing_mtDNA/SS4-MT-BIS-39_T7FOR-C07.pdf]

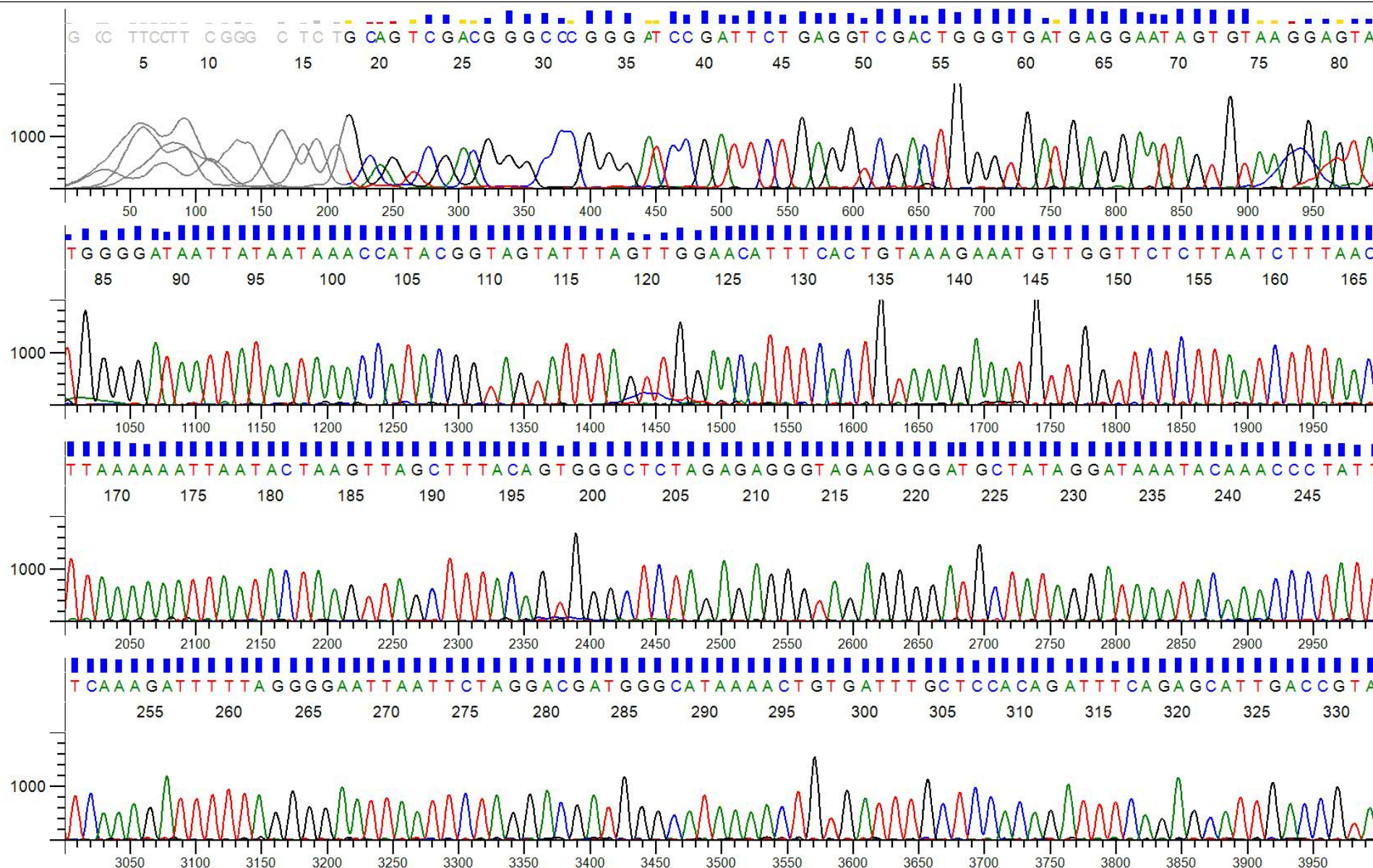

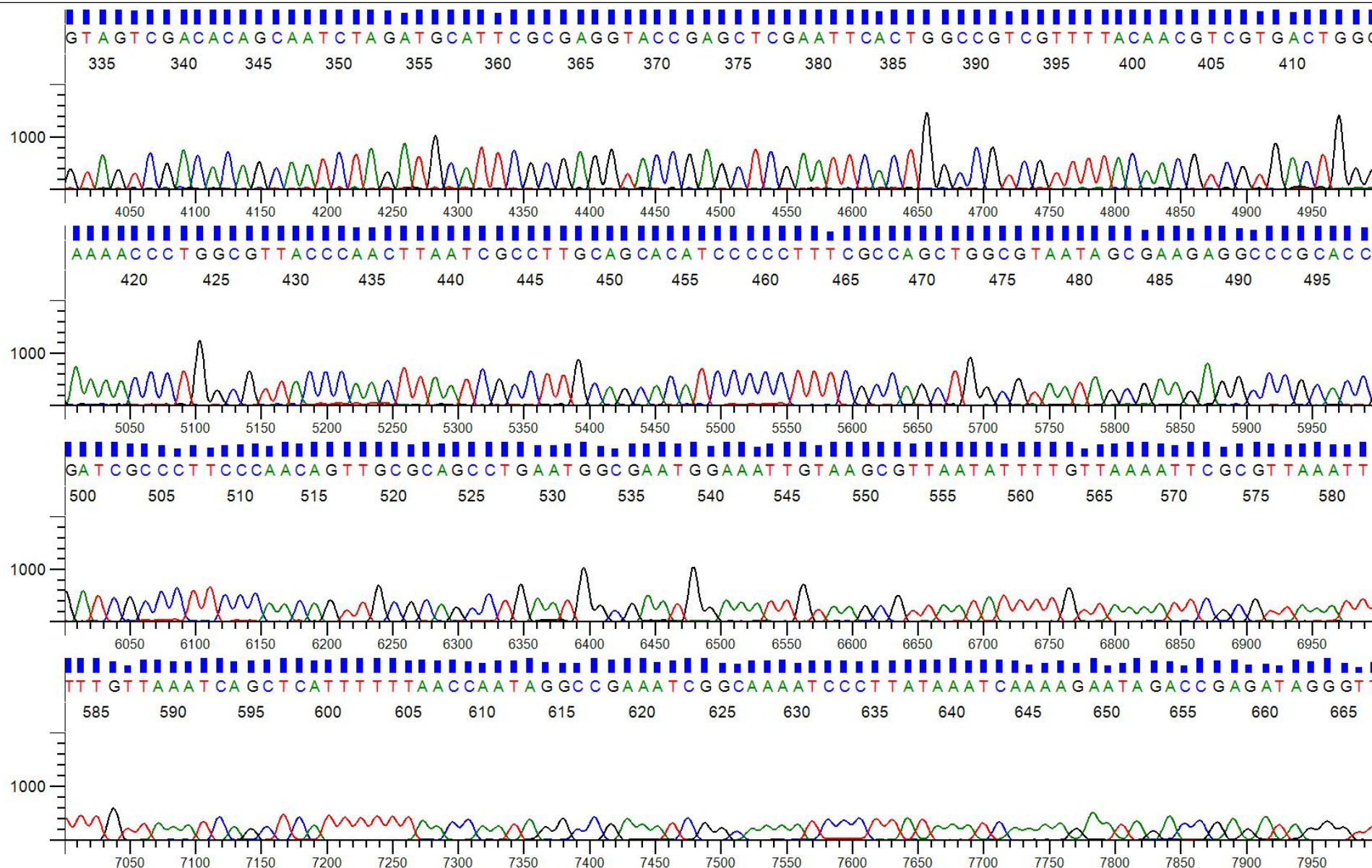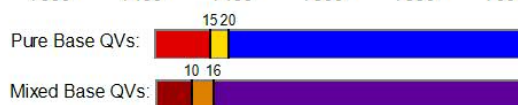

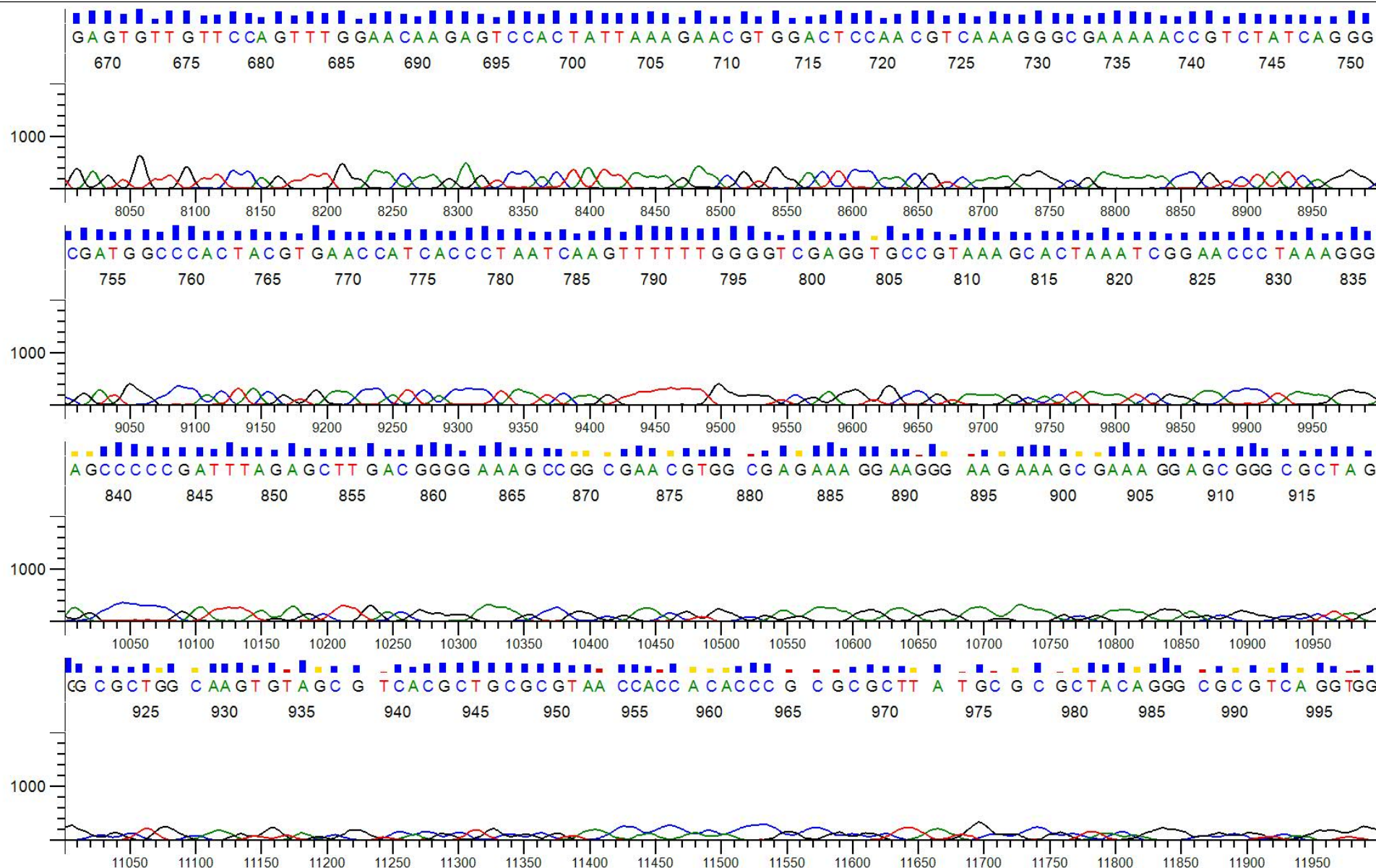

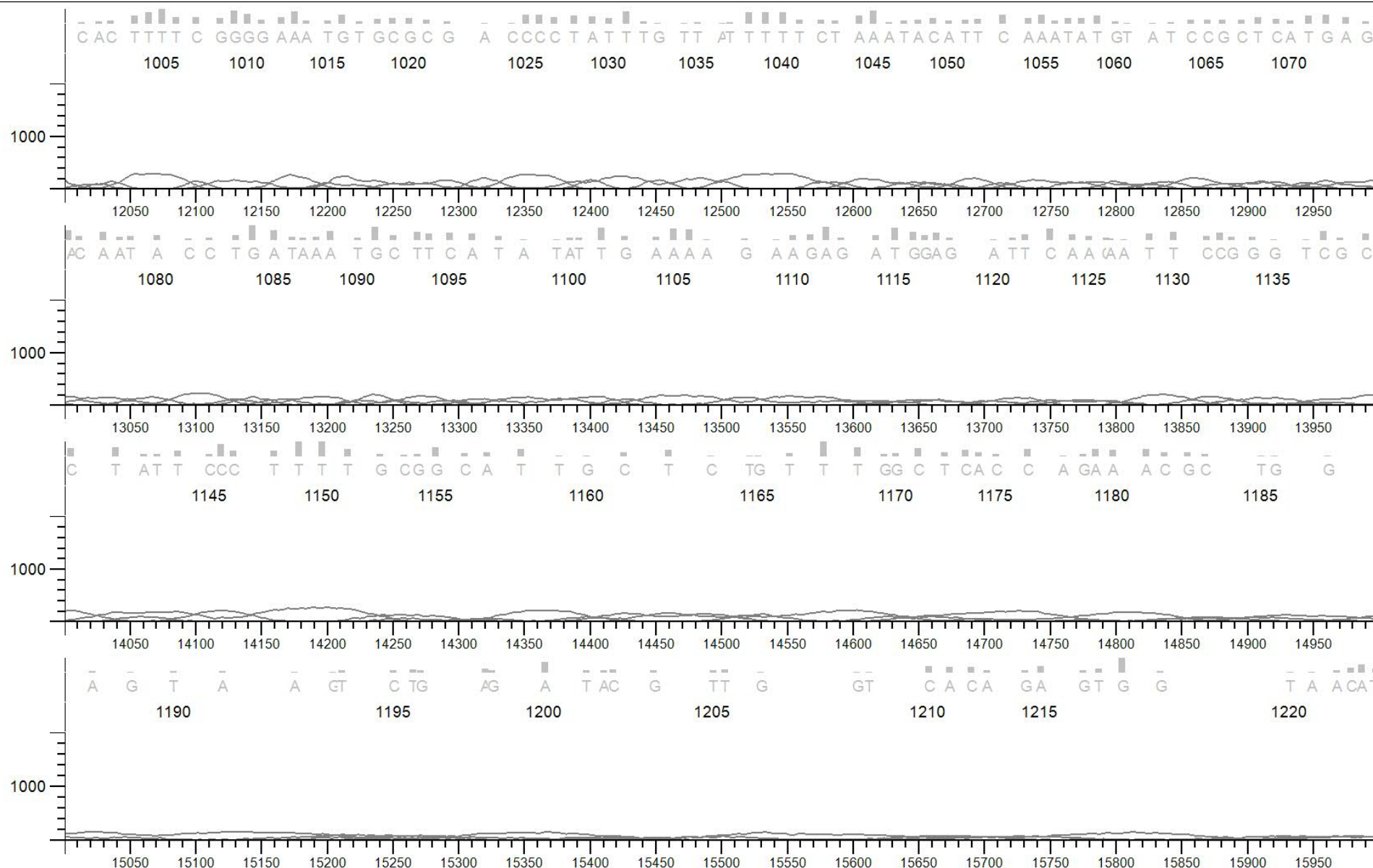

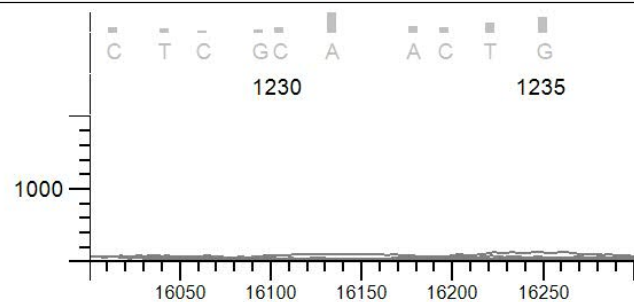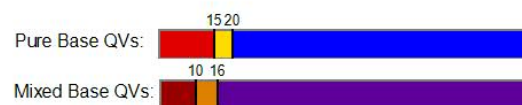

Supplement: Figure 3—source data 2. [file elife-69916-fig3-data2.zip › Figure 3B.C_Source data3_Bisulphite sequencing_mtDNA/SS4-MT-BIS-29_T7FOR-G06.pdf]

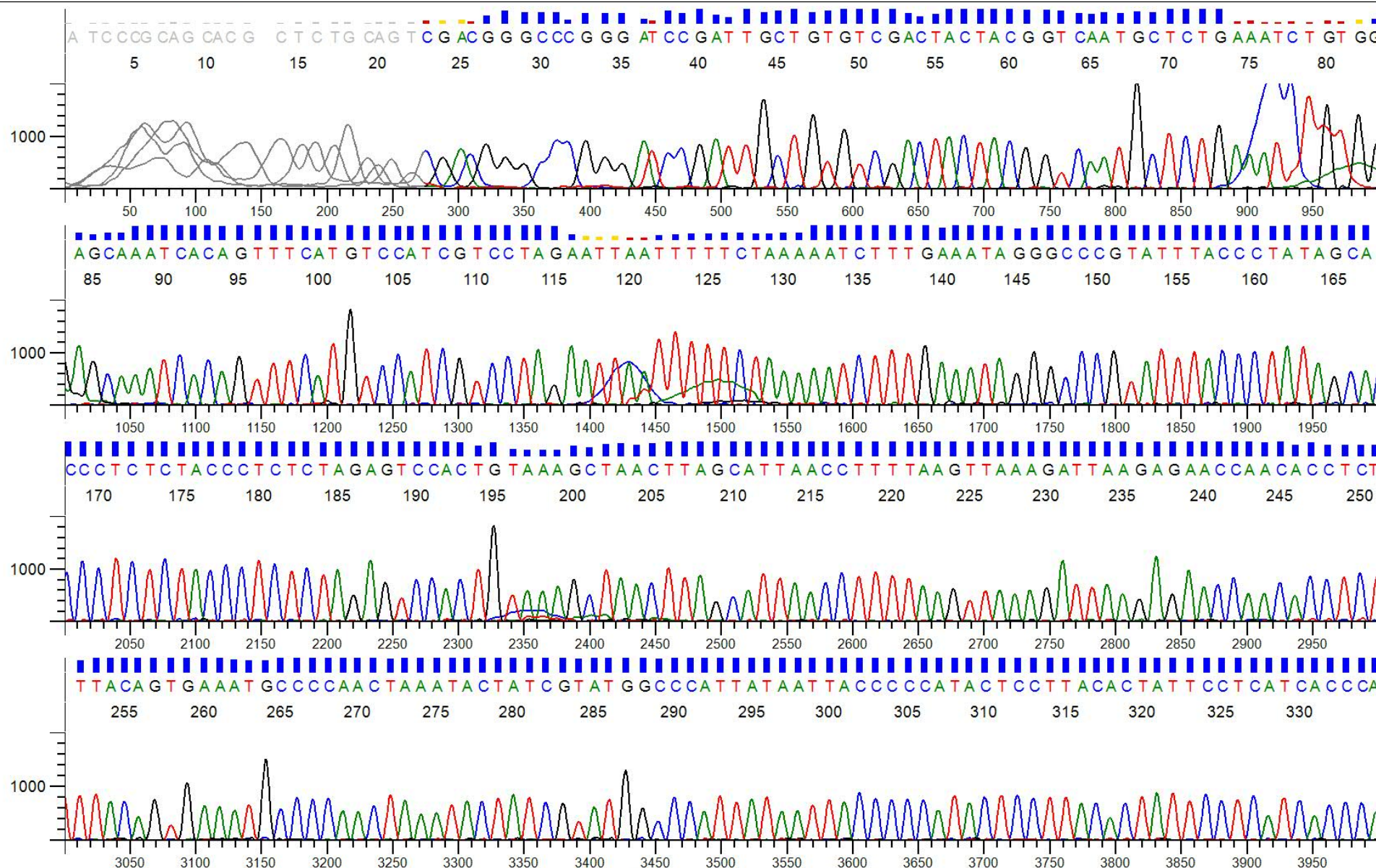

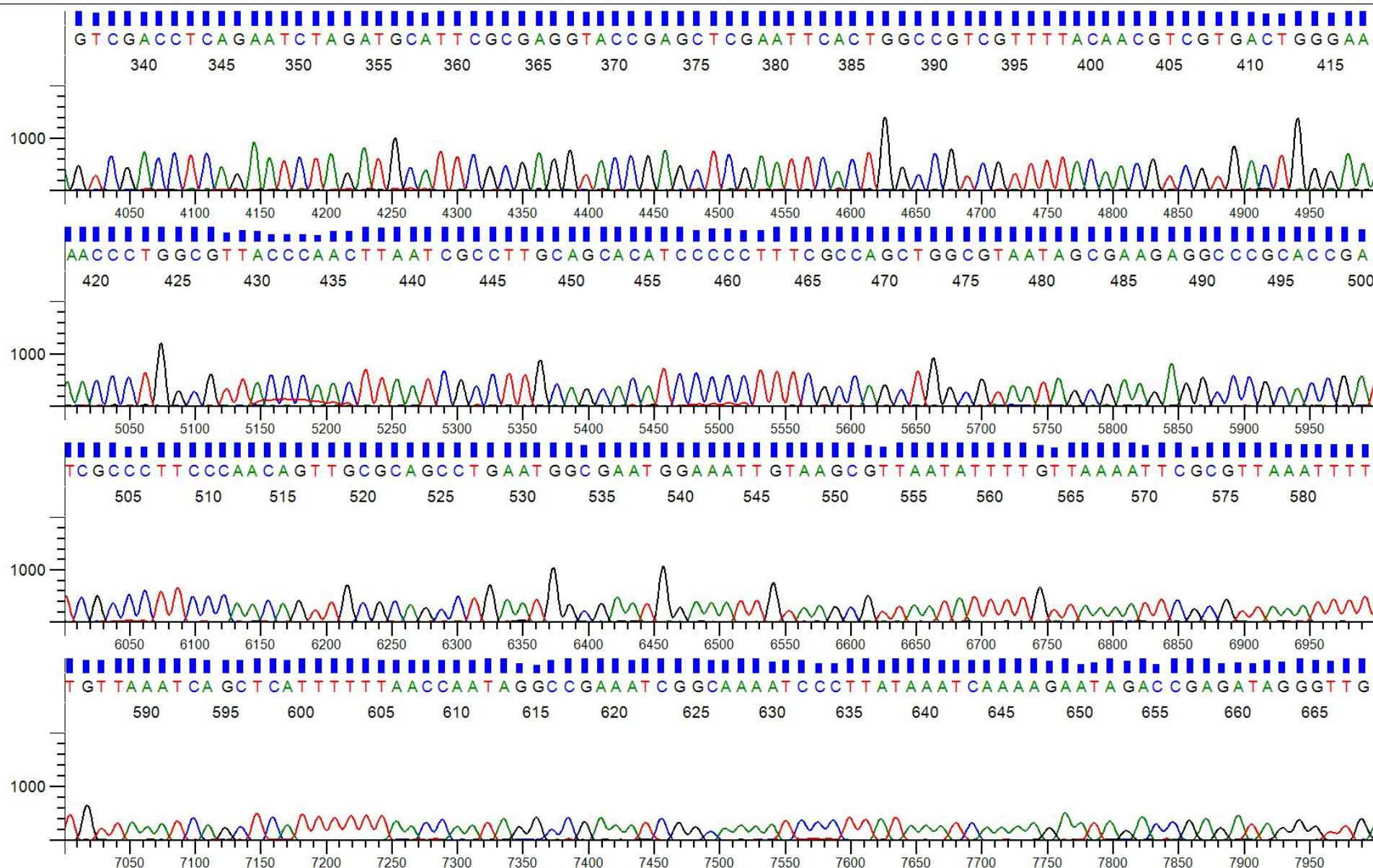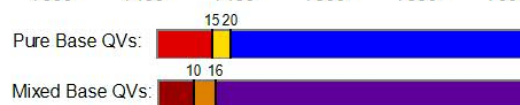

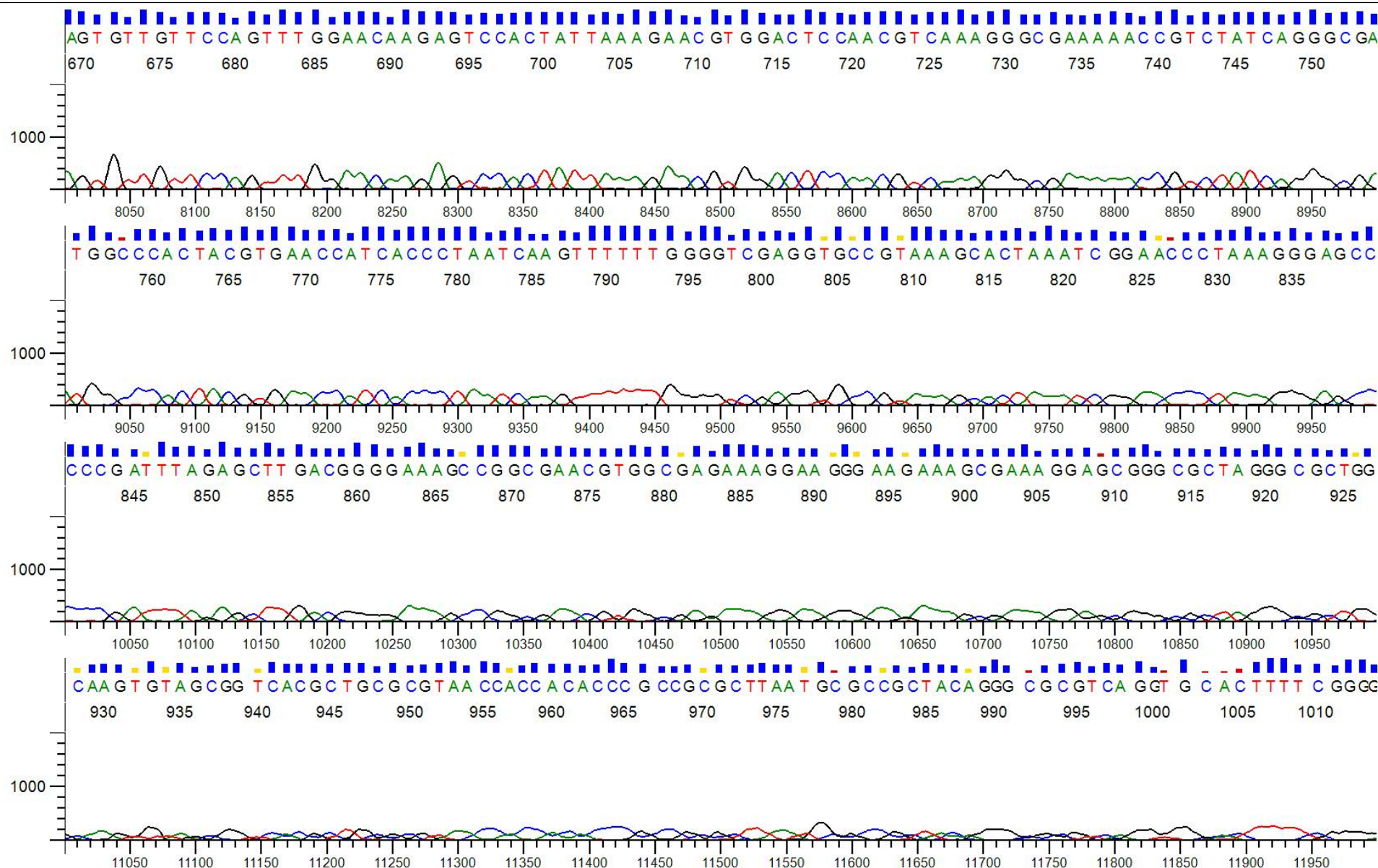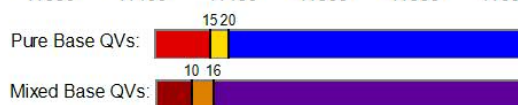

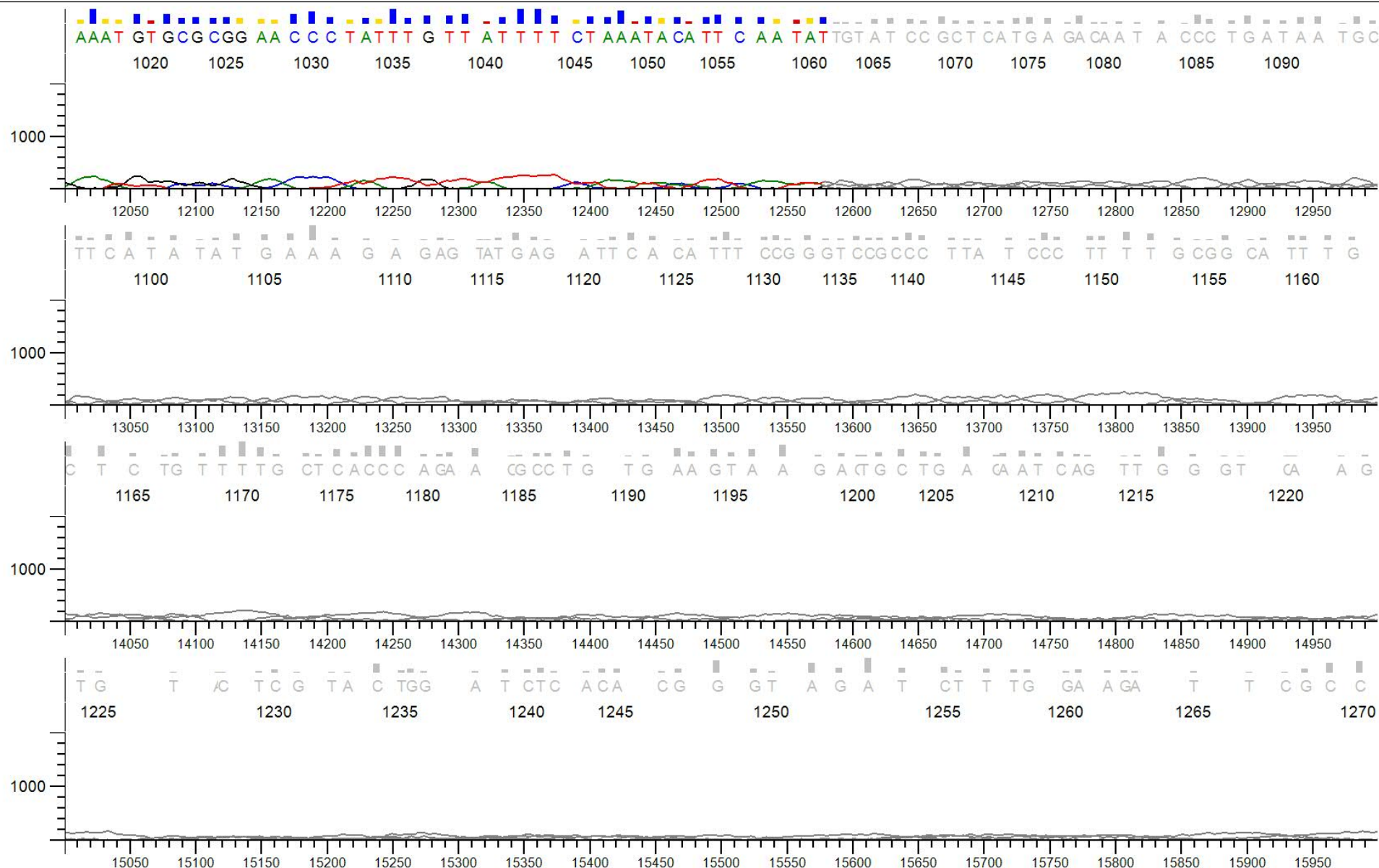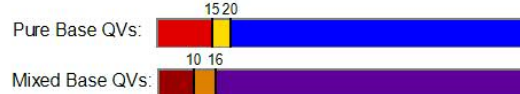

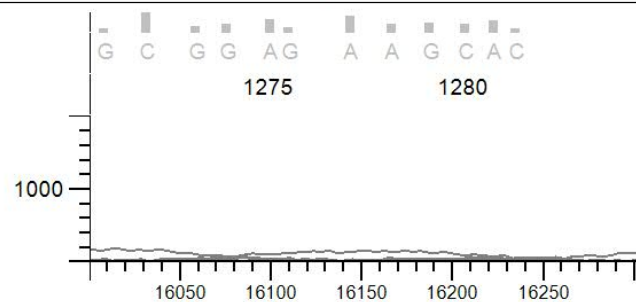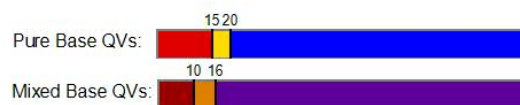

Supplement: Figure 3—source data 2. [file elife-69916-fig3-data2.zip › Figure 3B.C_Source data3_Bisulphite sequencing_mtDNA/SS4-MT-BIS-2_T7FOR-G06.pdf]

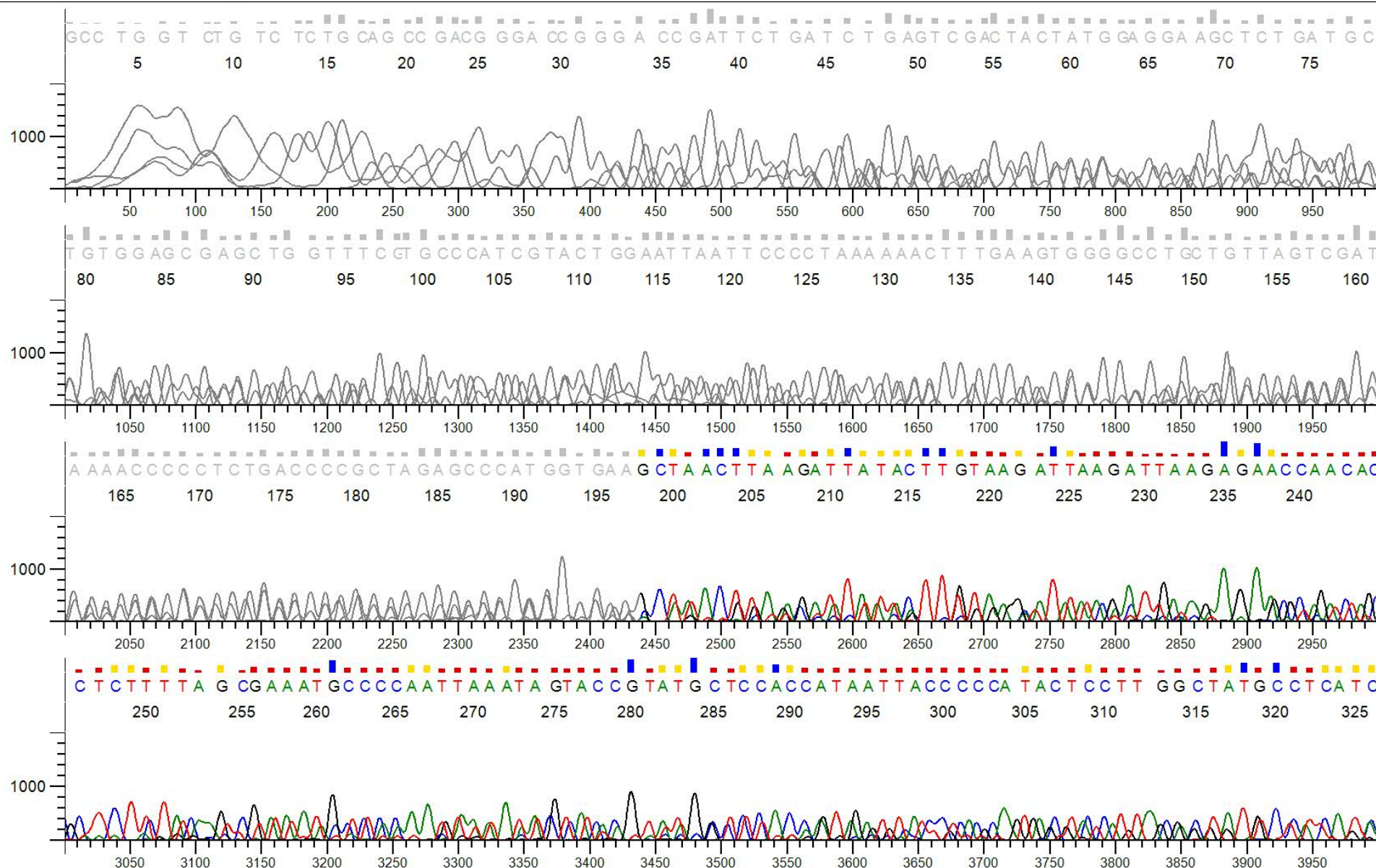

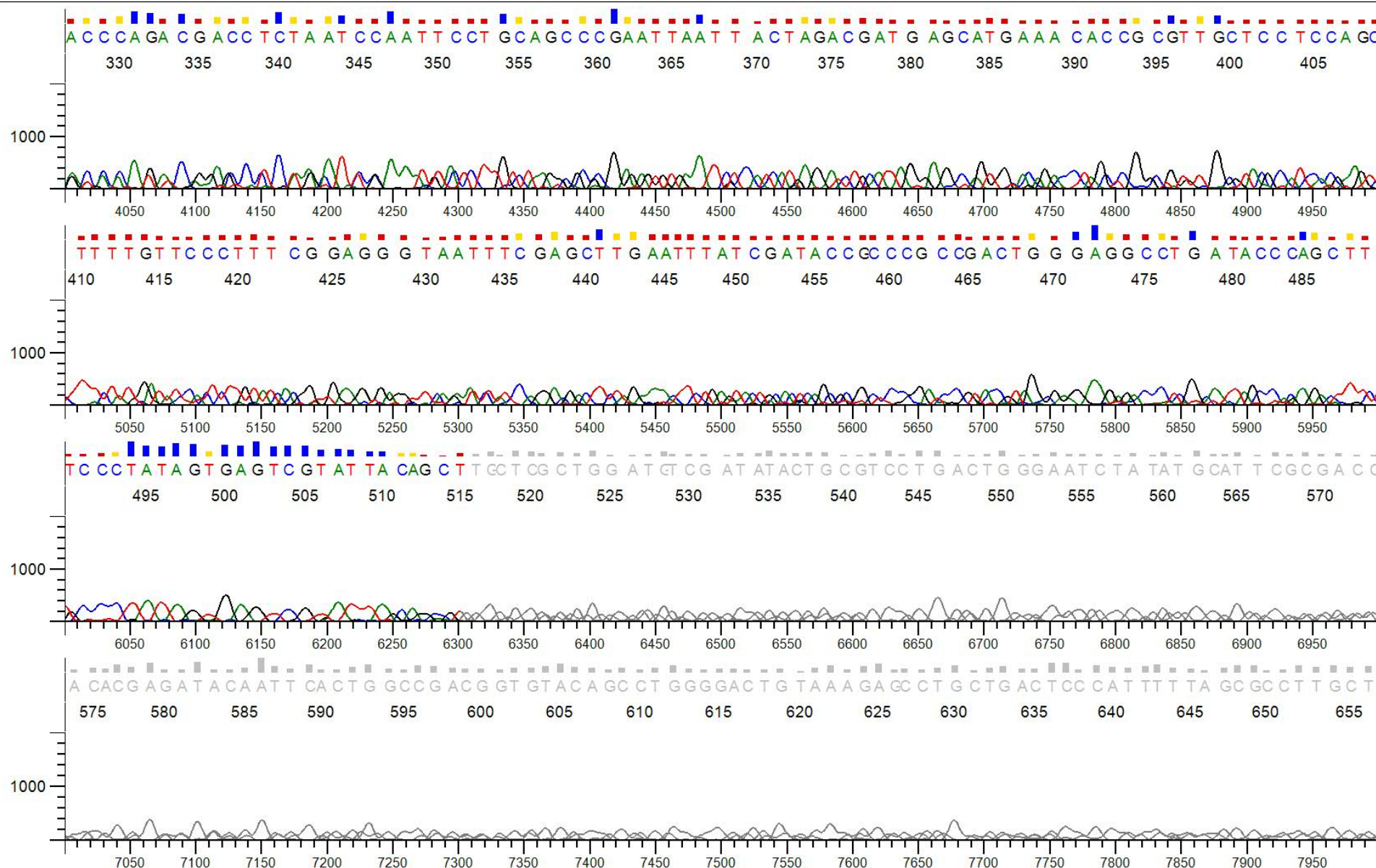

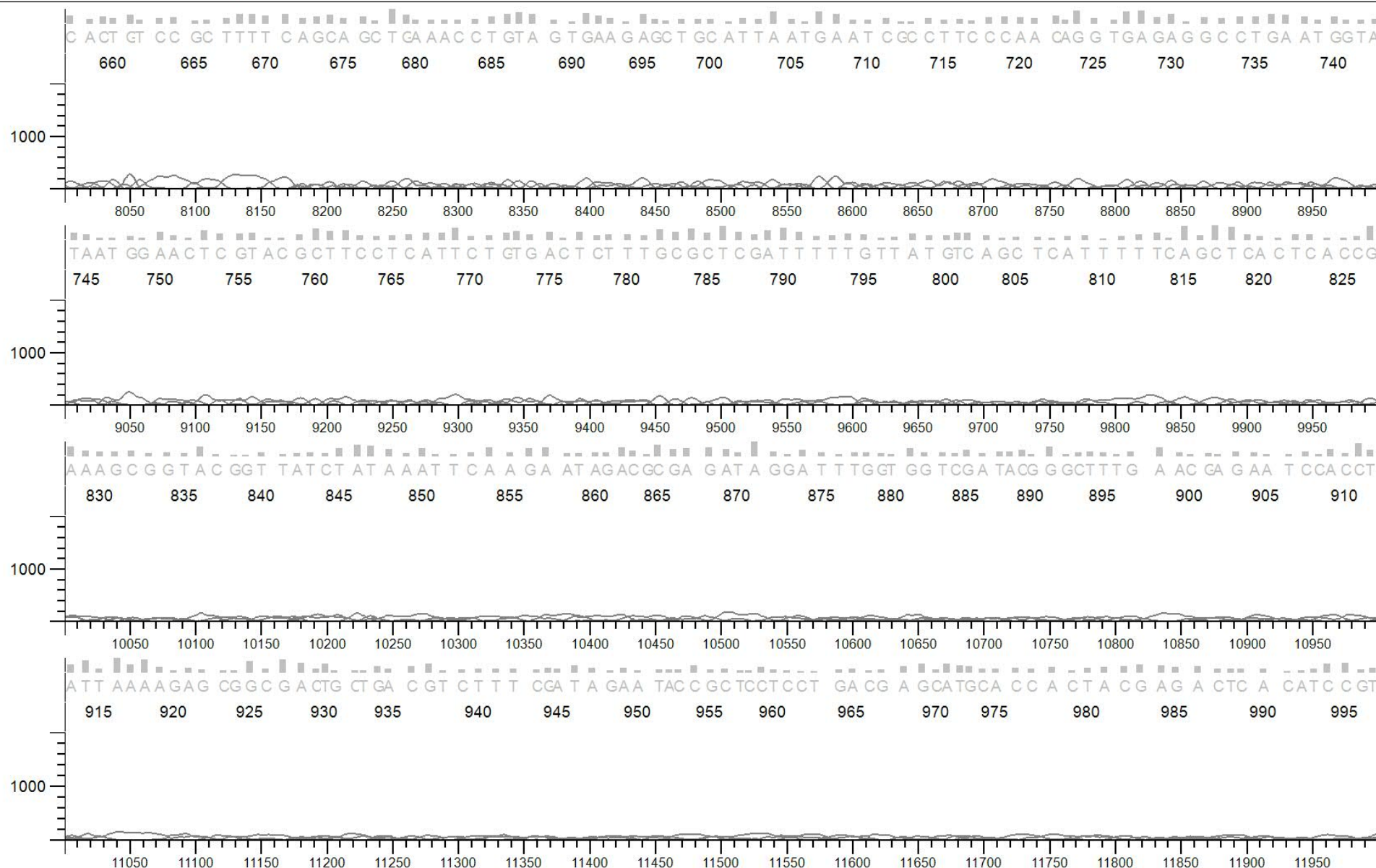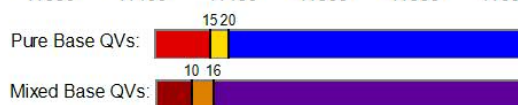

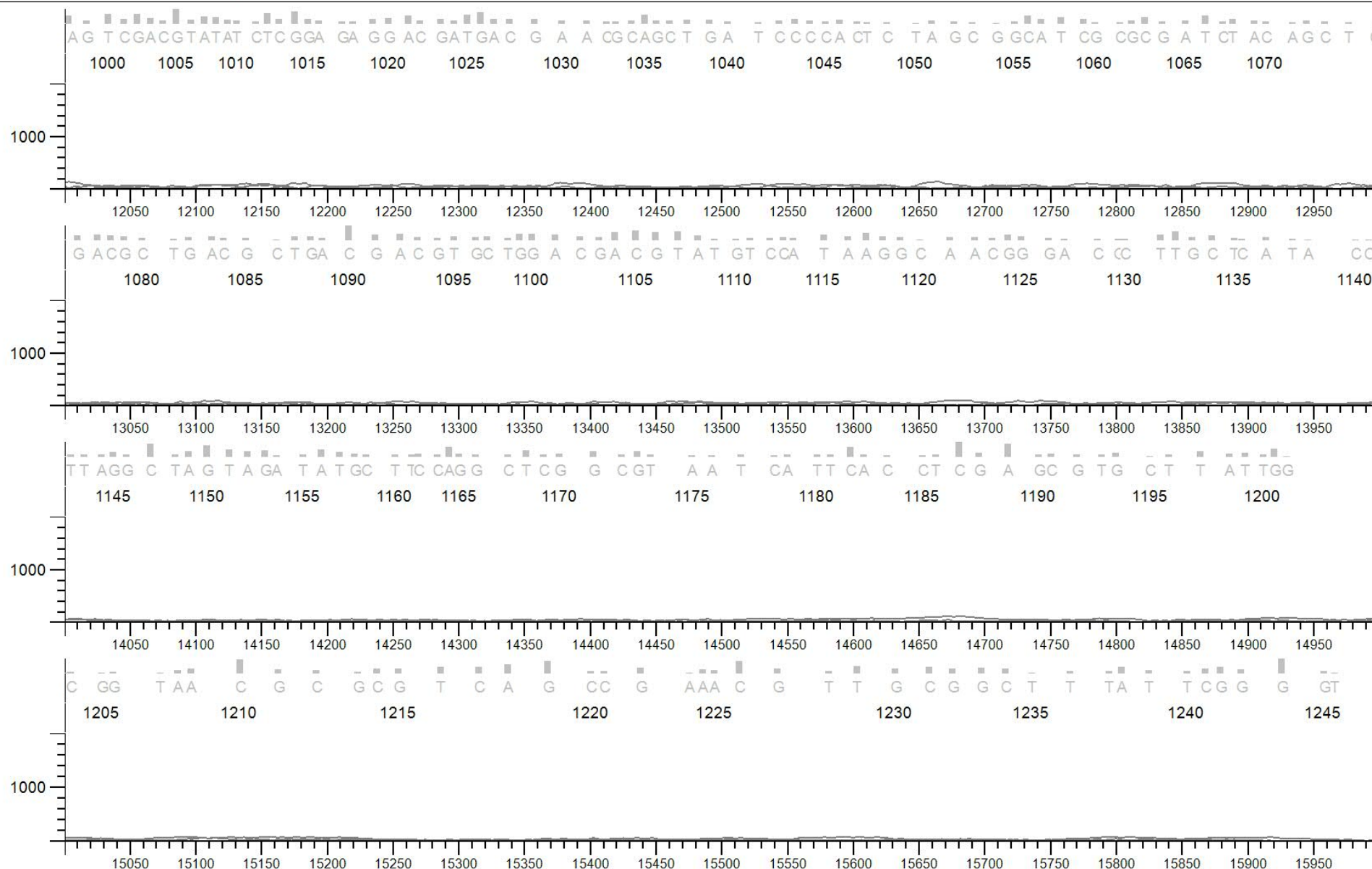

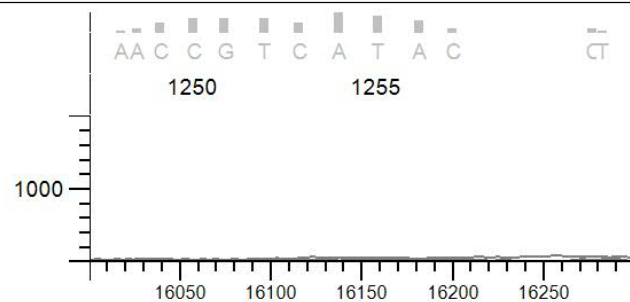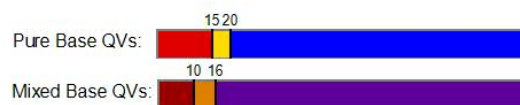

Supplement: Figure 3—source data 2. [file elife-69916-fig3-data2.zip › Figure 3B.C_Source data3_Bisulphite sequencing_mtDNA/SD-MT-DNA-BSF-2.4_T7FOR-H05.pdf]

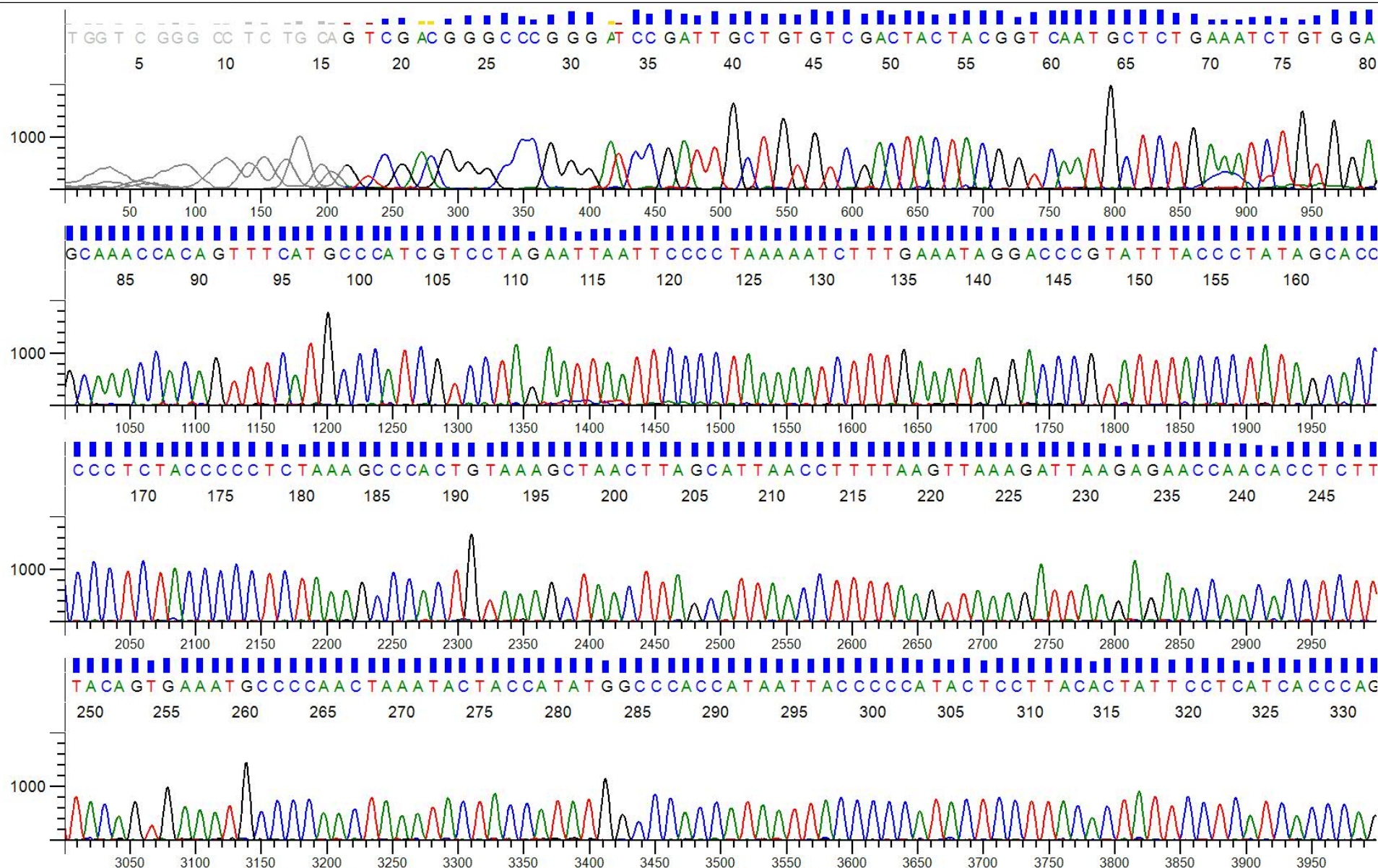

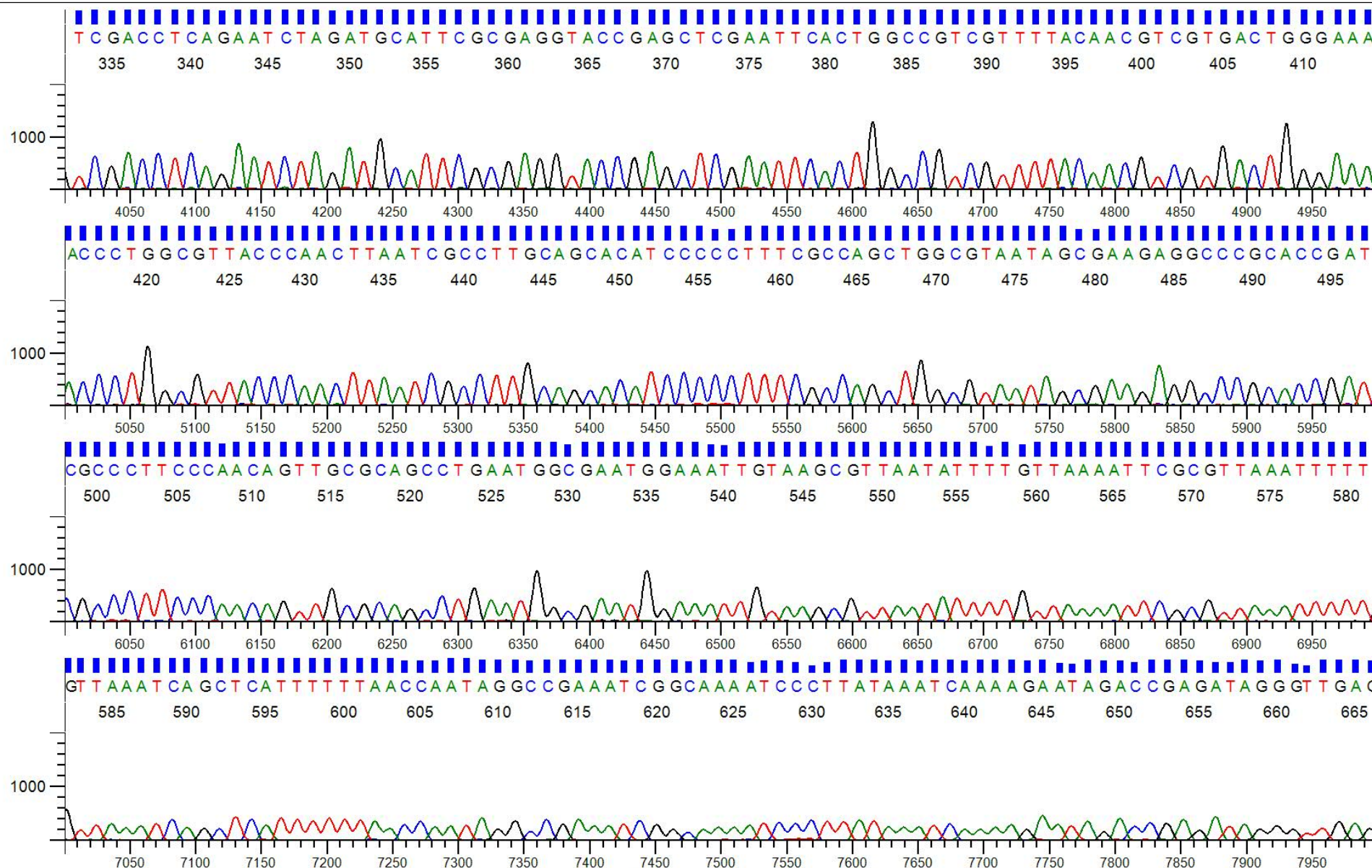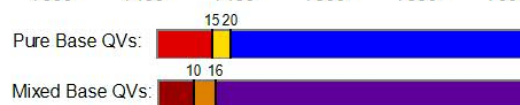

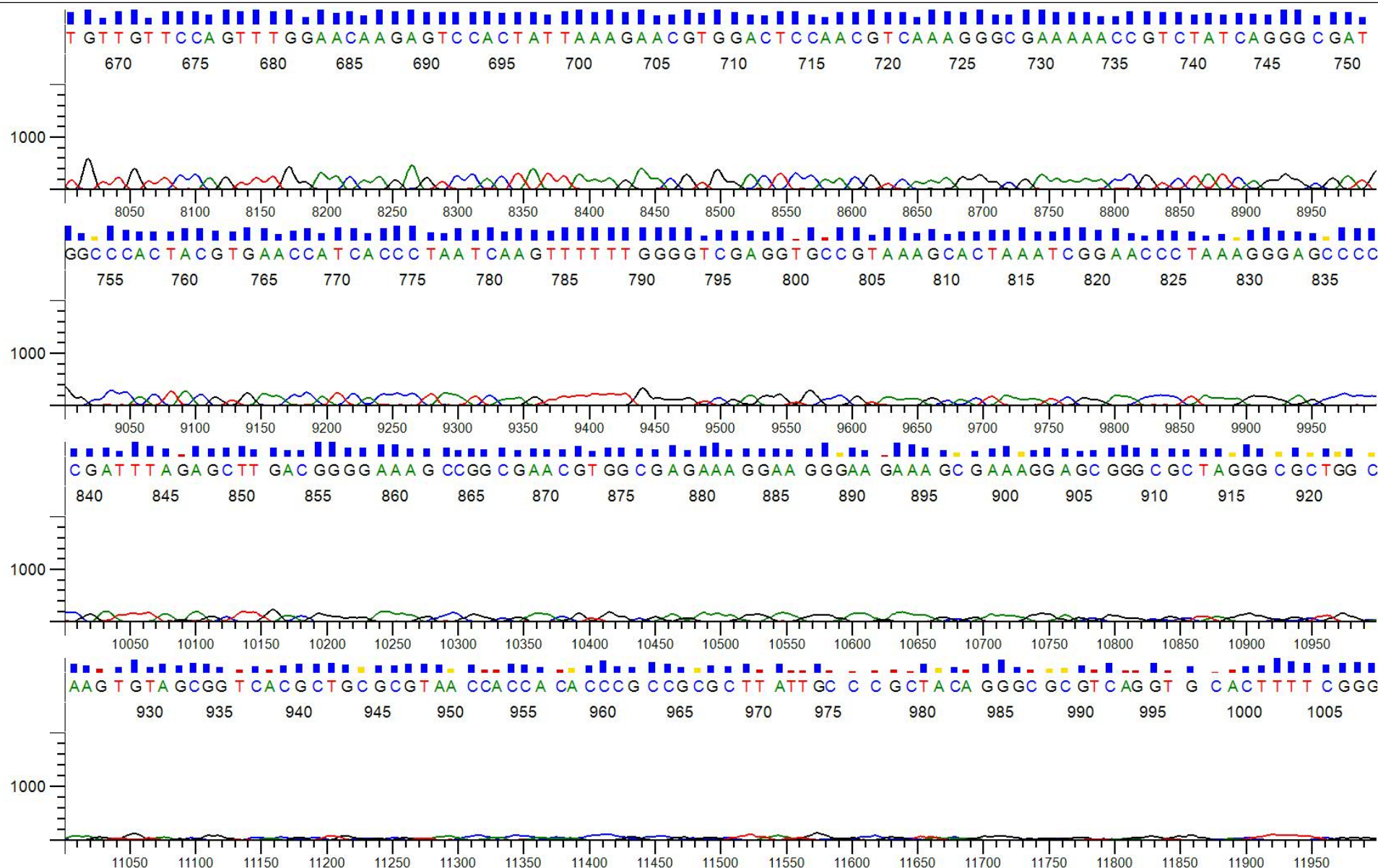

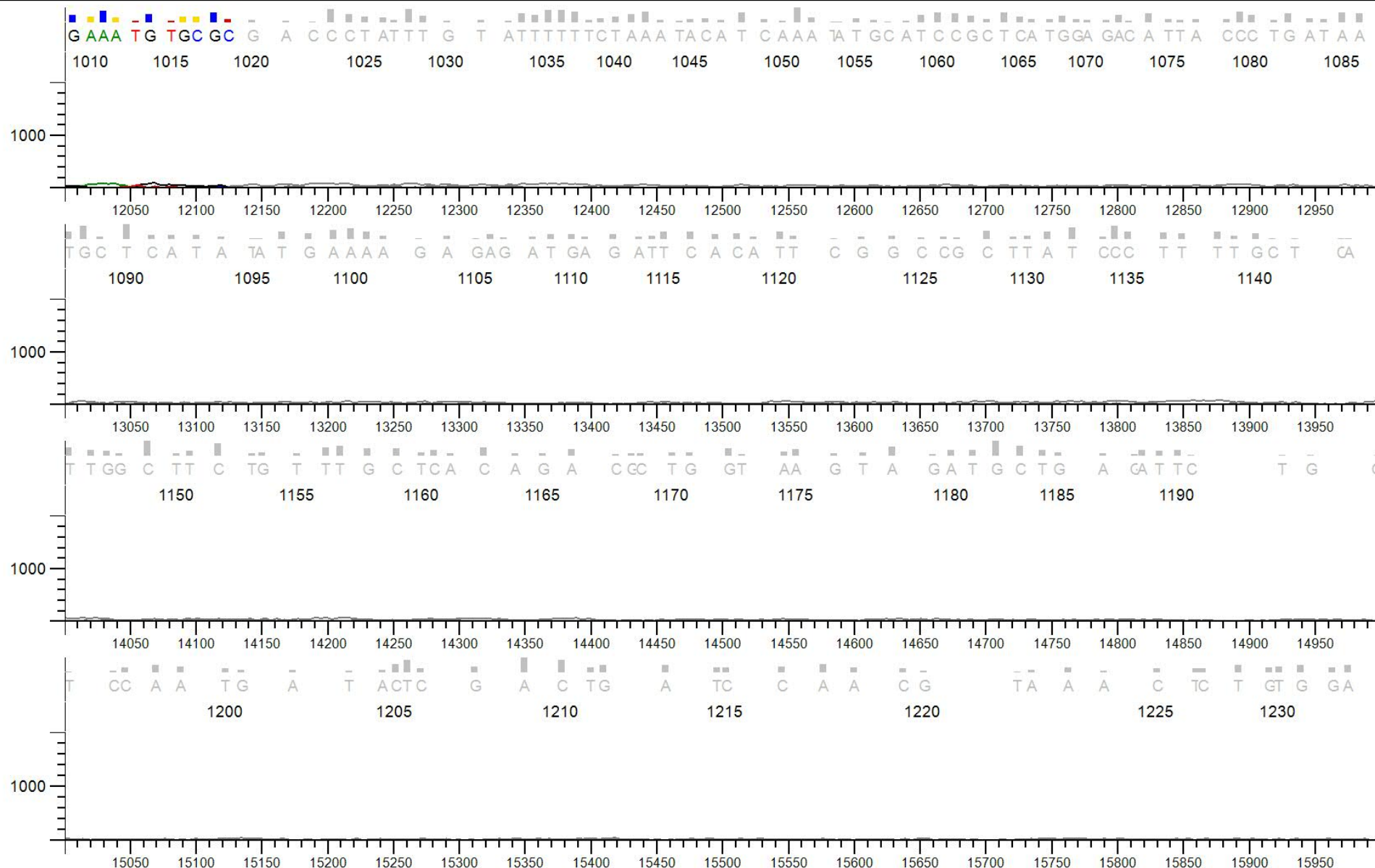

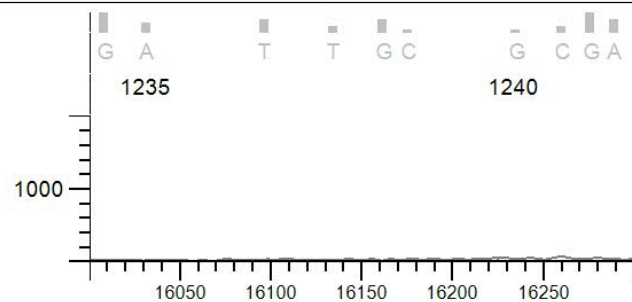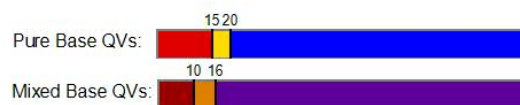

Supplement: Figure 3—source data 2. [file elife-69916-fig3-data2.zip › Figure 3B.C_Source data3_Bisulphite sequencing_mtDNA/SD_MTDNA_BSF_1.3_T7FOR-B02.pdf]

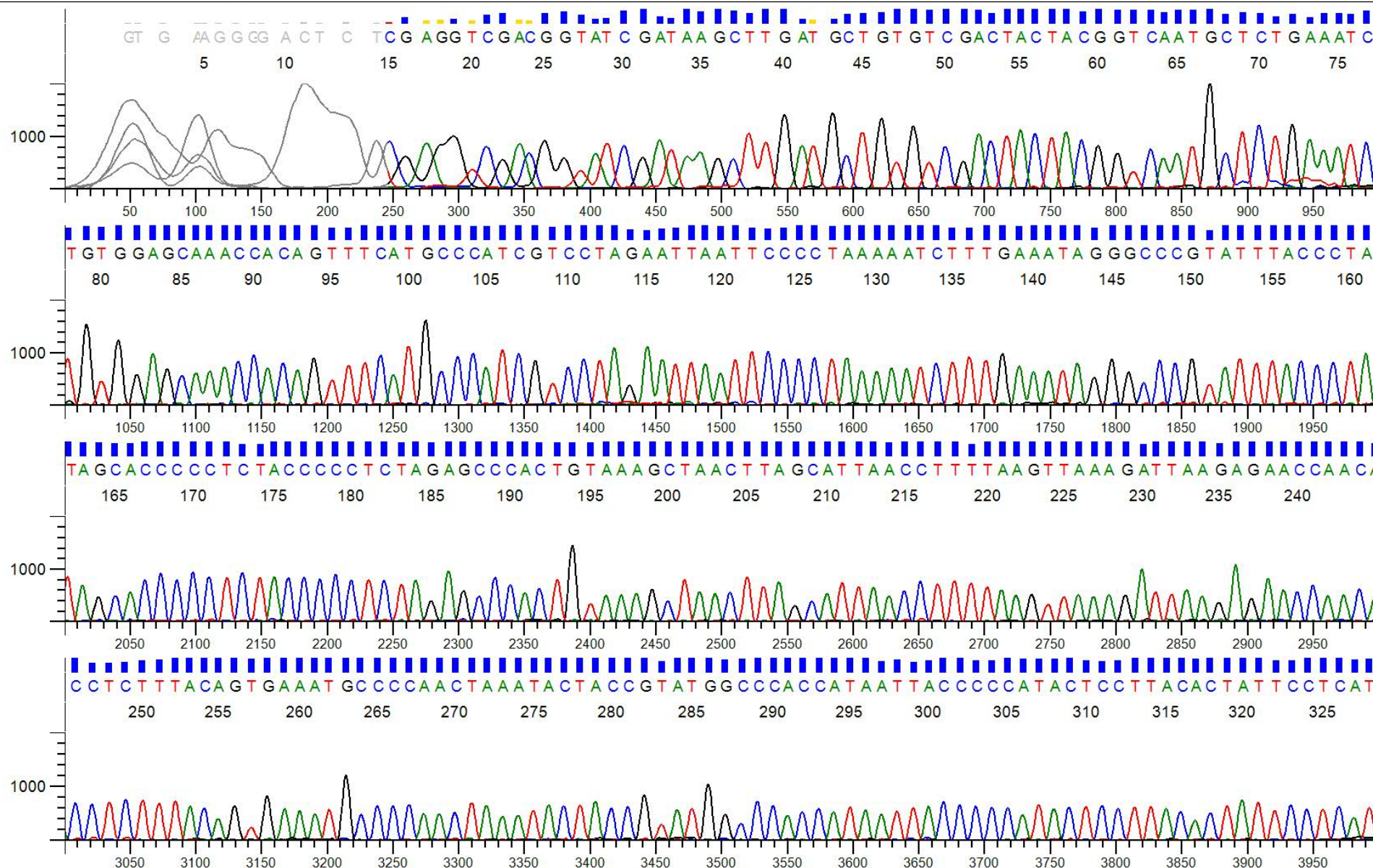

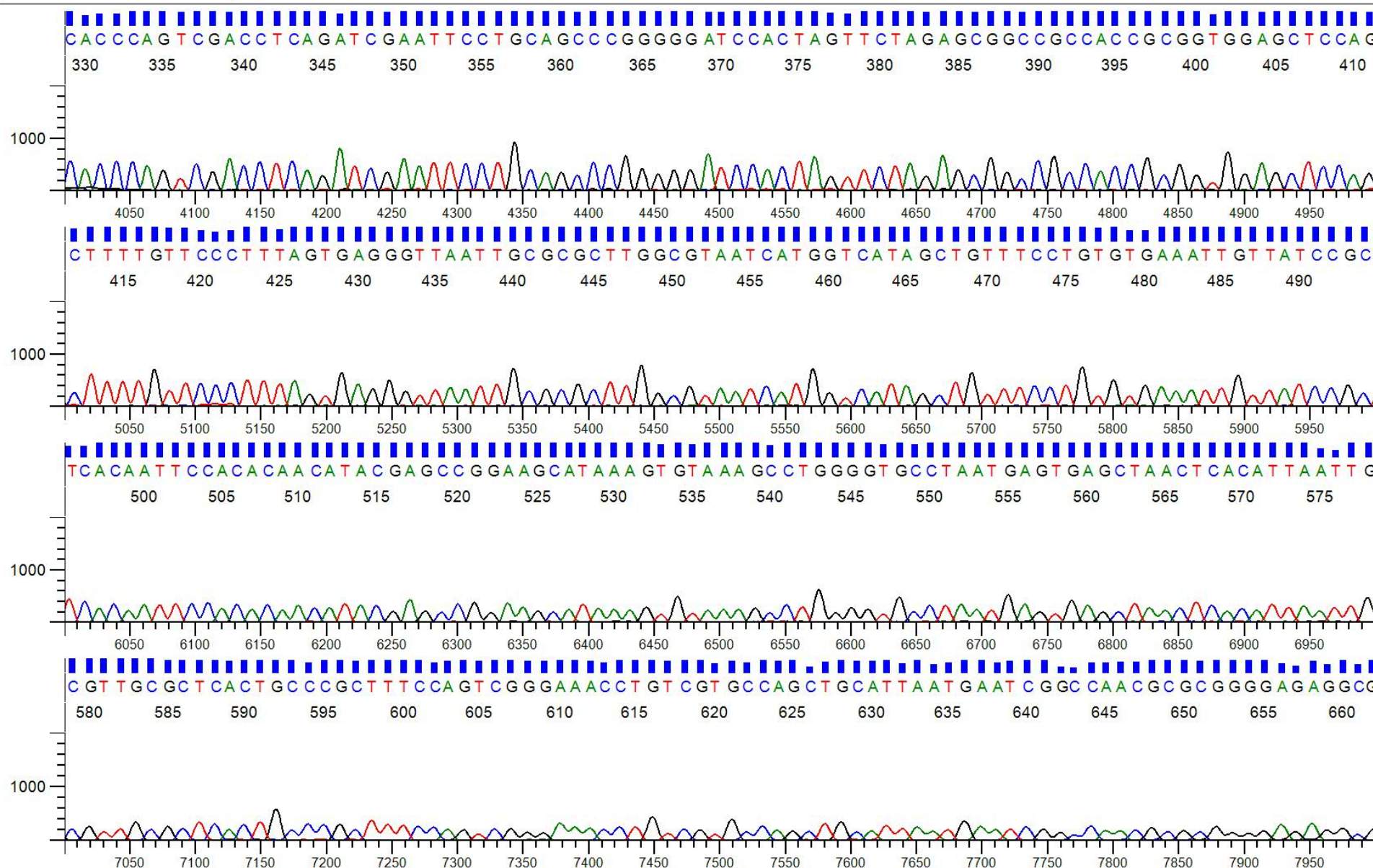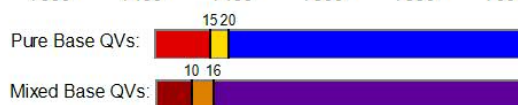

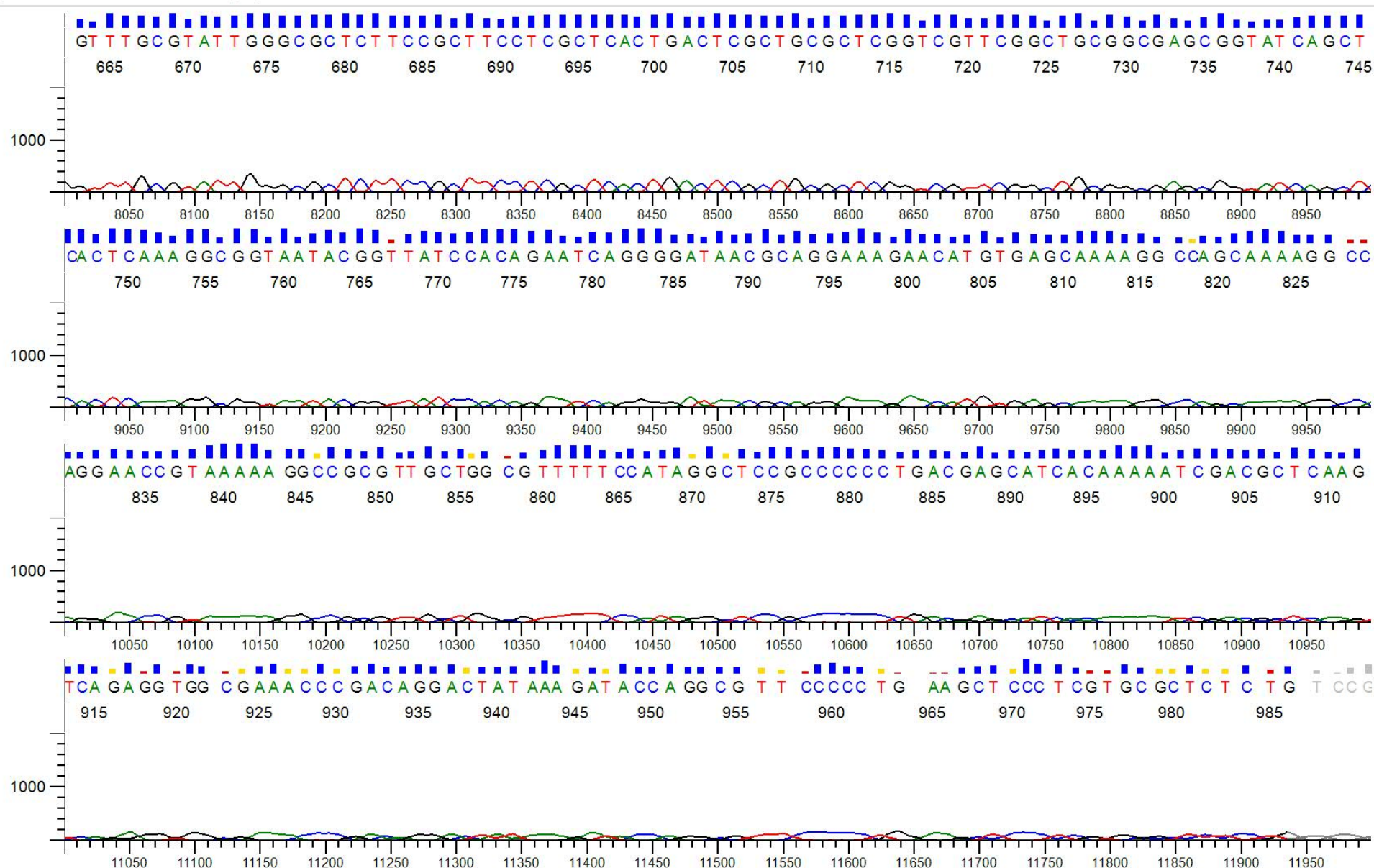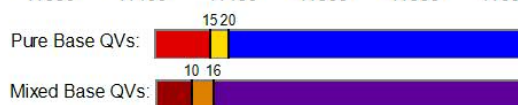

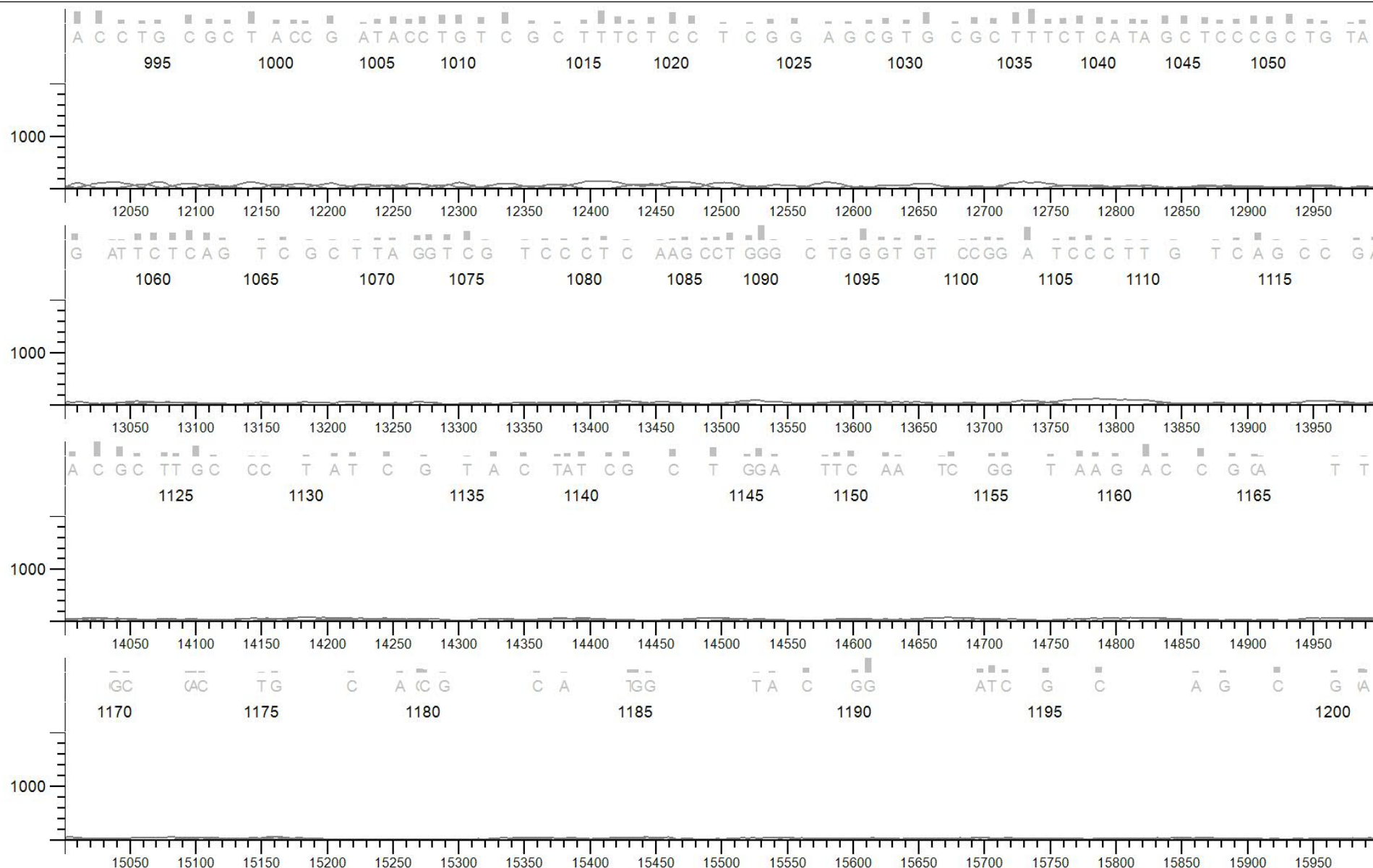

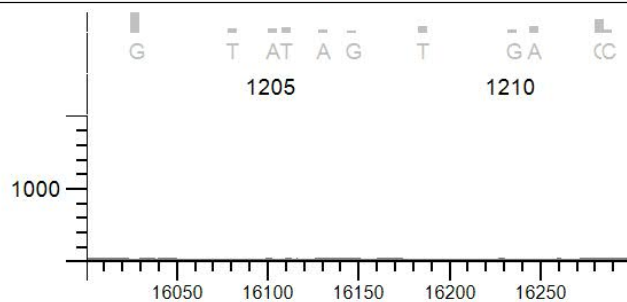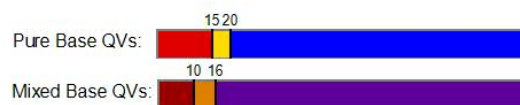

Supplement: Figure 3—source data 2. [file elife-69916-fig3-data2.zip › Figure 3B.C_Source data3_Bisulphite sequencing_mtDNA/SD-MT-DNA-BSF-2.1_T7FOR-F05.pdf]

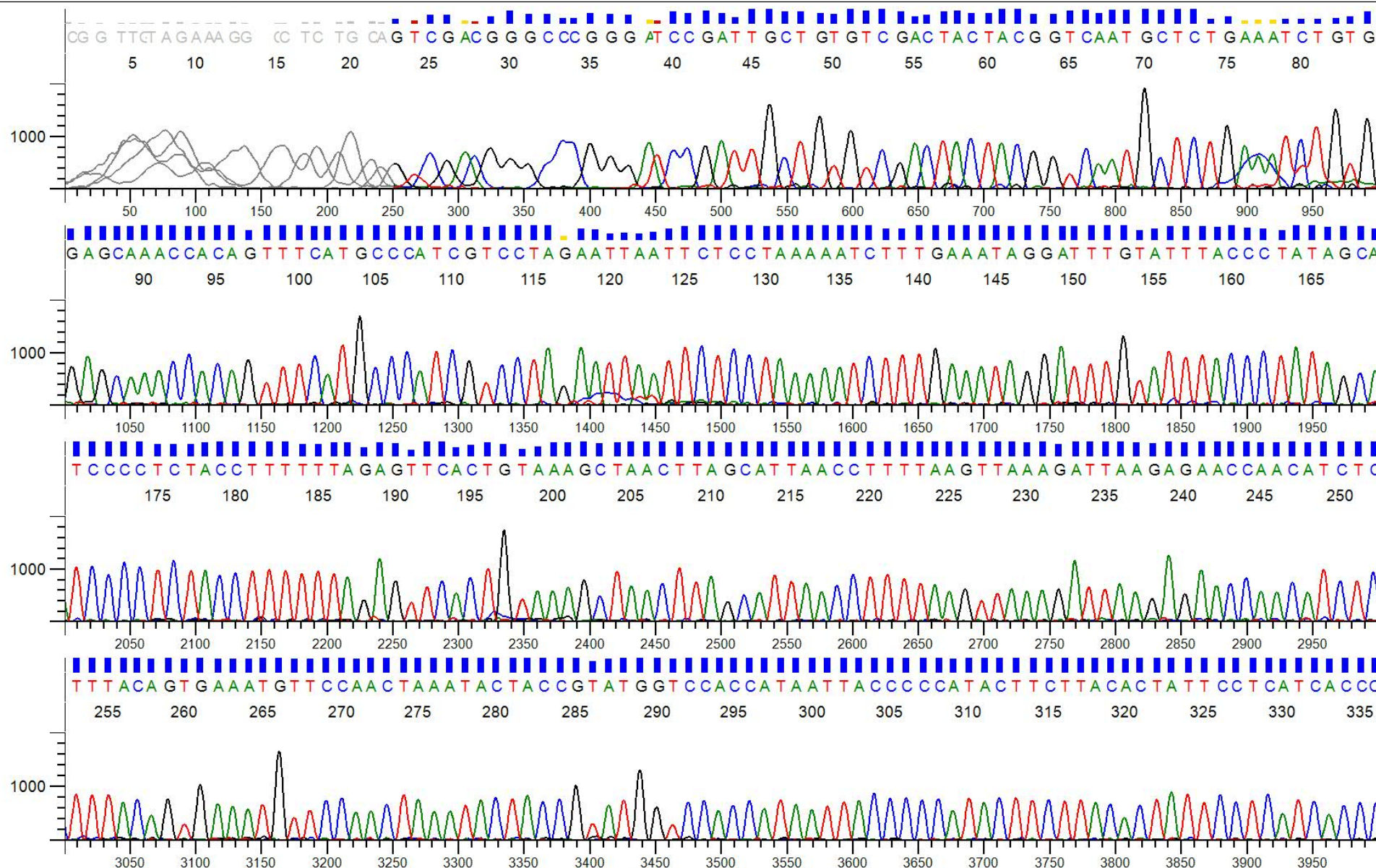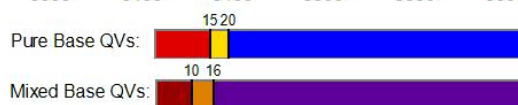

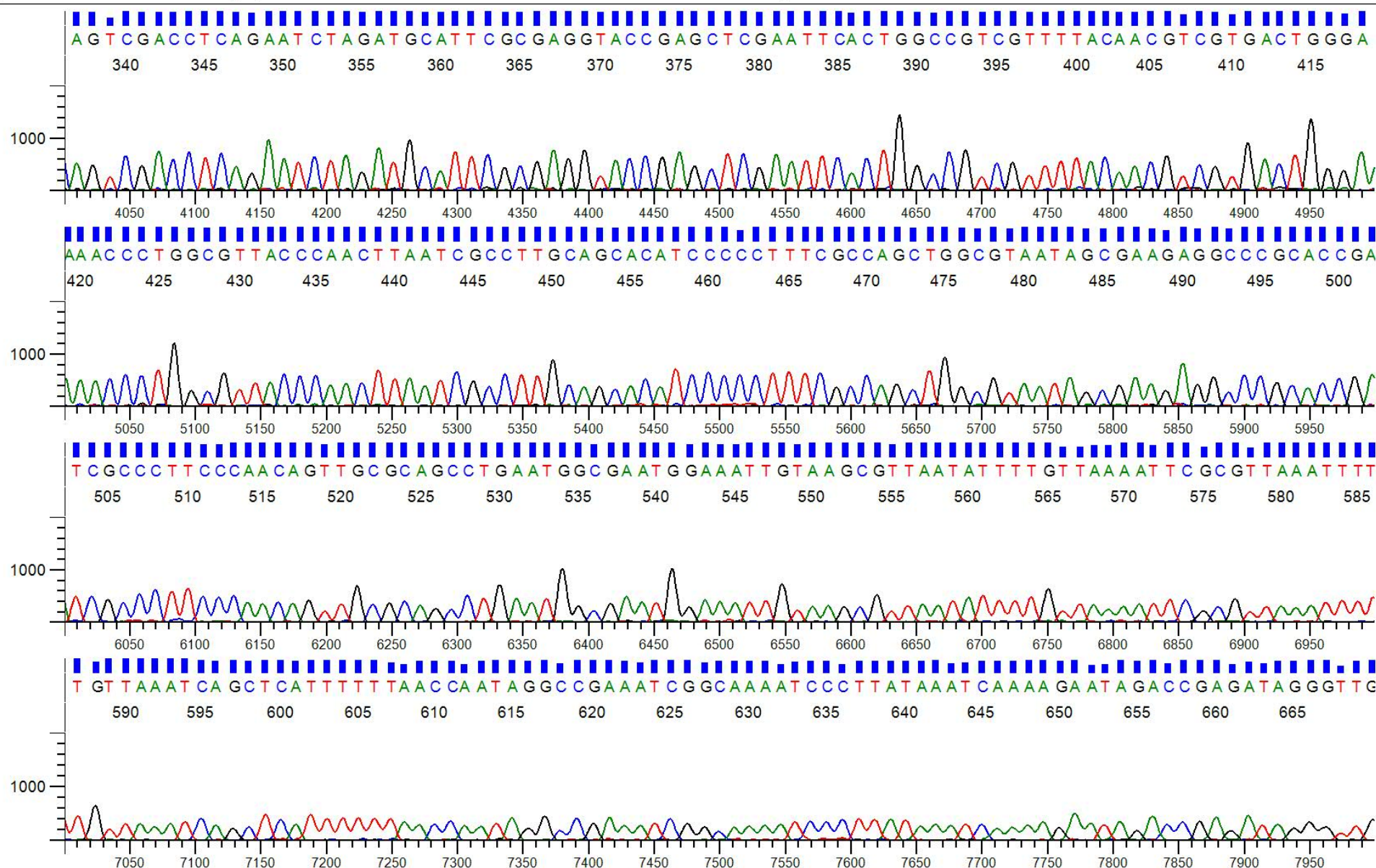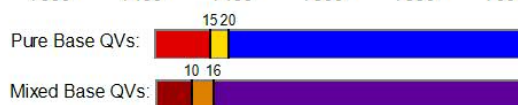

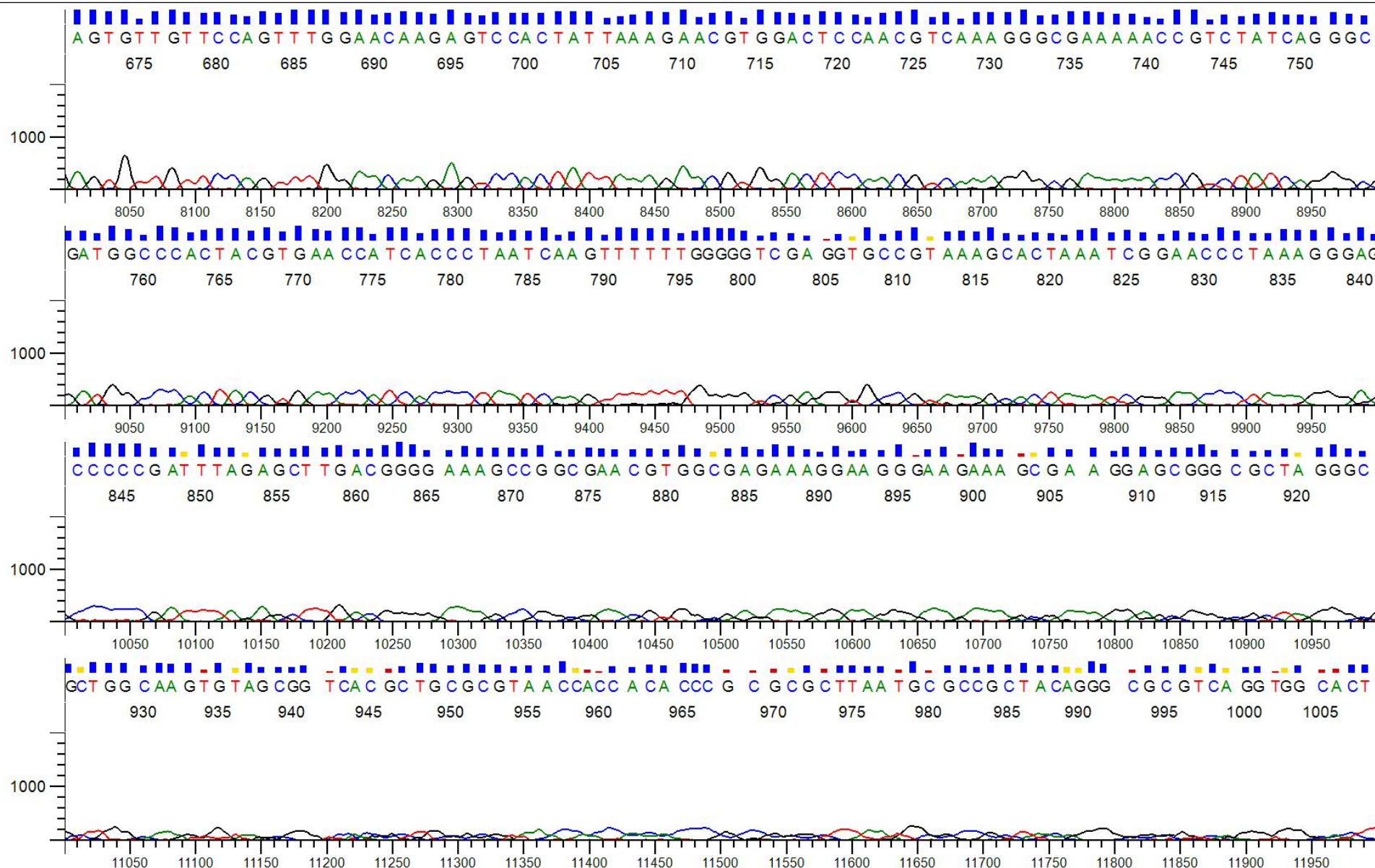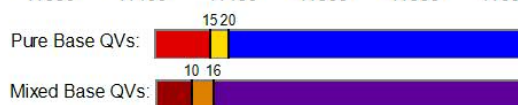

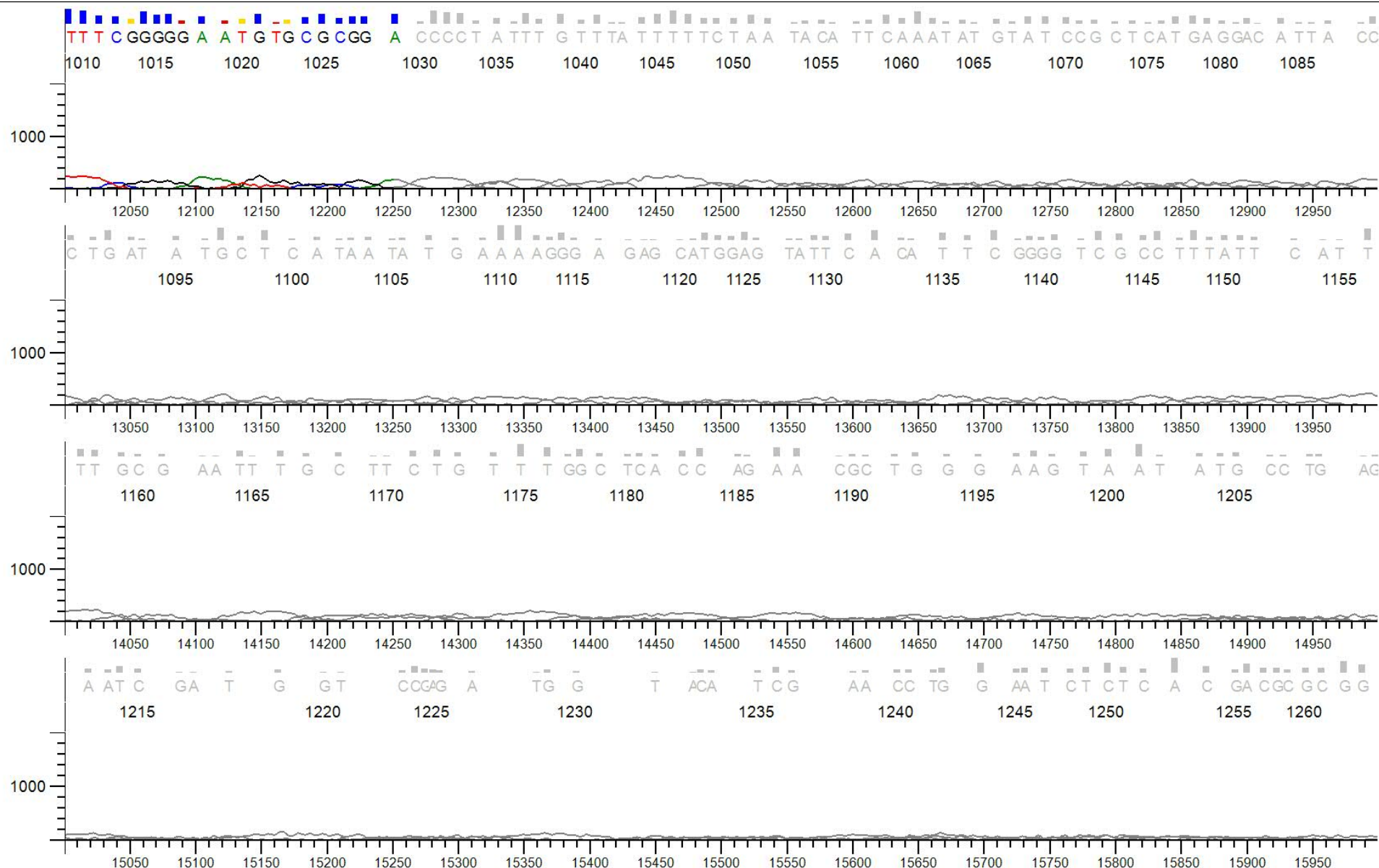

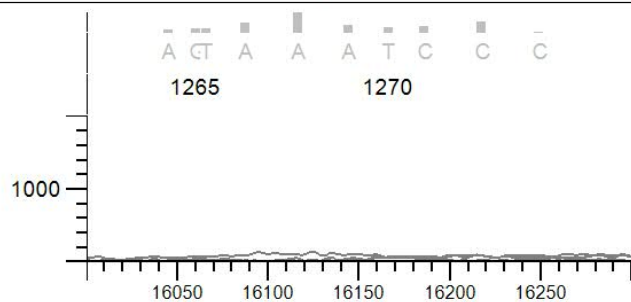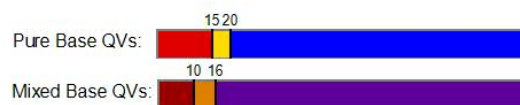

Supplement: Figure 3—source data 2. [file elife-69916-fig3-data2.zip › Figure 3B.C_Source data3_Bisulphite sequencing_mtDNA/SS4_MT_BIS_1.3_T7FOR.pdf]

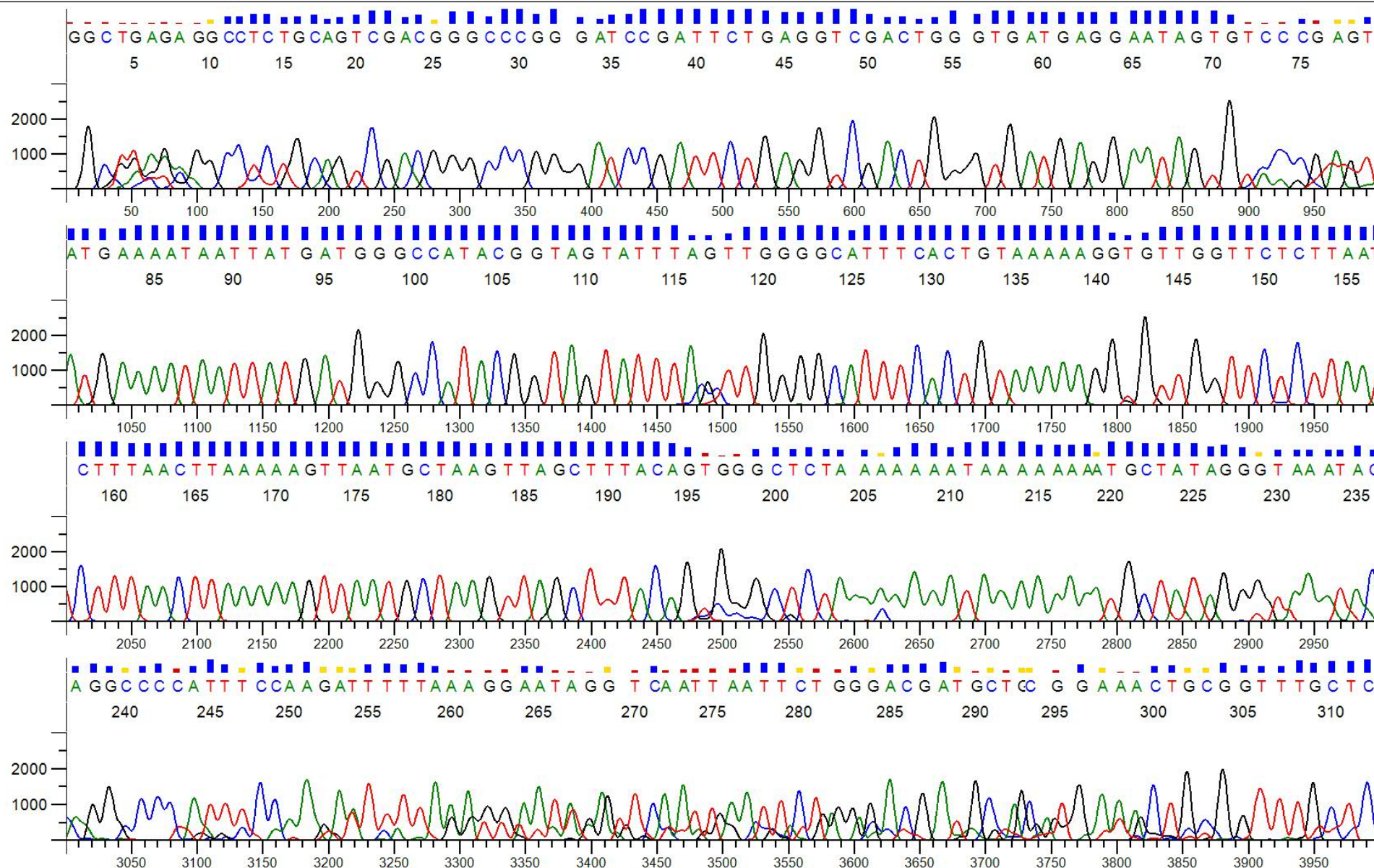

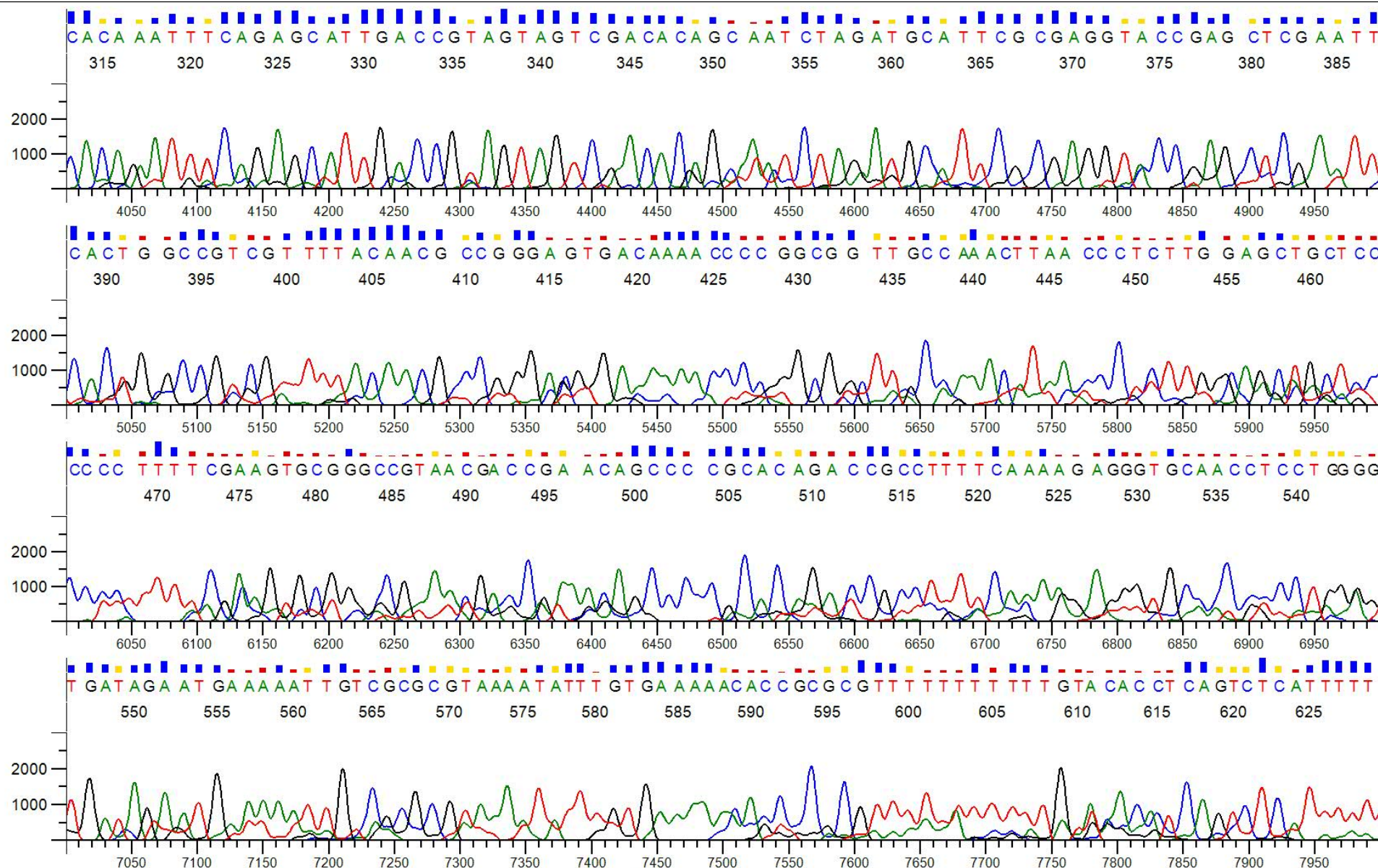

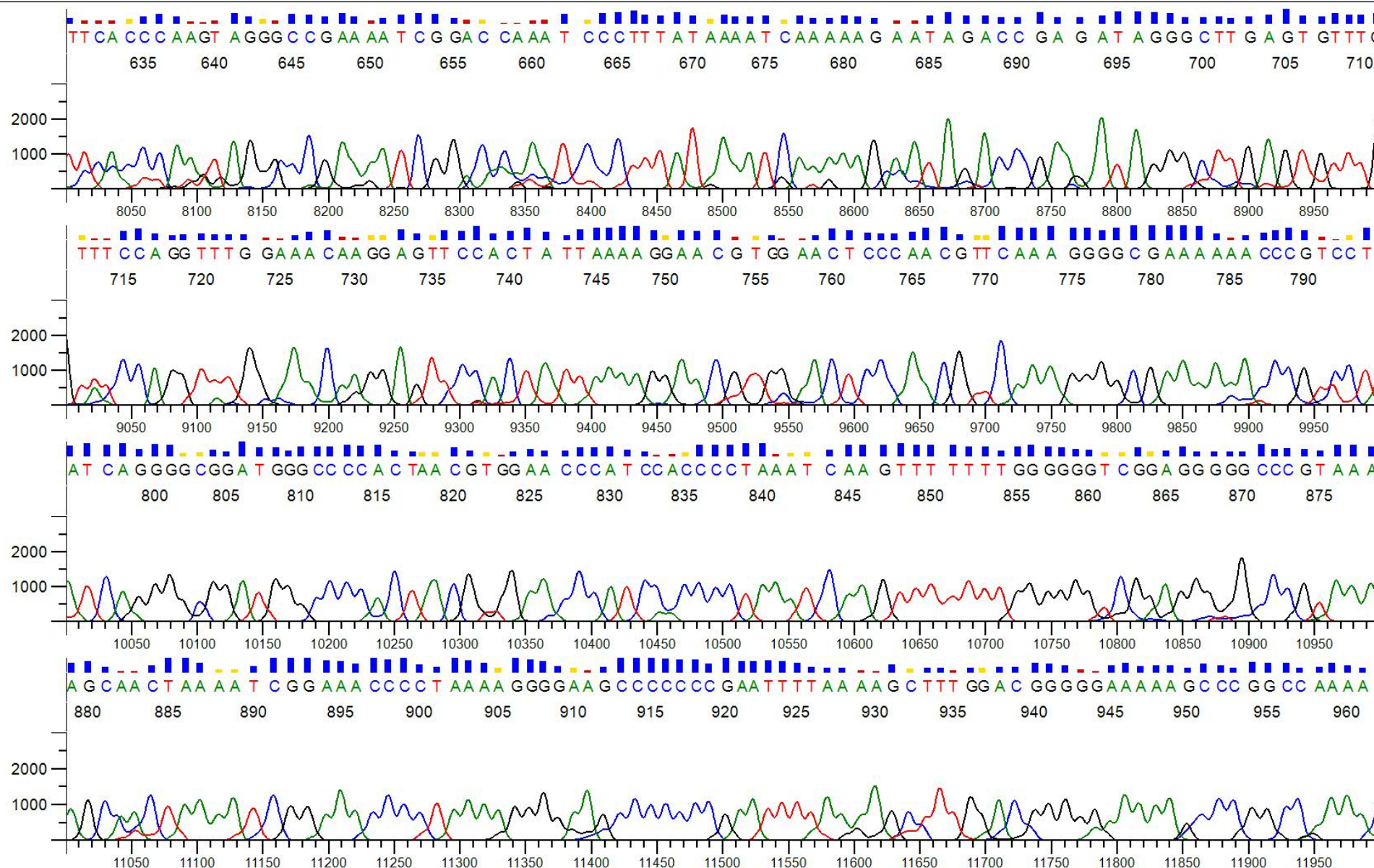

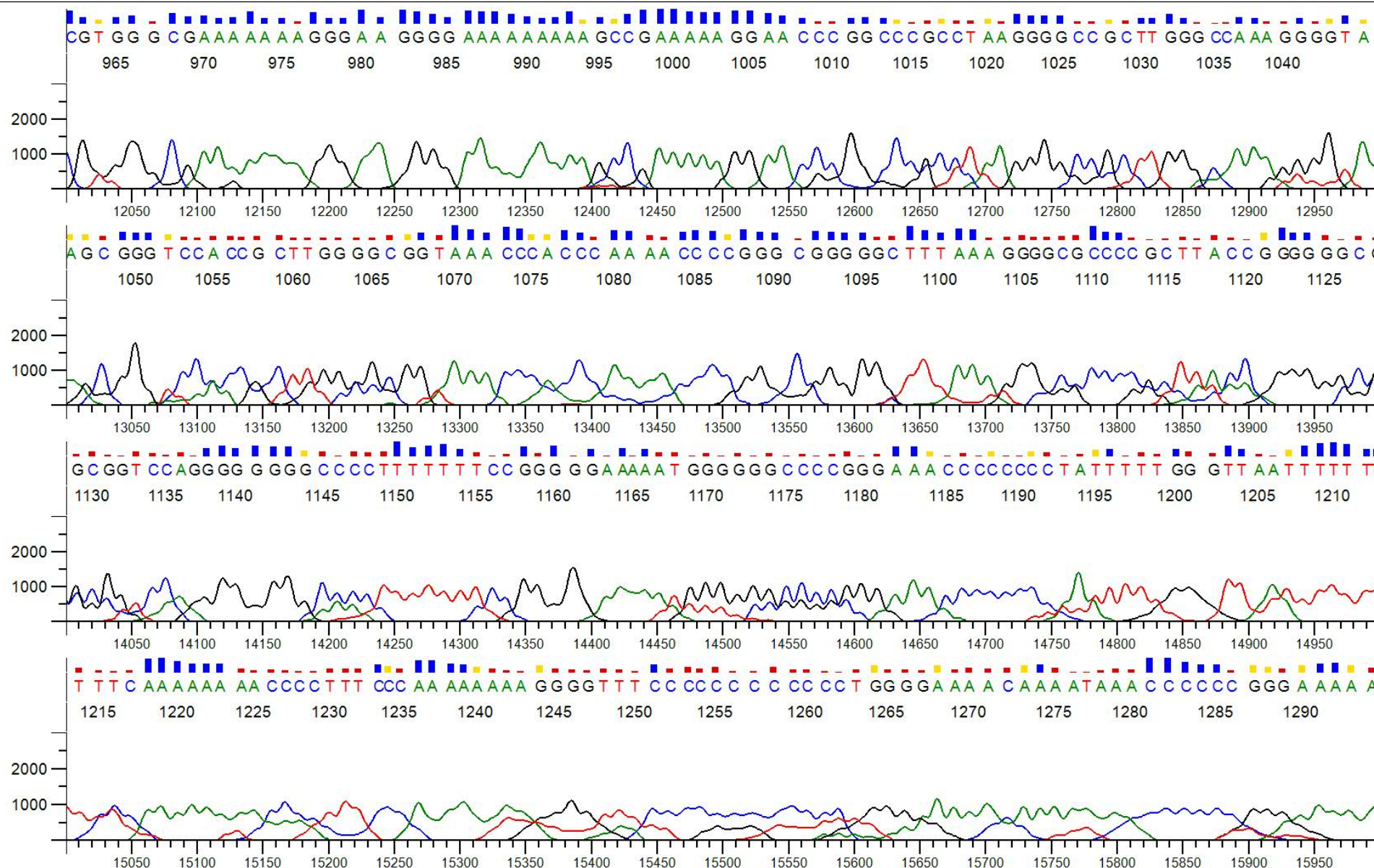

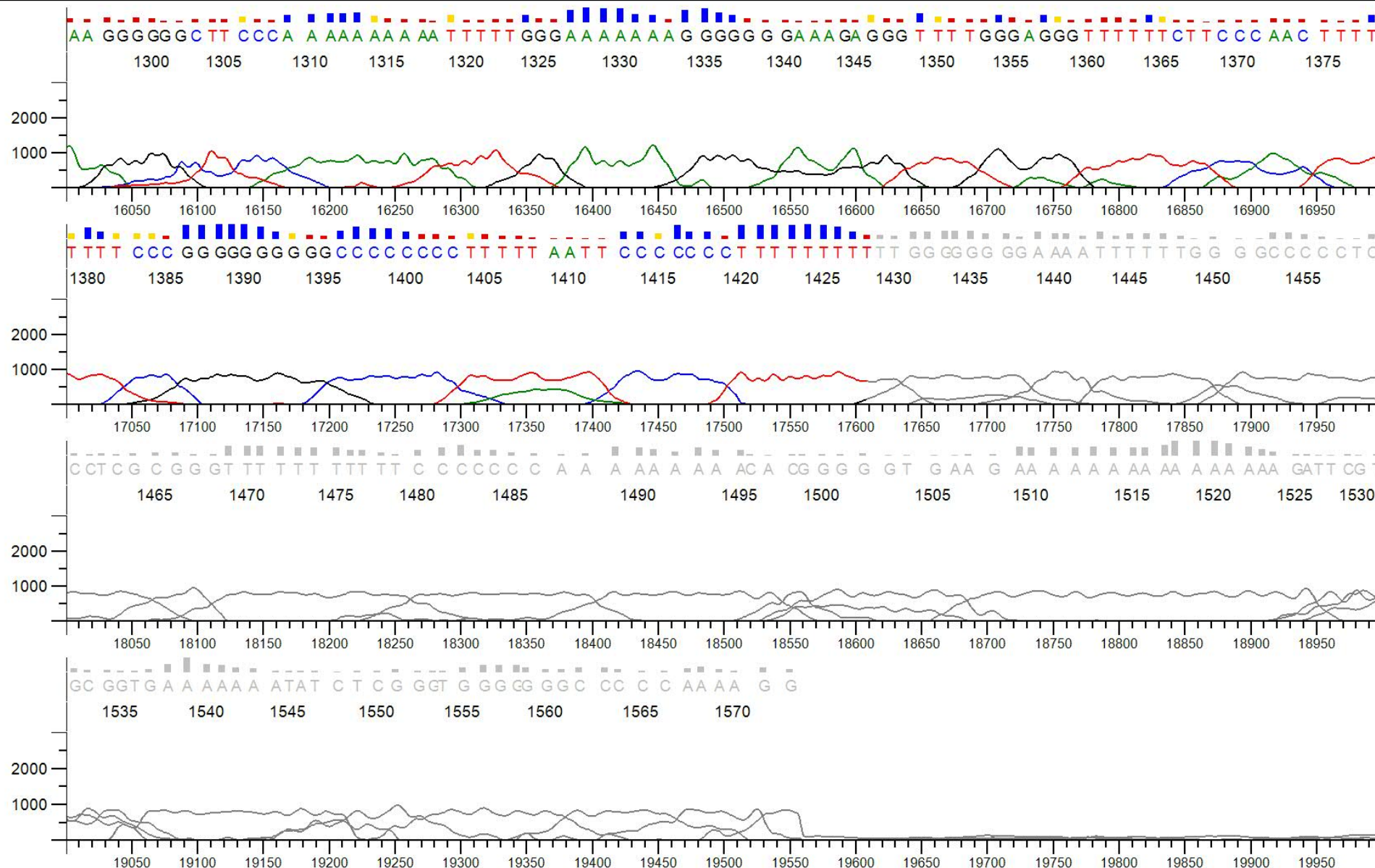

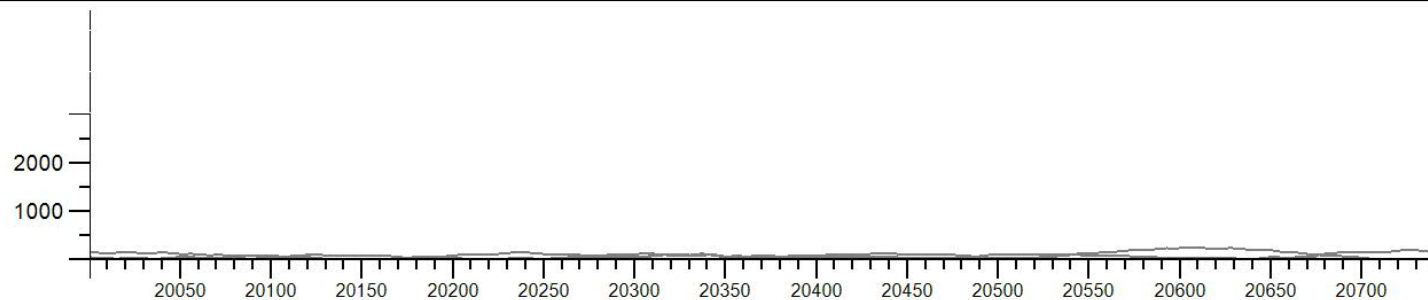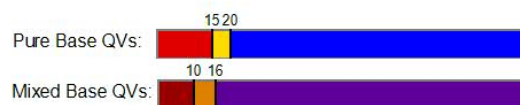

Supplement: Figure 3—source data 2. [file elife-69916-fig3-data2.zip › Figure 3B.C_Source data3_Bisulphite sequencing_mtDNA/SS4-MT-BIS-2.23_T7FOR.pdf]

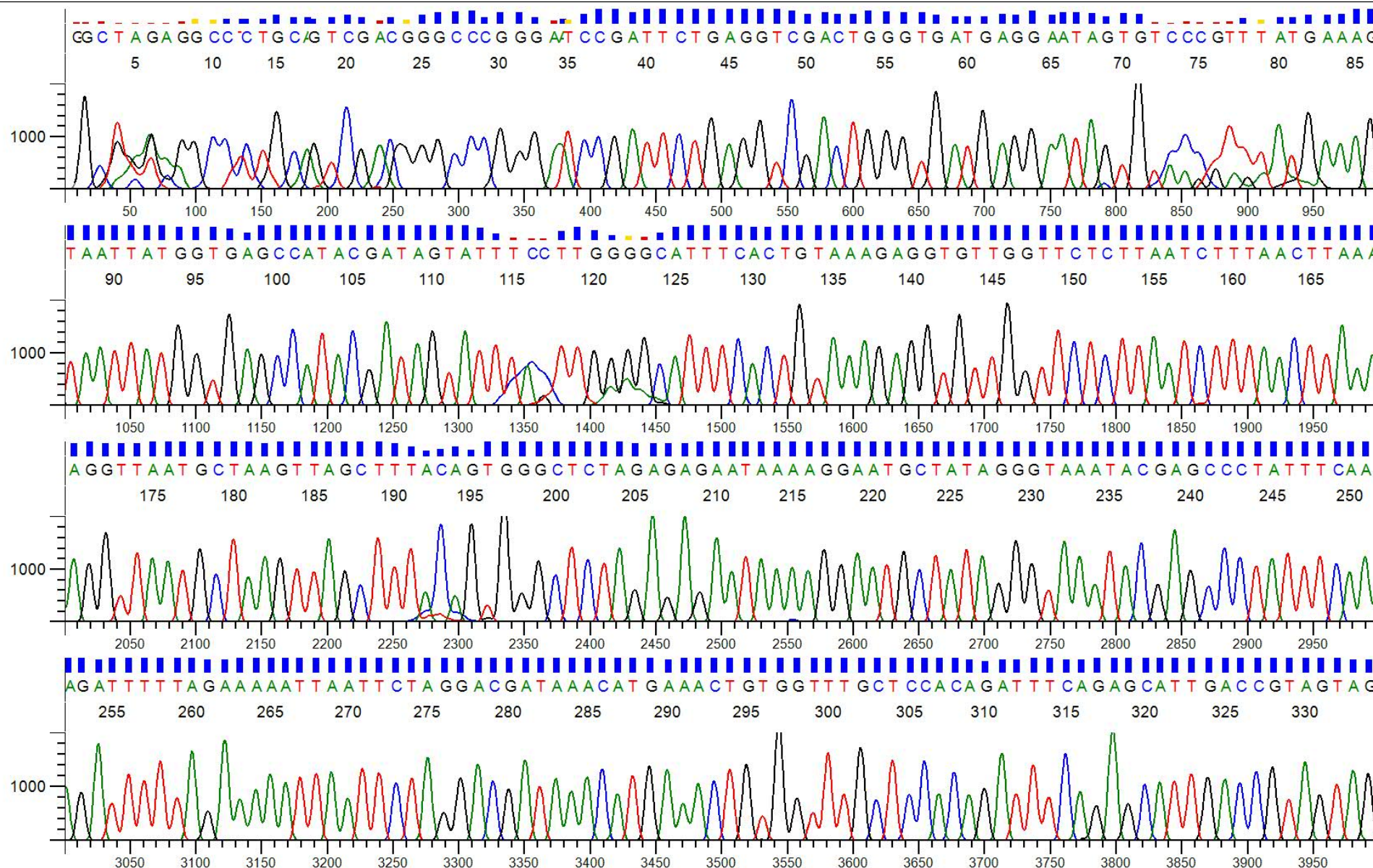

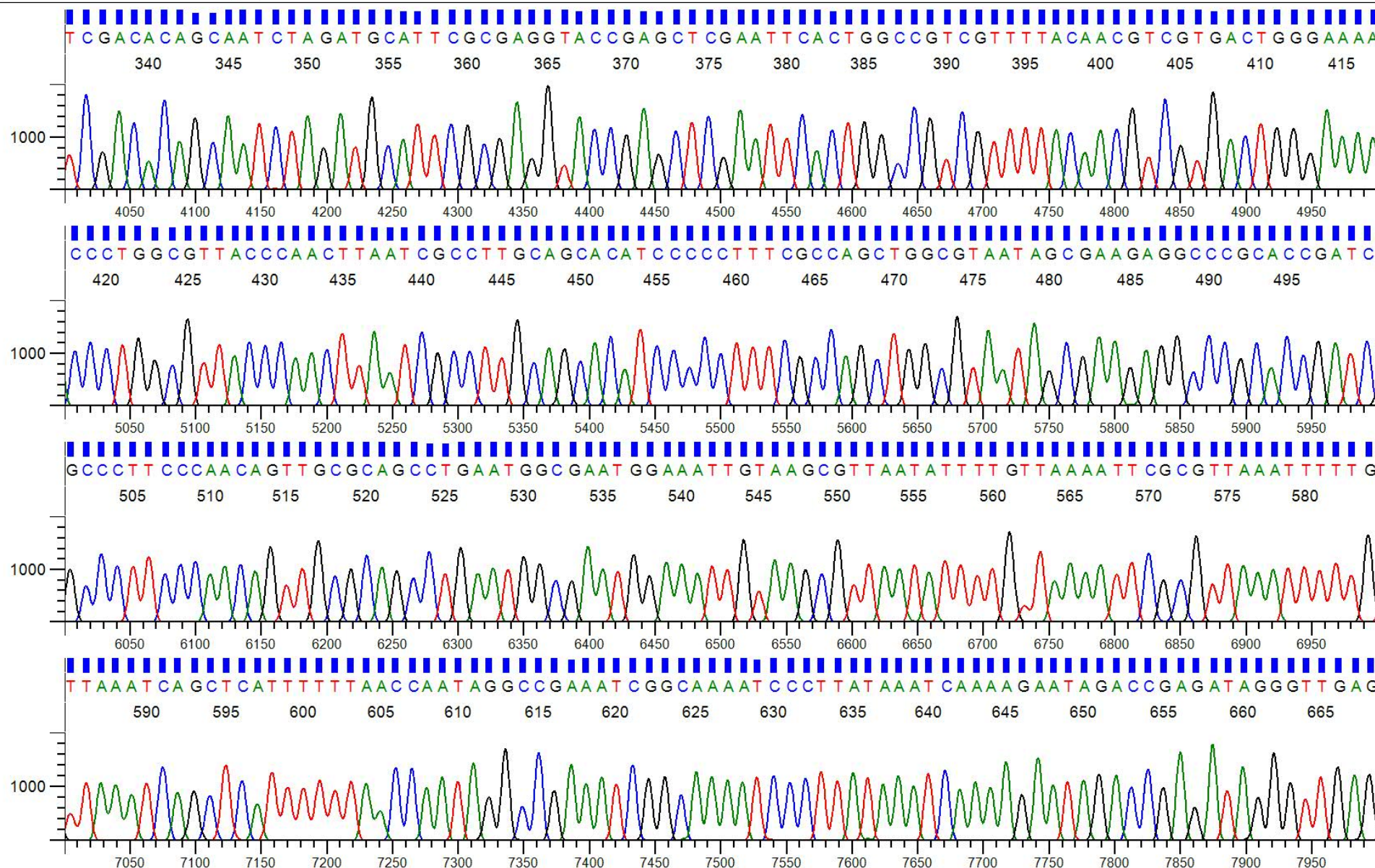

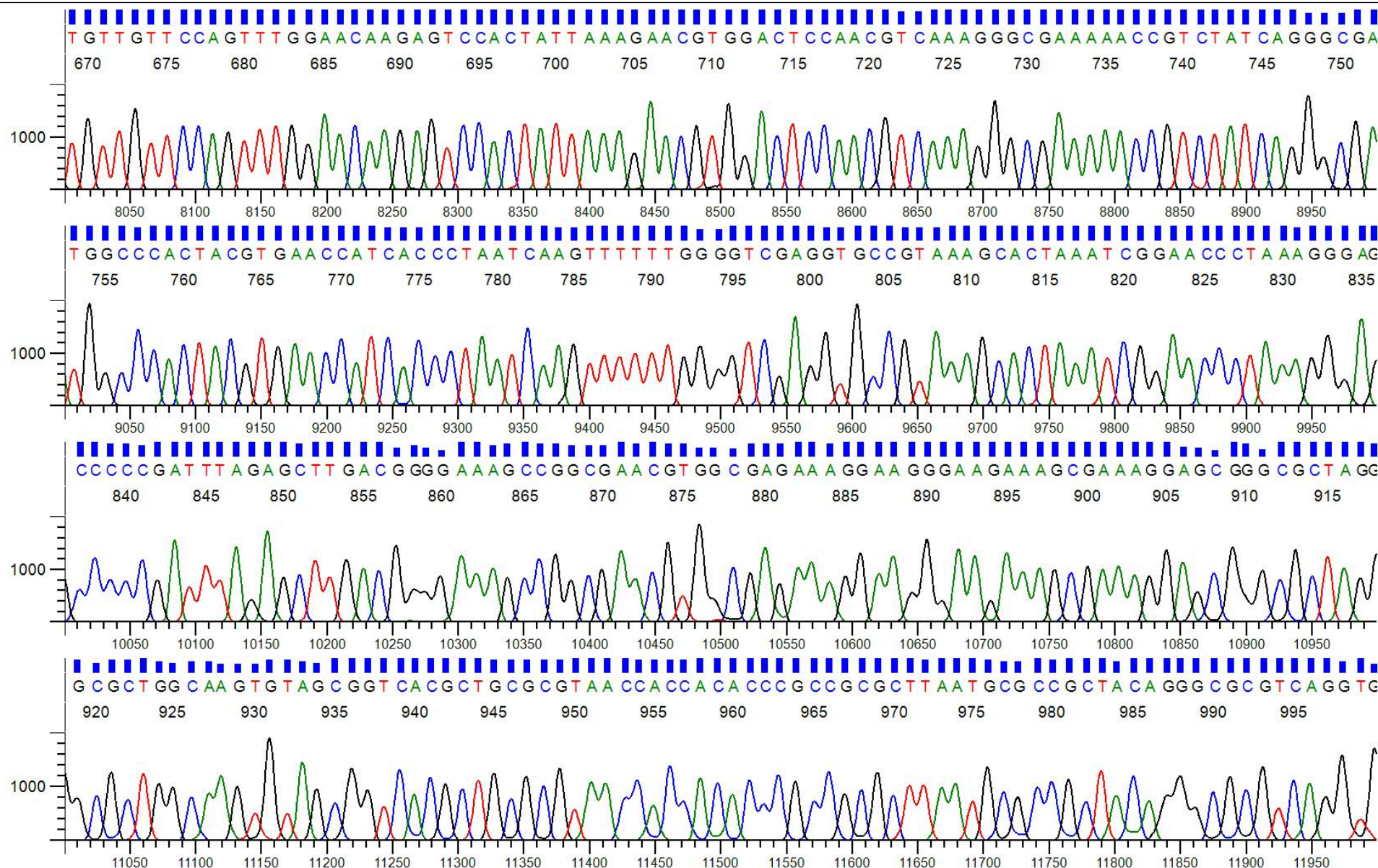

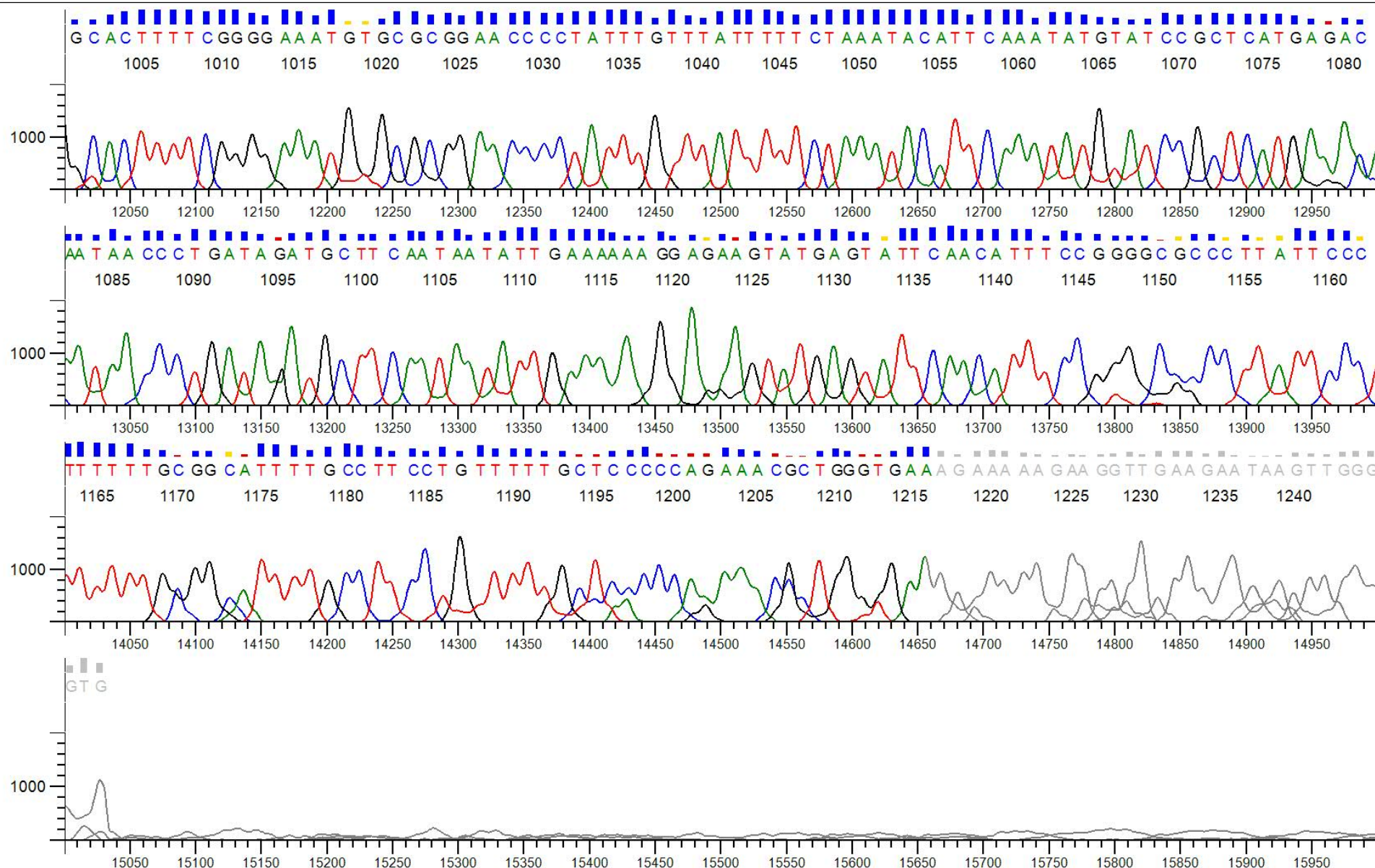

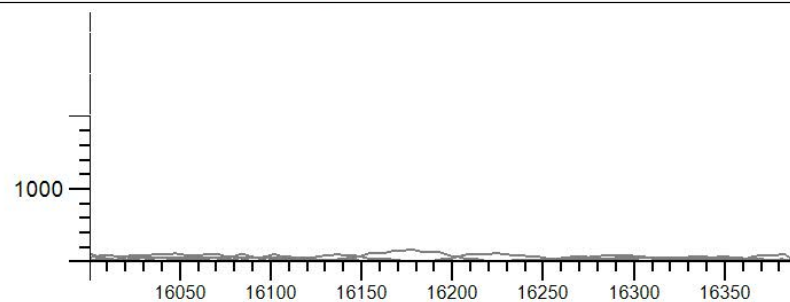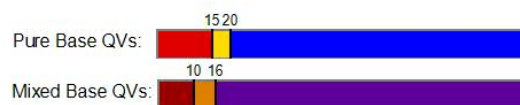

Supplement: Figure 3—source data 2. [file elife-69916-fig3-data2.zip › Figure 3B.C_Source data3_Bisulphite sequencing_mtDNA/SS4-MT-BIS-37_T7FOR-B07.pdf]

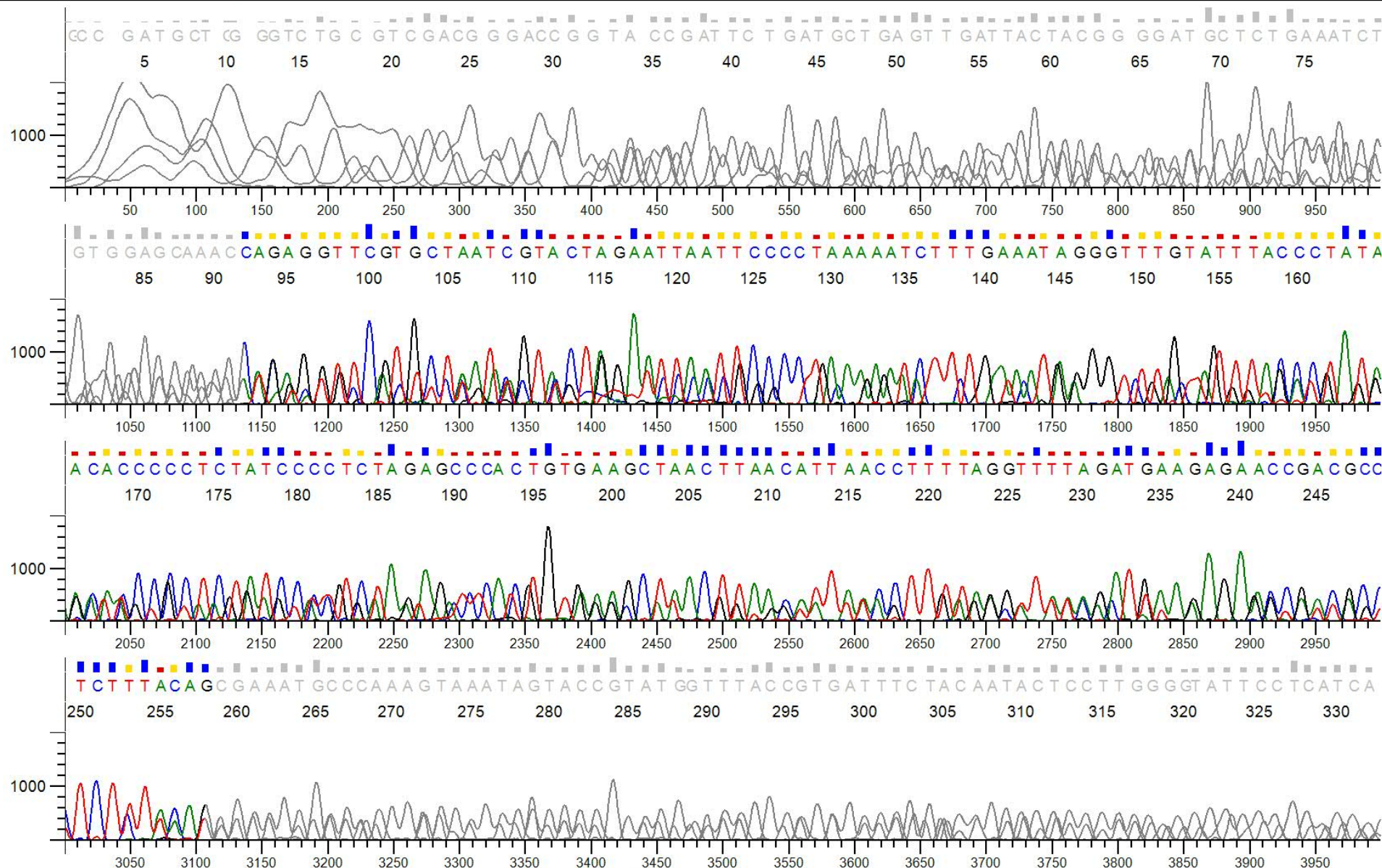

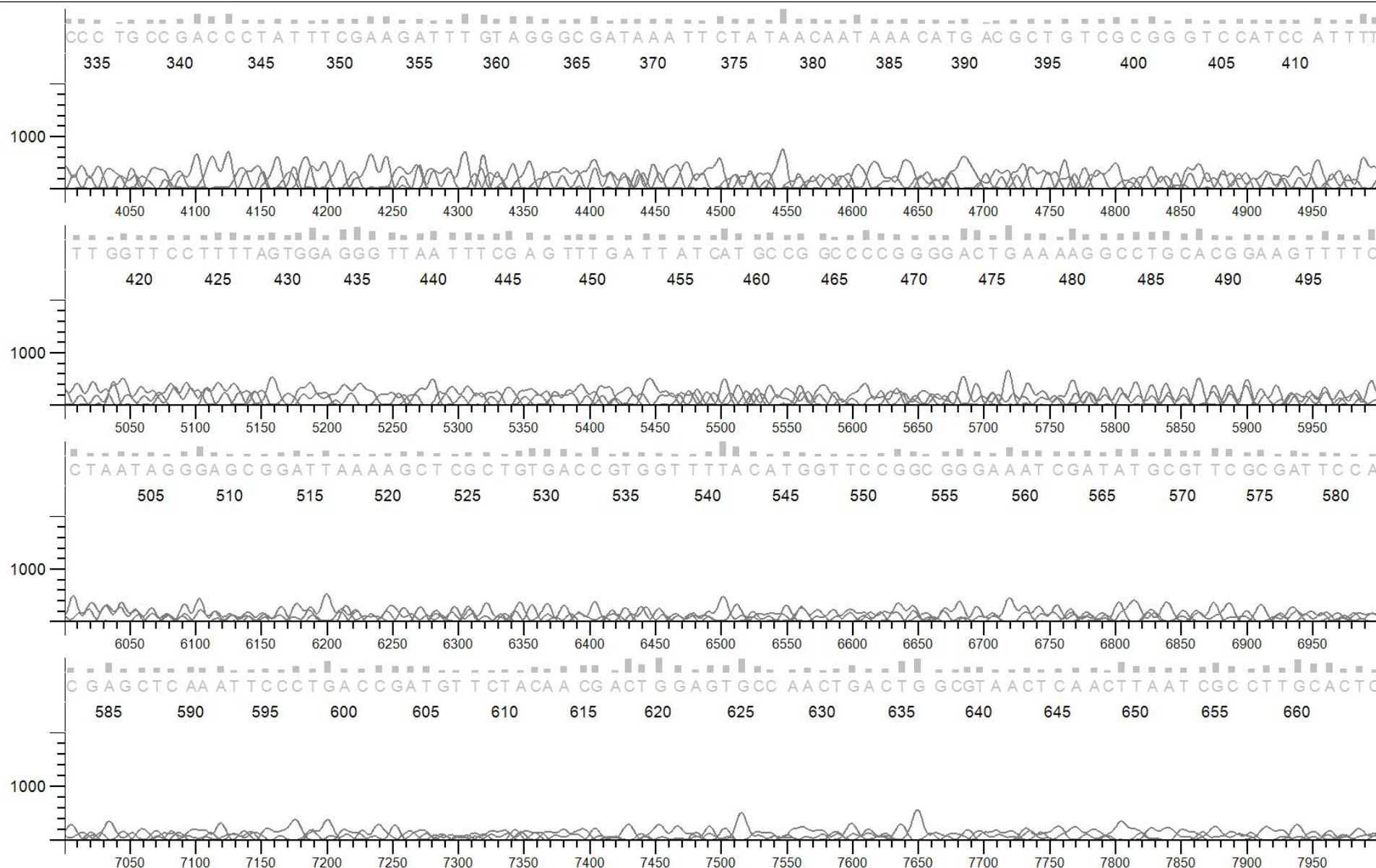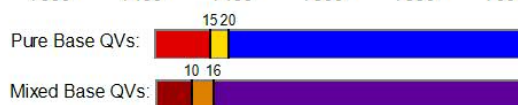

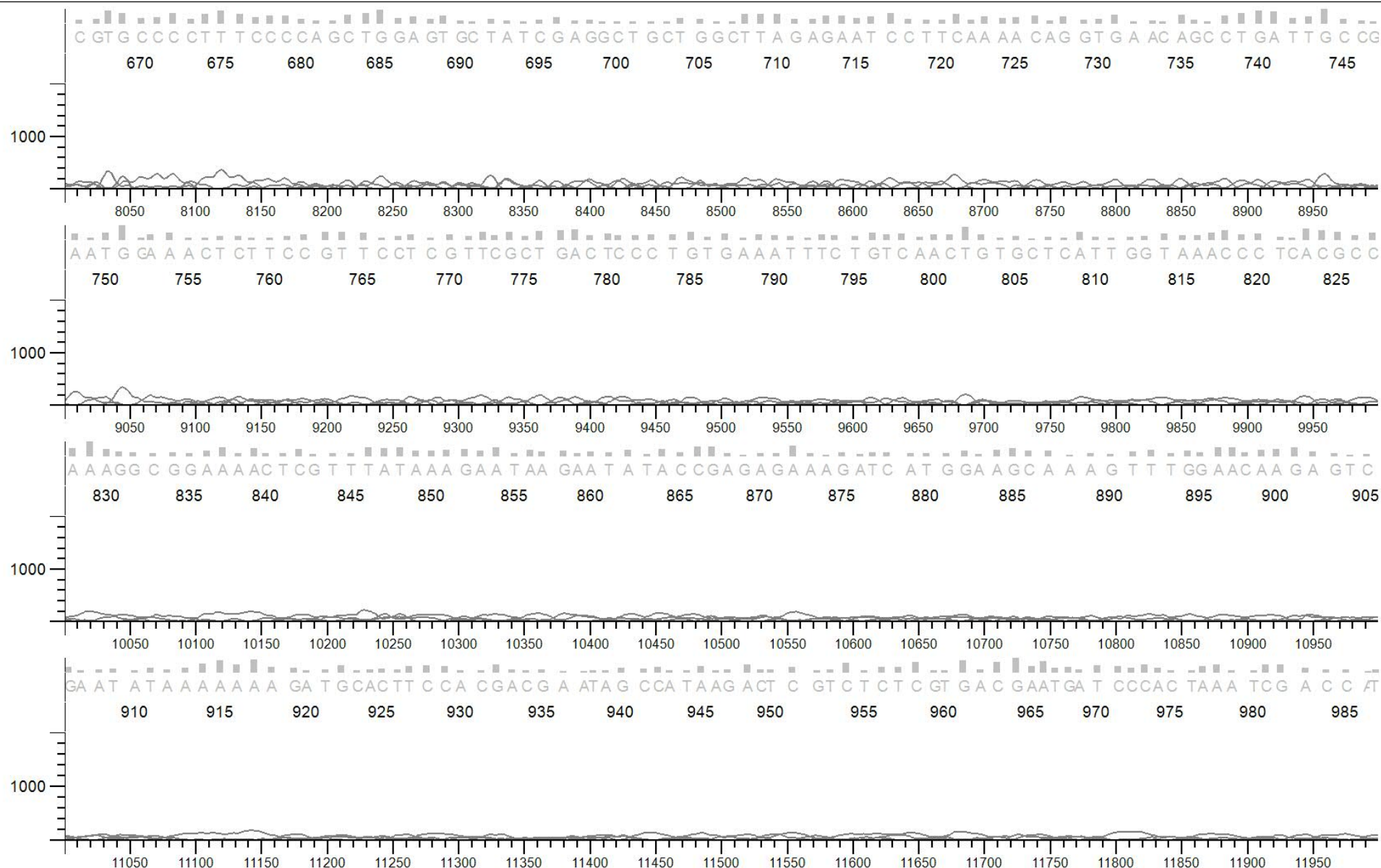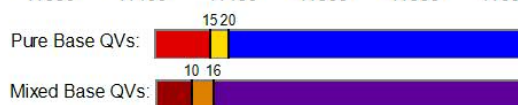

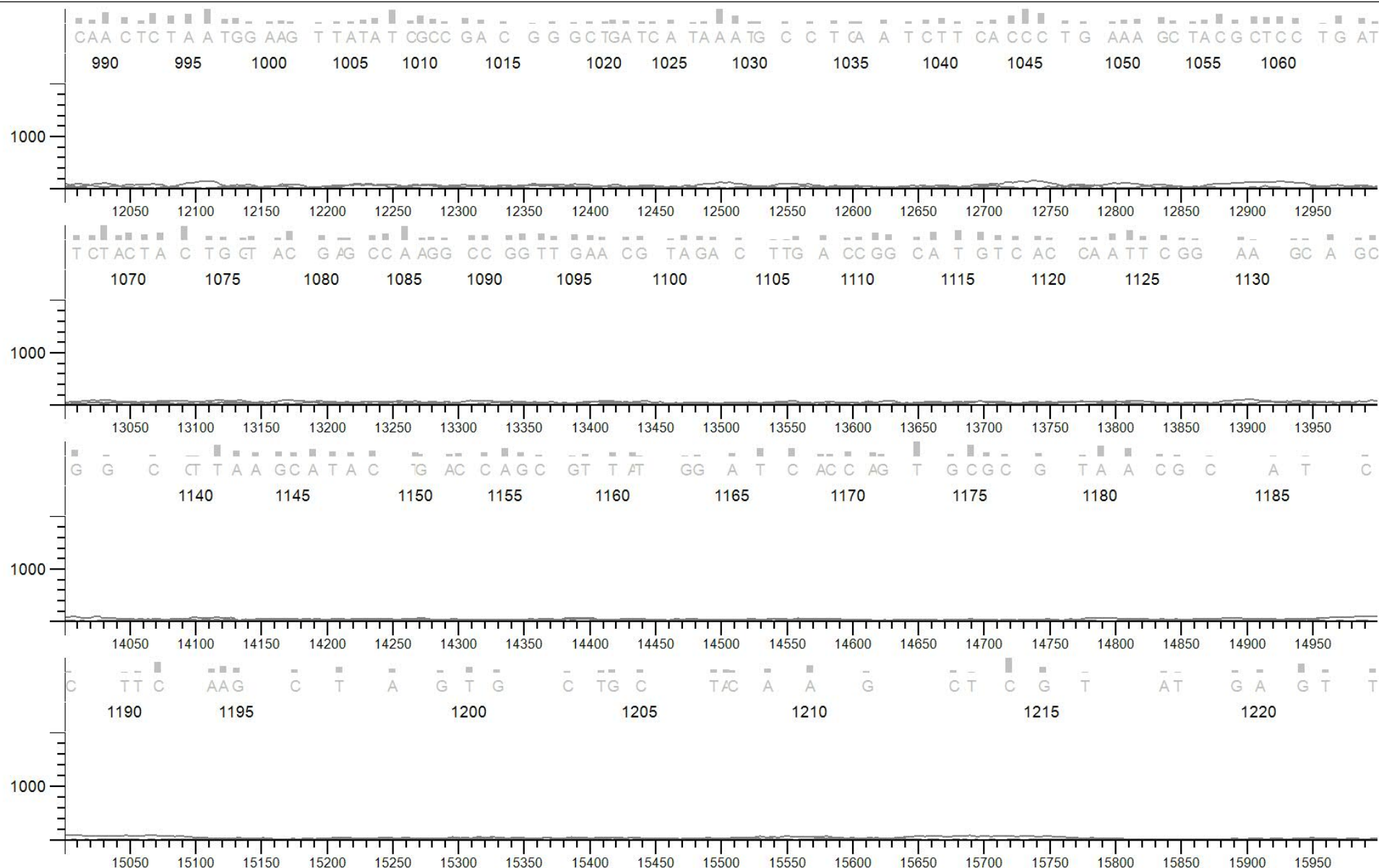

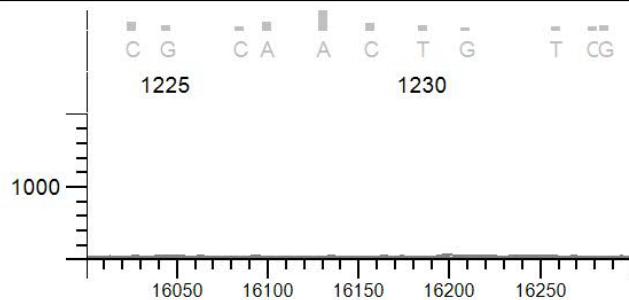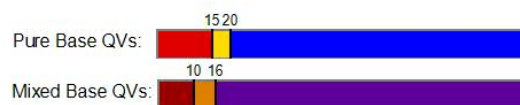

Supplement: Figure 3—source data 2. [file elife-69916-fig3-data2.zip › Figure 3B.C_Source data3_Bisulphite sequencing_mtDNA/SD_MTDNA_BSF_1.18_T7FOR-E05.pdf]

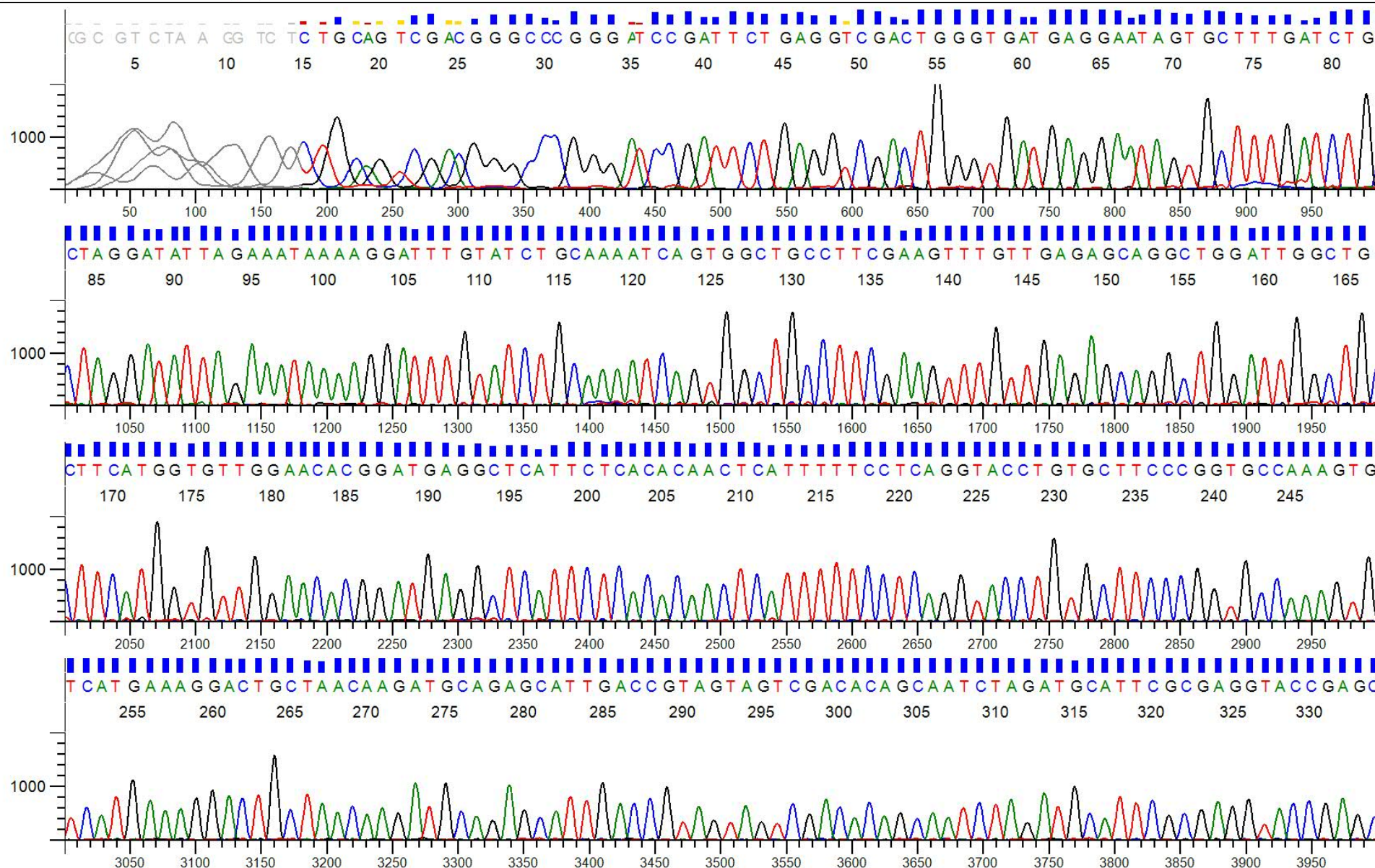

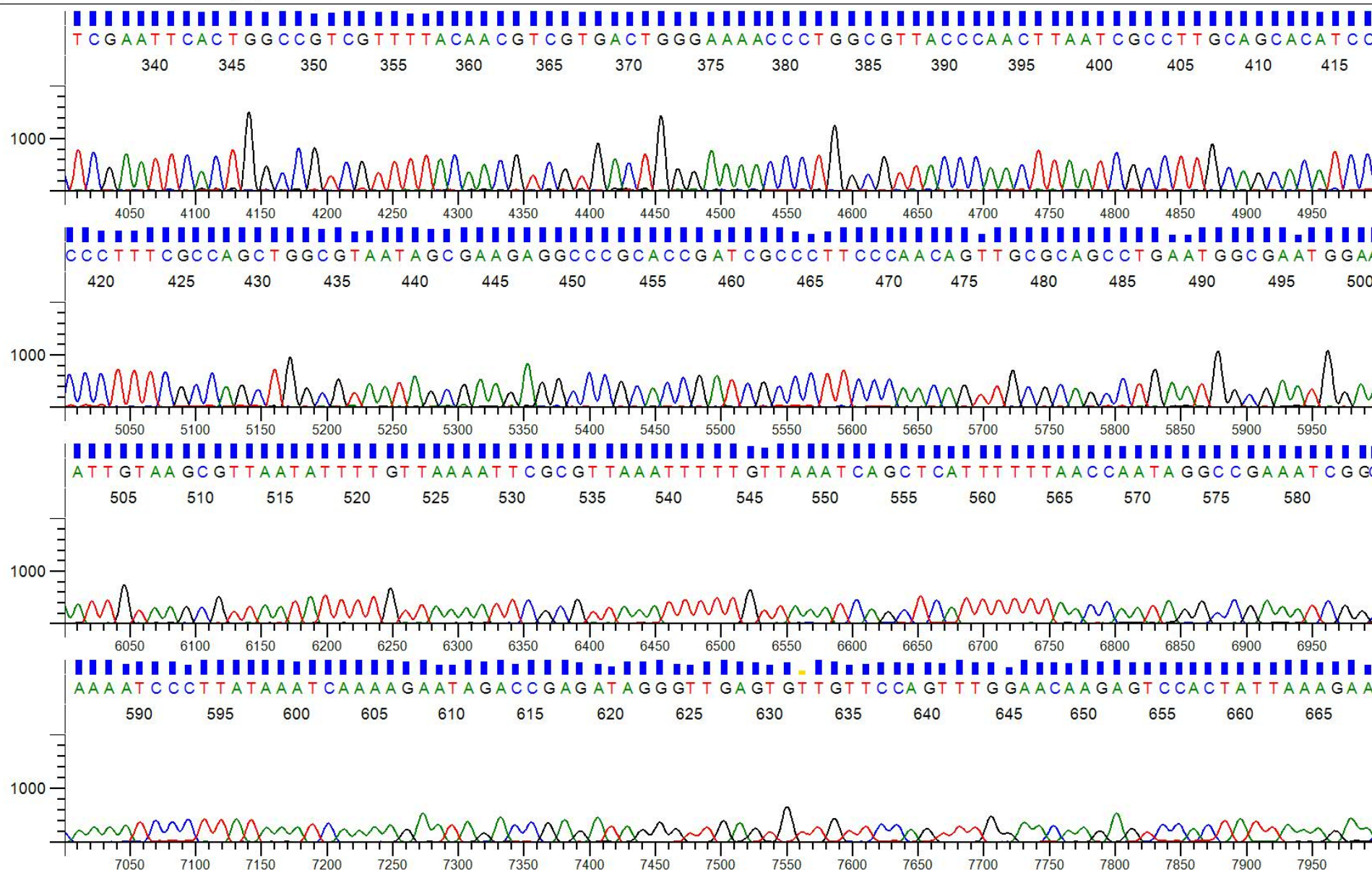

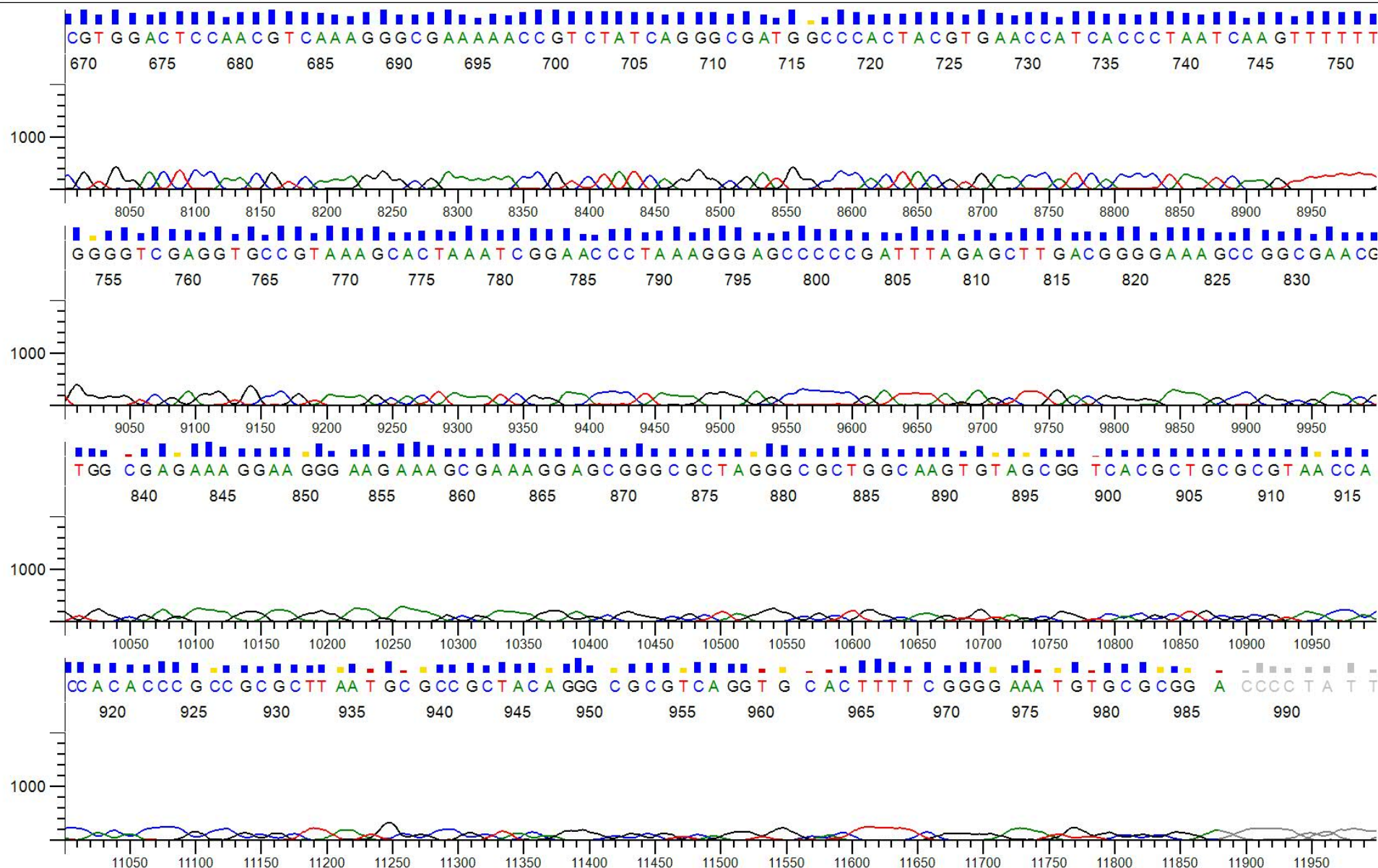

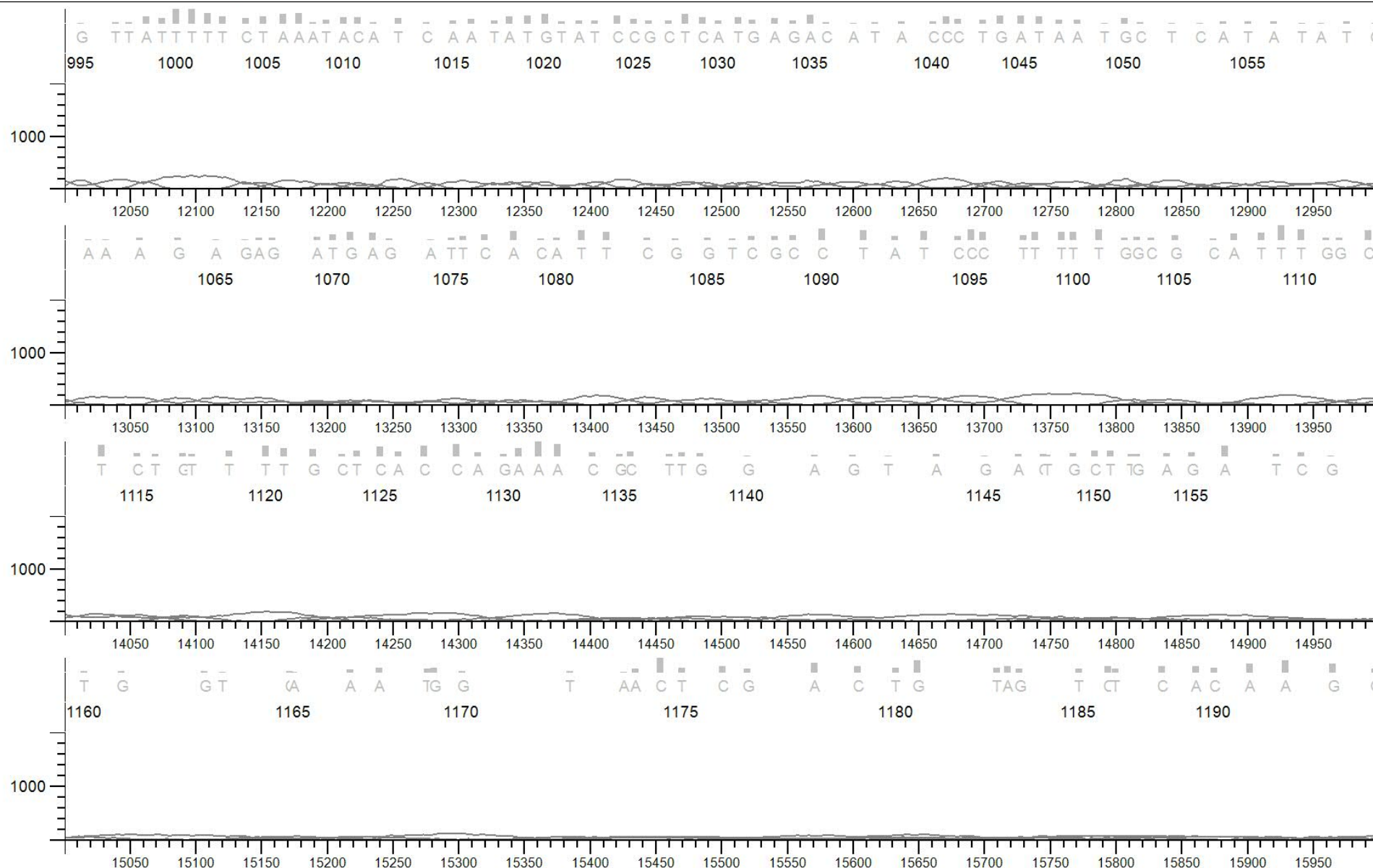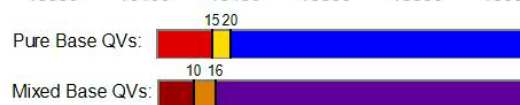

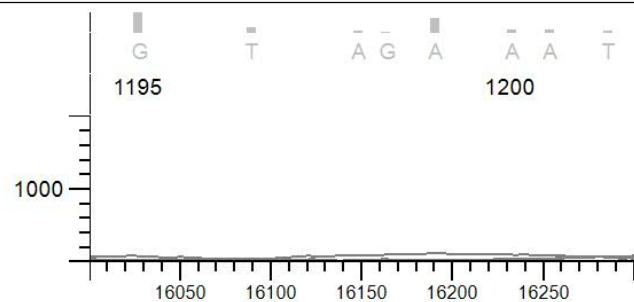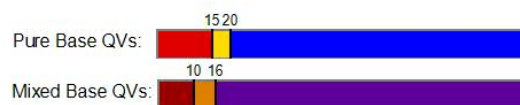

Supplement: Figure 3—source data 2. [file elife-69916-fig3-data2.zip › Figure 3B.C_Source data3_Bisulphite sequencing_mtDNA/SS4-MT-BIS-2.25_T7FOR.pdf]

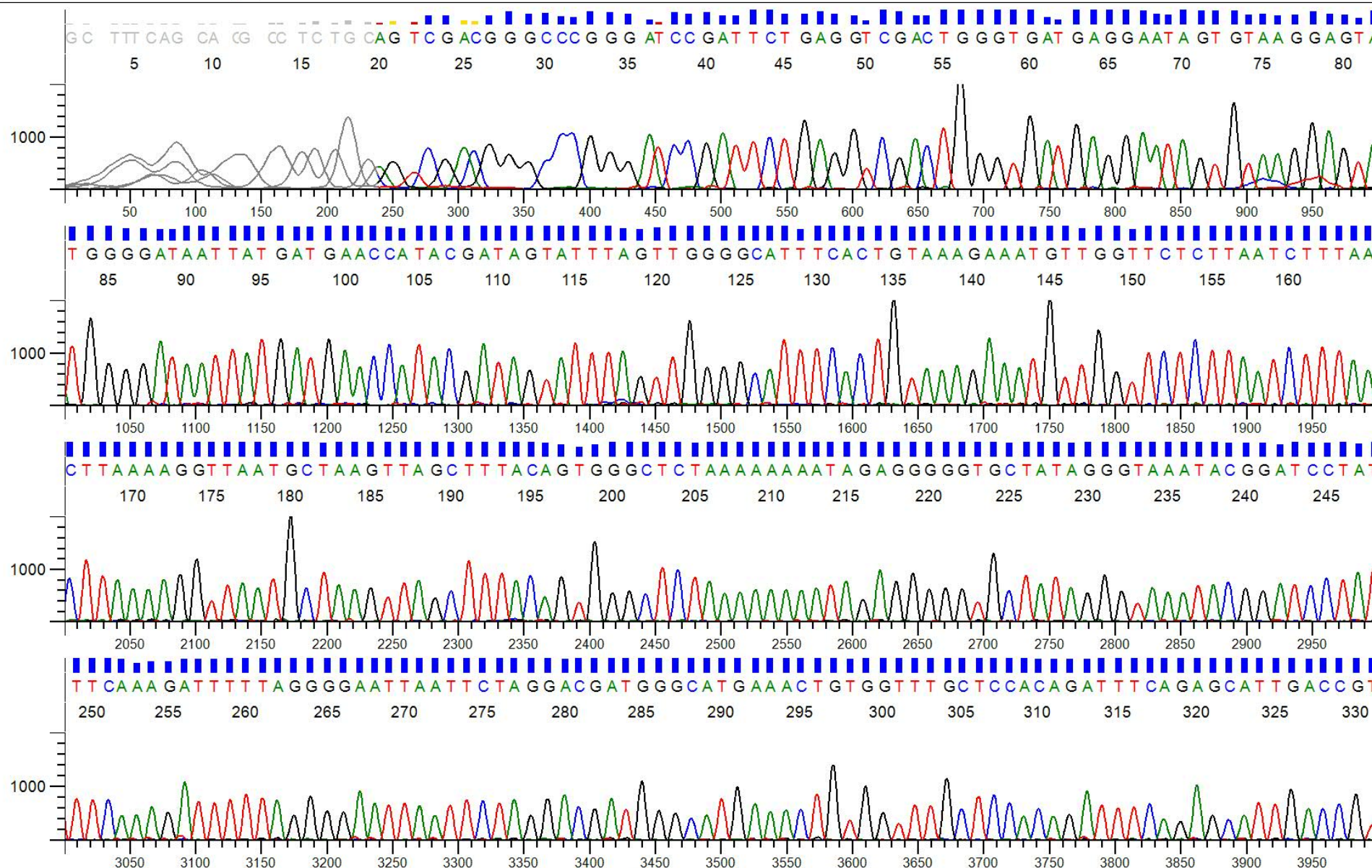

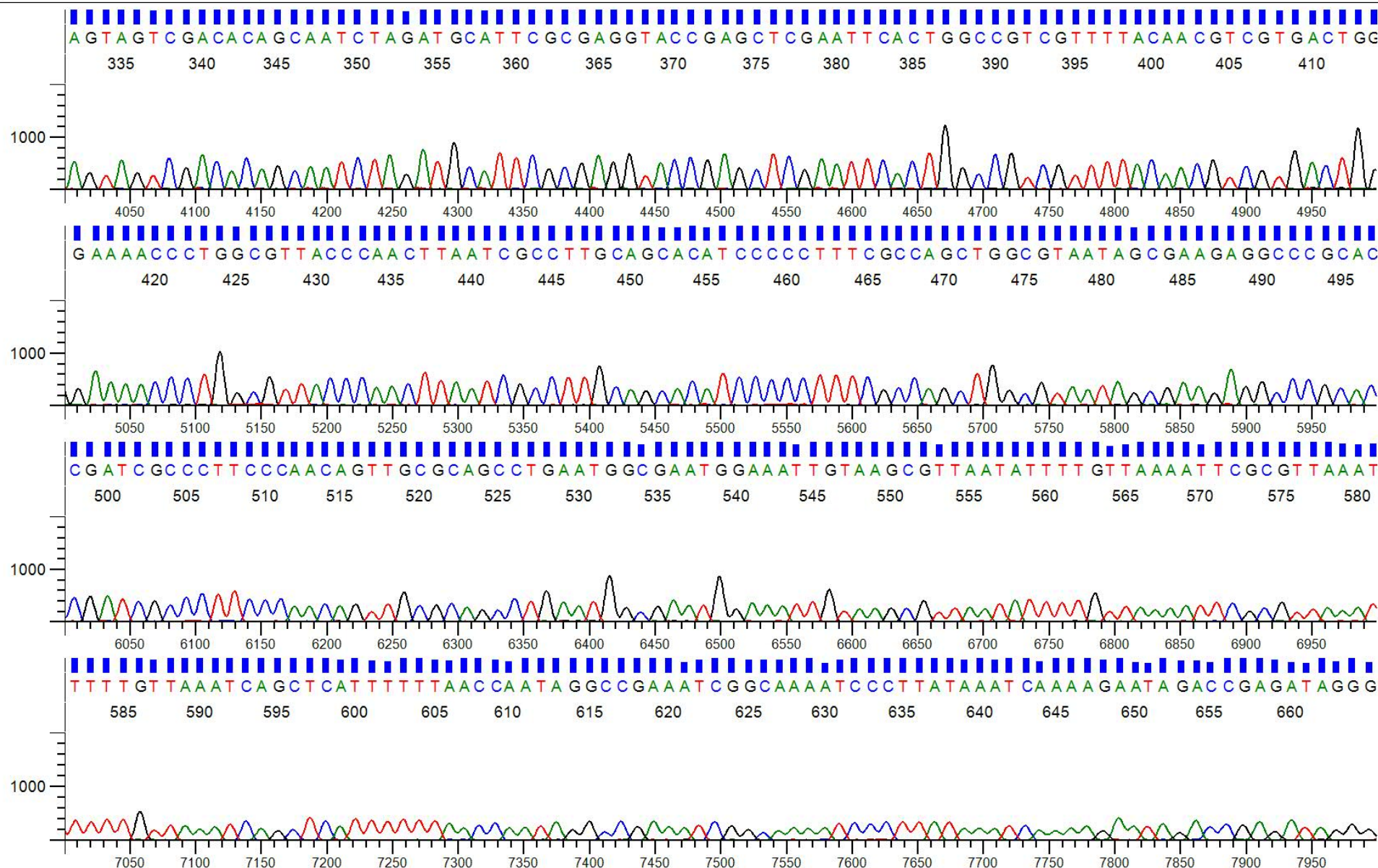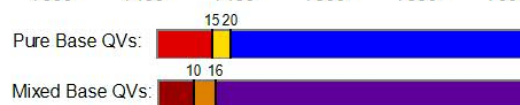

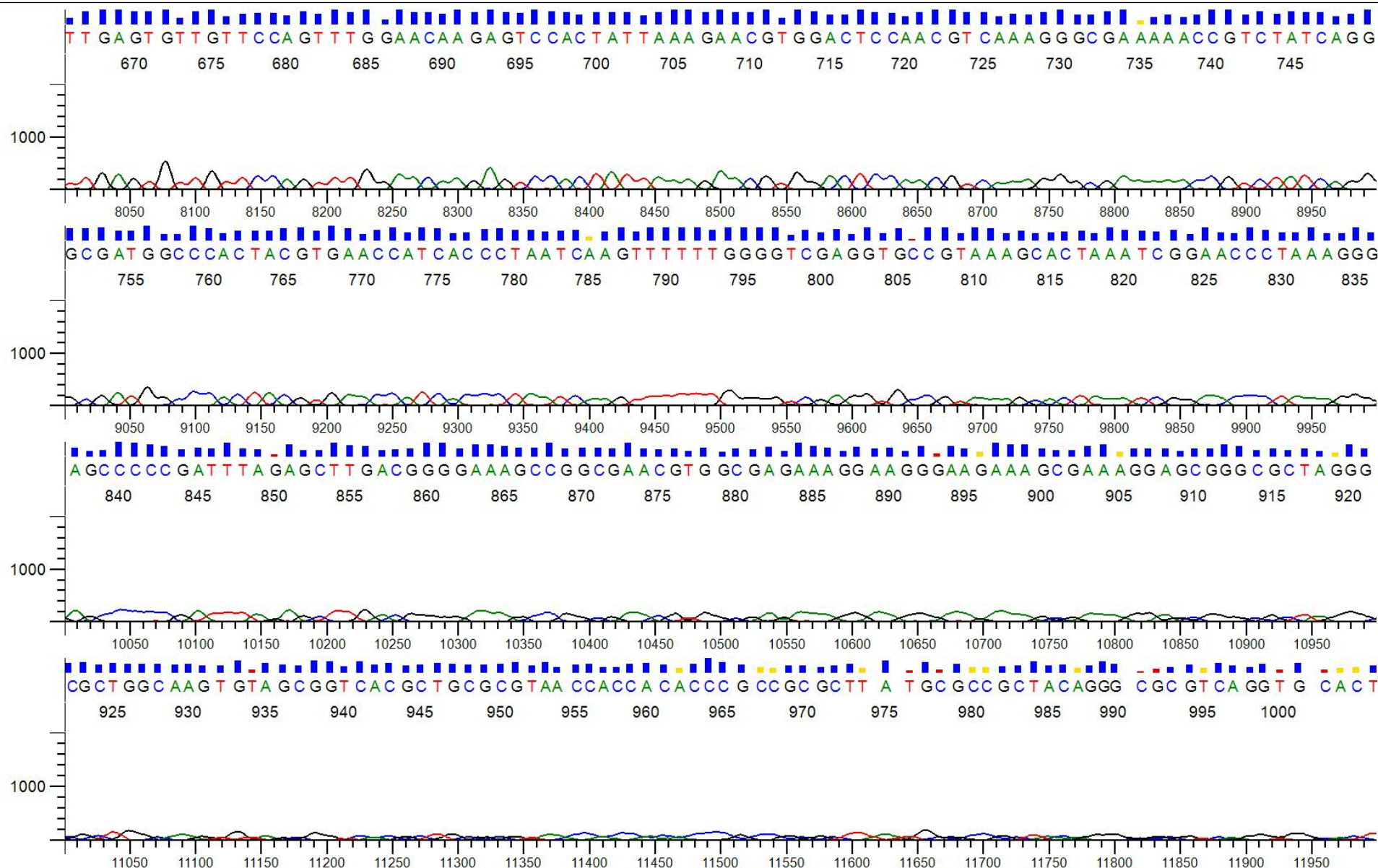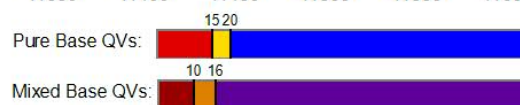

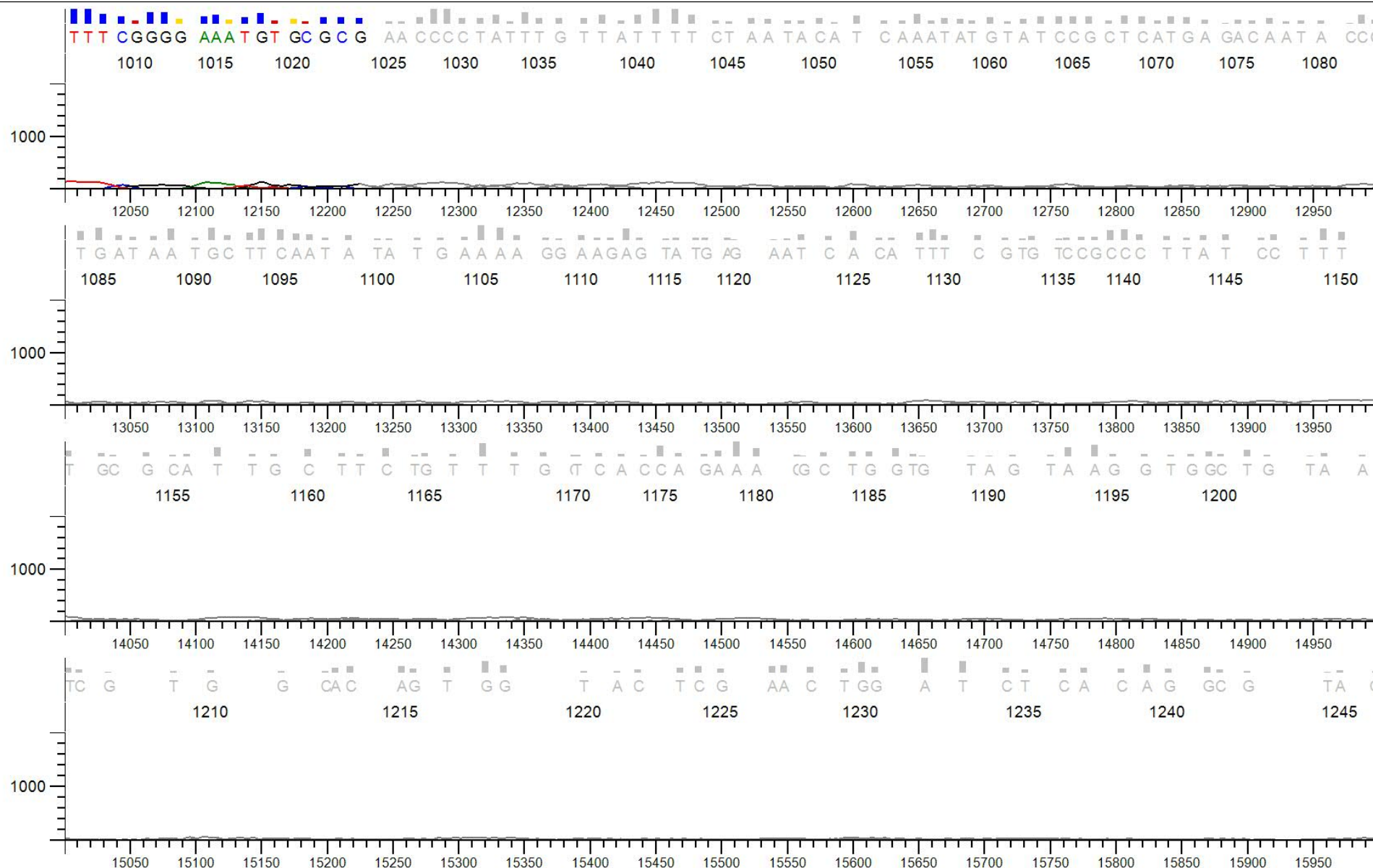

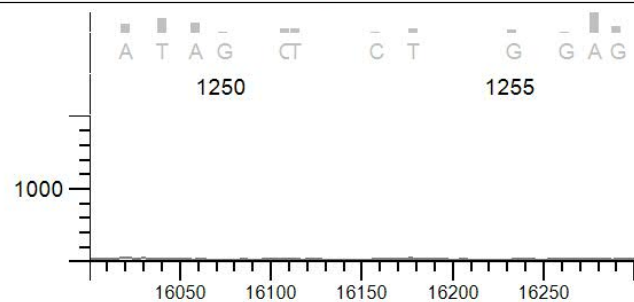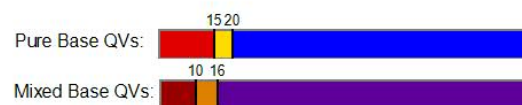

Supplement: Figure 3—source data 2. [file elife-69916-fig3-data2.zip › Figure 3B.C_Source data3_Bisulphite sequencing_mtDNA/SD_MTDNA_BSF_1.9_T7FOR-C02.pdf]

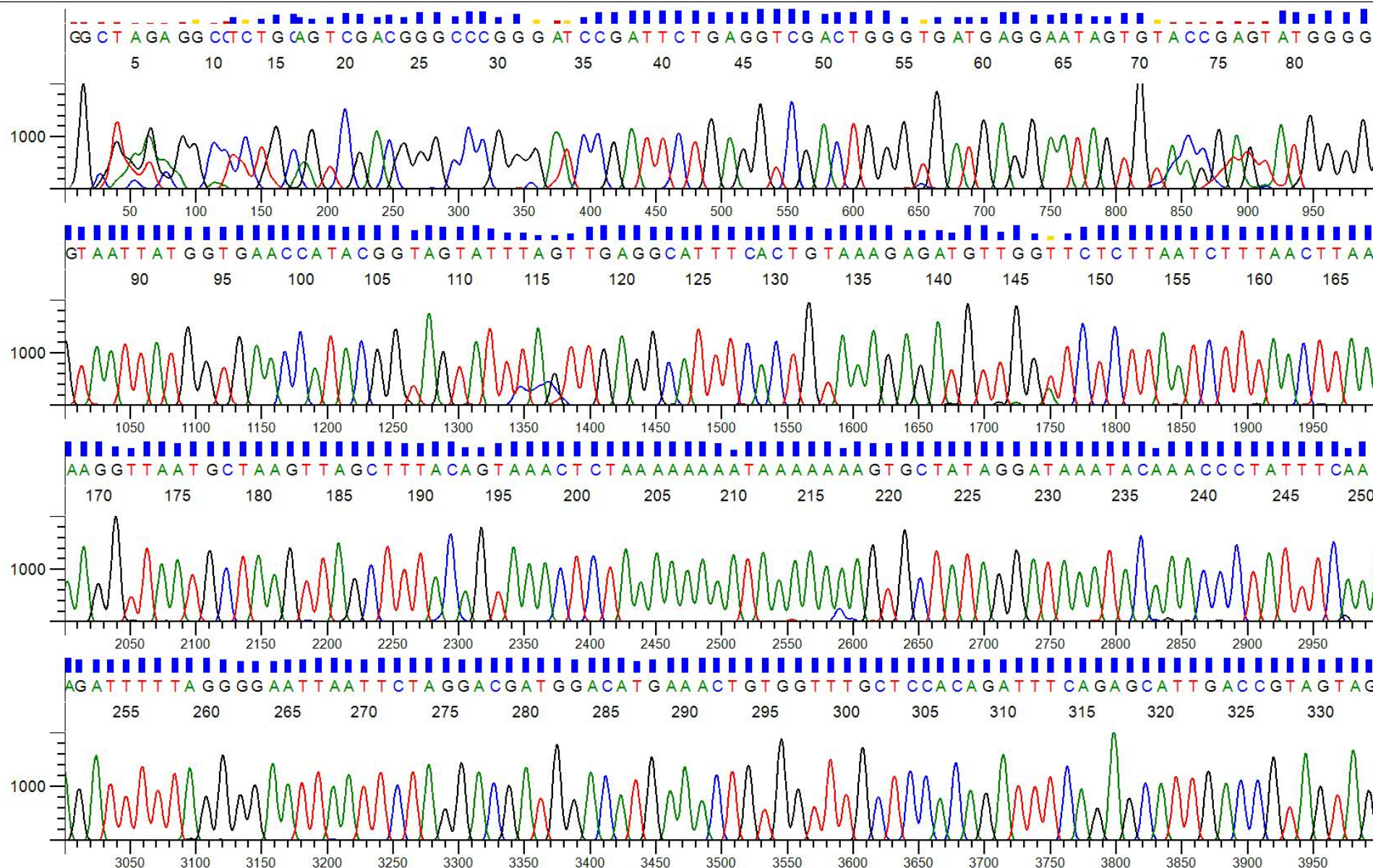

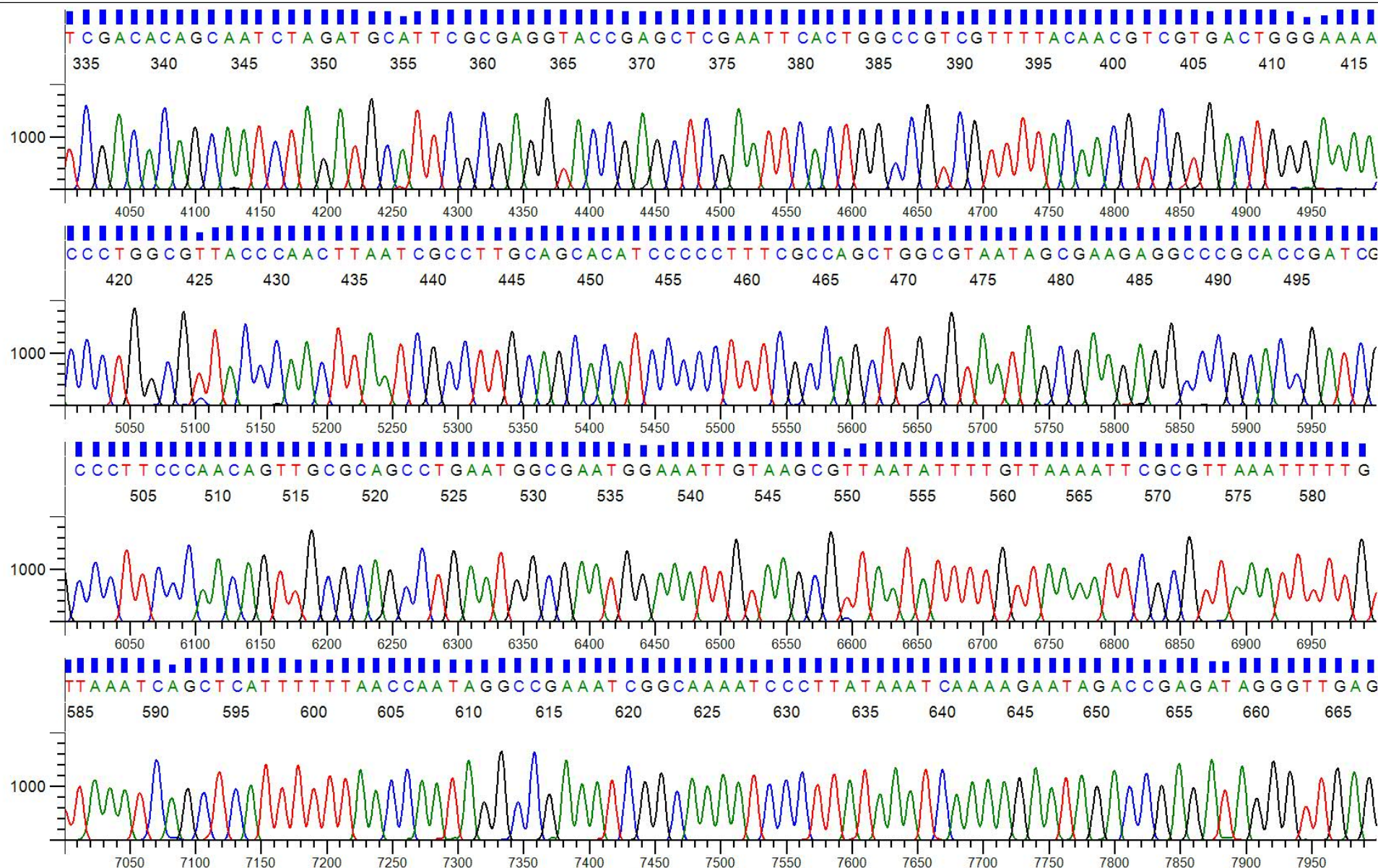

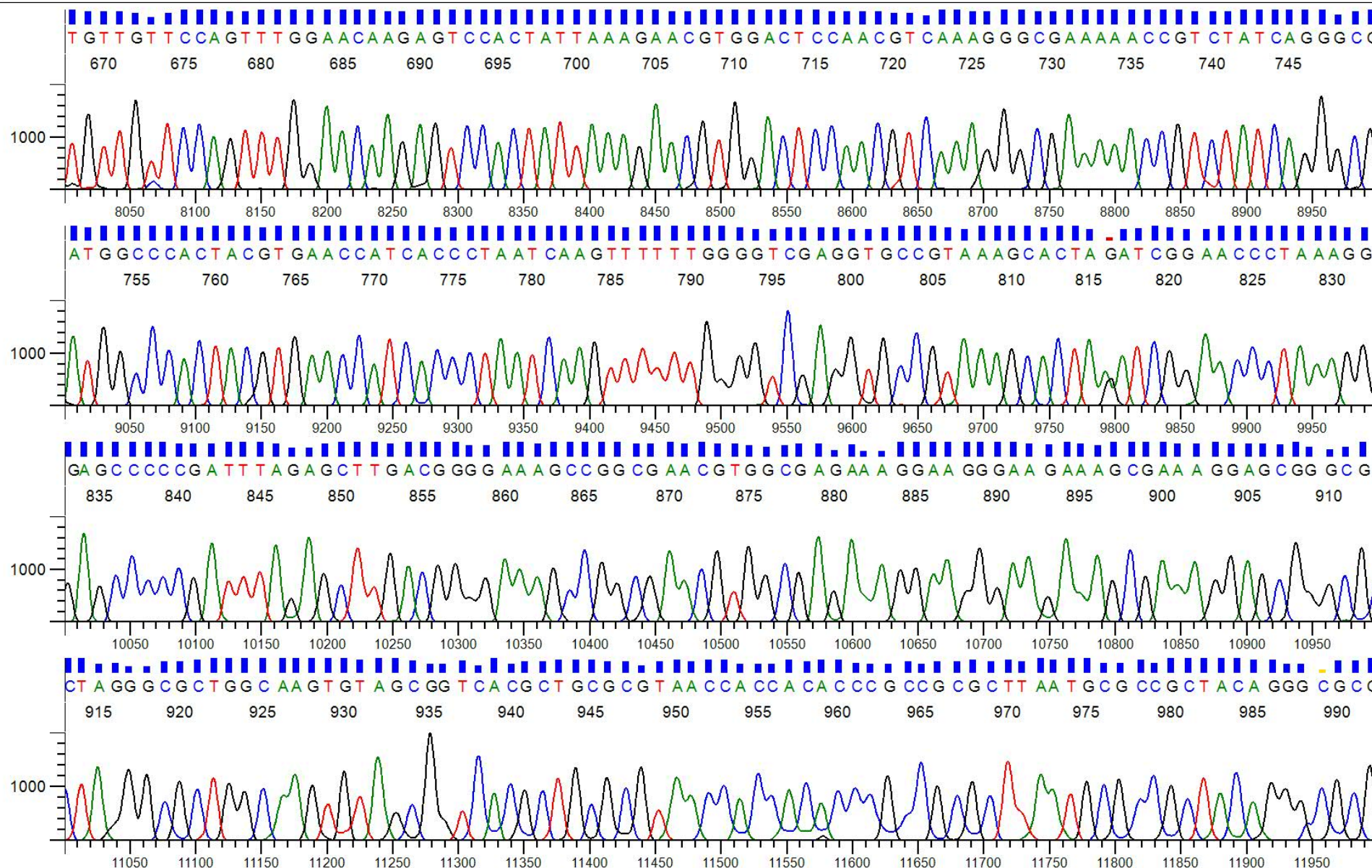

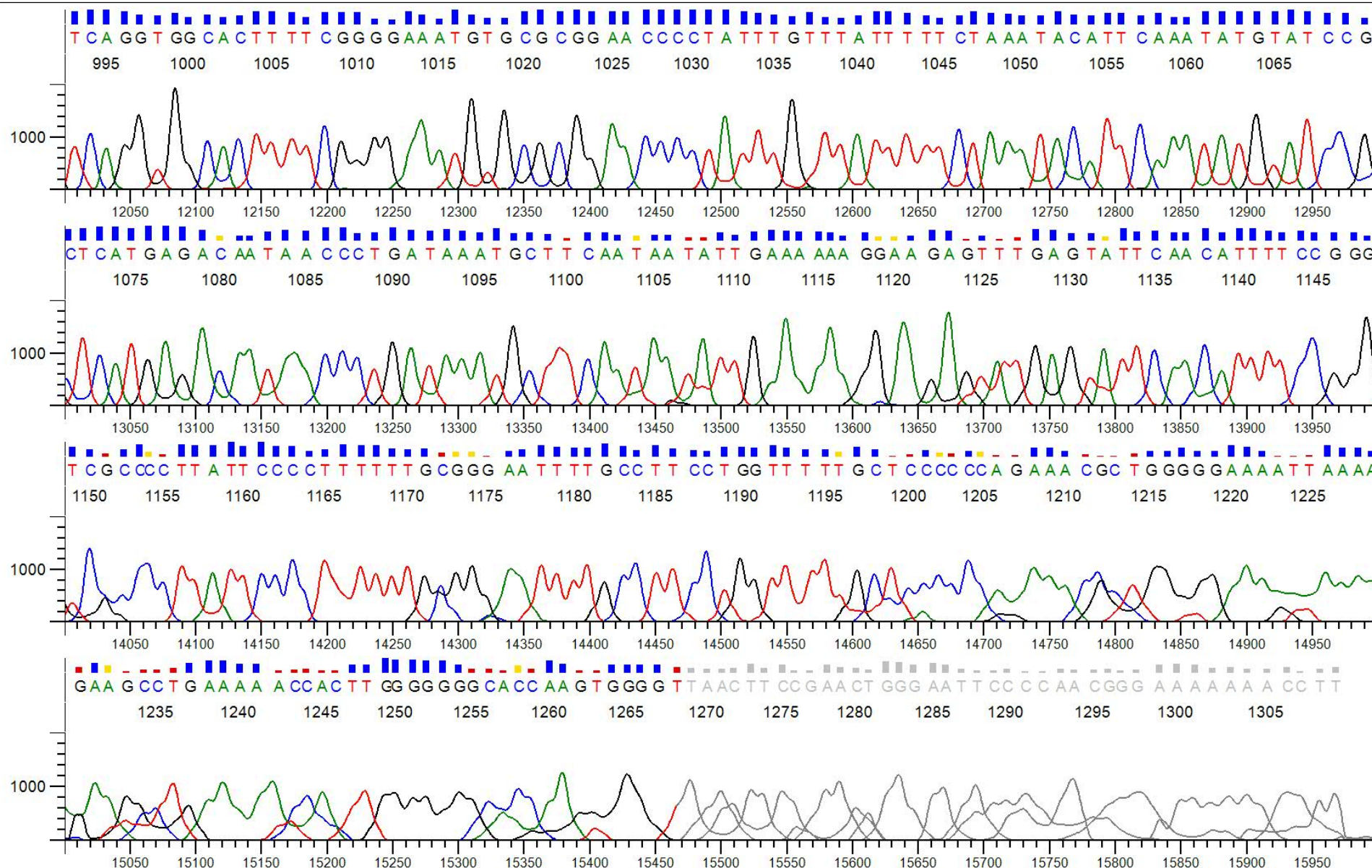

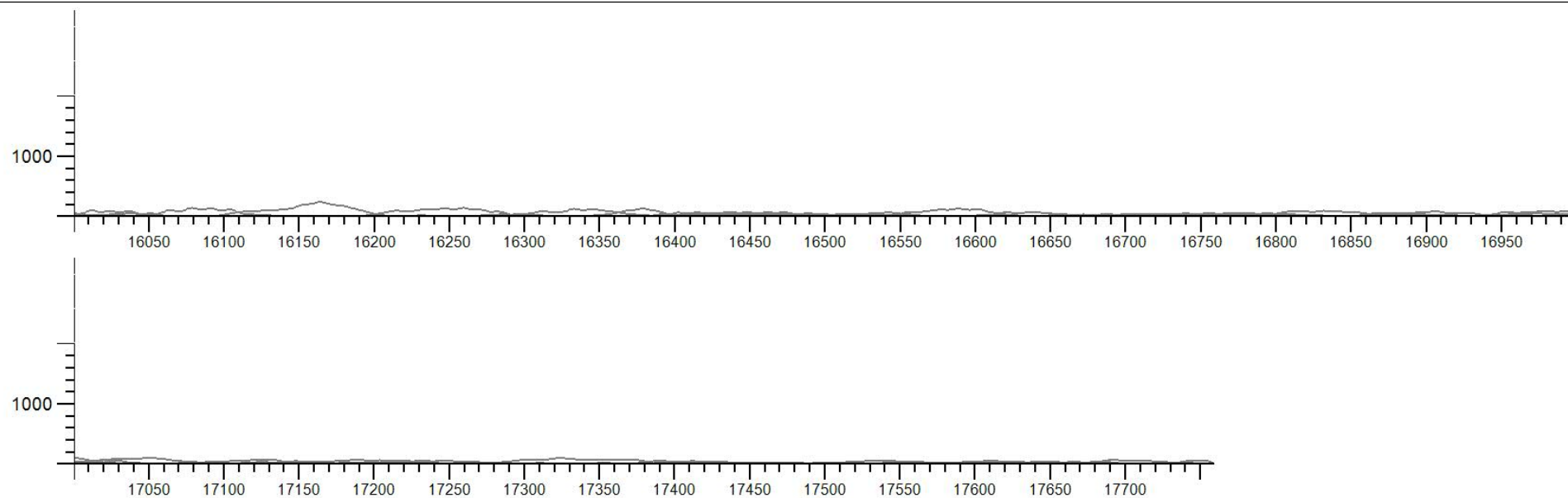

Supplement: Figure 3—source data 2. [file elife-69916-fig3-data2.zip › Figure 3B.C_Source data3_Bisulphite sequencing_mtDNA/SS4-MT-BIS-44_T7FOR-F07.pdf]

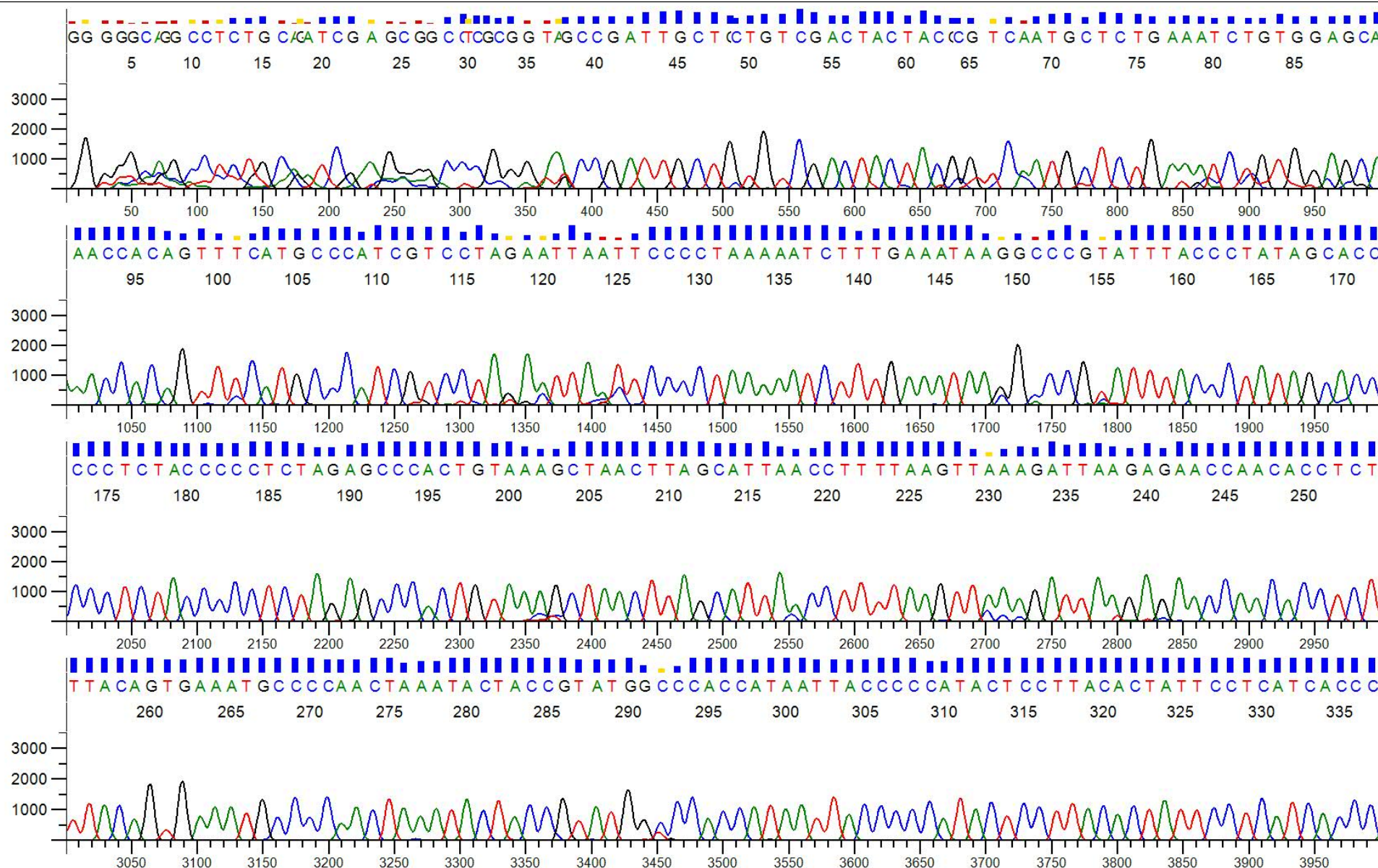

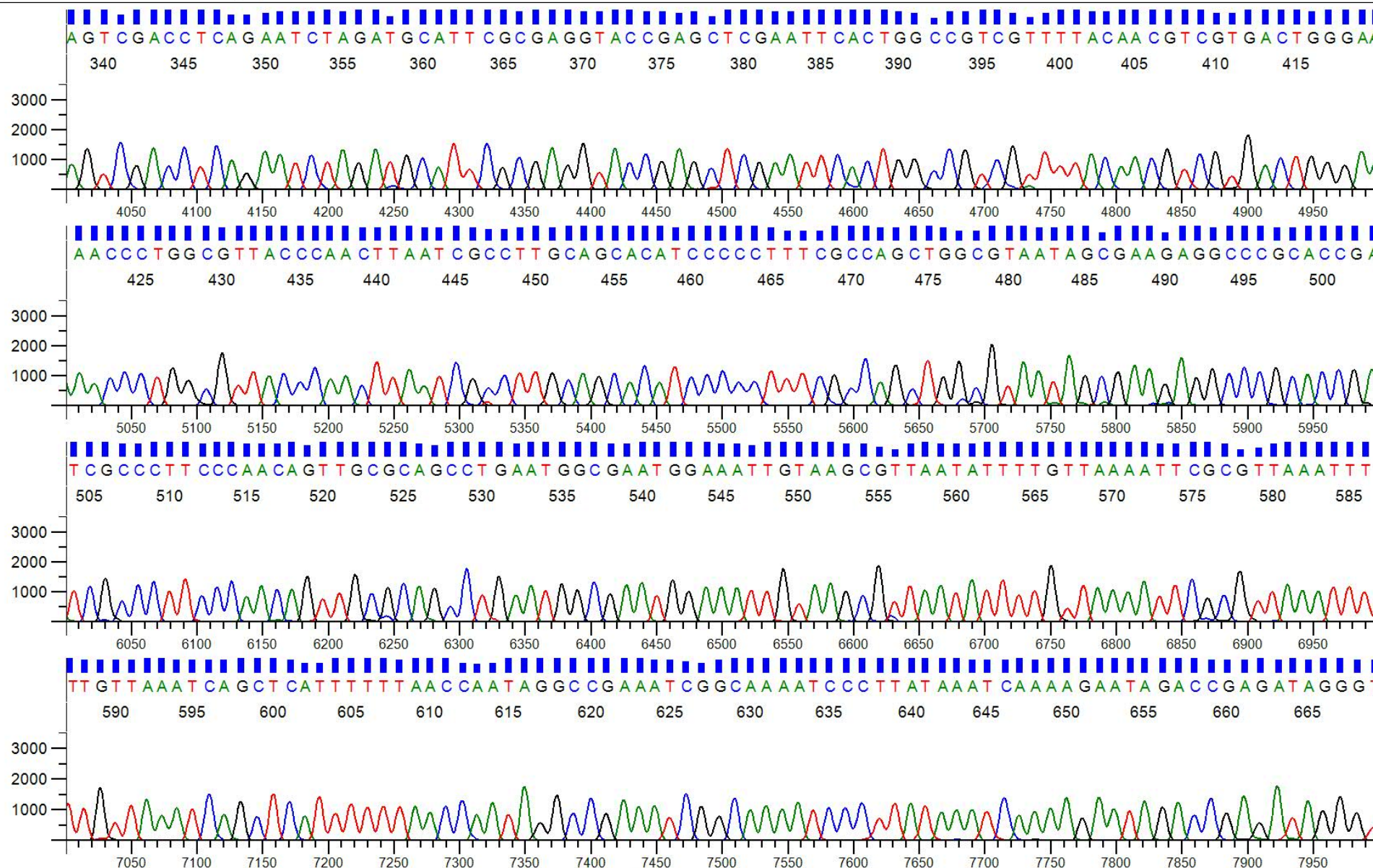

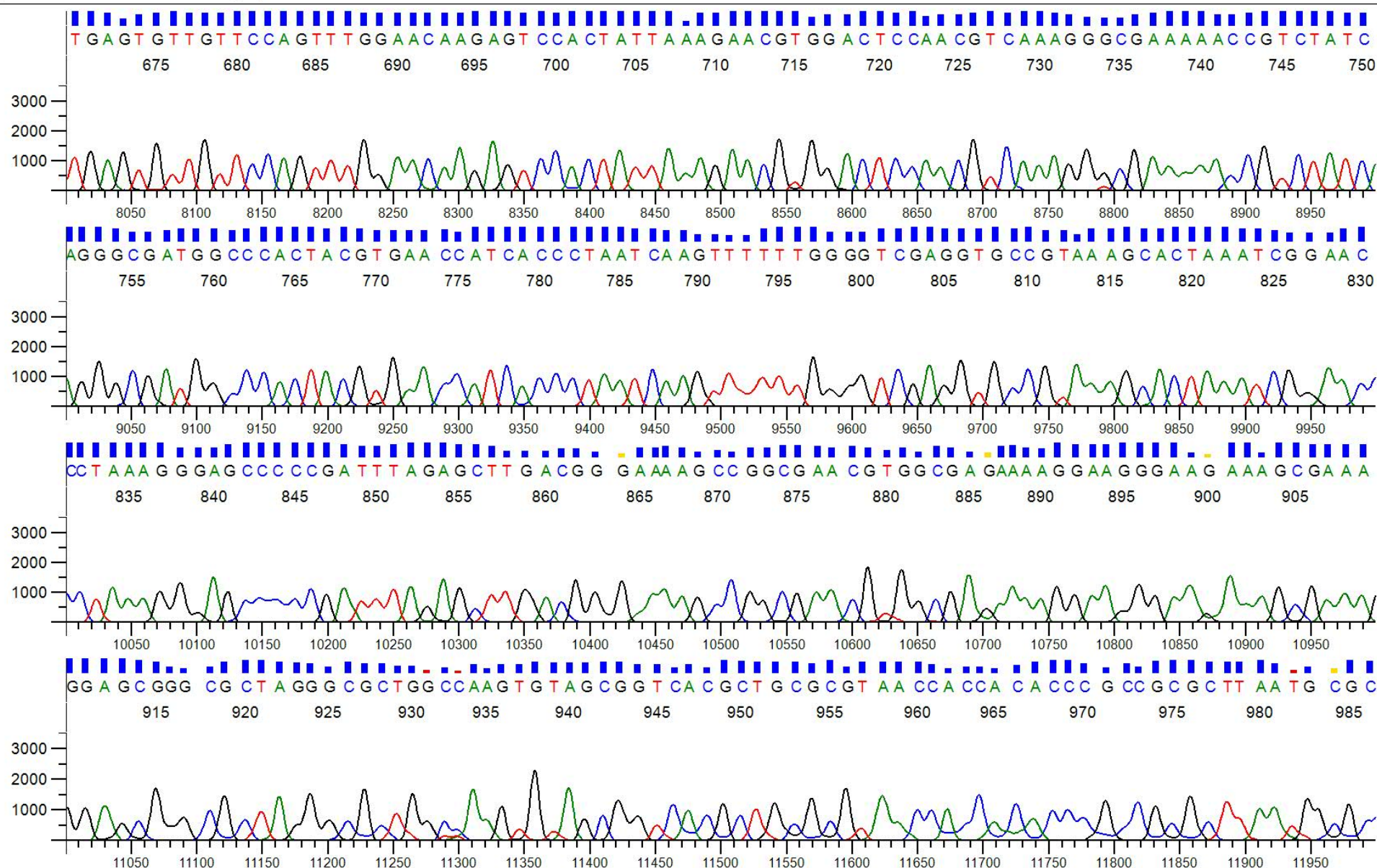

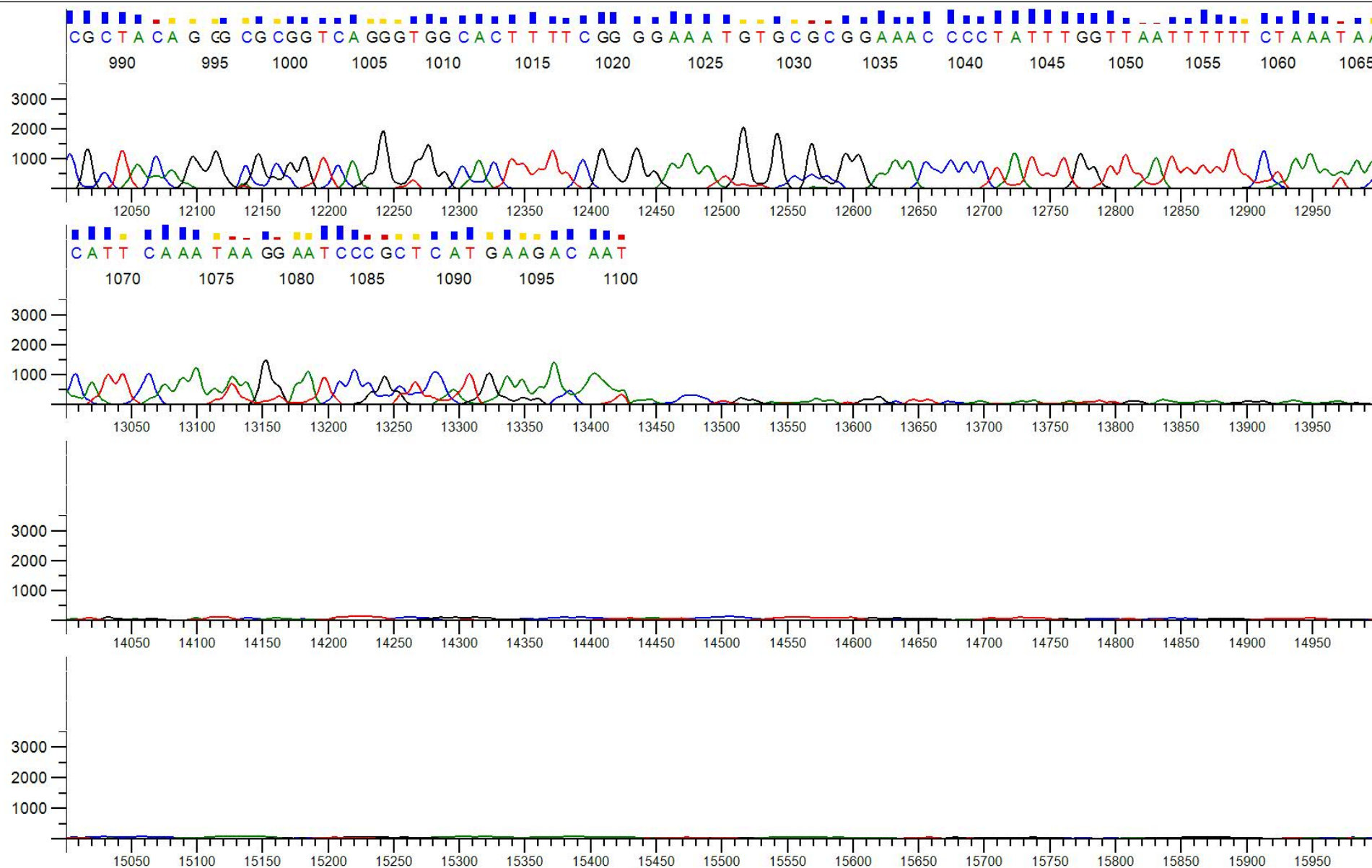

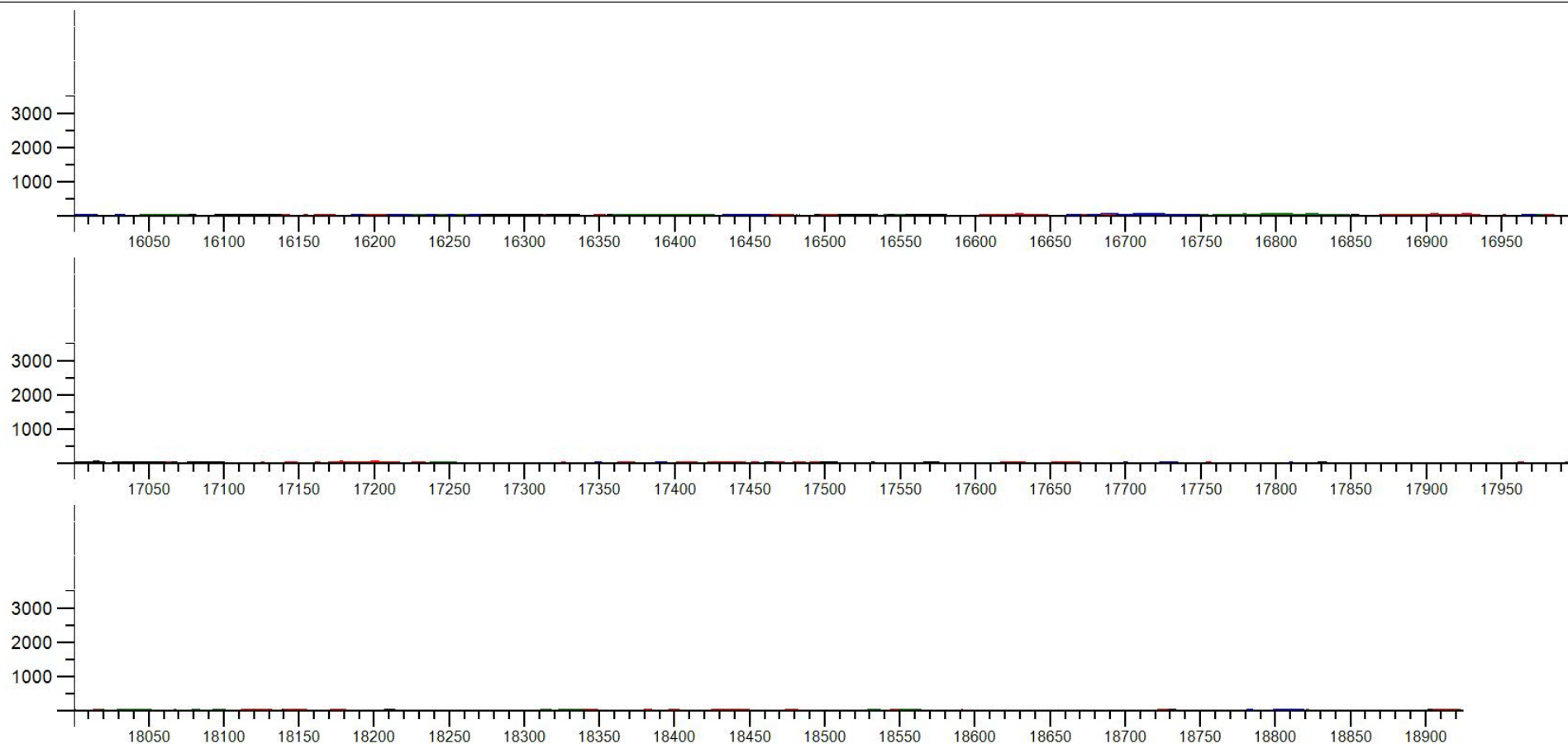

Supplement: Figure 3—source data 2. [file elife-69916-fig3-data2.zip › Figure 3B.C_Source data3_Bisulphite sequencing_mtDNA/SD-MTDNA-BSF-VKKII-1.3_T7-FOR-C04.pdf]

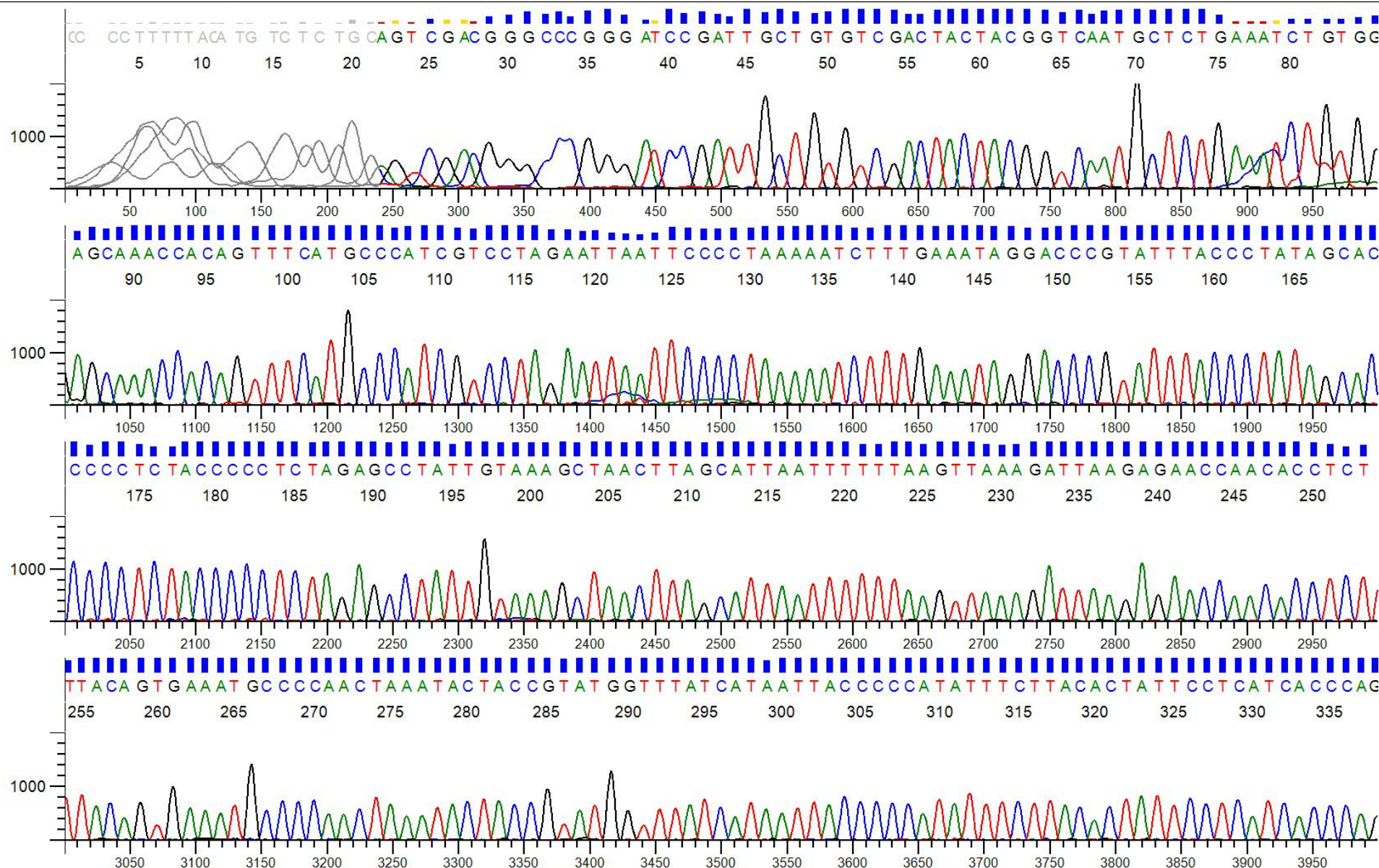

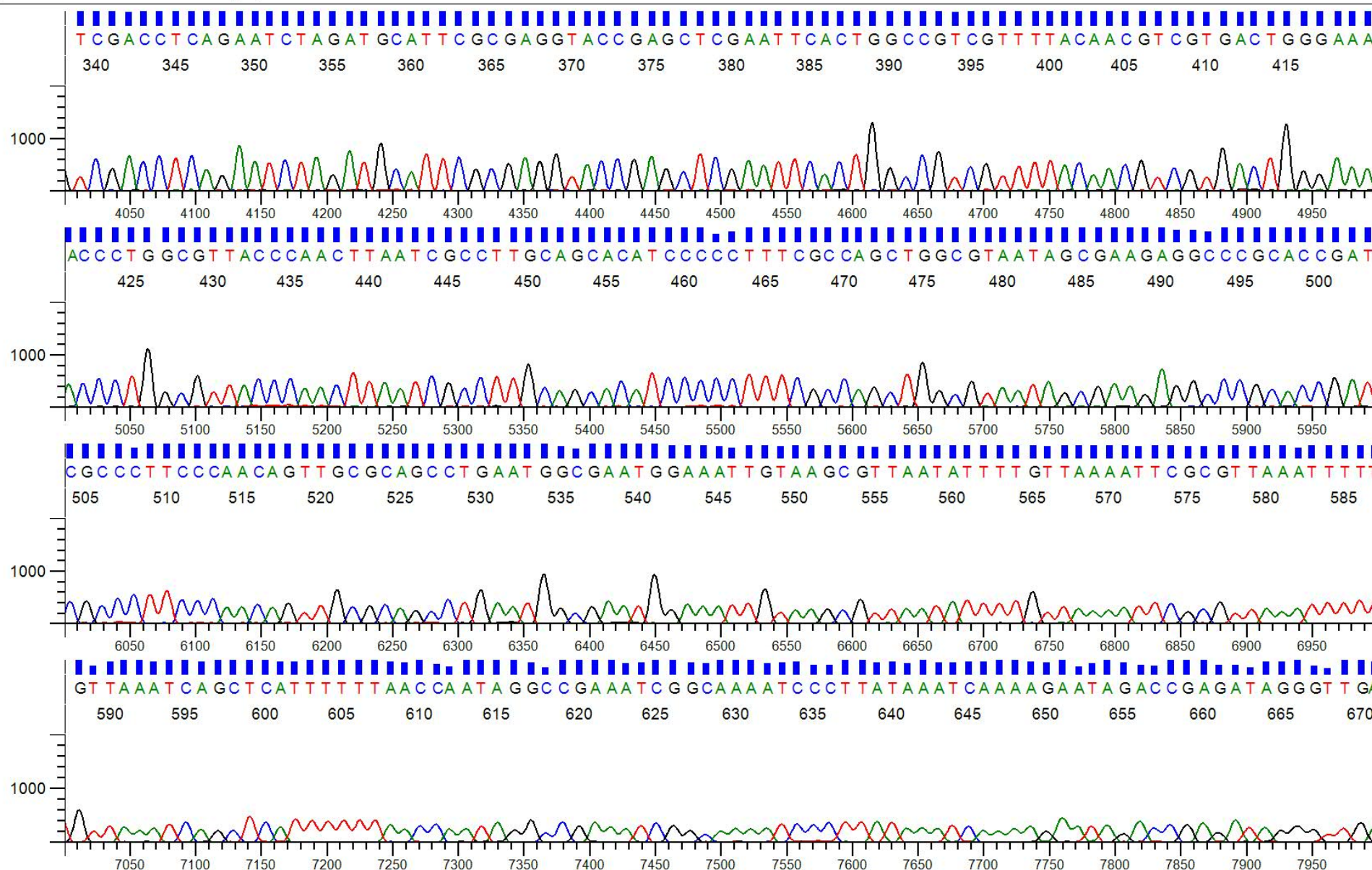

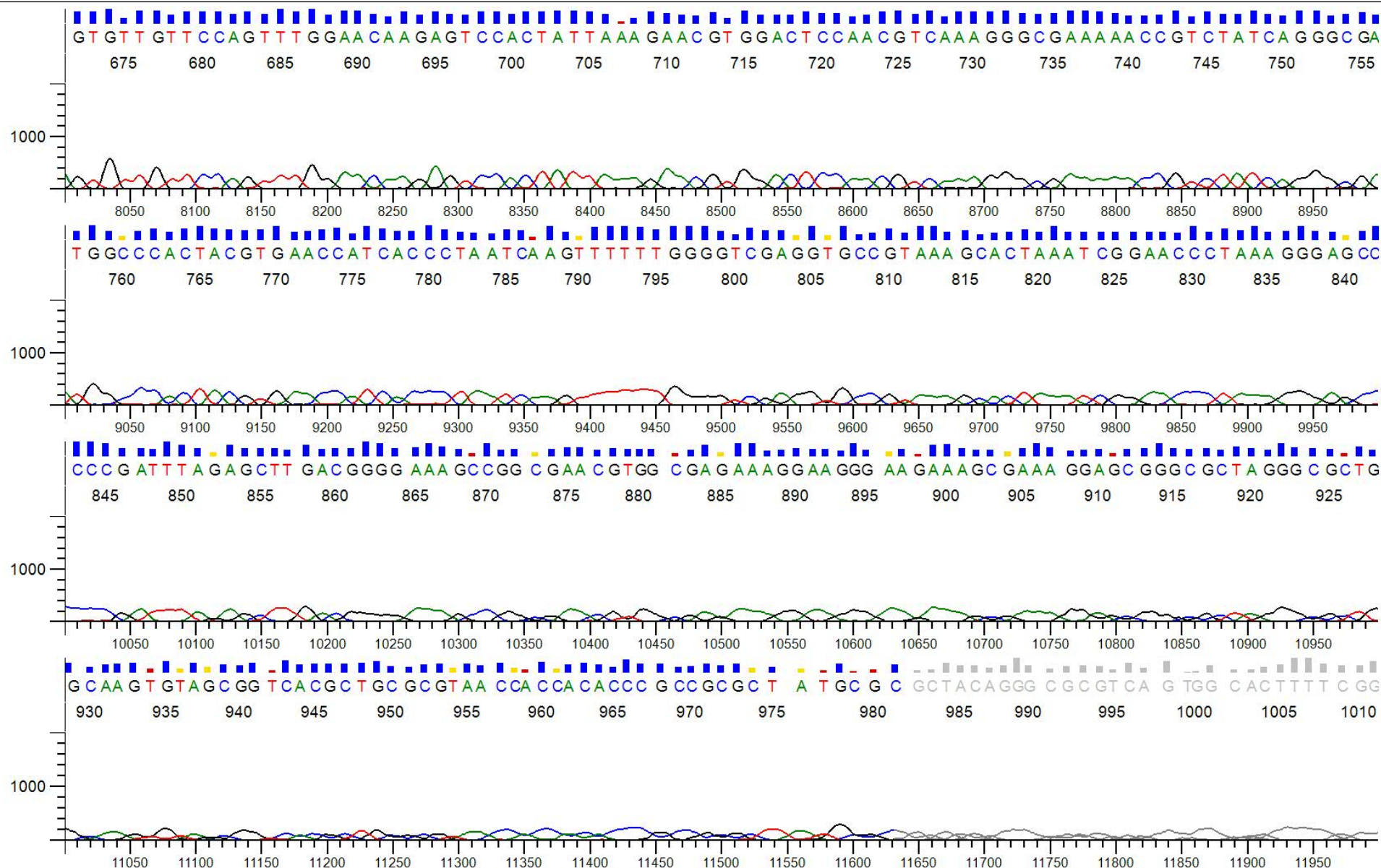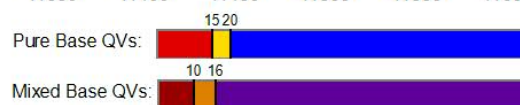

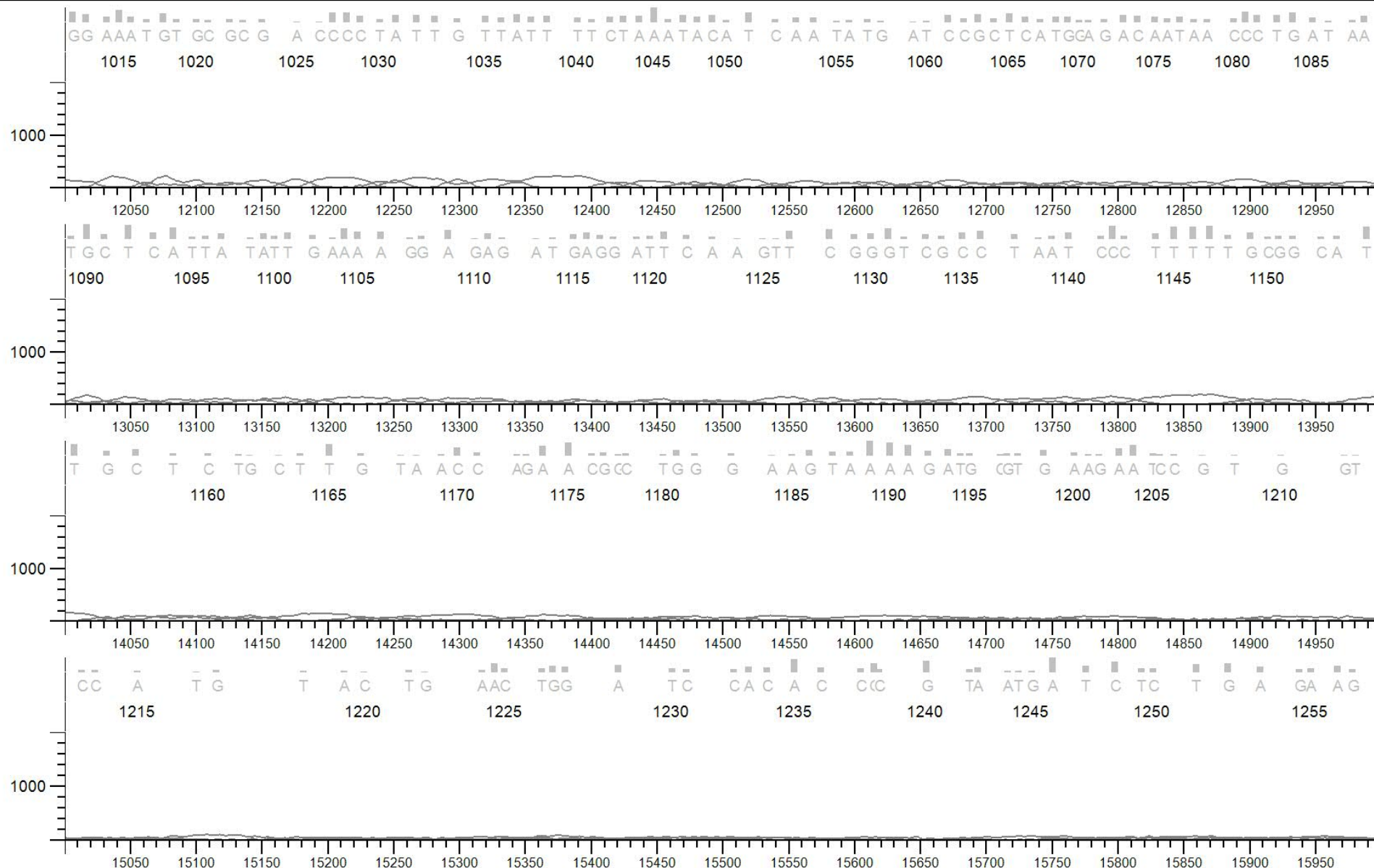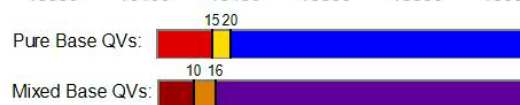

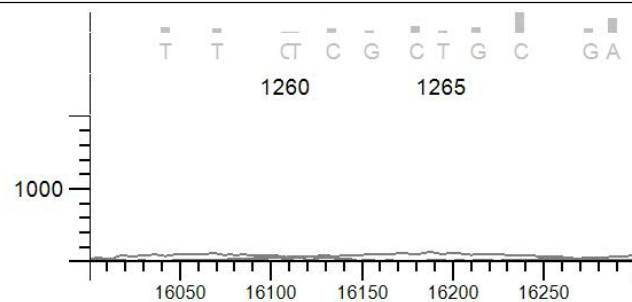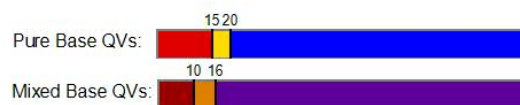

Supplement: Figure 3—source data 2. [file elife-69916-fig3-data2.zip › Figure 3B.C_Source data3_Bisulphite sequencing_mtDNA/SS4-MT-BIS-3_T7FOR-H06.pdf]

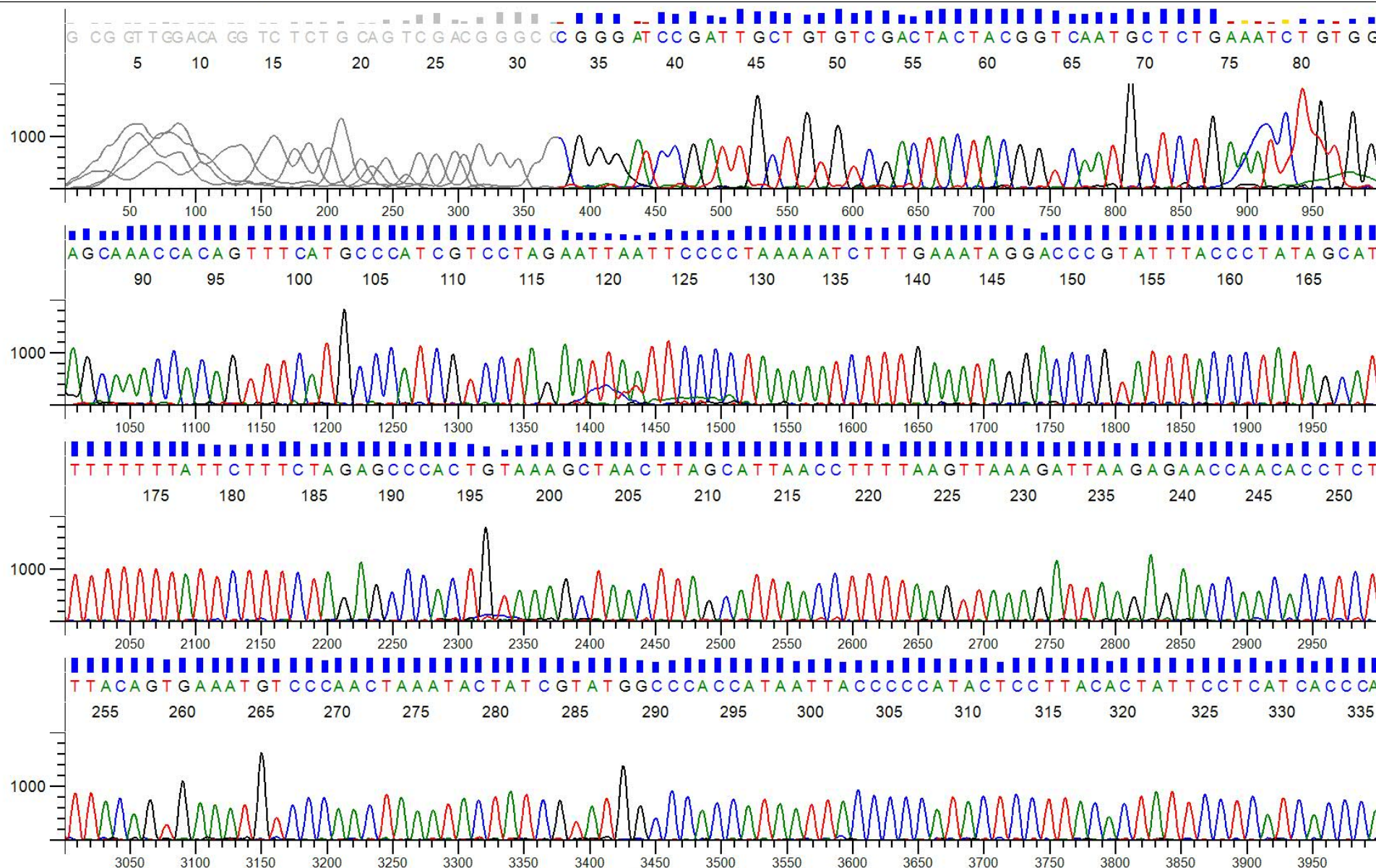

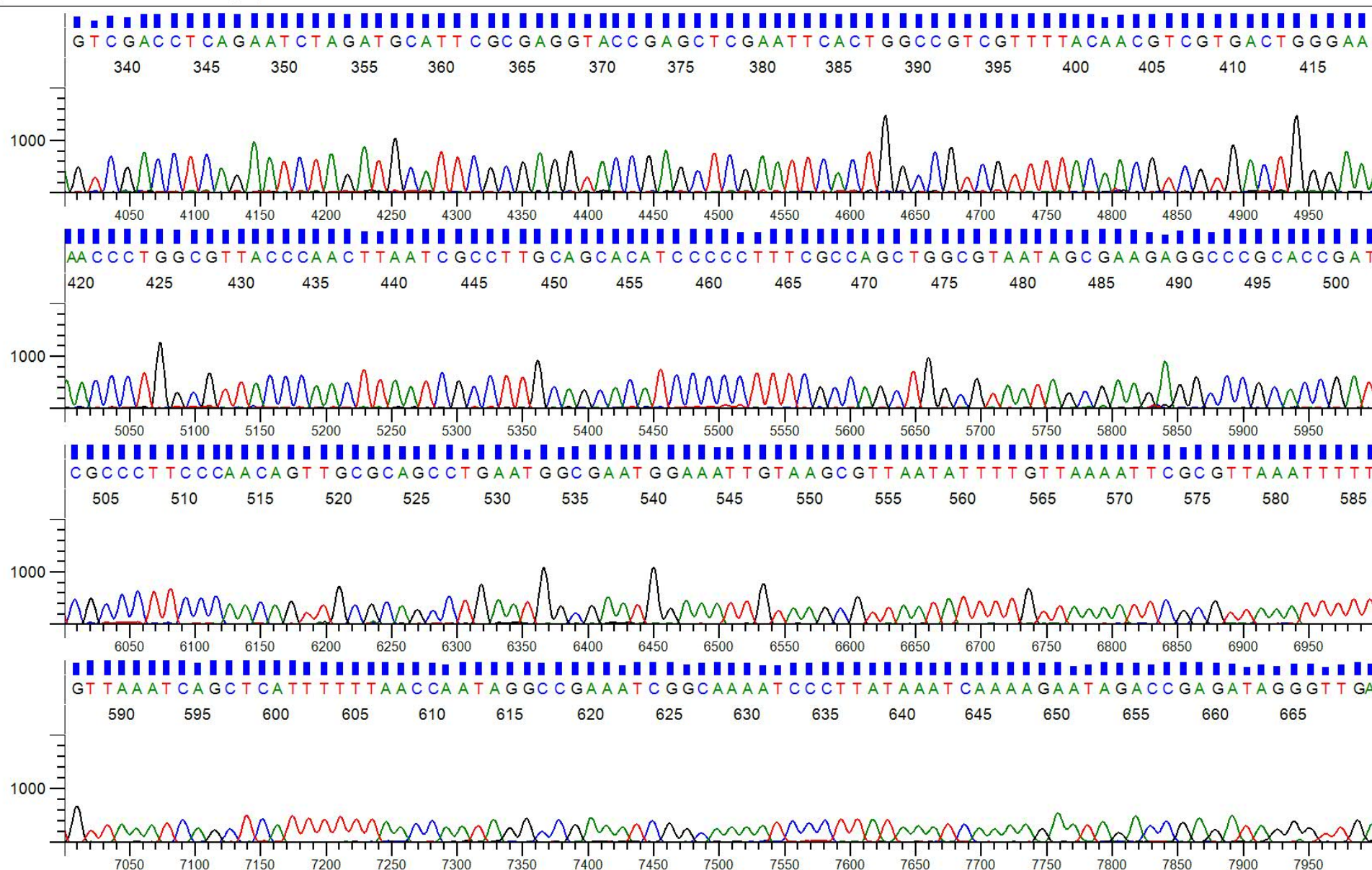

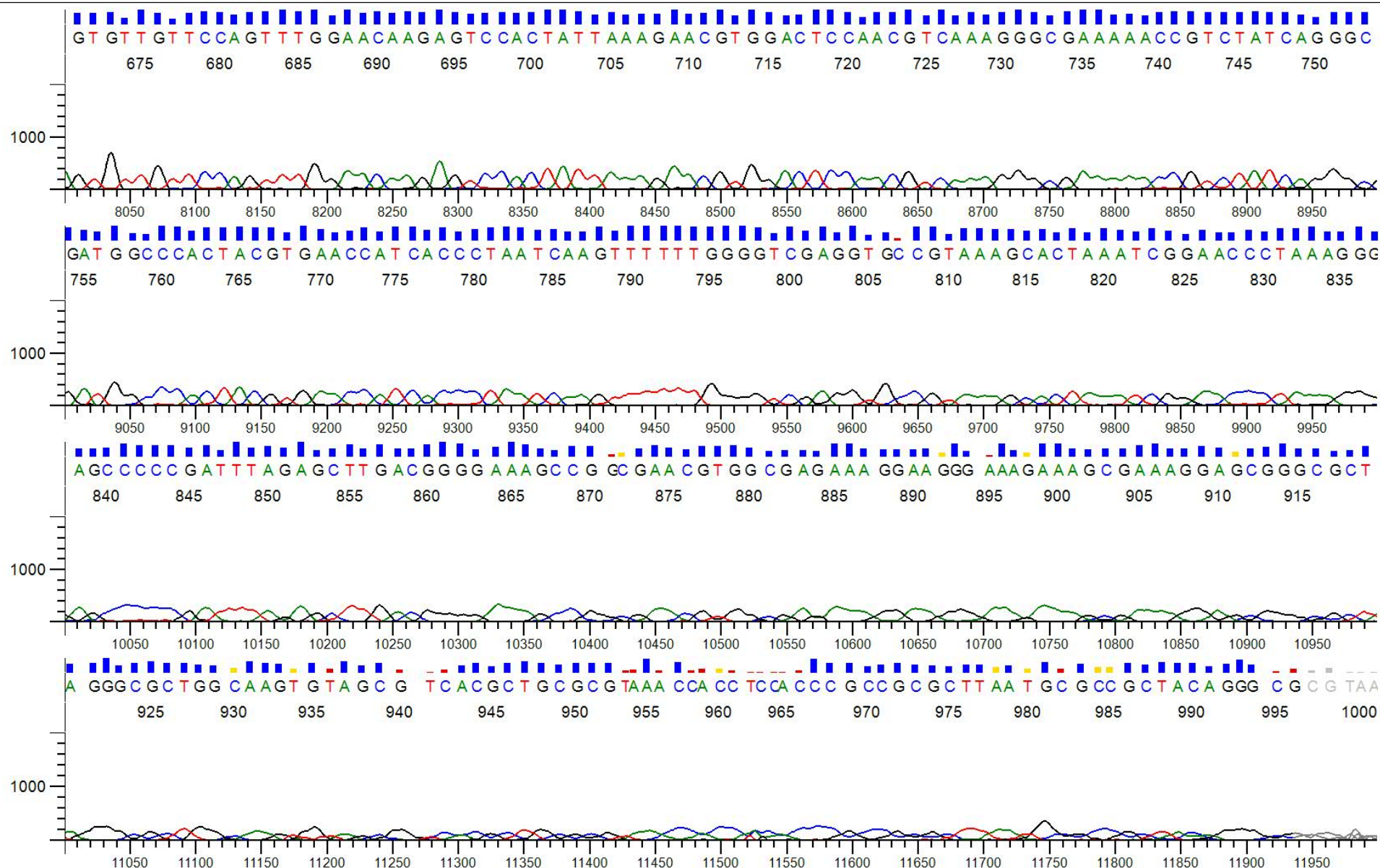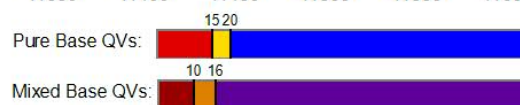

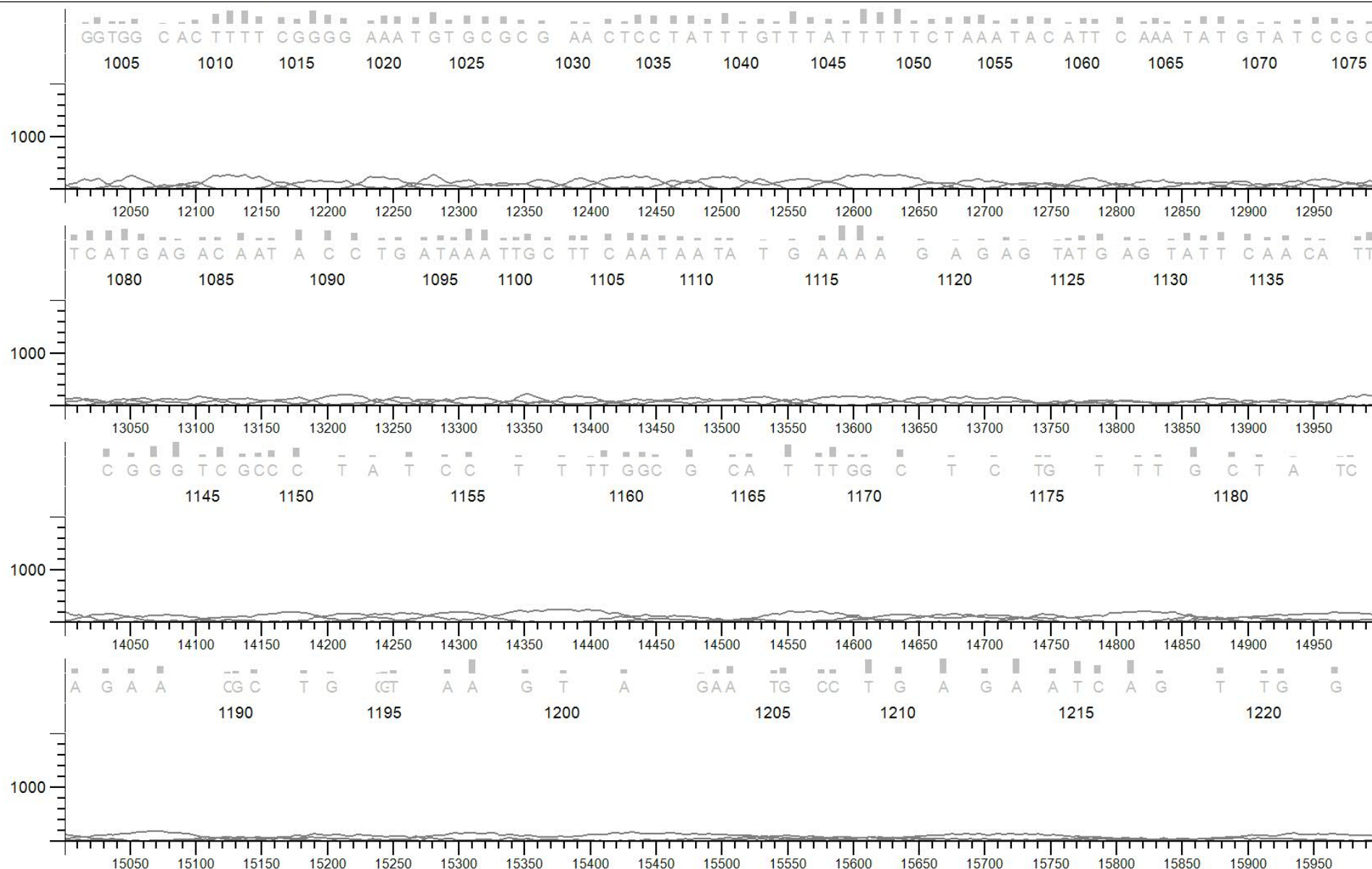

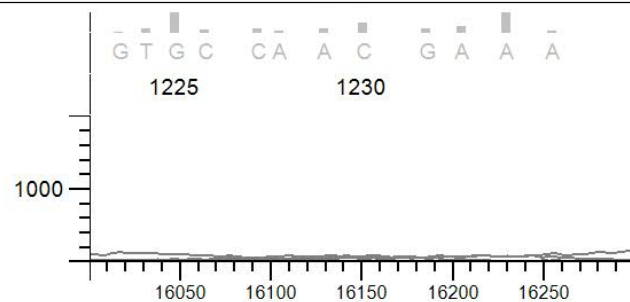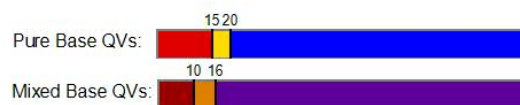

Supplement: Figure 3—source data 2. [file elife-69916-fig3-data2.zip › Figure 3B.C_Source data3_Bisulphite sequencing_mtDNA/SS4-MT-BIS-1.6_T7FOR.pdf]

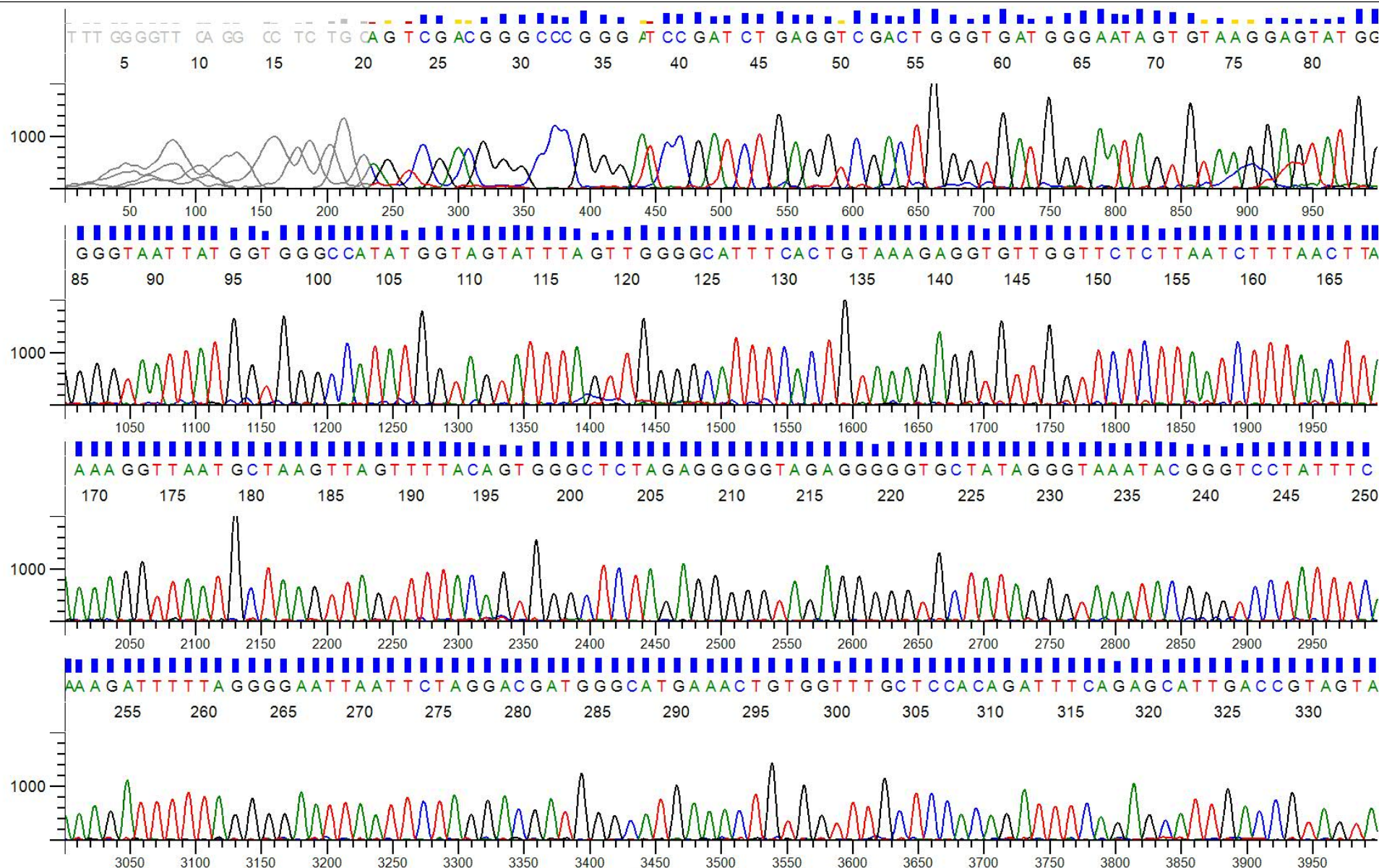

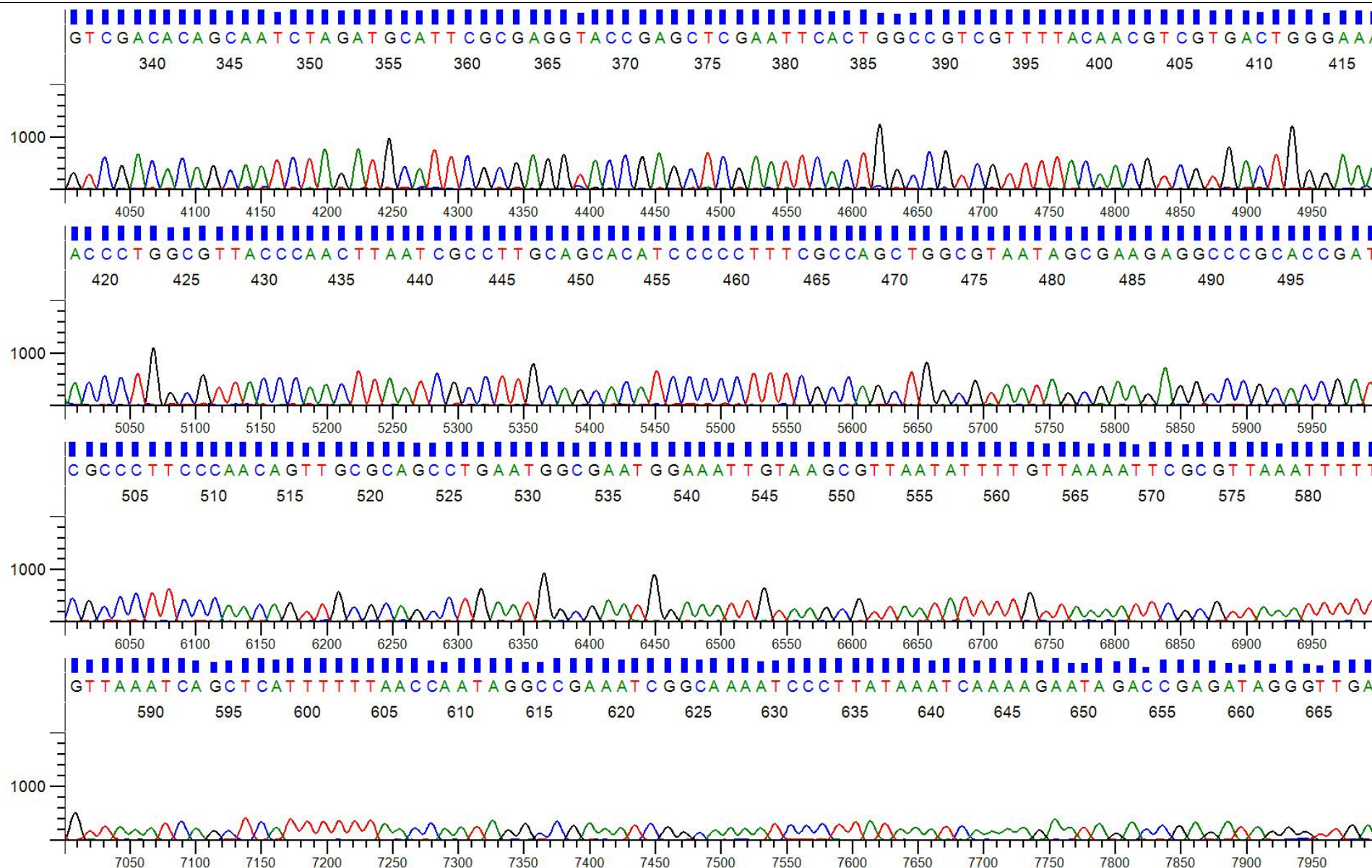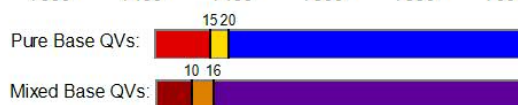

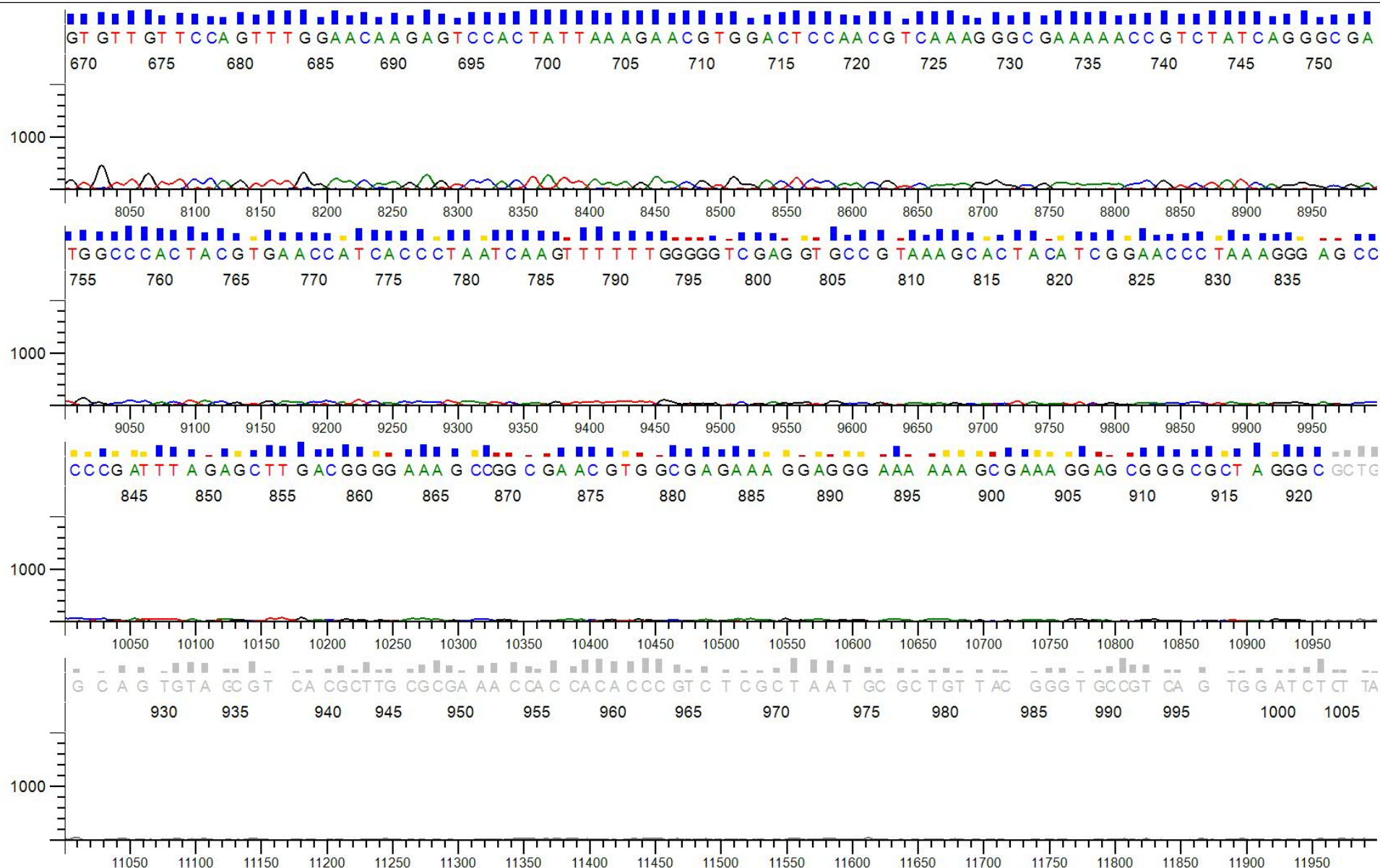

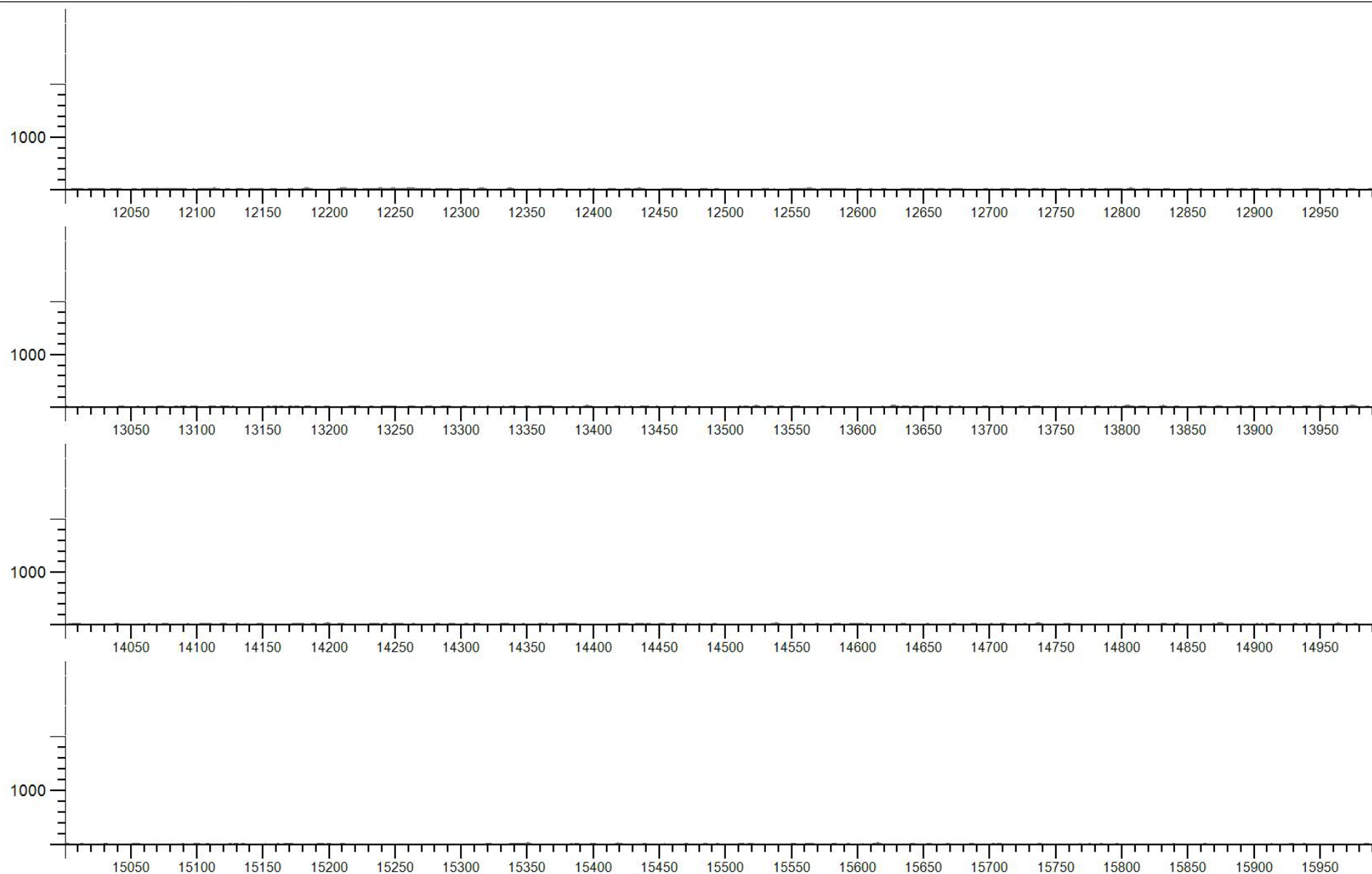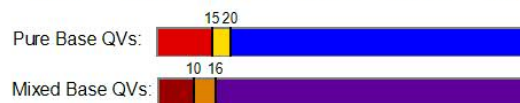

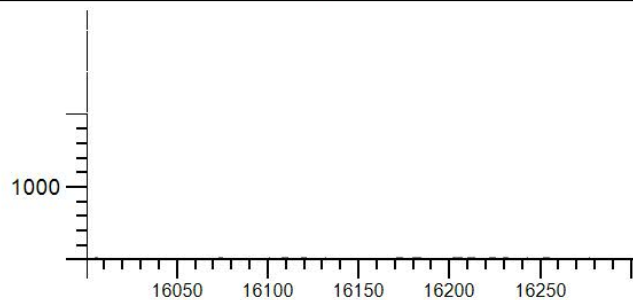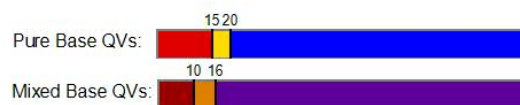

Supplement: Figure 3—source data 2. [file elife-69916-fig3-data2.zip › Figure 3B.C_Source data3_Bisulphite sequencing_mtDNA/SD_MTDNA_BSF_1.7_T7FOR-A02.pdf]

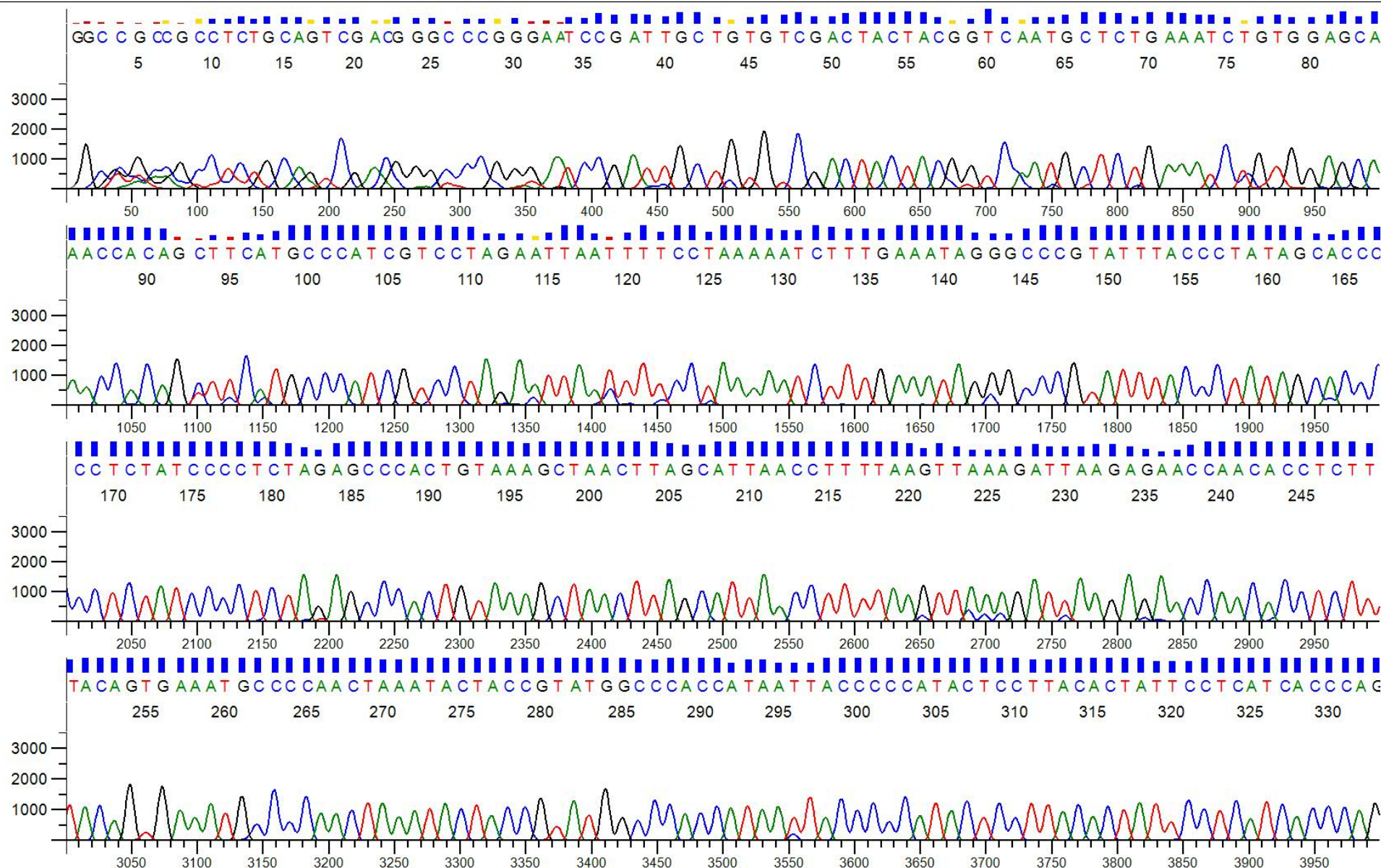

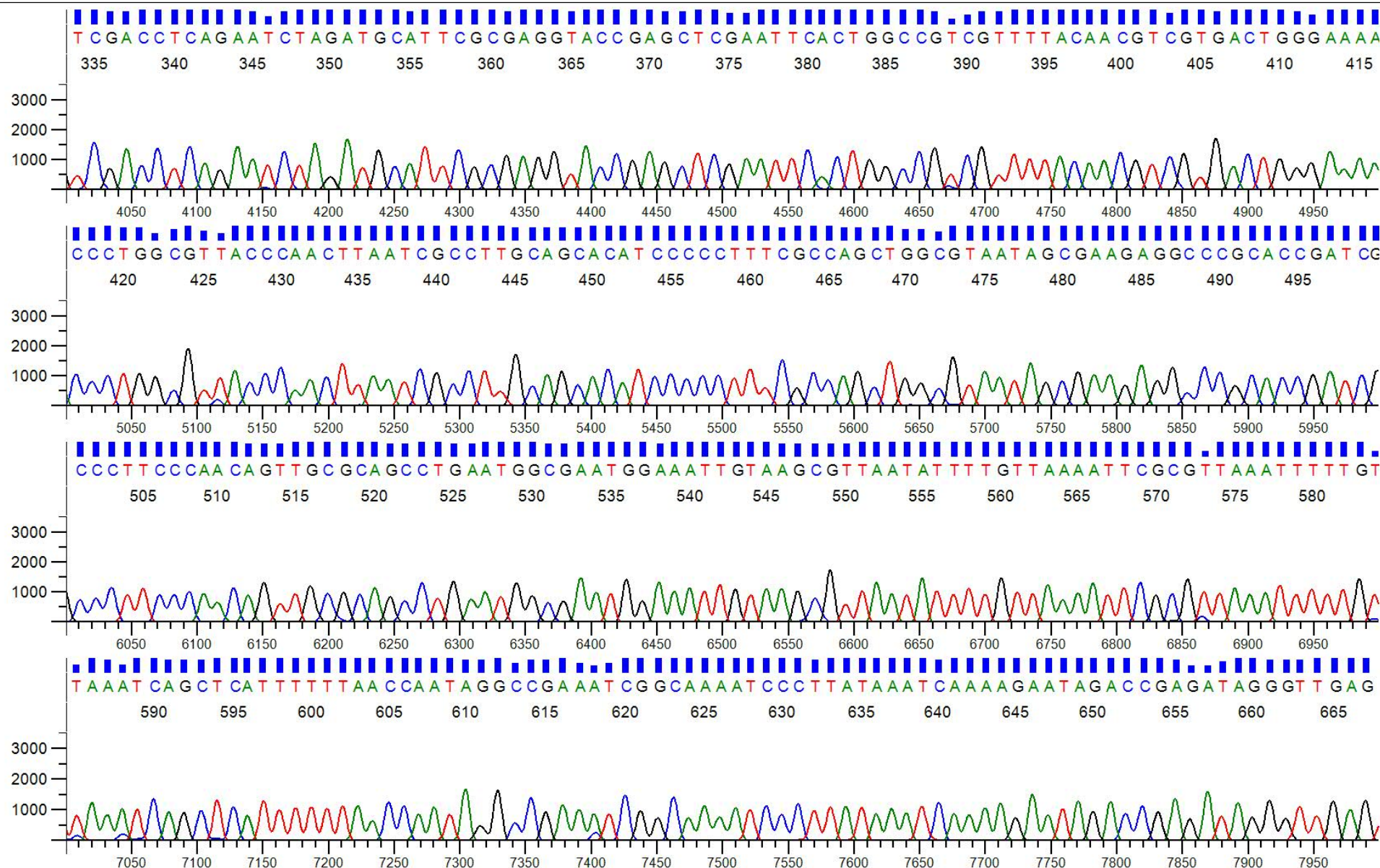

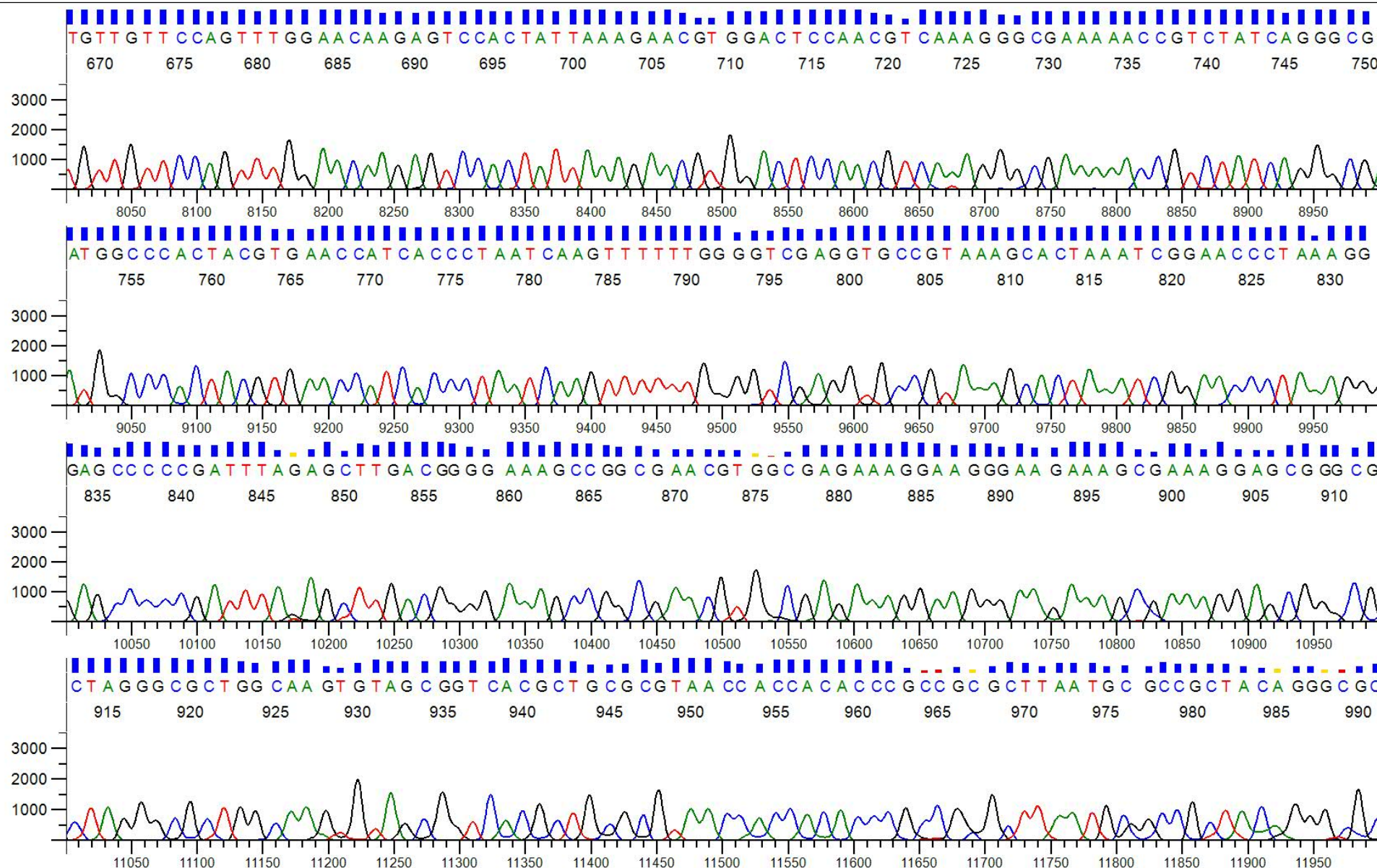

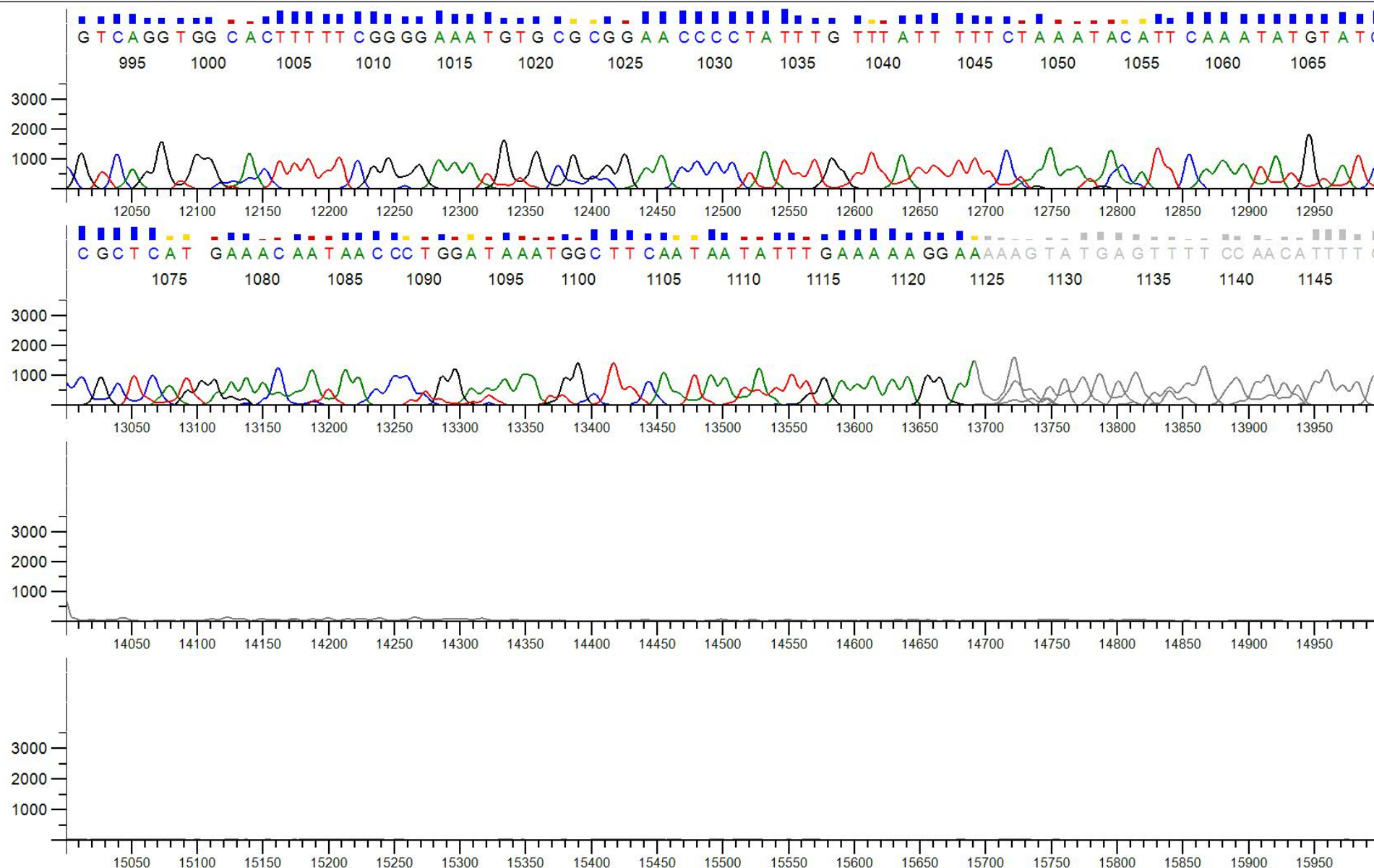

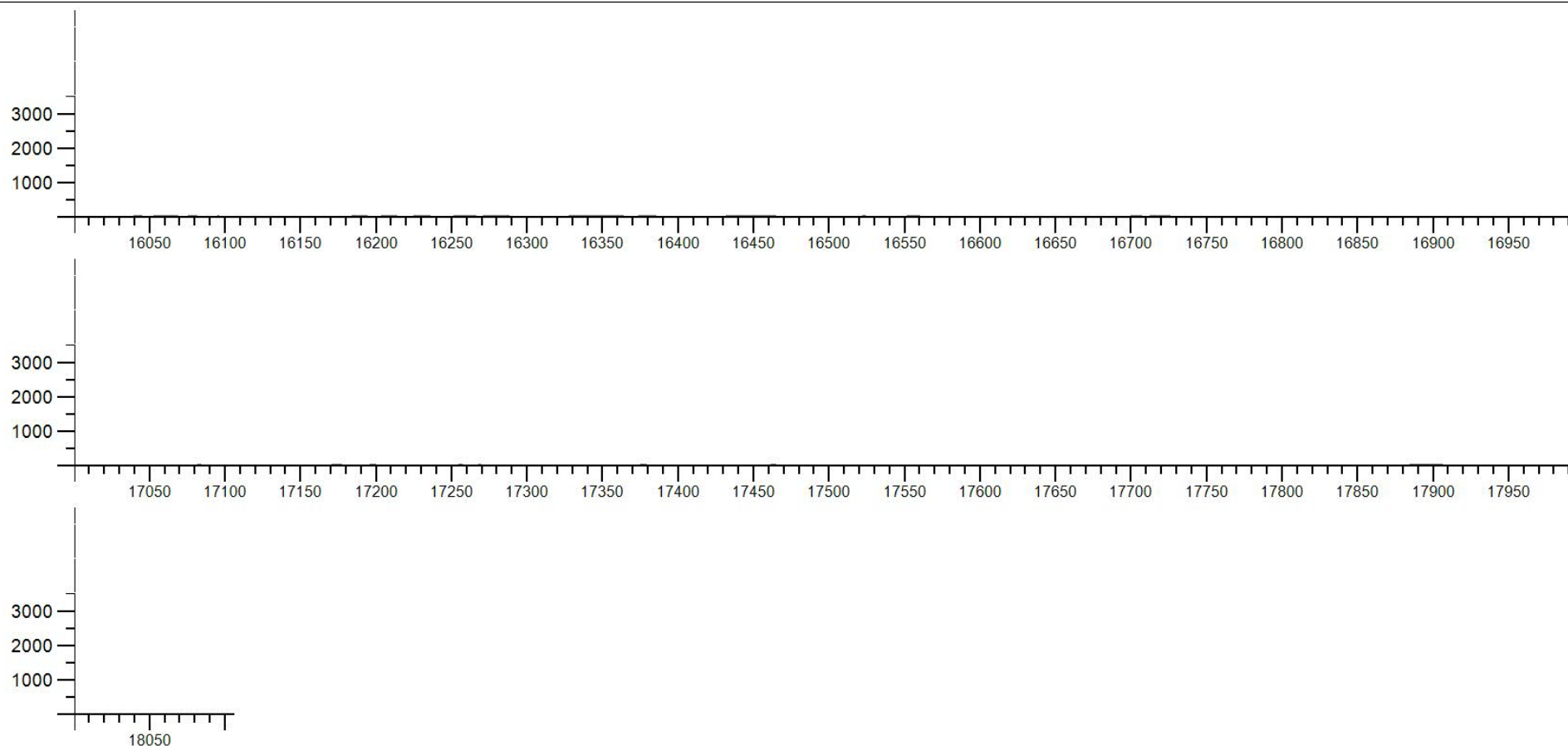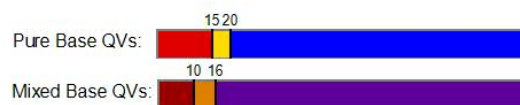

Supplement: Figure 3—source data 2. [file elife-69916-fig3-data2.zip › Figure 3B.C_Source data3_Bisulphite sequencing_mtDNA/SD-MTDNA-BSF-VKKII-1.4_T7-FOR-D04.pdf]

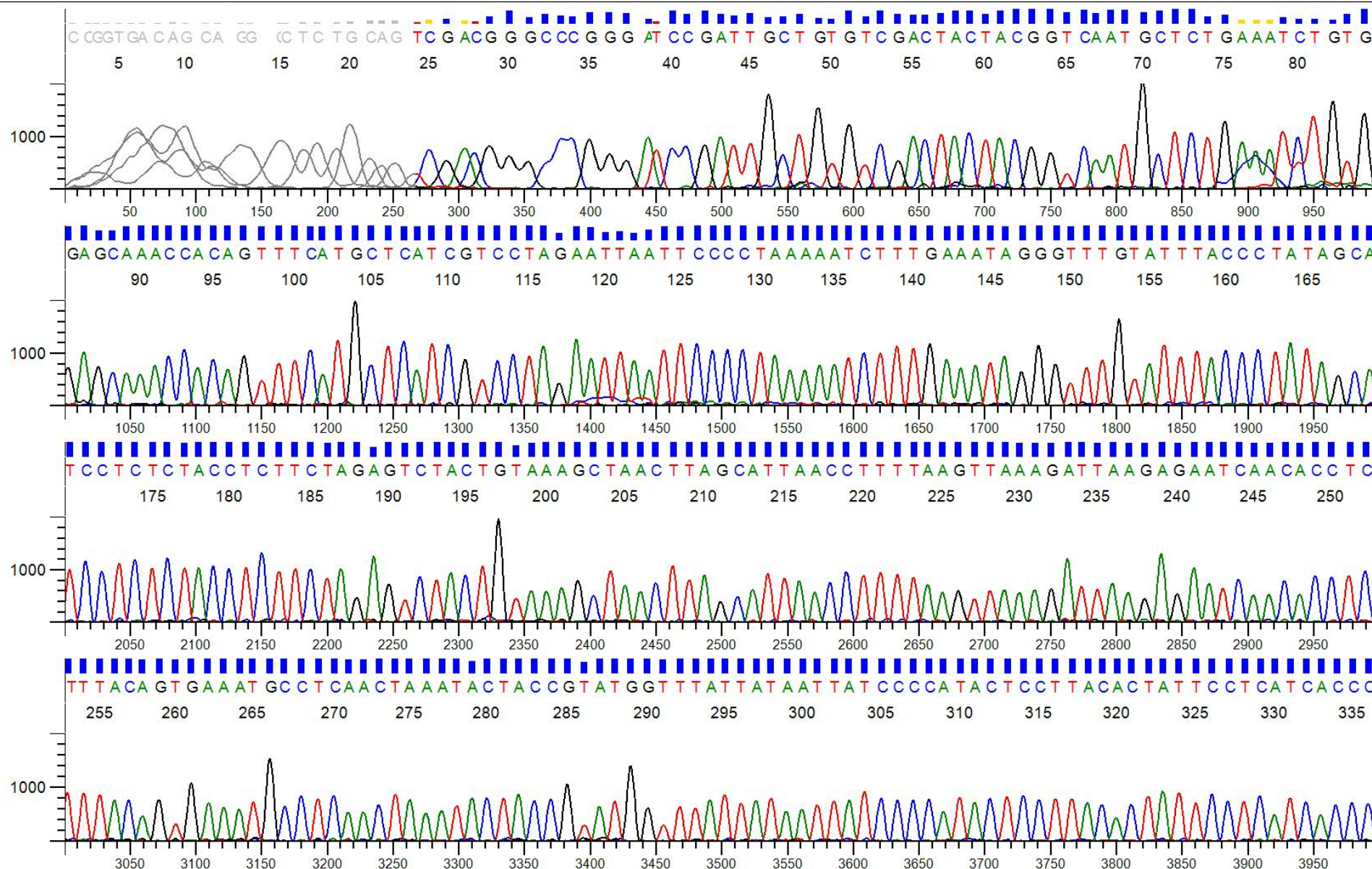

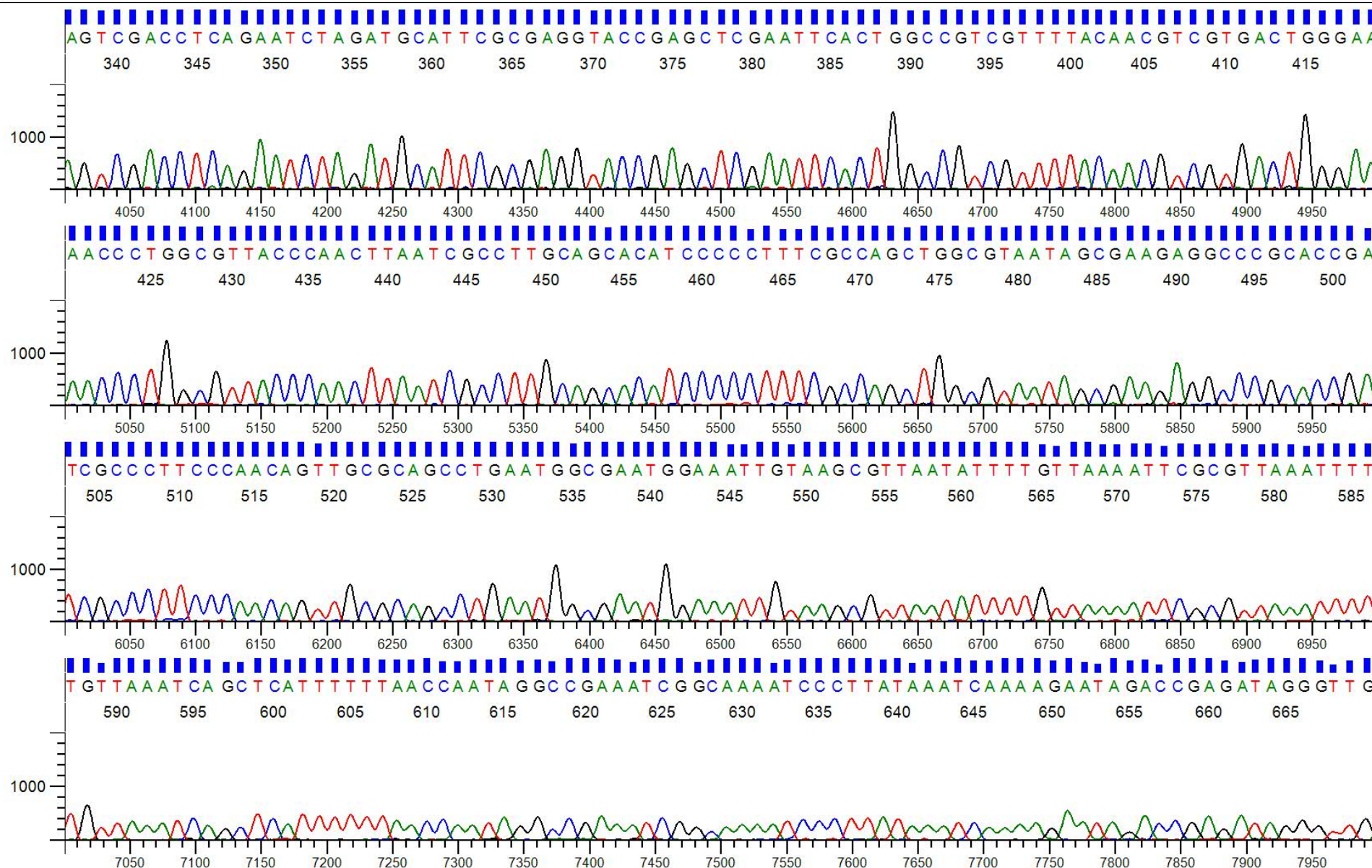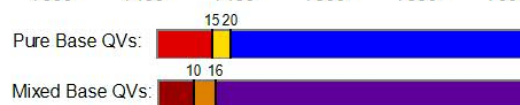

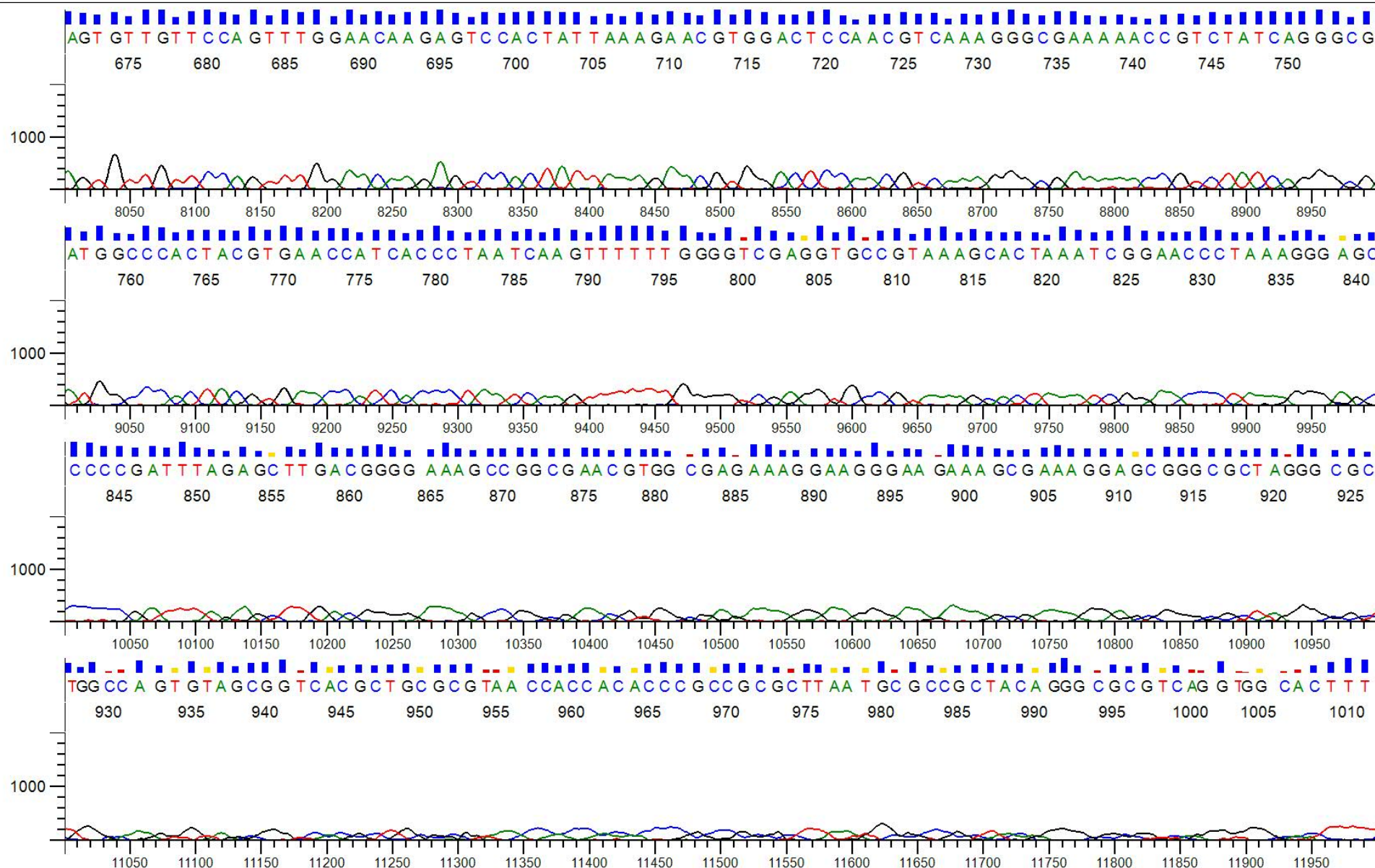

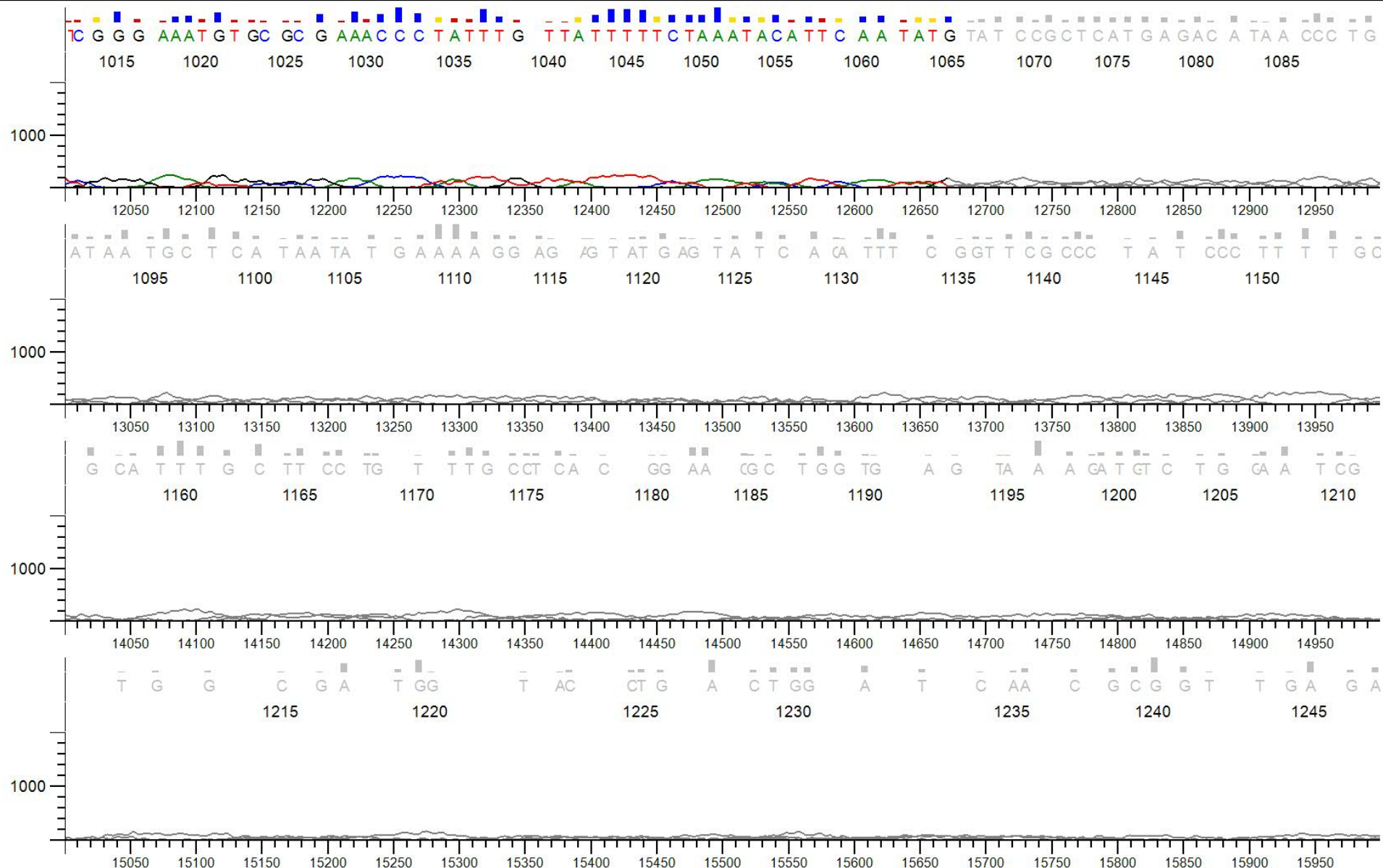

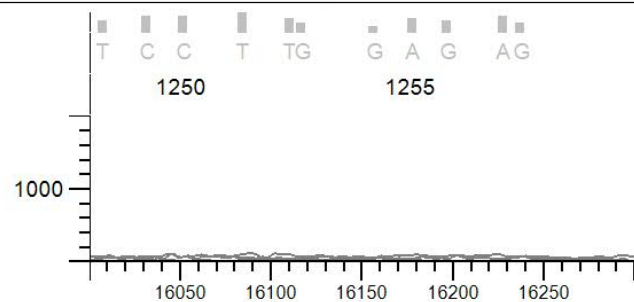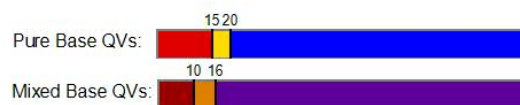

Supplement: Figure 3—source data 2. [file elife-69916-fig3-data2.zip › Figure 3B.C_Source data3_Bisulphite sequencing_mtDNA/SS4_MT_BIS_1.2_T7FOR.pdf]

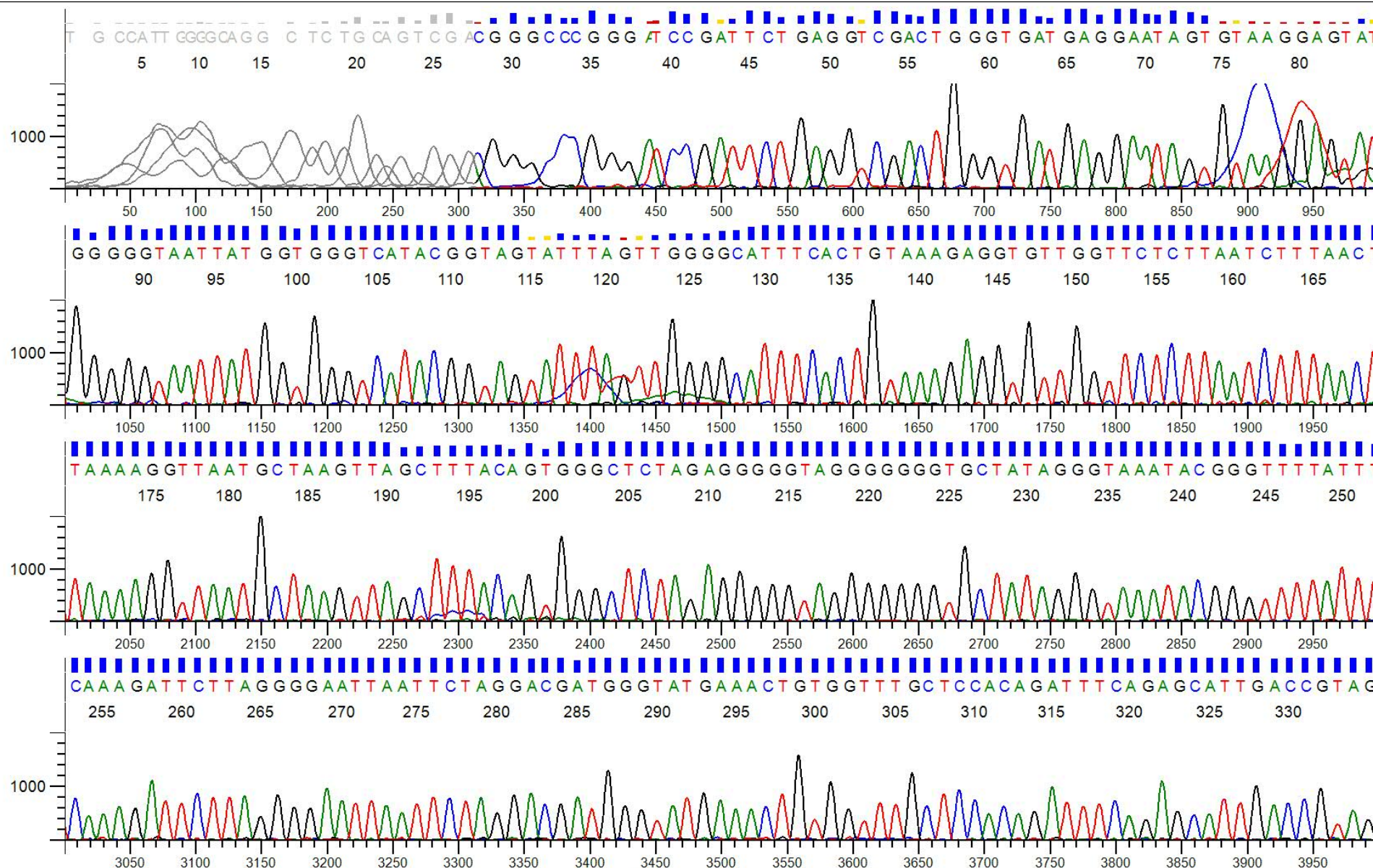

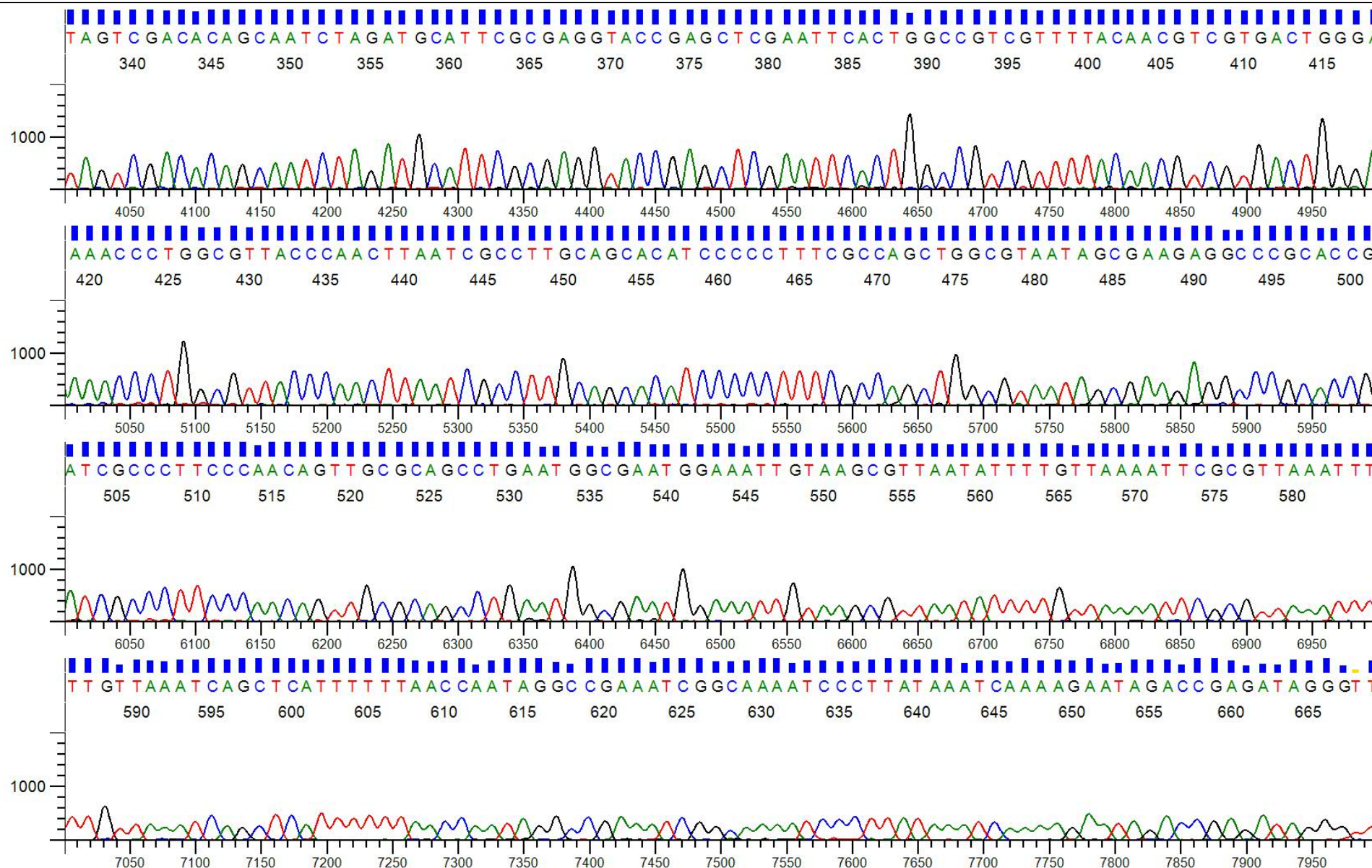

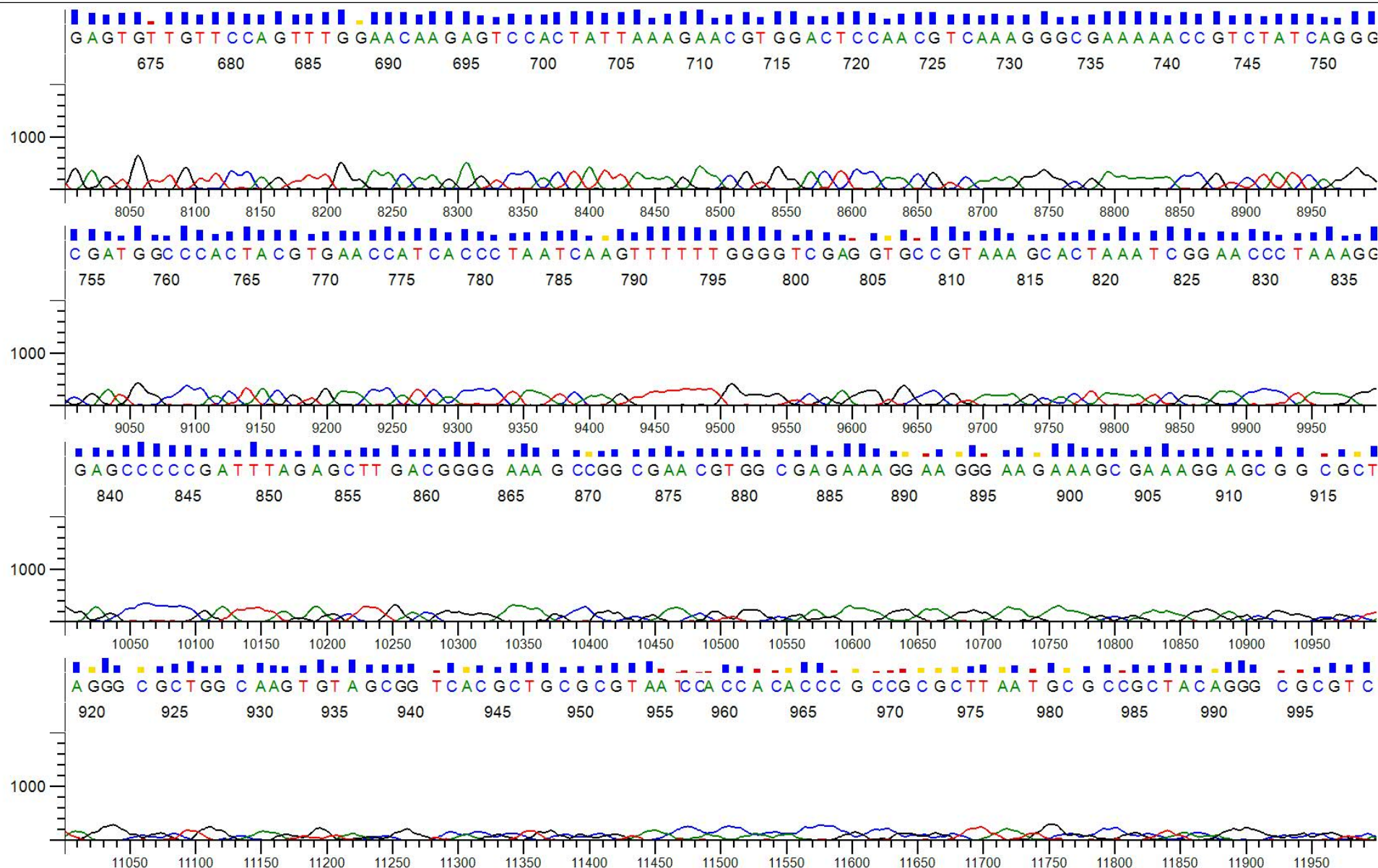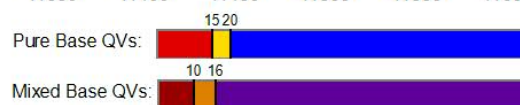

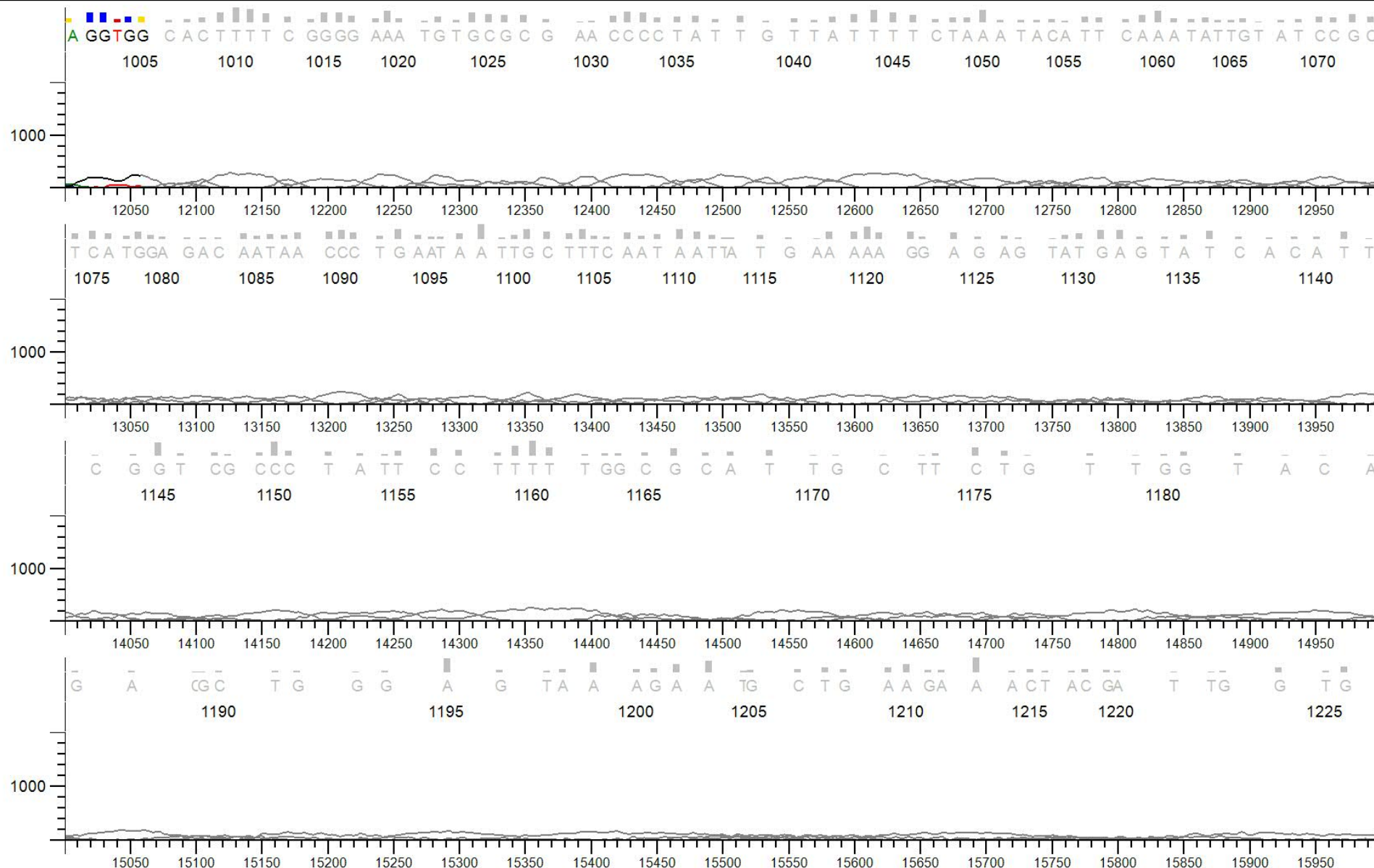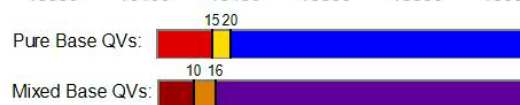

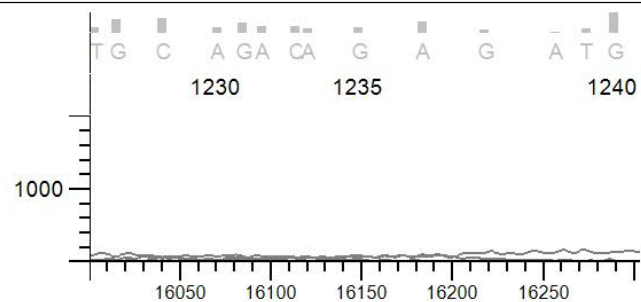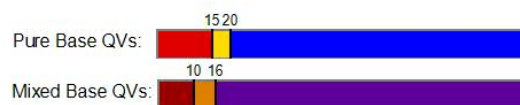

Supplement: Figure 3—source data 2. [file elife-69916-fig3-data2.zip › Figure 3B.C_Source data3_Bisulphite sequencing_mtDNA/SS4-MT-BIS-2.7_T7FOR.pdf]

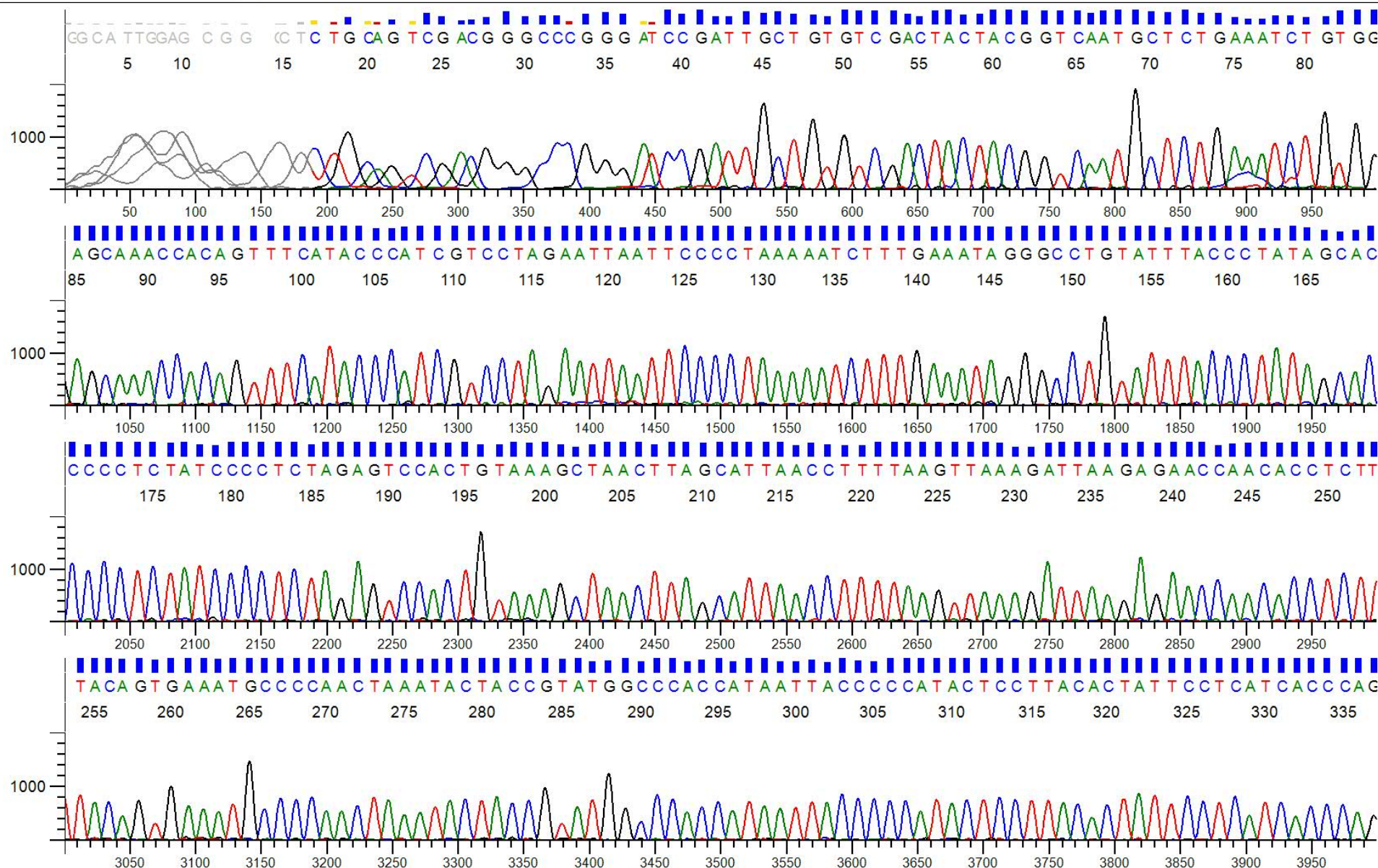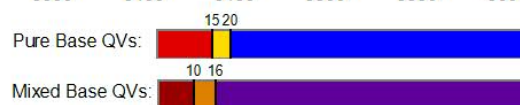

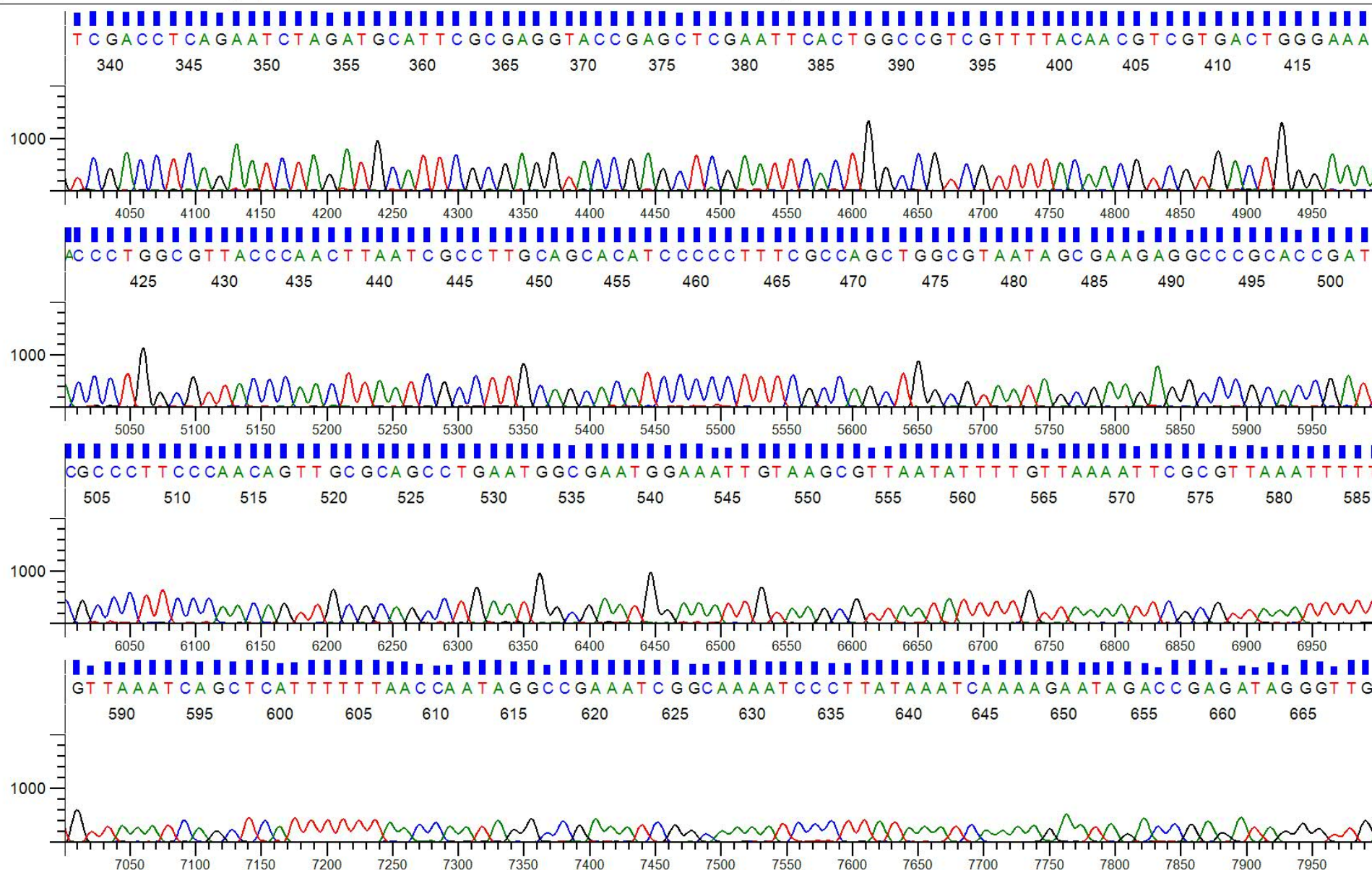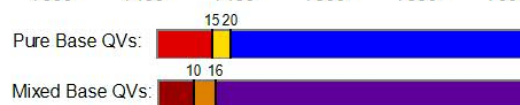

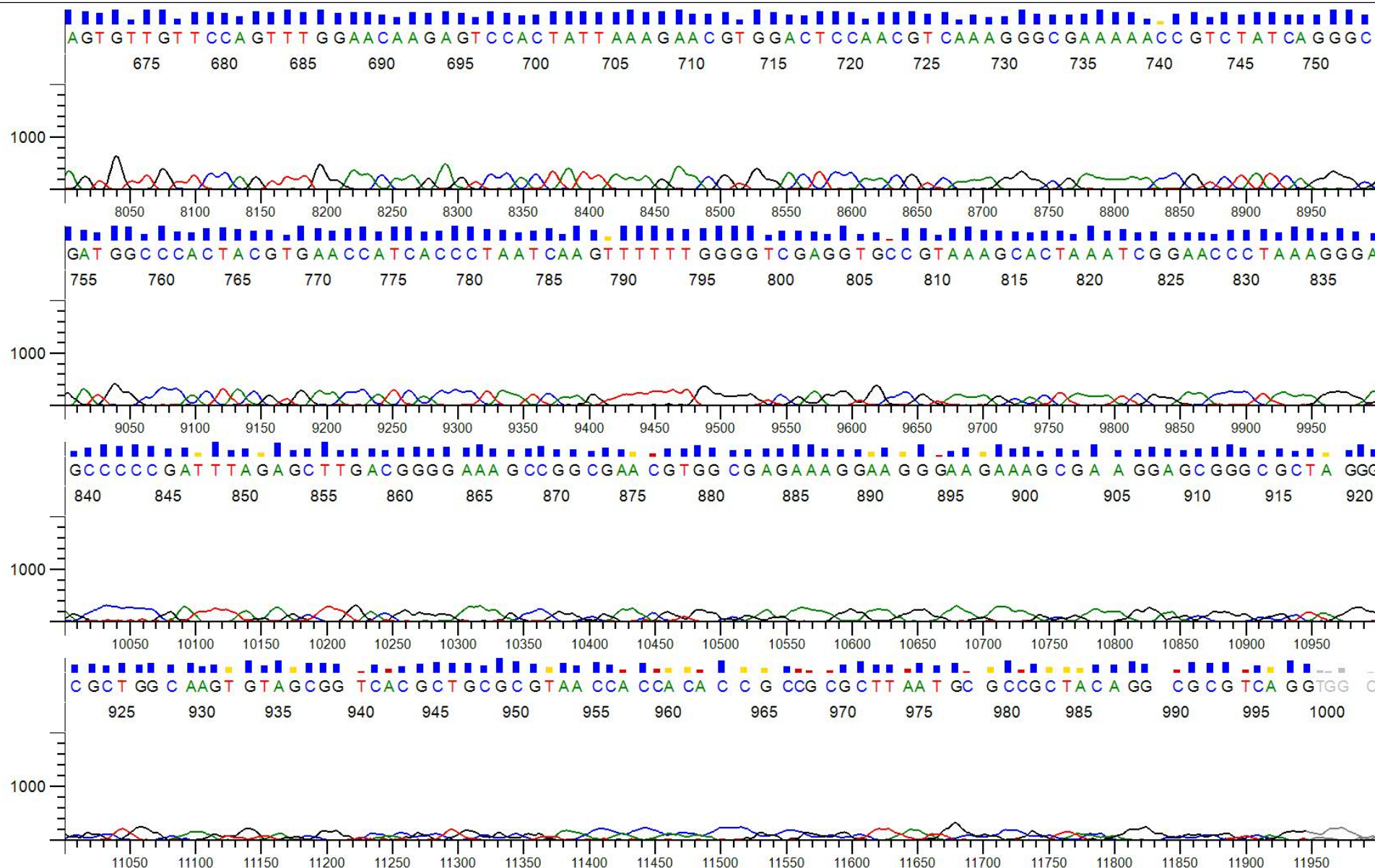

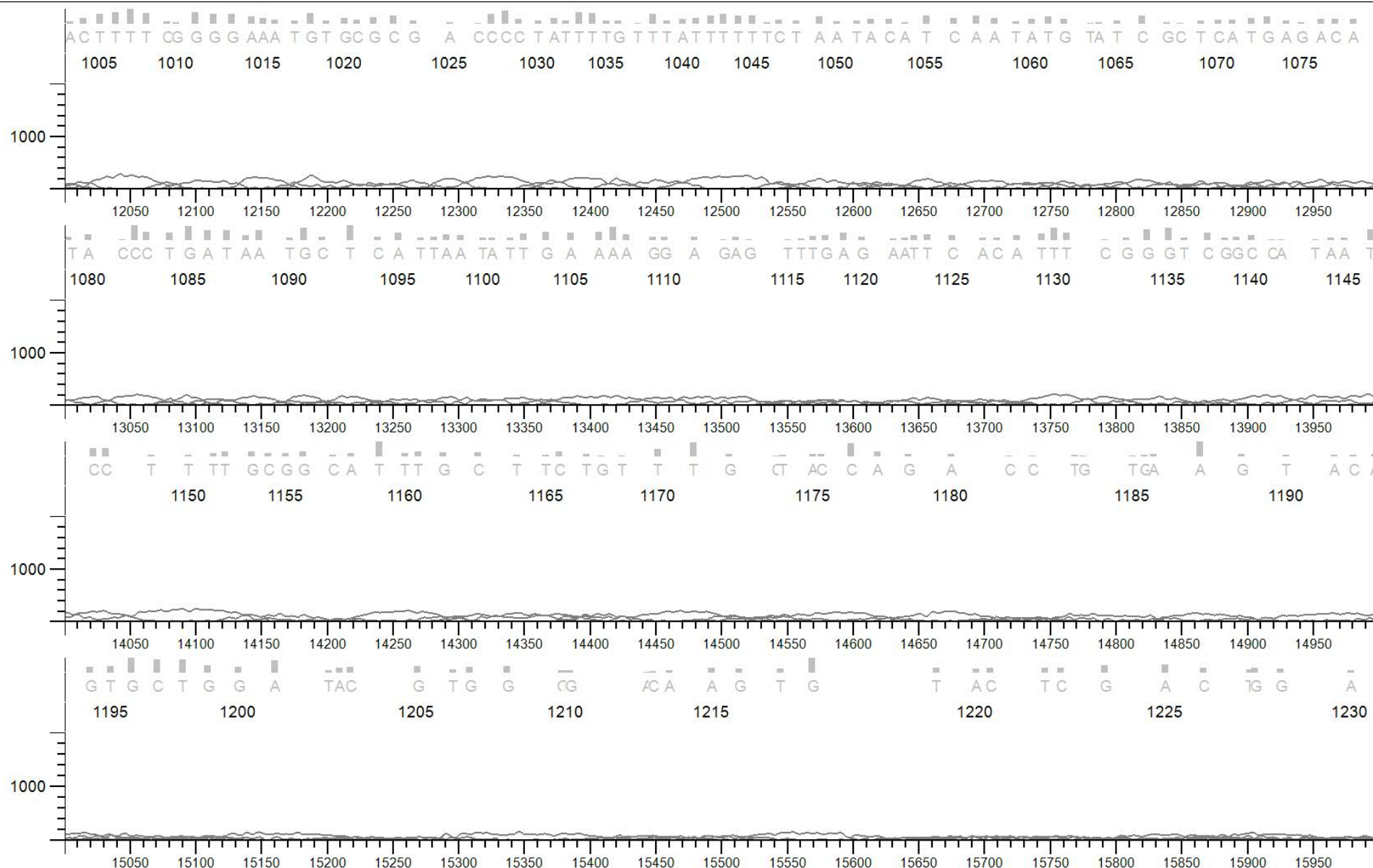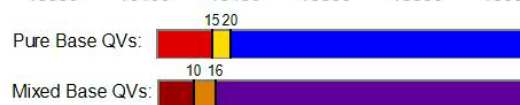

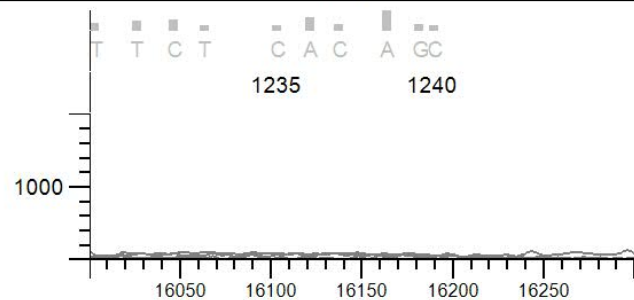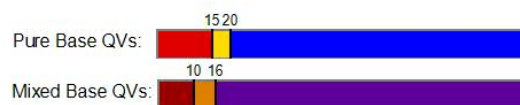

Supplement: Figure 3—source data 2. [file elife-69916-fig3-data2.zip › Figure 3B.C_Source data3_Bisulphite sequencing_mtDNA/SS4_MT_BIS_4_T7FOR-A01.pdf]

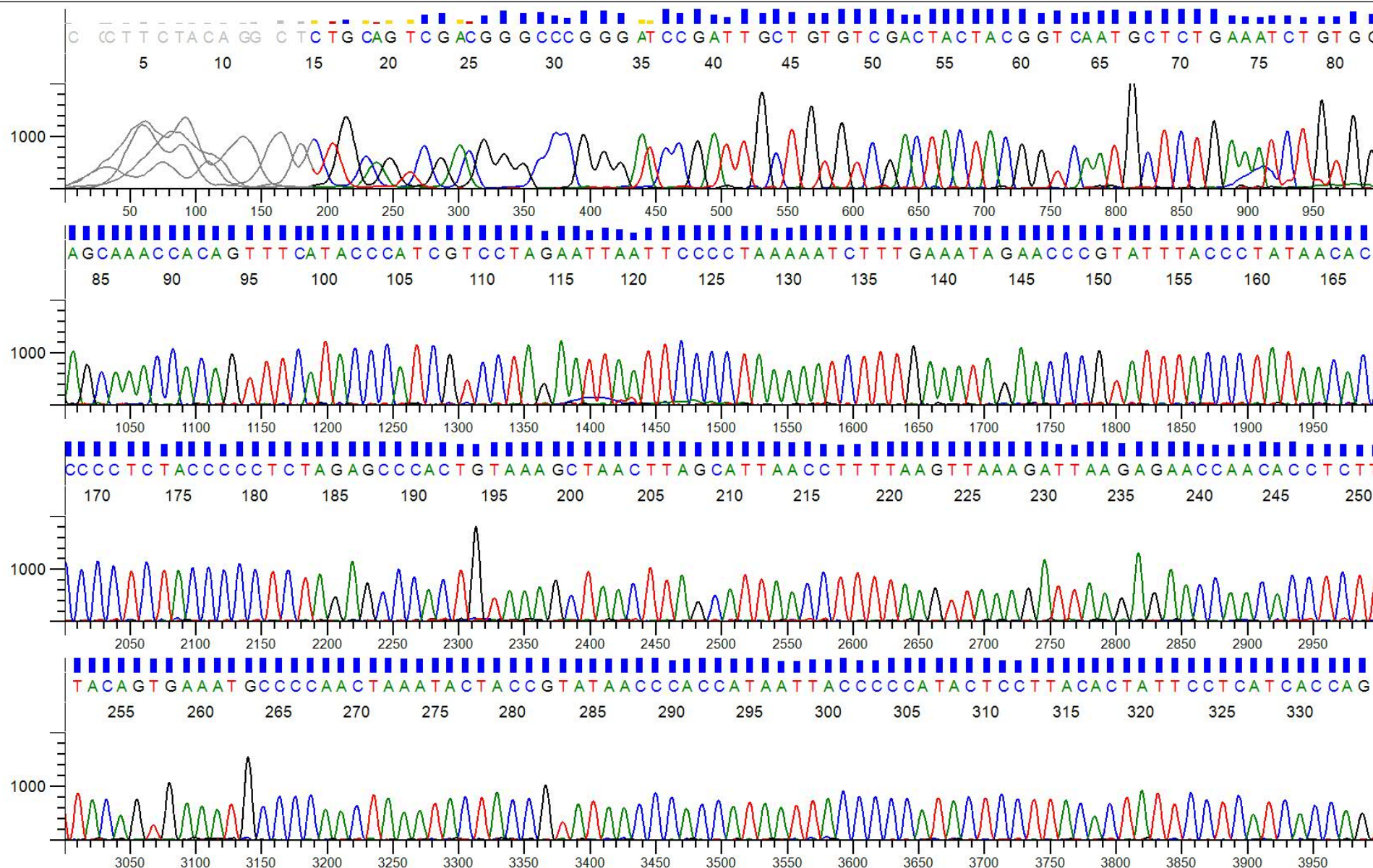

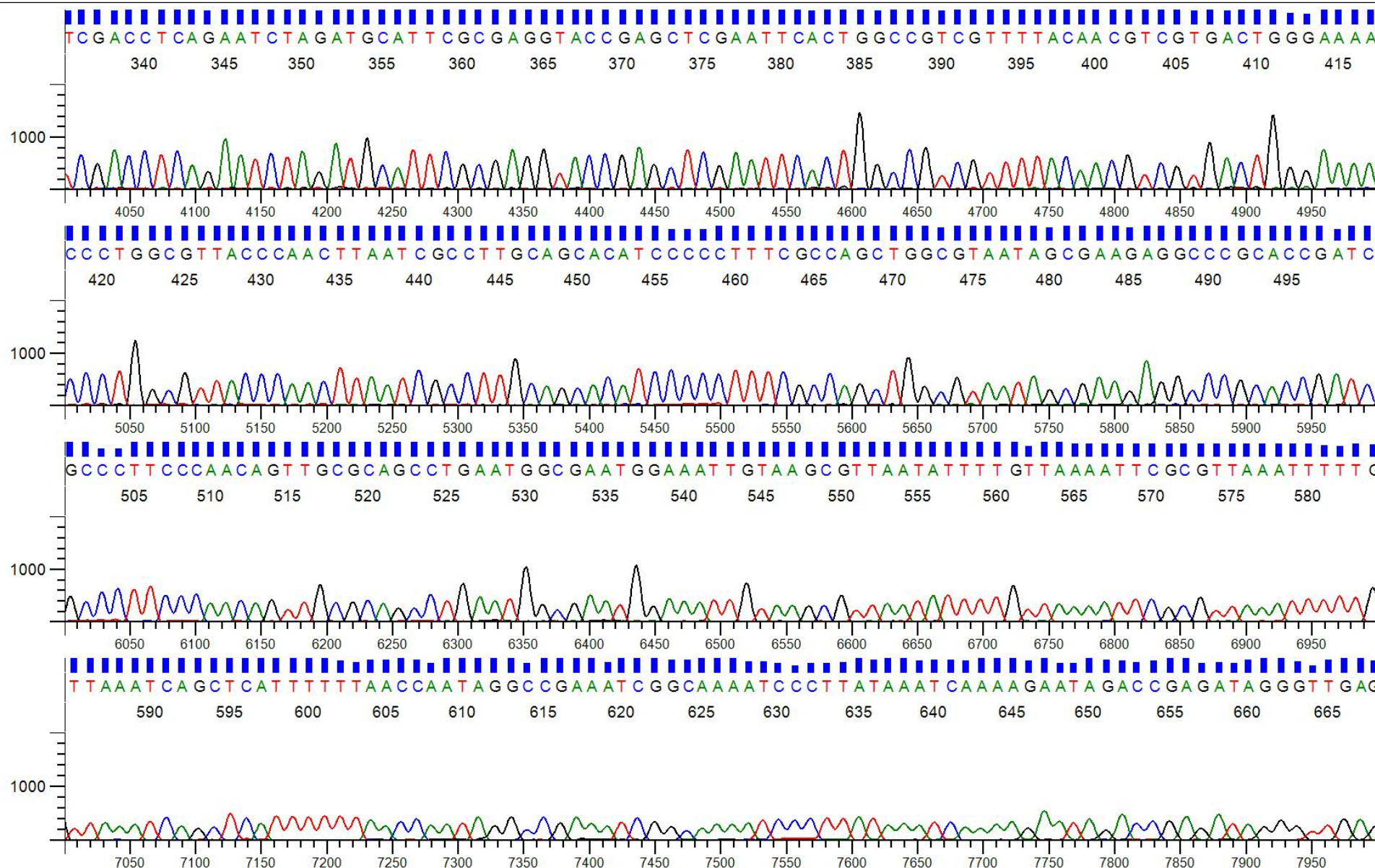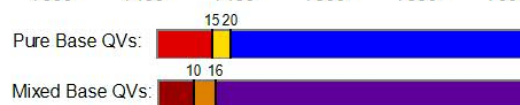

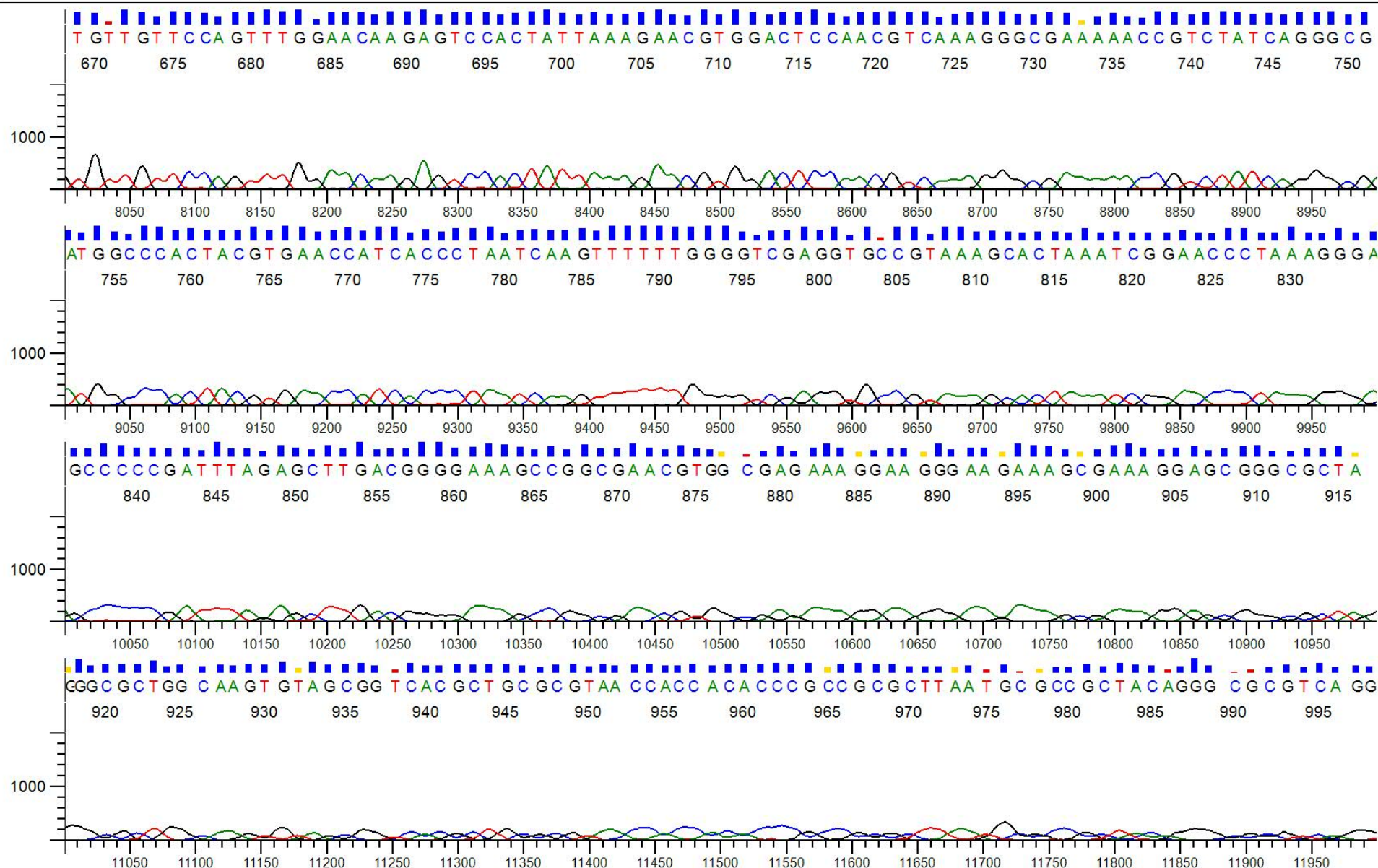

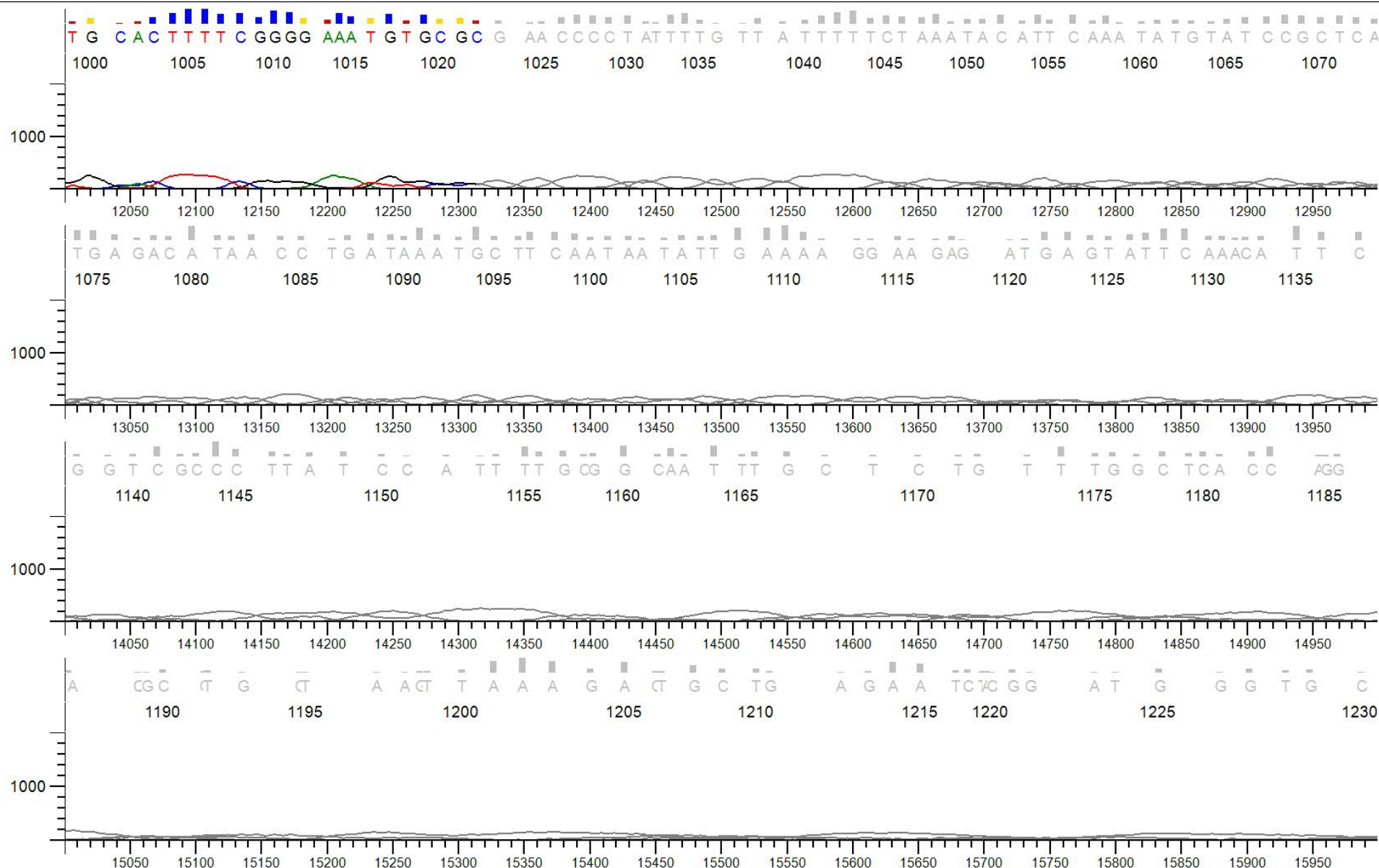

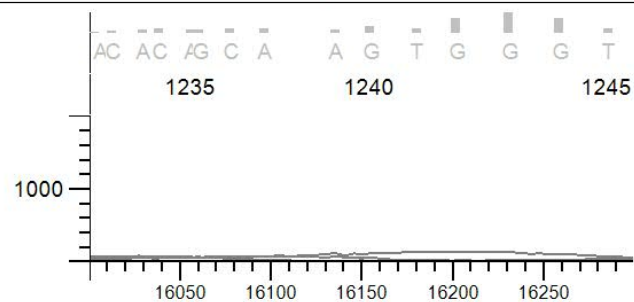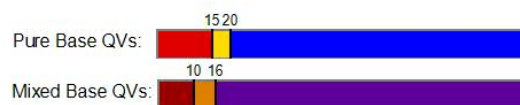

Supplement: Figure 3—source data 2. [file elife-69916-fig3-data2.zip › Figure 3B.C_Source data3_Bisulphite sequencing_mtDNA/SS4-MT-BIS-2.11_T7FOR.pdf]

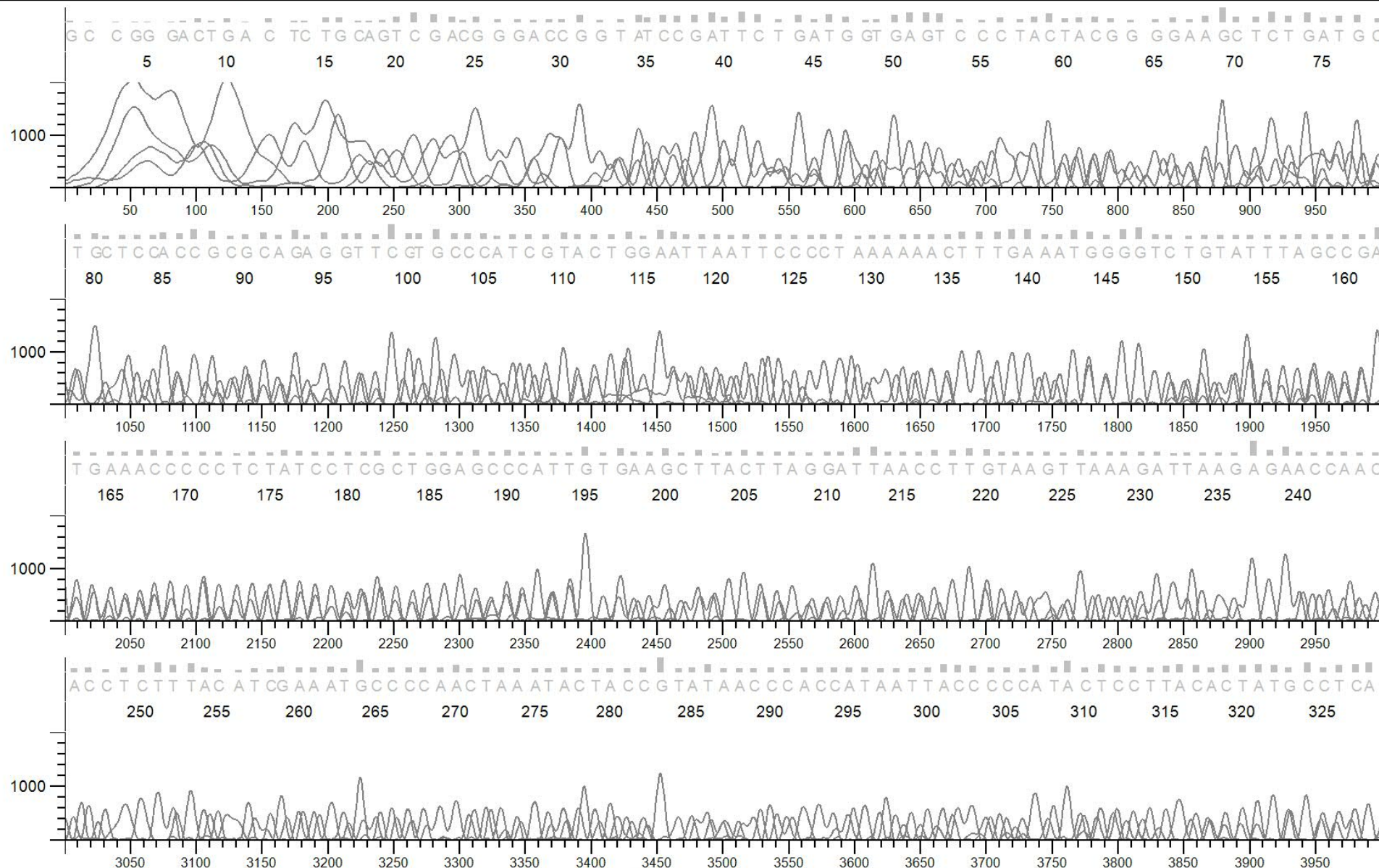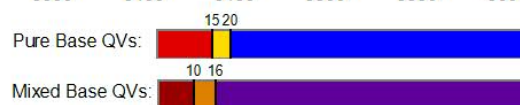

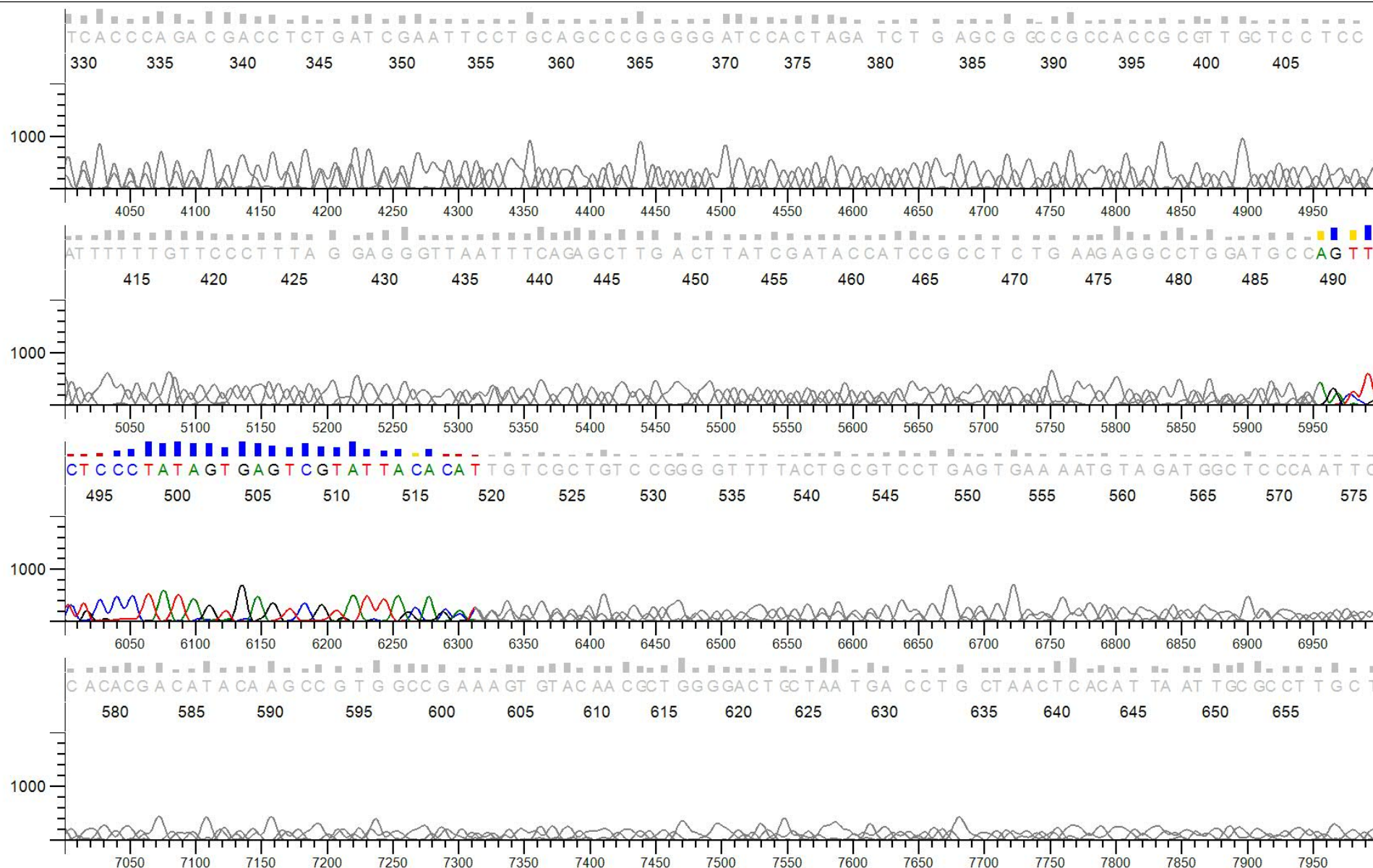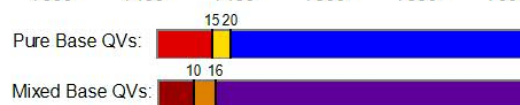

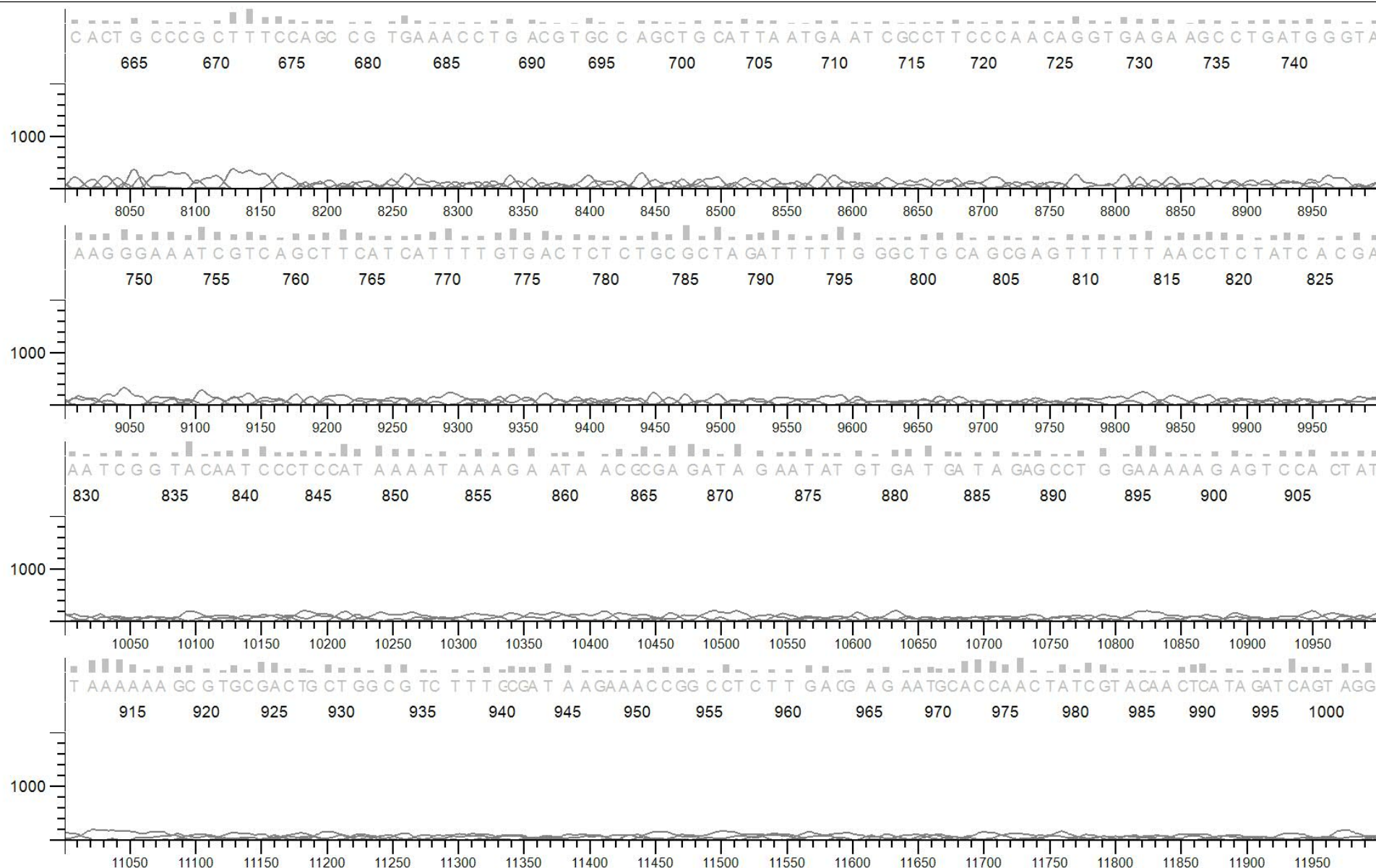

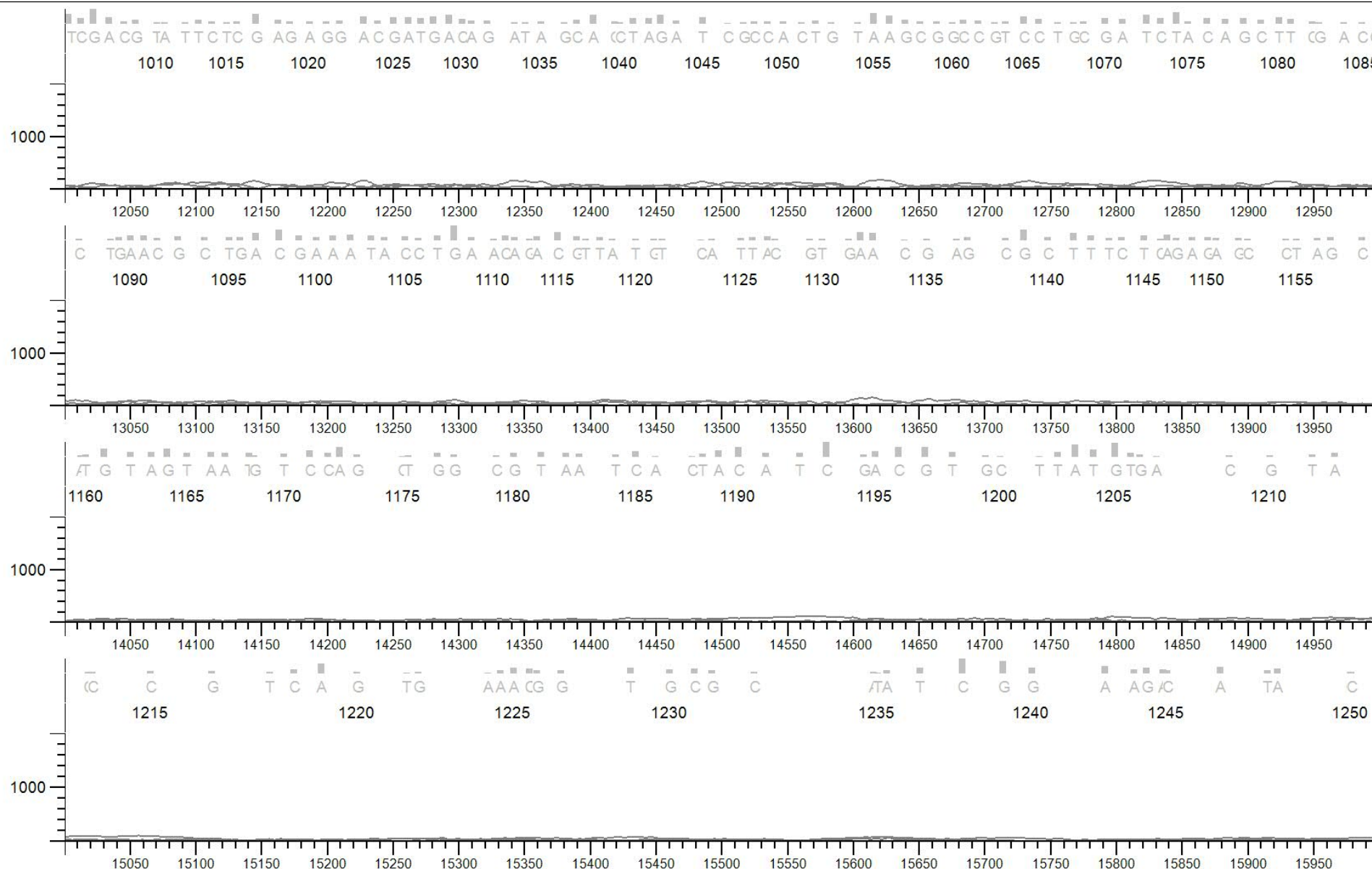

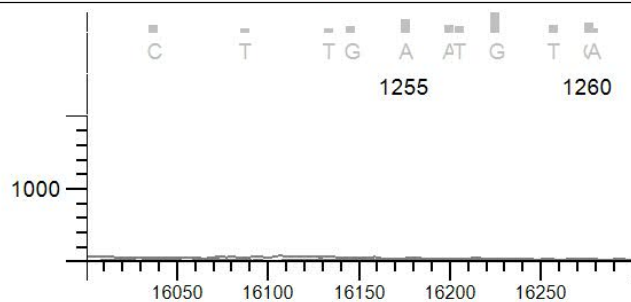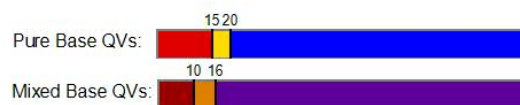

Supplement: Figure 3—source data 2. [file elife-69916-fig3-data2.zip › Figure 3B.C_Source data3_Bisulphite sequencing_mtDNA/SD-MT-DNA-BSF-1.10_T7FOR-C05.pdf]

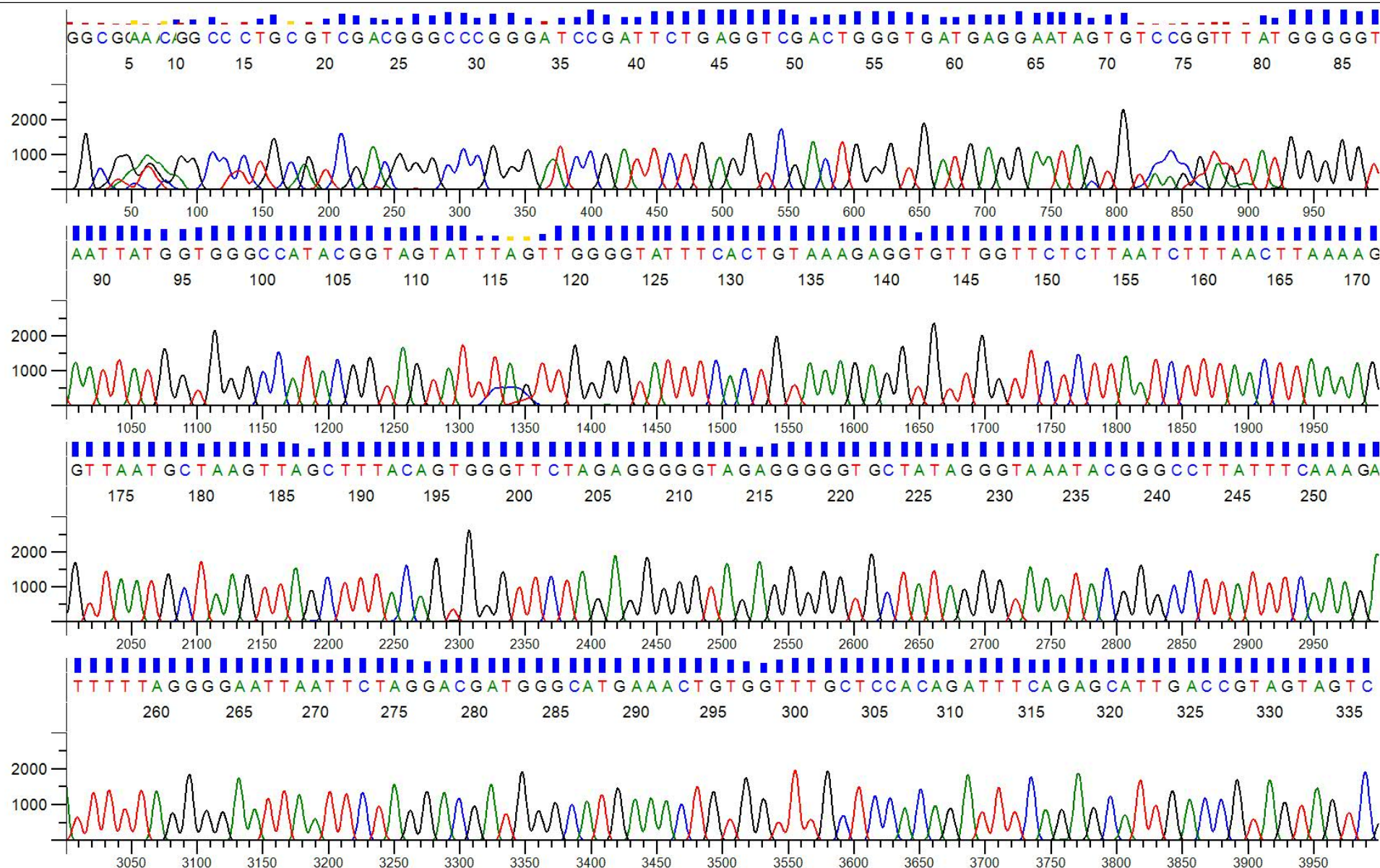

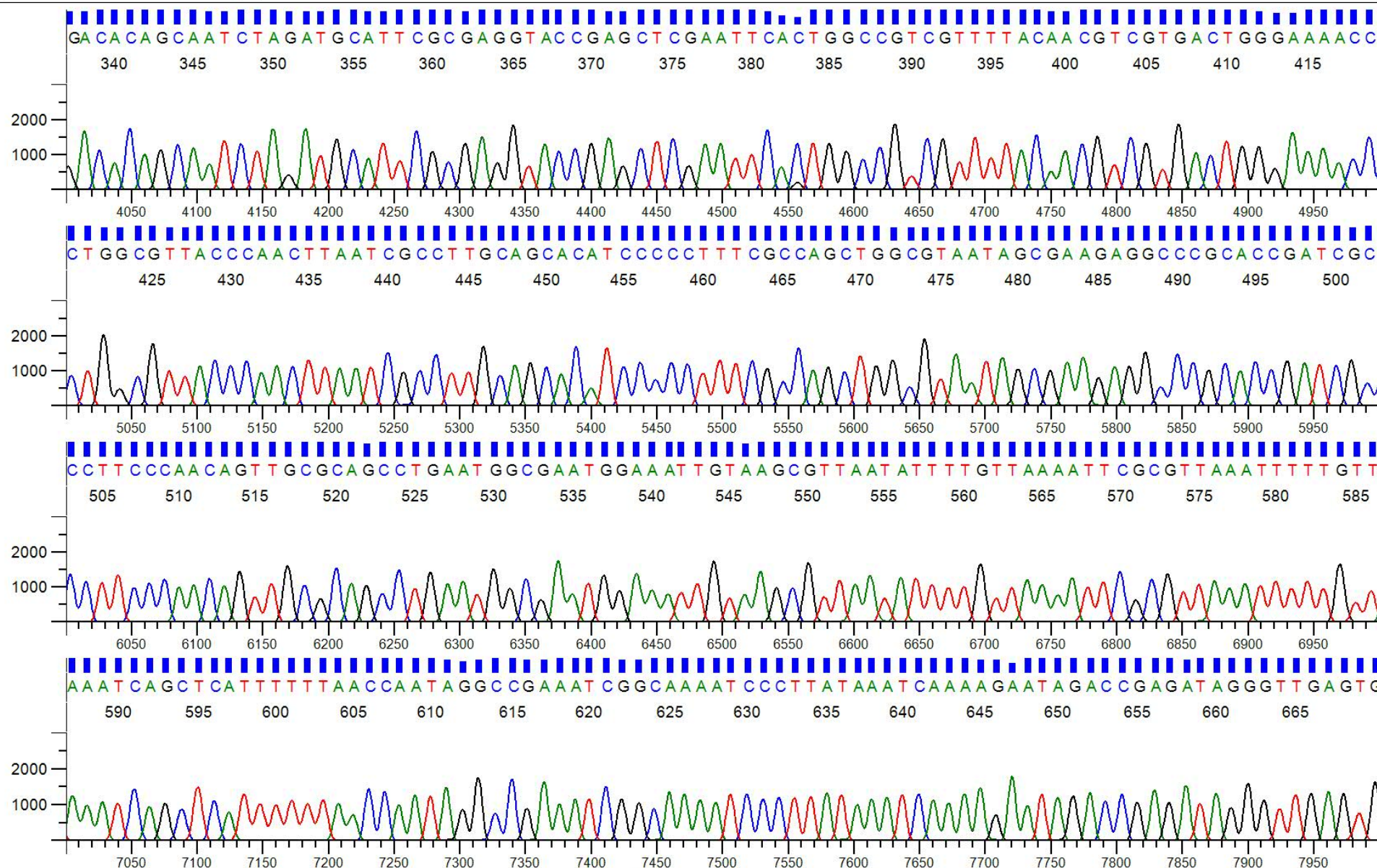

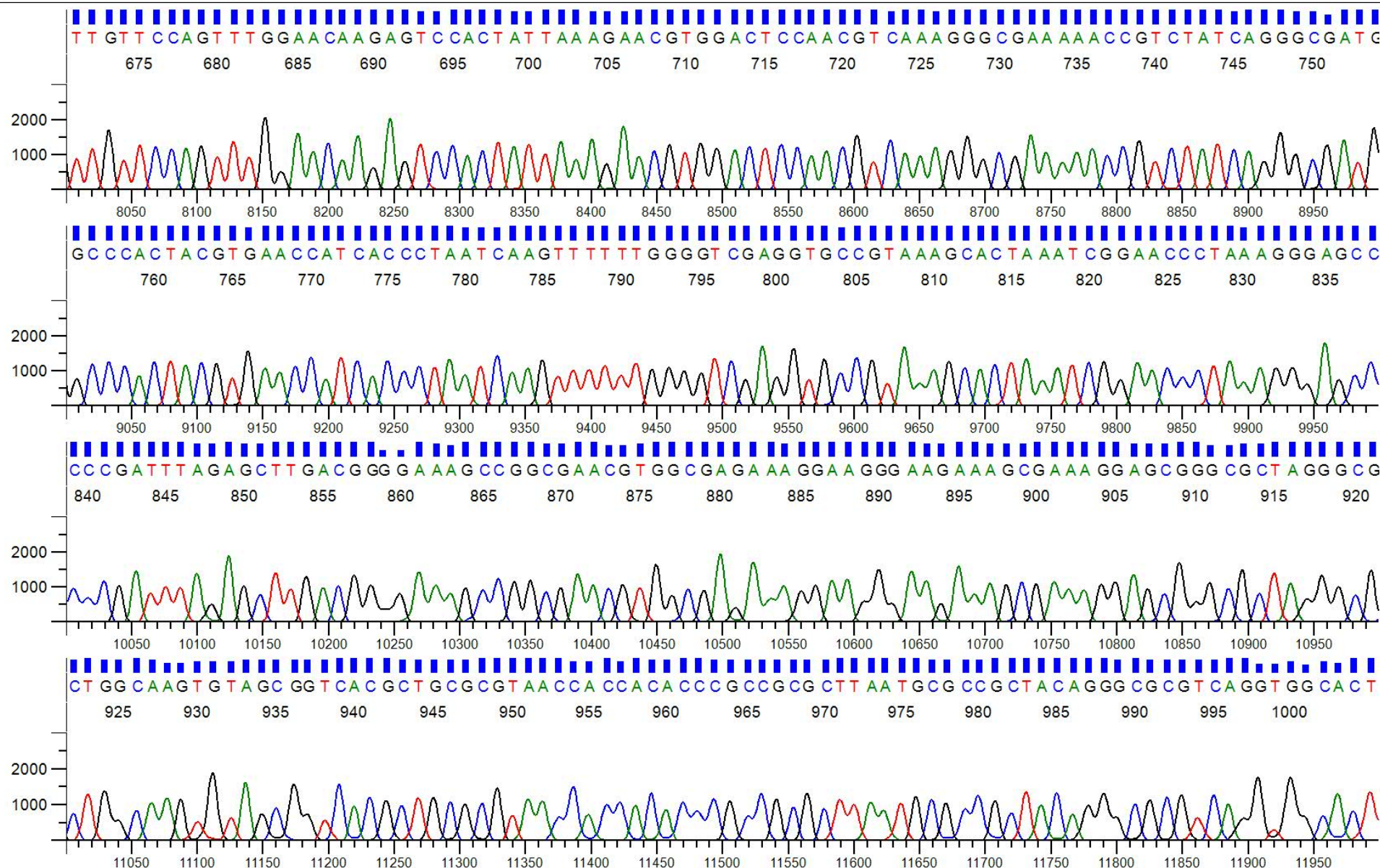

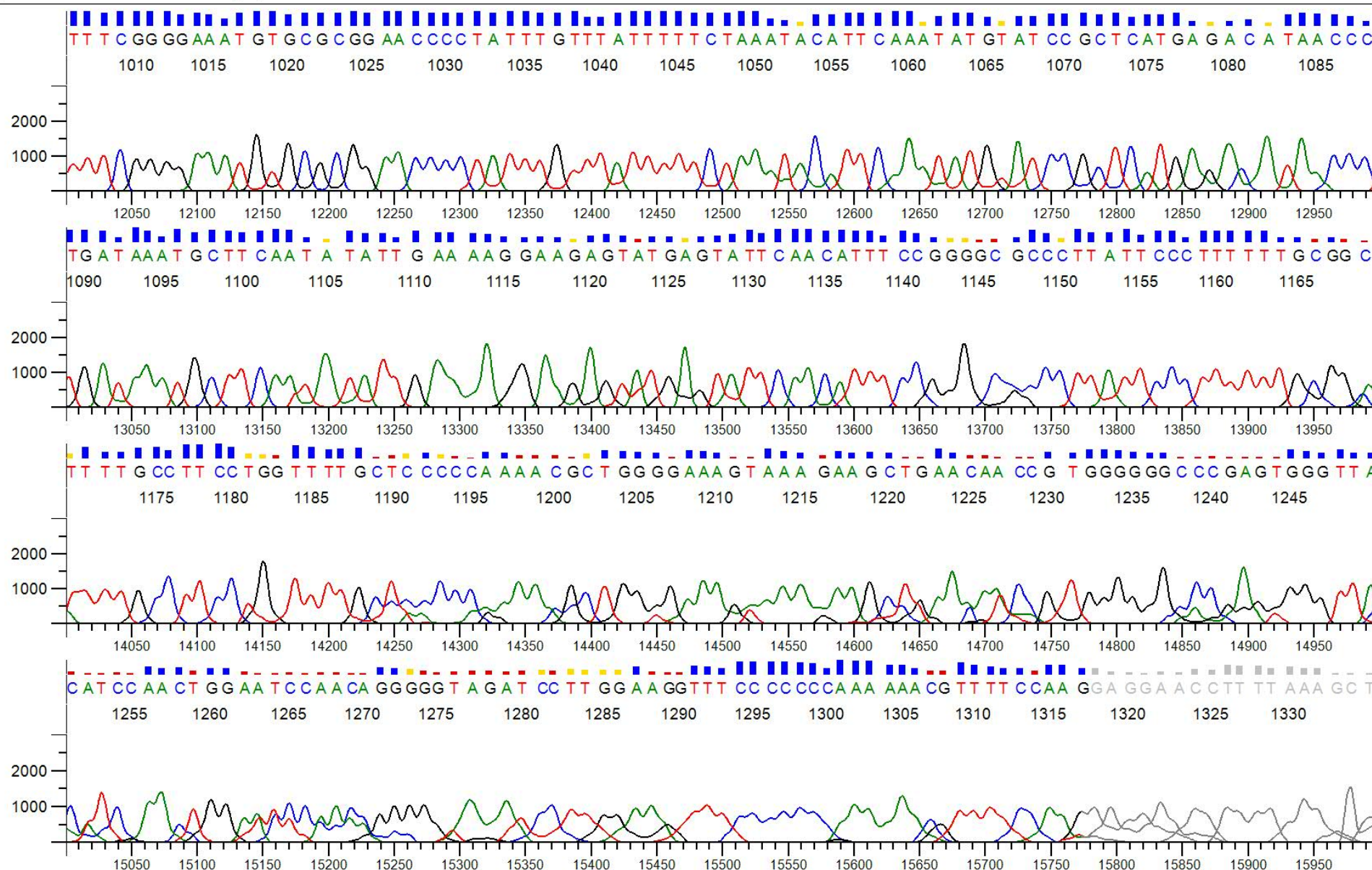

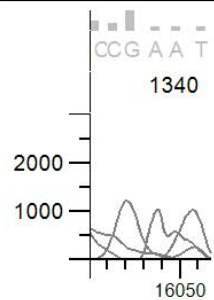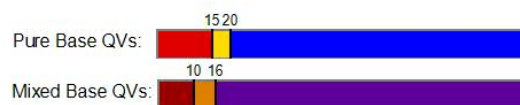

Supplement: Figure 3—source data 2. [file elife-69916-fig3-data2.zip › Figure 3B.C_Source data3_Bisulphite sequencing_mtDNA/SS4-MT-BIS-2.19_T7FOR.pdf]

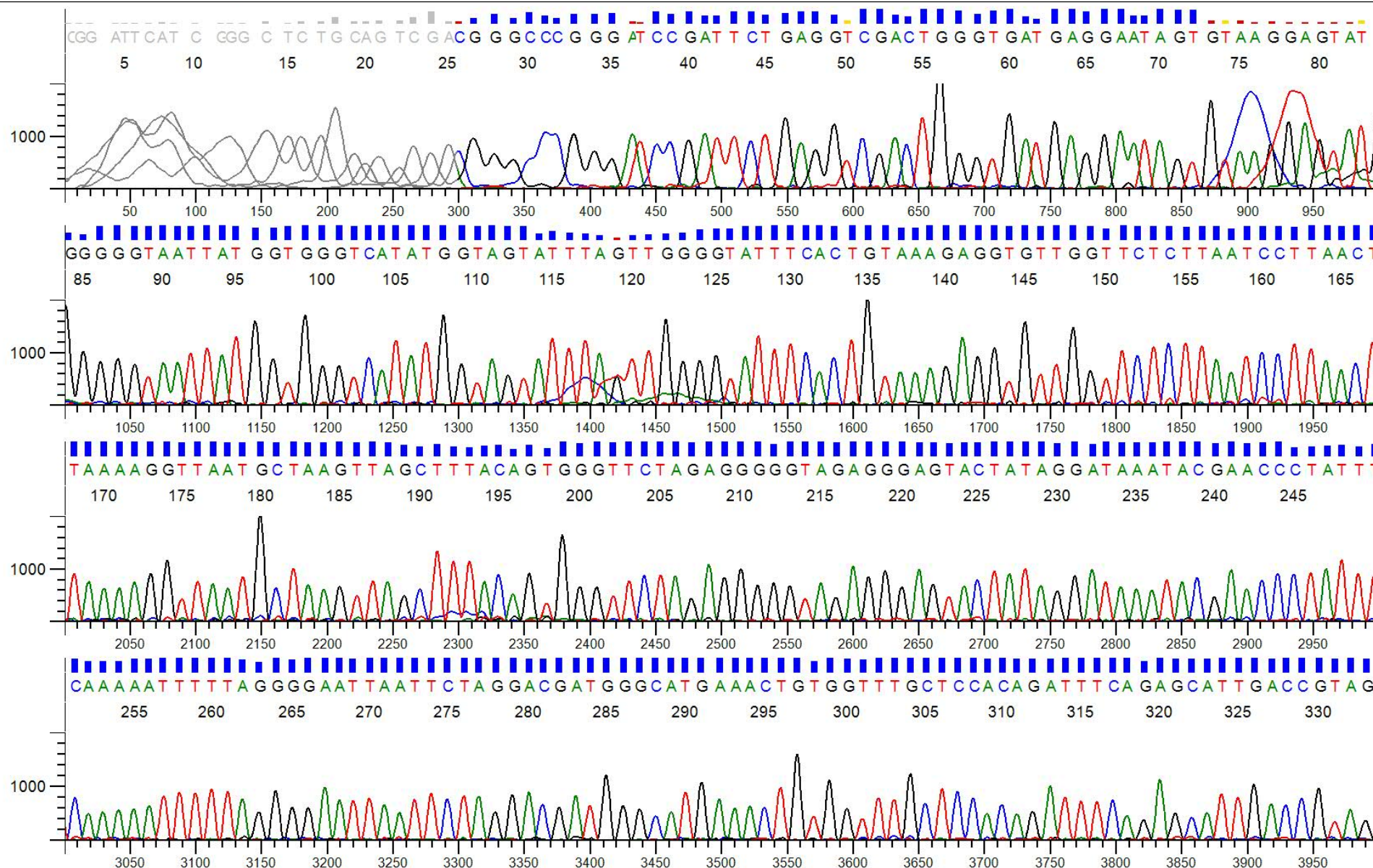

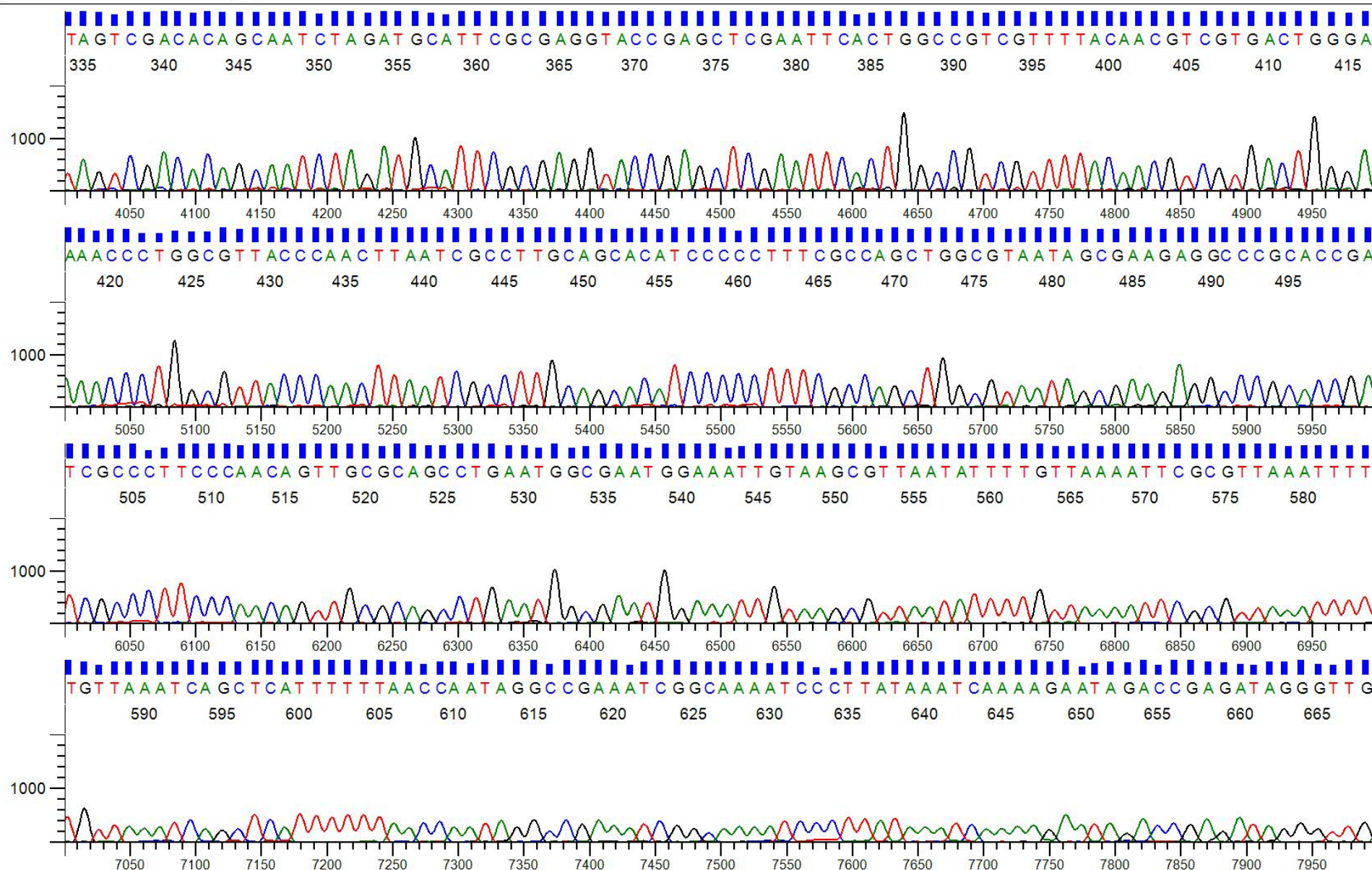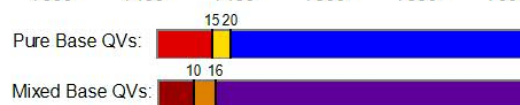

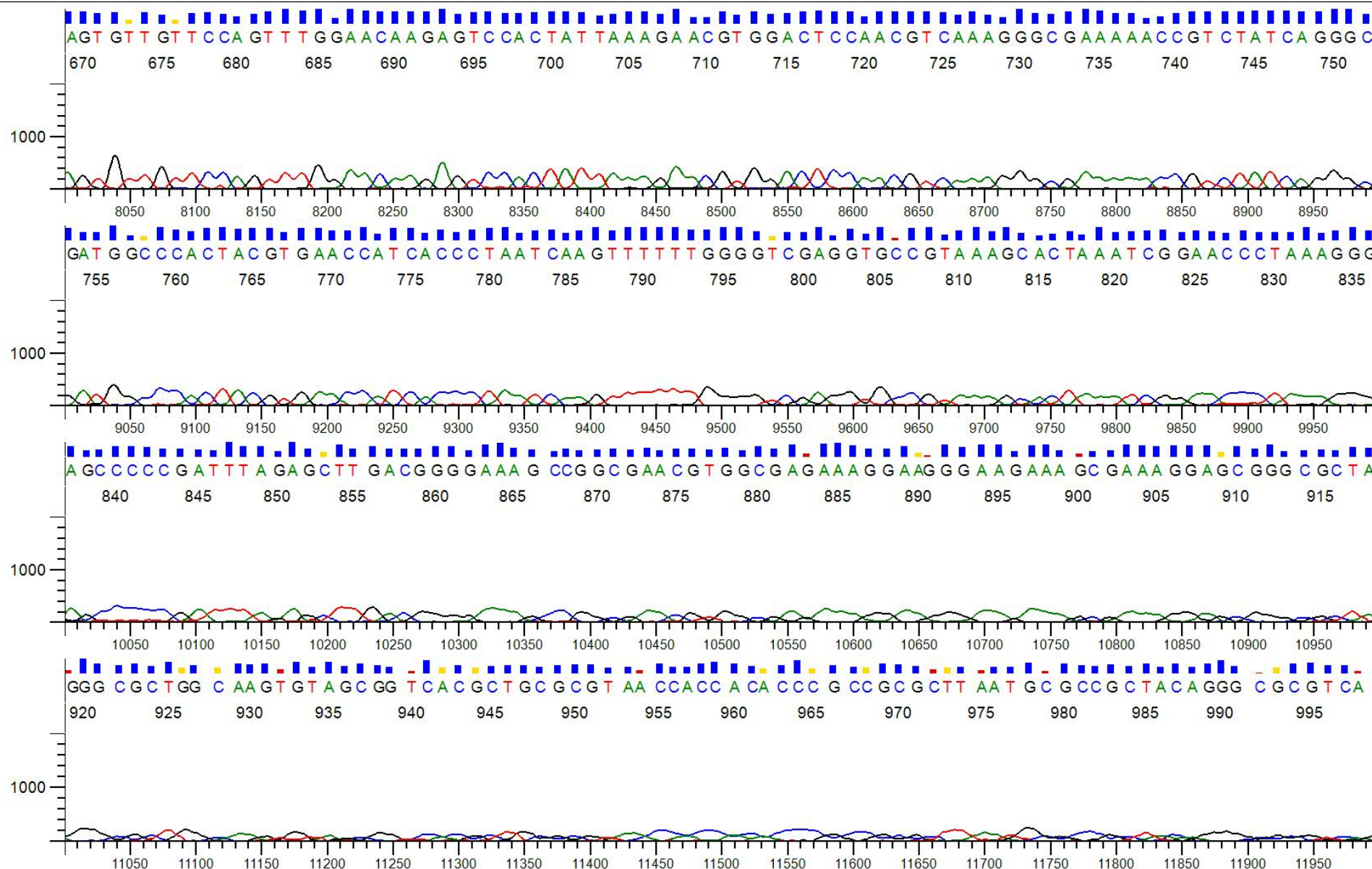

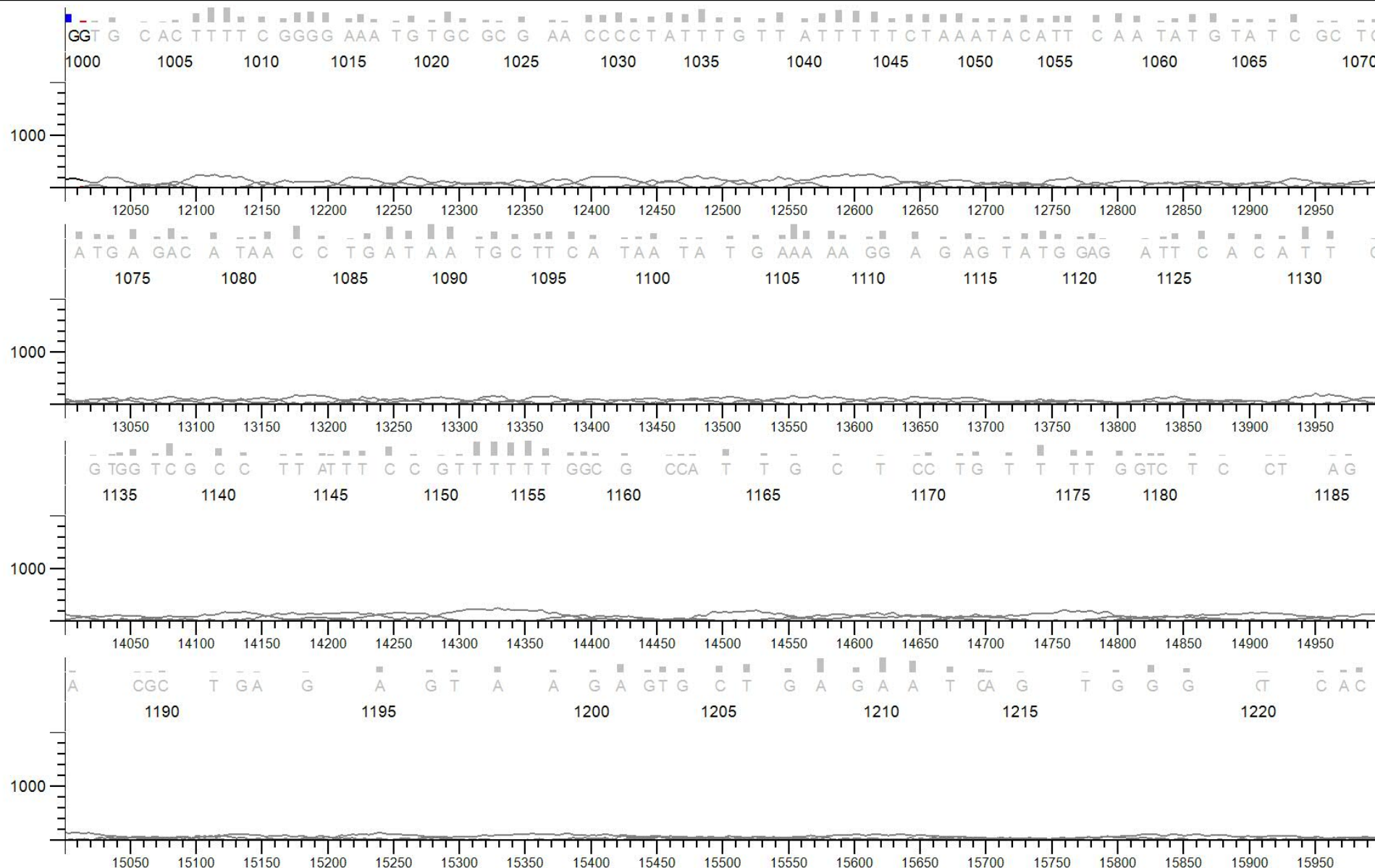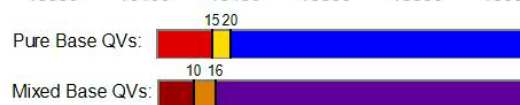

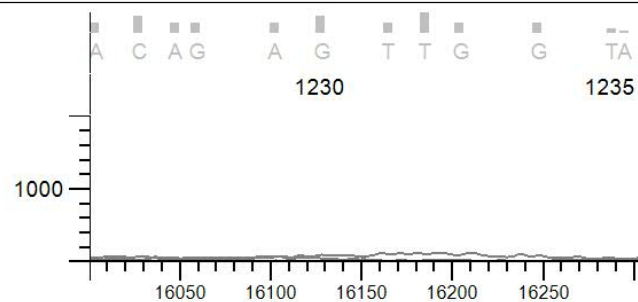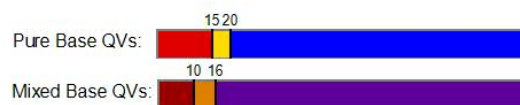

Supplement: Figure 3—source data 2. [file elife-69916-fig3-data2.zip › Figure 3B.C_Source data3_Bisulphite sequencing_mtDNA/SS4-MT-BIS-1.8_T7FOR.pdf]

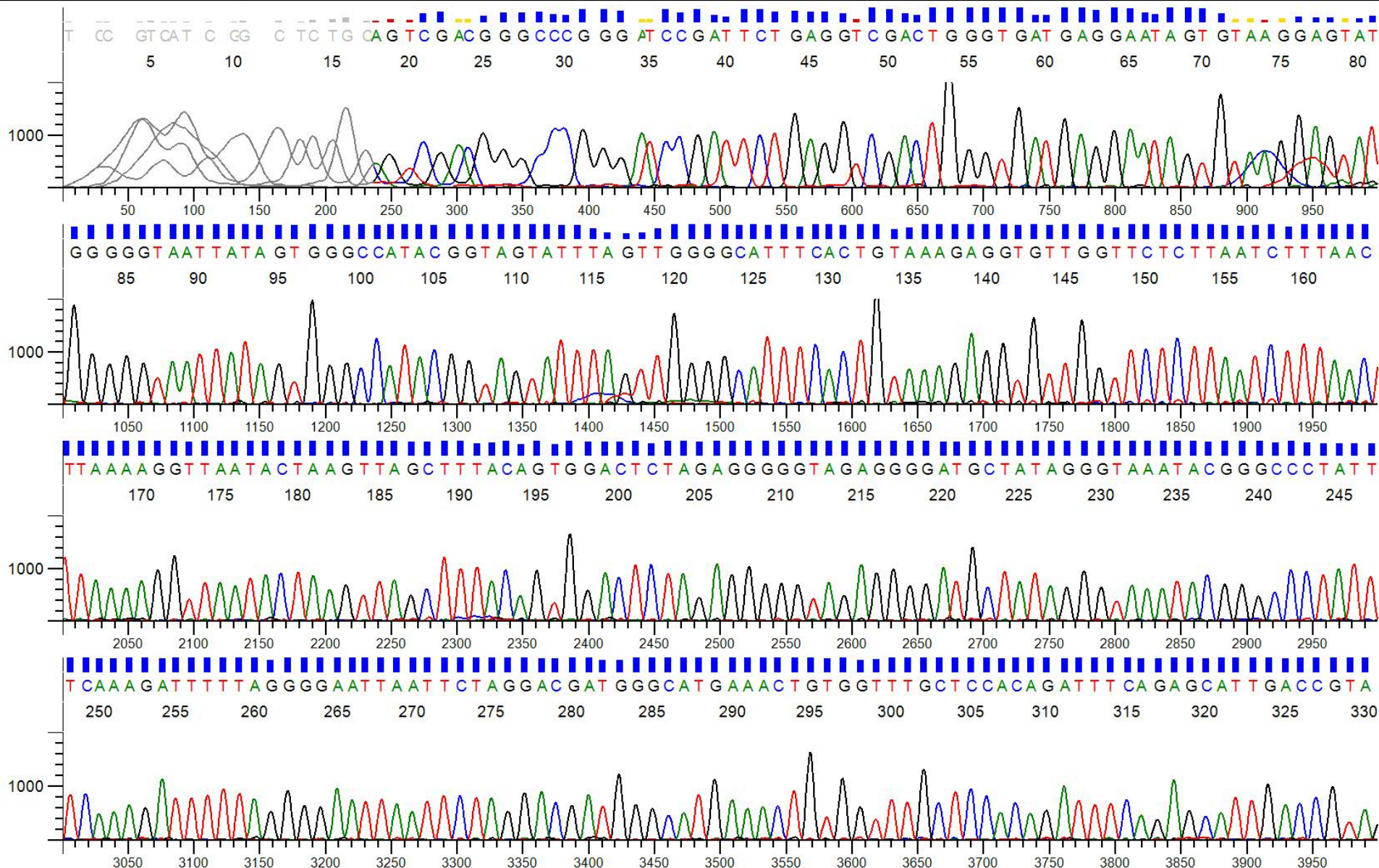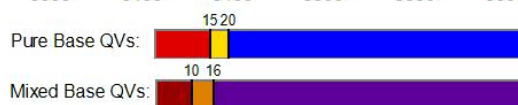

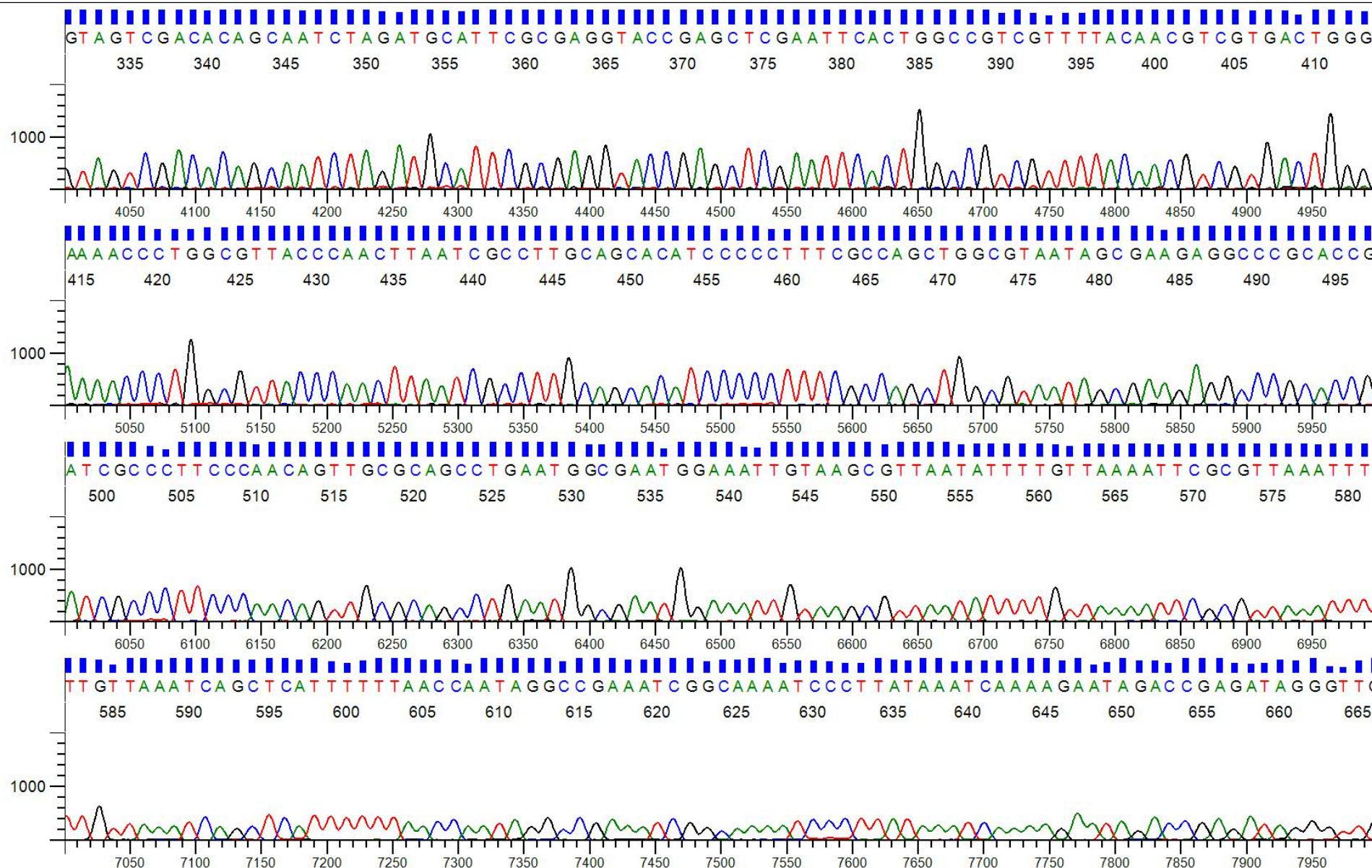

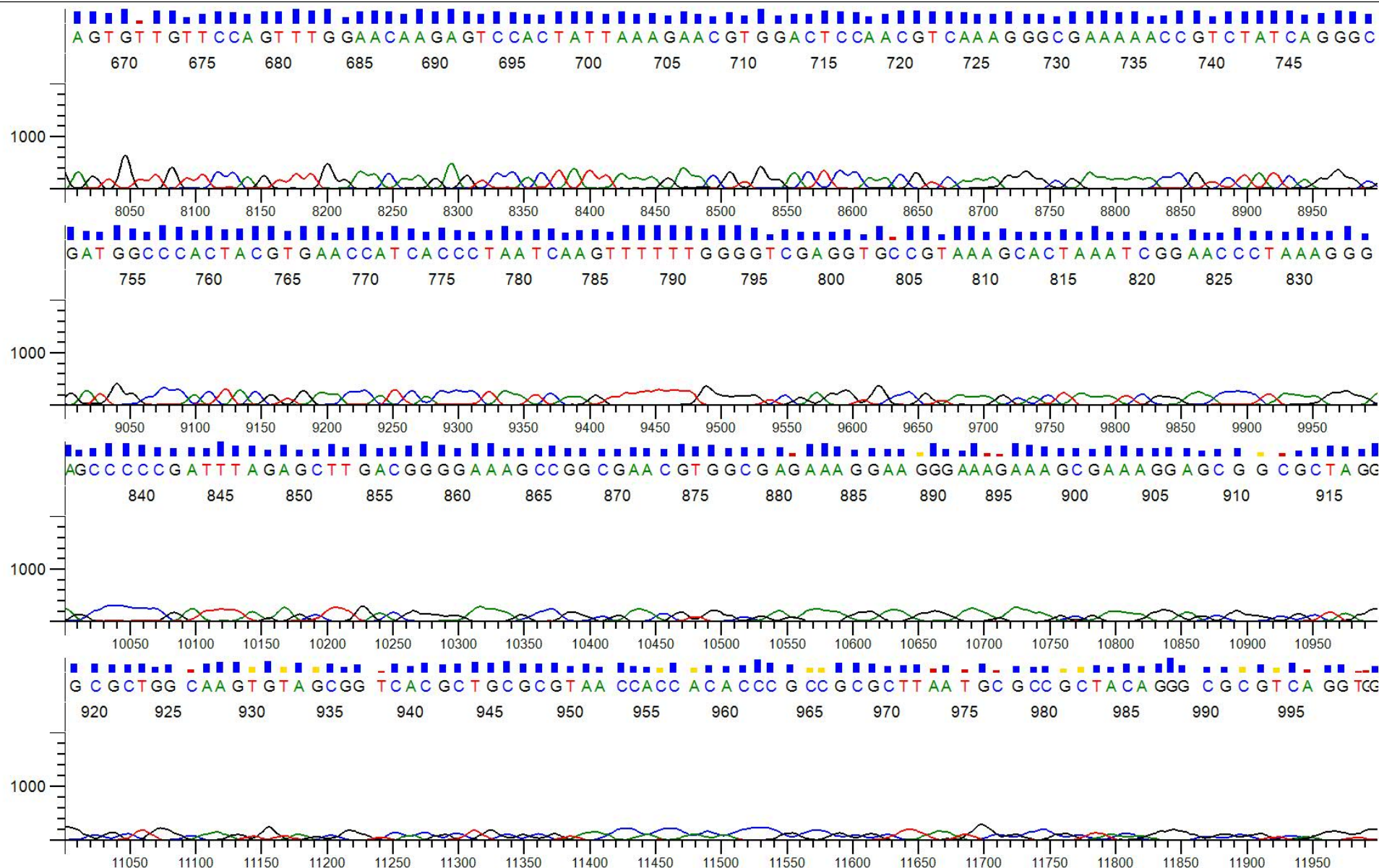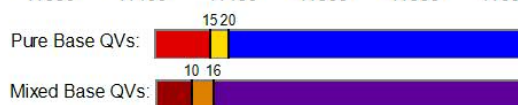

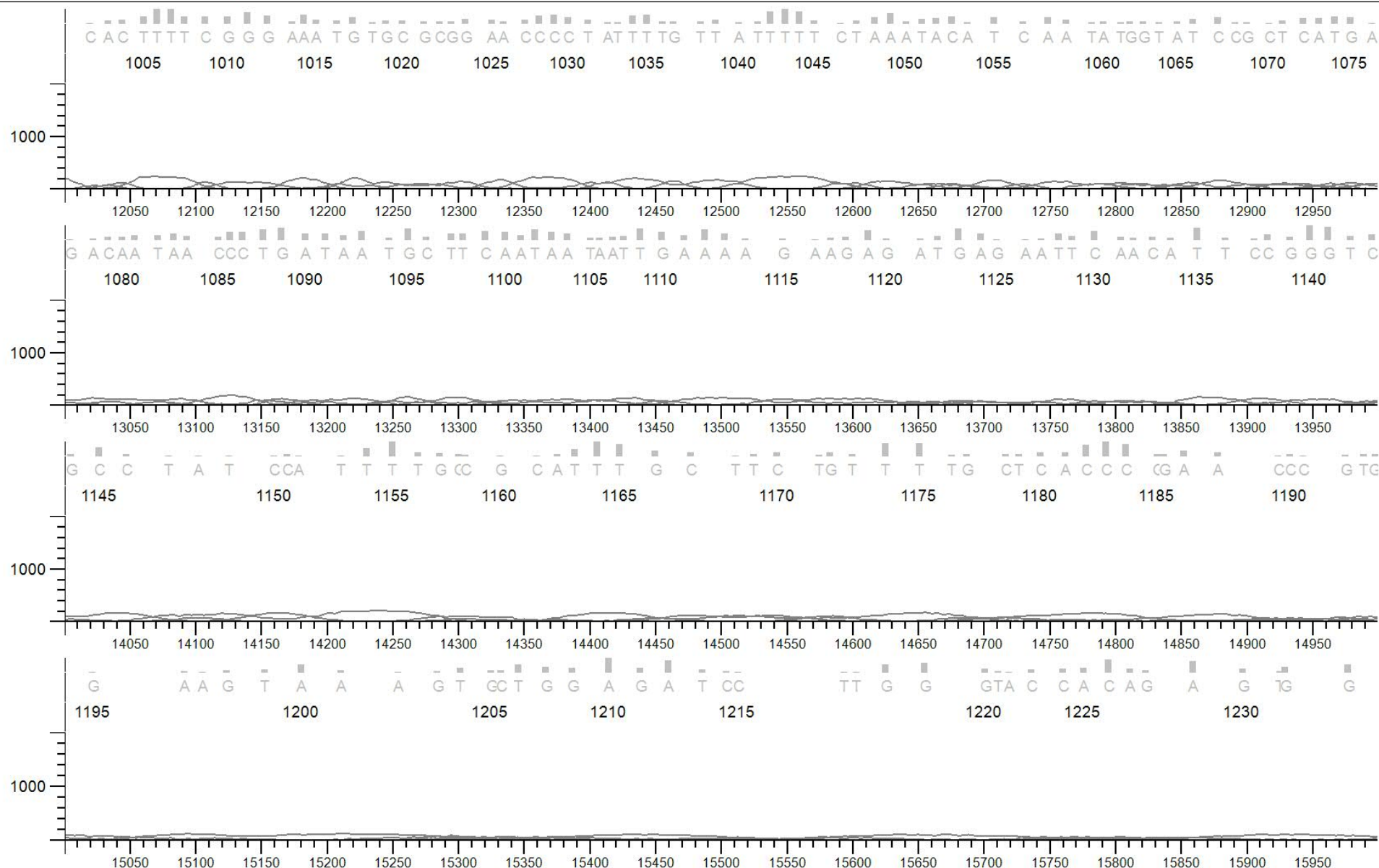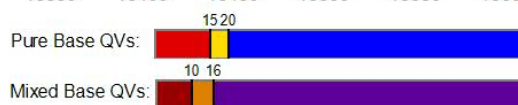

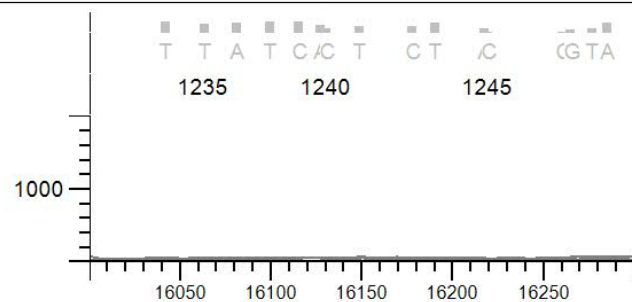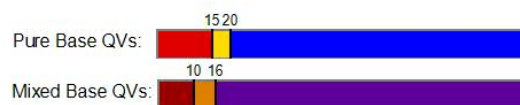

Supplement: Figure 3—source data 2. [file elife-69916-fig3-data2.zip › Figure 3B.C_Source data3_Bisulphite sequencing_mtDNA/SS4-MT-BIS-2.2_T7FOR.pdf]

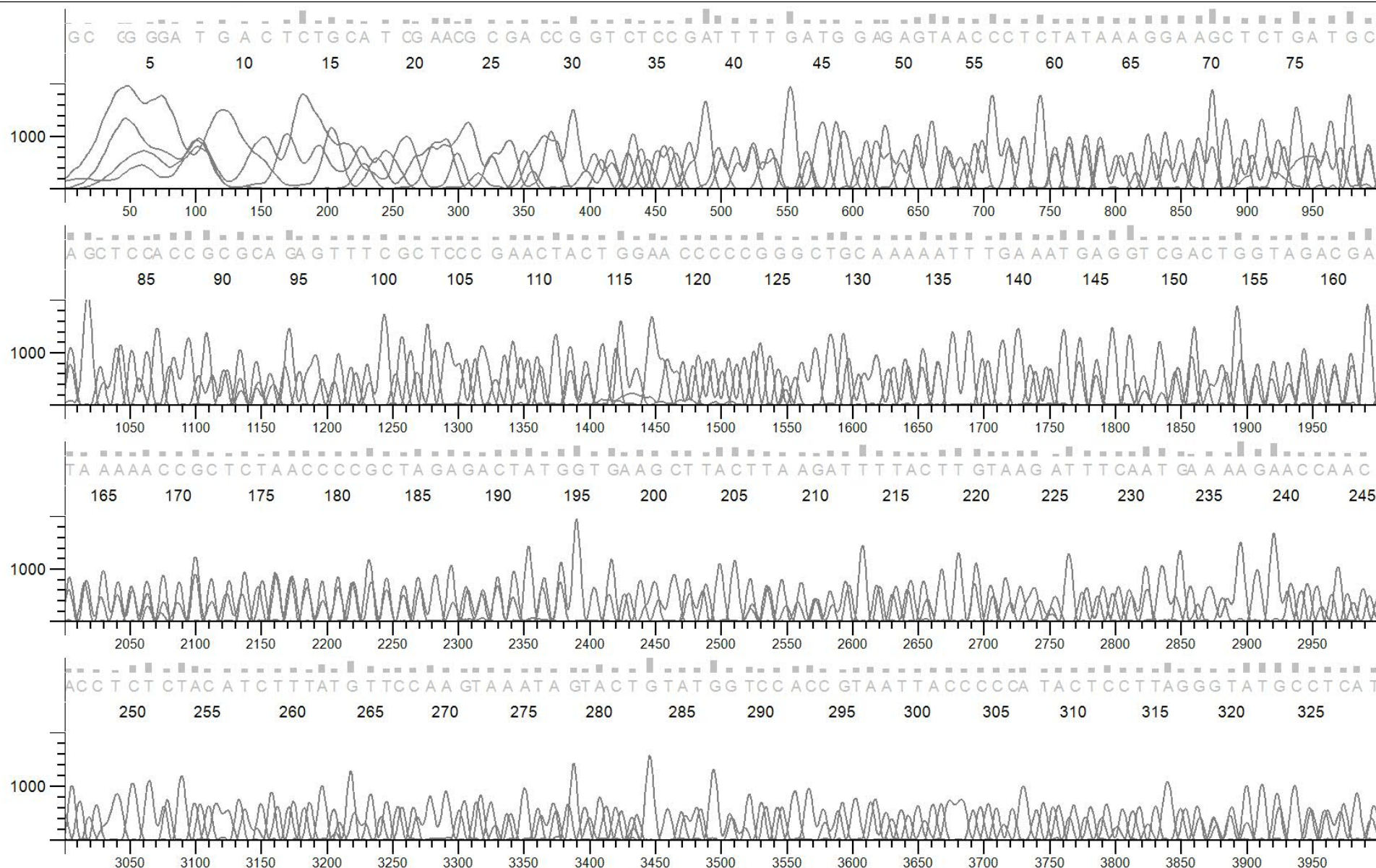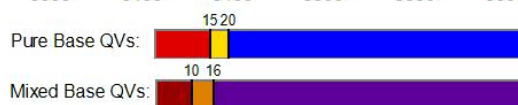

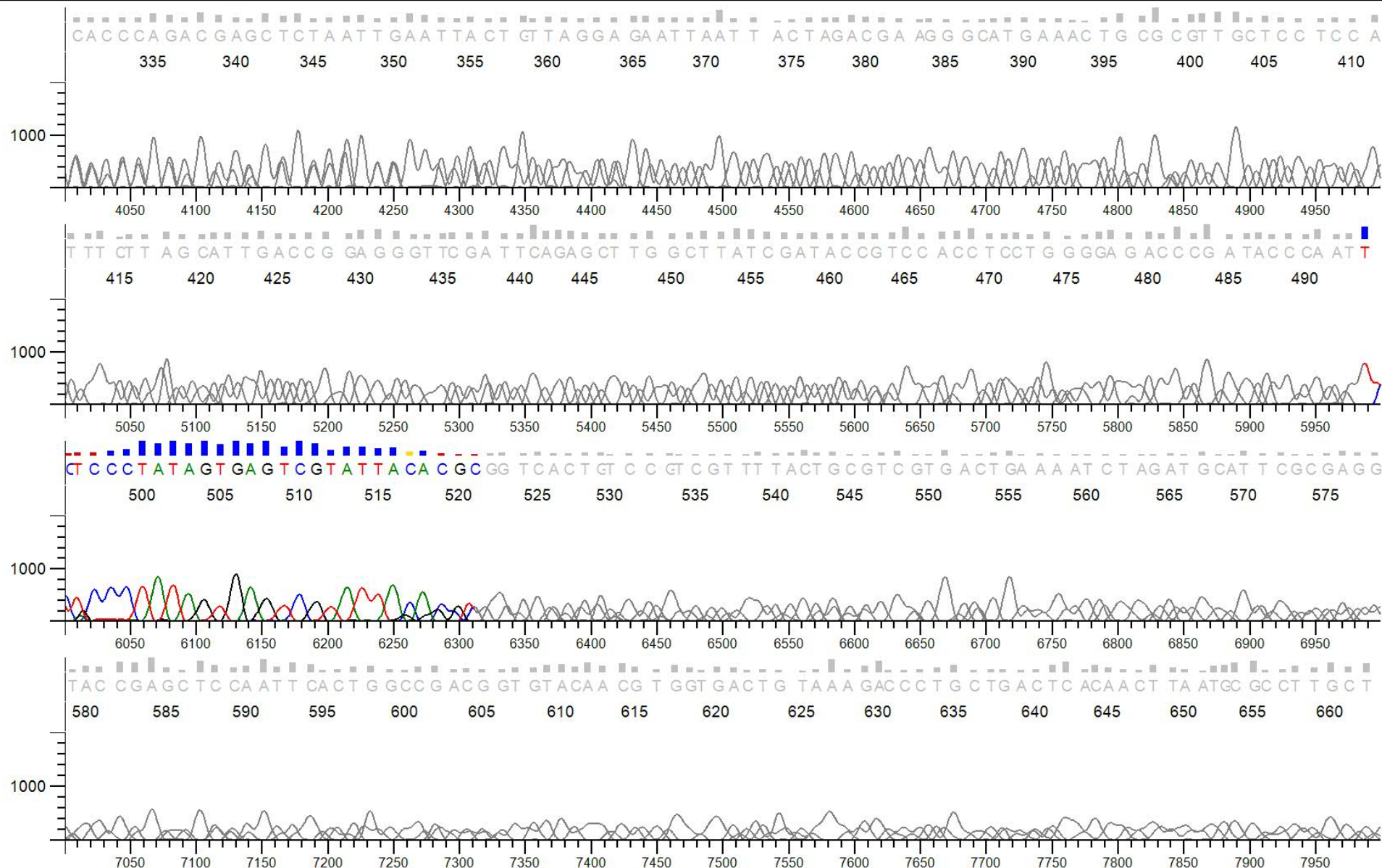

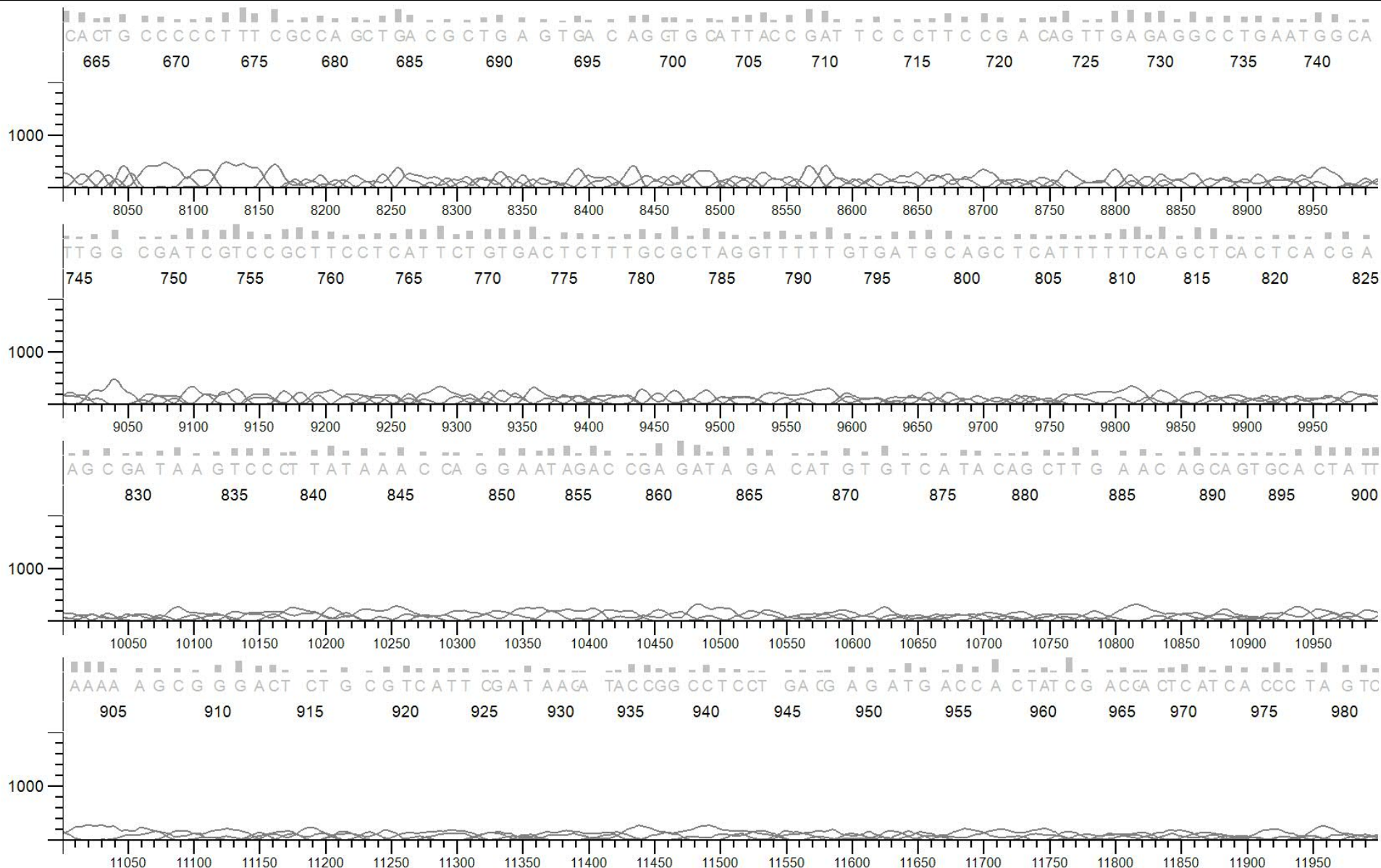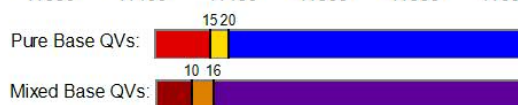

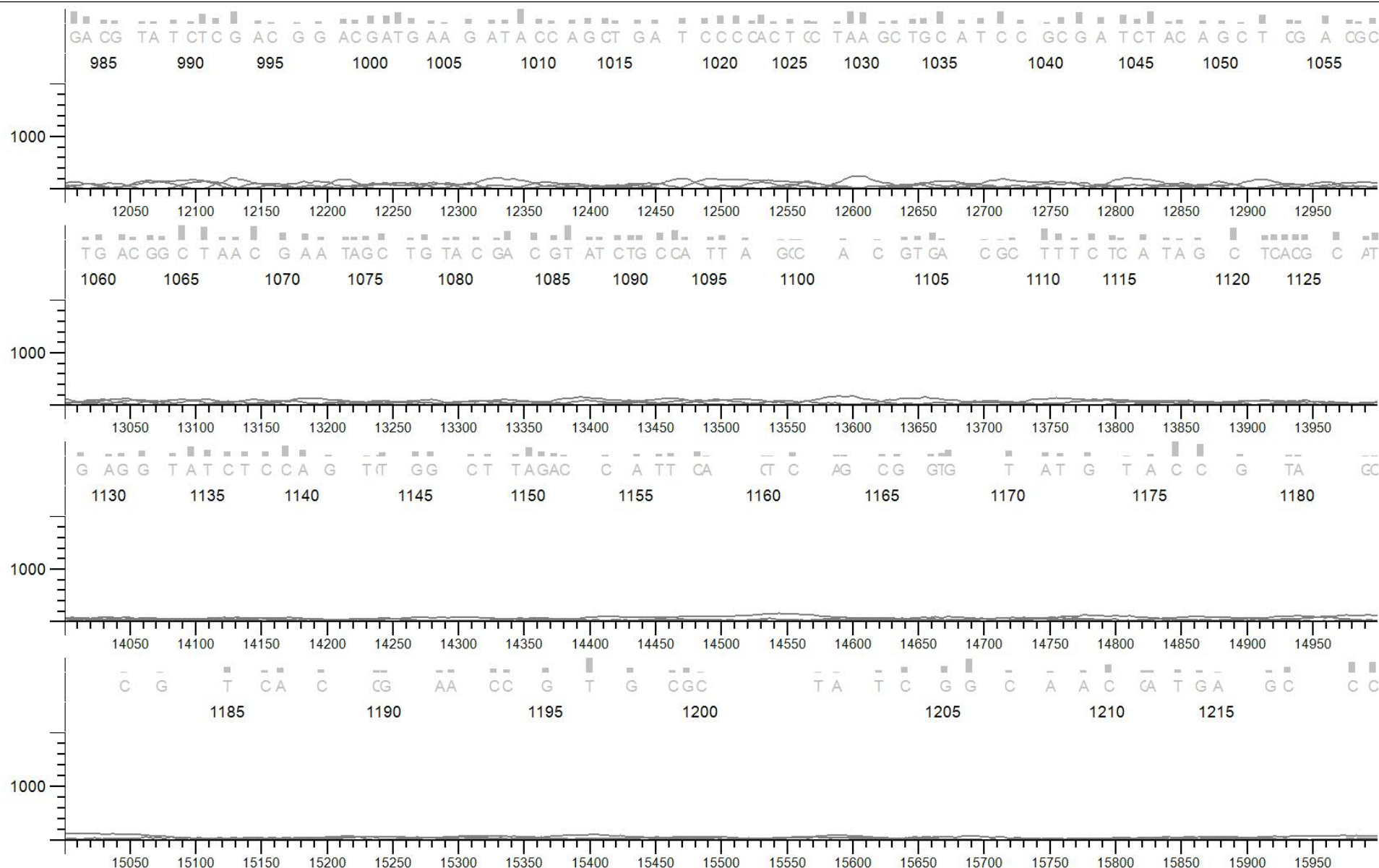

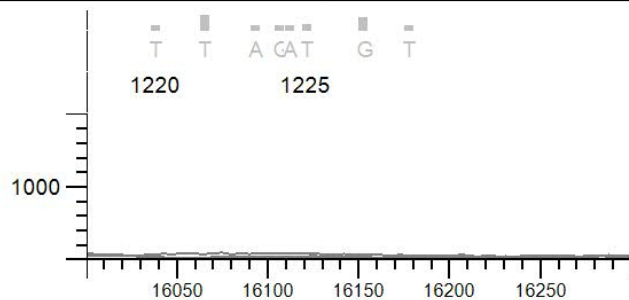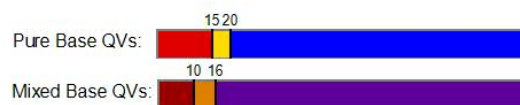

Supplement: Figure 3—source data 2. [file elife-69916-fig3-data2.zip › Figure 3B.C_Source data3_Bisulphite sequencing_mtDNA/SD-MT-DNA-BSF-2.2_T7FOR-G05 .pdf]

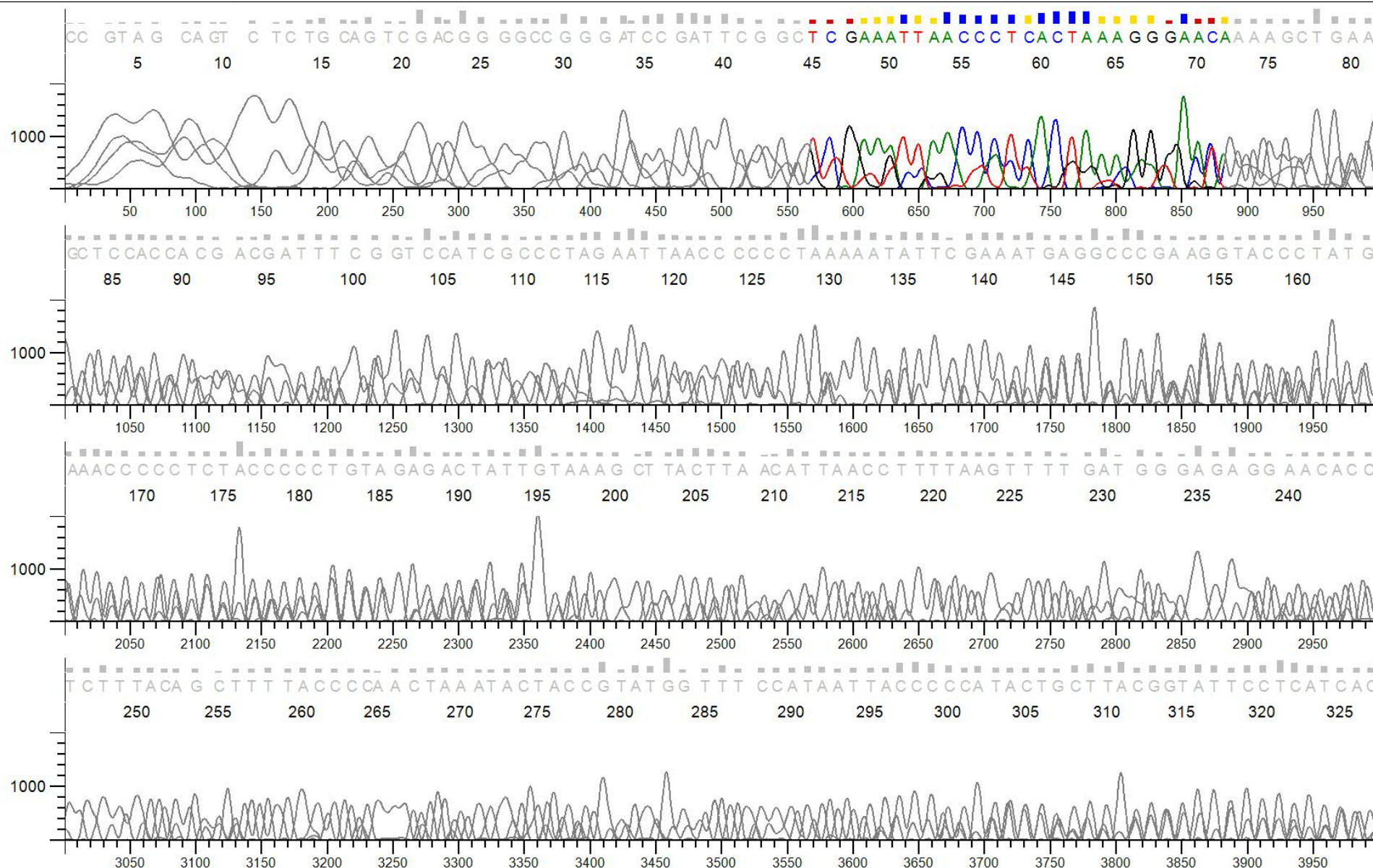

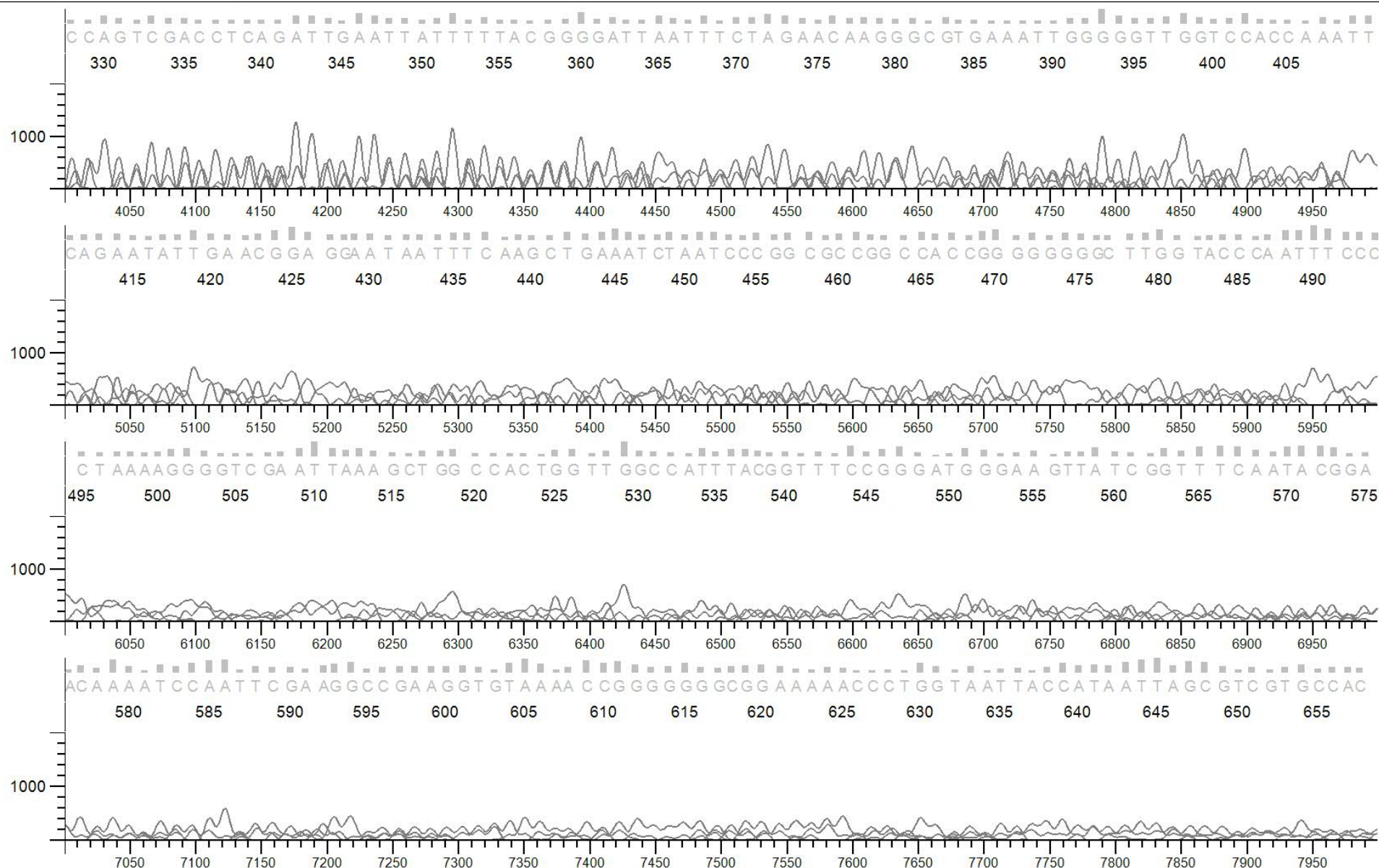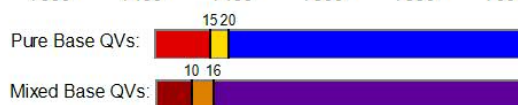

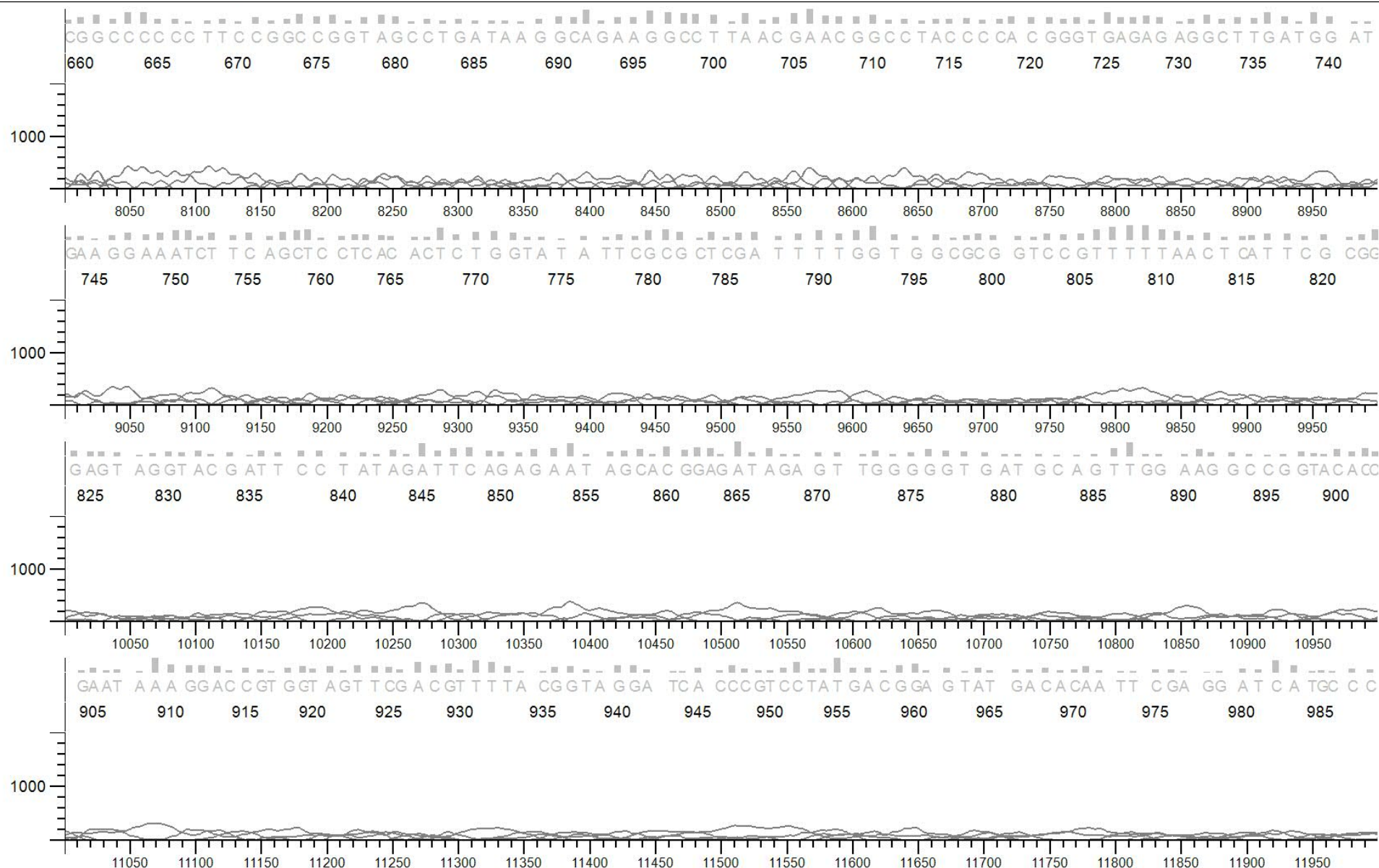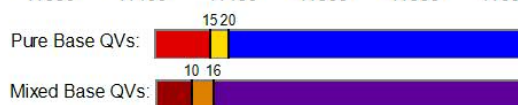

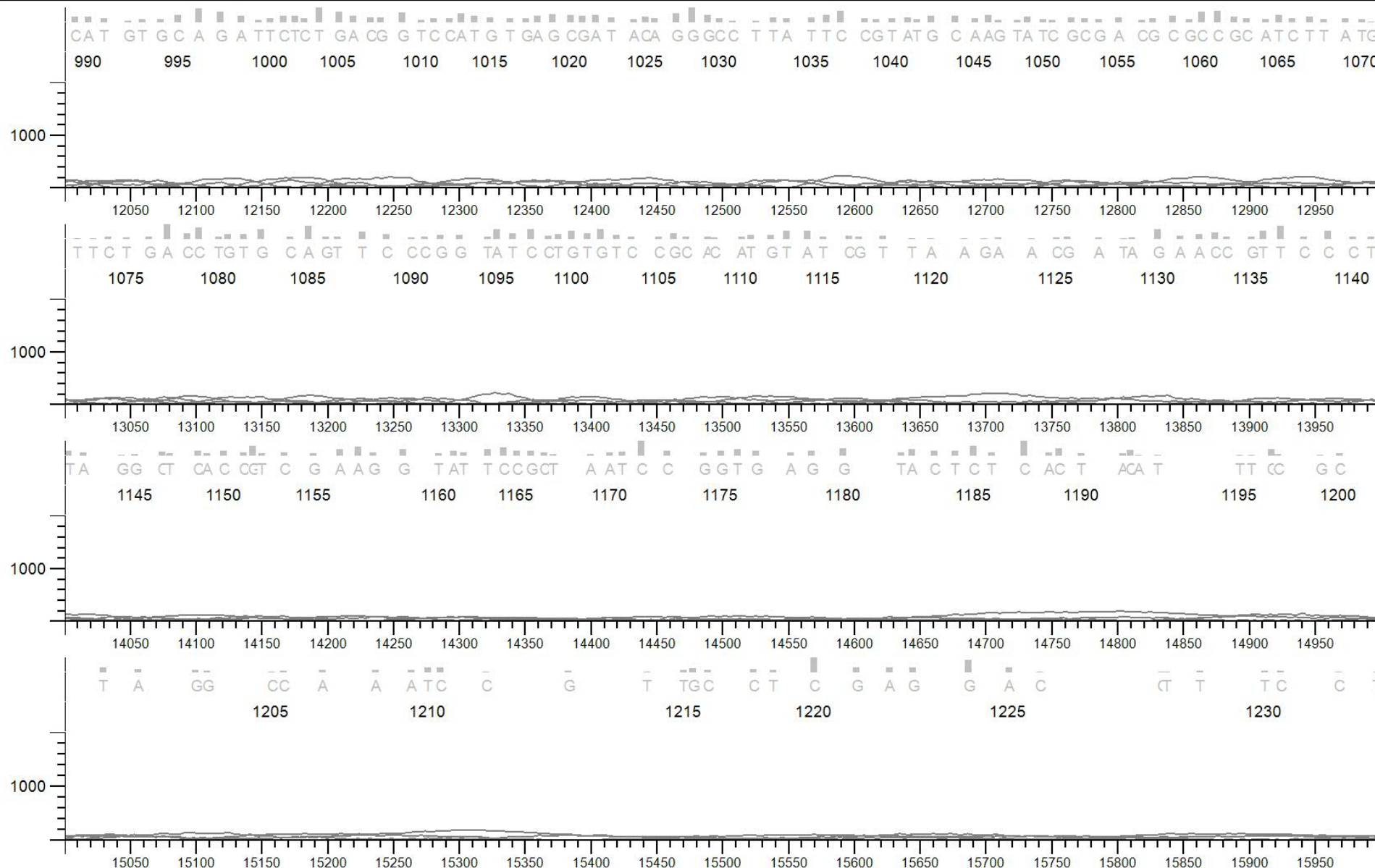

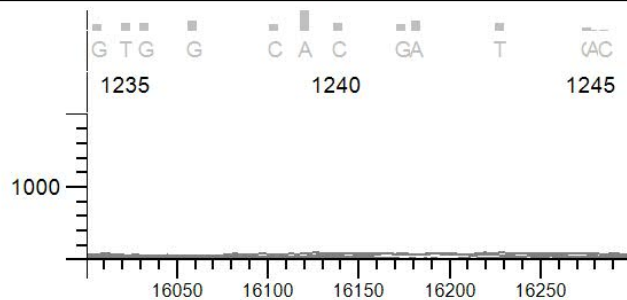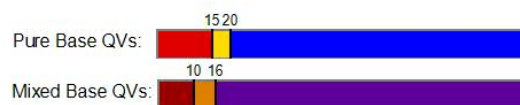

Supplement: Figure 3—source data 2. [file elife-69916-fig3-data2.zip › Figure 3B.C_Source data3_Bisulphite sequencing_mtDNA/SD-MT-DNA-BSF-2.3_T7FOR-D05.pdf]

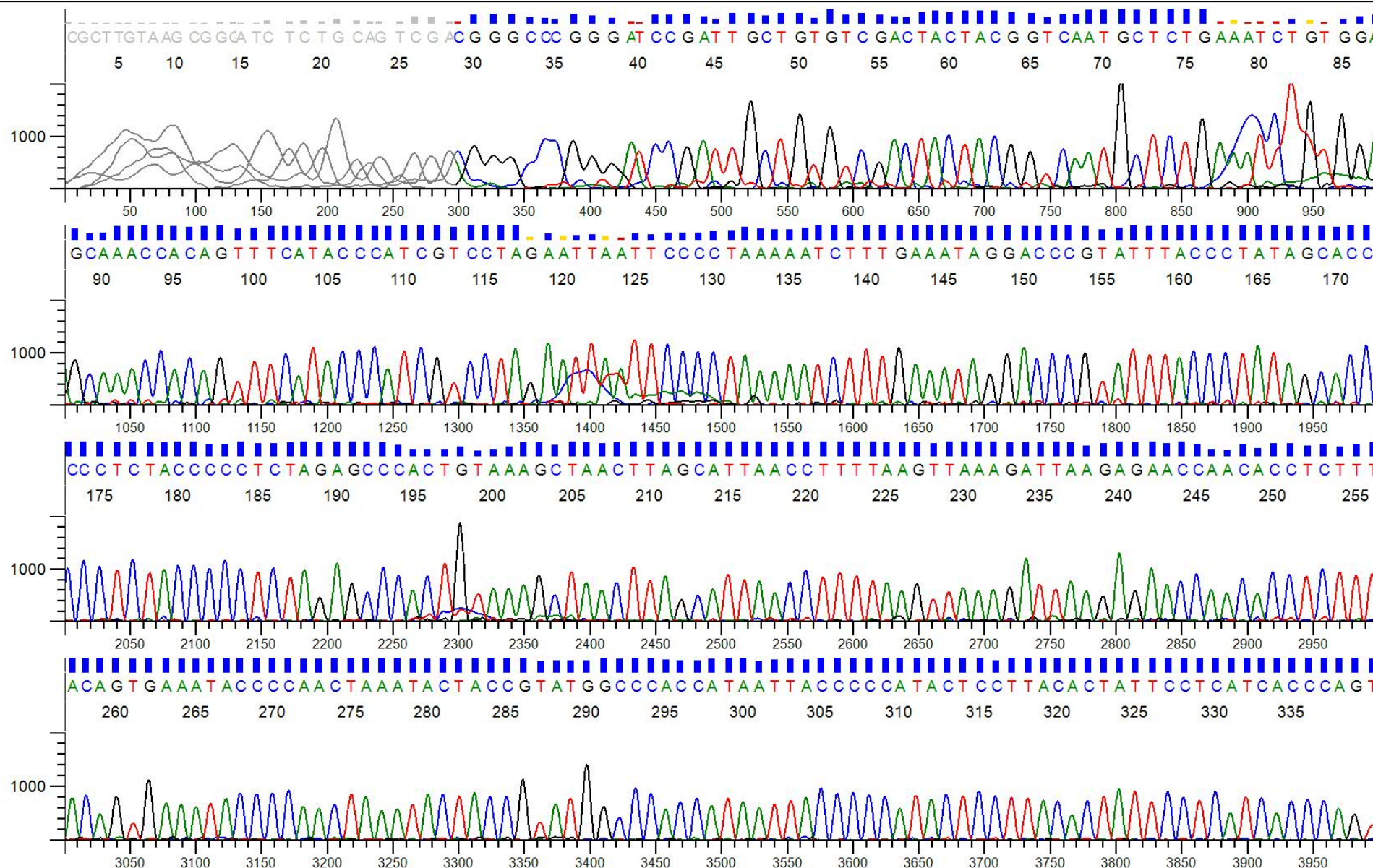

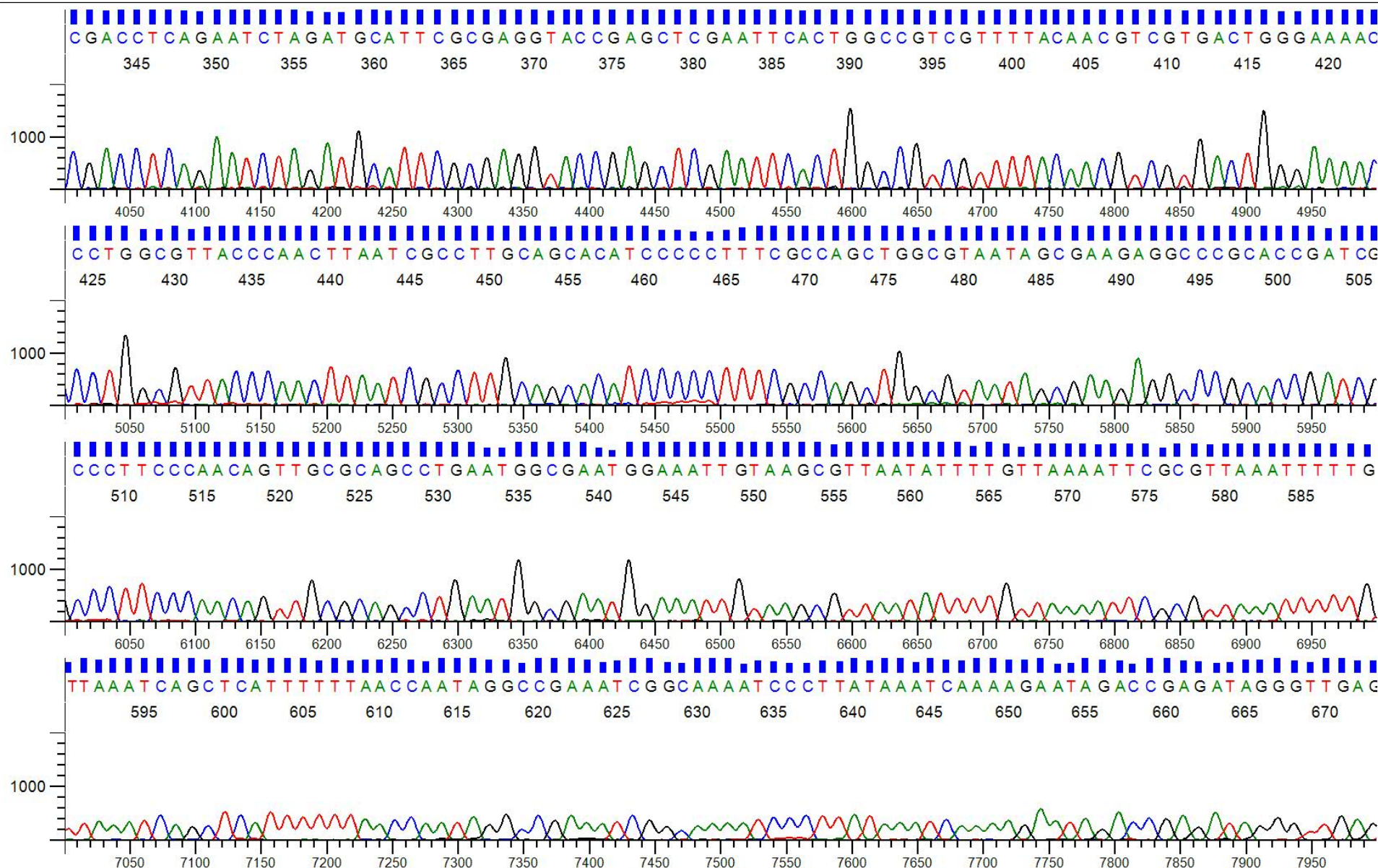

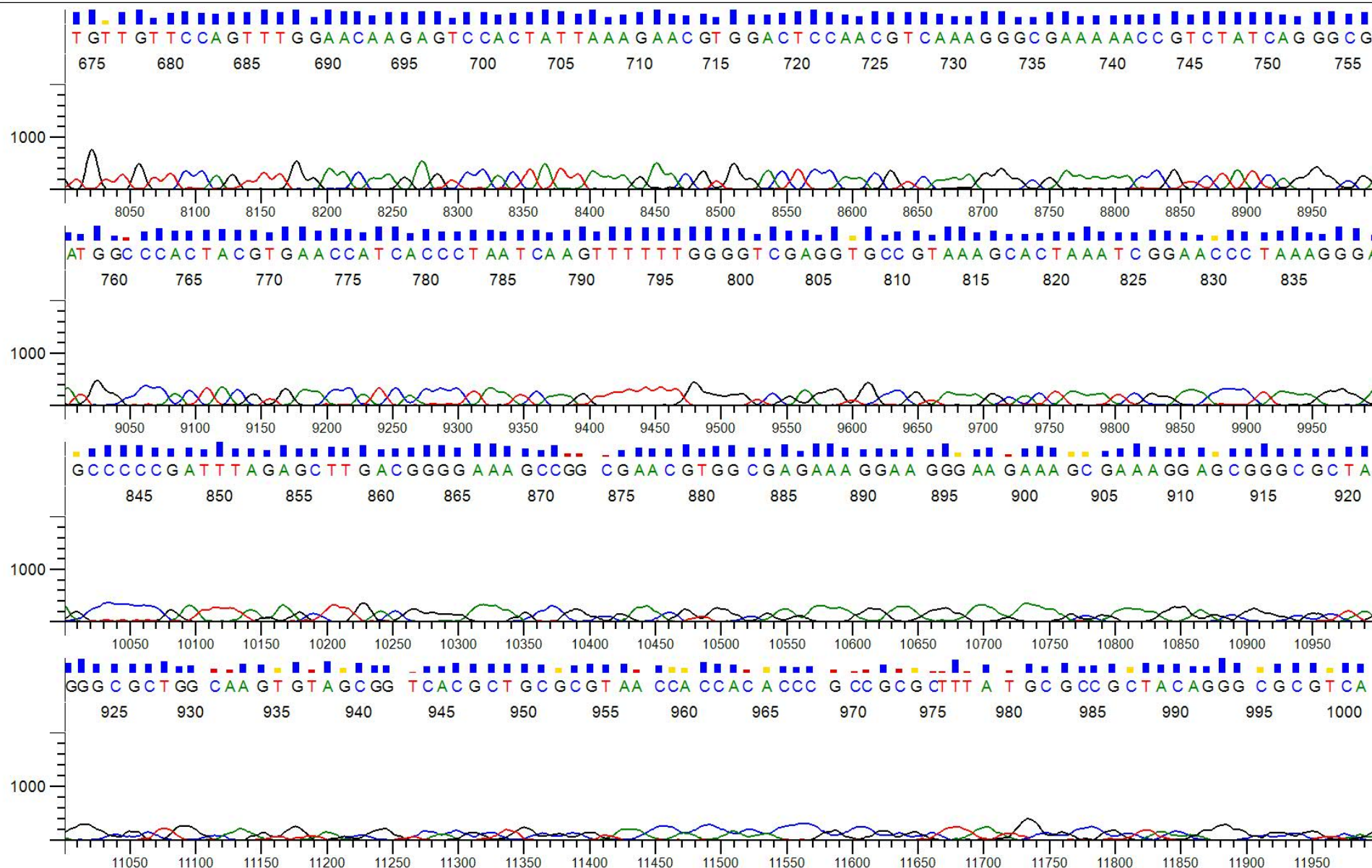

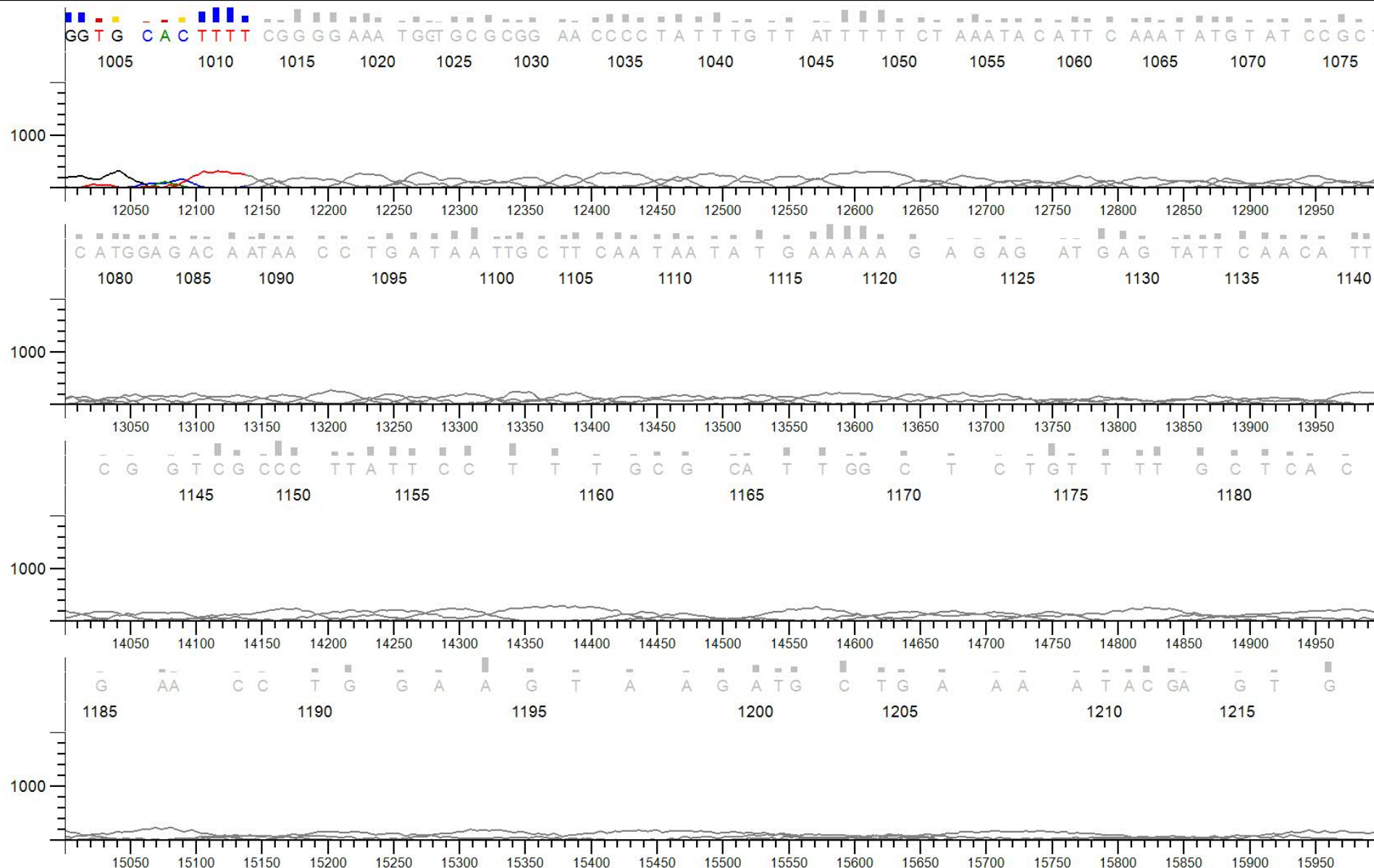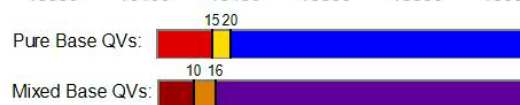

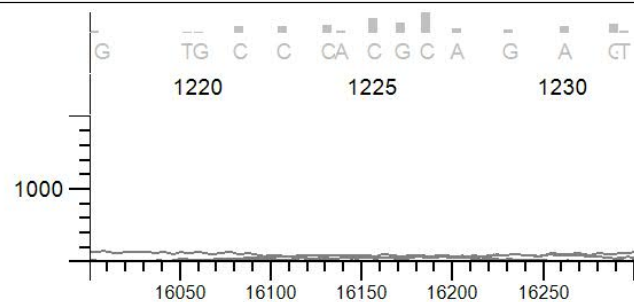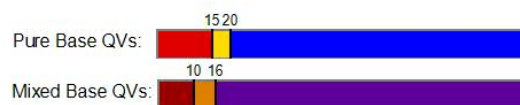

Supplement: Figure 3—source data 2. [file elife-69916-fig3-data2.zip › Figure 3B.C_Source data3_Bisulphite sequencing_mtDNA/SS4-MT-BIS-2.15_T7FOR.pdf]

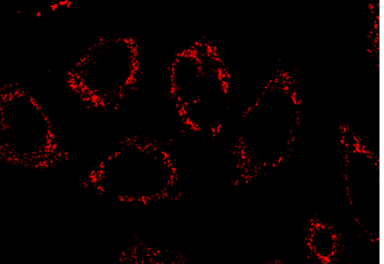

Supplement: Figure 4—source data 1. [file elife-69916-fig4-data1.zip › Figure4_Source data_BG4 localization to mtDNA/Figure 4A_Representative image_293T and HeLa_BG4_mitotracker red/Figure 4A_Representative image_HeLa_BG4_mtDR Red.tif]

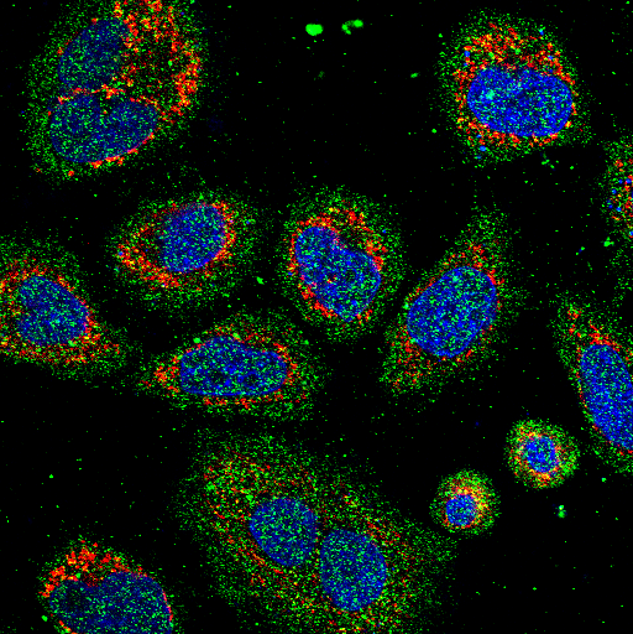

Supplement: Figure 4—source data 1. [file elife-69916-fig4-data1.zip › Figure4_Source data_BG4 localization to mtDNA/Figure 4A_Representative image_293T and HeLa_BG4_mitotracker red/Figure 4A_Representative image_293T_BG4_mitochondria.tif]

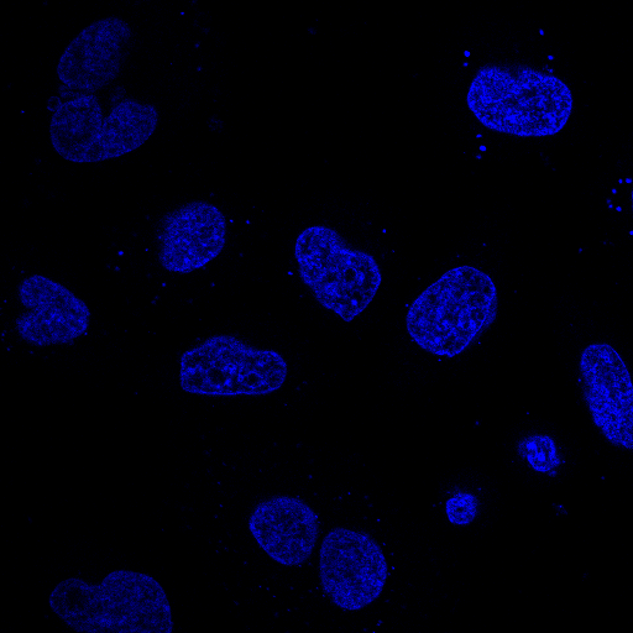

Supplement: Figure 4—source data 1. [file elife-69916-fig4-data1.zip › Figure4_Source data_BG4 localization to mtDNA/Figure 4A_Representative image_293T and HeLa_BG4_mitotracker red/Figure 2A_Representative image_293T_BG4_mitochondria_2.tif]

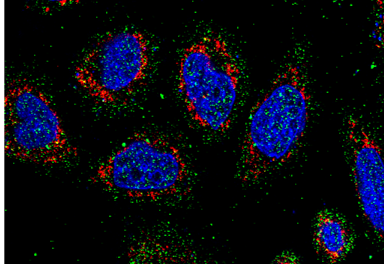

Supplement: Figure 4—source data 1. [file elife-69916-fig4-data1.zip › Figure4_Source data_BG4 localization to mtDNA/Figure 4A_Representative image_293T and HeLa_BG4_mitotracker red/Figure 4A_Representative image_HeLa_BG4_merged.tif]

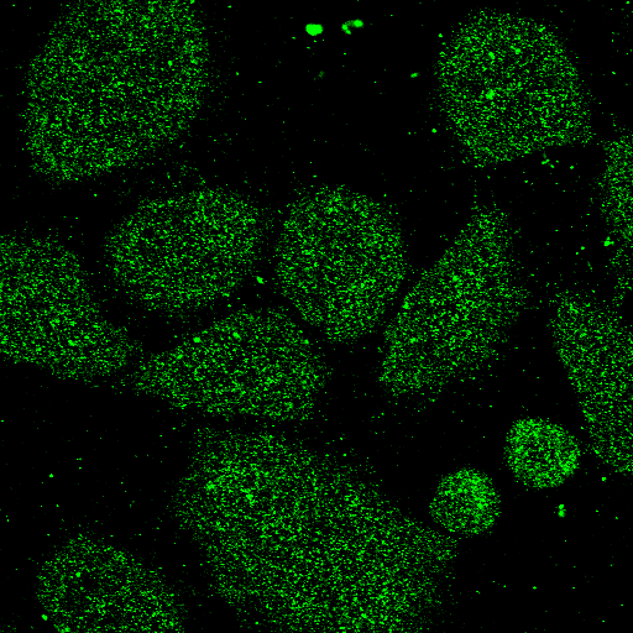

Supplement: Figure 4—source data 1. [file elife-69916-fig4-data1.zip › Figure4_Source data_BG4 localization to mtDNA/Figure 4A_Representative image_293T and HeLa_BG4_mitotracker red/Figure 4A_Representative image_293T_BG4_mitochondria_1.tif]

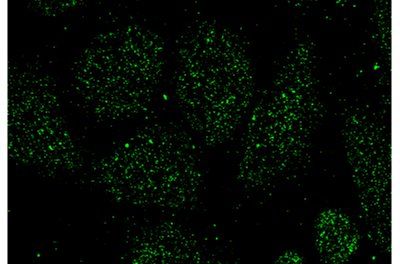

Supplement: Figure 4—source data 1. [file elife-69916-fig4-data1.zip › Figure4_Source data_BG4 localization to mtDNA/Figure 4A_Representative image_293T and HeLa_BG4_mitotracker red/Figure 4A_Representative image_HeLa_BG4_BG4 stain.tif]

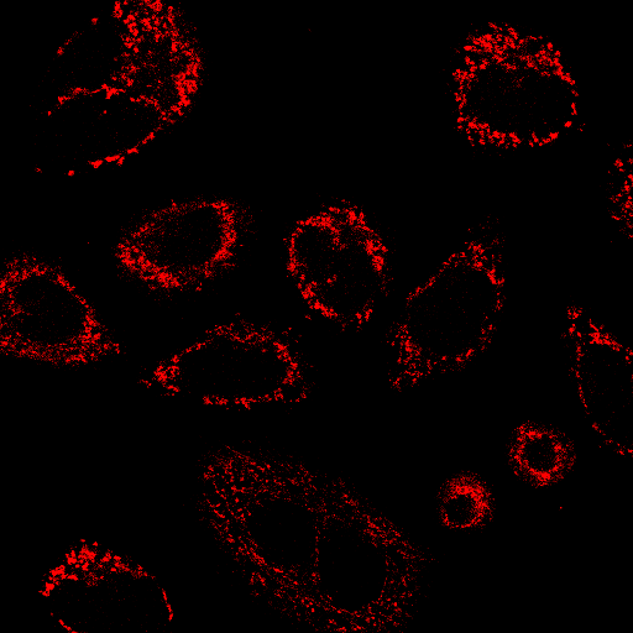

Supplement: Figure 4—source data 1. [file elife-69916-fig4-data1.zip › Figure4_Source data_BG4 localization to mtDNA/Figure 4A_Representative image_293T and HeLa_BG4_mitotracker red/Figure 4A_Representative image_293T_BG4_mitochondria_3.tif]

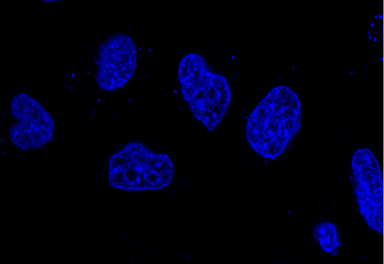

Supplement: Figure 4—source data 1. [file elife-69916-fig4-data1.zip › Figure4_Source data_BG4 localization to mtDNA/Figure 4A_Representative image_293T and HeLa_BG4_mitotracker red/Figure 4A_Representative image_HeLa_BG4_DAPI.tif]

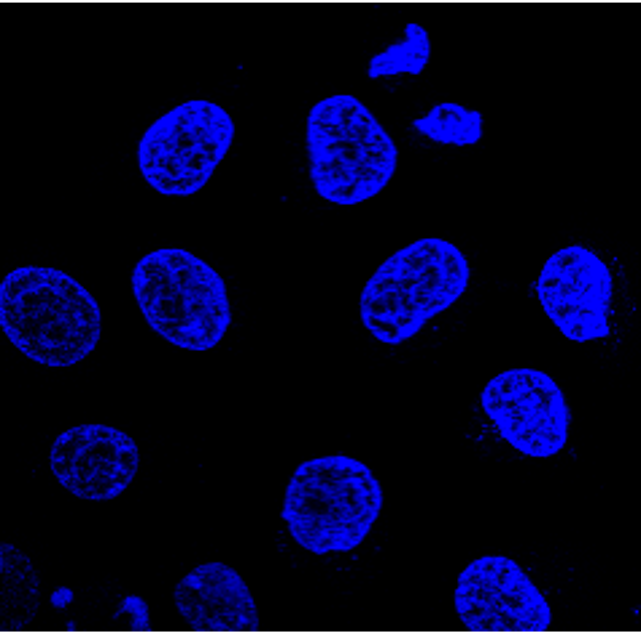

Supplement: Figure 4—source data 1. [file elife-69916-fig4-data1.zip › Figure4_Source data_BG4 localization to mtDNA/Figure 4D_Representative image_rho cells_BG4_mitotracker red/Figure 4D_Representative image_rho cells_BG4_mito tracker red_nuclear stain.tif]

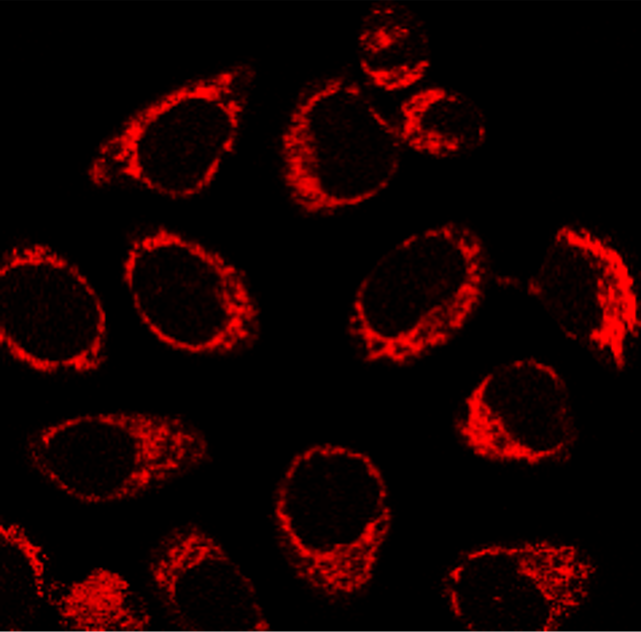

Supplement: Figure 4—source data 1. [file elife-69916-fig4-data1.zip › Figure4_Source data_BG4 localization to mtDNA/Figure 4D_Representative image_rho cells_BG4_mitotracker red/Figure 4D_Representative image_rho cells_BG4_mito tracker red.tif]

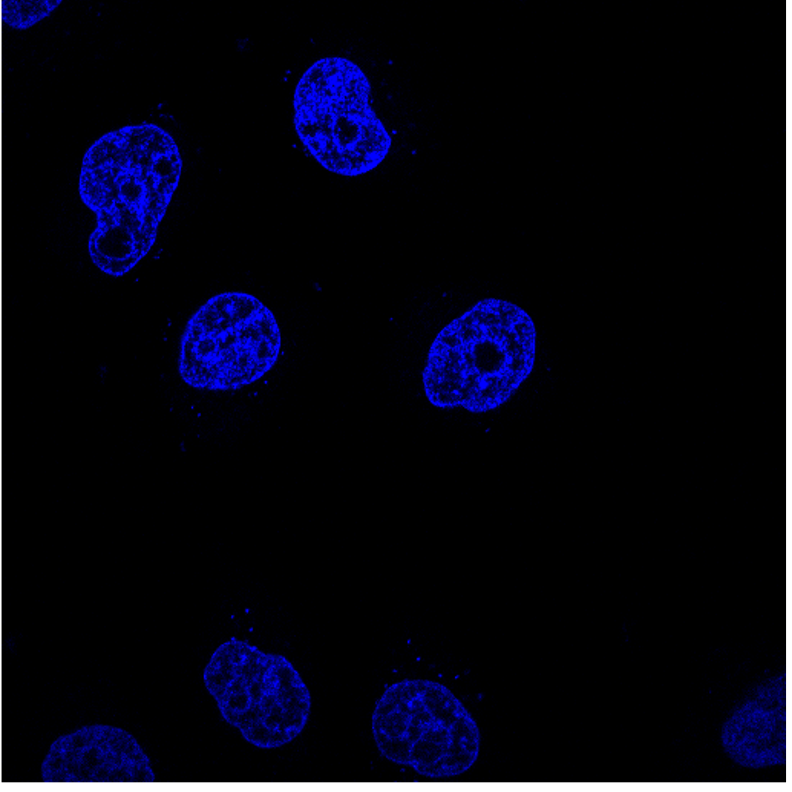

Supplement: Figure 4—source data 1. [file elife-69916-fig4-data1.zip › Figure4_Source data_BG4 localization to mtDNA/Figure 4D_Representative image_rho cells_BG4_mitotracker red/Figure 4D_Representative image_rho cell_sec control_DAPI.tif]

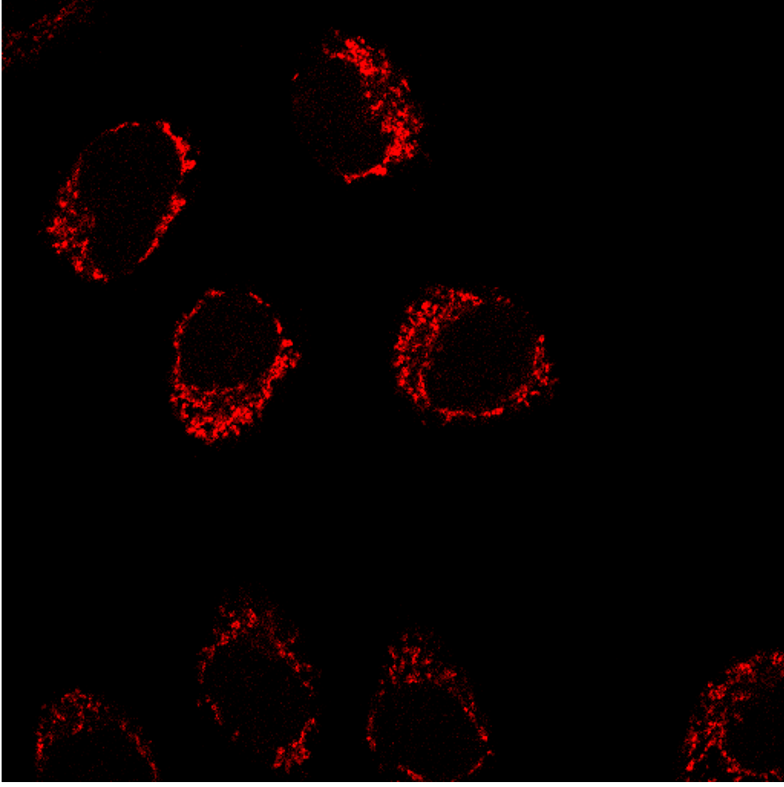

Supplement: Figure 4—source data 1. [file elife-69916-fig4-data1.zip › Figure4_Source data_BG4 localization to mtDNA/Figure 4D_Representative image_rho cells_BG4_mitotracker red/Figure 4D_Representative image_rho cell_sec control_mtTracker red.tif]

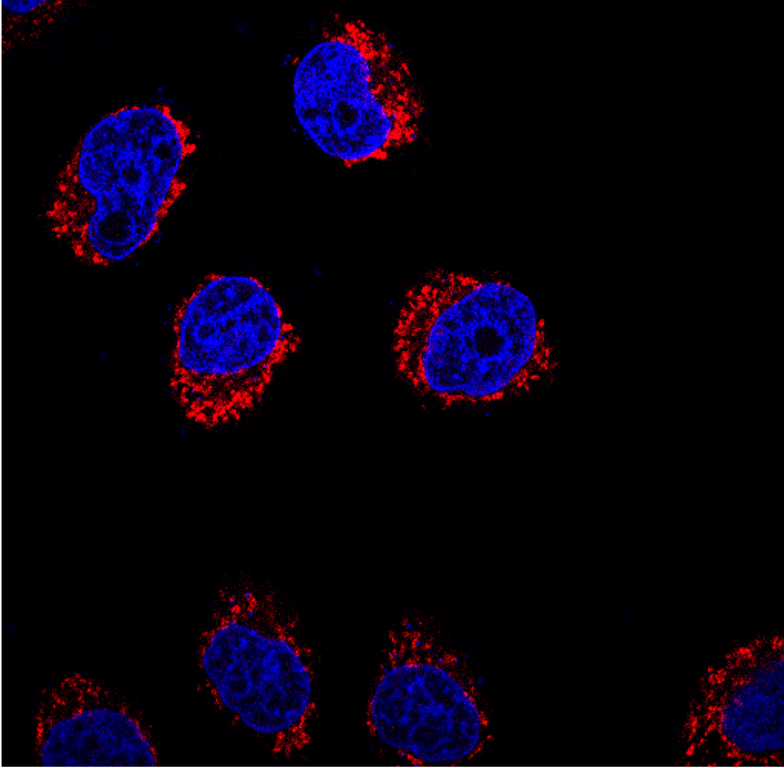

Supplement: Figure 4—source data 1. [file elife-69916-fig4-data1.zip › Figure4_Source data_BG4 localization to mtDNA/Figure 4D_Representative image_rho cells_BG4_mitotracker red/Figure 4D_Representative image_rho cell_sec control_merged.tif]

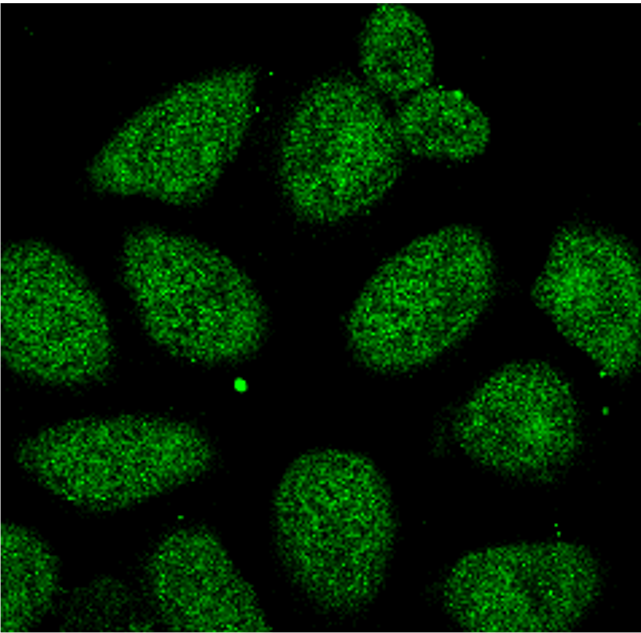

Supplement: Figure 4—source data 1. [file elife-69916-fig4-data1.zip › Figure4_Source data_BG4 localization to mtDNA/Figure 4D_Representative image_rho cells_BG4_mitotracker red/Figure 4D_Representative image_rho cells_BG4_mito tracker red_BG4.tif]

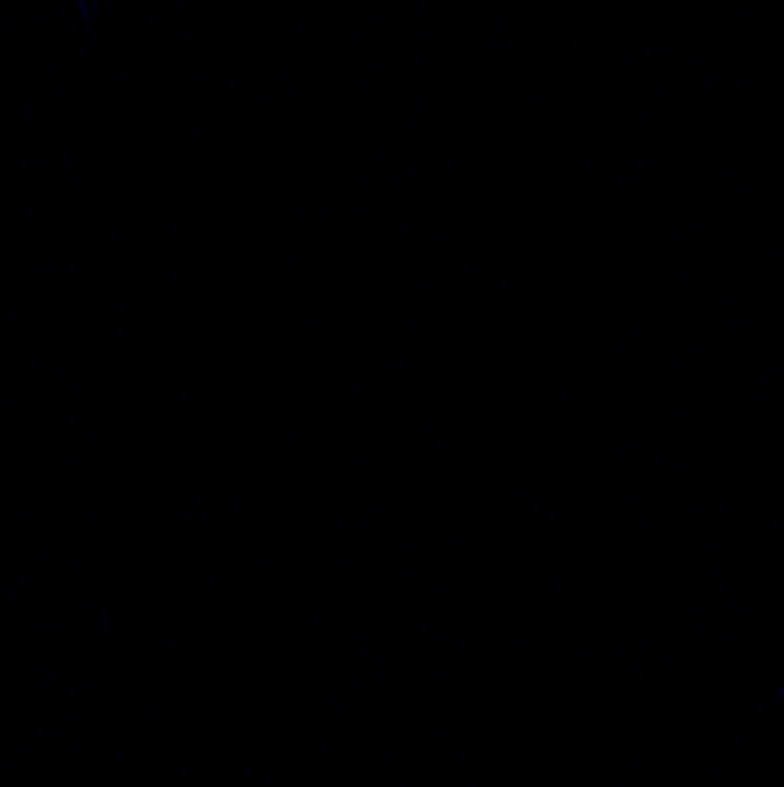

Supplement: Figure 4—source data 1. [file elife-69916-fig4-data1.zip › Figure4_Source data_BG4 localization to mtDNA/Figure 4D_Representative image_rho cells_BG4_mitotracker red/Figure 4D_Representative image_rho cell_sec control_BG4.tif]

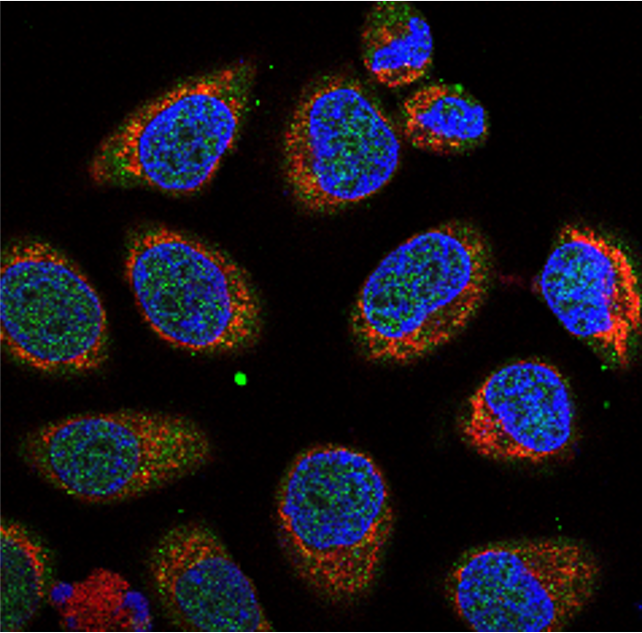

Supplement: Figure 4—source data 1. [file elife-69916-fig4-data1.zip › Figure4_Source data_BG4 localization to mtDNA/Figure 4D_Representative image_rho cells_BG4_mitotracker red/Figure 4D_Representative image_rho cells_BG4_mito tracker red_merged.tif]

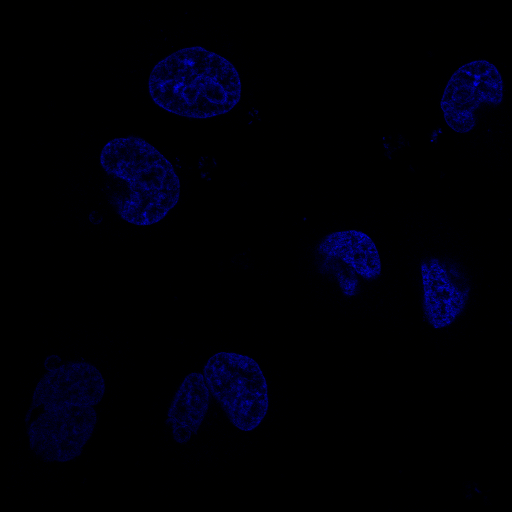

Supplement: Figure 4—source data 1. [file elife-69916-fig4-data1.zip › Figure4_Source data_BG4 localization to mtDNA/Figure 4D-E_IF images_rho cell_BG4/Figure 4D-E_Source data.tif (5).frames/1_0004_C001T001.tif]

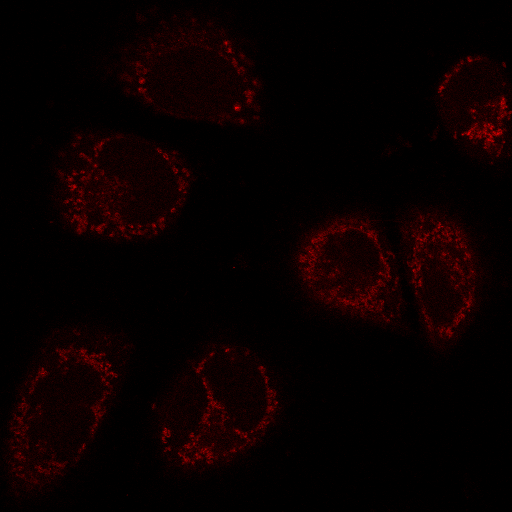

Supplement: Figure 4—source data 1. [file elife-69916-fig4-data1.zip › Figure4_Source data_BG4 localization to mtDNA/Figure 4D-E_IF images_rho cell_BG4/Figure 4D-E_Source data.tif (5).frames/1_0004_C002T001.tif]

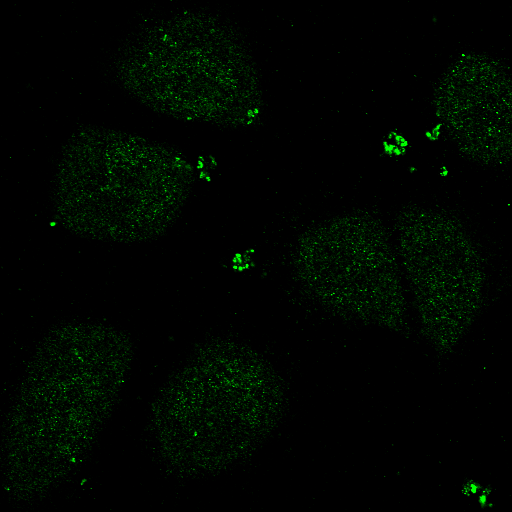

Supplement: Figure 4—source data 1. [file elife-69916-fig4-data1.zip › Figure4_Source data_BG4 localization to mtDNA/Figure 4D-E_IF images_rho cell_BG4/Figure 4D-E_Source data.tif (5).frames/1_0004_C003T001.tif]

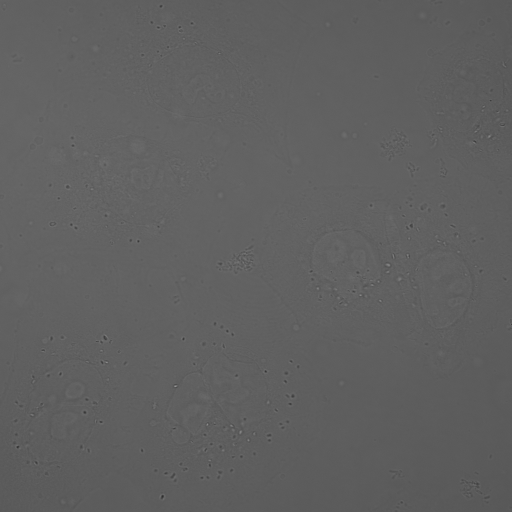

Supplement: Figure 4—source data 1. [file elife-69916-fig4-data1.zip › Figure4_Source data_BG4 localization to mtDNA/Figure 4D-E_IF images_rho cell_BG4/Figure 4D-E_Source data.tif (5).frames/1_0004_C004T001.tif]

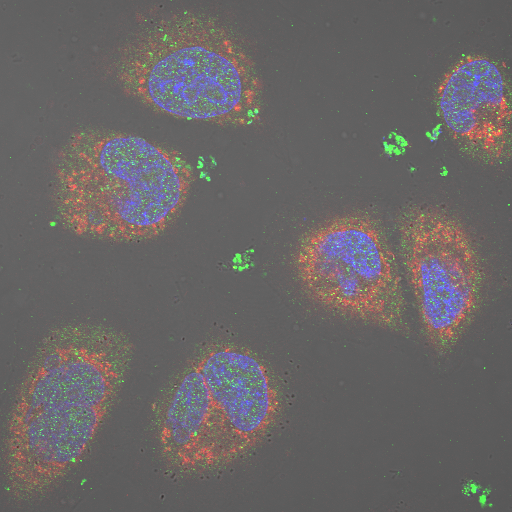

Supplement: Figure 4—source data 1. [file elife-69916-fig4-data1.zip › Figure4_Source data_BG4 localization to mtDNA/Figure 4D-E_IF images_rho cell_BG4/Figure 4D-E_Source data.tif (5).frames/1_0004_T001.tif]

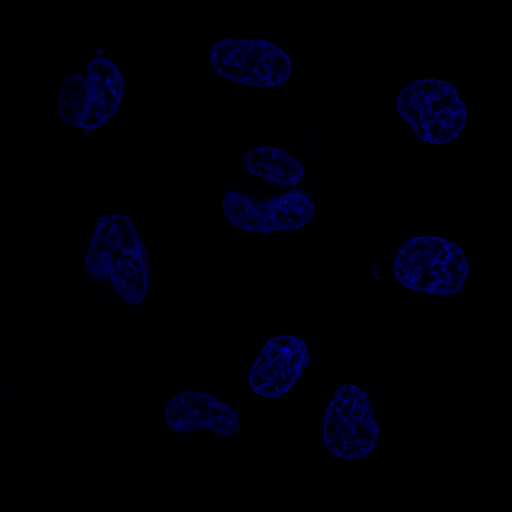

Supplement: Figure 4—source data 1. [file elife-69916-fig4-data1.zip › Figure4_Source data_BG4 localization to mtDNA/Figure 4D-E_IF images_rho cell_BG4/Figure 4D-E_Source data.tif (4).frames/1_0003_C001T001.tif]

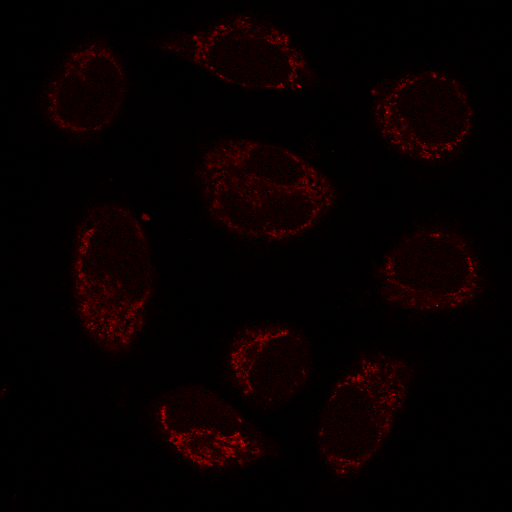

Supplement: Figure 4—source data 1. [file elife-69916-fig4-data1.zip › Figure4_Source data_BG4 localization to mtDNA/Figure 4D-E_IF images_rho cell_BG4/Figure 4D-E_Source data.tif (4).frames/1_0003_C002T001.tif]

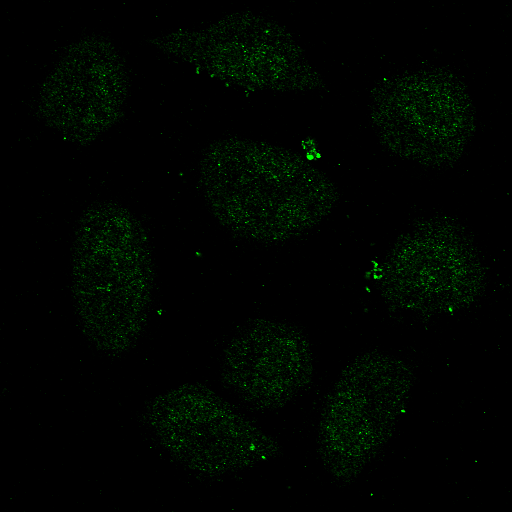

Supplement: Figure 4—source data 1. [file elife-69916-fig4-data1.zip › Figure4_Source data_BG4 localization to mtDNA/Figure 4D-E_IF images_rho cell_BG4/Figure 4D-E_Source data.tif (4).frames/1_0003_C003T001.tif]

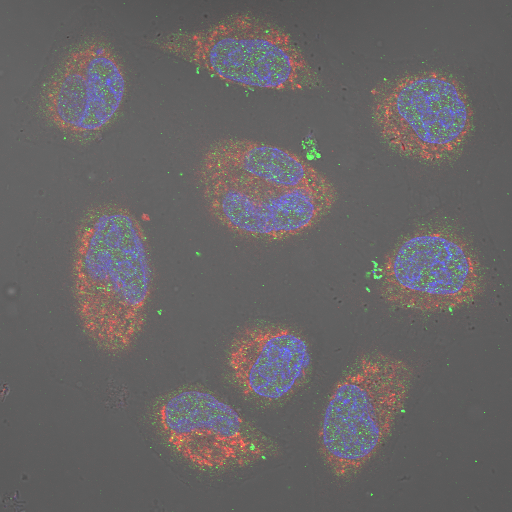

Supplement: Figure 4—source data 1. [file elife-69916-fig4-data1.zip › Figure4_Source data_BG4 localization to mtDNA/Figure 4D-E_IF images_rho cell_BG4/Figure 4D-E_Source data.tif (4).frames/1_0003_T001.tif]

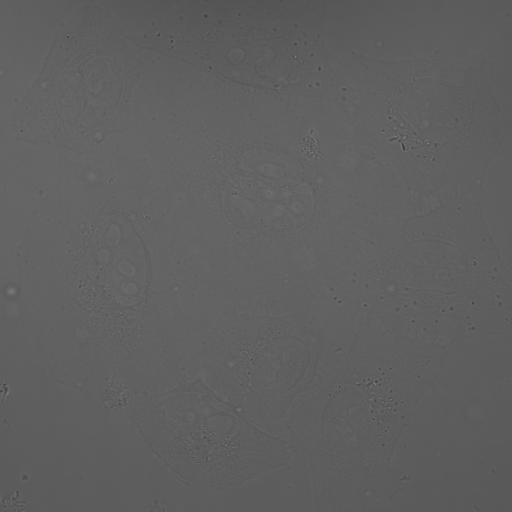

Supplement: Figure 4—source data 1. [file elife-69916-fig4-data1.zip › Figure4_Source data_BG4 localization to mtDNA/Figure 4D-E_IF images_rho cell_BG4/Figure 4D-E_Source data.tif (4).frames/1_0003_C004T001.tif]

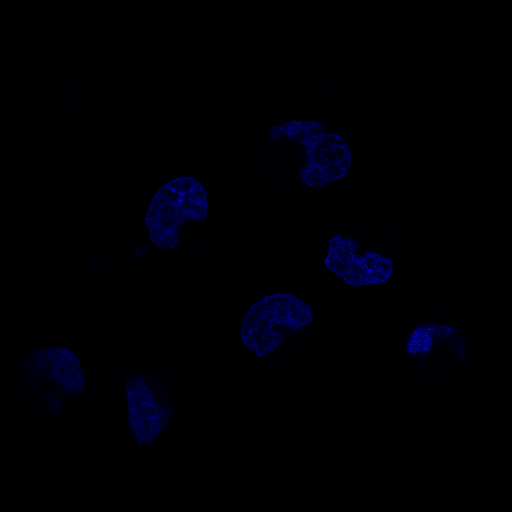

Supplement: Figure 4—source data 1. [file elife-69916-fig4-data1.zip › Figure4_Source data_BG4 localization to mtDNA/Figure 4D-E_IF images_rho cell_BG4/Figure 4D-E_Source data.tif (6).frames/1_0005_C001T001.tif]

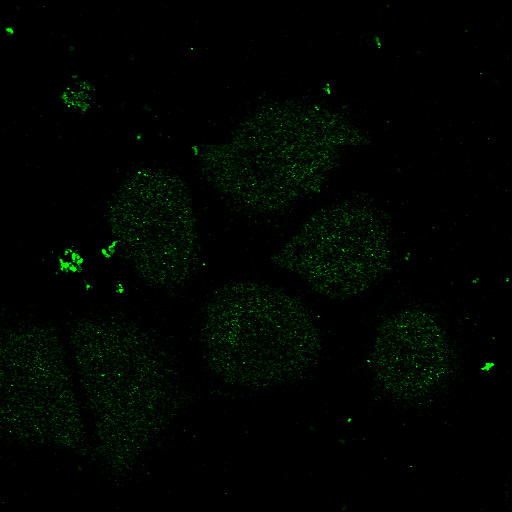

Supplement: Figure 4—source data 1. [file elife-69916-fig4-data1.zip › Figure4_Source data_BG4 localization to mtDNA/Figure 4D-E_IF images_rho cell_BG4/Figure 4D-E_Source data.tif (6).frames/1_0005_C003T001.tif]

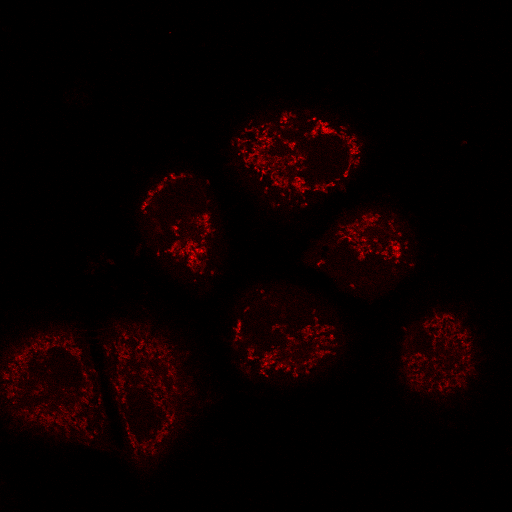

Supplement: Figure 4—source data 1. [file elife-69916-fig4-data1.zip › Figure4_Source data_BG4 localization to mtDNA/Figure 4D-E_IF images_rho cell_BG4/Figure 4D-E_Source data.tif (6).frames/1_0005_C002T001.tif]

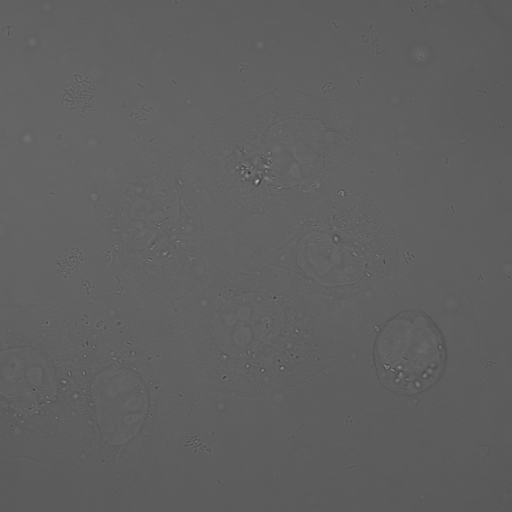

Supplement: Figure 4—source data 1. [file elife-69916-fig4-data1.zip › Figure4_Source data_BG4 localization to mtDNA/Figure 4D-E_IF images_rho cell_BG4/Figure 4D-E_Source data.tif (6).frames/1_0005_C004T001.tif]

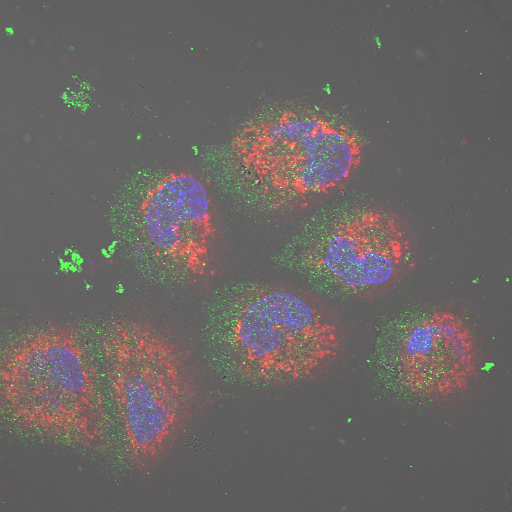

Supplement: Figure 4—source data 1. [file elife-69916-fig4-data1.zip › Figure4_Source data_BG4 localization to mtDNA/Figure 4D-E_IF images_rho cell_BG4/Figure 4D-E_Source data.tif (6).frames/1_0005_T001.tif]

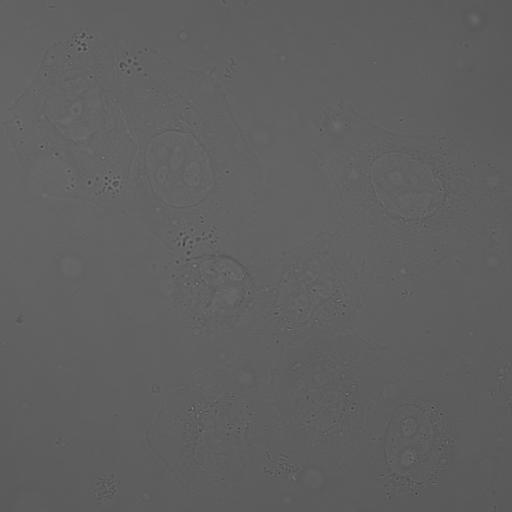

Supplement: Figure 4—source data 1. [file elife-69916-fig4-data1.zip › Figure4_Source data_BG4 localization to mtDNA/Figure 4D-E_IF images_rho cell_BG4/Figure 4D-E_Source data.tif (7).frames/1_0006_C004T001.tif]

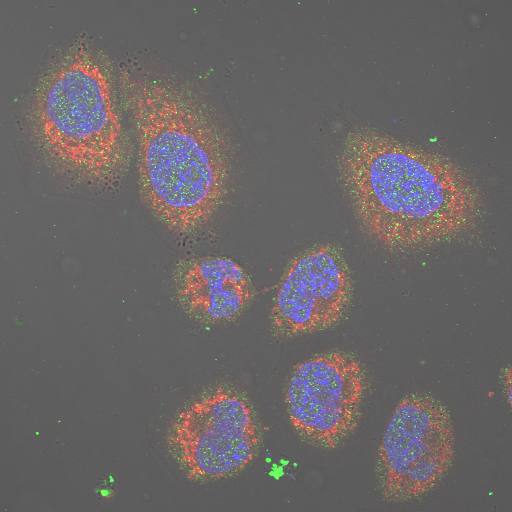

Supplement: Figure 4—source data 1. [file elife-69916-fig4-data1.zip › Figure4_Source data_BG4 localization to mtDNA/Figure 4D-E_IF images_rho cell_BG4/Figure 4D-E_Source data.tif (7).frames/1_0006_T001.tif]

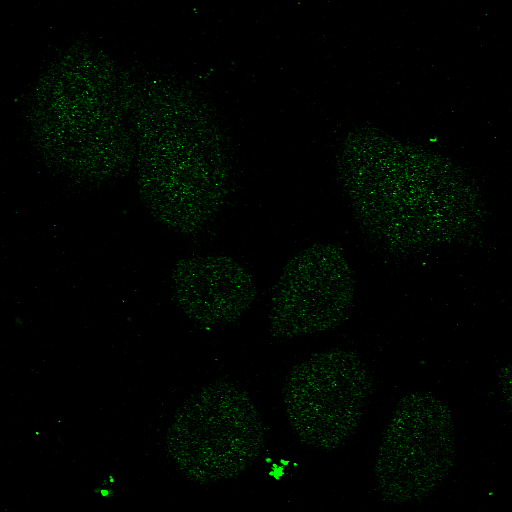

Supplement: Figure 4—source data 1. [file elife-69916-fig4-data1.zip › Figure4_Source data_BG4 localization to mtDNA/Figure 4D-E_IF images_rho cell_BG4/Figure 4D-E_Source data.tif (7).frames/1_0006_C003T001.tif]

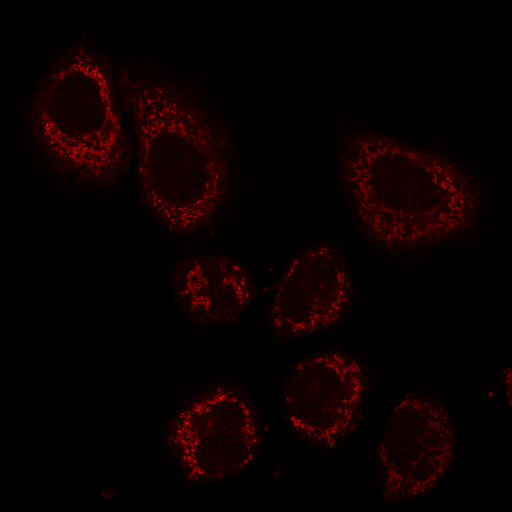

Supplement: Figure 4—source data 1. [file elife-69916-fig4-data1.zip › Figure4_Source data_BG4 localization to mtDNA/Figure 4D-E_IF images_rho cell_BG4/Figure 4D-E_Source data.tif (7).frames/1_0006_C002T001.tif]

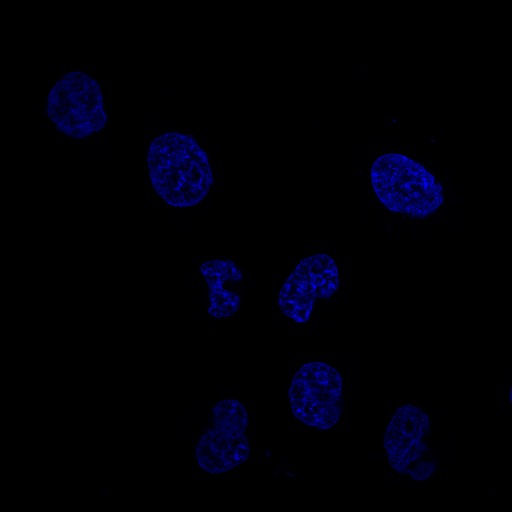

Supplement: Figure 4—source data 1. [file elife-69916-fig4-data1.zip › Figure4_Source data_BG4 localization to mtDNA/Figure 4D-E_IF images_rho cell_BG4/Figure 4D-E_Source data.tif (7).frames/1_0006_C001T001.tif]

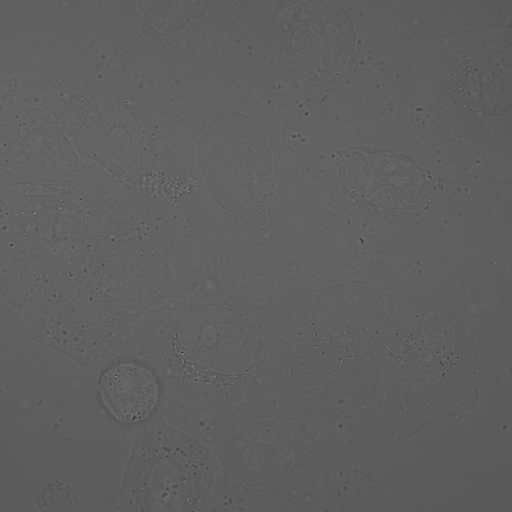

Supplement: Figure 4—source data 1. [file elife-69916-fig4-data1.zip › Figure4_Source data_BG4 localization to mtDNA/Figure 4D-E_IF images_rho cell_BG4/Figure 4D-E_Source data.tif (2).frames/1_0001_C004T001.tif]

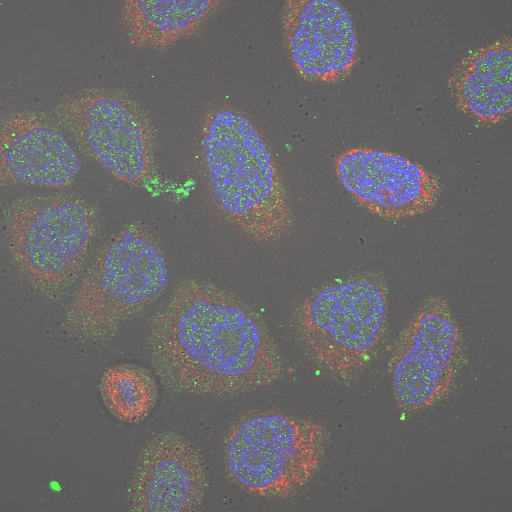

Supplement: Figure 4—source data 1. [file elife-69916-fig4-data1.zip › Figure4_Source data_BG4 localization to mtDNA/Figure 4D-E_IF images_rho cell_BG4/Figure 4D-E_Source data.tif (2).frames/1_0001_T001.tif]
