# Supplementary material for: Unleashing a novel function of Endonuclease G in mitochondrial genome instability
Source: eLife. 2022 Nov 17;11:e69916. doi: 10.7554/eLife.69916 (PMC9711528; doi:10.7554/eLife.69916)
Supplement: Figure 1—source data 2. [file elife-69916-fig1-data2.zip › Figure 1_Source data2_Supplementary/Figure S1C_EMSA gel profile_Mitochondrial region_presence_absence_KCl.pptx]

## Slide 1
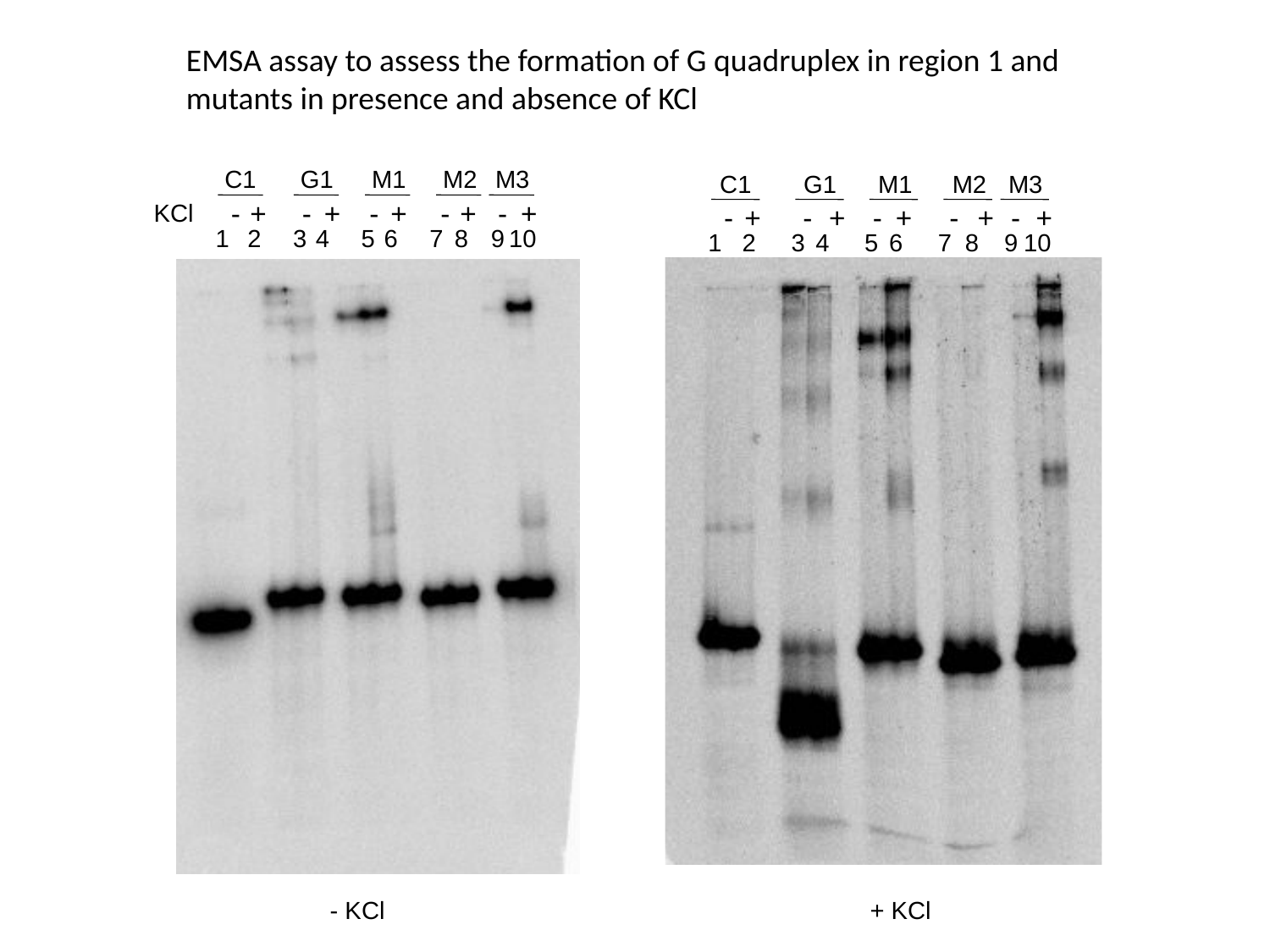

EMSA assay to assess the formation of G quadruplex in region 1 and mutants in presence and absence of KCl
C1
G1
M1
M2
M3
C1
G1
M1
M2
M3
-
+
-
 +
-
+
-
 +
-
+
1
2
3
4
5
6
7
8
9
10
-
+
-
 +
-
+
-
 +
-
+
KCl
1
2
3
4
5
6
7
8
9
10
- KCl
+ KCl
