# Supplementary material for: Unleashing a novel function of Endonuclease G in mitochondrial genome instability
Source: eLife. 2022 Nov 17;11:e69916. doi: 10.7554/eLife.69916 (PMC9711528; doi:10.7554/eLife.69916)
Supplement: Figure 1—source data 2. [file elife-69916-fig1-data2.zip › Figure 1_Source data2_Supplementary/SD_170421_G1 and mutatnts EMSA (1).pptx]

## Slide 1
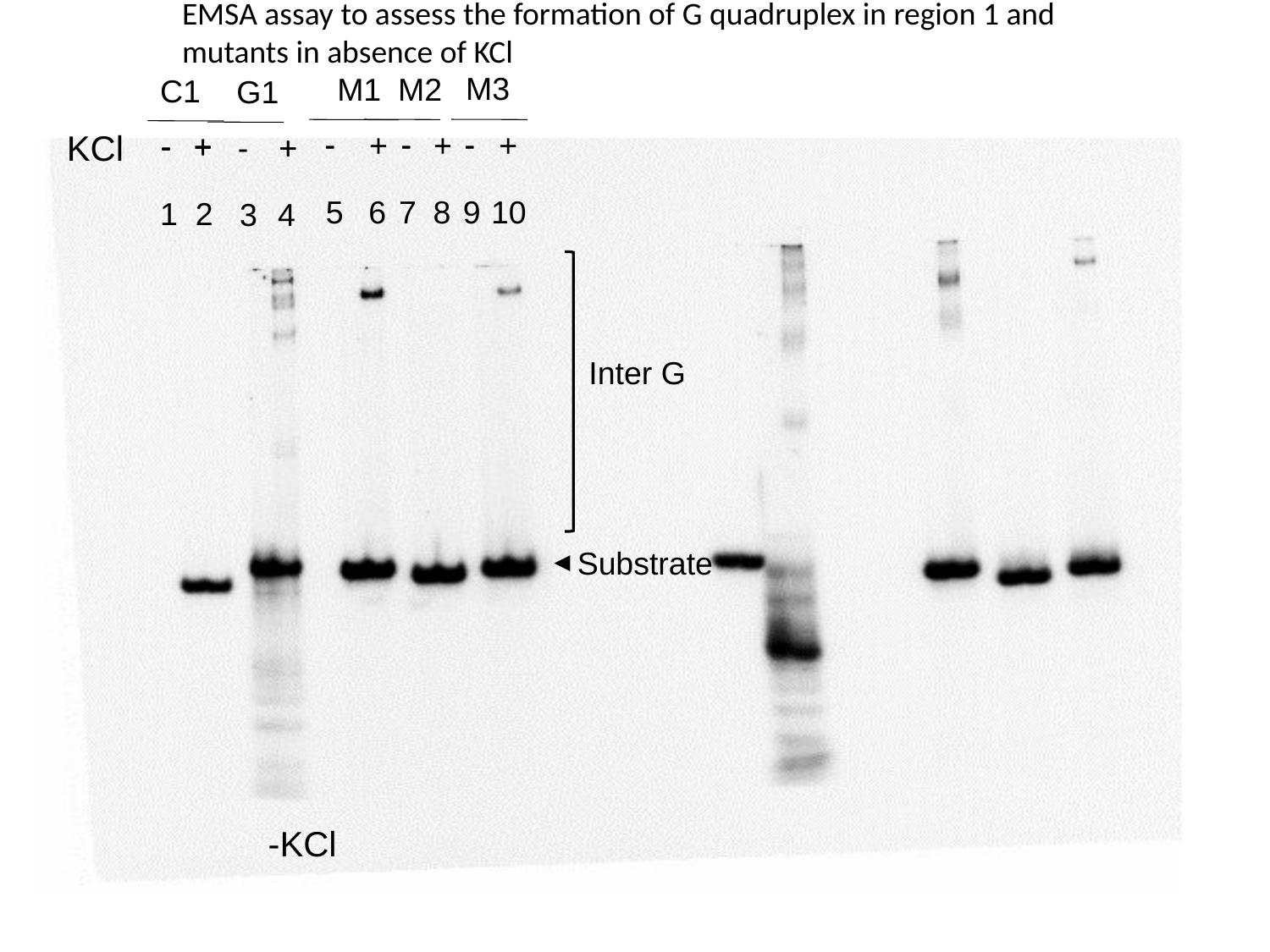

EMSA assay to assess the formation of G quadruplex in region 1 and mutants in absence of KCl
M3
M2
M1
C1
G1
-
-
-
-
-
-
+
+
+
-
-
KCl
-
+
+
-
+
+
7
8
9
10
5
6
1
2
3
4
Inter G
Substrate
-KCl

## Slide 2
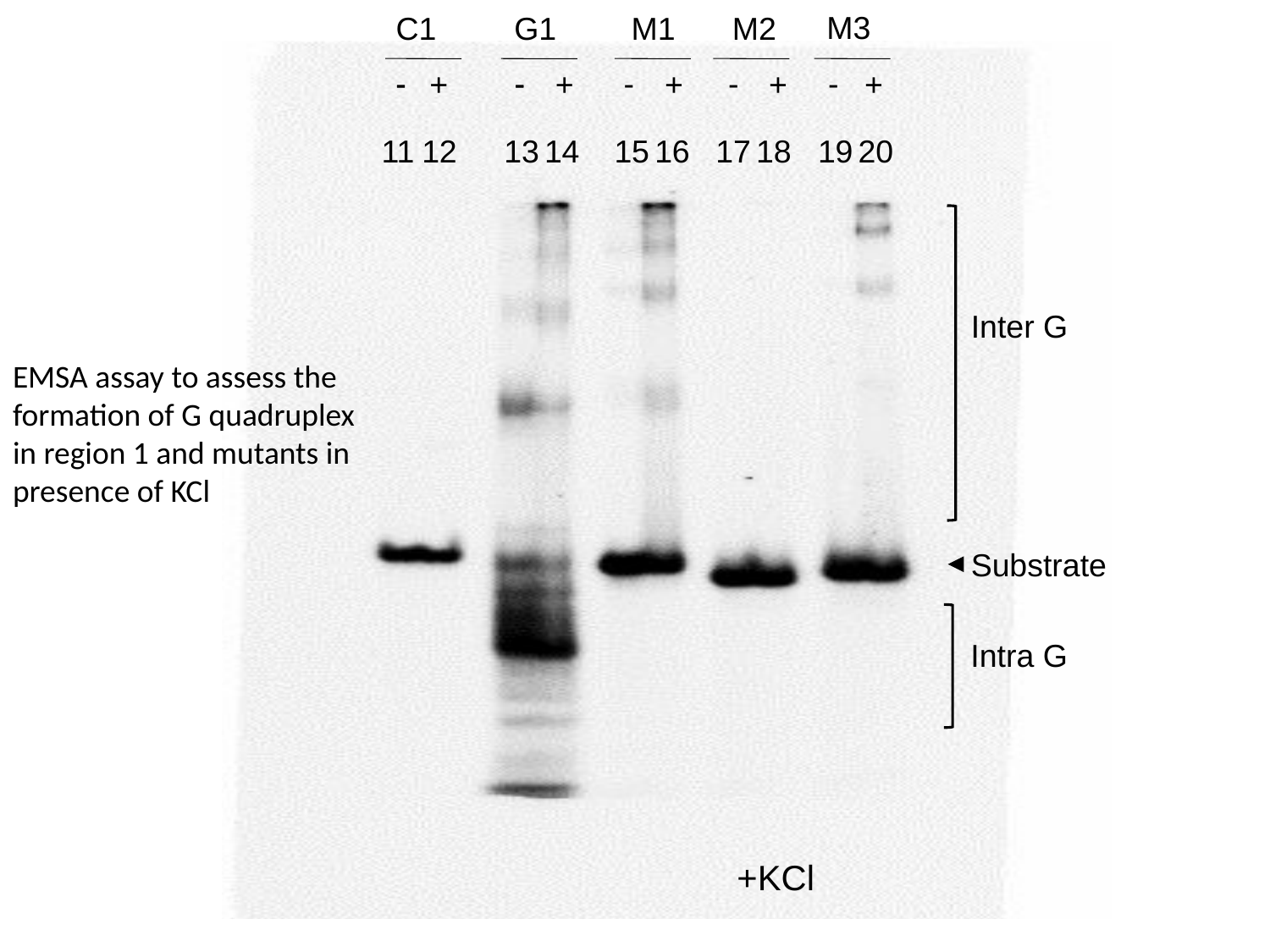

M3
C1
M2
G1
M1
-
-
-
-
-
-
-
+
+
+
+
+
11
12
13
14
15
16
17
18
19
20
Inter G
EMSA assay to assess the formation of G quadruplex in region 1 and mutants in presence of KCl
Substrate
Intra G
+KCl
