# Supplementary material for: Unleashing a novel function of Endonuclease G in mitochondrial genome instability
Source: eLife. 2022 Nov 17;11:e69916. doi: 10.7554/eLife.69916 (PMC9711528; doi:10.7554/eLife.69916)
Supplement: Figure 2—source data 1. [file elife-69916-fig2-data1.zip › Figure2_Source data1_main/Figure 2E_Gel profile_Primer extension_Different ions/Figure 2E_Gel profile_Primer extension_Different ion.pptx]

## Slide 1
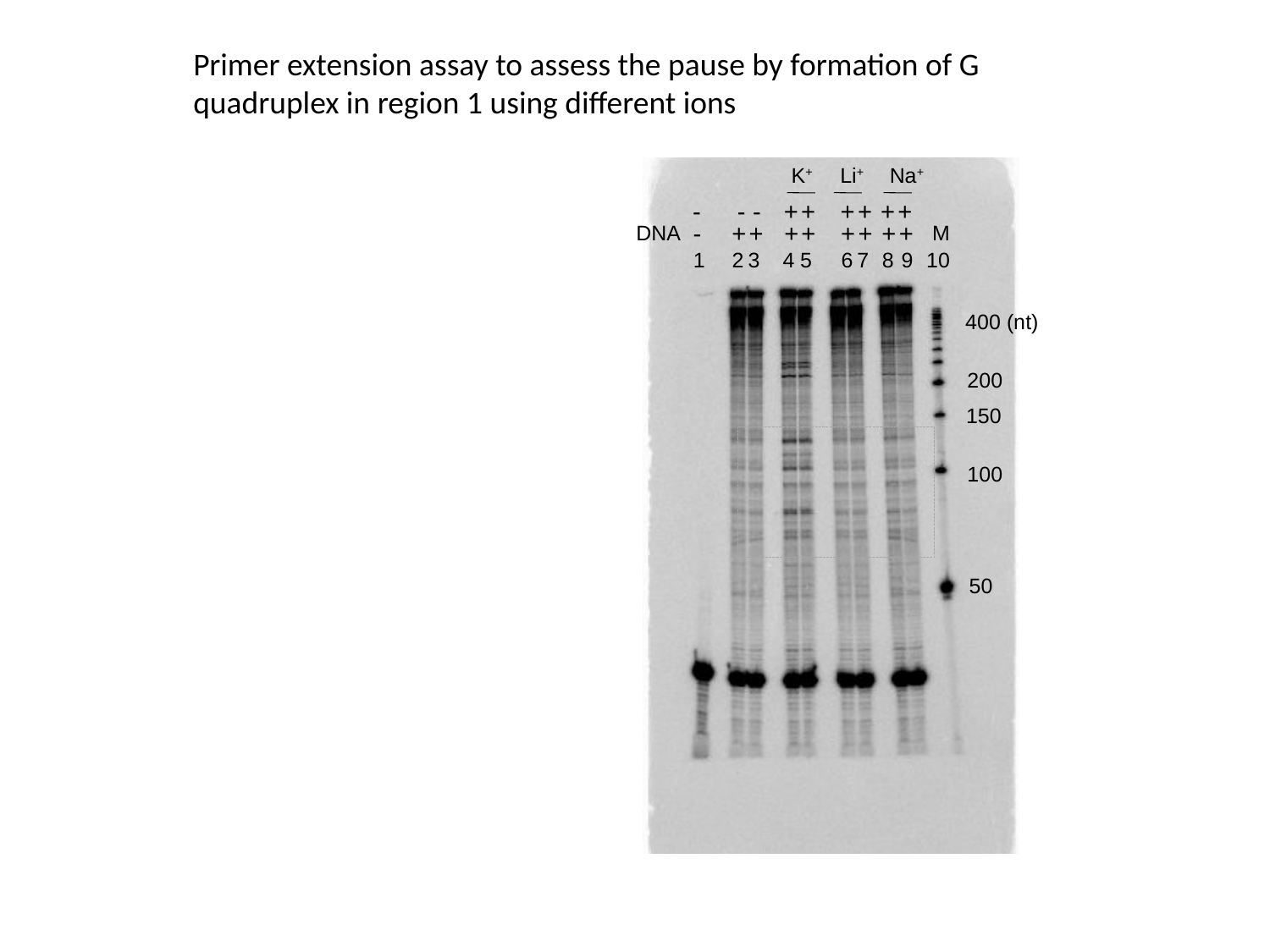

Primer extension assay to assess the pause by formation of G quadruplex in region 1 using different ions
K+
Li+
Na+
-
-
-
+
+
+
+
+
+
-
+
+
+
+
+
+
+
+
DNA
M
10
1
2
3
4
5
6
7
8
9
400 (nt)
200
150
100
50
