# Supplementary material for: Unleashing a novel function of Endonuclease G in mitochondrial genome instability
Source: eLife. 2022 Nov 17;11:e69916. doi: 10.7554/eLife.69916 (PMC9711528; doi:10.7554/eLife.69916)
Supplement: Figure 2—source data 1. [file elife-69916-fig2-data1.zip › Figure2_Source data1_main/Figure 2C_Gel profile_Primer extension_potassium increasing concentration/Figure 1C_Gel profile_Primer extension_potassium increasing concentration.pptx]

## Slide 1
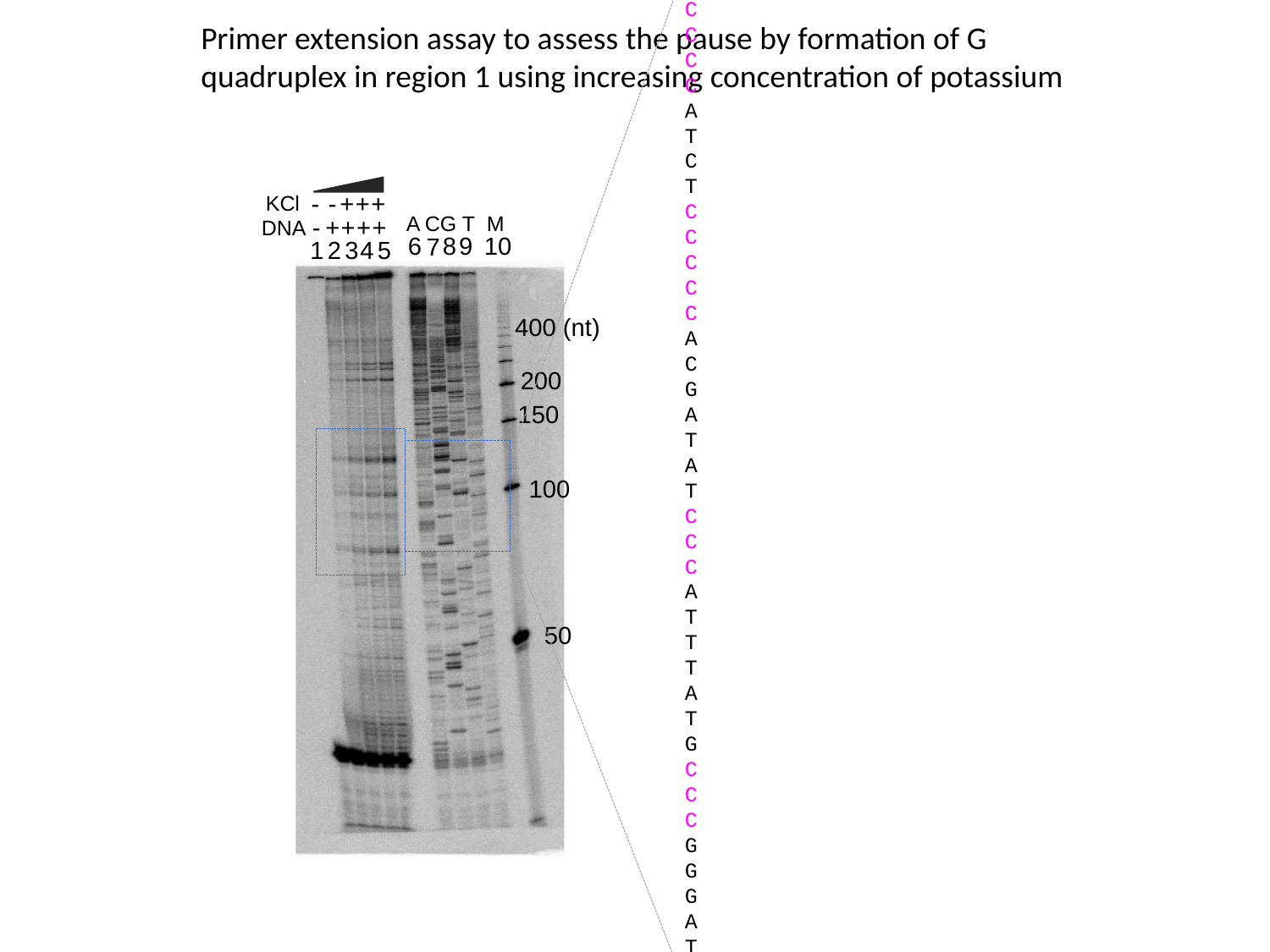

3’
T
C
C
C
C
C
A
T
C
T
C
C
C
C
C
A
C
G
A
T
A
T
C
C
C
A
T
T
T
A
T
G
C
C
C
G
G
G
A
T
5’
Primer extension assay to assess the pause by formation of G quadruplex in region 1 using increasing concentration of potassium
-
-
+
+
+
KCl
A CG T
M
-
+
+
+
+
DNA
6
8
9
10
7
1
2
3
4
5
400 (nt)
200
150
100
50
