# Supplementary material for: Unleashing a novel function of Endonuclease G in mitochondrial genome instability
Source: eLife. 2022 Nov 17;11:e69916. doi: 10.7554/eLife.69916 (PMC9711528; doi:10.7554/eLife.69916)
Supplement: Figure 2—source data 1. [file elife-69916-fig2-data1.zip › Figure2_Source data1_main/Figure 2D_Source data_Gel profile/Figure 2D_Gel profile_Primer extension_wild type vs mutant plasmid.pptx]

## Slide 1
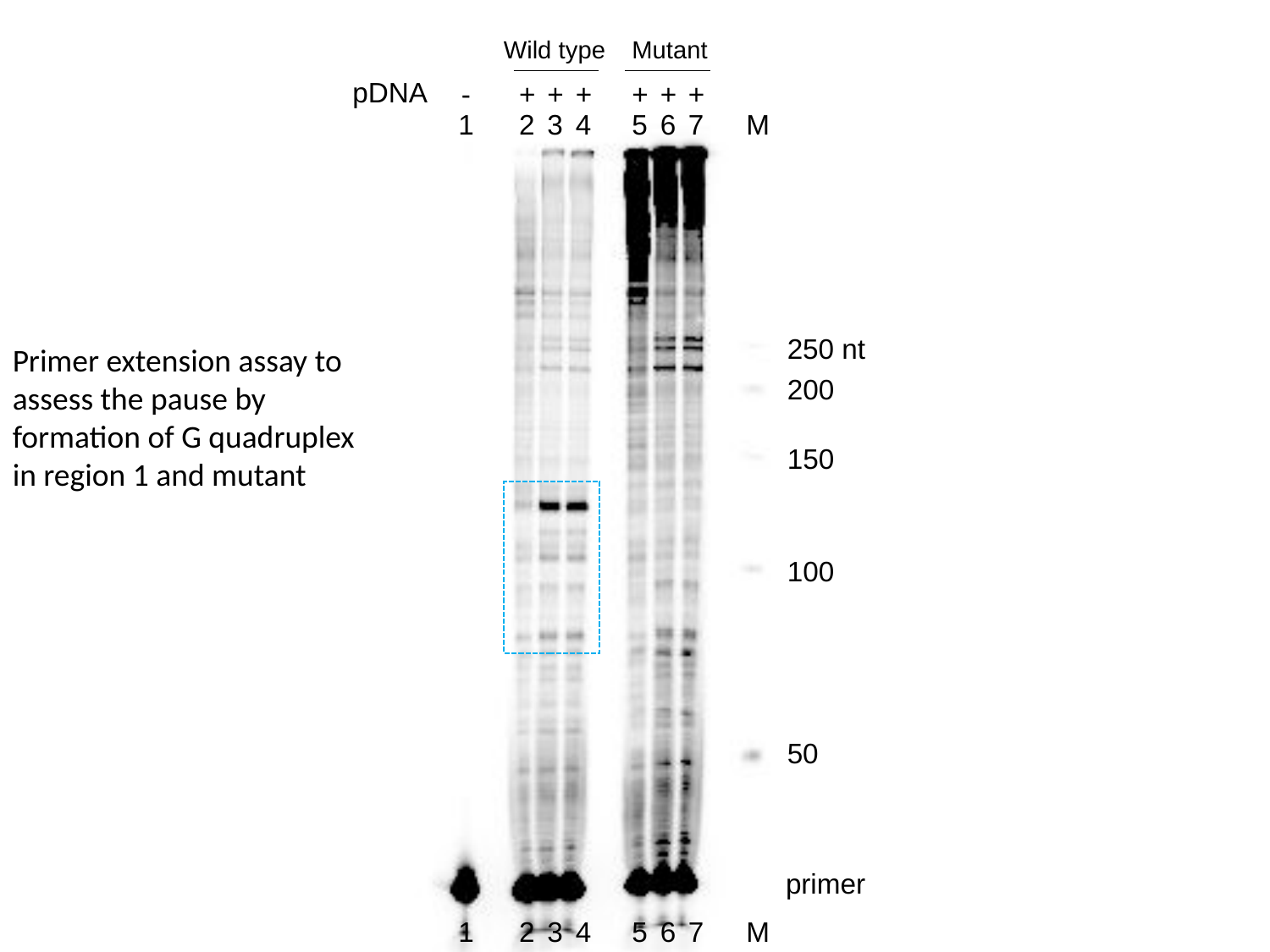

Wild type
Mutant
pDNA
-
+
+
+
+
+
+
1
2
3
4
5
6
7
M
250 nt
Primer extension assay to assess the pause by formation of G quadruplex in region 1 and mutant
200
150
100
50
primer
1
2
3
4
5
6
7
M
