# Supplementary figures and images for: Unleashing a novel function of Endonuclease G in mitochondrial genome instability (part 2 of 4)
Source: eLife. 2022 Nov 17;11:e69916. doi: 10.7554/eLife.69916 (PMC9711528; doi:10.7554/eLife.69916)

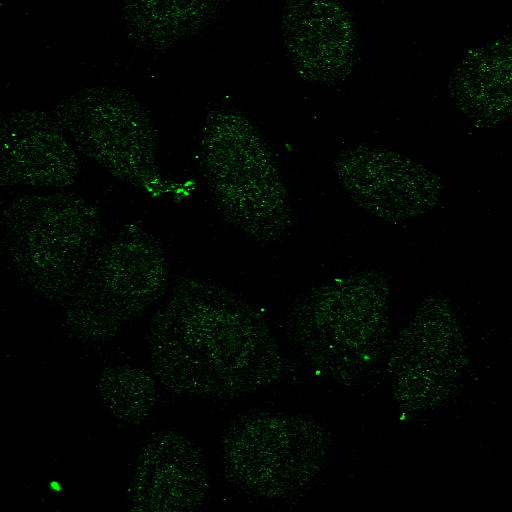

Supplement: Figure 4—source data 1. [file elife-69916-fig4-data1.zip › Figure4_Source data_BG4 localization to mtDNA/Figure 4D-E_IF images_rho cell_BG4/Figure 4D-E_Source data.tif (2).frames/1_0001_C003T001.tif]

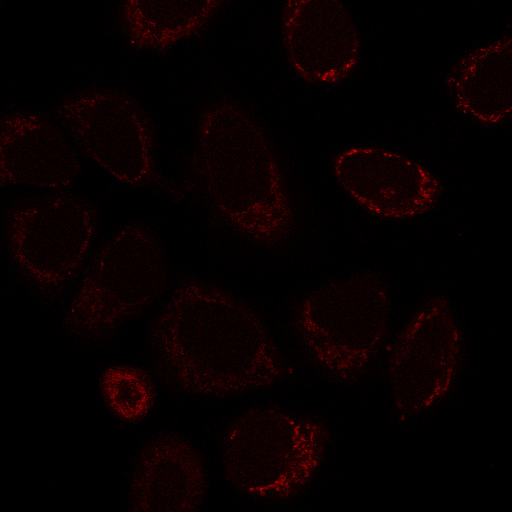

Supplement: Figure 4—source data 1. [file elife-69916-fig4-data1.zip › Figure4_Source data_BG4 localization to mtDNA/Figure 4D-E_IF images_rho cell_BG4/Figure 4D-E_Source data.tif (2).frames/1_0001_C002T001.tif]

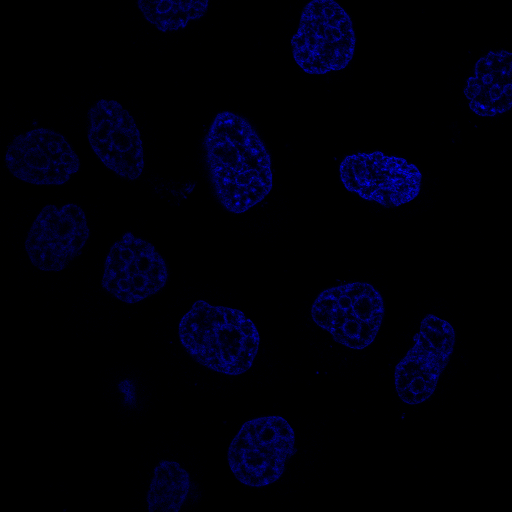

Supplement: Figure 4—source data 1. [file elife-69916-fig4-data1.zip › Figure4_Source data_BG4 localization to mtDNA/Figure 4D-E_IF images_rho cell_BG4/Figure 4D-E_Source data.tif (2).frames/1_0001_C001T001.tif]

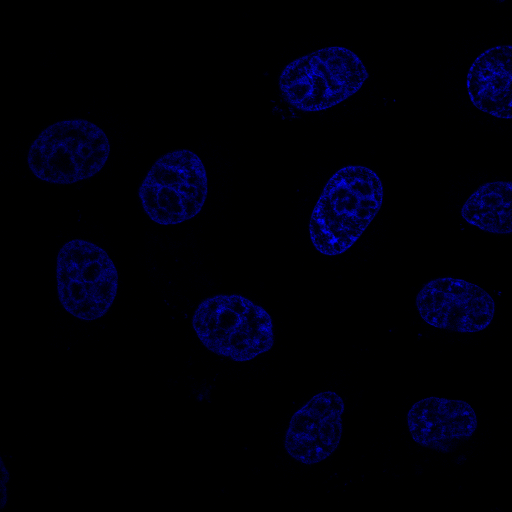

Supplement: Figure 4—source data 1. [file elife-69916-fig4-data1.zip › Figure4_Source data_BG4 localization to mtDNA/Figure 4D-E_IF images_rho cell_BG4/Figure 4D-E_Source data.tif (3).frames/1_0002_C001T001.tif]

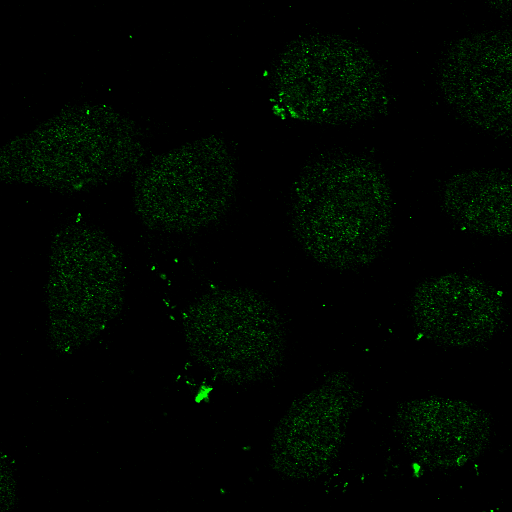

Supplement: Figure 4—source data 1. [file elife-69916-fig4-data1.zip › Figure4_Source data_BG4 localization to mtDNA/Figure 4D-E_IF images_rho cell_BG4/Figure 4D-E_Source data.tif (3).frames/1_0002_C003T001.tif]

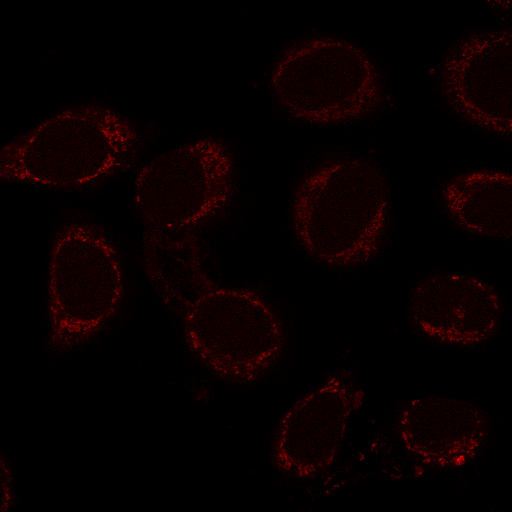

Supplement: Figure 4—source data 1. [file elife-69916-fig4-data1.zip › Figure4_Source data_BG4 localization to mtDNA/Figure 4D-E_IF images_rho cell_BG4/Figure 4D-E_Source data.tif (3).frames/1_0002_C002T001.tif]

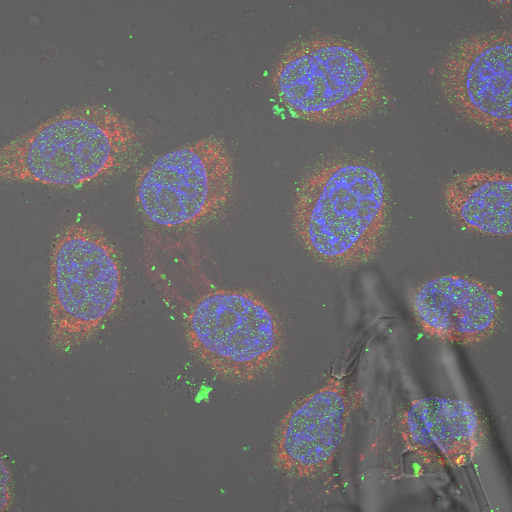

Supplement: Figure 4—source data 1. [file elife-69916-fig4-data1.zip › Figure4_Source data_BG4 localization to mtDNA/Figure 4D-E_IF images_rho cell_BG4/Figure 4D-E_Source data.tif (3).frames/1_0002_T001.tif]

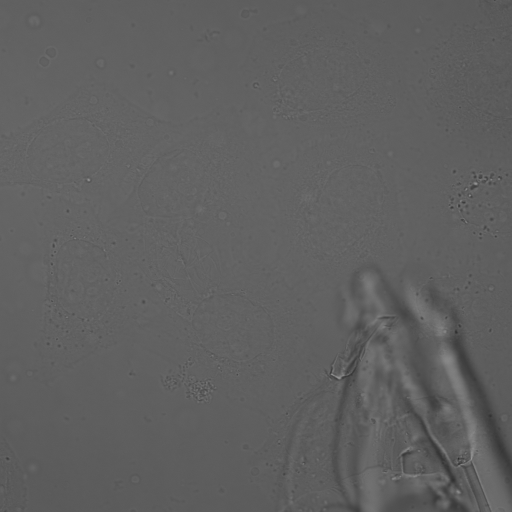

Supplement: Figure 4—source data 1. [file elife-69916-fig4-data1.zip › Figure4_Source data_BG4 localization to mtDNA/Figure 4D-E_IF images_rho cell_BG4/Figure 4D-E_Source data.tif (3).frames/1_0002_C004T001.tif]

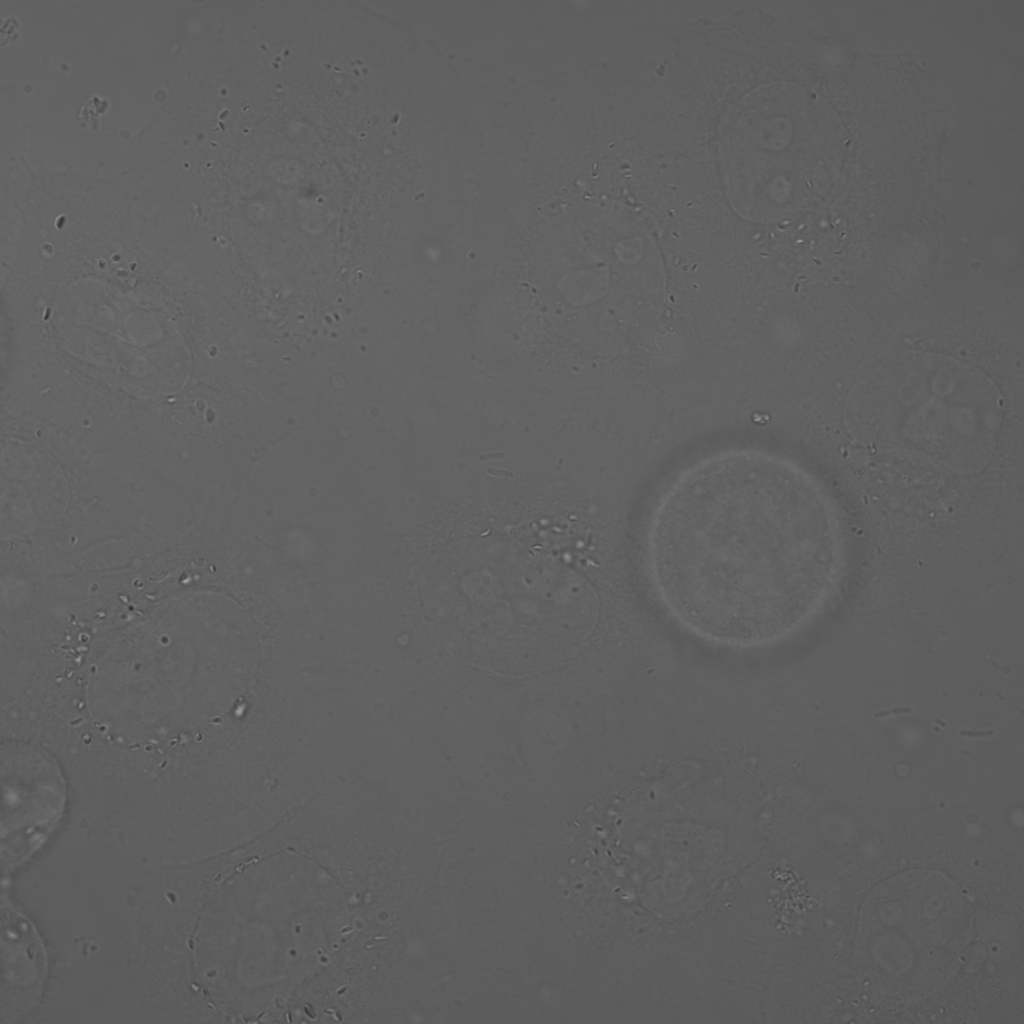

Supplement: Figure 4—source data 1. [file elife-69916-fig4-data1.zip › Figure4_Source data_BG4 localization to mtDNA/Figure 4D-E_IF images_rho cell_BG4/Figure 4D-E_Source data.tif (8).frames/1_0007_C004T001.tif]

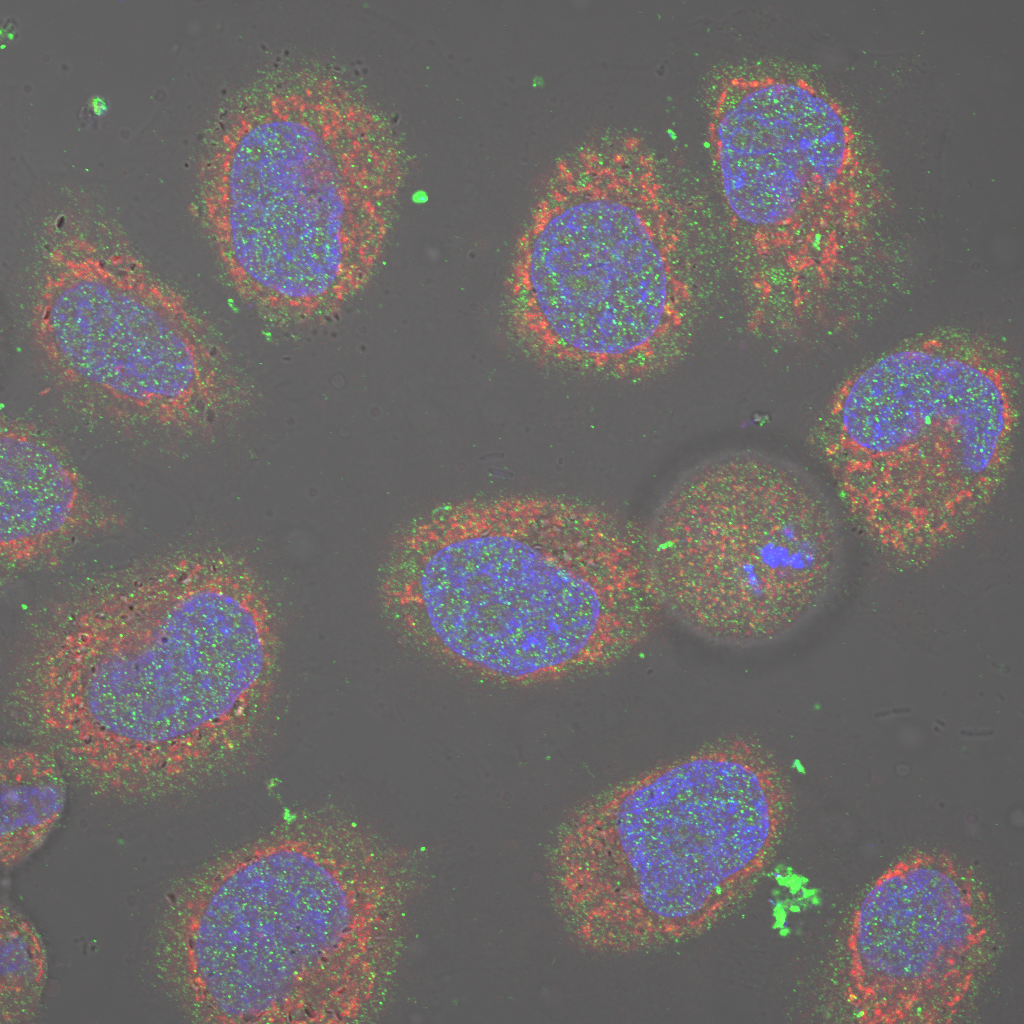

Supplement: Figure 4—source data 1. [file elife-69916-fig4-data1.zip › Figure4_Source data_BG4 localization to mtDNA/Figure 4D-E_IF images_rho cell_BG4/Figure 4D-E_Source data.tif (8).frames/1_0007_T001.tif]

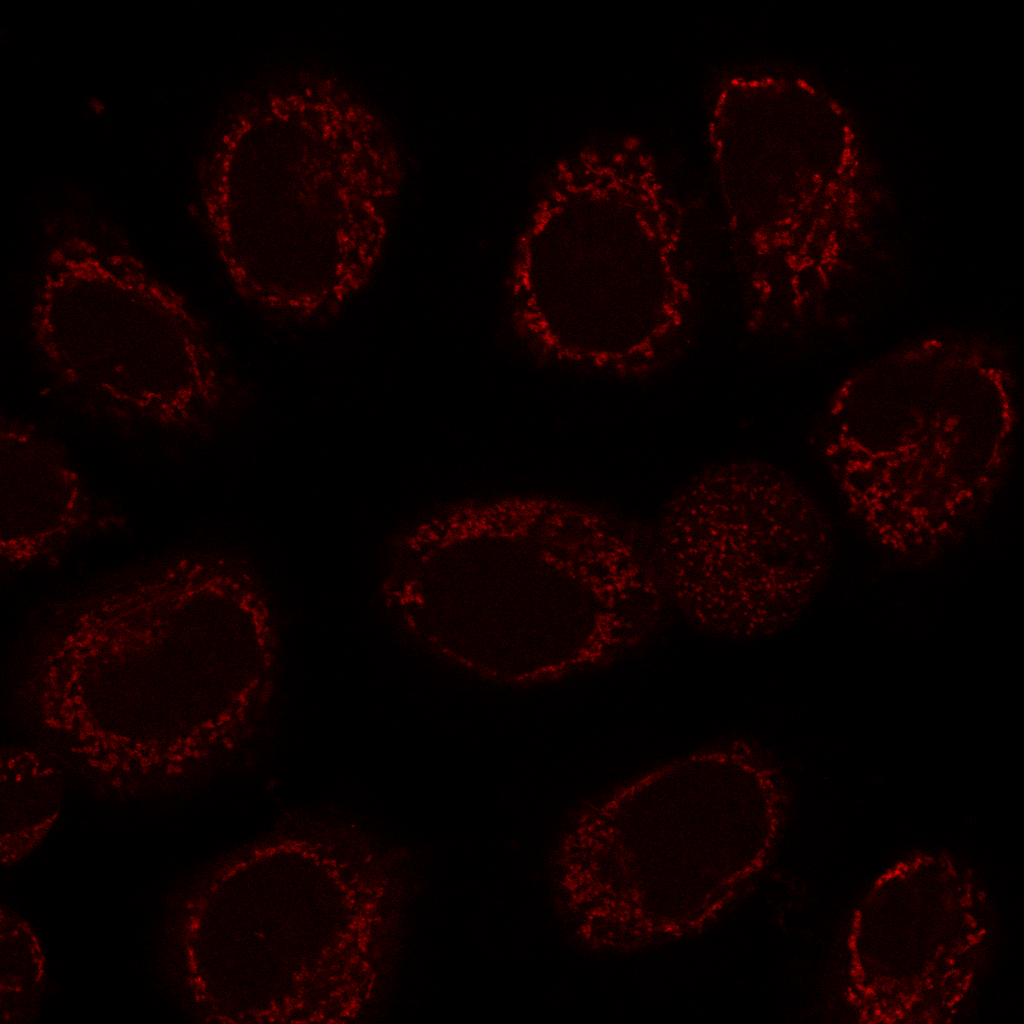

Supplement: Figure 4—source data 1. [file elife-69916-fig4-data1.zip › Figure4_Source data_BG4 localization to mtDNA/Figure 4D-E_IF images_rho cell_BG4/Figure 4D-E_Source data.tif (8).frames/1_0007_C002T001.tif]

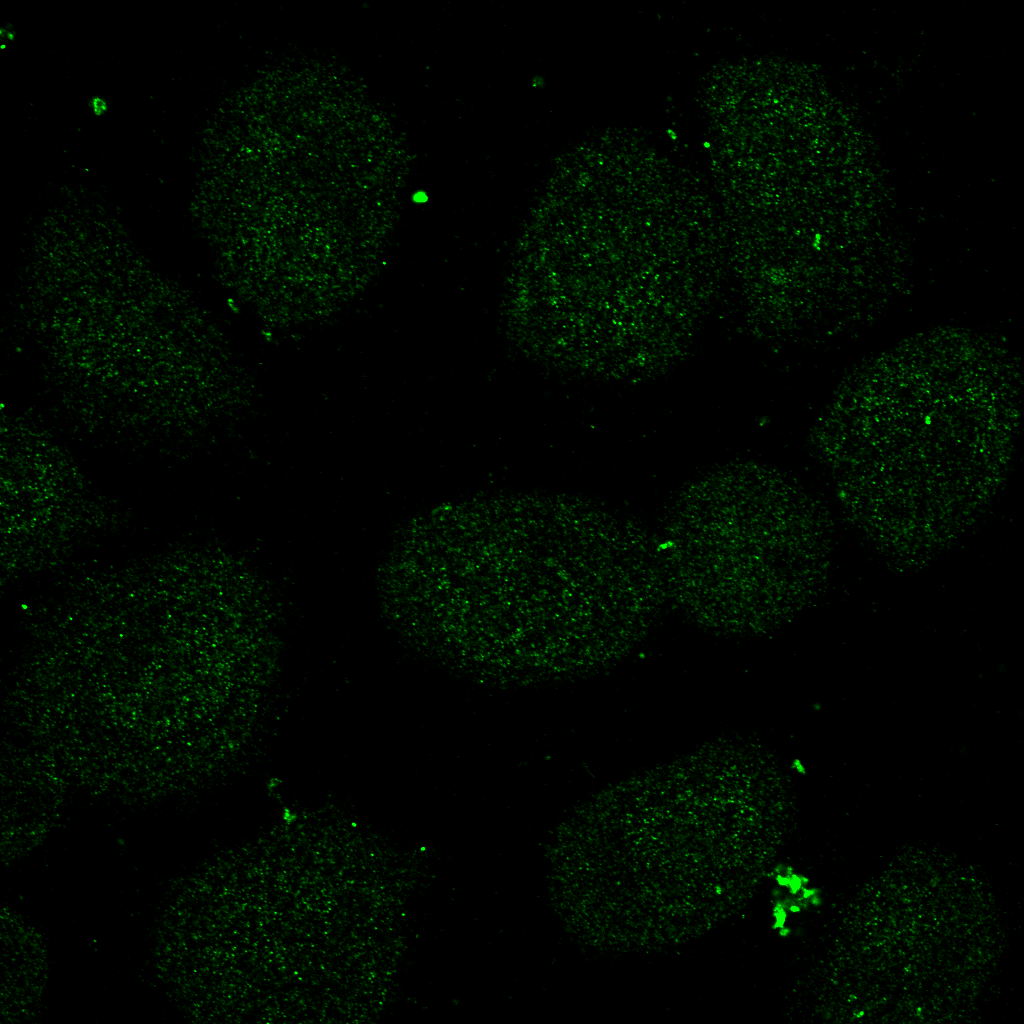

Supplement: Figure 4—source data 1. [file elife-69916-fig4-data1.zip › Figure4_Source data_BG4 localization to mtDNA/Figure 4D-E_IF images_rho cell_BG4/Figure 4D-E_Source data.tif (8).frames/1_0007_C003T001.tif]

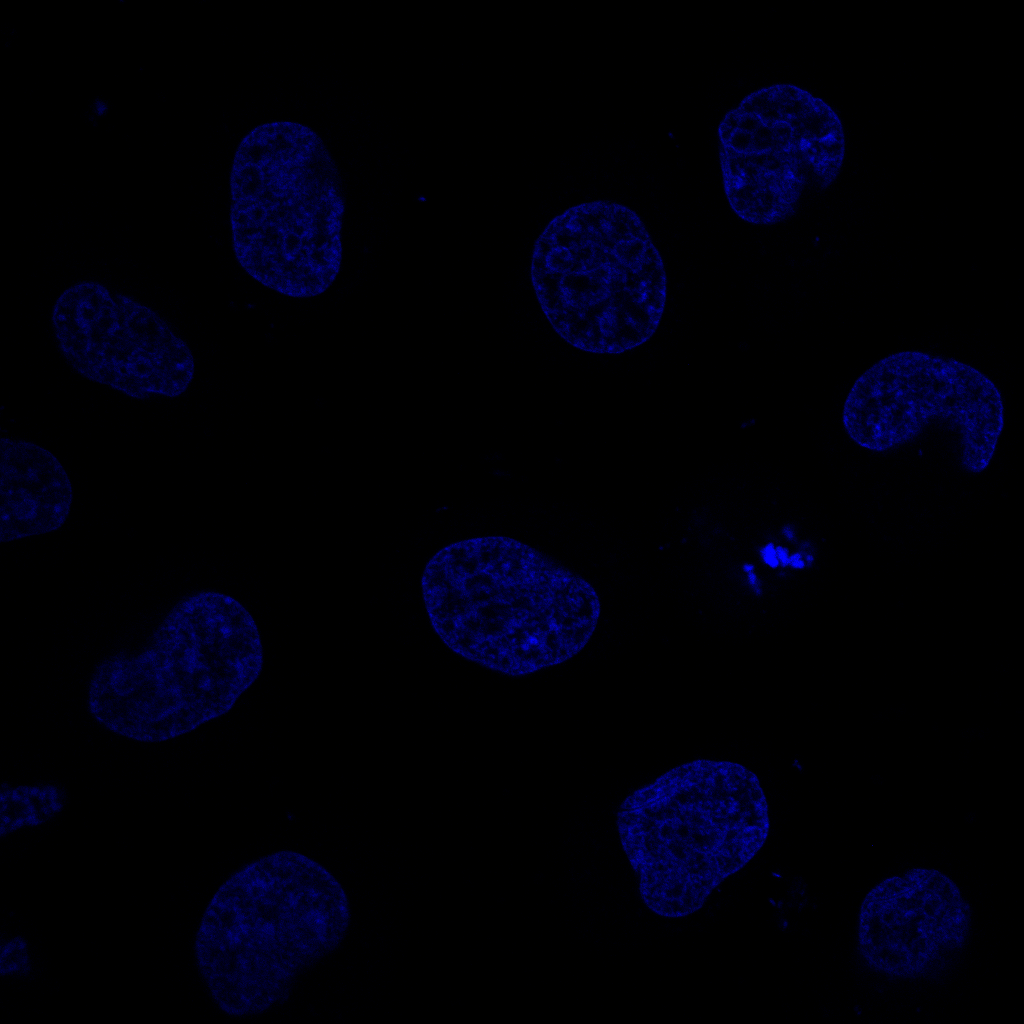

Supplement: Figure 4—source data 1. [file elife-69916-fig4-data1.zip › Figure4_Source data_BG4 localization to mtDNA/Figure 4D-E_IF images_rho cell_BG4/Figure 4D-E_Source data.tif (8).frames/1_0007_C001T001.tif]

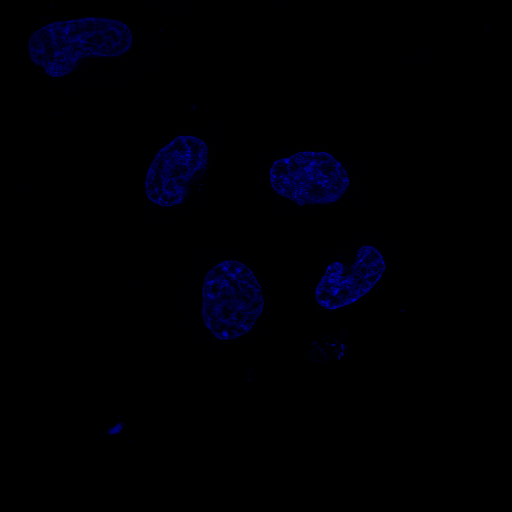

Supplement: Figure 4—source data 1. [file elife-69916-fig4-data1.zip › Figure4_Source data_BG4 localization to mtDNA/Figure 4D-E_IF images_rho cell_BG4/Figure 4D-E_Source data.tif (11).frames/1_0010_C001T001.tif]

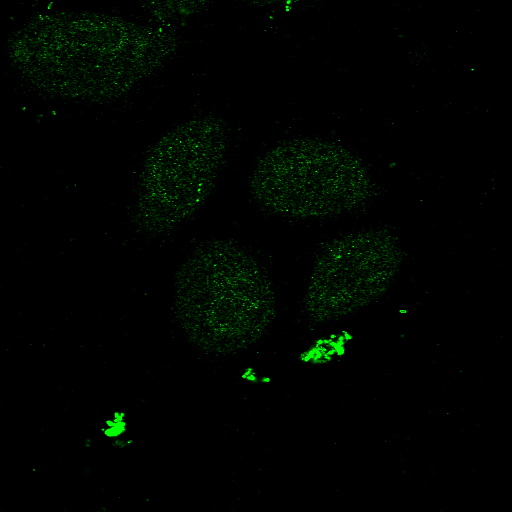

Supplement: Figure 4—source data 1. [file elife-69916-fig4-data1.zip › Figure4_Source data_BG4 localization to mtDNA/Figure 4D-E_IF images_rho cell_BG4/Figure 4D-E_Source data.tif (11).frames/1_0010_C003T001.tif]

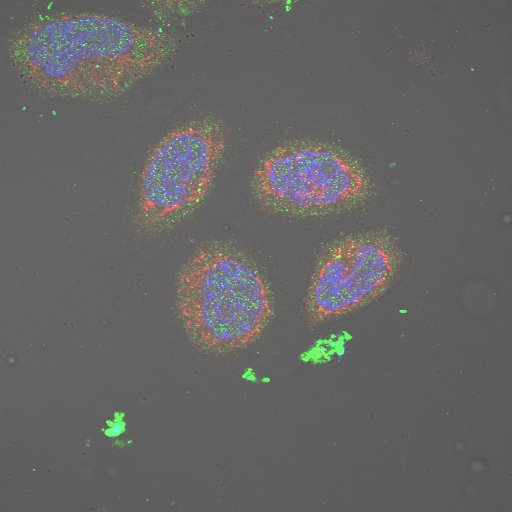

Supplement: Figure 4—source data 1. [file elife-69916-fig4-data1.zip › Figure4_Source data_BG4 localization to mtDNA/Figure 4D-E_IF images_rho cell_BG4/Figure 4D-E_Source data.tif (11).frames/1_0010_T001.tif]

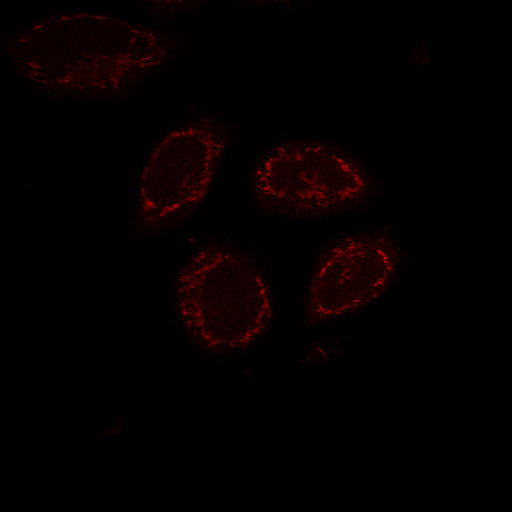

Supplement: Figure 4—source data 1. [file elife-69916-fig4-data1.zip › Figure4_Source data_BG4 localization to mtDNA/Figure 4D-E_IF images_rho cell_BG4/Figure 4D-E_Source data.tif (11).frames/1_0010_C002T001.tif]

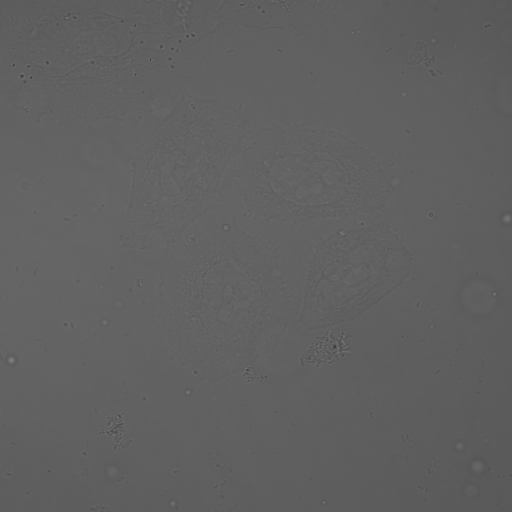

Supplement: Figure 4—source data 1. [file elife-69916-fig4-data1.zip › Figure4_Source data_BG4 localization to mtDNA/Figure 4D-E_IF images_rho cell_BG4/Figure 4D-E_Source data.tif (11).frames/1_0010_C004T001.tif]

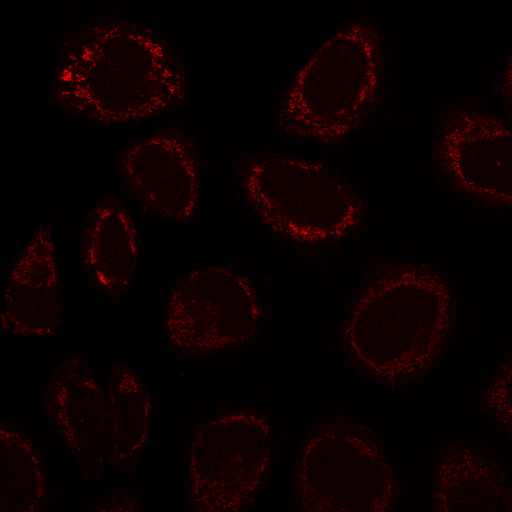

Supplement: Figure 4—source data 1. [file elife-69916-fig4-data1.zip › Figure4_Source data_BG4 localization to mtDNA/Figure 4D-E_IF images_rho cell_BG4/Figure 4D-E_Source data.tif (9).frames/1_0008_C002T001.tif]

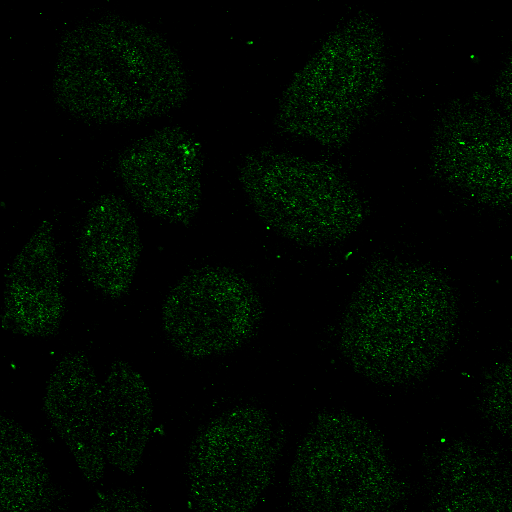

Supplement: Figure 4—source data 1. [file elife-69916-fig4-data1.zip › Figure4_Source data_BG4 localization to mtDNA/Figure 4D-E_IF images_rho cell_BG4/Figure 4D-E_Source data.tif (9).frames/1_0008_C003T001.tif]

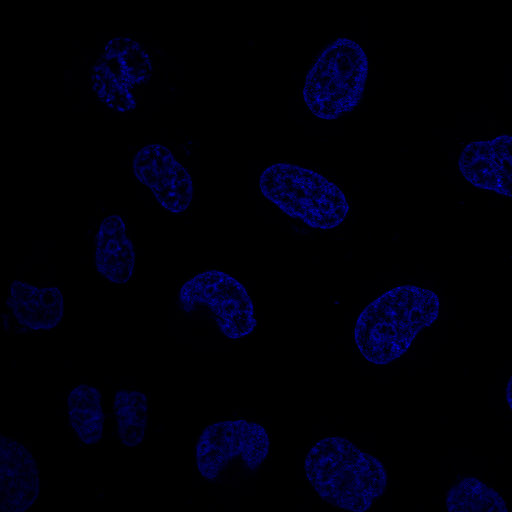

Supplement: Figure 4—source data 1. [file elife-69916-fig4-data1.zip › Figure4_Source data_BG4 localization to mtDNA/Figure 4D-E_IF images_rho cell_BG4/Figure 4D-E_Source data.tif (9).frames/1_0008_C001T001.tif]

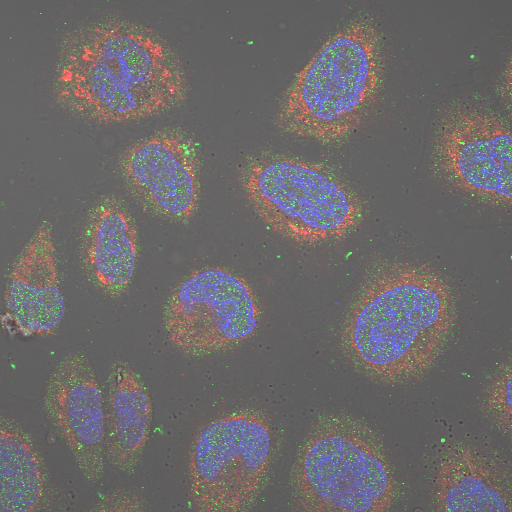

Supplement: Figure 4—source data 1. [file elife-69916-fig4-data1.zip › Figure4_Source data_BG4 localization to mtDNA/Figure 4D-E_IF images_rho cell_BG4/Figure 4D-E_Source data.tif (9).frames/1_0008_T001.tif]

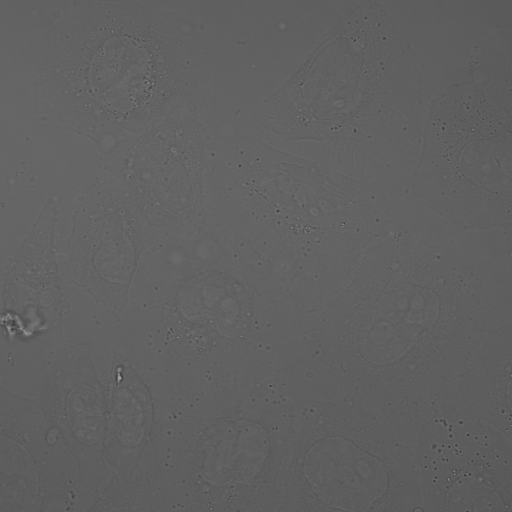

Supplement: Figure 4—source data 1. [file elife-69916-fig4-data1.zip › Figure4_Source data_BG4 localization to mtDNA/Figure 4D-E_IF images_rho cell_BG4/Figure 4D-E_Source data.tif (9).frames/1_0008_C004T001.tif]

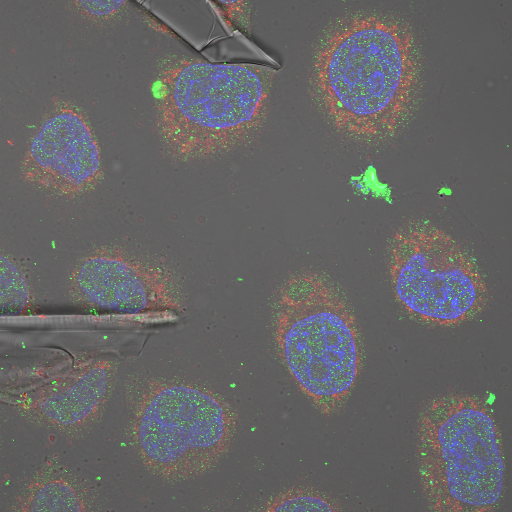

Supplement: Figure 4—source data 1. [file elife-69916-fig4-data1.zip › Figure4_Source data_BG4 localization to mtDNA/Figure 4D-E_IF images_rho cell_BG4/Figure 4D-E_Source data.tif.frames/1_T001.tif]

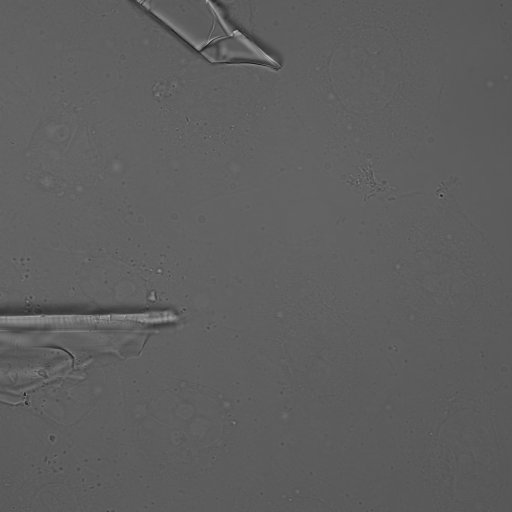

Supplement: Figure 4—source data 1. [file elife-69916-fig4-data1.zip › Figure4_Source data_BG4 localization to mtDNA/Figure 4D-E_IF images_rho cell_BG4/Figure 4D-E_Source data.tif.frames/1_C004T001.tif]

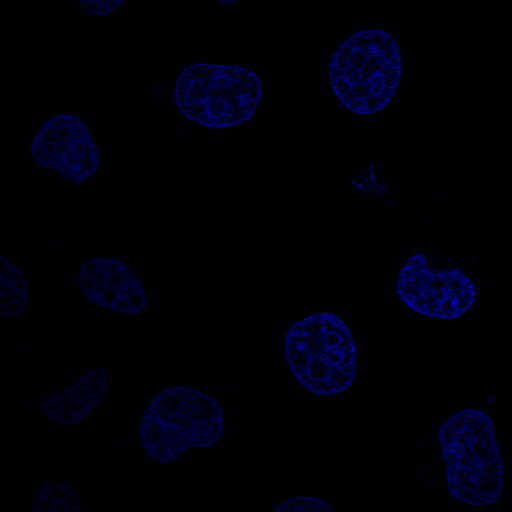

Supplement: Figure 4—source data 1. [file elife-69916-fig4-data1.zip › Figure4_Source data_BG4 localization to mtDNA/Figure 4D-E_IF images_rho cell_BG4/Figure 4D-E_Source data.tif.frames/1_C001T001.tif]

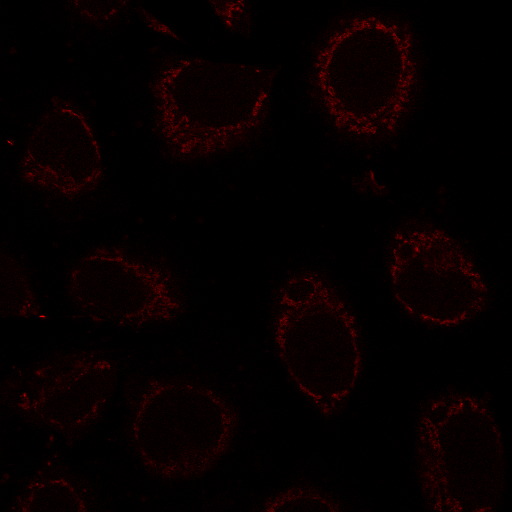

Supplement: Figure 4—source data 1. [file elife-69916-fig4-data1.zip › Figure4_Source data_BG4 localization to mtDNA/Figure 4D-E_IF images_rho cell_BG4/Figure 4D-E_Source data.tif.frames/1_C002T001.tif]

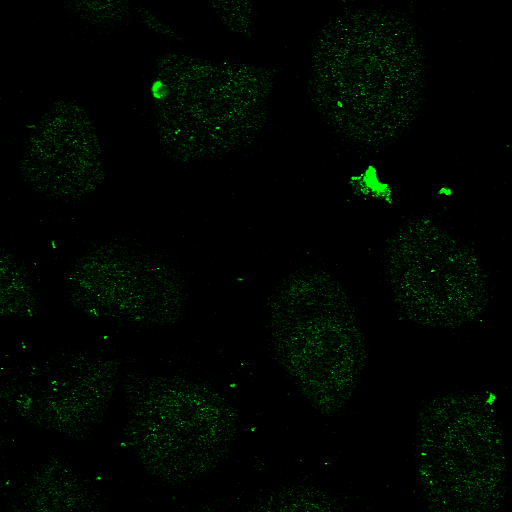

Supplement: Figure 4—source data 1. [file elife-69916-fig4-data1.zip › Figure4_Source data_BG4 localization to mtDNA/Figure 4D-E_IF images_rho cell_BG4/Figure 4D-E_Source data.tif.frames/1_C003T001.tif]

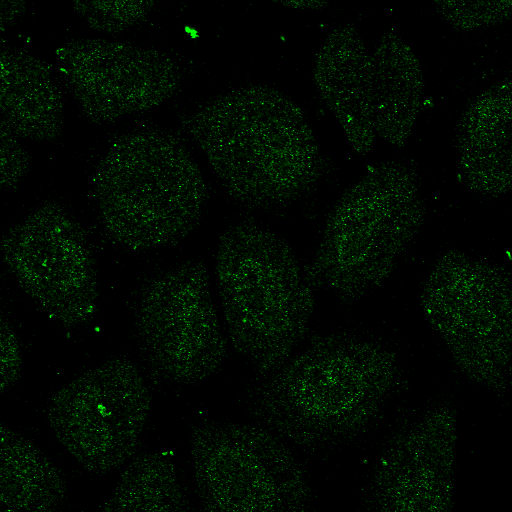

Supplement: Figure 4—source data 1. [file elife-69916-fig4-data1.zip › Figure4_Source data_BG4 localization to mtDNA/Figure 4D-E_IF images_rho cell_BG4/Figure 4D-E_Source data.tif (10).frames/1_0009_C003T001.tif]

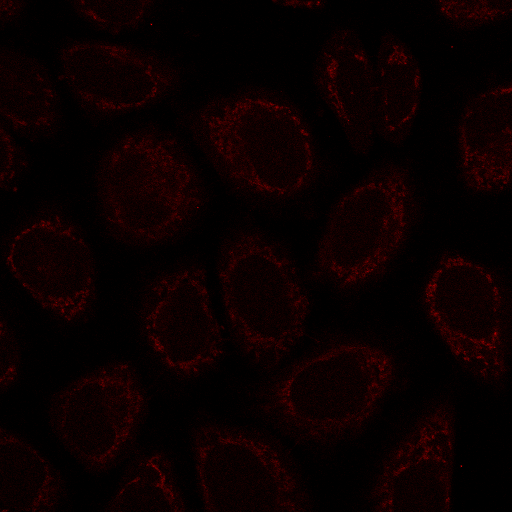

Supplement: Figure 4—source data 1. [file elife-69916-fig4-data1.zip › Figure4_Source data_BG4 localization to mtDNA/Figure 4D-E_IF images_rho cell_BG4/Figure 4D-E_Source data.tif (10).frames/1_0009_C002T001.tif]

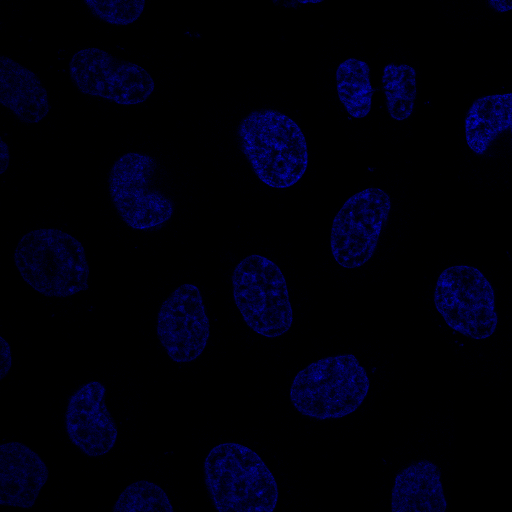

Supplement: Figure 4—source data 1. [file elife-69916-fig4-data1.zip › Figure4_Source data_BG4 localization to mtDNA/Figure 4D-E_IF images_rho cell_BG4/Figure 4D-E_Source data.tif (10).frames/1_0009_C001T001.tif]

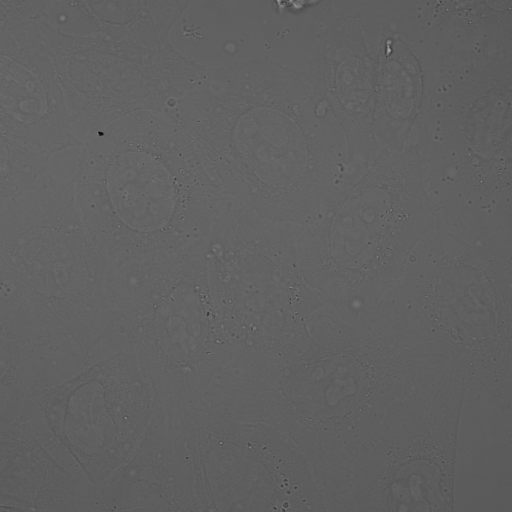

Supplement: Figure 4—source data 1. [file elife-69916-fig4-data1.zip › Figure4_Source data_BG4 localization to mtDNA/Figure 4D-E_IF images_rho cell_BG4/Figure 4D-E_Source data.tif (10).frames/1_0009_C004T001.tif]

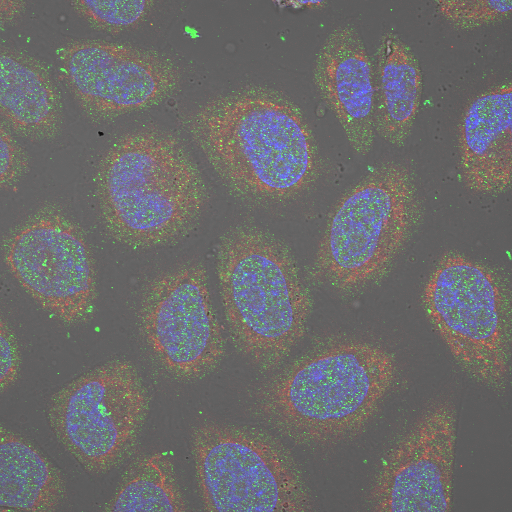

Supplement: Figure 4—source data 1. [file elife-69916-fig4-data1.zip › Figure4_Source data_BG4 localization to mtDNA/Figure 4D-E_IF images_rho cell_BG4/Figure 4D-E_Source data.tif (10).frames/1_0009_T001.tif]

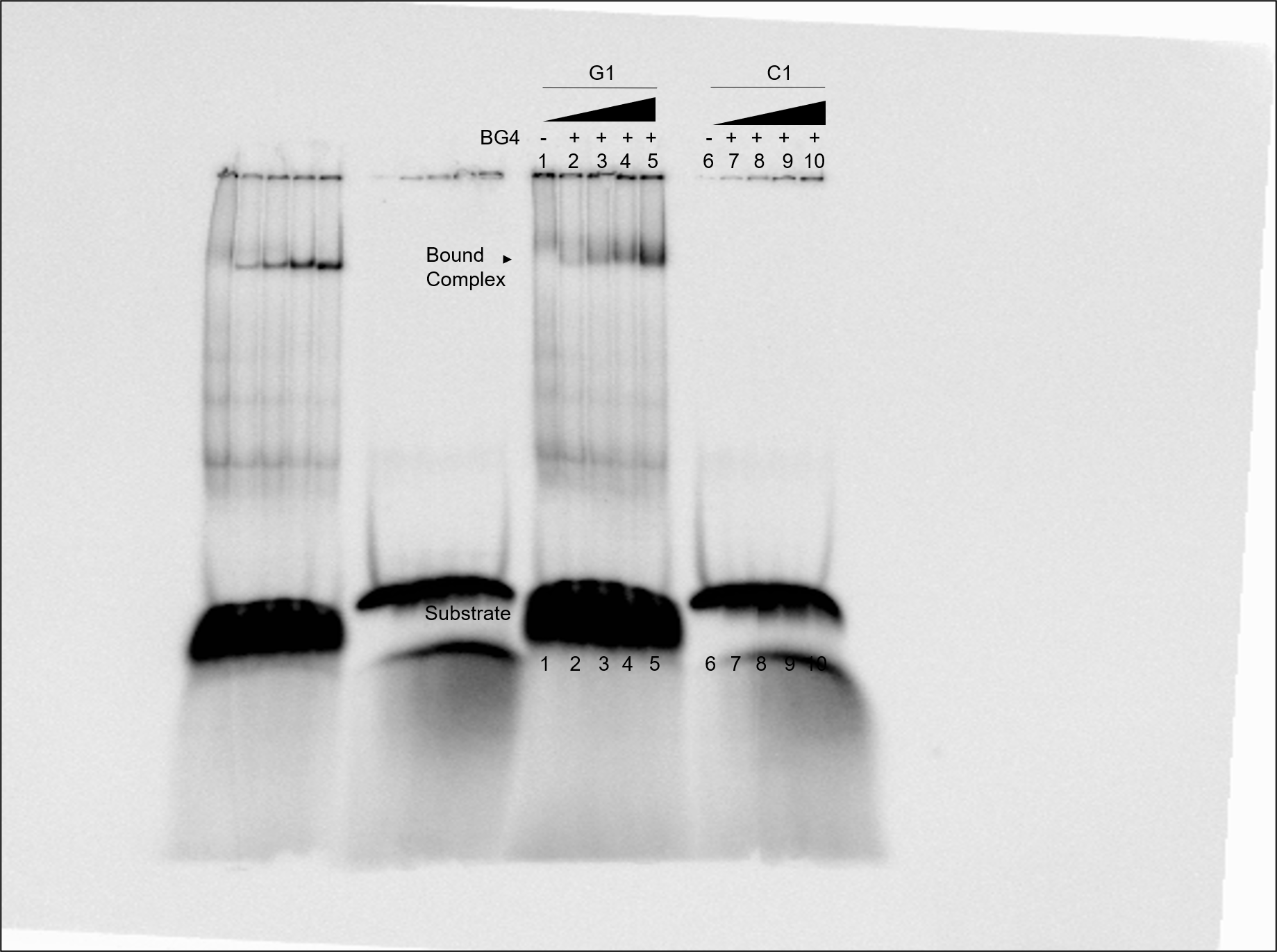

Supplement: Figure 4—source data 2. [file elife-69916-fig4-data2.zip › Figure 4_Source data_Supplementary/FigureS4B_Source data/Figure S4B_Gel profile_BG4 binding_mitochondrial region binding for G and C strand.tif]

## Slide 1
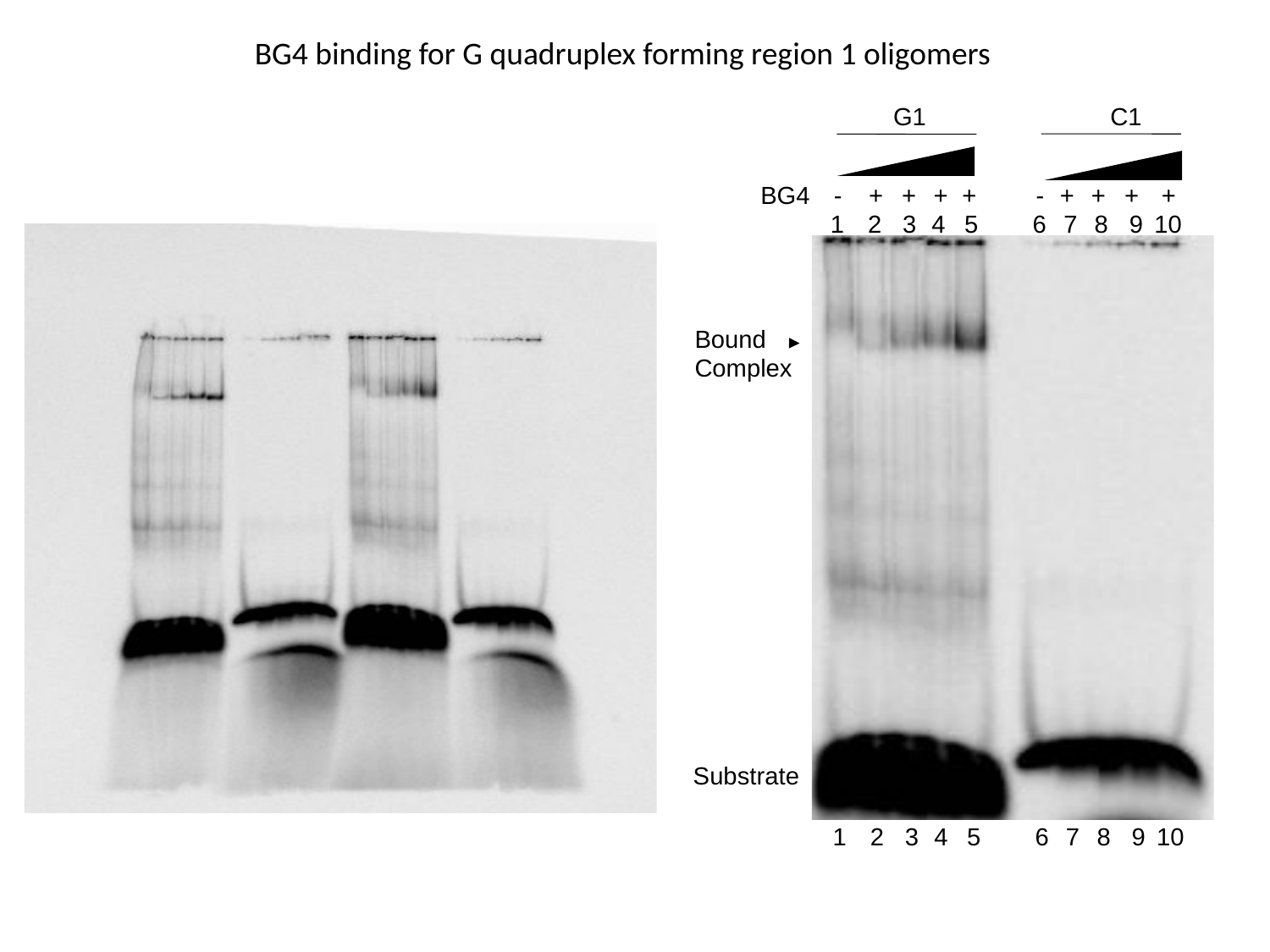

BG4 binding for G quadruplex forming region 1 oligomers
C1
G1
BG4
-
+
+
+
+
-
+
+
+
+
1
2
3
4
5
6
7
8
9
10
1
2
3
4
5
6
7
8
9
10
Bound Complex
Substrate

Supplement: Figure 4—source data 2. [file elife-69916-fig4-data2.zip › Figure 4_Source data_Supplementary/FigureS4B_Source data/Figure S4B_Gel profile_BG4 binding_mitochondrial region binding.pptx]

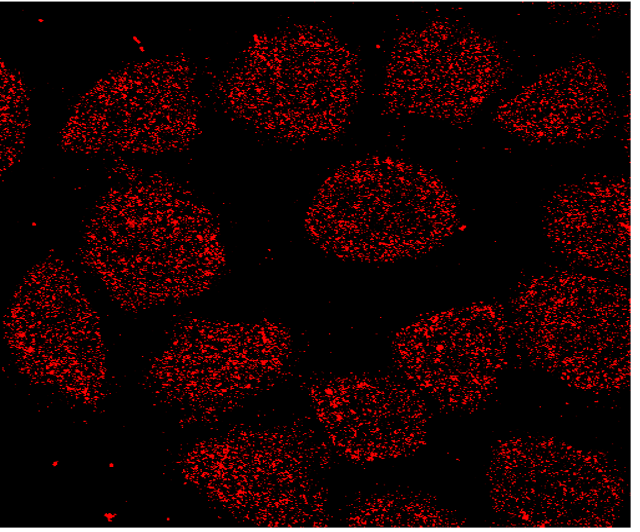

Supplement: Figure 4—source data 2. [file elife-69916-fig4-data2.zip › Figure 4_Source data_Supplementary/FigureS4A_Representative image_HeLa cells_BG4_mitotracker green/Figure S4A_Represenatative image_HeLa cells_BG4_Mitotracker green_BG4.tif]

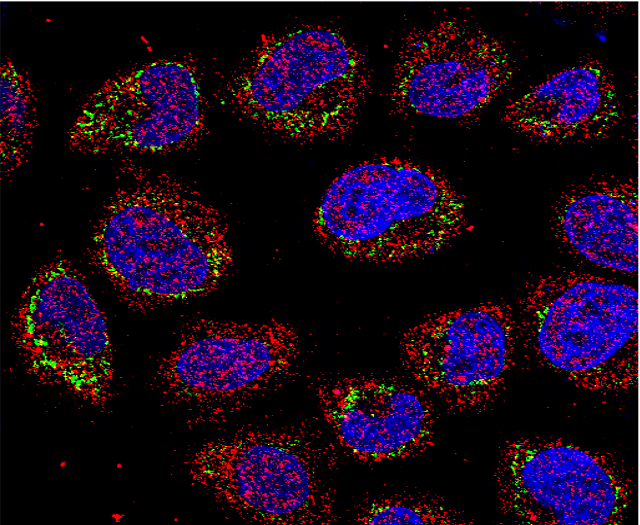

Supplement: Figure 4—source data 2. [file elife-69916-fig4-data2.zip › Figure 4_Source data_Supplementary/FigureS4A_Representative image_HeLa cells_BG4_mitotracker green/Figure S4A_Represenatative image_HeLa cells_BG4_Mitotracker green_merged.tif]

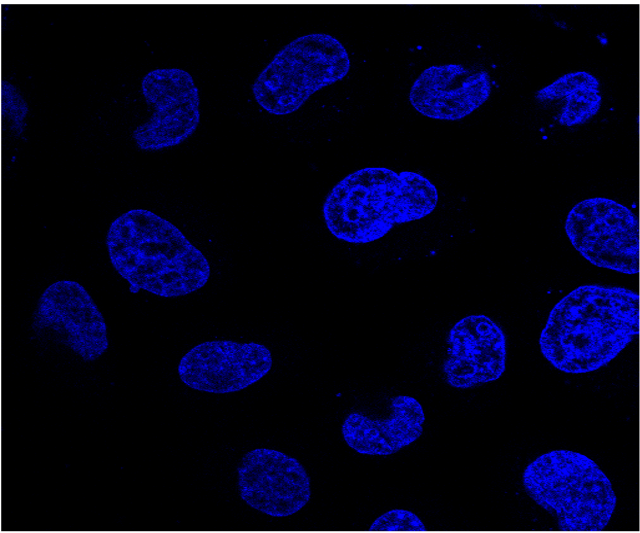

Supplement: Figure 4—source data 2. [file elife-69916-fig4-data2.zip › Figure 4_Source data_Supplementary/FigureS4A_Representative image_HeLa cells_BG4_mitotracker green/Figure S4A_Represenatative image_HeLa cells_BG4_Mitotracker green_nucleus.tif]

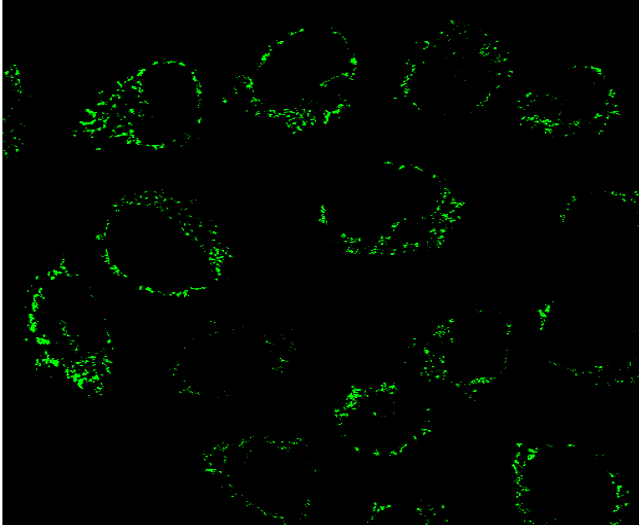

Supplement: Figure 4—source data 2. [file elife-69916-fig4-data2.zip › Figure 4_Source data_Supplementary/FigureS4A_Representative image_HeLa cells_BG4_mitotracker green/Figure S4A_Represenatative image_HeLa cells_BG4_Mitotracker green_mitotracker green.tif]

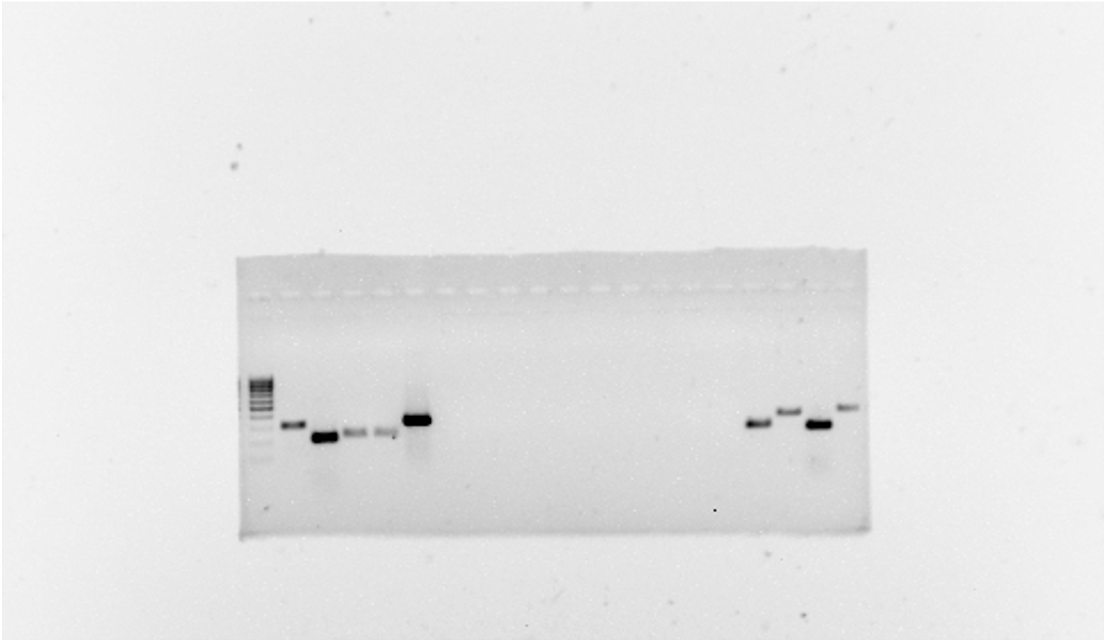

Supplement: Figure 5—source data 1. [file elife-69916-fig5-data1.zip › Figure5_Source data_BG4 ChIP pulldown/Figure 5C_Gel profile_mitoChIP_BG4 pulldown/Figure 2G_Gel profile_mitoChIP_BG4 pull down.Tif]

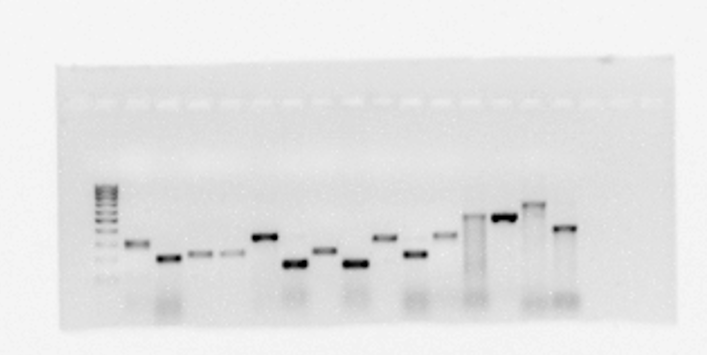

Supplement: Figure 5—source data 1. [file elife-69916-fig5-data1.zip › Figure5_Source data_BG4 ChIP pulldown/Figure 5C_Gel profile_mitoChIP_BG4 pulldown/Figure 2G_Gel profile_mitoChIP_BG4_Input.Tif]

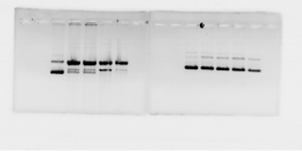

Supplement: Figure 6—source data 1. [file elife-69916-fig6-data1.zip › Figure6_Sourcedata_activity assay mitochondrial extracts/Figure 6B_Activity assay of mitochondrial extract/Figure 6B_Activity assay of mitochondrial extracts for wild type and mutatnt plasmid.tif]

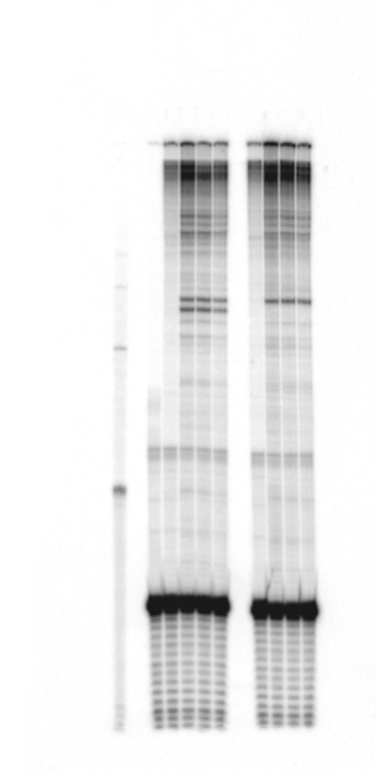

Supplement: Figure 6—source data 1. [file elife-69916-fig6-data1.zip › Figure6_Sourcedata_activity assay mitochondrial extracts/Figure 6E_Primer extension_Mitochondrial extracts_pDI1 vs pDI2/Figure 6E_primer extension_mitochondrial testes extracts on pDI1 and pDI2.tif]

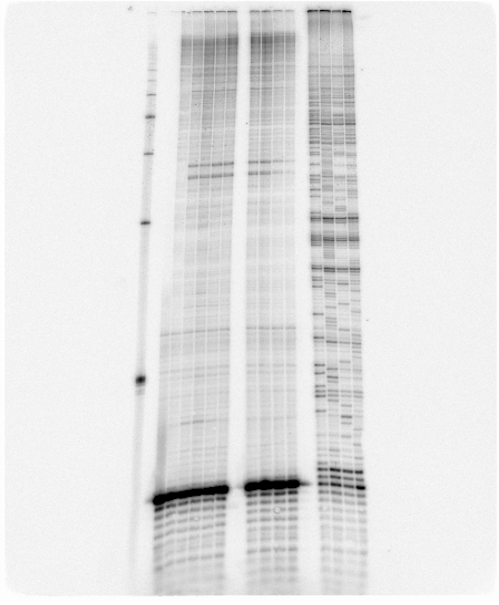

Supplement: Figure 6—source data 1. [file elife-69916-fig6-data1.zip › Figure6_Sourcedata_activity assay mitochondrial extracts/Figure 6D_Primer extension_mitochondrial extracts/Figure 6D_primer extension_mitochondrial testes and spleen extracts on pDI1.tif]

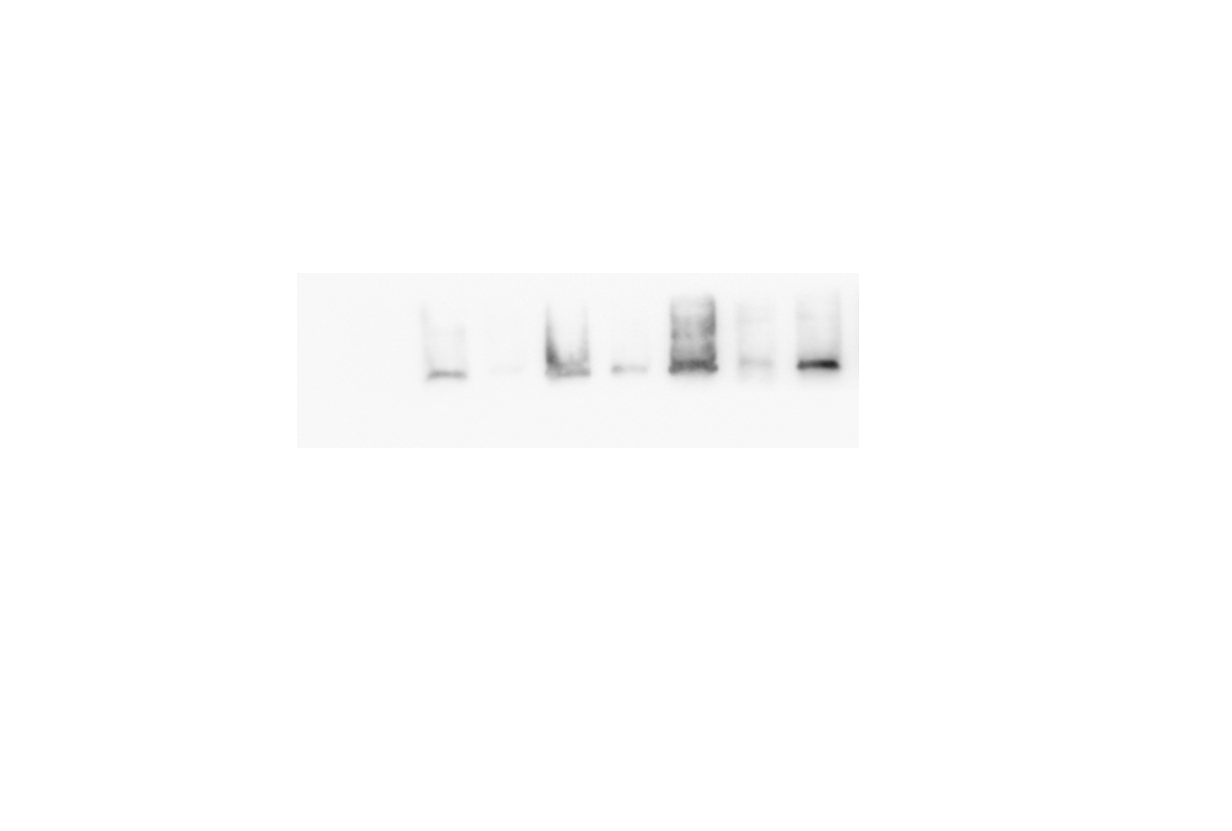

Supplement: Figure 6—source data 1. [file elife-69916-fig6-data1.zip › Figure6_Sourcedata_activity assay mitochondrial extracts/Figure 6A_western blotting_mitochondrial extract purity check/Figure 6A_western blotting_Cytochrome C_ME vs CE extracts.tif]

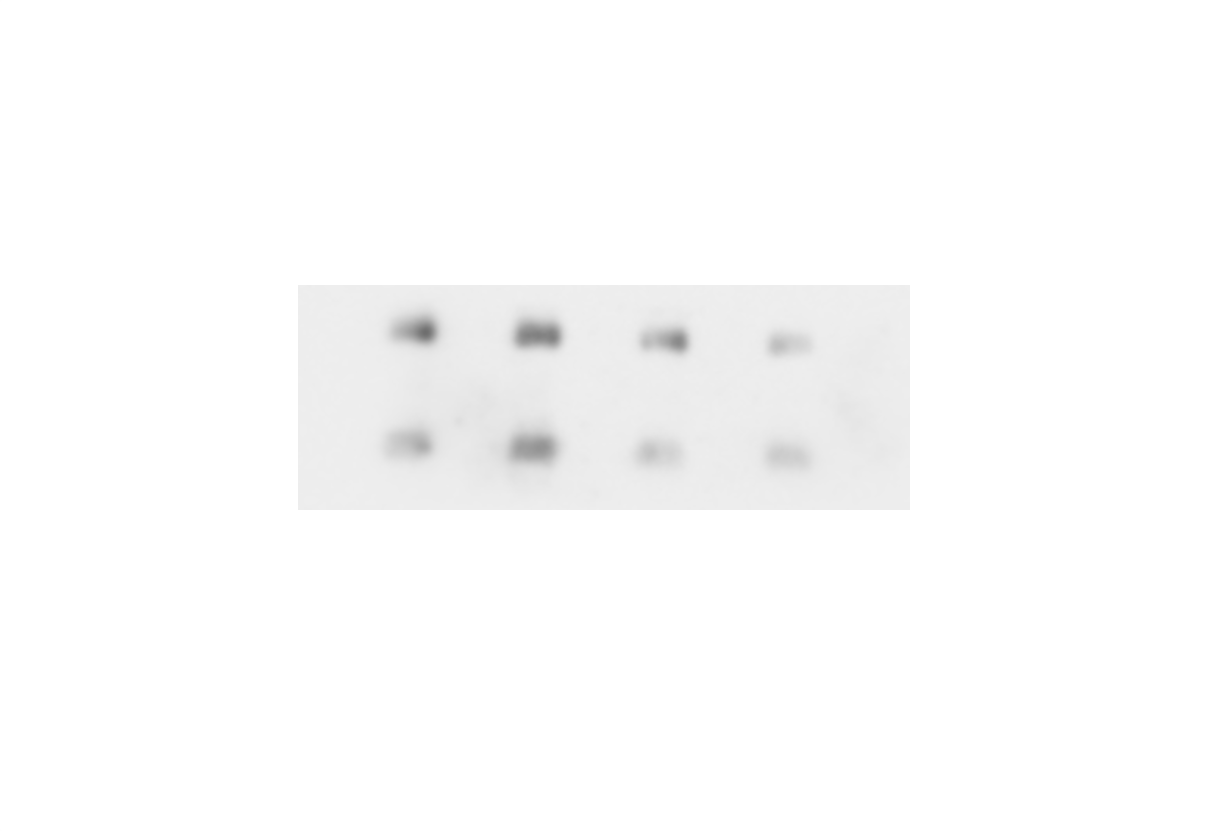

Supplement: Figure 6—source data 1. [file elife-69916-fig6-data1.zip › Figure6_Sourcedata_activity assay mitochondrial extracts/Figure 6A_western blotting_mitochondrial extract purity check/Figure 6A_western blotting_PCNA_ME vs CE extracts.tif]

## Slide 1
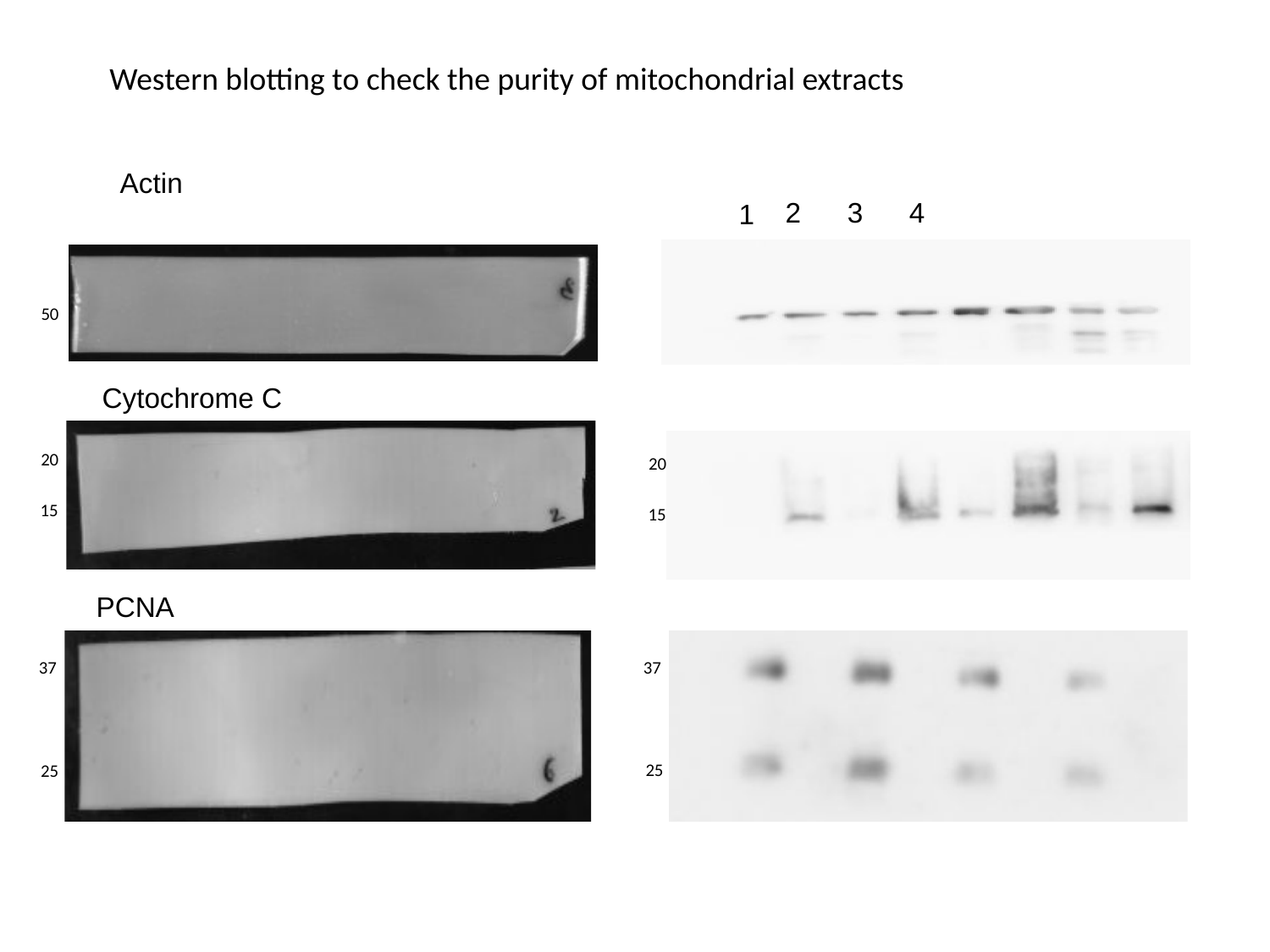

Western blotting to check the purity of mitochondrial extracts
Actin
4
2
3
1
50
Cytochrome C
20
20
15
15
PCNA
37
37
25
25

Supplement: Figure 6—source data 1. [file elife-69916-fig6-data1.zip › Figure6_Sourcedata_activity assay mitochondrial extracts/Figure 6A_western blotting_mitochondrial extract purity check/Figure 6A_.pptx]

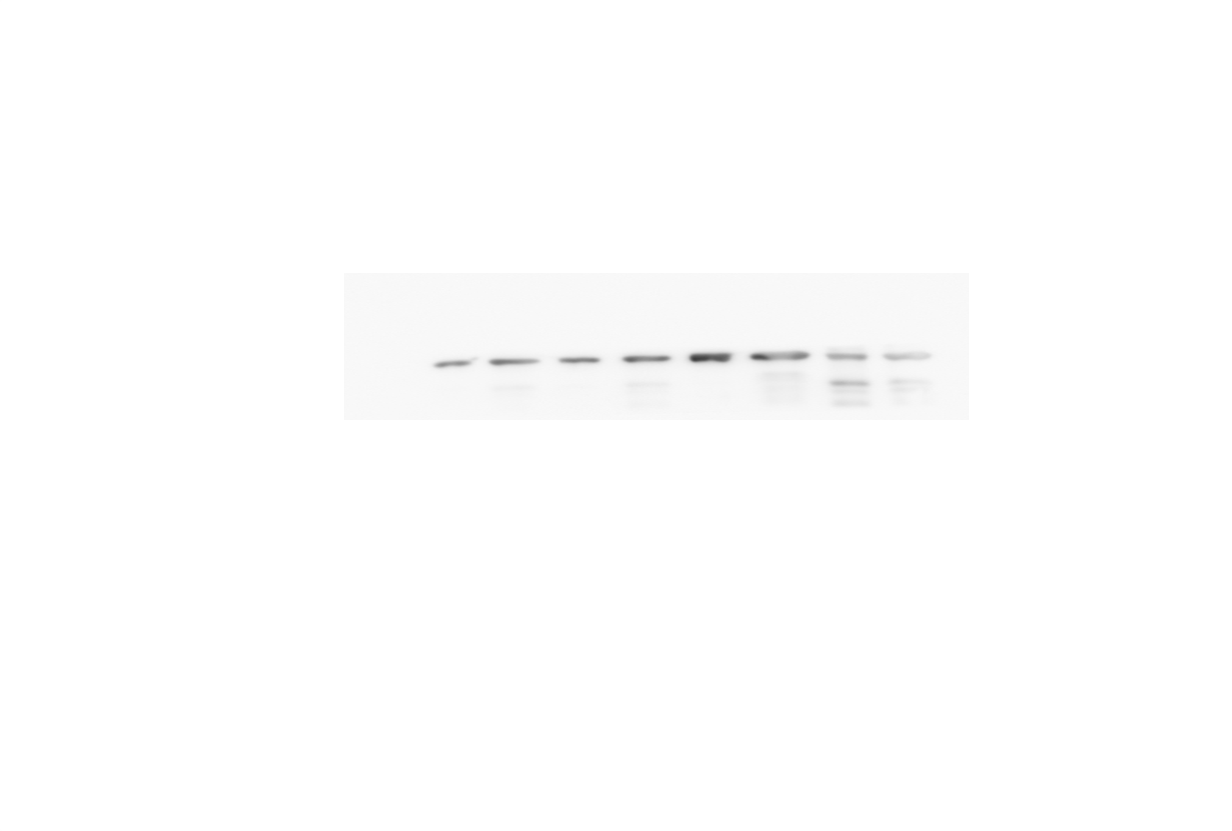

Supplement: Figure 6—source data 1. [file elife-69916-fig6-data1.zip › Figure6_Sourcedata_activity assay mitochondrial extracts/Figure 6A_western blotting_mitochondrial extract purity check/Figure 6A_western blotting_Actin_ME vs CE extracts.tif]

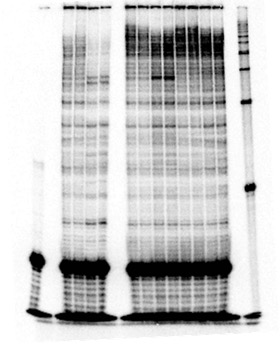

Supplement: Figure 6—source data 2. [file elife-69916-fig6-data2.zip › Figure6_Sourcedata_Supplementary/Figure S6B_Cleavage assay on plasmid bearing wildtype and mutant G4 sequence.jpg]

# SciGenom Trace Viewer

Sample :pDI1\_VKK11\_10017-3\_5557  
Trim Start :22  
Trim End :972  
Qv20 Bases :950

Run start: 2013/06/11 09:46:32  
Run stop: 2013/06/11 12:02:06  
PDF created: 2013/06/11 12:18:35

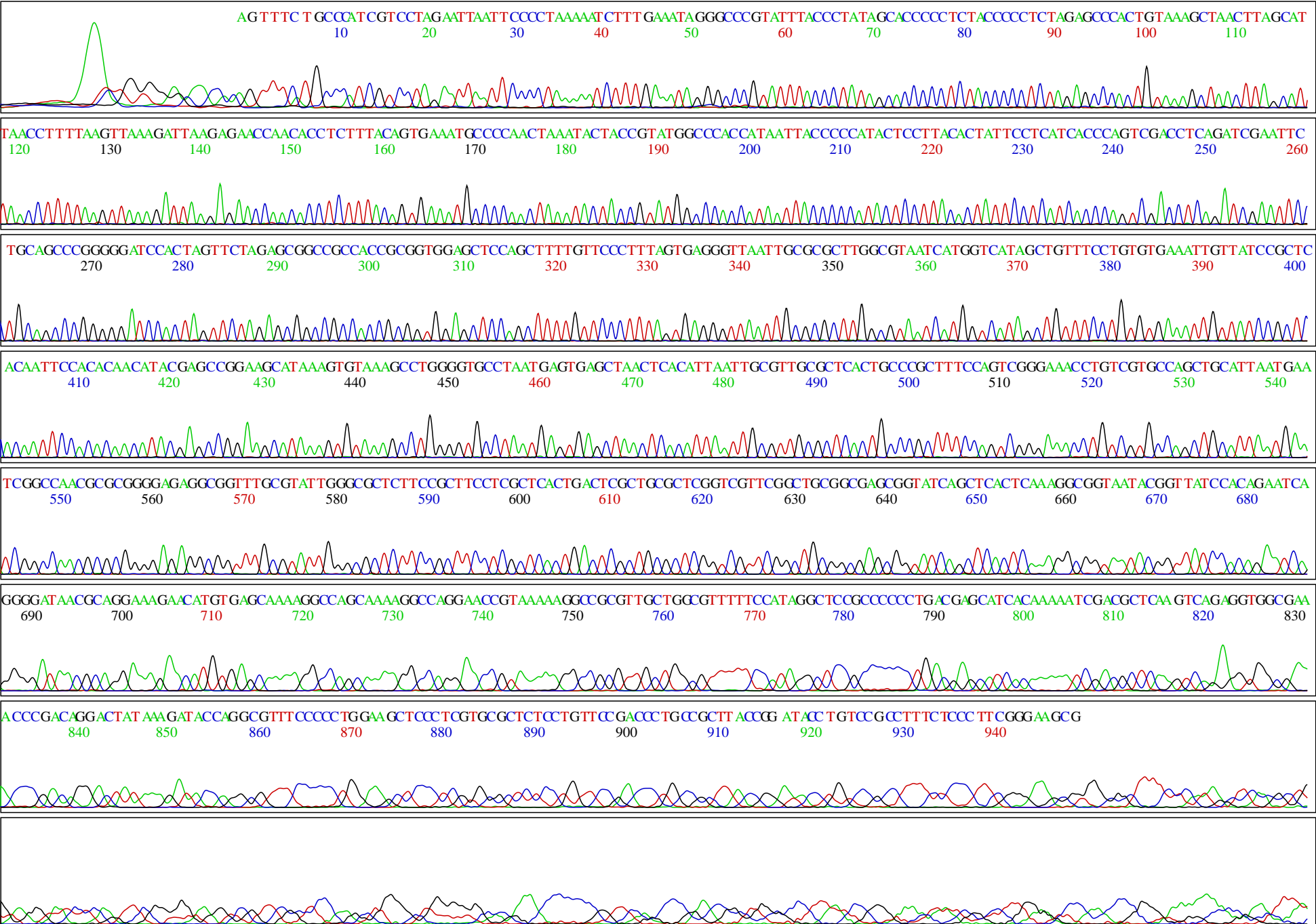

Supplement: Figure 6—source data 2. [file elife-69916-fig6-data2.zip › Figure6_Sourcedata_Supplementary/Figure S6A_pDI1 sequencing.pdf]

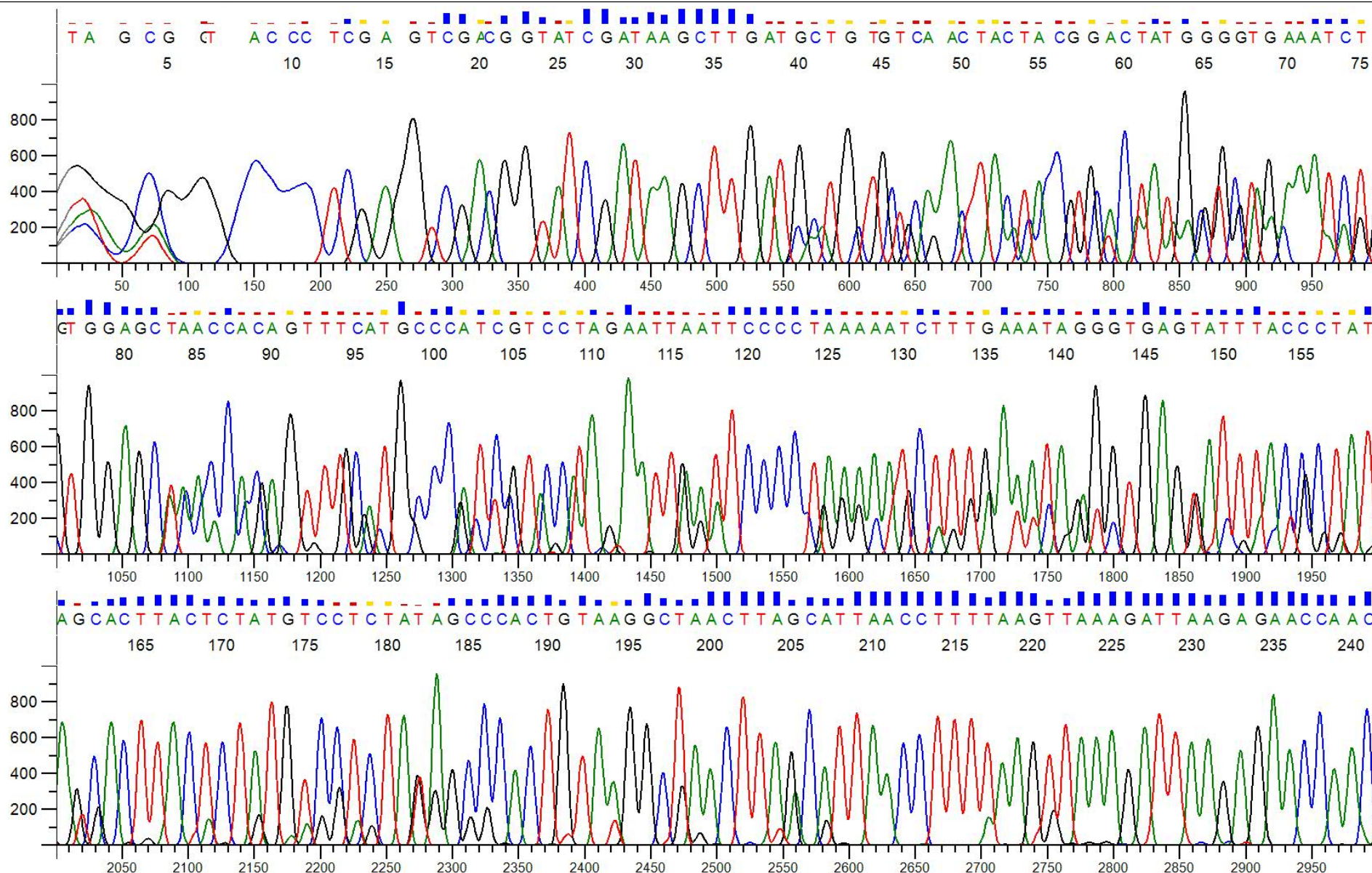

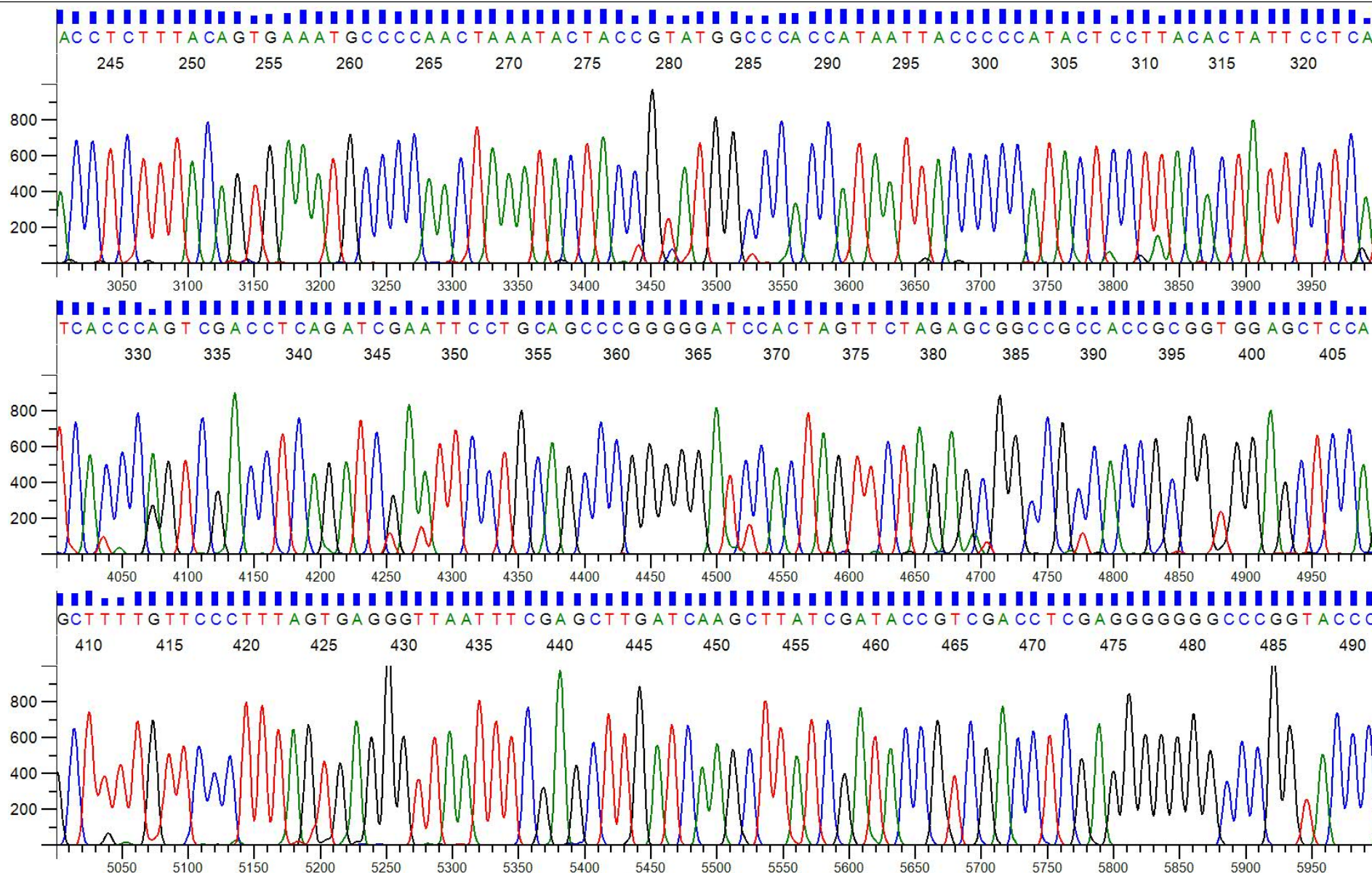

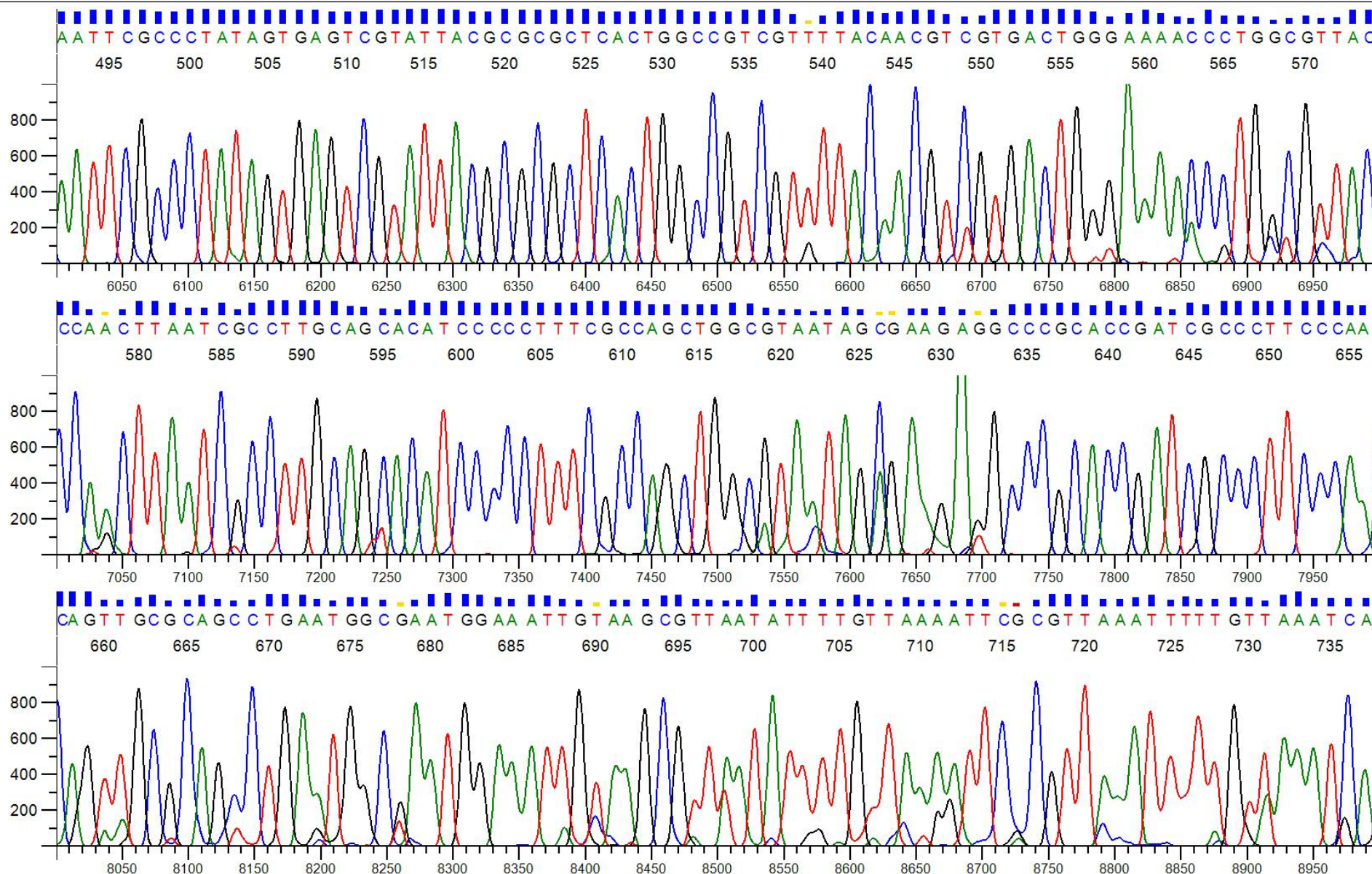

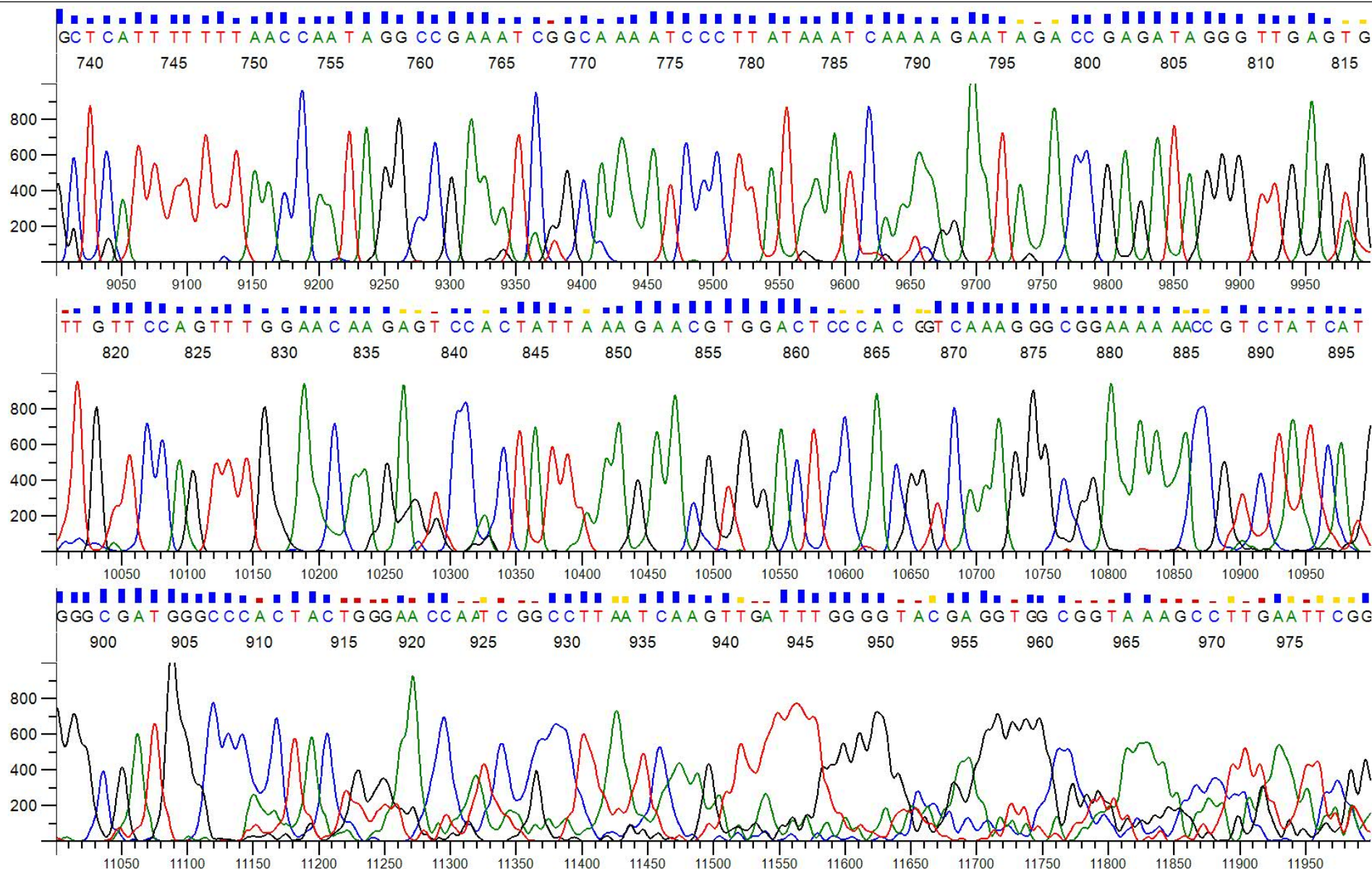

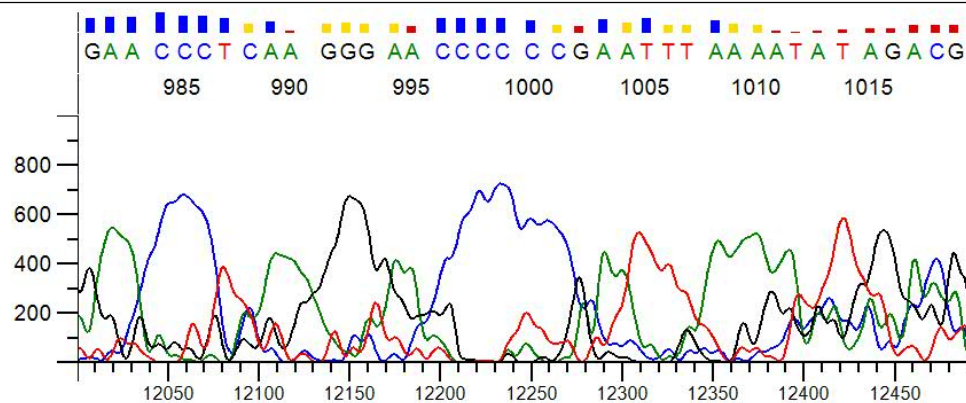

Supplement: Figure 6—source data 2. [file elife-69916-fig6-data2.zip › Figure6_Sourcedata_Supplementary/Figure S6A_pDR4 sequencing.pdf]

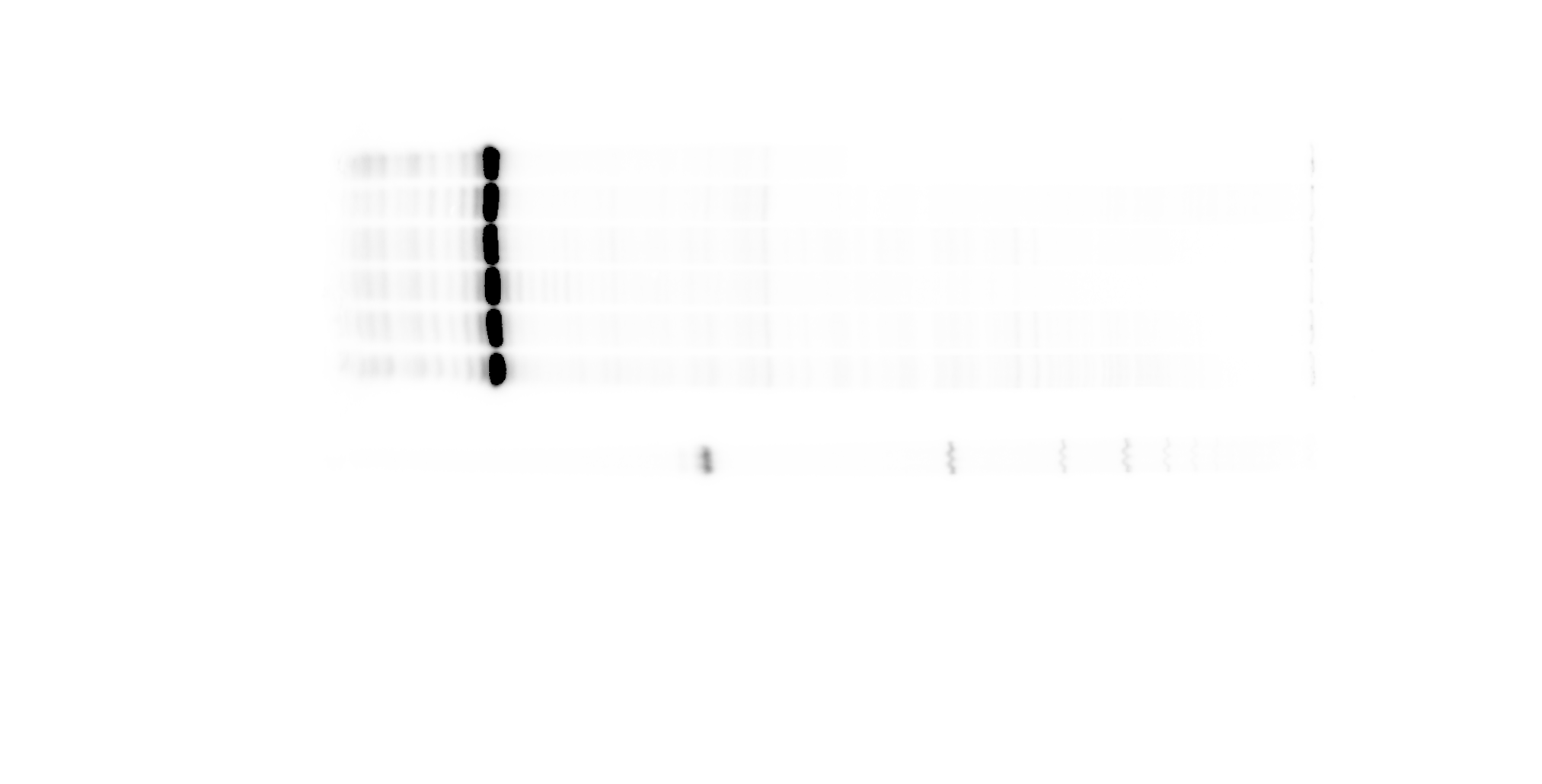

Supplement: Figure 7—source data 1. [file elife-69916-fig7-data1.zip › Figure 7_Sourcedata_activity of Endonuclease G/Figure 7J_Primer extension_IP_reconstitution/Figure 7J_primer extn_IP_reconstitution_Endo-G.tif]

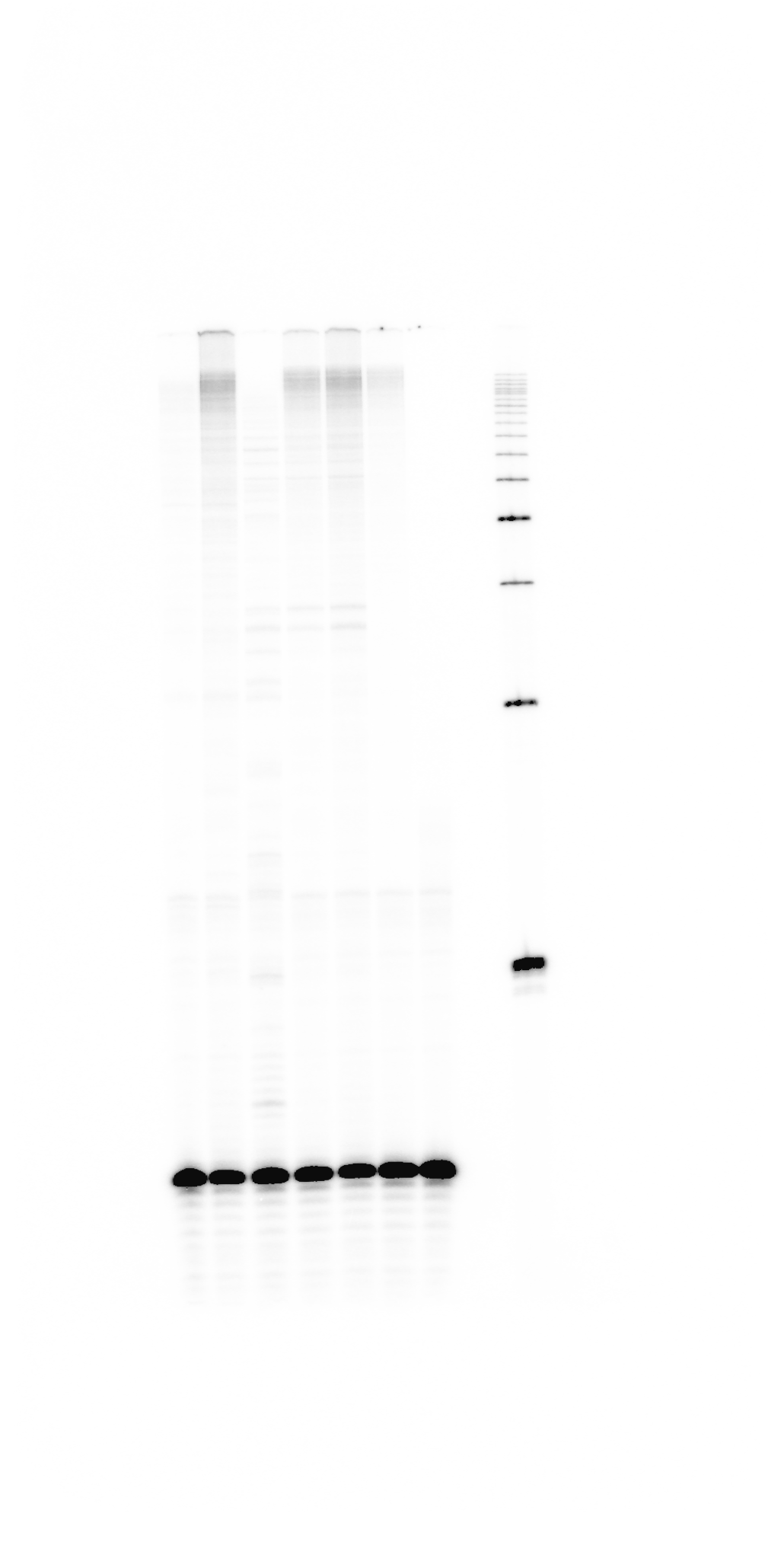

Supplement: Figure 7—source data 1. [file elife-69916-fig7-data1.zip › Figure 7_Sourcedata_activity of Endonuclease G/Figure 7H_Gel_Primer extension_Different endonuclease immunodepletion/FIgure 7H_Gel_Primer extension_Different nucleases IP.tif]

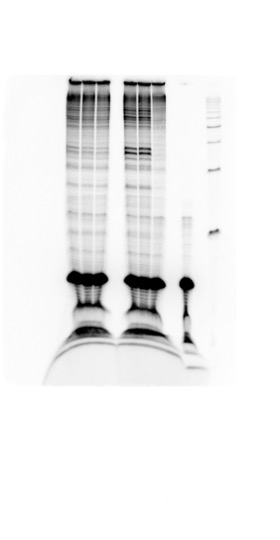

Supplement: Figure 7—source data 1. [file elife-69916-fig7-data1.zip › Figure 7_Sourcedata_activity of Endonuclease G/Figure 7D_Gel profile for wild type vs mutant EndoG cleavage assay /Figure7D_Gel profile of cleavage assay using either wt or mutant EndoG.jpg]

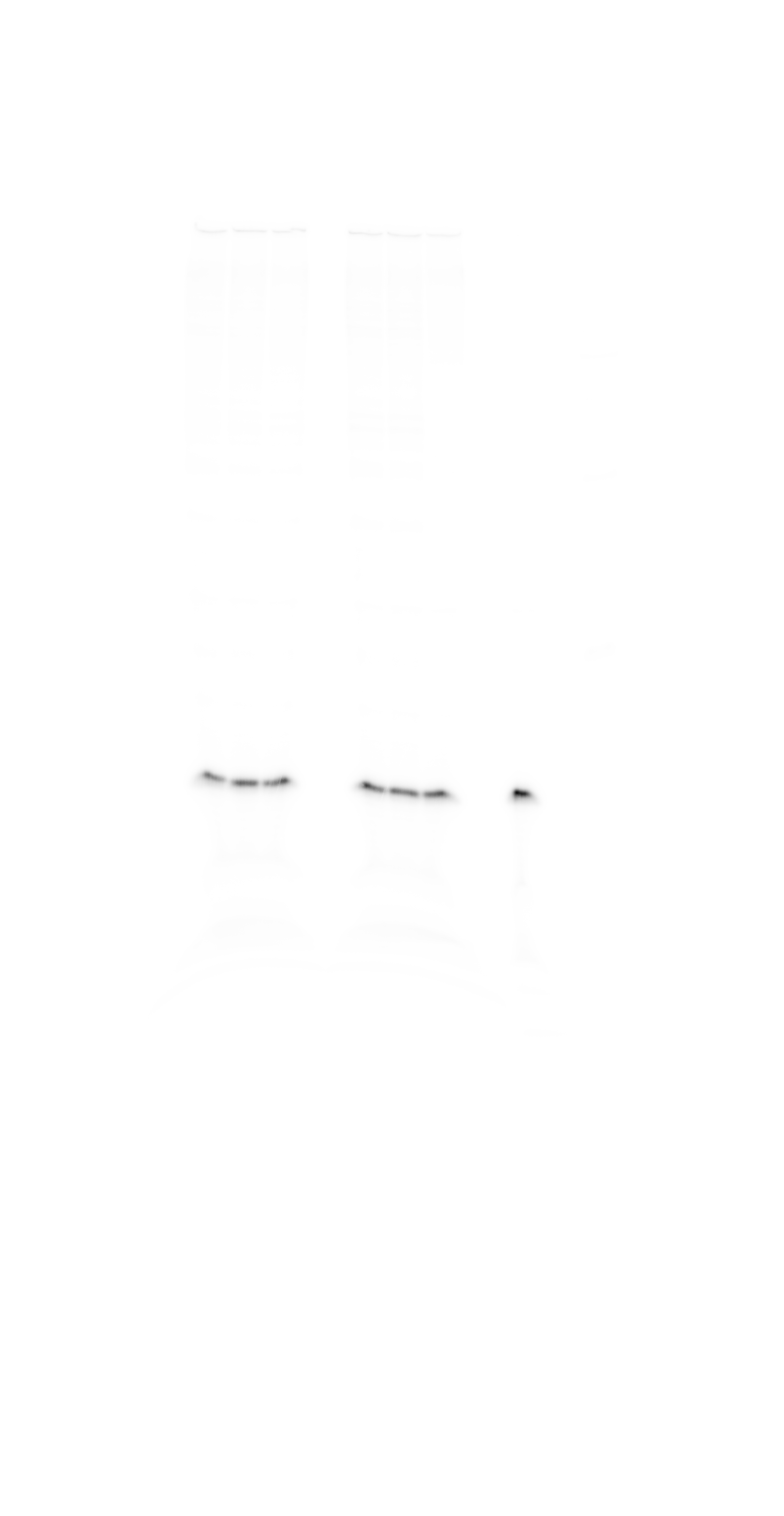

Supplement: Figure 7—source data 1. [file elife-69916-fig7-data1.zip › Figure 7_Sourcedata_activity of Endonuclease G/Figure 7D_Gel profile for wild type vs mutant EndoG cleavage assay /Figure 7D_Gel profile of leavage assay using either wtEndoG or mutant.tif]

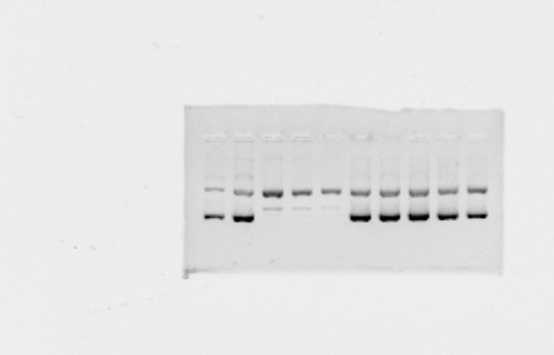

Supplement: Figure 7—source data 1. [file elife-69916-fig7-data1.zip › Figure 7_Sourcedata_activity of Endonuclease G/Figure 7A_Activity assay_purified Endonuclease/Figure 7A_Activity assay of purified EndoG for wild type and mutatnt plasmid.tif]

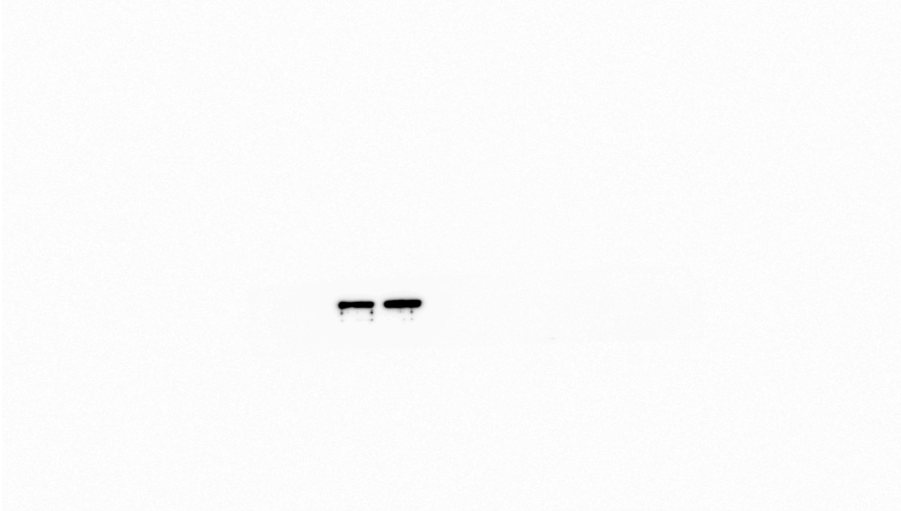

Supplement: Figure 7—source data 1. [file elife-69916-fig7-data1.zip › Figure 7_Sourcedata_activity of Endonuclease G/Figure 7G_western_immunodepletion of MGME1/Figure 7G_Gel image profile for Actin_Immunodepletion of MGME1 extracts.tif]

## Slide 1
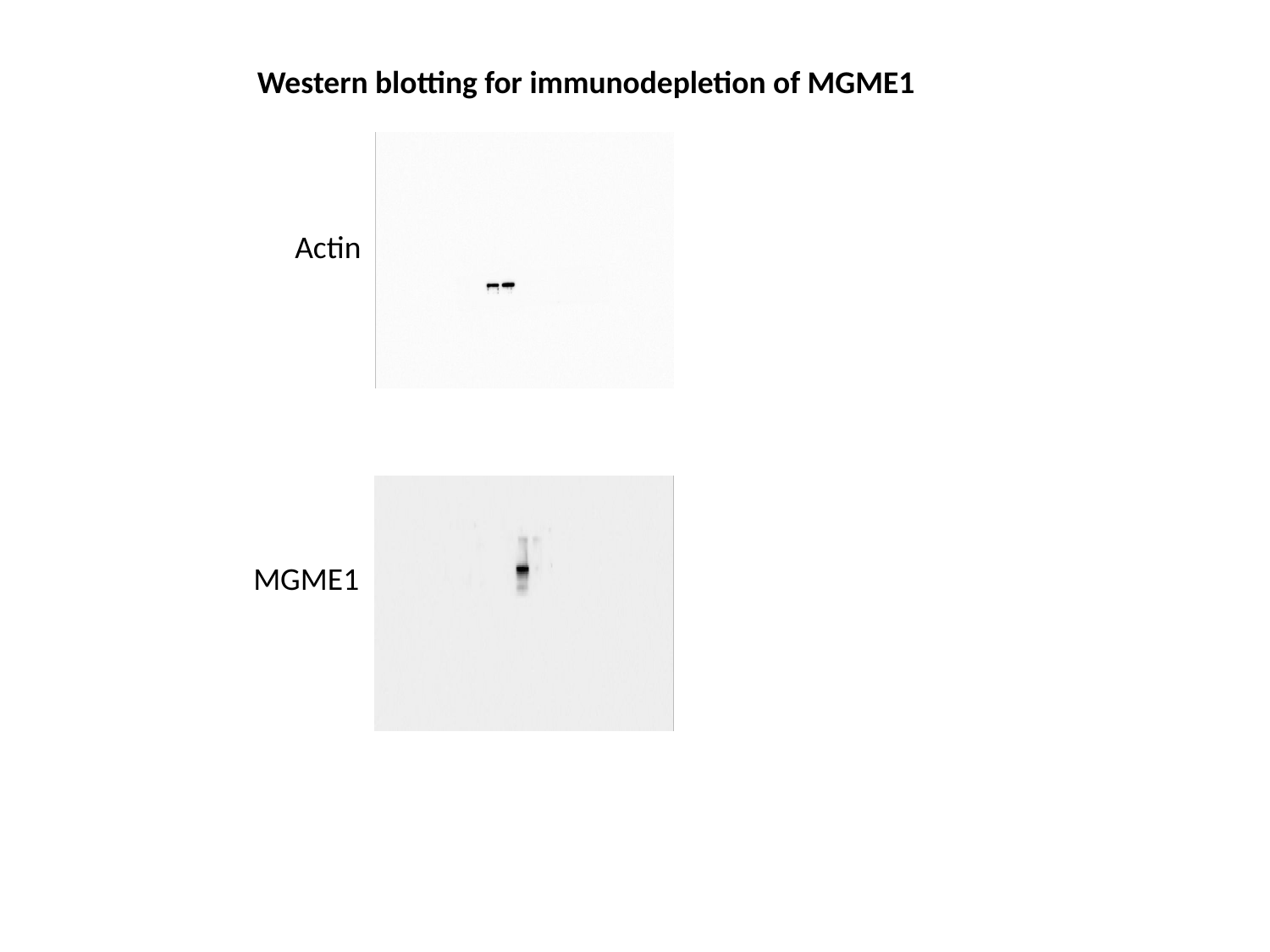

Western blotting for immunodepletion of MGME1
Actin
MGME1

Supplement: Figure 7—source data 1. [file elife-69916-fig7-data1.zip › Figure 7_Sourcedata_activity of Endonuclease G/Figure 7G_western_immunodepletion of MGME1/Figure 7G_Source data_labeled.pptx]

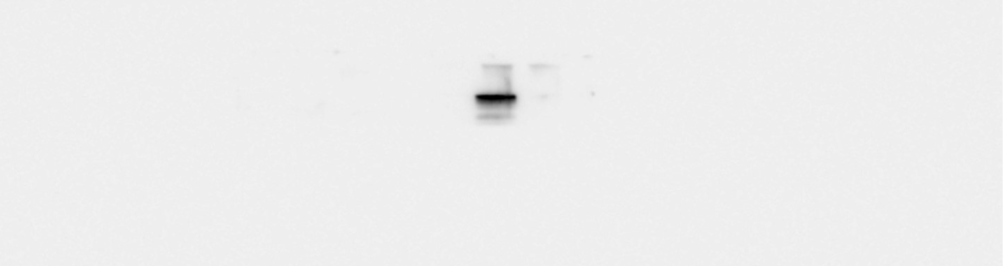

Supplement: Figure 7—source data 1. [file elife-69916-fig7-data1.zip › Figure 7_Sourcedata_activity of Endonuclease G/Figure 7G_western_immunodepletion of MGME1/Figure 7G_Gel image profile for MGME1_Immunodepletion of MGME1 extracts.tif]

## Slide 1
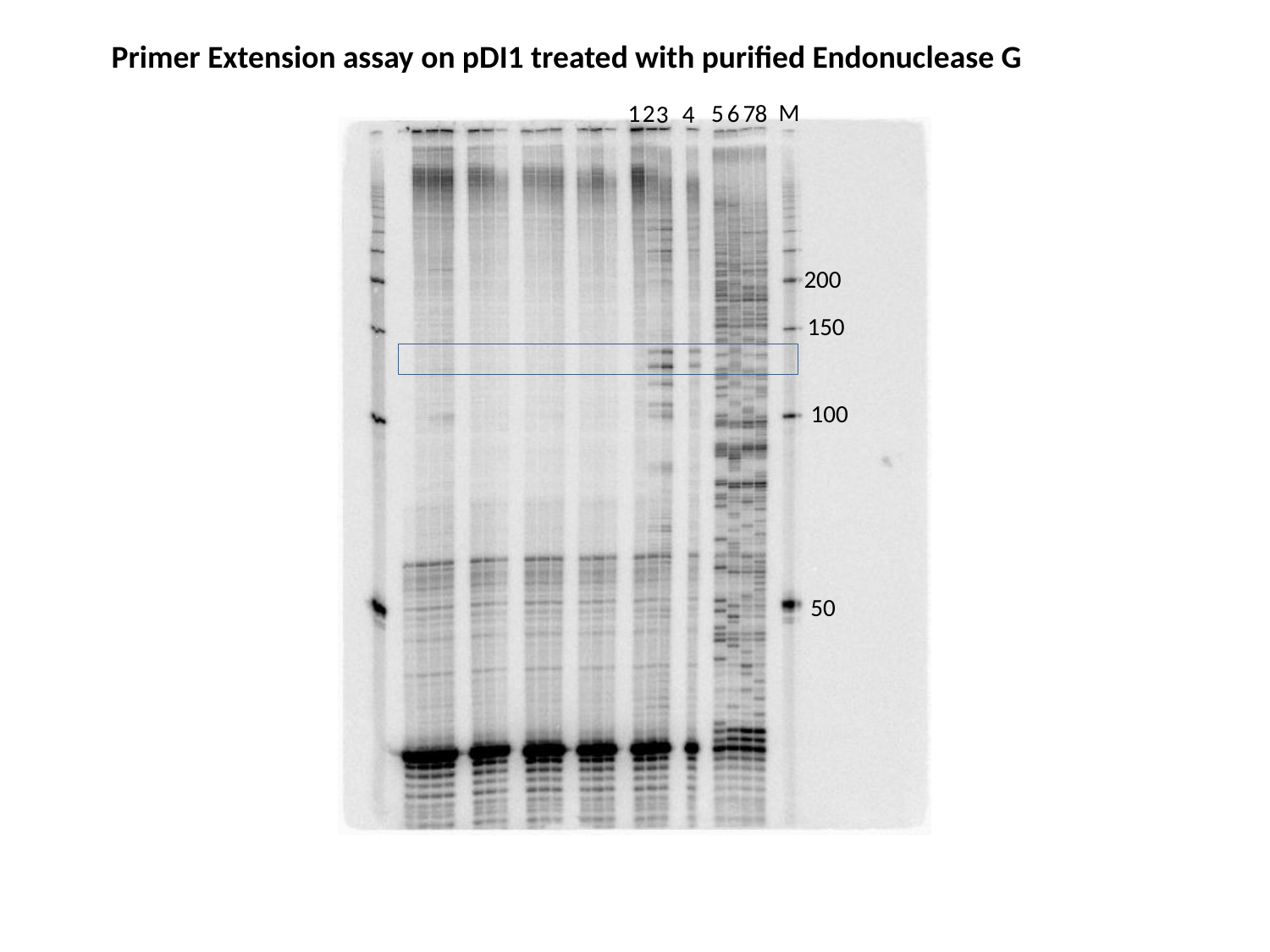

Primer Extension assay on pDI1 treated with purified Endonuclease G
M
5
7
8
6
1
2
3
4
200
150
100
50

Supplement: Figure 7—source data 1. [file elife-69916-fig7-data1.zip › Figure 7_Sourcedata_activity of Endonuclease G/Figure 7C_primer extension_Endonuclease screening/Figure 7C_primer extension_purified_Endonuclease screening.pptx]

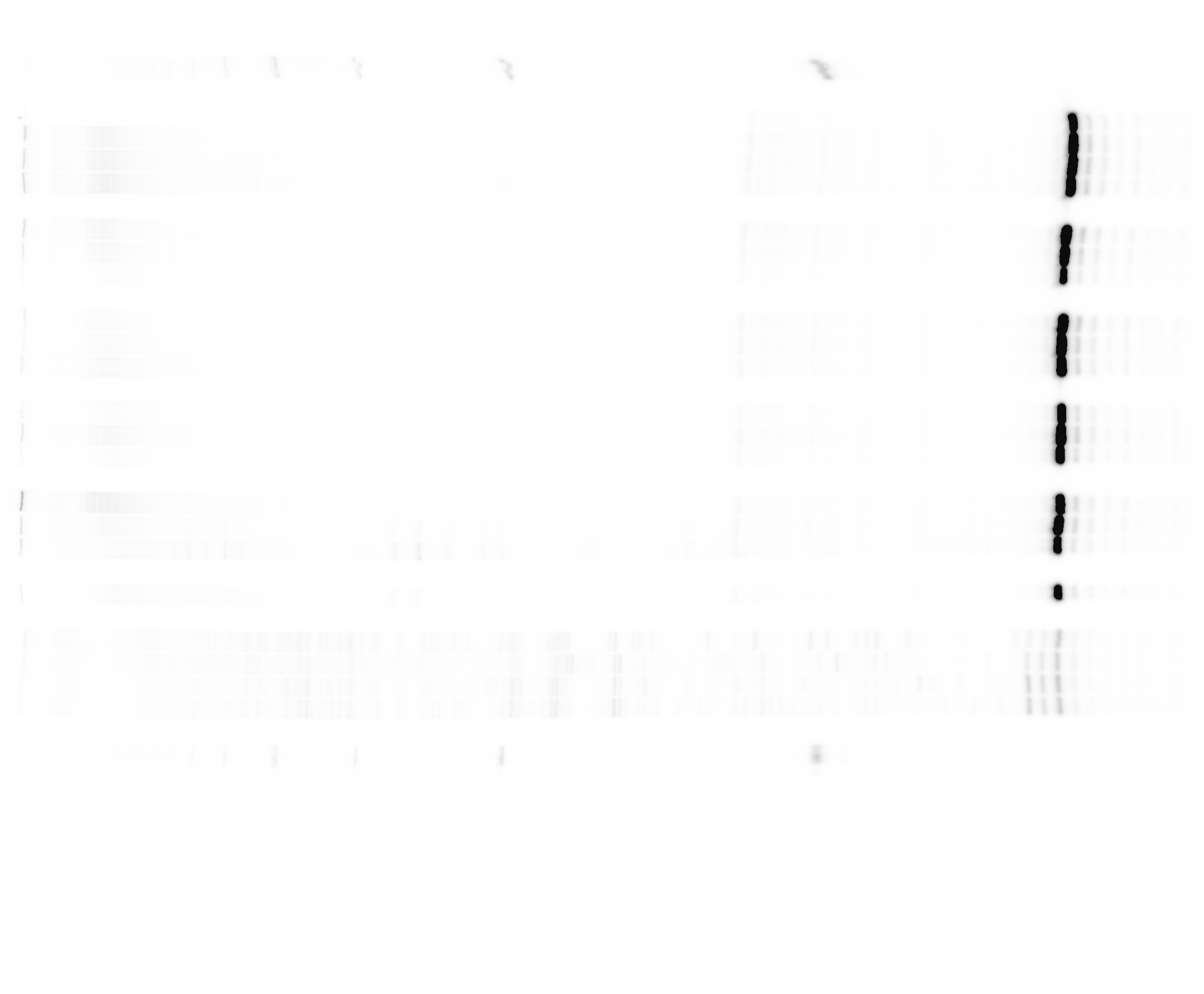

Supplement: Figure 7—source data 1. [file elife-69916-fig7-data1.zip › Figure 7_Sourcedata_activity of Endonuclease G/Figure 7C_primer extension_Endonuclease screening/Figure 7C_primer extension_purified_Endonuclease screening.tif]

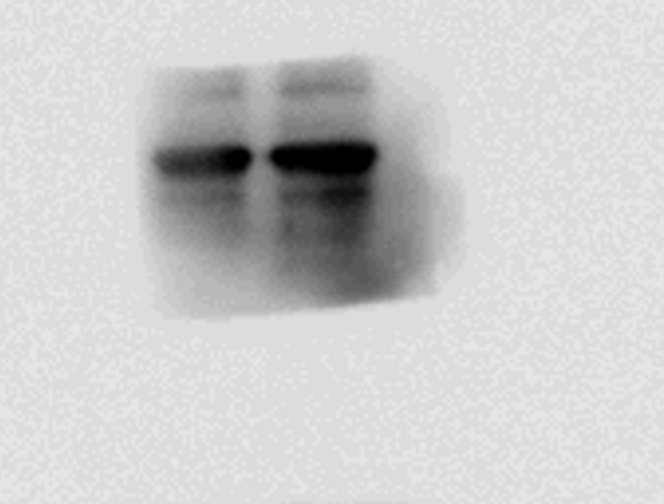

Supplement: Figure 7—source data 1. [file elife-69916-fig7-data1.zip › Figure 7_Sourcedata_activity of Endonuclease G/Figure 7F_western_Immunodepletion of EndoG/Figure 7F_Gel image profile for Actin_Immunodepletion of EndoG extracts.tif]

## Slide 1
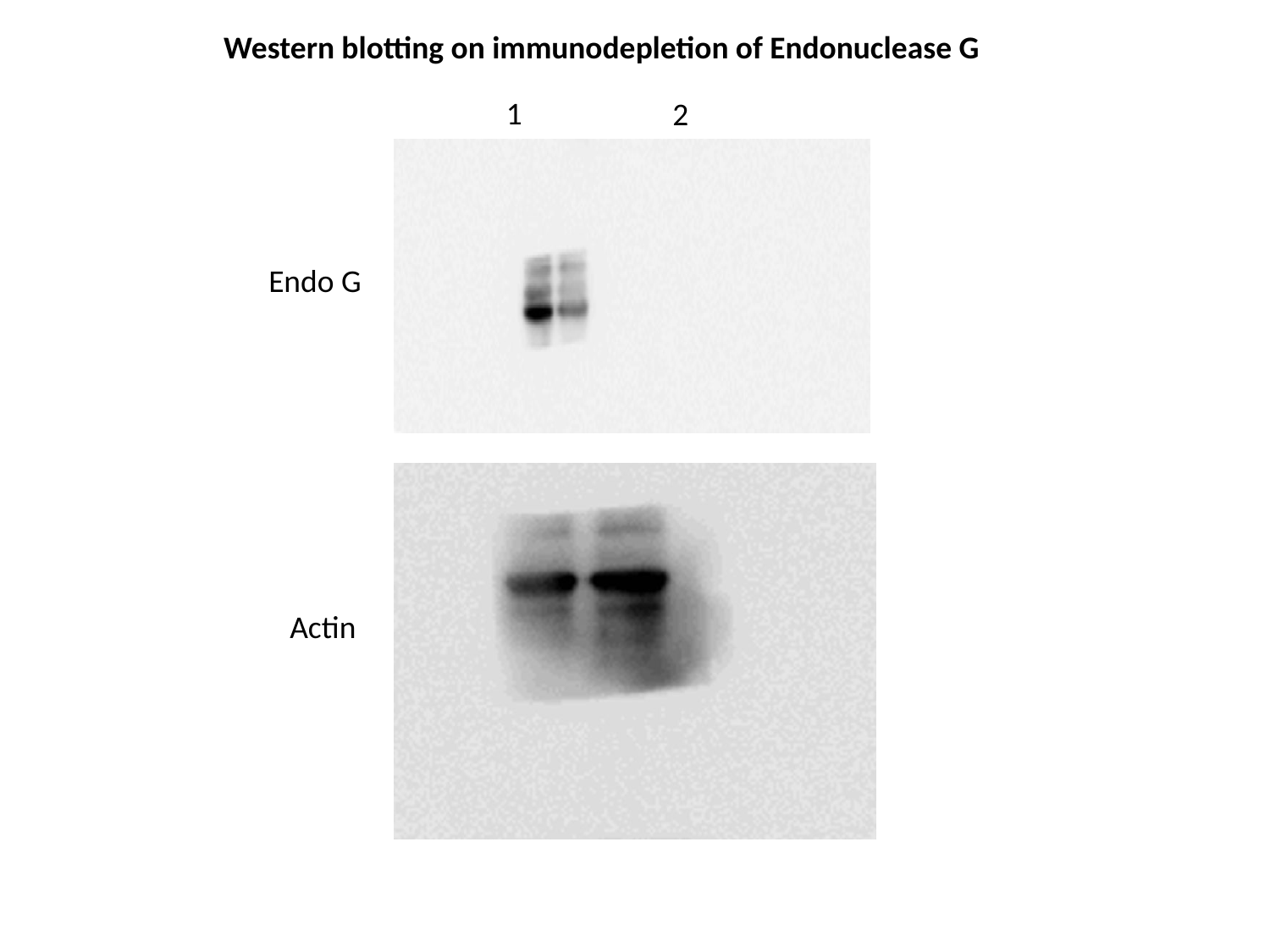

Western blotting on immunodepletion of Endonuclease G
1
2
Endo G
Actin

Supplement: Figure 7—source data 1. [file elife-69916-fig7-data1.zip › Figure 7_Sourcedata_activity of Endonuclease G/Figure 7F_western_Immunodepletion of EndoG/Figure 7F_Labeled gel.pptx]

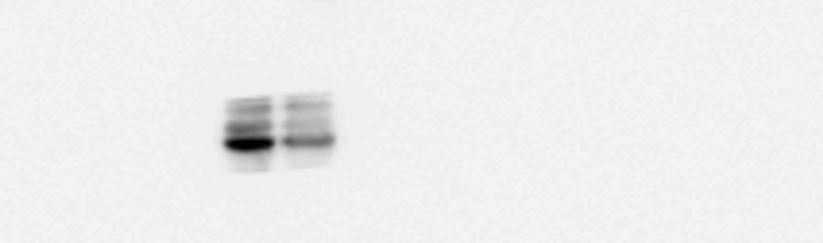

Supplement: Figure 7—source data 1. [file elife-69916-fig7-data1.zip › Figure 7_Sourcedata_activity of Endonuclease G/Figure 7F_western_Immunodepletion of EndoG/Figure 7F_Gel image profile for EndoG_Immunodepletion of EndoG extracts.tif]

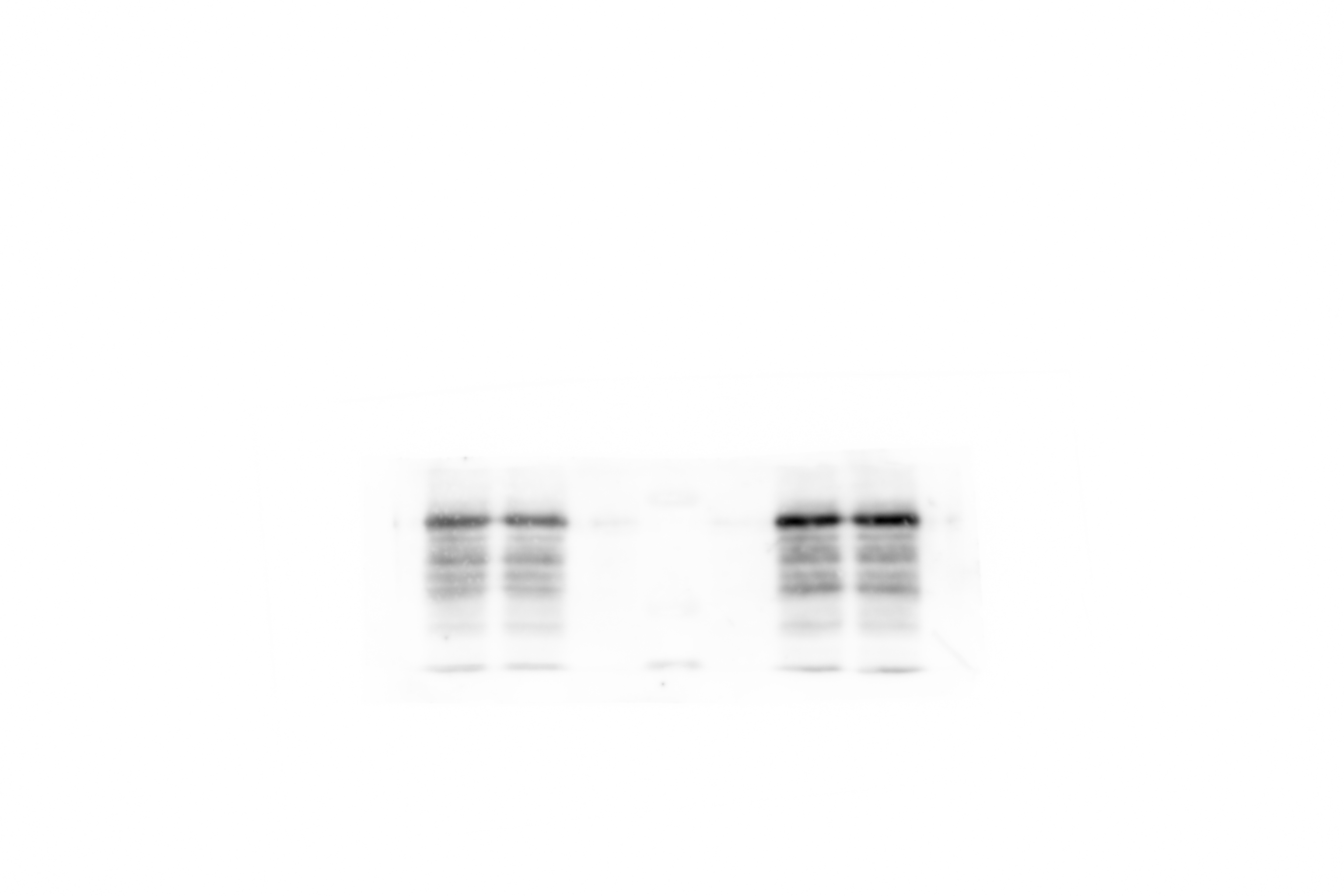

Supplement: Figure 7—source data 2. [file elife-69916-fig7-data2.zip › Figure 7_Source data_Supplementary/Figure S7C_Western blot for confirmation of EndoG and its mutant.tif]

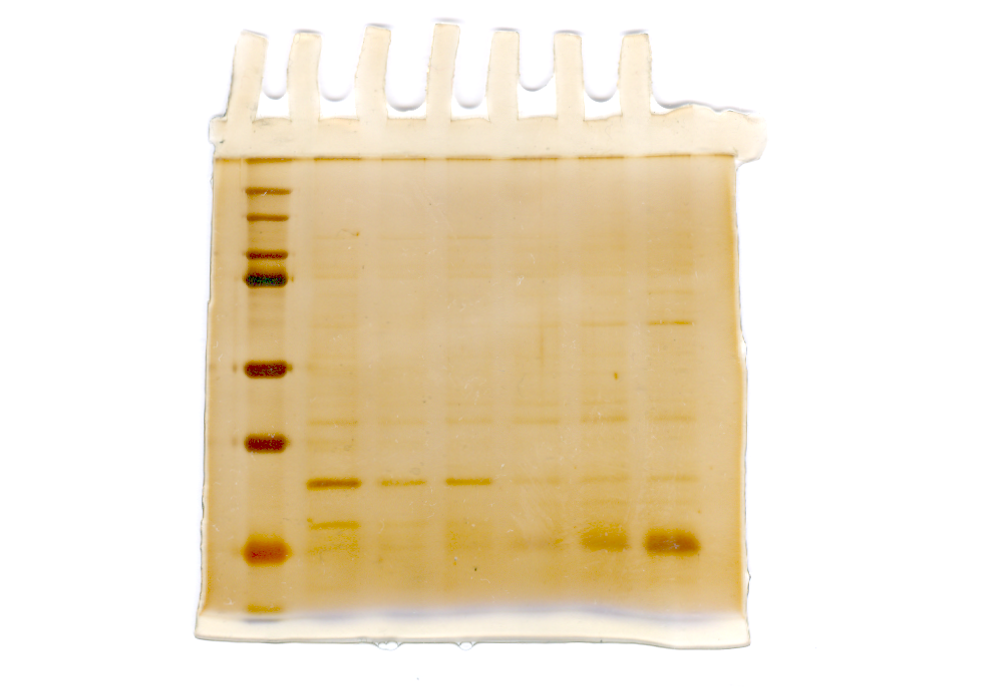

Supplement: Figure 7—source data 2. [file elife-69916-fig7-data2.zip › Figure 7_Source data_Supplementary/Figure S7B_Purification profile for Endog mutant.tif]

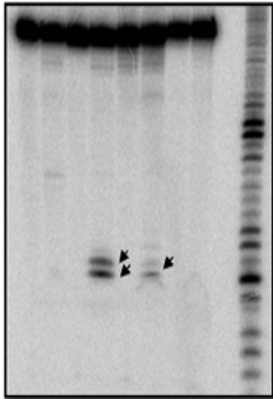

Supplement: Figure 7—source data 2. [file elife-69916-fig7-data2.zip › Figure 7_Source data_Supplementary/FIgure S7I_Activity assay after purification of RAGs/Figure S3I_Gel profile_Activity assay after Purification of RAG.tif]

## Slide 1
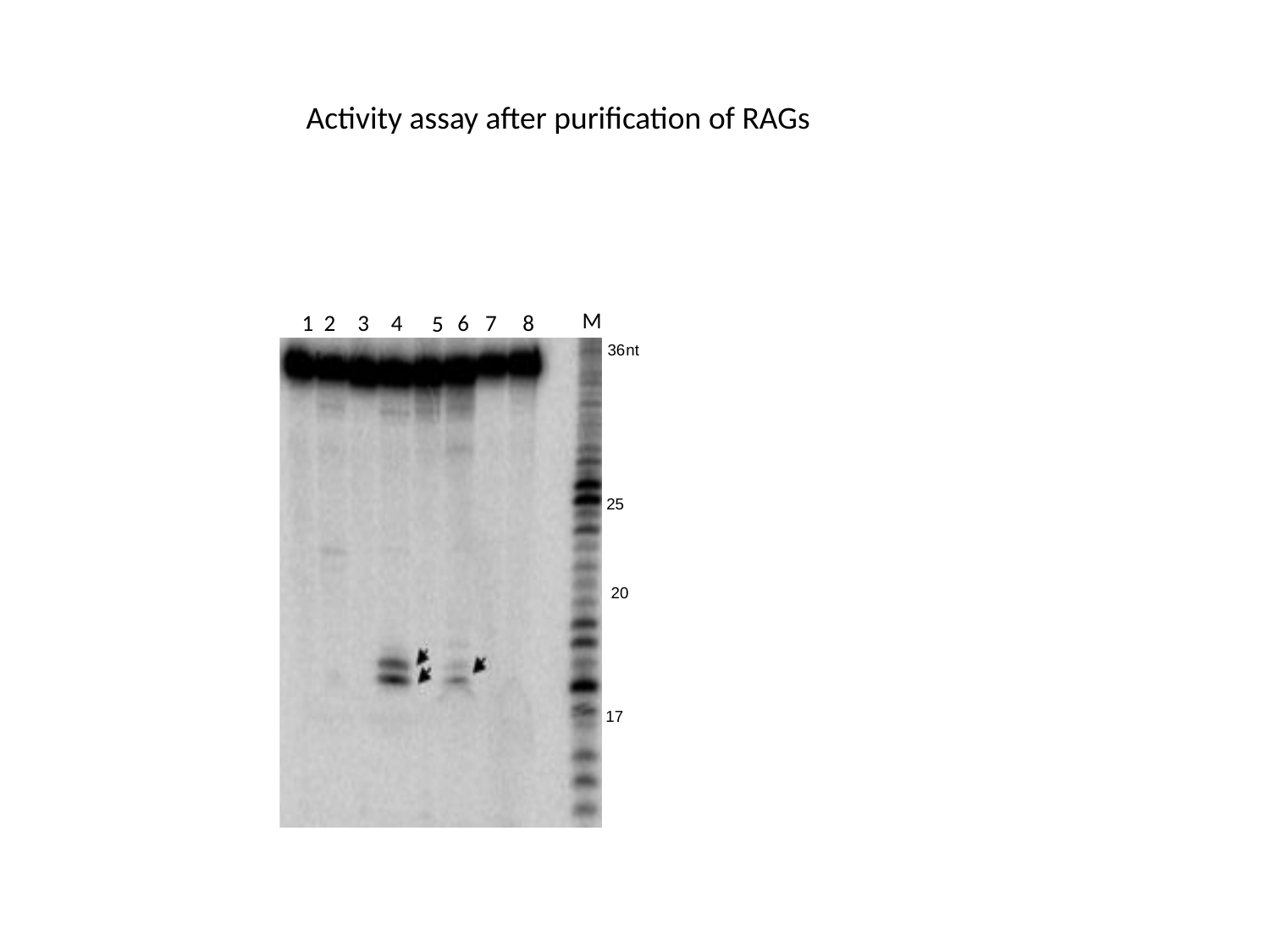

Activity assay after purification of RAGs
M
1
2
3
4
6
7
8
5
36
nt
25
20
17

Supplement: Figure 7—source data 2. [file elife-69916-fig7-data2.zip › Figure 7_Source data_Supplementary/FIgure S7I_Activity assay after purification of RAGs/Figure S3I_Activity assay after purification of RAGs.pptx]

## Slide 1
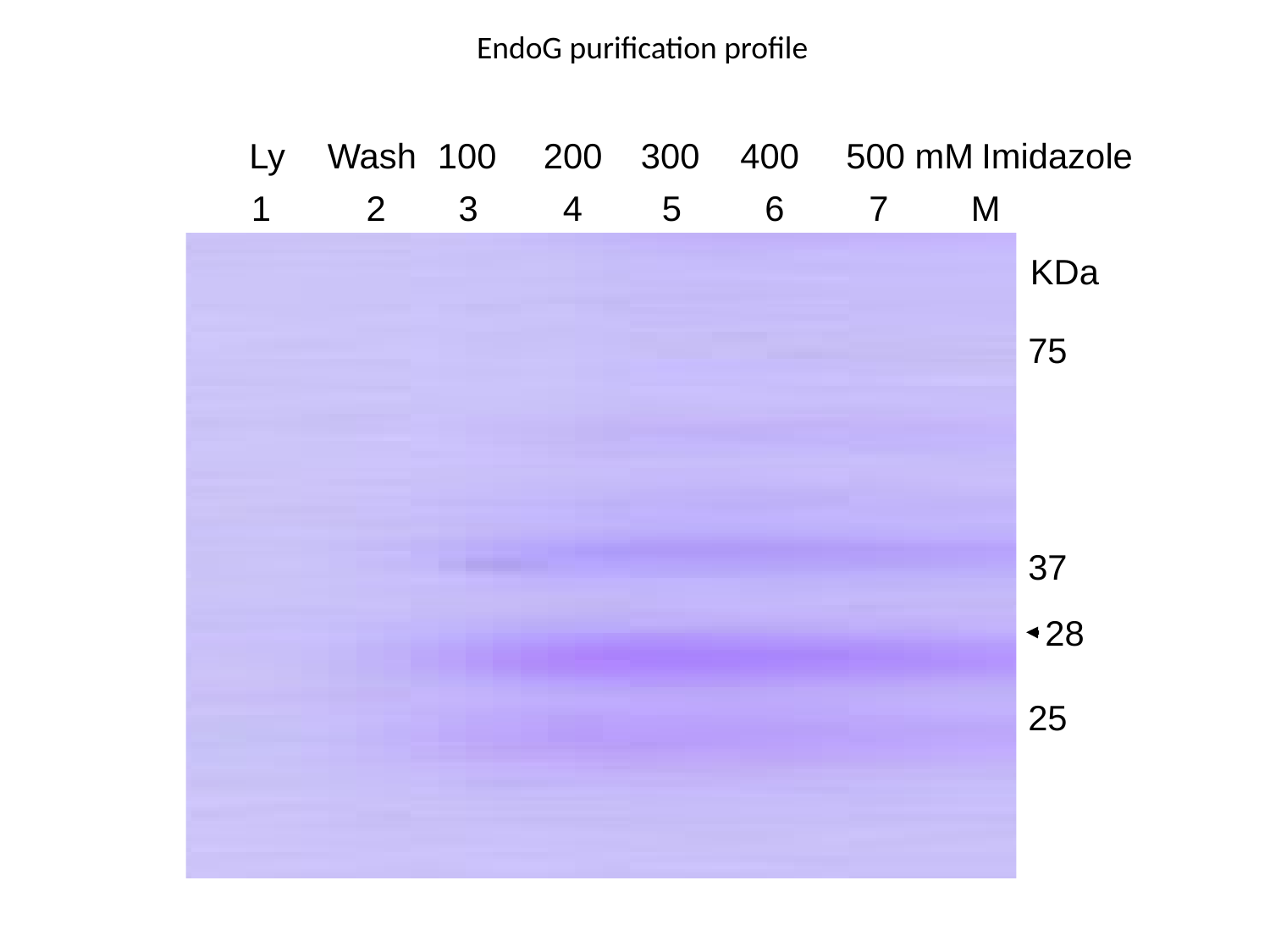

Ly
Wash
100
200
300
400
500 mM
Imidazole
EndoG purification profile
2
3
4
1
5
6
7
M
KDa
75
37
28
25

Supplement: Figure 7—source data 2. [file elife-69916-fig7-data2.zip › Figure 7_Source data_Supplementary/Figure S7A_Purification of EndoG/Figure S3A_Purification of EndoG.pptx]

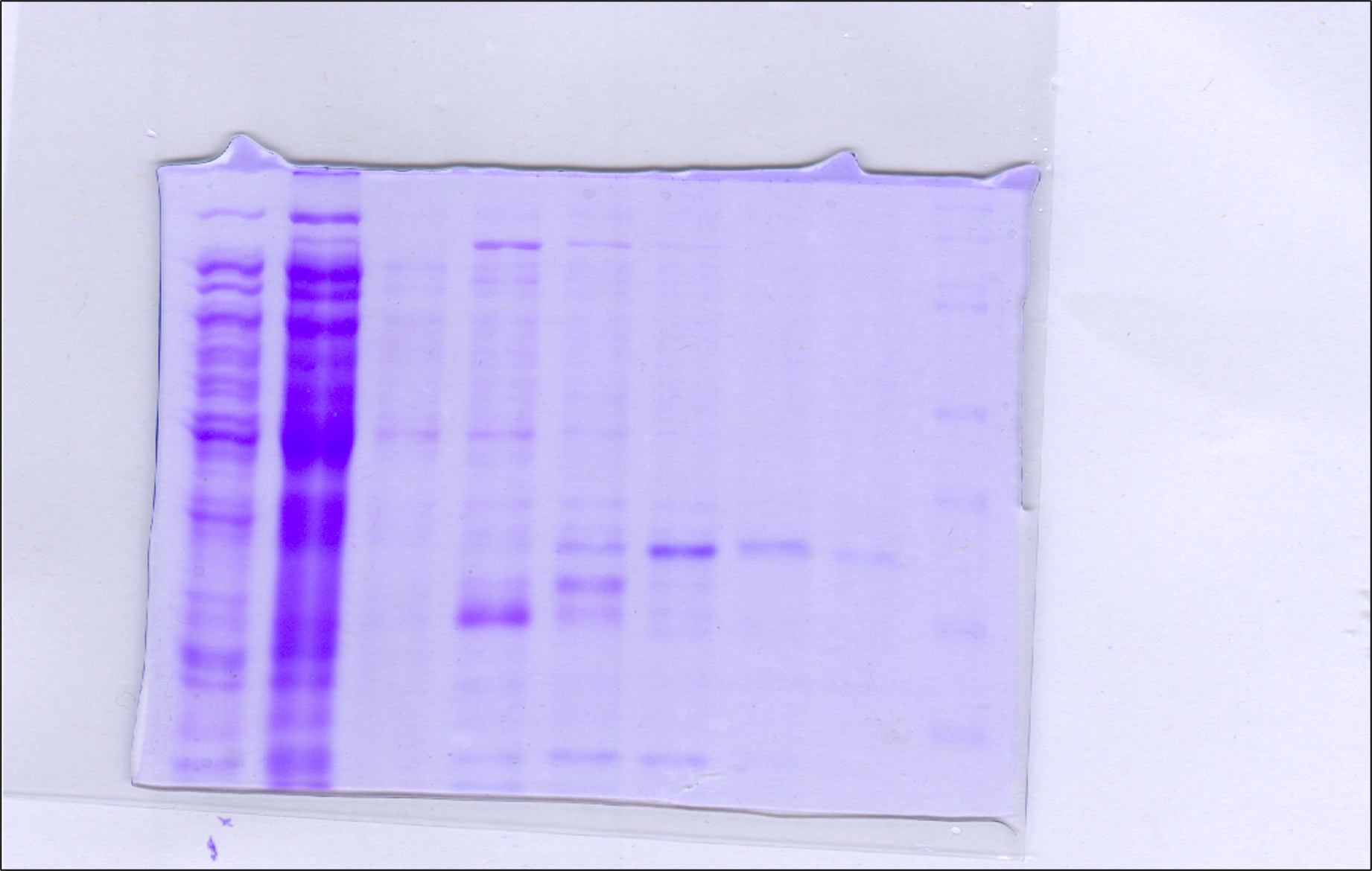

Supplement: Figure 7—source data 2. [file elife-69916-fig7-data2.zip › Figure 7_Source data_Supplementary/Figure S7A_Purification of EndoG/Figure S3A_Purification of EndoG.tif]

## Slide 1
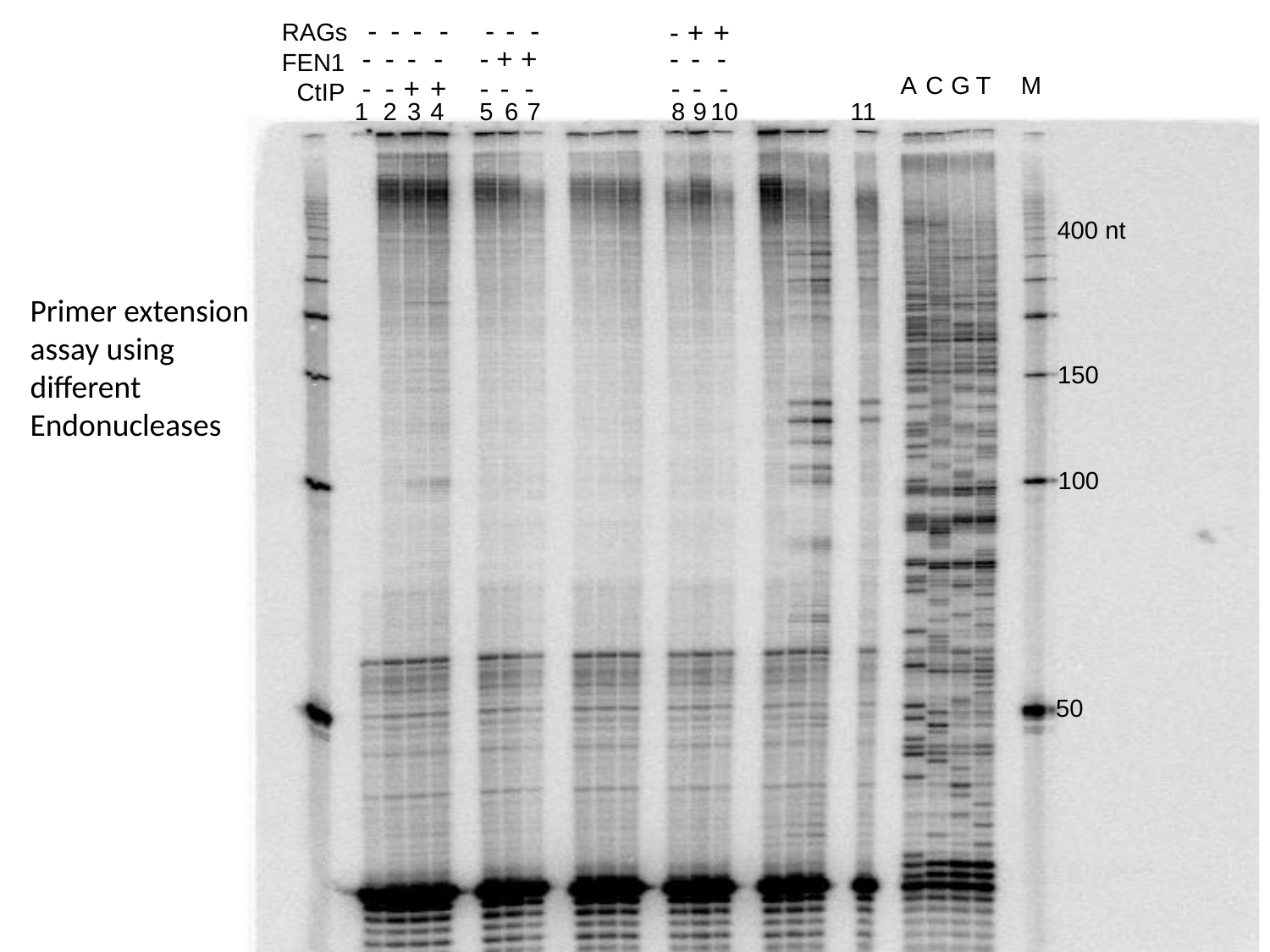

RAGs
-
-
-
-
-
-
-
-
+
+
FEN1
-
-
-
-
-
+
+
-
-
-
CtIP
-
-
+
+
-
-
-
-
-
-
A
C
G
T
M
1
2
3
4
5
6
7
8
9
10
11
400 nt
Primer extension assay using different Endonucleases
150
100
50

Supplement: Figure 7—source data 2. [file elife-69916-fig7-data2.zip › Figure 7_Source data_Supplementary/Figure S7J_Primer extension_purified Endonucleases screening/Figure S3J_primer extension_purified_Endonuclease screening.pptx]

## Slide 1
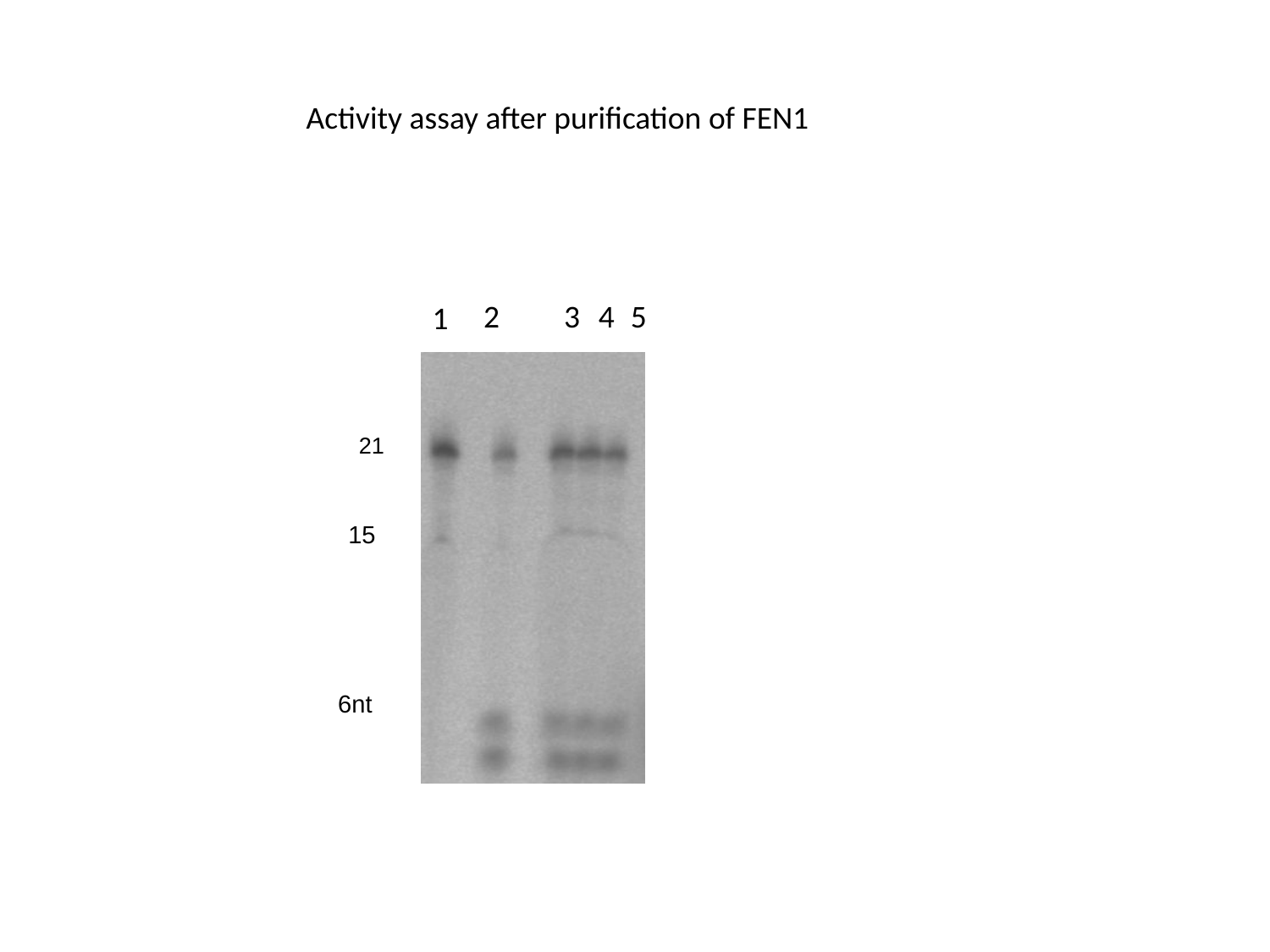

Activity assay after purification of FEN1
2
3
4
5
1
21
15
6nt

Supplement: Figure 7—source data 2. [file elife-69916-fig7-data2.zip › Figure 7_Source data_Supplementary/Figure S7G_Activity assay after purification of FEN1/Figure S3G_Activity assay after purification of FEN1.pptx]

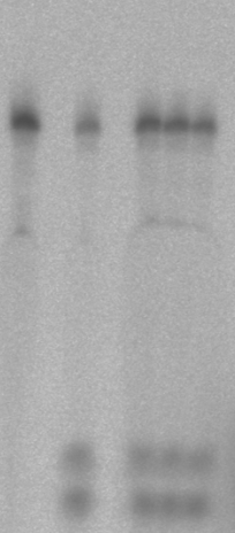

Supplement: Figure 7—source data 2. [file elife-69916-fig7-data2.zip › Figure 7_Source data_Supplementary/Figure S7G_Activity assay after purification of FEN1/Figure S3G_Gel profile_Activity assay after Purification of FEN1.tif]

## Slide 1
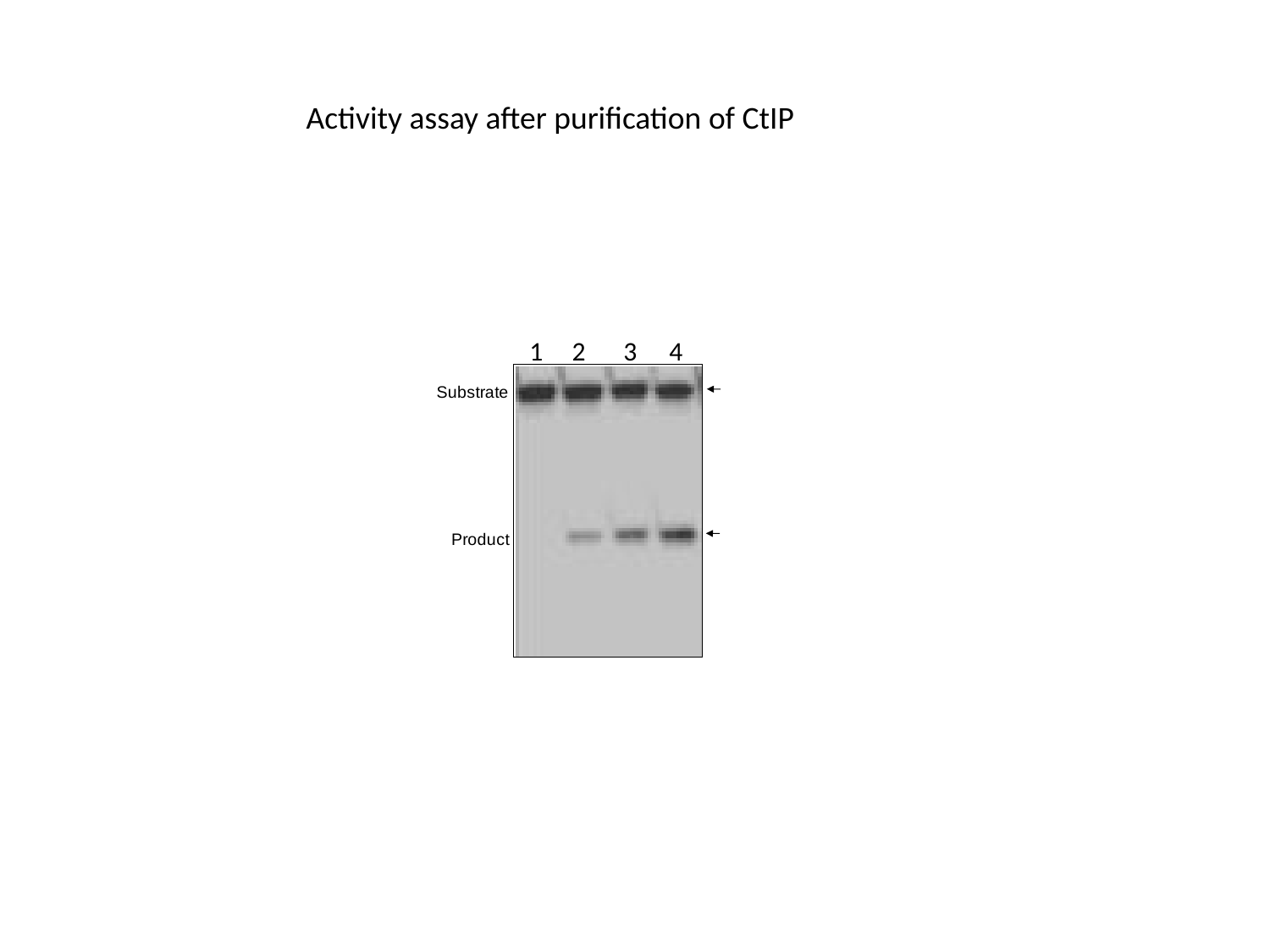

Activity assay after purification of CtIP

Supplement: Figure 7—source data 2. [file elife-69916-fig7-data2.zip › Figure 7_Source data_Supplementary/Figure S7E_Gel profile_Activity assay after purification of CtIP/Figure S3E_Activity assay after purification of CtIP.pptx]

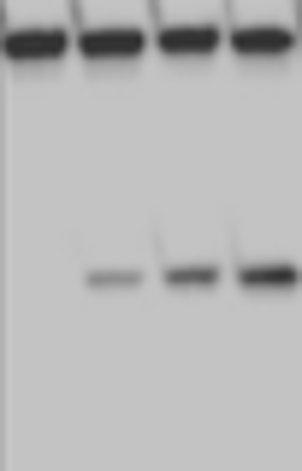

Supplement: Figure 7—source data 2. [file elife-69916-fig7-data2.zip › Figure 7_Source data_Supplementary/Figure S7E_Gel profile_Activity assay after purification of CtIP/Figure S3E_Gel profile_Activity assay after Purification of CtIP.tif]

## Slide 1
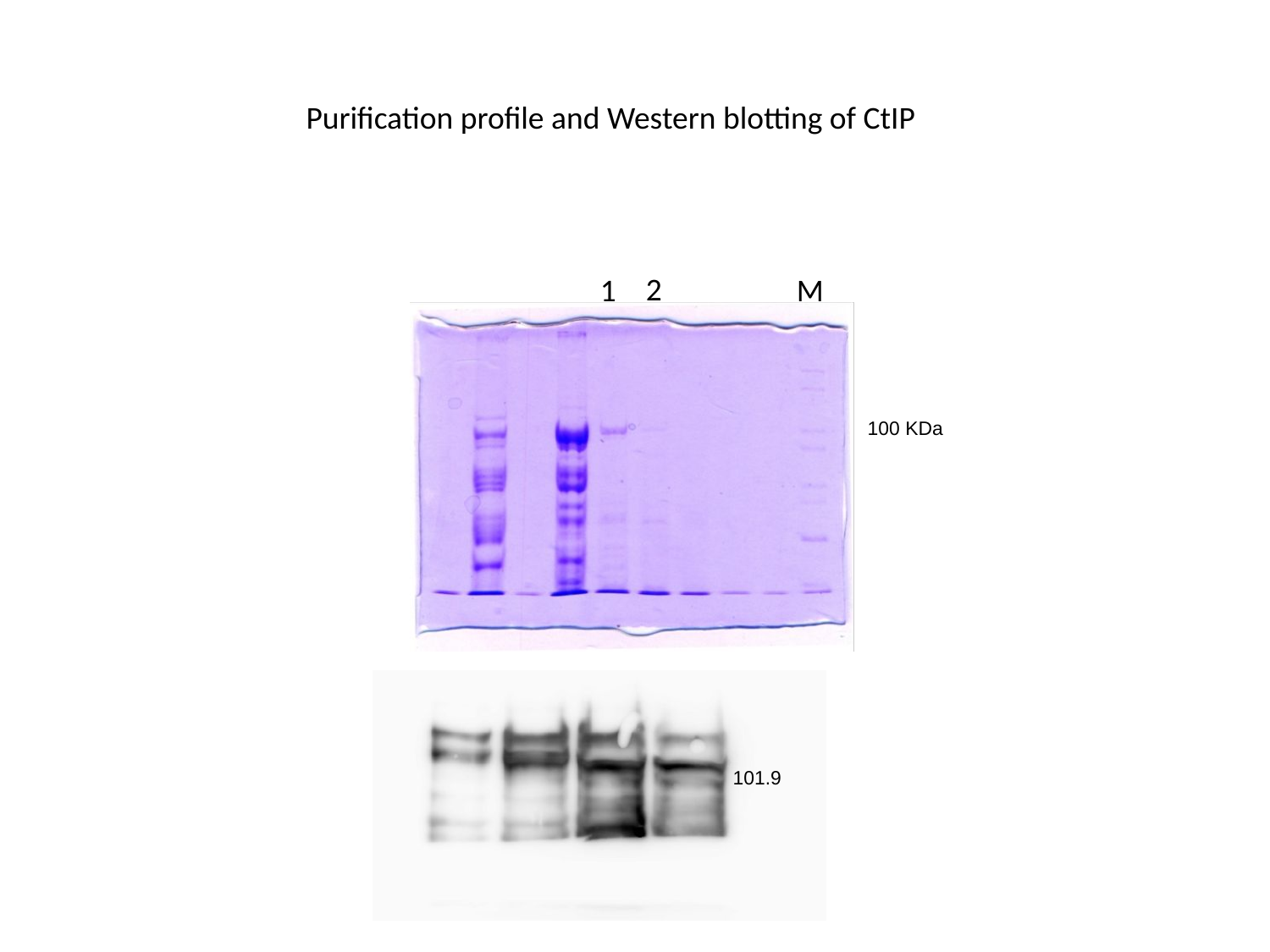

Purification profile and Western blotting of CtIP
2
1
M
100 KDa
101.9

Supplement: Figure 7—source data 2. [file elife-69916-fig7-data2.zip › Figure 7_Source data_Supplementary/Figure S7D_Purification of CtIP/Figure S3D_Purification and Western blotting for purification of CtIP.pptx]

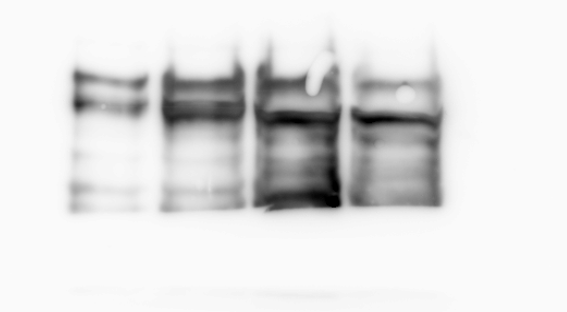

Supplement: Figure 7—source data 2. [file elife-69916-fig7-data2.zip › Figure 7_Source data_Supplementary/Figure S7D_Purification of CtIP/Figure S3D_Gel profile_Western blotting after Purification of CtIP.tif]

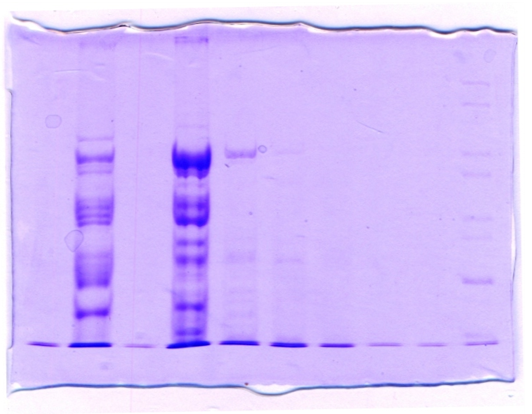

Supplement: Figure 7—source data 2. [file elife-69916-fig7-data2.zip › Figure 7_Source data_Supplementary/Figure S7D_Purification of CtIP/Figure S3D_Gel profile_Purification of CtIP.tif]

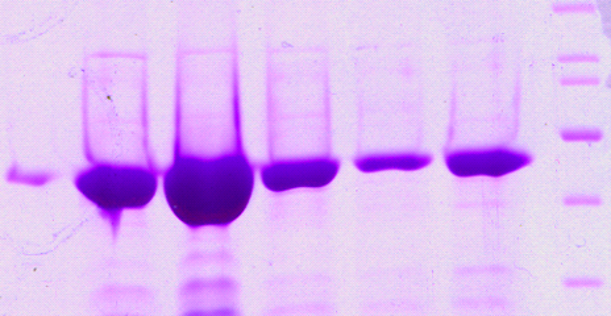

Supplement: Figure 7—source data 2. [file elife-69916-fig7-data2.zip › Figure 7_Source data_Supplementary/Figure S7F_Purification of FEN1/Figure S3F_Gel profile_Purification FEN1.tif]

## Slide 1
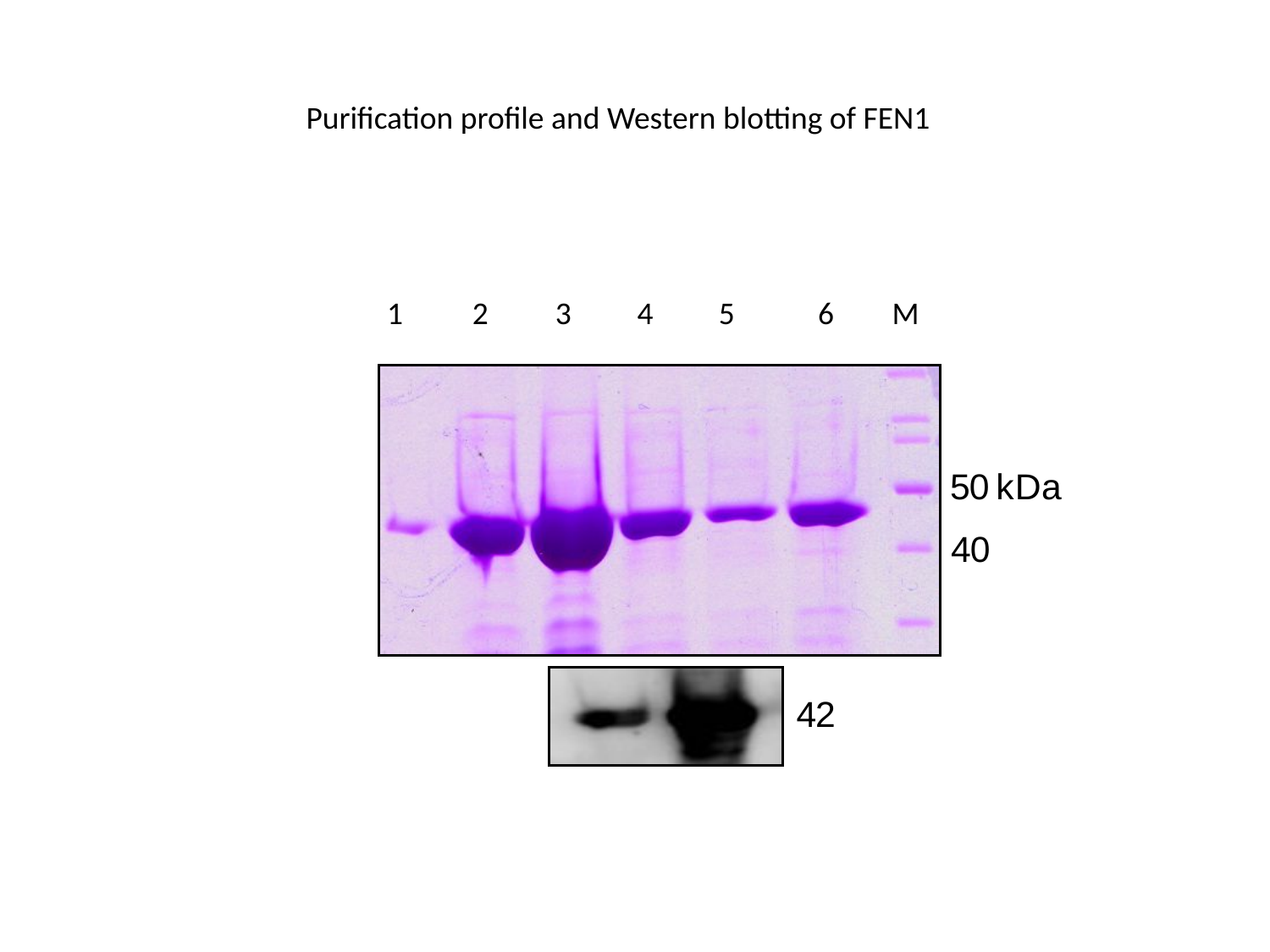

Purification profile and Western blotting of FEN1
1
2
3
4
5
6
M

Supplement: Figure 7—source data 2. [file elife-69916-fig7-data2.zip › Figure 7_Source data_Supplementary/Figure S7F_Purification of FEN1/Figure S3F_Purification and Western blotting for purification of FEN1.pptx]

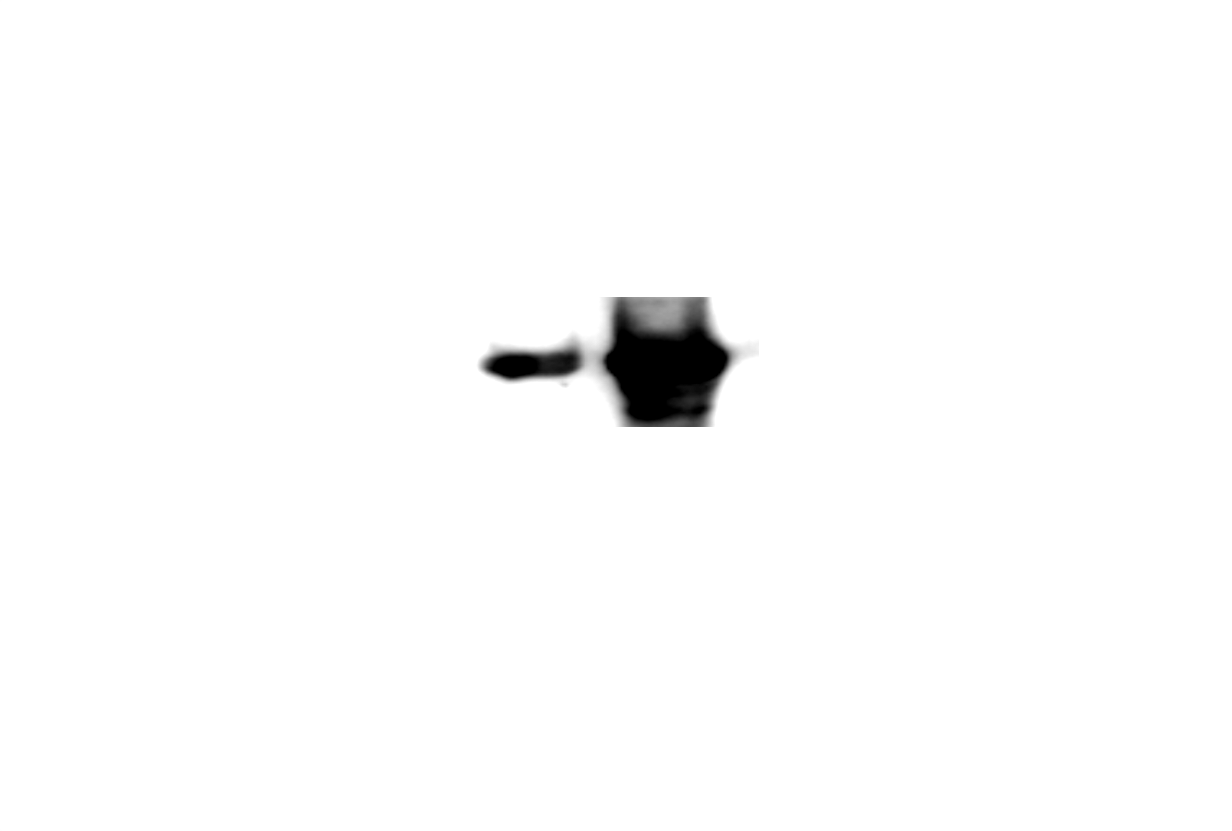

Supplement: Figure 7—source data 2. [file elife-69916-fig7-data2.zip › Figure 7_Source data_Supplementary/Figure S7F_Purification of FEN1/Figure S3F_Gel profile_Western blotting after Purification of FEN1.tif]

## Slide 1
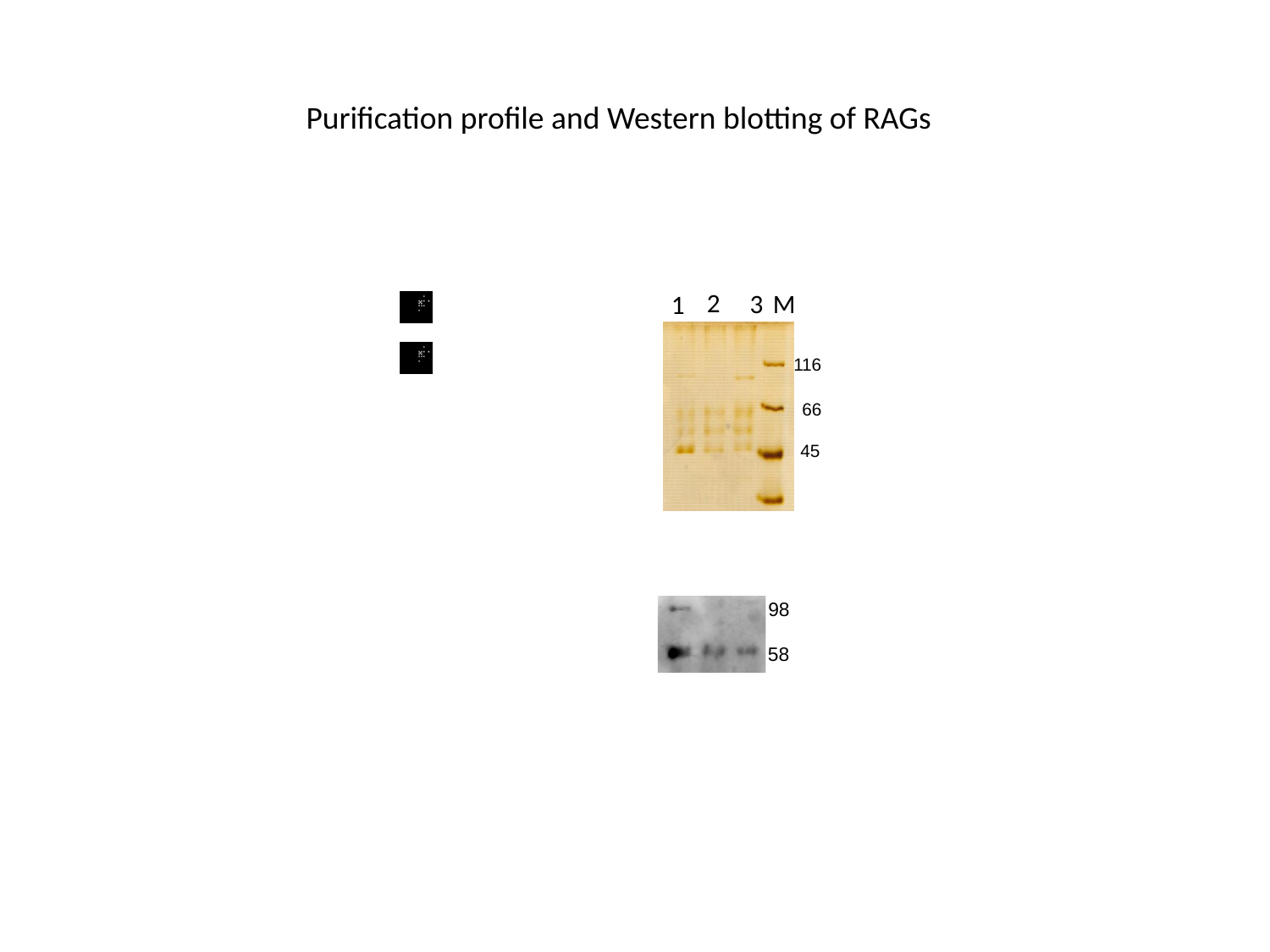

Purification profile and Western blotting of RAGs
2
3
M
1
116
66
45
98
58

Supplement: Figure 7—source data 2. [file elife-69916-fig7-data2.zip › Figure 7_Source data_Supplementary/Figure S7H_Purification of RAG/Figure S3H_Purification and Western blotting for purification of RAG.pptx]

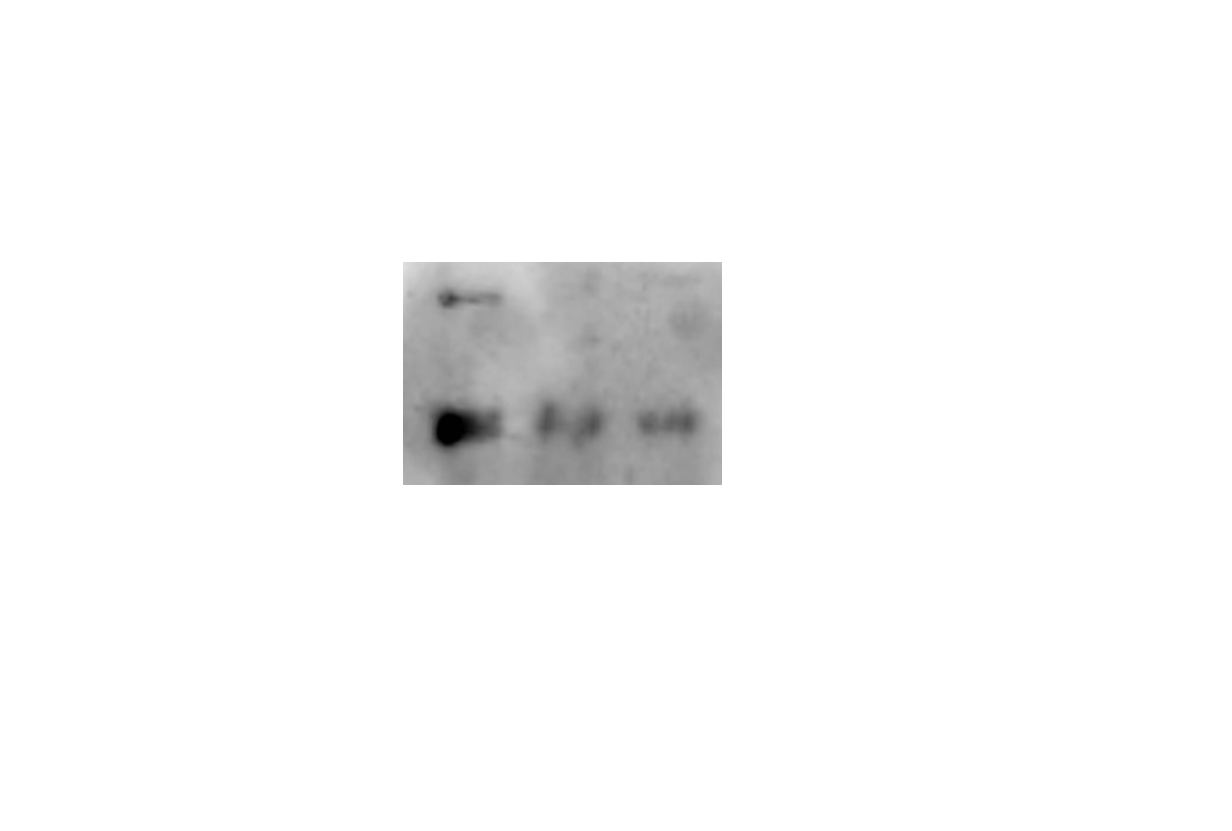

Supplement: Figure 7—source data 2. [file elife-69916-fig7-data2.zip › Figure 7_Source data_Supplementary/Figure S7H_Purification of RAG/Figure S3H_Western blotting after Purification of RAG.tif]

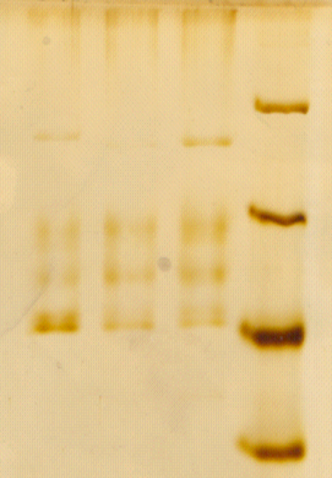

Supplement: Figure 7—source data 2. [file elife-69916-fig7-data2.zip › Figure 7_Source data_Supplementary/Figure S7H_Purification of RAG/Figure S3H_Gel profile_Purification of RAG.tif]

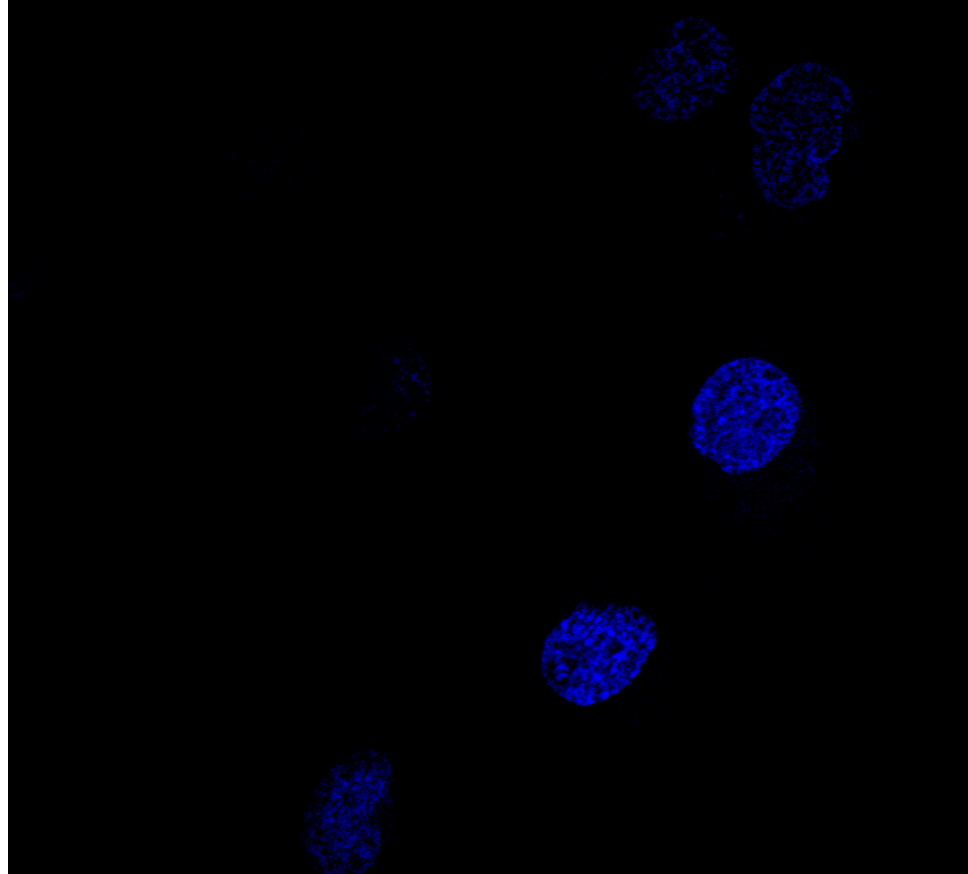

Supplement: Figure 8—source data 1. [file elife-69916-fig8-data1.zip › Figure8_Sourcedata_localization of Endonuclease G/Figure 8C_Representative image_Co IF_TFAM and EndoG/Figure 3H_Representative image_Co IF_TFAM and EndoG_Sec control_DAPI.tif]

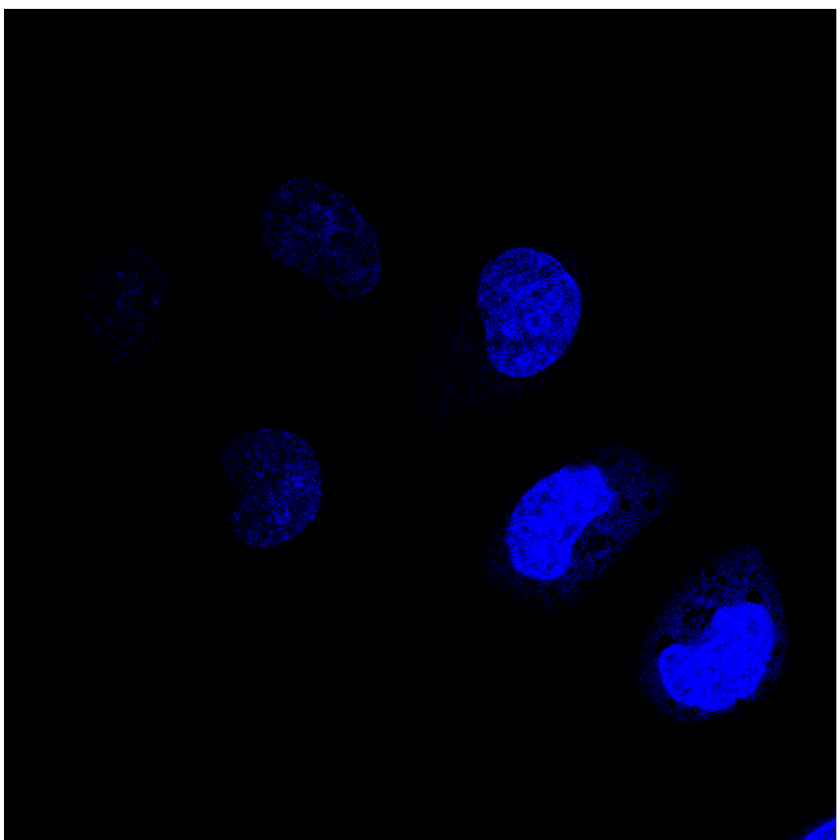

Supplement: Figure 8—source data 1. [file elife-69916-fig8-data1.zip › Figure8_Sourcedata_localization of Endonuclease G/Figure 8C_Representative image_Co IF_TFAM and EndoG/Figure 3H_Representative image_Co IF_TFAM and EndoG exp_DAPI.tif]

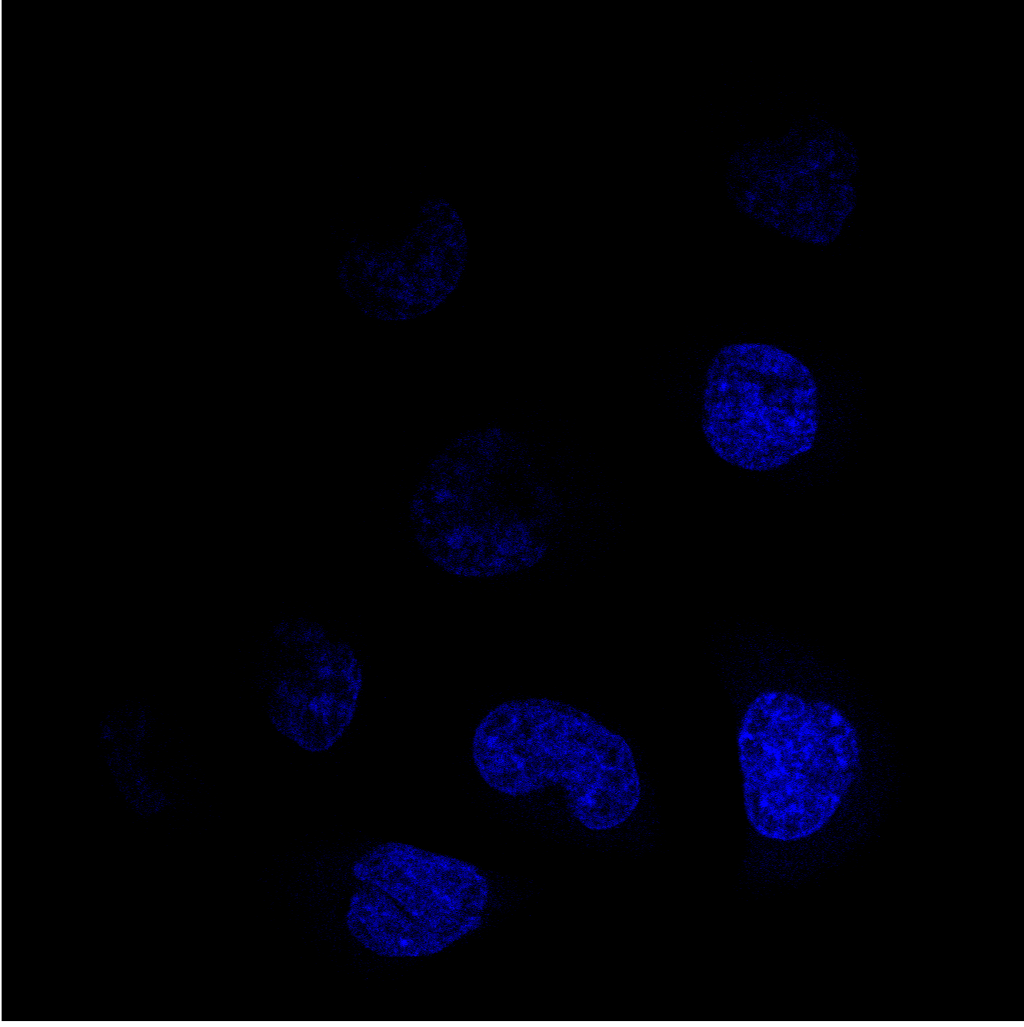

Supplement: Figure 8—source data 1. [file elife-69916-fig8-data1.zip › Figure8_Sourcedata_localization of Endonuclease G/Figure 8C_Representative image_Co IF_TFAM and EndoG/Figure 3H_Representative image_Co IF_TFAM and EndoG_Sec control2_DAPI.tif]

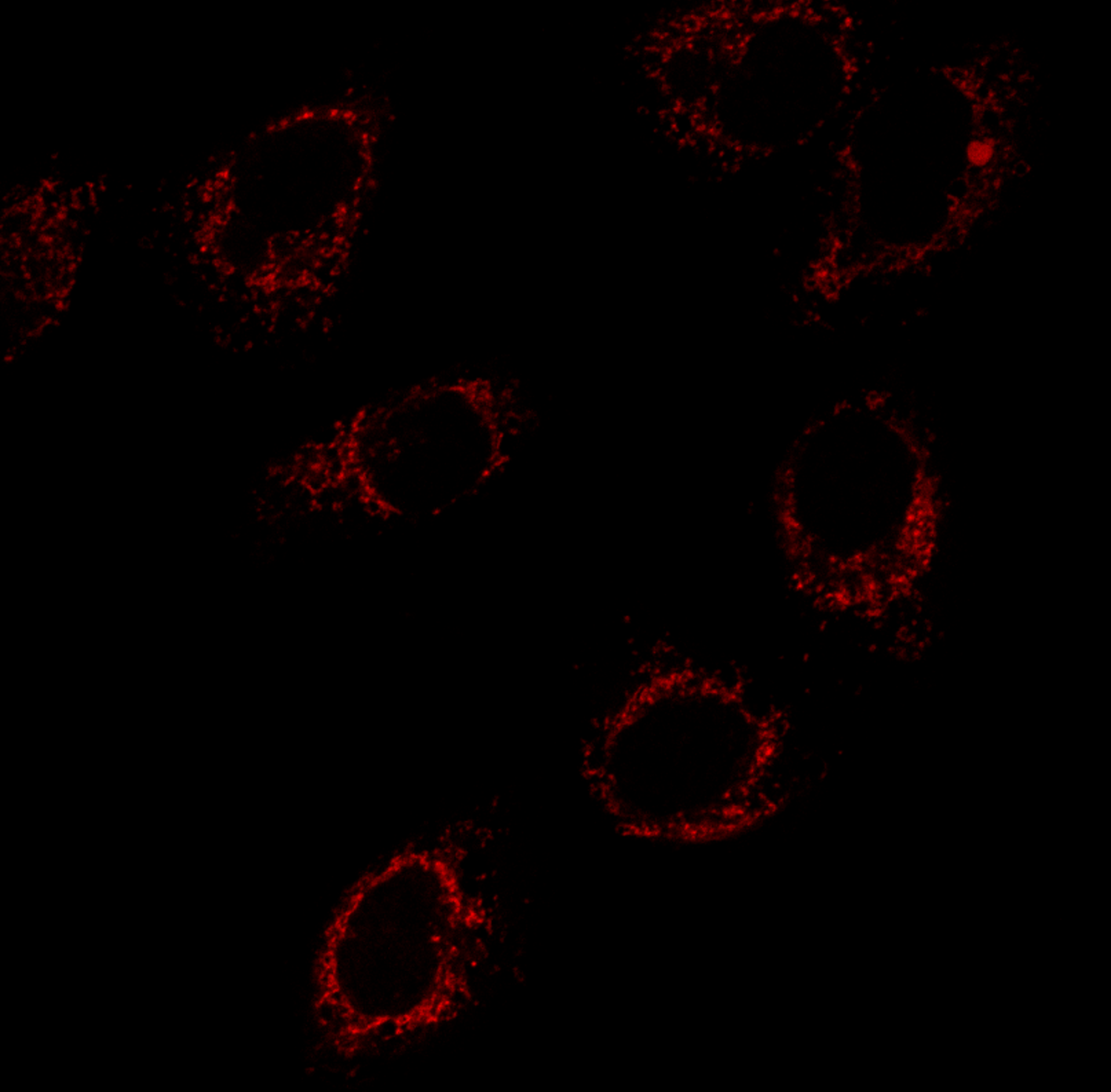

Supplement: Figure 8—source data 1. [file elife-69916-fig8-data1.zip › Figure8_Sourcedata_localization of Endonuclease G/Figure 8C_Representative image_Co IF_TFAM and EndoG/Figure 3H_Representative image_Co IF_TFAM and EndoG_Sec control_TFAM.tif]

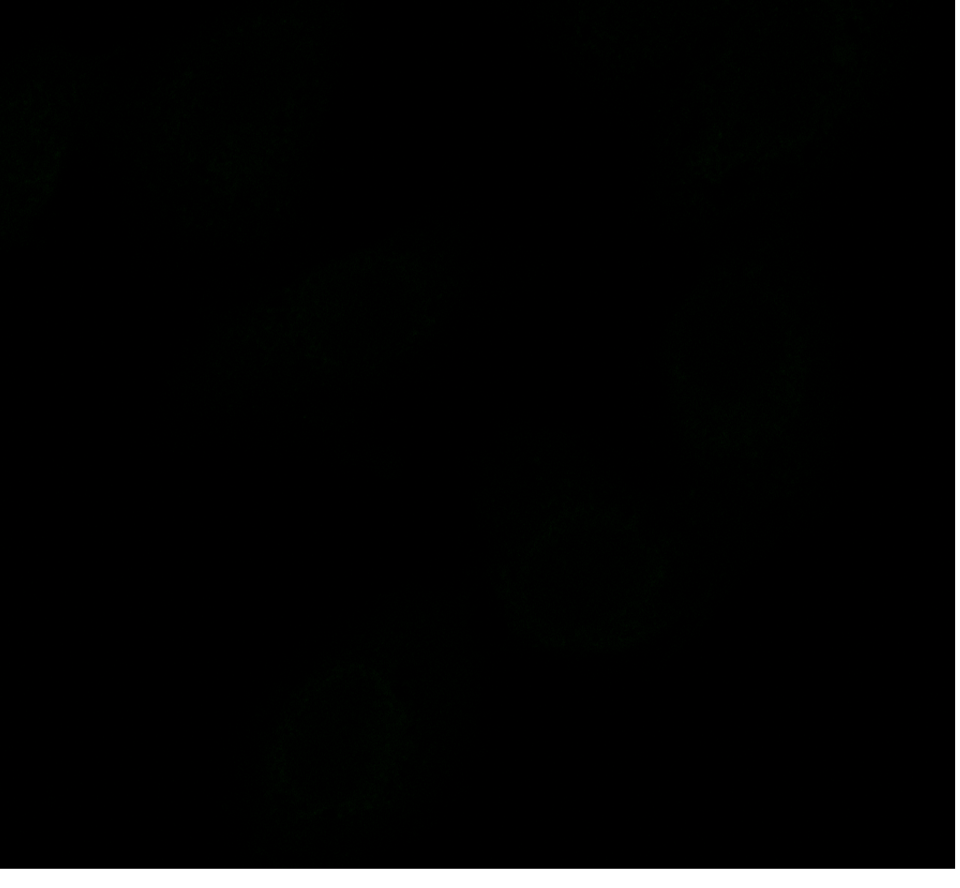

Supplement: Figure 8—source data 1. [file elife-69916-fig8-data1.zip › Figure8_Sourcedata_localization of Endonuclease G/Figure 8C_Representative image_Co IF_TFAM and EndoG/Figure 3H_Representative image_Co IF_TFAM and EndoG_Sec control_Endo G.tif]

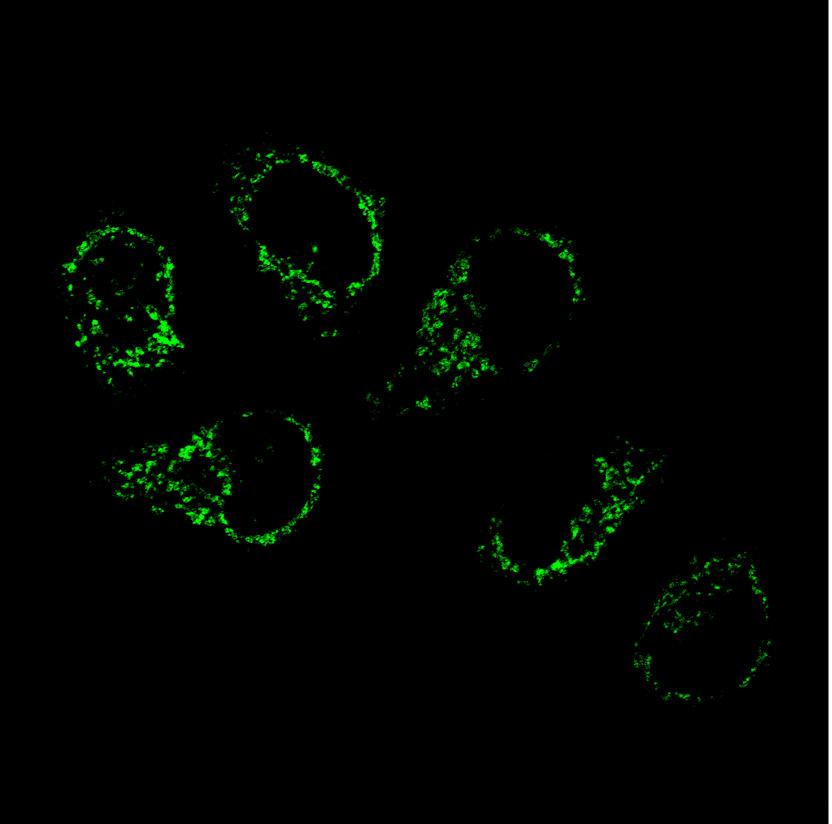

Supplement: Figure 8—source data 1. [file elife-69916-fig8-data1.zip › Figure8_Sourcedata_localization of Endonuclease G/Figure 8C_Representative image_Co IF_TFAM and EndoG/Figure 3H_Representative image_Co IF_TFAM and EndoG exp_EndoG.tif]

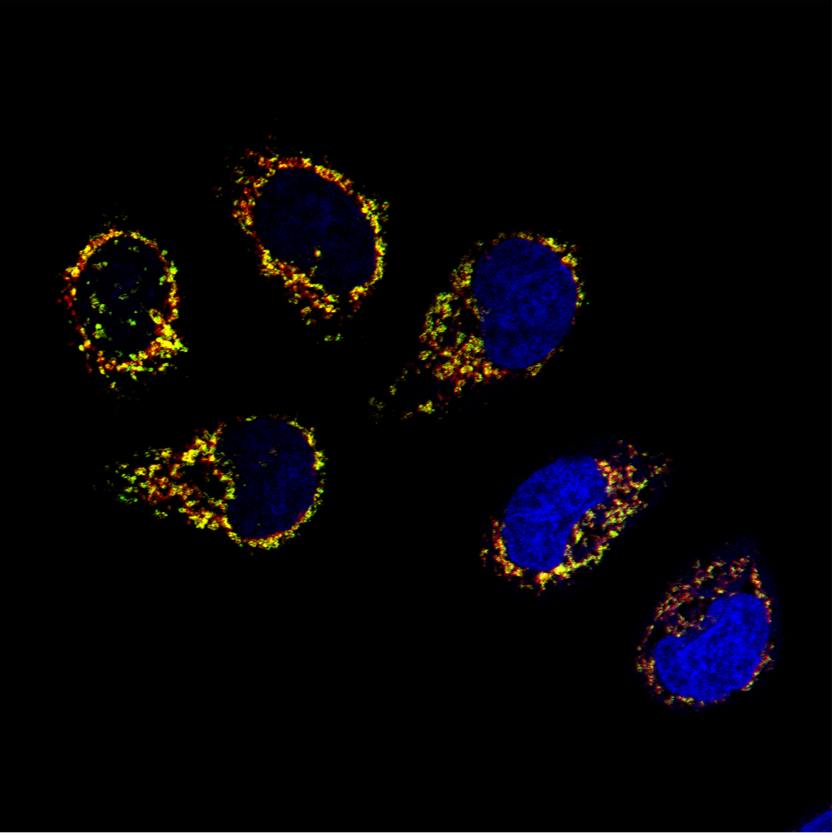

Supplement: Figure 8—source data 1. [file elife-69916-fig8-data1.zip › Figure8_Sourcedata_localization of Endonuclease G/Figure 8C_Representative image_Co IF_TFAM and EndoG/Figure 3H_Representative image_Co IF_TFAM and EndoG exp_merged.tif]

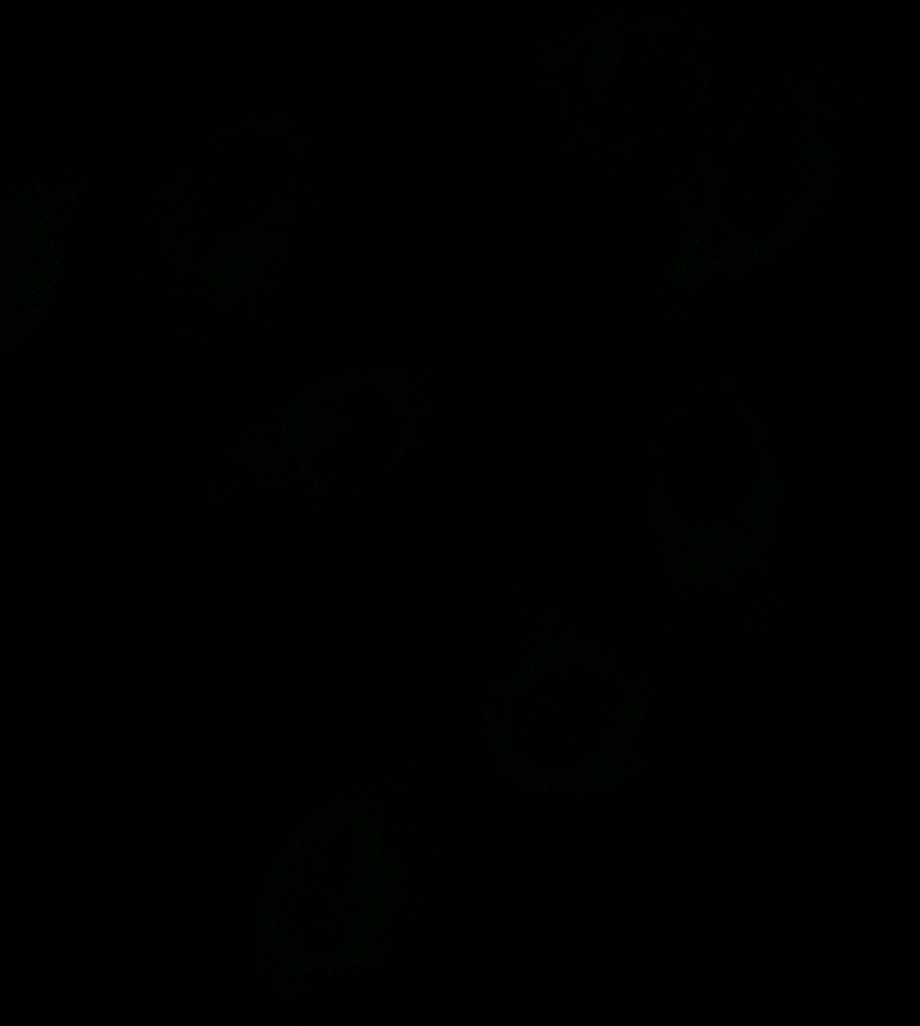

Supplement: Figure 8—source data 1. [file elife-69916-fig8-data1.zip › Figure8_Sourcedata_localization of Endonuclease G/Figure 8C_Representative image_Co IF_TFAM and EndoG/Figure 3H_Representative image_Co IF_TFAM and EndoG_Sec control2_TFAM.tif]

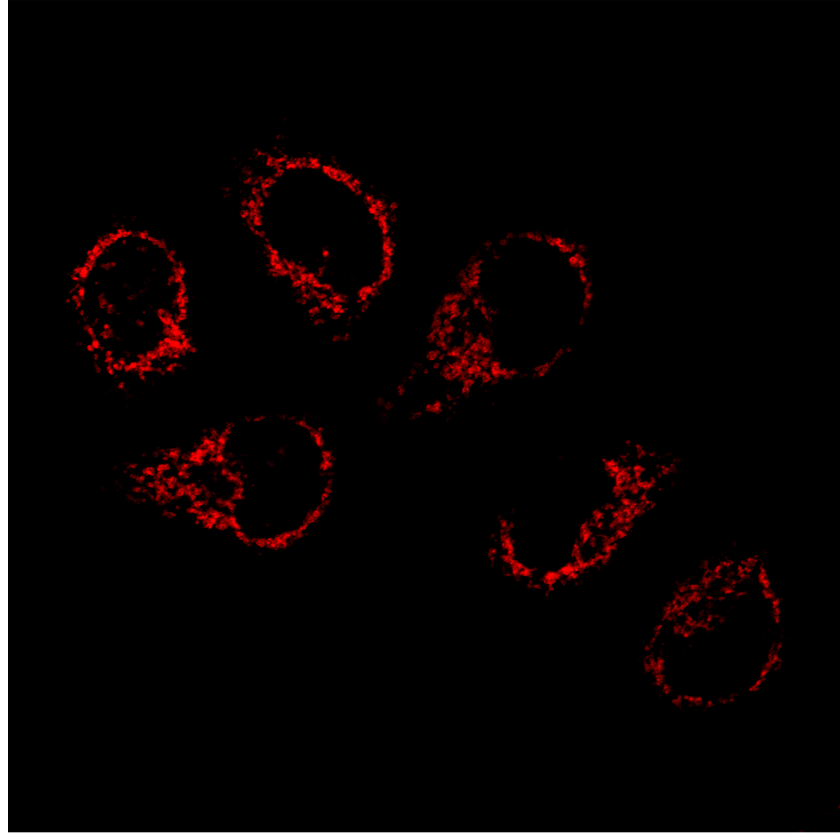

Supplement: Figure 8—source data 1. [file elife-69916-fig8-data1.zip › Figure8_Sourcedata_localization of Endonuclease G/Figure 8C_Representative image_Co IF_TFAM and EndoG/Figure 3H_Representative image_Co IF_TFAM and EndoG exp_TFAM.tif]

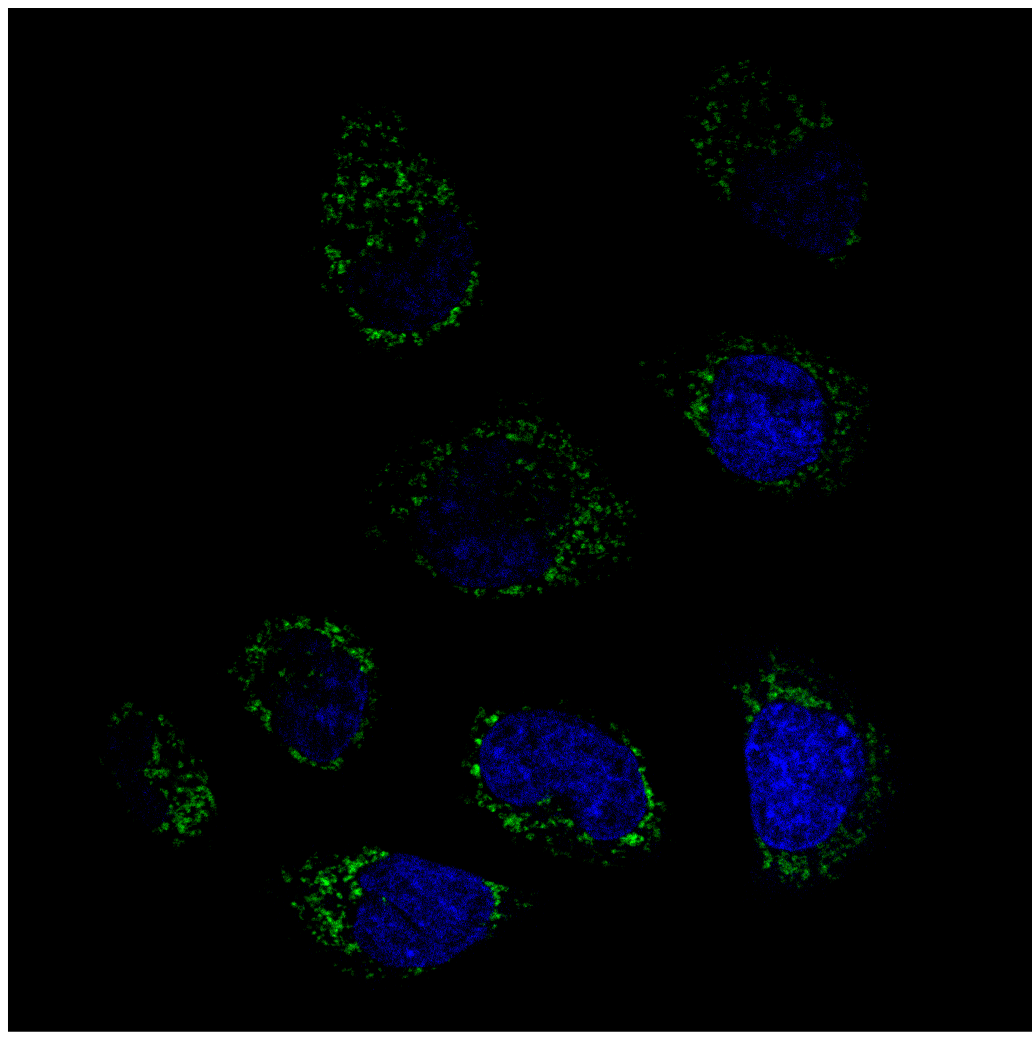

Supplement: Figure 8—source data 1. [file elife-69916-fig8-data1.zip › Figure8_Sourcedata_localization of Endonuclease G/Figure 8C_Representative image_Co IF_TFAM and EndoG/Figure 3H_Representative image_Co IF_TFAM and EndoG_Sec control2_merged.tif]

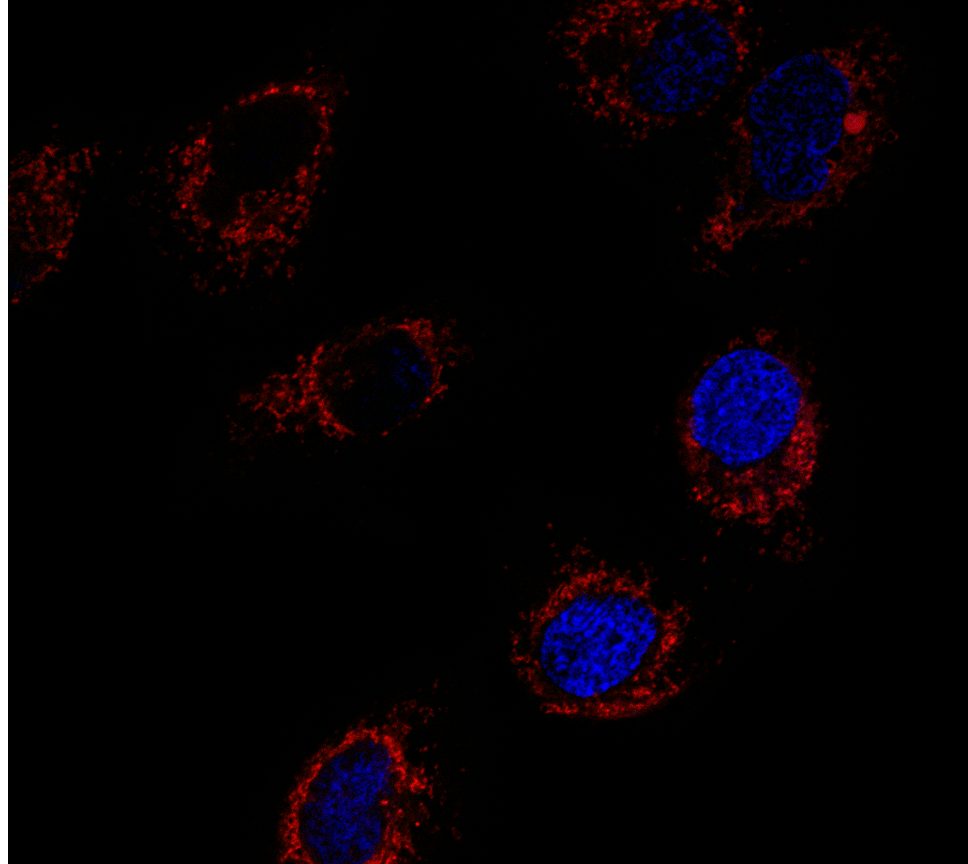

Supplement: Figure 8—source data 1. [file elife-69916-fig8-data1.zip › Figure8_Sourcedata_localization of Endonuclease G/Figure 8C_Representative image_Co IF_TFAM and EndoG/Figure 3H_Representative image_Co IF_TFAM and EndoG_Sec control_merged.tif]

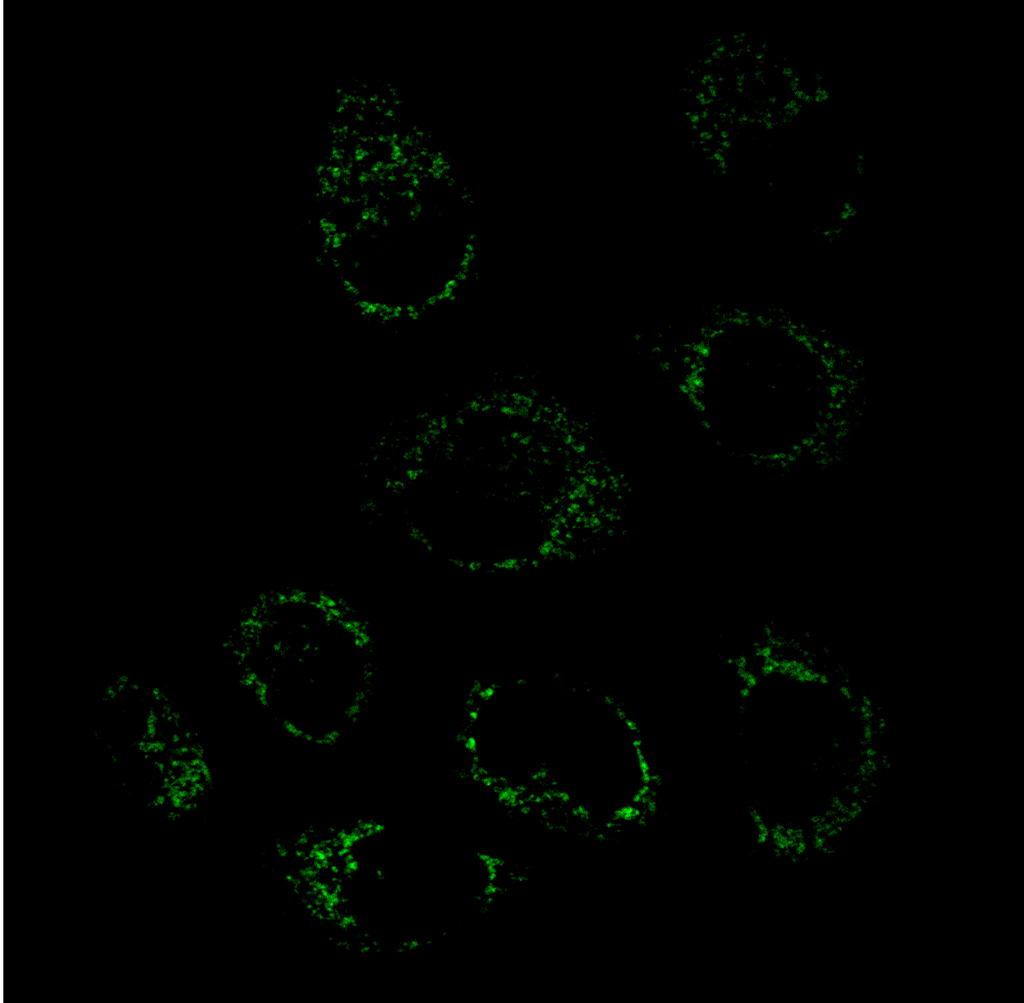

Supplement: Figure 8—source data 1. [file elife-69916-fig8-data1.zip › Figure8_Sourcedata_localization of Endonuclease G/Figure 8C_Representative image_Co IF_TFAM and EndoG/Figure 3H_Representative image_Co IF_TFAM and EndoG_Sec control2_EndoG.tif]

## Slide 1
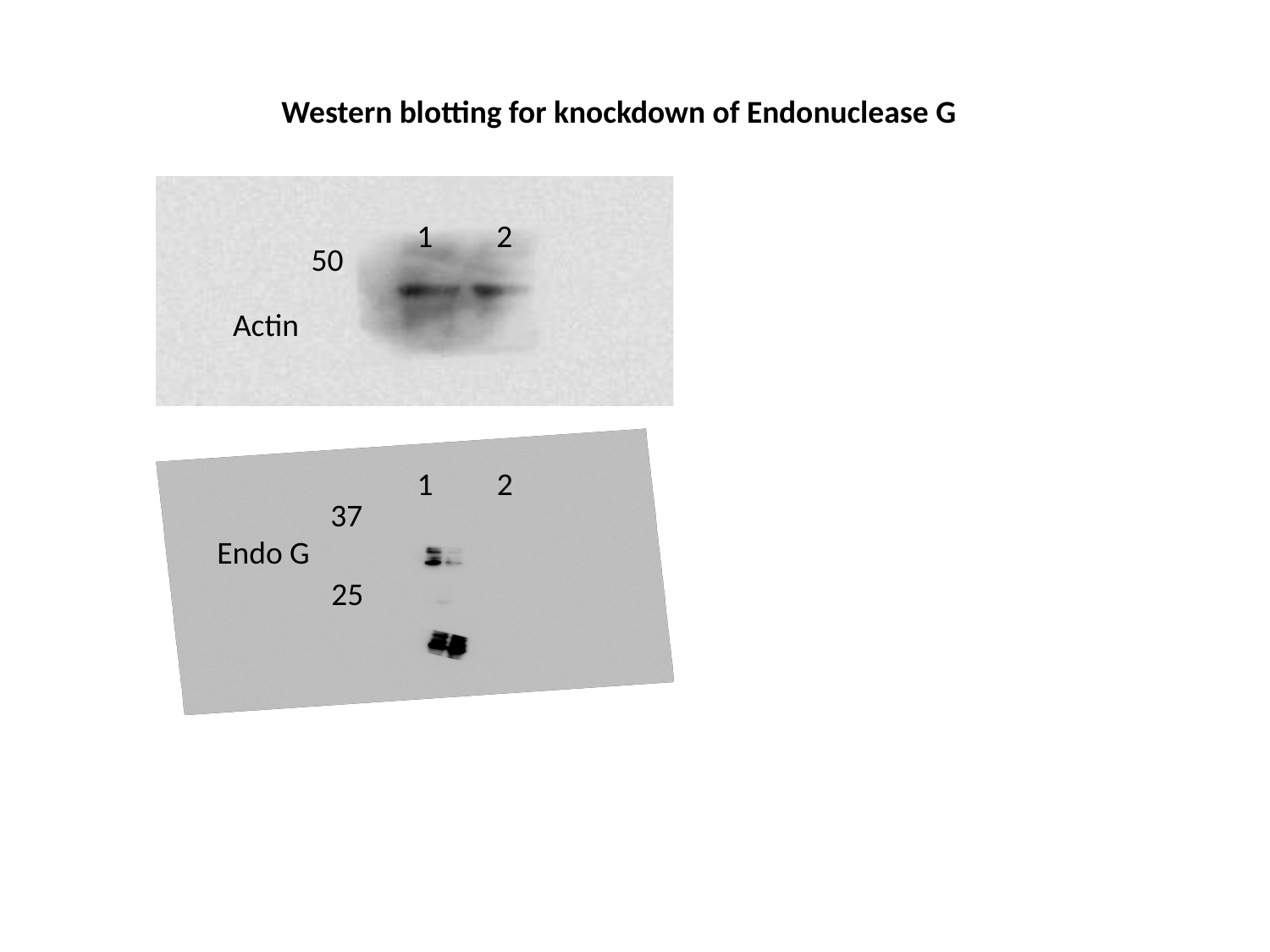

Western blotting for knockdown of Endonuclease G
1
2
50
Actin
1
2
37
Endo G
25

Supplement: Figure 8—source data 1. [file elife-69916-fig8-data1.zip › Figure8_Sourcedata_localization of Endonuclease G/Figure 8E_Western blotting_shRNA mediated knockdown of EndoG/Figure 8E_Source data_Label.pptx]

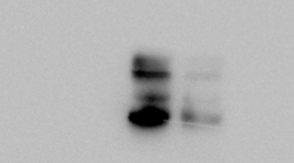

Supplement: Figure 8—source data 1. [file elife-69916-fig8-data1.zip › Figure8_Sourcedata_localization of Endonuclease G/Figure 8E_Western blotting_shRNA mediated knockdown of EndoG/Figure 8E_Gel image for EndoG_shRNA mediated knockdown.tif]

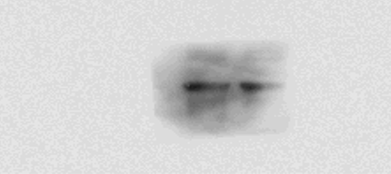

Supplement: Figure 8—source data 1. [file elife-69916-fig8-data1.zip › Figure8_Sourcedata_localization of Endonuclease G/Figure 8E_Western blotting_shRNA mediated knockdown of EndoG/Figure 3O_Gel image for Actin_shRNA mediated knockdown.tif]

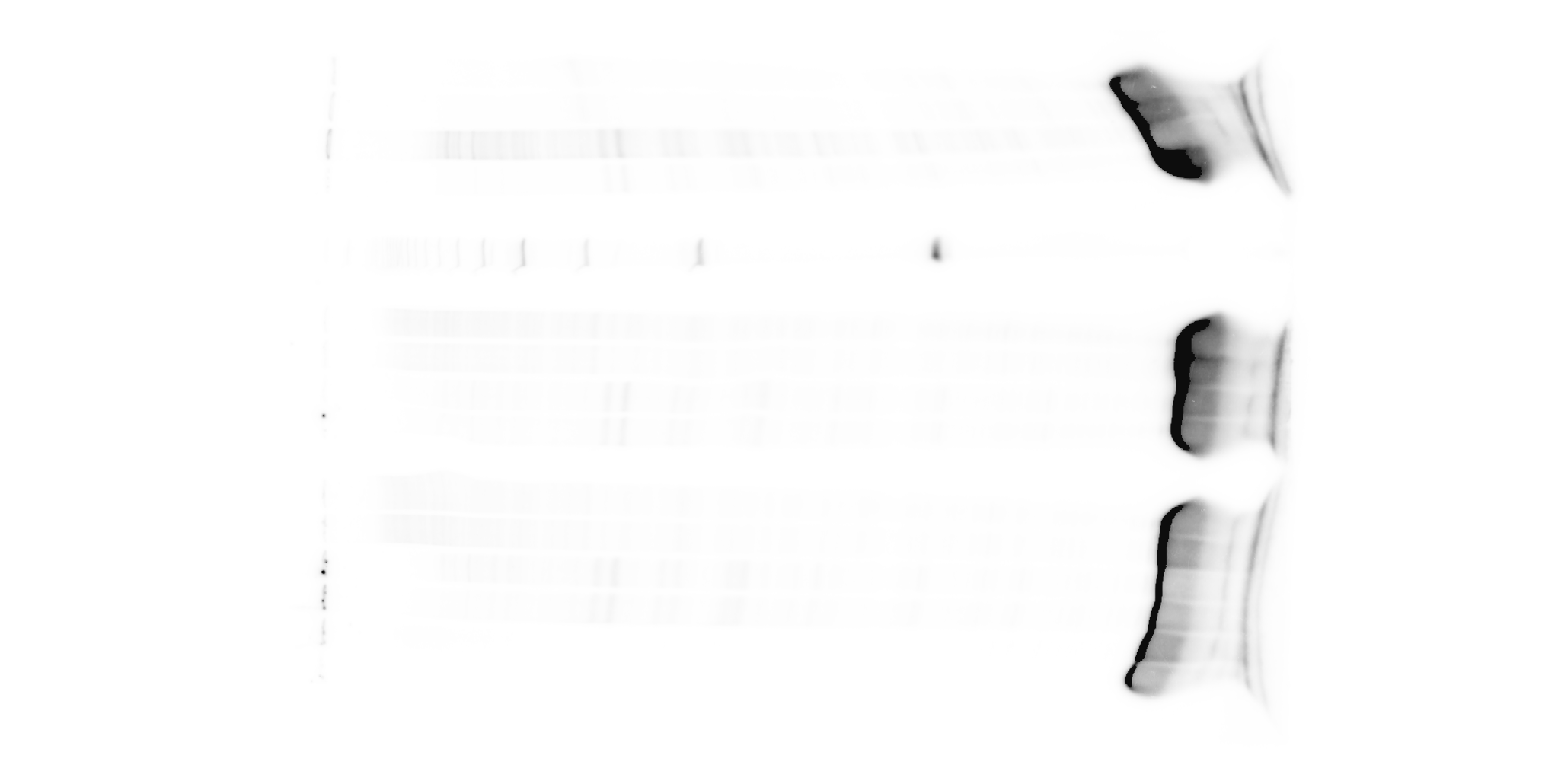

Supplement: Figure 8—source data 1. [file elife-69916-fig8-data1.zip › Figure8_Sourcedata_localization of Endonuclease G/Figure 8F_Primer extension_Endonuclease G_knockdown/Figure 8F_primer extension_Endonuclease G_shRNA mediated cleavage.tif]
