# Supplementary material for: Unleashing a novel function of Endonuclease G in mitochondrial genome instability
Source: eLife. 2022 Nov 17;11:e69916. doi: 10.7554/eLife.69916 (PMC9711528; doi:10.7554/eLife.69916)
Supplement: Figure 5—source data 1. [file elife-69916-fig5-data1.zip › Figure5_Source data_BG4 ChIP pulldown/Figure 5C_Gel profile_mitoChIP_BG4 pulldown/Figure 2G_Gel_mitoChIP_BG4.pptx]

## Slide 1
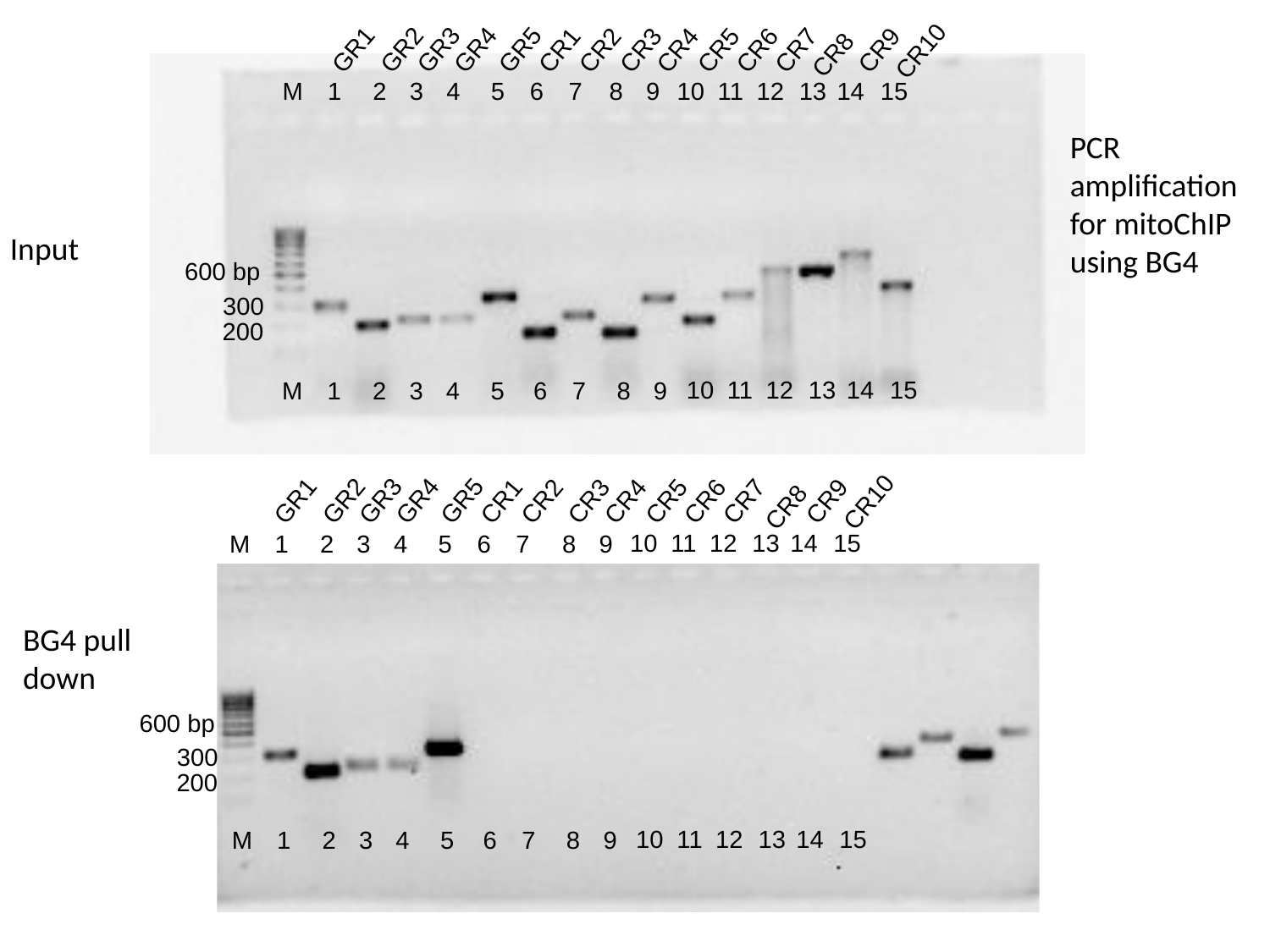

CR8
10
11
12
13
14
15
M
1
2
3
4
5
6
7
8
9
600 bp
300
200
10
11
12
13
14
15
M
1
2
3
4
5
6
7
8
9
GR1
GR2
GR3
GR4
GR5
CR1
CR2
CR3
CR4
CR5
CR6
CR7
CR9
CR10
PCR amplification for mitoChIP using BG4
Input
GR1
GR2
GR3
GR4
GR5
CR1
CR2
CR3
CR4
CR5
CR6
CR7
CR9
CR10
CR8
10
11
12
13
14
15
M
1
2
3
4
5
6
7
8
9
600 bp
300
200
10
11
12
13
14
15
M
1
2
3
4
5
6
7
8
9
BG4 pull down
