# Supplementary material for: Unleashing a novel function of Endonuclease G in mitochondrial genome instability
Source: eLife. 2022 Nov 17;11:e69916. doi: 10.7554/eLife.69916 (PMC9711528; doi:10.7554/eLife.69916)
Supplement: Figure 6—source data 1. [file elife-69916-fig6-data1.zip › Figure6_Sourcedata_activity assay mitochondrial extracts/Figure 6B_Activity assay of mitochondrial extract/Figure 6B_Activity assay of mitochondrial extracts.pptx]

## Slide 1
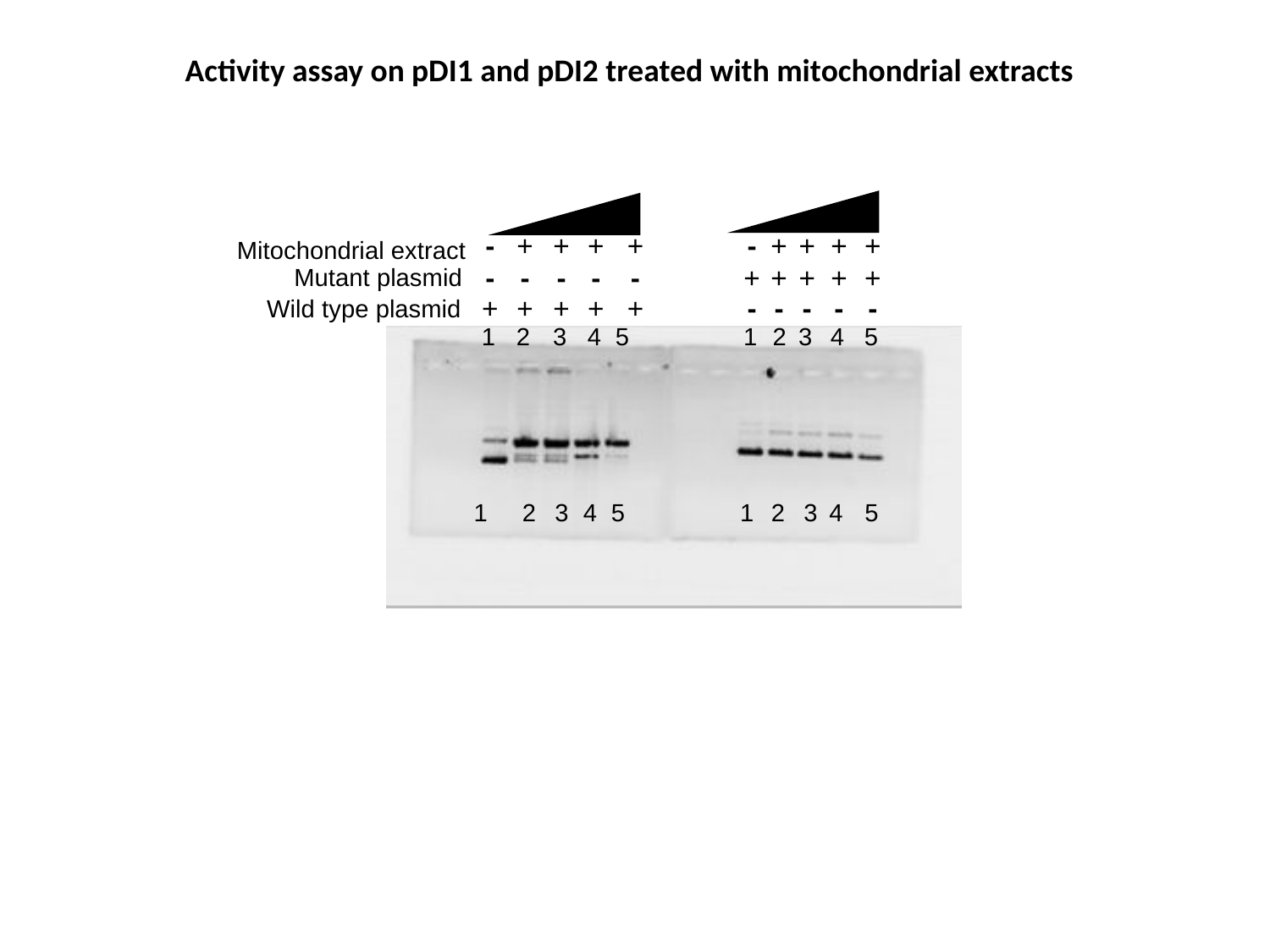

Activity assay on pDI1 and pDI2 treated with mitochondrial extracts
-
+
+
+
+
-
+
+
+
+
Mitochondrial extract
-
-
-
-
-
+
+
+
+
+
Mutant plasmid
+
+
+
+
+
-
-
-
-
-
Wild type plasmid
1
2
3
4
5
1
2
3
4
5
1
2
3
4
5
1
2
3
4
5
