# Supplementary material for: Unleashing a novel function of Endonuclease G in mitochondrial genome instability
Source: eLife. 2022 Nov 17;11:e69916. doi: 10.7554/eLife.69916 (PMC9711528; doi:10.7554/eLife.69916)
Supplement: Figure 6—source data 1. [file elife-69916-fig6-data1.zip › Figure6_Sourcedata_activity assay mitochondrial extracts/Figure 6E_Primer extension_Mitochondrial extracts_pDI1 vs pDI2/Figure 6E_Primer extension_mitochondrial testes extracts on pDI1 vs pDI2.pptx]

## Slide 1
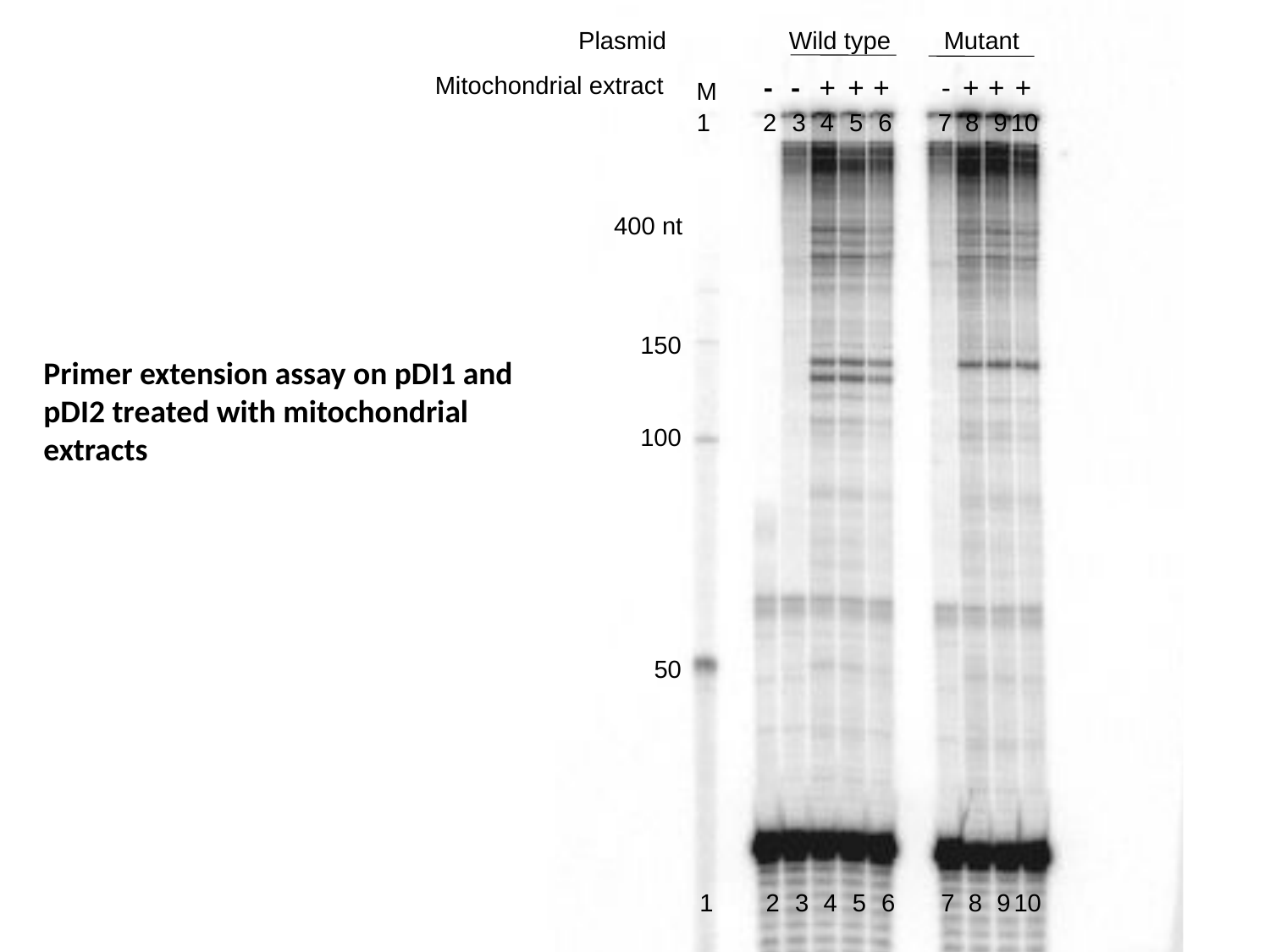

Plasmid
Wild type
Mutant
Mitochondrial extract
M
-
-
+
+
+
-
+
+
+
1
2
3
4
5
6
7
8
9
10
400 nt
150
100
50
1
2
3
4
5
6
7
8
9
10
Primer extension assay on pDI1 and pDI2 treated with mitochondrial extracts
