# Supplementary material for: Unleashing a novel function of Endonuclease G in mitochondrial genome instability
Source: eLife. 2022 Nov 17;11:e69916. doi: 10.7554/eLife.69916 (PMC9711528; doi:10.7554/eLife.69916)
Supplement: Figure 6—source data 1. [file elife-69916-fig6-data1.zip › Figure6_Sourcedata_activity assay mitochondrial extracts/Figure 6D_Primer extension_mitochondrial extracts/Figure 6D_Primer extension_mitochondrial testes and spleen extracts extracts.pptx]

## Slide 1
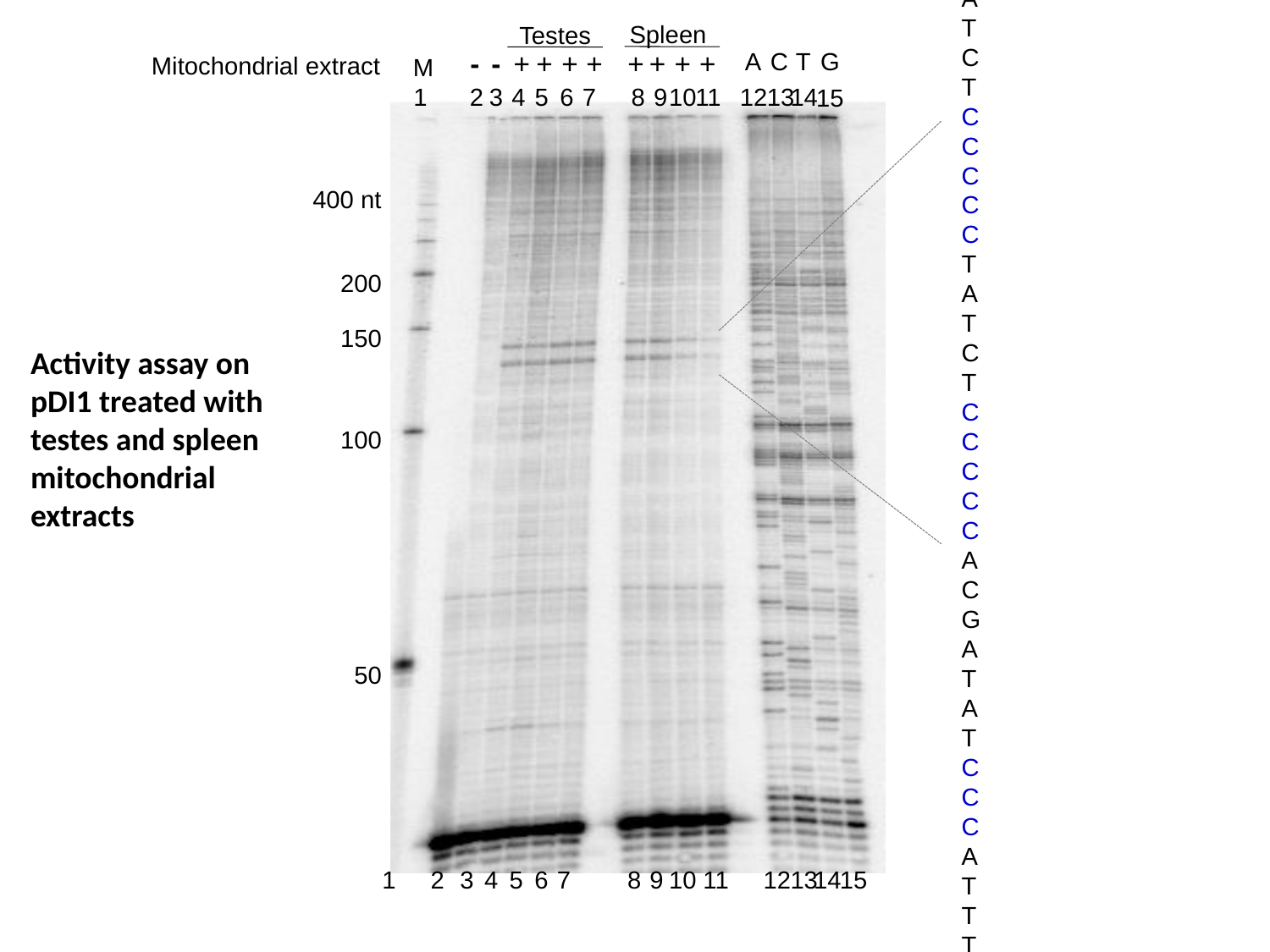

3’
C
C
C
G
A
G
A
T
C
T
C
C
C
C
C
T
A
T
C
T
C
C
C
C
C
A
C
G
A
T
A
T
C
C
C
A
T
T
T
A
T
G
C
C
C
G
G
G
A
T
5’
Spleen
Testes
M
-
-
+
+
+
+
+
+
+
+
A
C
T
G
Mitochondrial extract
14
1
2
3
4
5
6
7
8
9
10
11
12
13
15
400 nt
200
150
Activity assay on pDI1 treated with testes and spleen mitochondrial extracts
100
50
1
2
3
4
5
6
7
8
9
10
11
12
13
14
15
