# Supplementary material for: Unleashing a novel function of Endonuclease G in mitochondrial genome instability
Source: eLife. 2022 Nov 17;11:e69916. doi: 10.7554/eLife.69916 (PMC9711528; doi:10.7554/eLife.69916)

SciGenom Trace Viewer

Sample :pDI2_VKK11_10017-4_5557
Trim Start :31
Trim End :973
Qv20 Bases :942

Run start: 2013/06/11 09:46:32
Run stop: 2013/06/11 12:02:06
PDF created: 2013/06/11 12:18:37

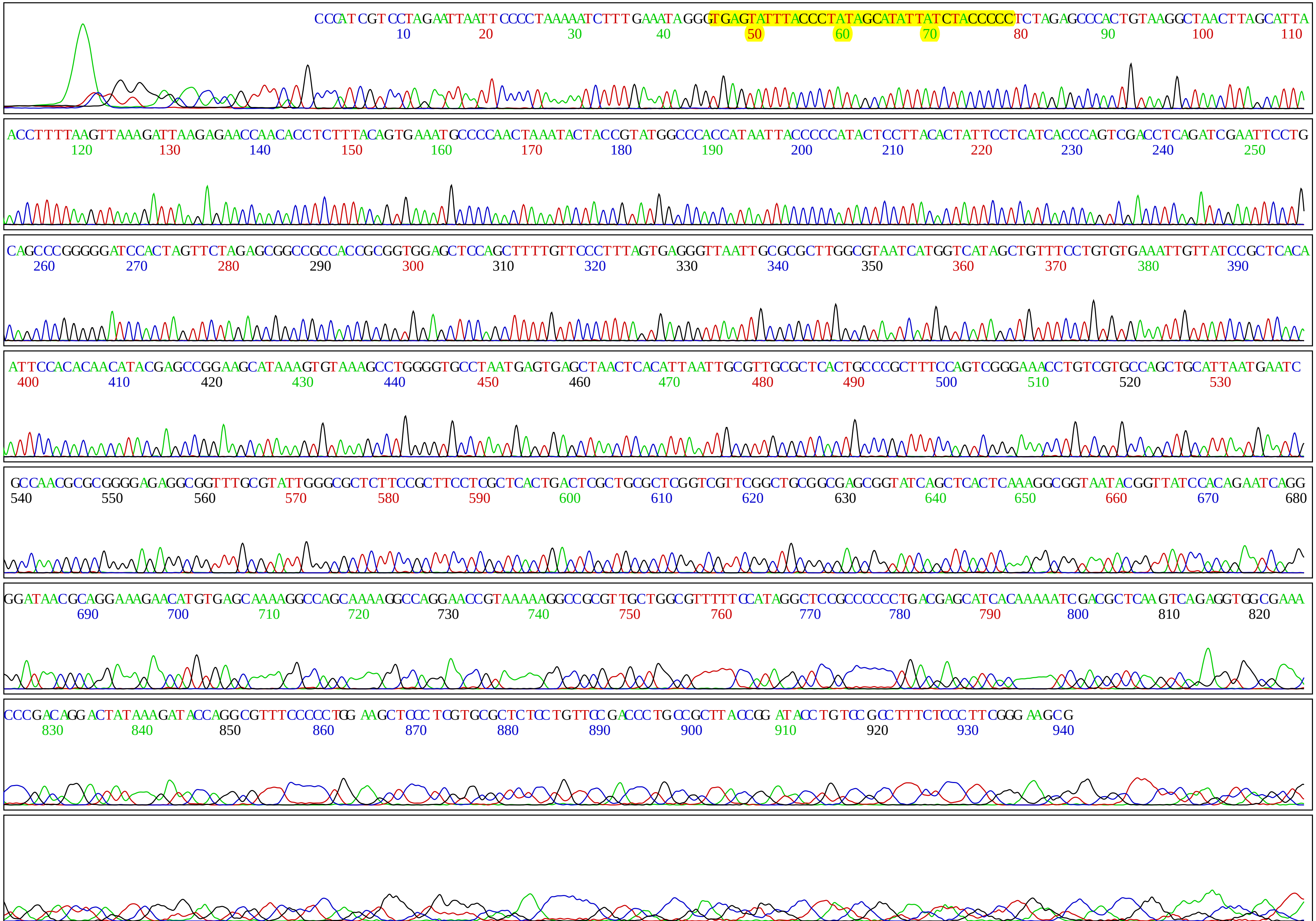

Supplement: Figure 6—source data 2. [file elife-69916-fig6-data2.zip › Figure6_Sourcedata_Supplementary/Figure S6A_pDI2 sequencing.pdf]
