# Supplementary material for: Unleashing a novel function of Endonuclease G in mitochondrial genome instability
Source: eLife. 2022 Nov 17;11:e69916. doi: 10.7554/eLife.69916 (PMC9711528; doi:10.7554/eLife.69916)
Supplement: Figure 6—source data 2. [file elife-69916-fig6-data2.zip › Figure6_Sourcedata_Supplementary/Figure S6B_Cleavage assay on plasmids bearing the wild type and mutant plasmid.pptx]

## Slide 1
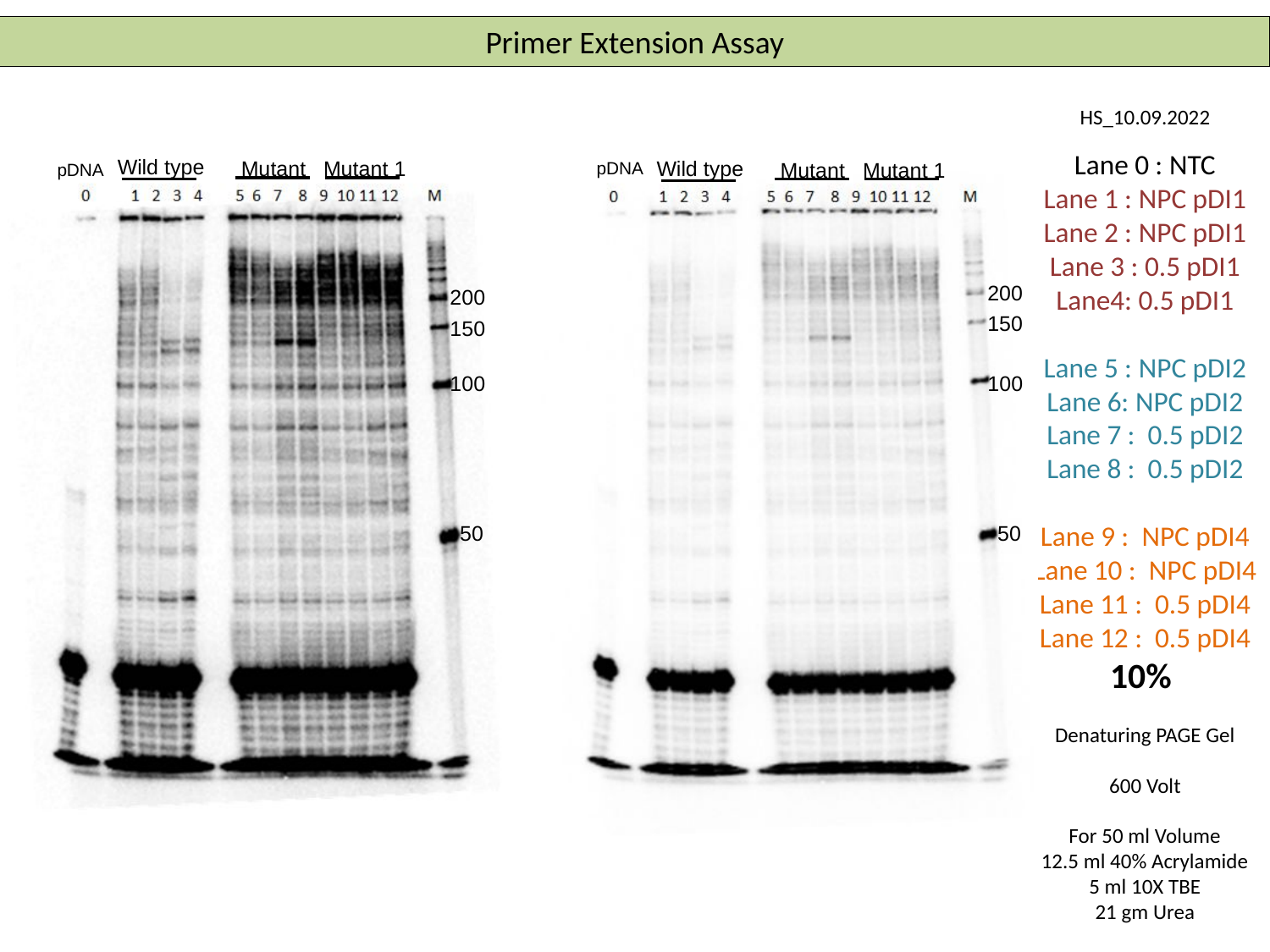

Primer Extension Assay
HS_10.09.2022
Lane 0 : NTC
Lane 1 : NPC pDI1
Lane 2 : NPC pDI1
Lane 3 : 0.5 pDI1
Lane4: 0.5 pDI1
Lane 5 : NPC pDI2
Lane 6: NPC pDI2
Lane 7 : 0.5 pDI2
Lane 8 : 0.5 pDI2
Lane 9 : NPC pDI4
Lane 10 : NPC pDI4
Lane 11 : 0.5 pDI4
Lane 12 : 0.5 pDI4
Wild type
Wild type
Mutant
Mutant 1
Mutant
Mutant 1
pDNA
pDNA
200
200
150
50
150
100
100
50
50
10%
Denaturing PAGE Gel
600 Volt
For 50 ml Volume
12.5 ml 40% Acrylamide
5 ml 10X TBE
21 gm Urea
