# Supplementary material for: Unleashing a novel function of Endonuclease G in mitochondrial genome instability
Source: eLife. 2022 Nov 17;11:e69916. doi: 10.7554/eLife.69916 (PMC9711528; doi:10.7554/eLife.69916)
Supplement: Figure 7—source data 1. [file elife-69916-fig7-data1.zip › Figure 7_Sourcedata_activity of Endonuclease G/Figure 7J_Primer extension_IP_reconstitution/Figure 7J_Primer extn_IP_reconstitution.pptx]

## Slide 1
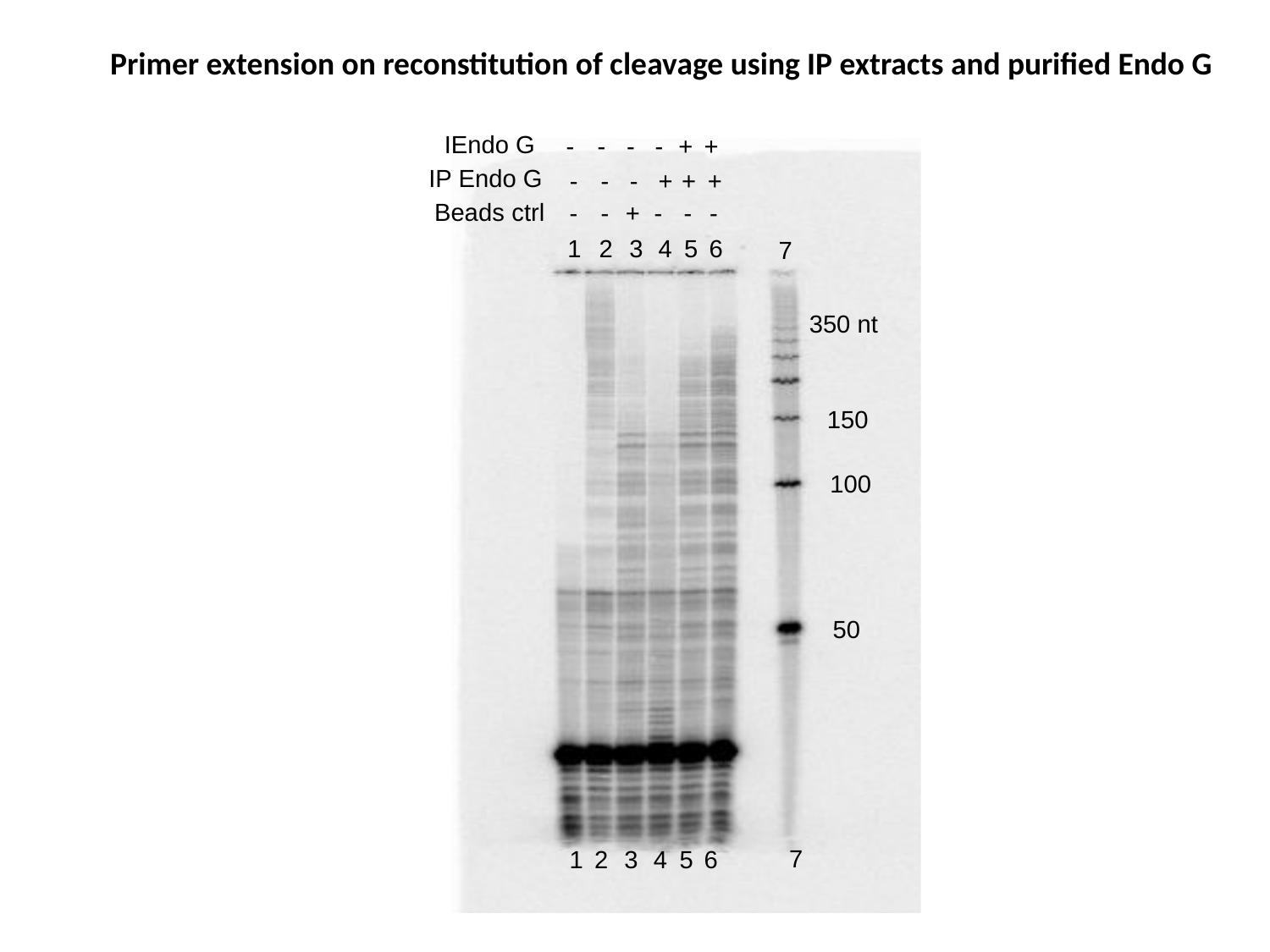

Primer extension on reconstitution of cleavage using IP extracts and purified Endo G
-
-
-
-
+
+
IEndo G
-
-
-
+
+
+
IP Endo G
-
-
+
-
-
-
Beads ctrl
1
2
3
4
5
6
7
350 nt
150
100
50
7
1
2
3
4
5
6
