# Supplementary material for: Unleashing a novel function of Endonuclease G in mitochondrial genome instability
Source: eLife. 2022 Nov 17;11:e69916. doi: 10.7554/eLife.69916 (PMC9711528; doi:10.7554/eLife.69916)
Supplement: Figure 7—source data 1. [file elife-69916-fig7-data1.zip › Figure 7_Sourcedata_activity of Endonuclease G/Figure 7H_Gel_Primer extension_Different endonuclease immunodepletion/FIgure 7H_Gel_Primer extension_Different nucleases IP.pptx]

## Slide 1
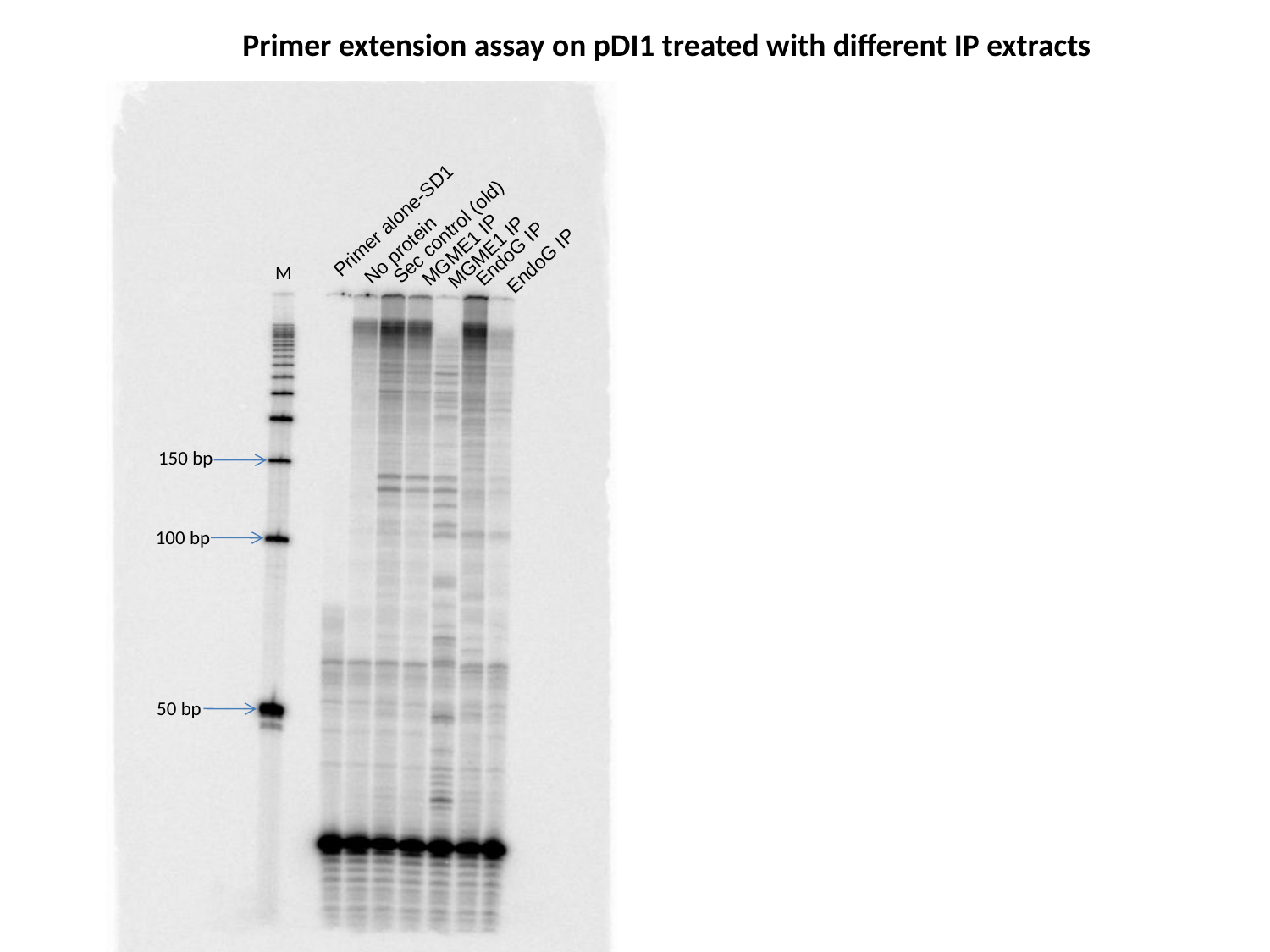

Primer extension assay on pDI1 treated with different IP extracts
Primer alone-SD1
Sec control (old)
No protein
MGME1 IP
MGME1 IP
EndoG IP
EndoG IP
 M
150 bp
100 bp
50 bp
