# Supplementary material for: Unleashing a novel function of Endonuclease G in mitochondrial genome instability
Source: eLife. 2022 Nov 17;11:e69916. doi: 10.7554/eLife.69916 (PMC9711528; doi:10.7554/eLife.69916)
Supplement: Figure 7—source data 1. [file elife-69916-fig7-data1.zip › Figure 7_Sourcedata_activity of Endonuclease G/Figure 7A_Activity assay_purified Endonuclease/Figure 7A_Activity assay of purified Endonuclease G for wild type and mutant.pptx]

## Slide 1
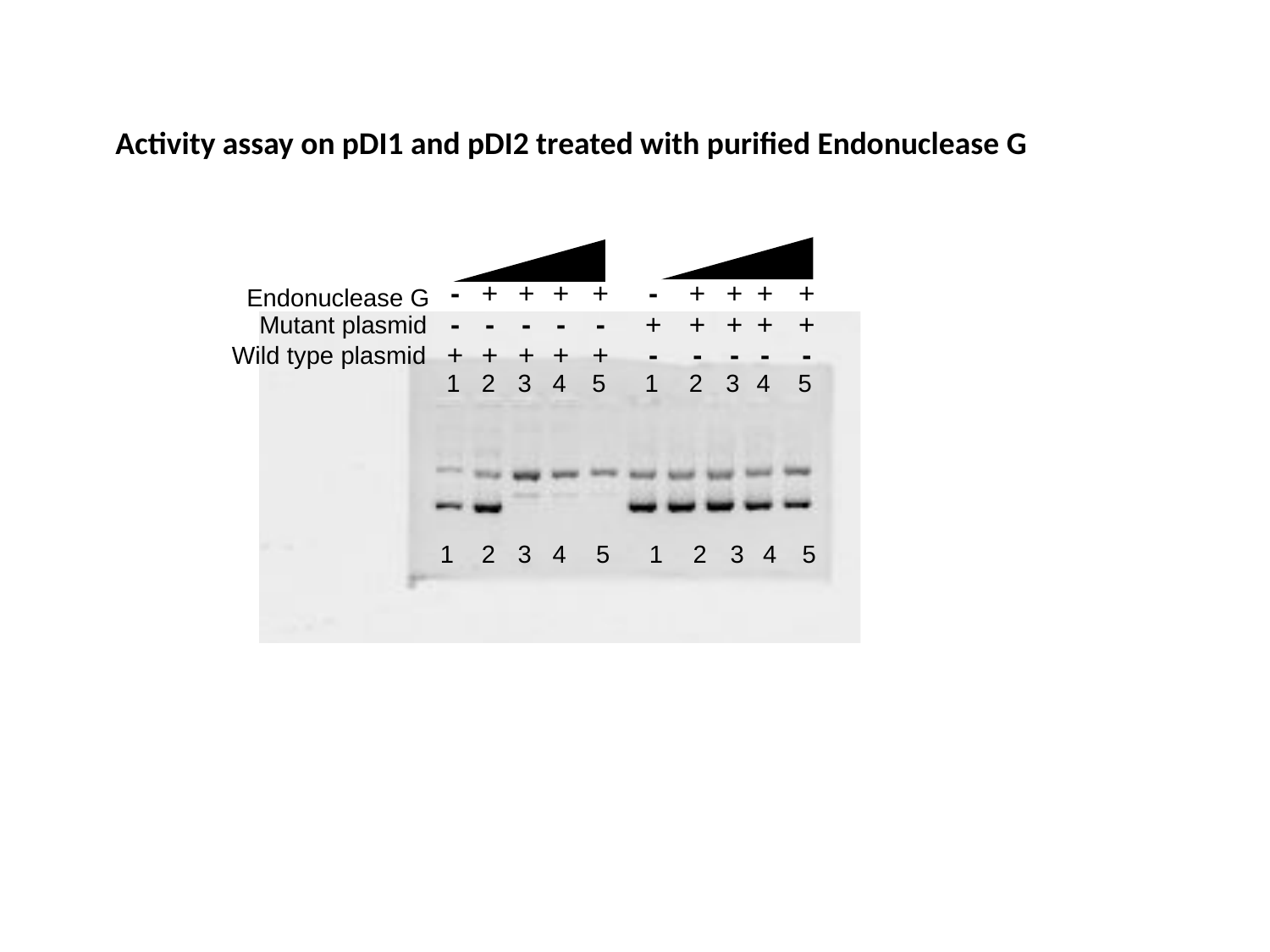

Activity assay on pDI1 and pDI2 treated with purified Endonuclease G
-
+
+
+
+
-
+
+
+
+
Endonuclease G
-
-
-
-
-
+
+
+
+
+
Mutant plasmid
+
+
+
+
+
-
-
-
-
-
Wild type plasmid
1
2
3
4
5
1
2
3
4
5
1
2
3
4
5
1
2
3
4
5
