# Supplementary material for: Unleashing a novel function of Endonuclease G in mitochondrial genome instability
Source: eLife. 2022 Nov 17;11:e69916. doi: 10.7554/eLife.69916 (PMC9711528; doi:10.7554/eLife.69916)
Supplement: Figure 8—source data 1. [file elife-69916-fig8-data1.zip › Figure8_Sourcedata_localization of Endonuclease G/Figure 8F_Primer extension_Endonuclease G_knockdown/Figure 8F_Primer extension_Endonuclease G knockdown.pptx]

## Slide 1
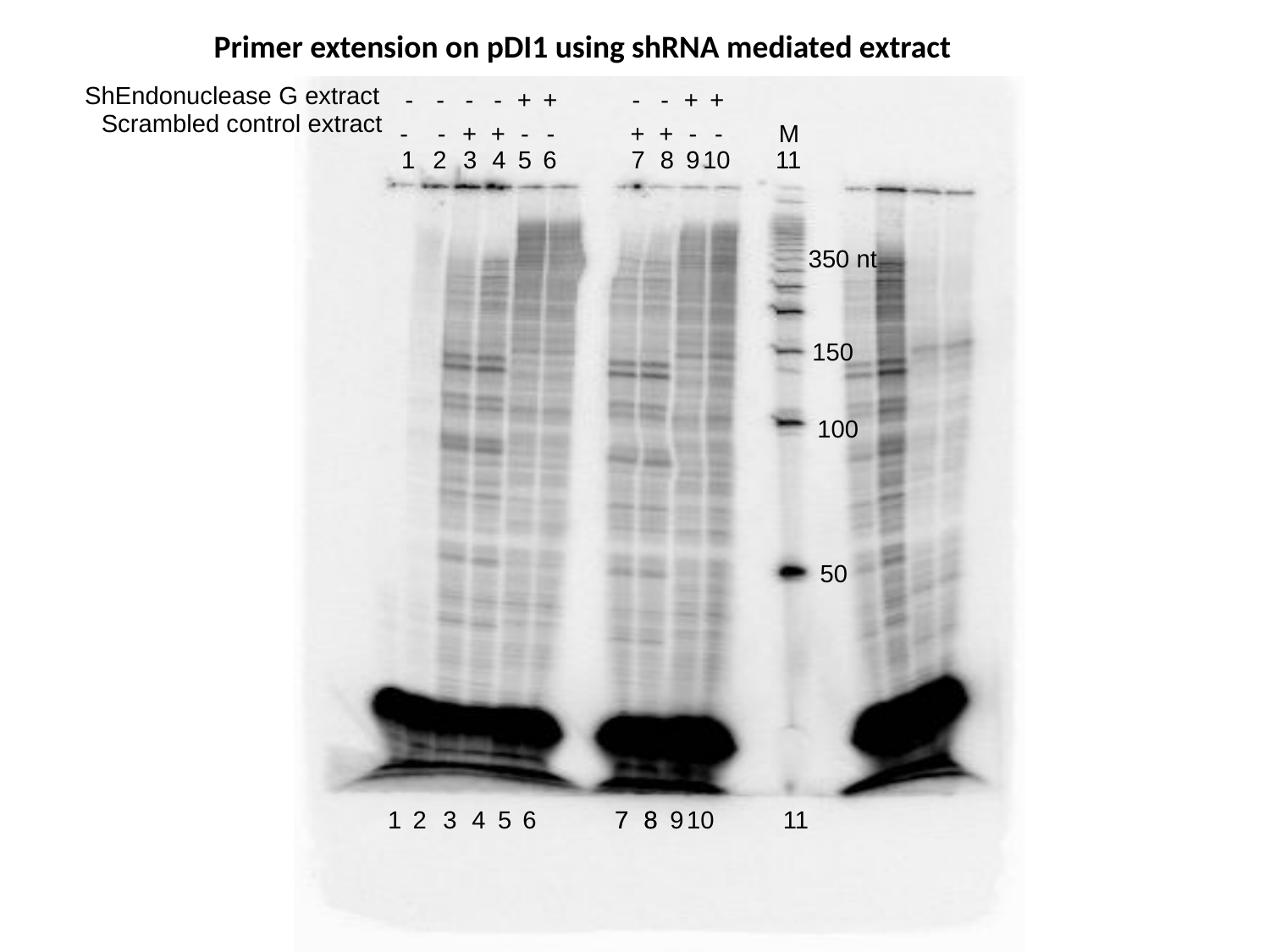

Primer extension on pDI1 using shRNA mediated extract
-
-
-
-
+
+
-
-
+
+
ShEndonuclease G extract
Scrambled control extract
-
-
+
+
-
-
+
+
-
-
M
1
2
3
4
5
6
7
8
9
10
11
350 nt
150
100
50
1
2
3
4
5
6
7
7
8
8
9
10
11
