# Supplementary figures and images for: Unleashing a novel function of Endonuclease G in mitochondrial genome instability (part 4 of 4)
Source: eLife. 2022 Nov 17;11:e69916. doi: 10.7554/eLife.69916 (PMC9711528; doi:10.7554/eLife.69916)

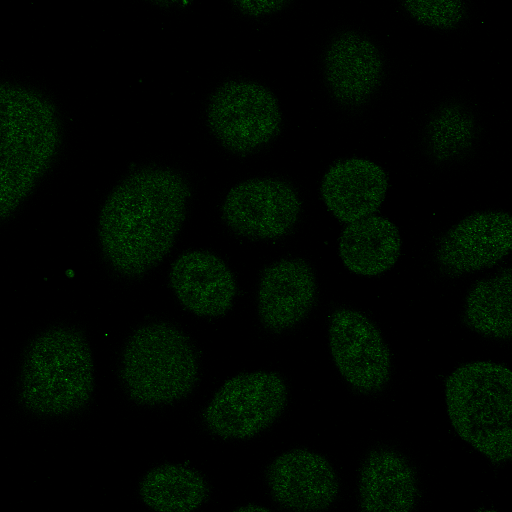

Supplement: Figure 8—source data 1. [file elife-69916-fig8-data1.zip › Figure8_Sourcedata_localization of Endonuclease G/Figure 8A_Representative images_localization of EndoG to mitochondria/Figure 8A_Source file_2_HeLa/1.tif.frames/1_C003T001.tif]

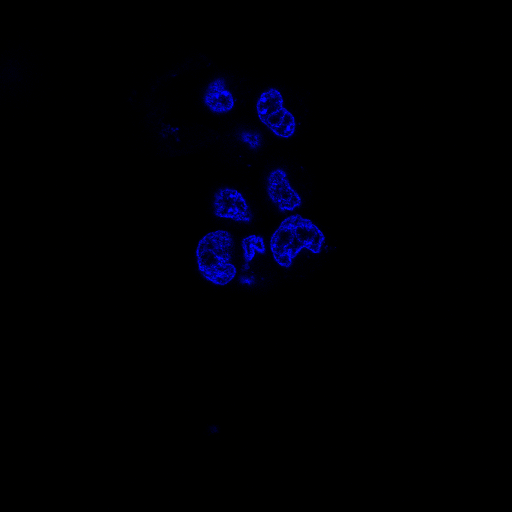

Supplement: Figure 8—source data 1. [file elife-69916-fig8-data1.zip › Figure8_Sourcedata_localization of Endonuclease G/Figure 8A_Representative images_localization of EndoG to mitochondria/Figure 8A_Source file_2_HeLa/1_0003.tif.frames/1_0003_C001T001.tif]

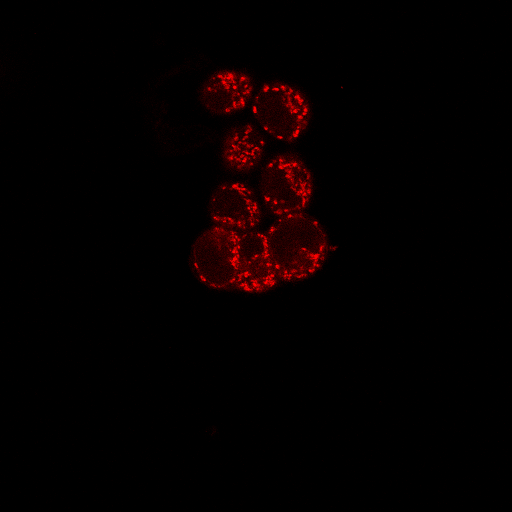

Supplement: Figure 8—source data 1. [file elife-69916-fig8-data1.zip › Figure8_Sourcedata_localization of Endonuclease G/Figure 8A_Representative images_localization of EndoG to mitochondria/Figure 8A_Source file_2_HeLa/1_0003.tif.frames/1_0003_C002T001.tif]

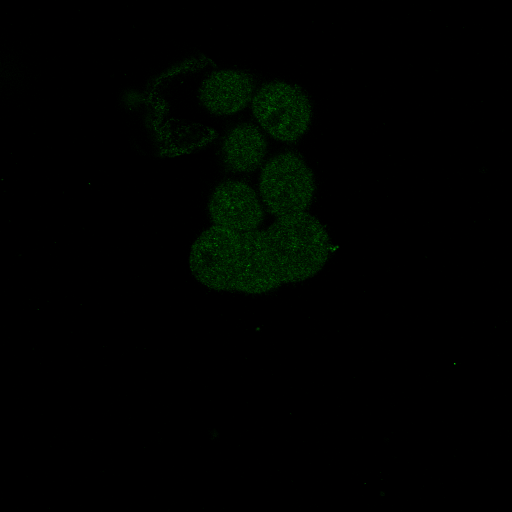

Supplement: Figure 8—source data 1. [file elife-69916-fig8-data1.zip › Figure8_Sourcedata_localization of Endonuclease G/Figure 8A_Representative images_localization of EndoG to mitochondria/Figure 8A_Source file_2_HeLa/1_0003.tif.frames/1_0003_C003T001.tif]

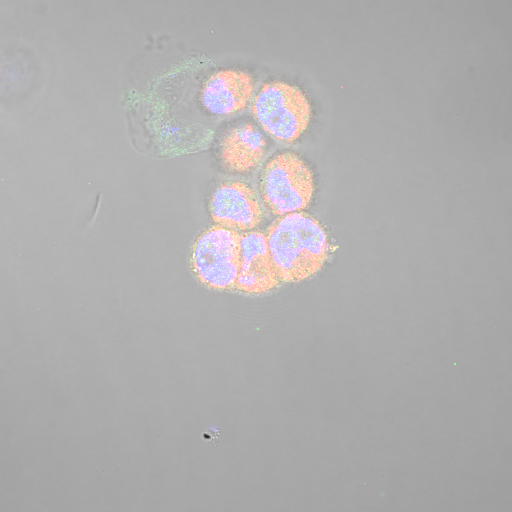

Supplement: Figure 8—source data 1. [file elife-69916-fig8-data1.zip › Figure8_Sourcedata_localization of Endonuclease G/Figure 8A_Representative images_localization of EndoG to mitochondria/Figure 8A_Source file_2_HeLa/1_0003.tif.frames/1_0003_T001.tif]

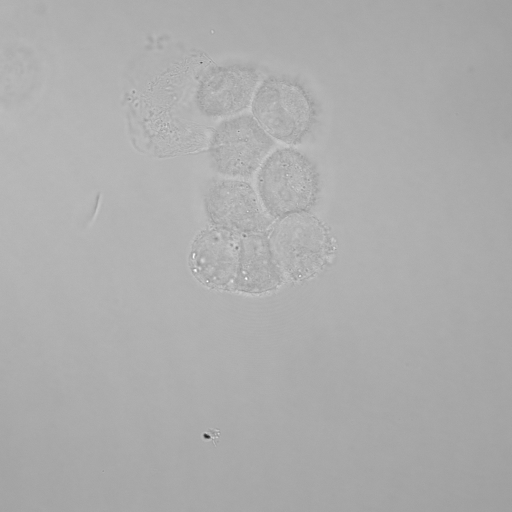

Supplement: Figure 8—source data 1. [file elife-69916-fig8-data1.zip › Figure8_Sourcedata_localization of Endonuclease G/Figure 8A_Representative images_localization of EndoG to mitochondria/Figure 8A_Source file_2_HeLa/1_0003.tif.frames/1_0003_C004T001.tif]

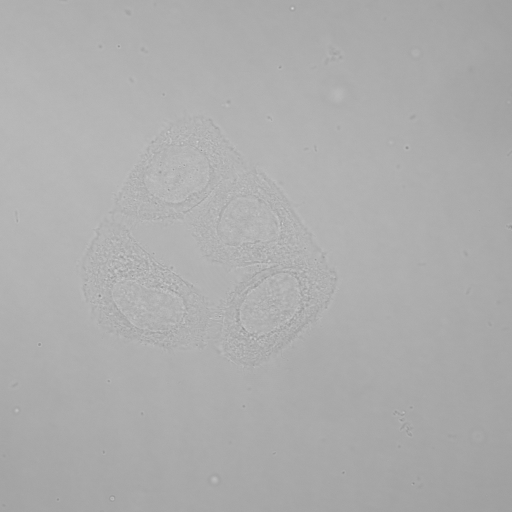

Supplement: Figure 8—source data 1. [file elife-69916-fig8-data1.zip › Figure8_Sourcedata_localization of Endonuclease G/Figure 8A_Representative images_localization of EndoG to mitochondria/Figure 8A_Source file_2_HeLa/1_0006.tif.frames/1_0006_C004T001.tif]

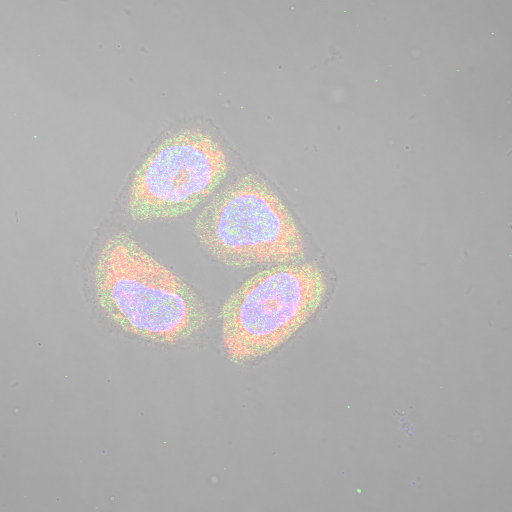

Supplement: Figure 8—source data 1. [file elife-69916-fig8-data1.zip › Figure8_Sourcedata_localization of Endonuclease G/Figure 8A_Representative images_localization of EndoG to mitochondria/Figure 8A_Source file_2_HeLa/1_0006.tif.frames/1_0006_T001.tif]

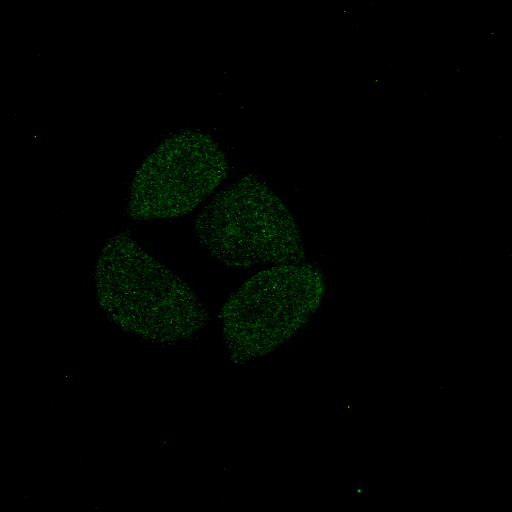

Supplement: Figure 8—source data 1. [file elife-69916-fig8-data1.zip › Figure8_Sourcedata_localization of Endonuclease G/Figure 8A_Representative images_localization of EndoG to mitochondria/Figure 8A_Source file_2_HeLa/1_0006.tif.frames/1_0006_C003T001.tif]

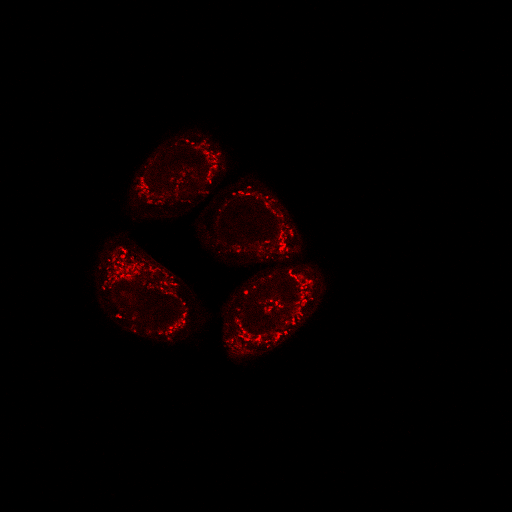

Supplement: Figure 8—source data 1. [file elife-69916-fig8-data1.zip › Figure8_Sourcedata_localization of Endonuclease G/Figure 8A_Representative images_localization of EndoG to mitochondria/Figure 8A_Source file_2_HeLa/1_0006.tif.frames/1_0006_C002T001.tif]

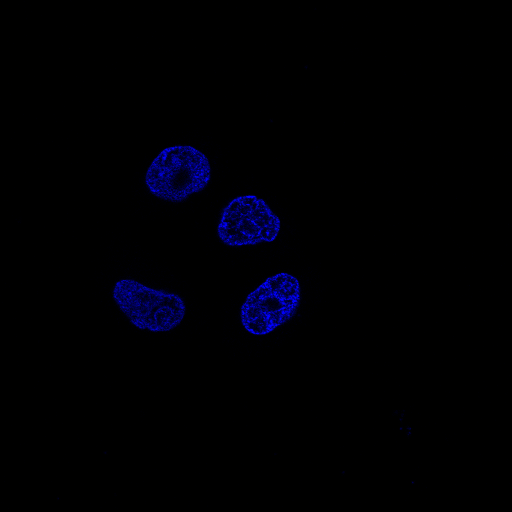

Supplement: Figure 8—source data 1. [file elife-69916-fig8-data1.zip › Figure8_Sourcedata_localization of Endonuclease G/Figure 8A_Representative images_localization of EndoG to mitochondria/Figure 8A_Source file_2_HeLa/1_0006.tif.frames/1_0006_C001T001.tif]

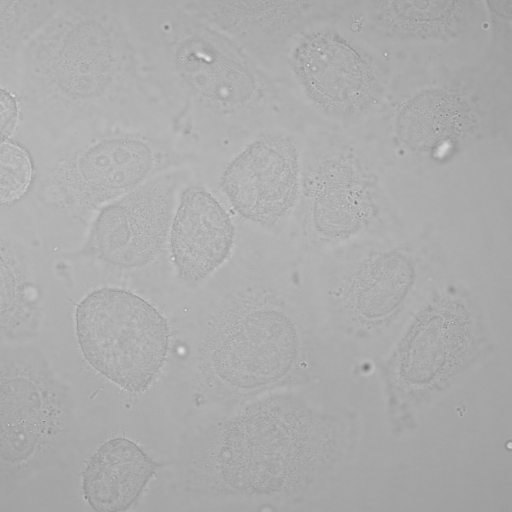

Supplement: Figure 8—source data 1. [file elife-69916-fig8-data1.zip › Figure8_Sourcedata_localization of Endonuclease G/Figure 8A_Representative images_localization of EndoG to mitochondria/Figure 8A_Source file_2_HeLa/1_0001.tif.frames/1_0001_C004T001.tif]

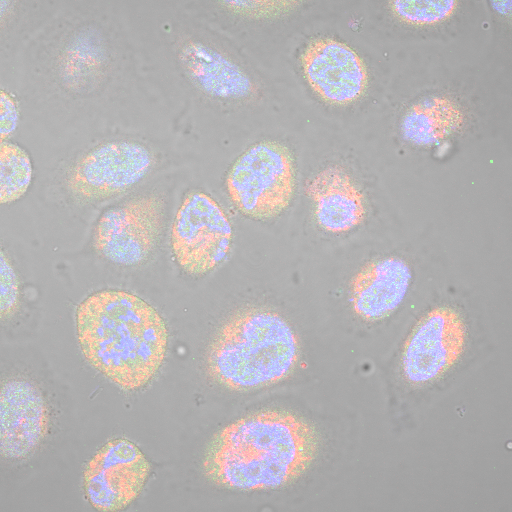

Supplement: Figure 8—source data 1. [file elife-69916-fig8-data1.zip › Figure8_Sourcedata_localization of Endonuclease G/Figure 8A_Representative images_localization of EndoG to mitochondria/Figure 8A_Source file_2_HeLa/1_0001.tif.frames/1_0001_T001.tif]

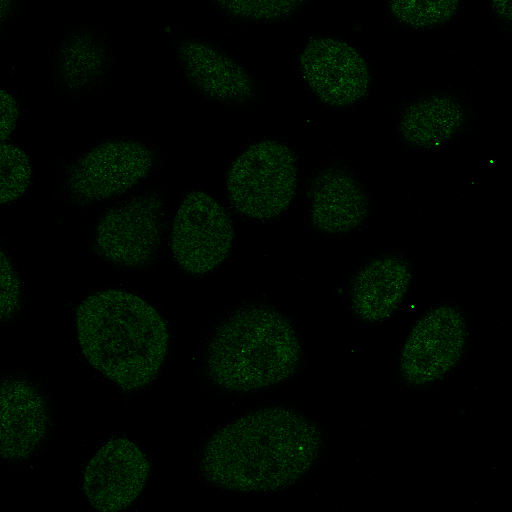

Supplement: Figure 8—source data 1. [file elife-69916-fig8-data1.zip › Figure8_Sourcedata_localization of Endonuclease G/Figure 8A_Representative images_localization of EndoG to mitochondria/Figure 8A_Source file_2_HeLa/1_0001.tif.frames/1_0001_C003T001.tif]

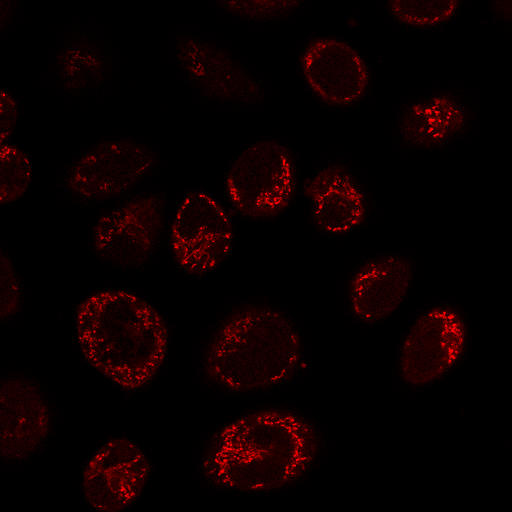

Supplement: Figure 8—source data 1. [file elife-69916-fig8-data1.zip › Figure8_Sourcedata_localization of Endonuclease G/Figure 8A_Representative images_localization of EndoG to mitochondria/Figure 8A_Source file_2_HeLa/1_0001.tif.frames/1_0001_C002T001.tif]

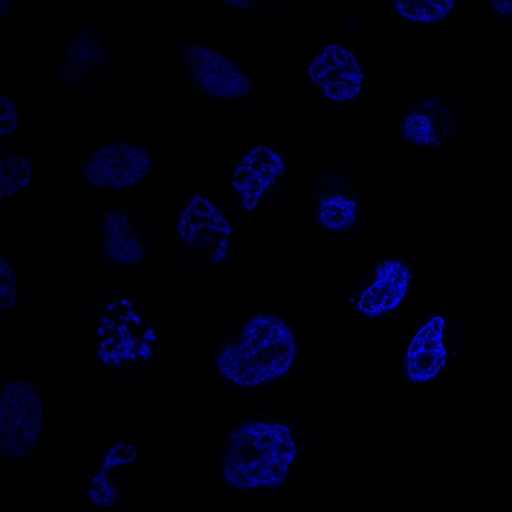

Supplement: Figure 8—source data 1. [file elife-69916-fig8-data1.zip › Figure8_Sourcedata_localization of Endonuclease G/Figure 8A_Representative images_localization of EndoG to mitochondria/Figure 8A_Source file_2_HeLa/1_0001.tif.frames/1_0001_C001T001.tif]

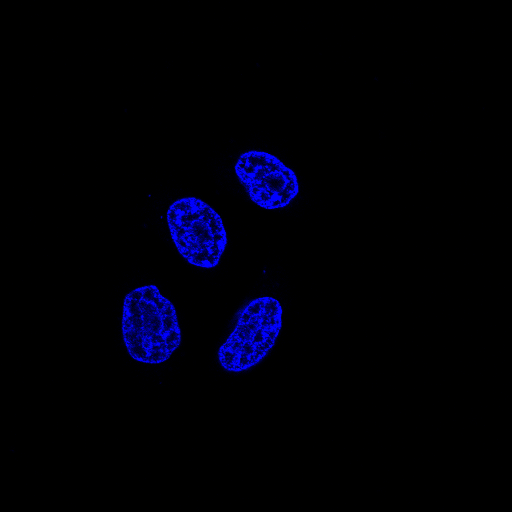

Supplement: Figure 8—source data 1. [file elife-69916-fig8-data1.zip › Figure8_Sourcedata_localization of Endonuclease G/Figure 8A_Representative images_localization of EndoG to mitochondria/Figure 8A_Source file_2_HeLa/1_0004.tif.frames/1_0004_C001T001.tif]

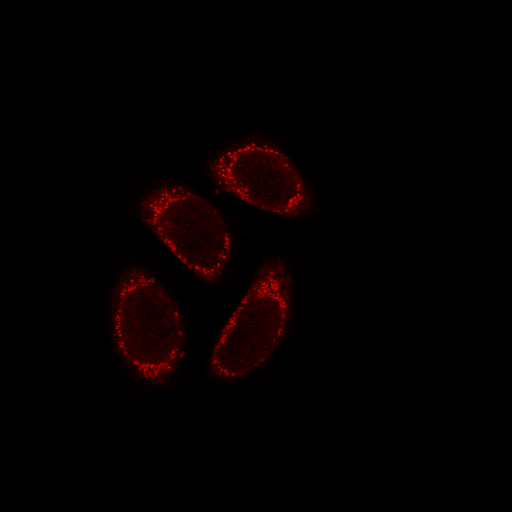

Supplement: Figure 8—source data 1. [file elife-69916-fig8-data1.zip › Figure8_Sourcedata_localization of Endonuclease G/Figure 8A_Representative images_localization of EndoG to mitochondria/Figure 8A_Source file_2_HeLa/1_0004.tif.frames/1_0004_C002T001.tif]

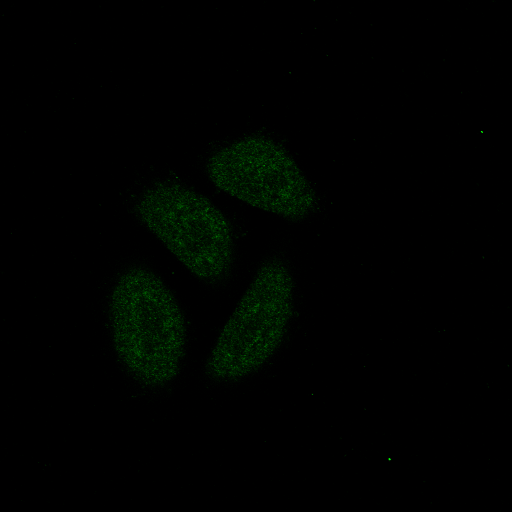

Supplement: Figure 8—source data 1. [file elife-69916-fig8-data1.zip › Figure8_Sourcedata_localization of Endonuclease G/Figure 8A_Representative images_localization of EndoG to mitochondria/Figure 8A_Source file_2_HeLa/1_0004.tif.frames/1_0004_C003T001.tif]

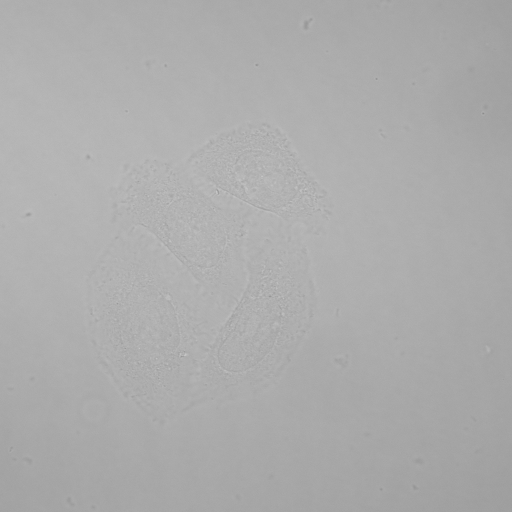

Supplement: Figure 8—source data 1. [file elife-69916-fig8-data1.zip › Figure8_Sourcedata_localization of Endonuclease G/Figure 8A_Representative images_localization of EndoG to mitochondria/Figure 8A_Source file_2_HeLa/1_0004.tif.frames/1_0004_C004T001.tif]

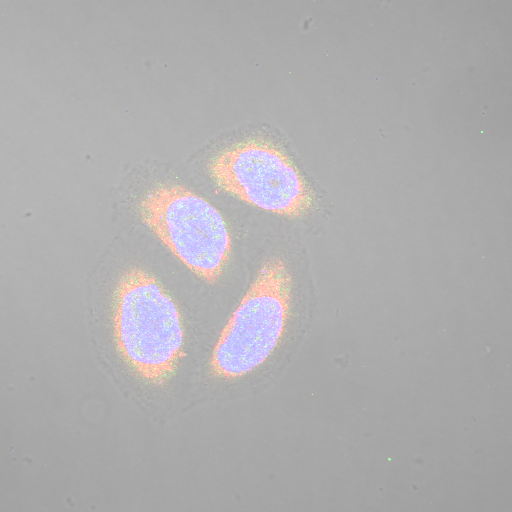

Supplement: Figure 8—source data 1. [file elife-69916-fig8-data1.zip › Figure8_Sourcedata_localization of Endonuclease G/Figure 8A_Representative images_localization of EndoG to mitochondria/Figure 8A_Source file_2_HeLa/1_0004.tif.frames/1_0004_T001.tif]

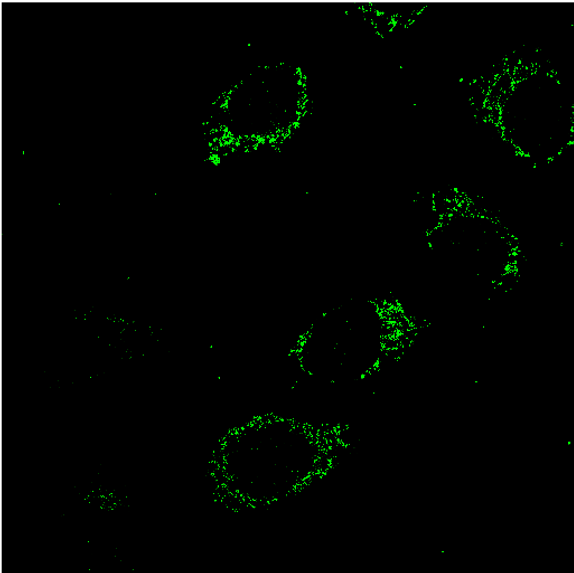

Supplement: Figure 8—source data 2. [file elife-69916-fig8-data2.zip › Figure 8_Source data_Supplementary/Figure S8_Image_EndoG colocalization_mitotracker green/Figure S4B_Representative image_EndoG and localization_HeLa_mitotracker green_mitotracker green.Tif]

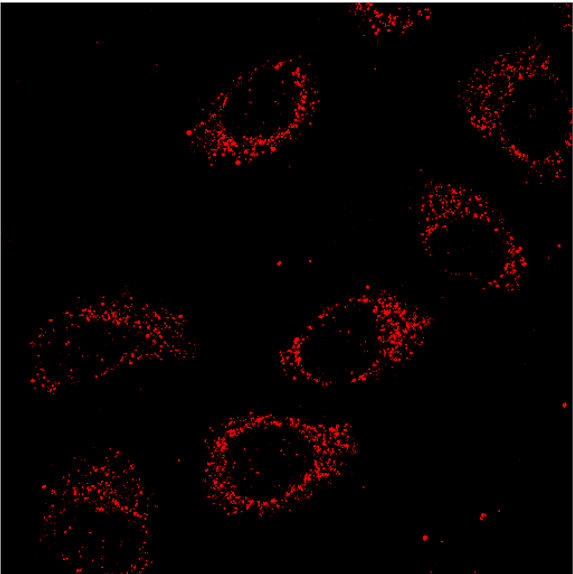

Supplement: Figure 8—source data 2. [file elife-69916-fig8-data2.zip › Figure 8_Source data_Supplementary/Figure S8_Image_EndoG colocalization_mitotracker green/Figure S4B_Representative image_EndoG and localization_HeLa_mitotracker green_EndoG.Tif]

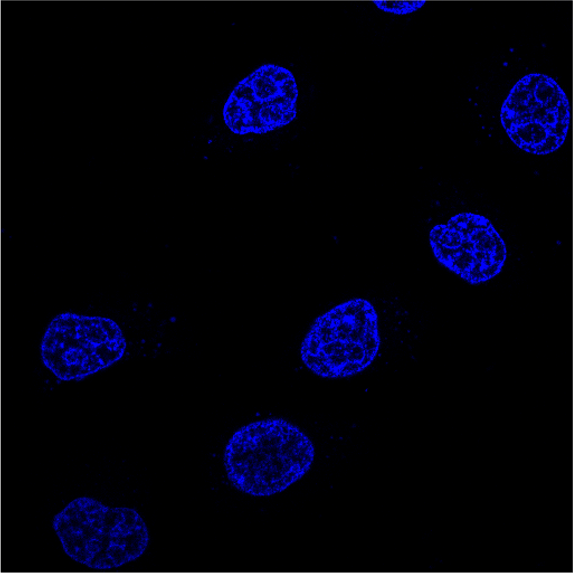

Supplement: Figure 8—source data 2. [file elife-69916-fig8-data2.zip › Figure 8_Source data_Supplementary/Figure S8_Image_EndoG colocalization_mitotracker green/Figure S4B_Representative image_EndoG and localization_HeLa_mitotracker green_nuclear stain.Tif]

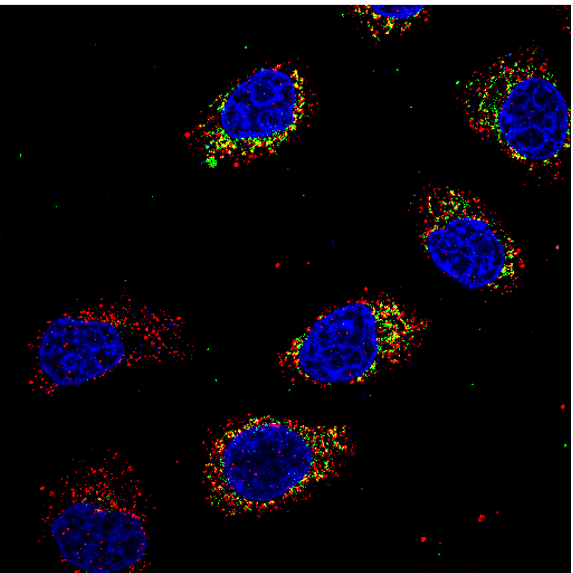

Supplement: Figure 8—source data 2. [file elife-69916-fig8-data2.zip › Figure 8_Source data_Supplementary/Figure S8_Image_EndoG colocalization_mitotracker green/Figure S4B_Representative image_EndoG and localization_HeLa_mitotracker green_merged.Tif]

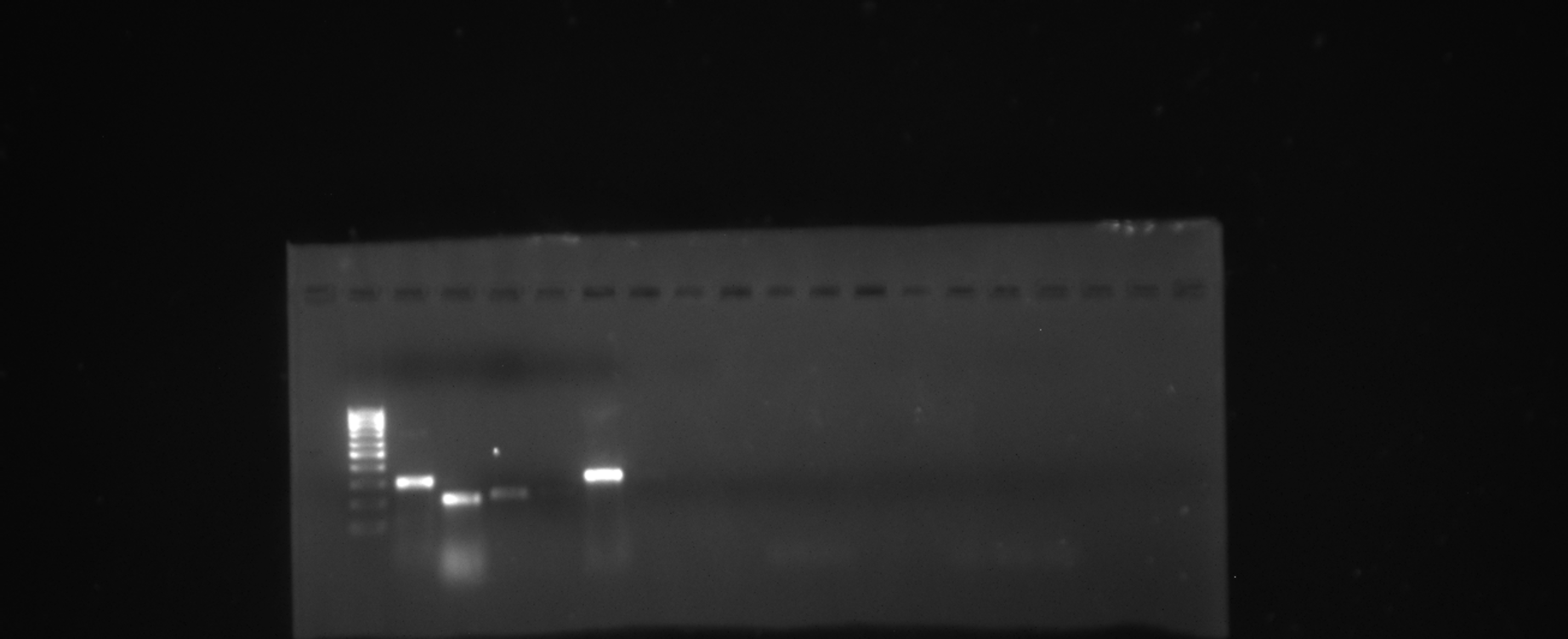

Supplement: Figure 9—source data 1. [file elife-69916-fig9-data1.zip › Figure9_Sourcedata_Binding of EndoG to mitochondria/Figure 9D_mCHIP_mitoextract with mito DNA_EndoG pull down/Figure 4D_m-ChIP of mito extract with mtDNA_EndoG pulldown.Tif]

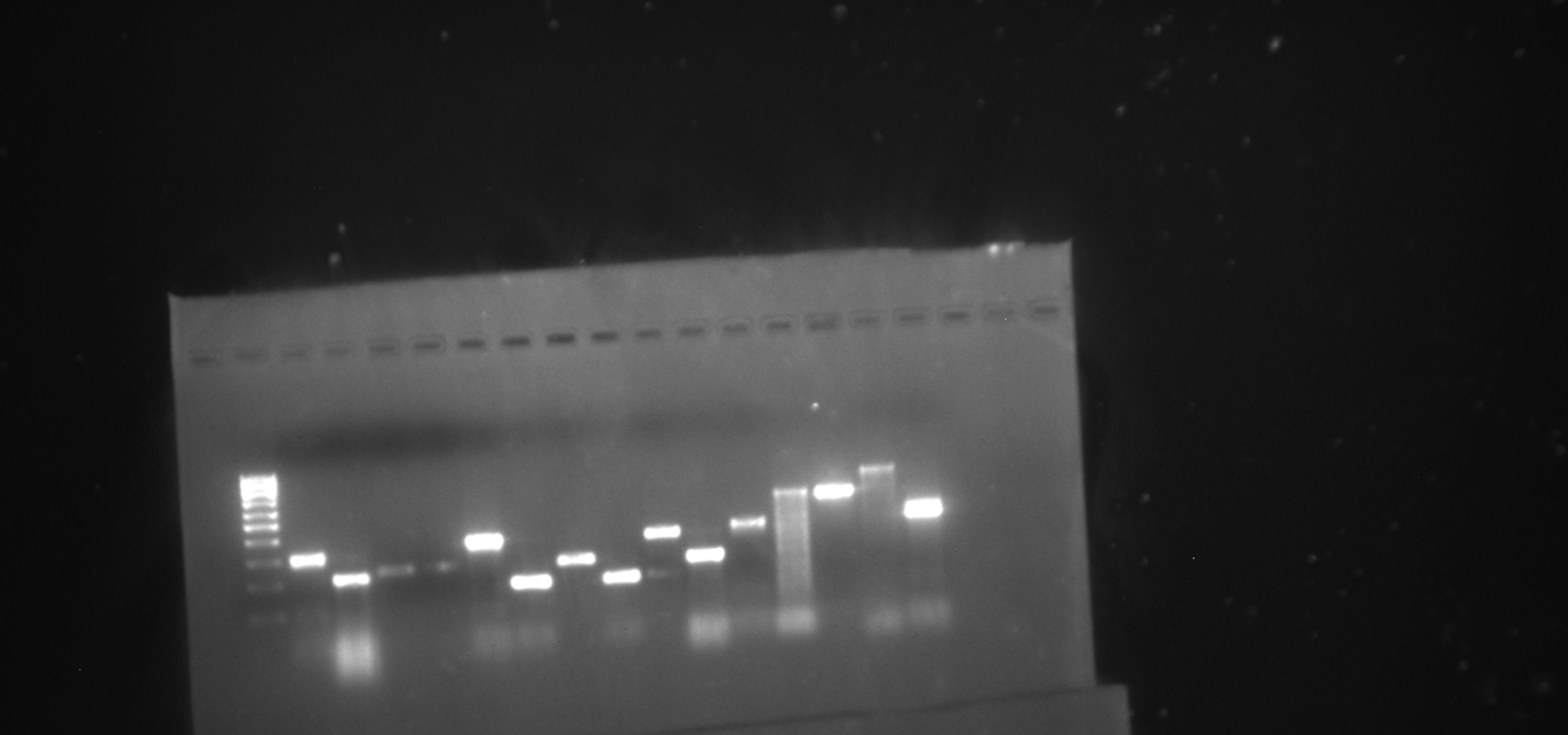

Supplement: Figure 9—source data 1. [file elife-69916-fig9-data1.zip › Figure9_Sourcedata_Binding of EndoG to mitochondria/Figure 9D_mCHIP_mitoextract with mito DNA_EndoG pull down/Figure 4D_m-ChIP of mito extract with mtDNA_Input.Tif]

## Slide 1
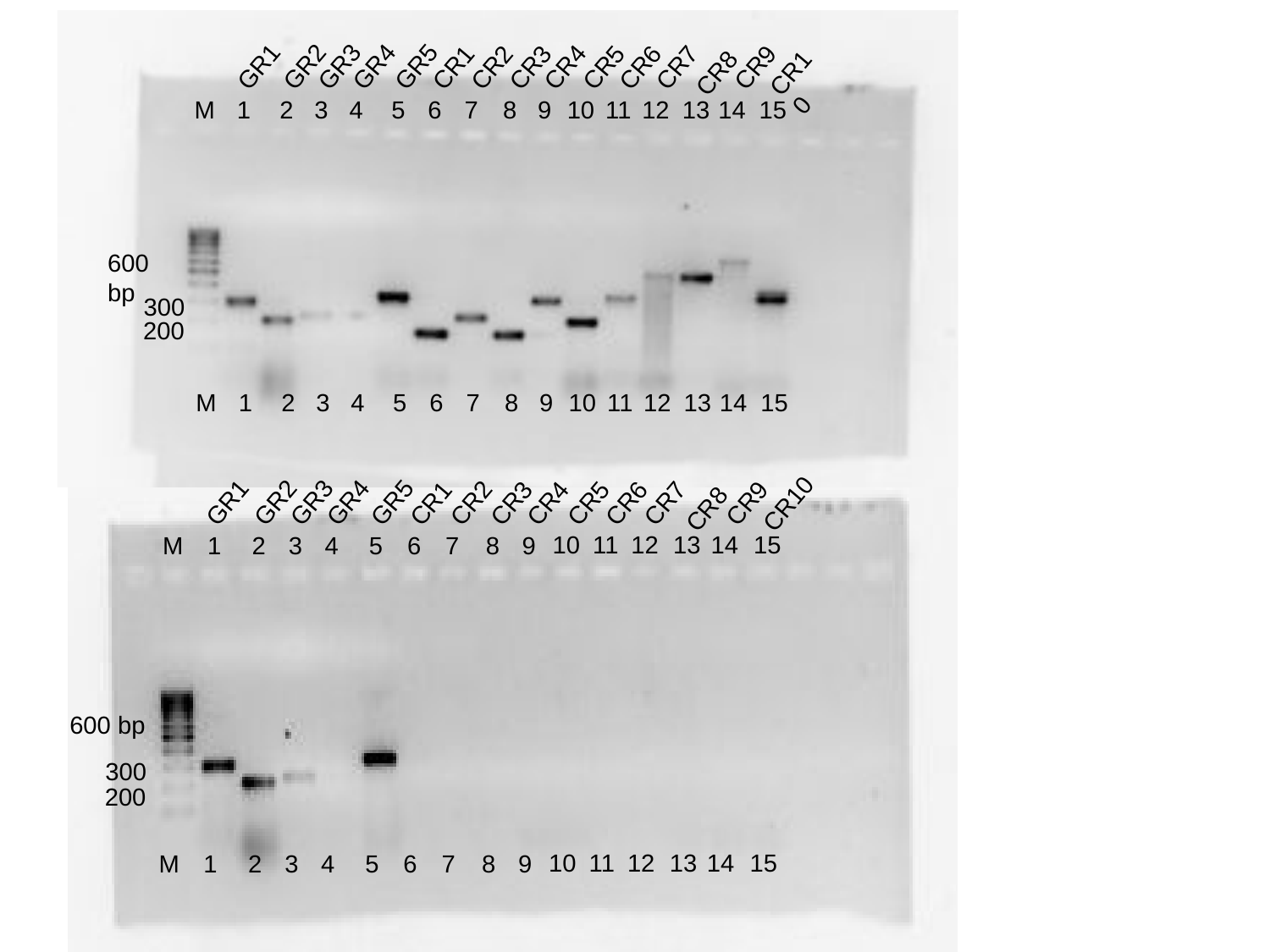

GR1
GR2
GR3
GR4
GR5
CR1
CR2
CR3
CR4
CR5
CR6
CR7
CR9
CR10
CR8
10
11
12
13
14
15
M
1
2
3
4
5
6
7
8
9
600 bp
300
200
10
11
12
13
14
15
M
1
2
3
4
5
6
7
8
9
GR1
GR2
GR3
GR4
GR5
CR1
CR2
CR3
CR4
CR5
CR6
CR7
CR9
CR10
CR8
10
11
12
13
14
15
M
1
2
3
4
5
6
7
8
9
10
11
12
13
14
15
M
1
2
3
4
5
6
7
8
9
600 bp
300
200

Supplement: Figure 9—source data 1. [file elife-69916-fig9-data1.zip › Figure9_Sourcedata_Binding of EndoG to mitochondria/Figure 9D_mCHIP_mitoextract with mito DNA_EndoG pull down/Figure 4D_m-ChIP of mito extract.pptx]

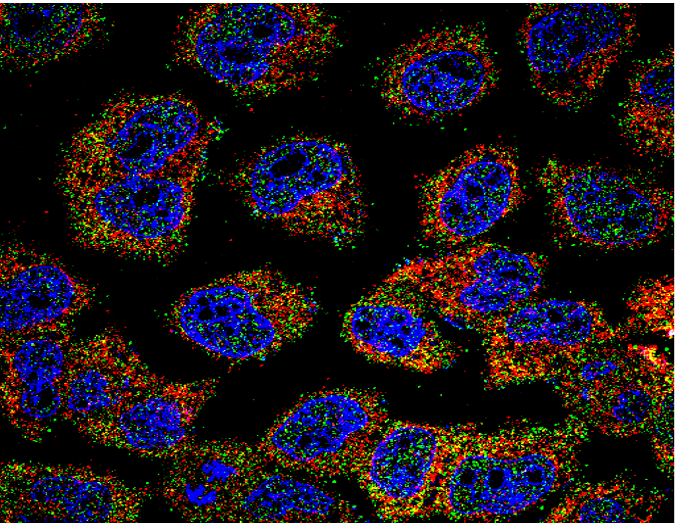

Supplement: Figure 9—source data 1. [file elife-69916-fig9-data1.zip › Figure9_Sourcedata_Binding of EndoG to mitochondria/Figure 9A_Image_BG4 and EndoG localization/Figure 4A_Representative image_EndoG and BG4 coIF_merged.tif]

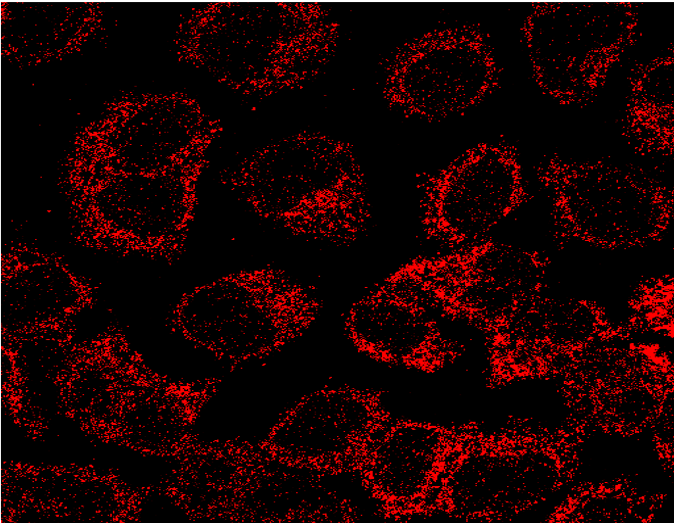

Supplement: Figure 9—source data 1. [file elife-69916-fig9-data1.zip › Figure9_Sourcedata_Binding of EndoG to mitochondria/Figure 9A_Image_BG4 and EndoG localization/Figure 4A_Representative image_EndoG and BG4 coIF_EndoG.tif]

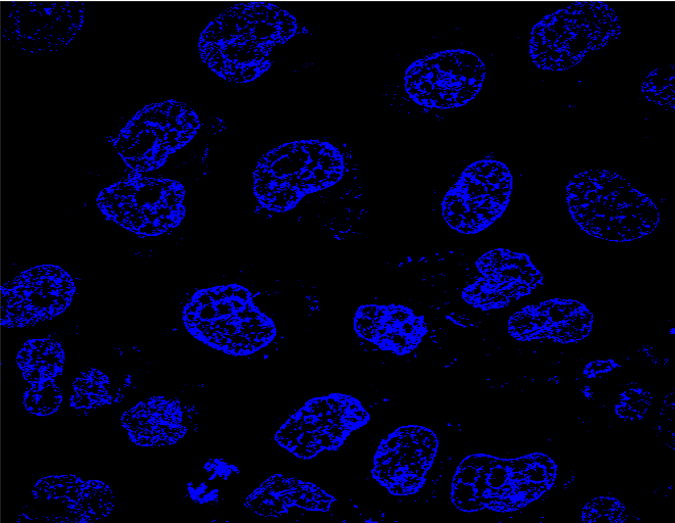

Supplement: Figure 9—source data 1. [file elife-69916-fig9-data1.zip › Figure9_Sourcedata_Binding of EndoG to mitochondria/Figure 9A_Image_BG4 and EndoG localization/Figure 4A_Representative image_EndoG and BG4 coIF_DAPI.tif]

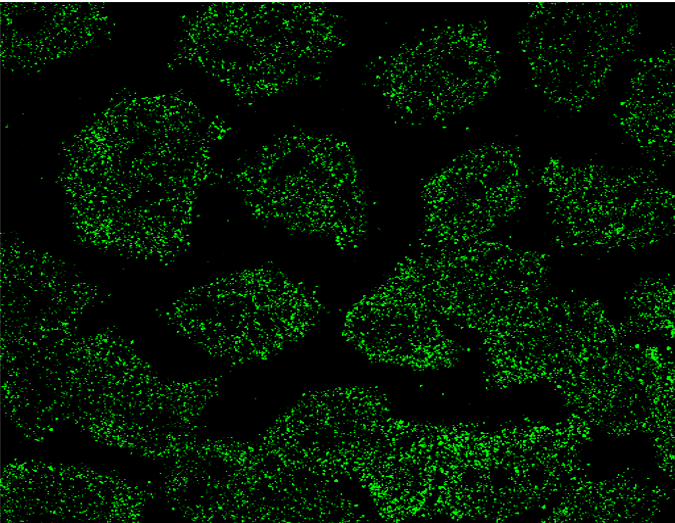

Supplement: Figure 9—source data 1. [file elife-69916-fig9-data1.zip › Figure9_Sourcedata_Binding of EndoG to mitochondria/Figure 9A_Image_BG4 and EndoG localization/Figure 4A_Representative image_EndoG and BG4 coIF_BG4.tif]

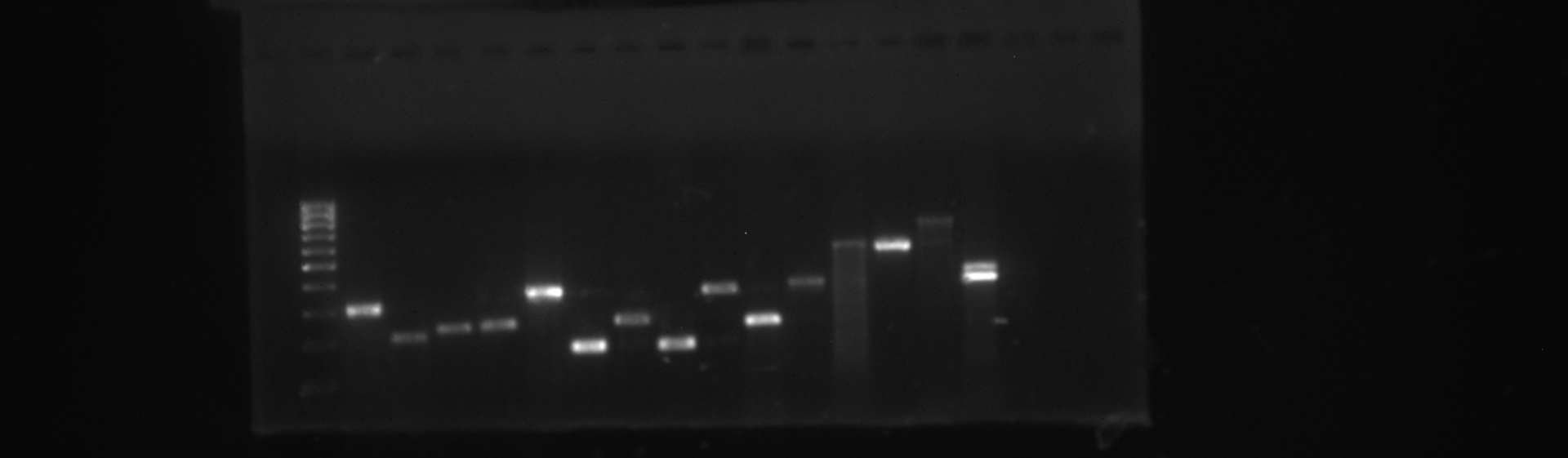

Supplement: Figure 9—source data 2. [file elife-69916-fig9-data2.zip › Figure 9_Source data_Supplementary/Figure S9D_Gel profile for Input and endoG pulldown_mitoChIP/Figure S5D_Gel profile_Input_MitoChIP.Tif]

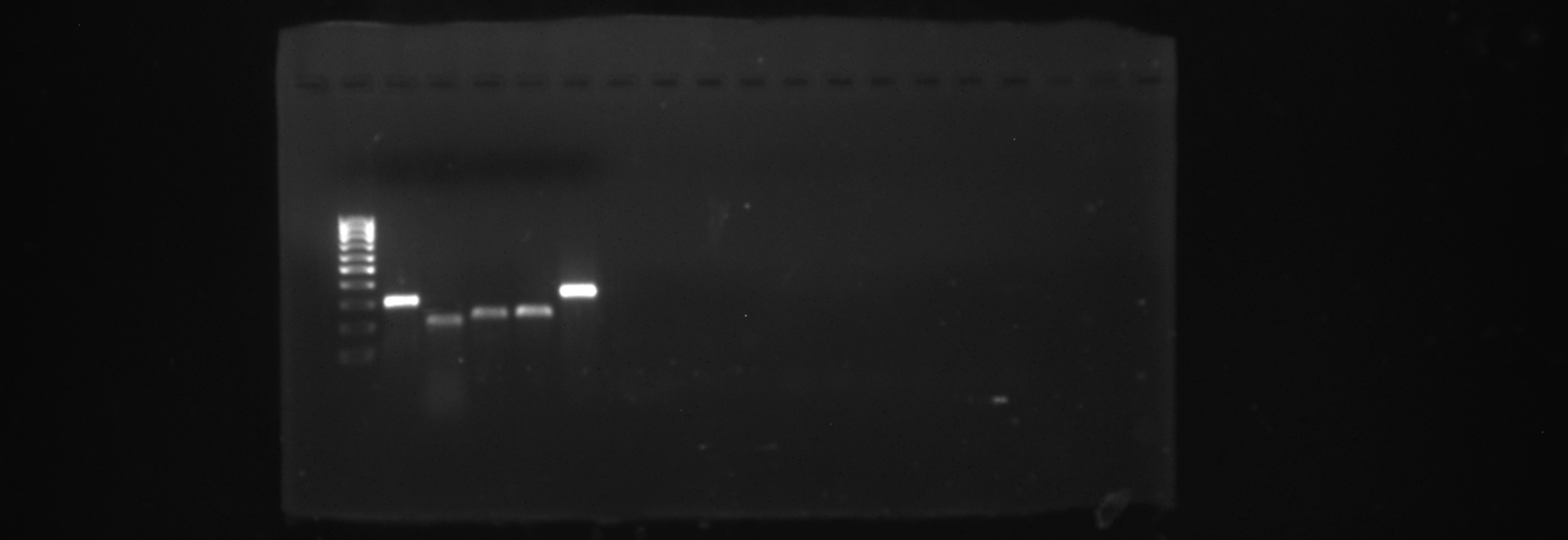

Supplement: Figure 9—source data 2. [file elife-69916-fig9-data2.zip › Figure 9_Source data_Supplementary/Figure S9D_Gel profile for Input and endoG pulldown_mitoChIP/Figure S5D_Gel profile_mitoChIP_EndoG pull down.Tif]

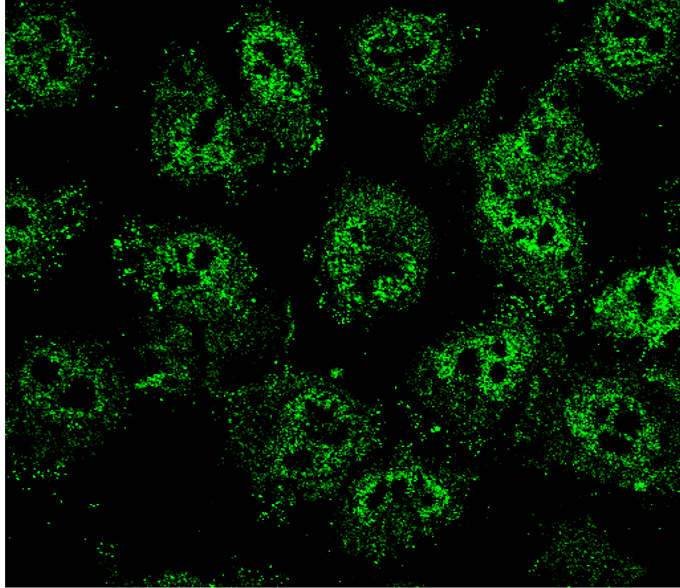

Supplement: Figure 9—source data 2. [file elife-69916-fig9-data2.zip › Figure 9_Source data_Supplementary/Figure S9A_Colocalization of EndoG and BG4/Figure S5A_Representative image_BG4 and EndoG localization_BG4 (1).Tif]

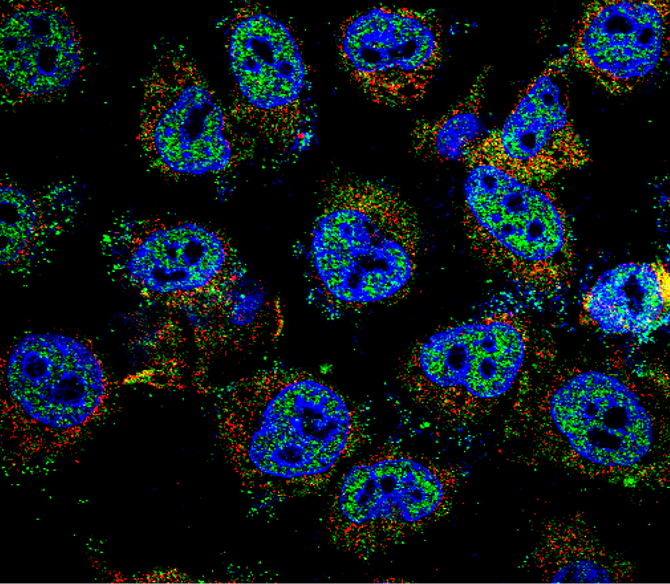

Supplement: Figure 9—source data 2. [file elife-69916-fig9-data2.zip › Figure 9_Source data_Supplementary/Figure S9A_Colocalization of EndoG and BG4/Figure S5A_Representative image_BG4 and EndoG localization_BG4 (4).Tif]

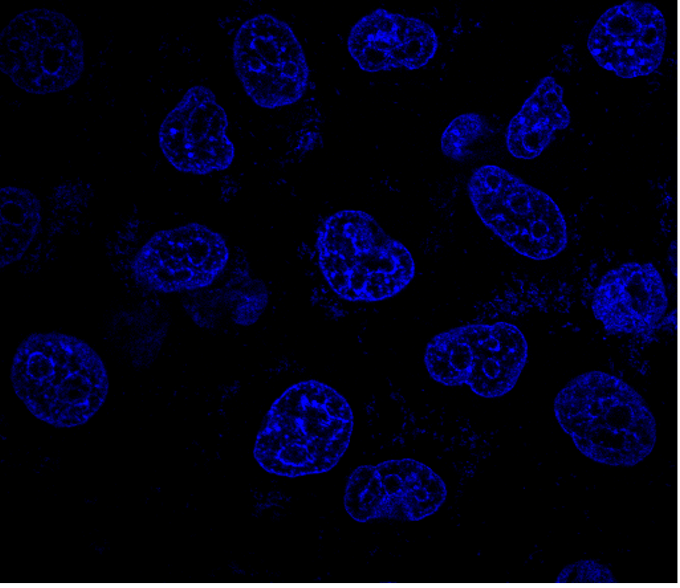

Supplement: Figure 9—source data 2. [file elife-69916-fig9-data2.zip › Figure 9_Source data_Supplementary/Figure S9A_Colocalization of EndoG and BG4/Figure S5A_Representative image_BG4 and EndoG localization_BG4 (2).Tif]

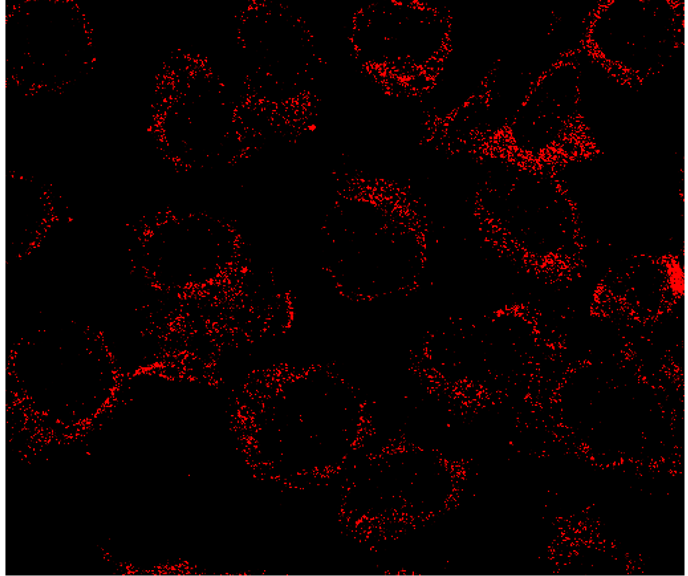

Supplement: Figure 9—source data 2. [file elife-69916-fig9-data2.zip › Figure 9_Source data_Supplementary/Figure S9A_Colocalization of EndoG and BG4/Figure S5A_Representative image_BG4 and EndoG localization_BG4 (3).Tif]

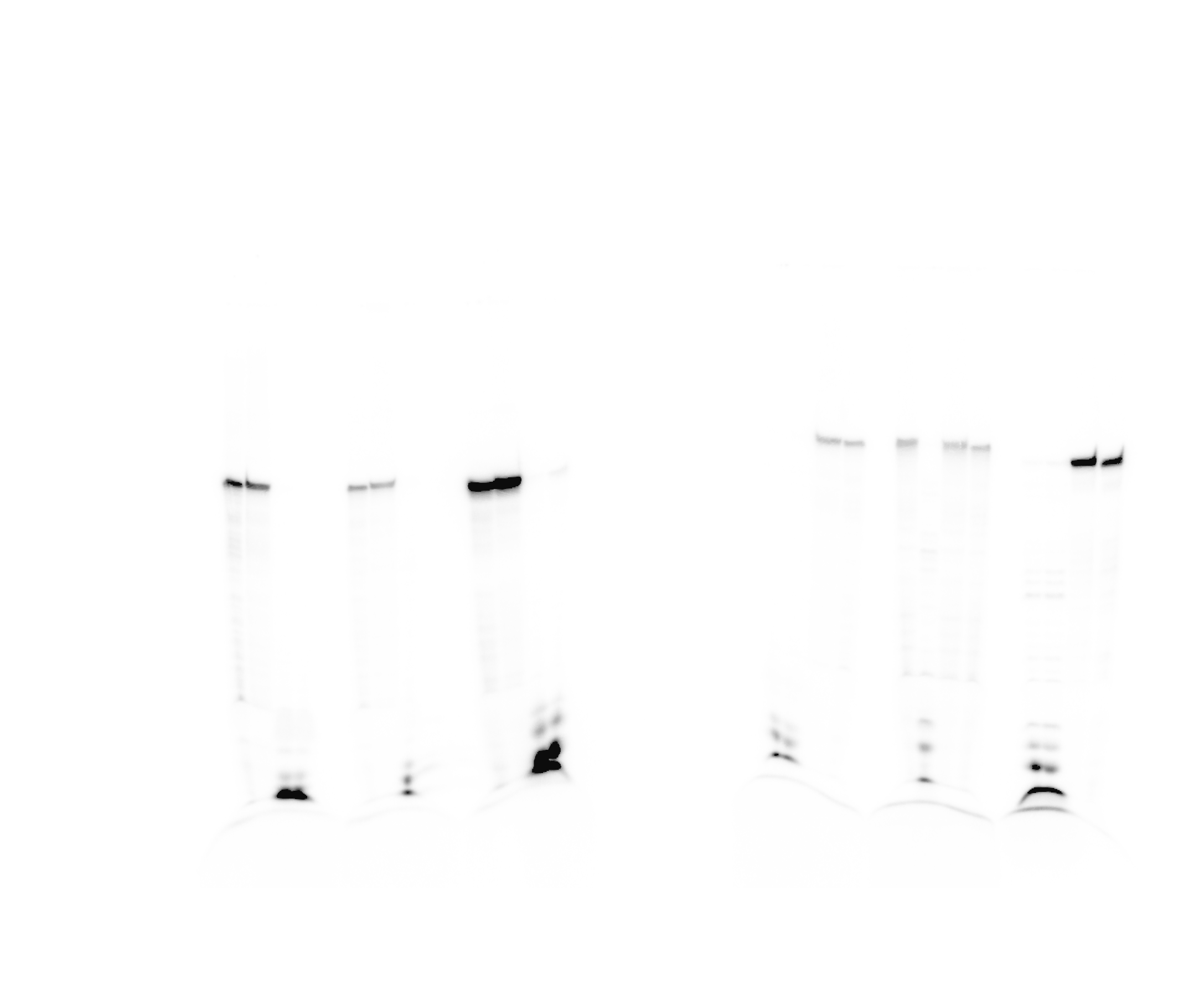

Supplement: Figure 9—source data 3. [file elife-69916-fig9-data3.zip › Figure 10_Sourcedata_Supplementary/Figure S10B_Gel profile_P1 nuclease assay/Figure S5G_Source file.tif]

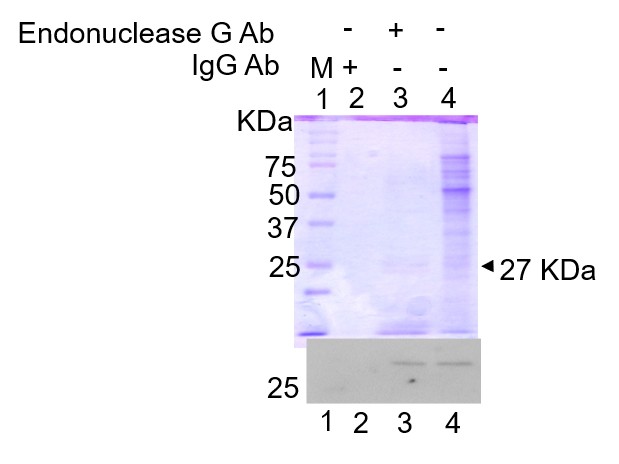

Supplement: Figure 9—source data 3. [file elife-69916-fig9-data3.zip › Figure 10_Sourcedata_Supplementary/Figure S10A_Gel profile after ChIP/FIgure S5F_Western Gel profile after ChIP.tif]

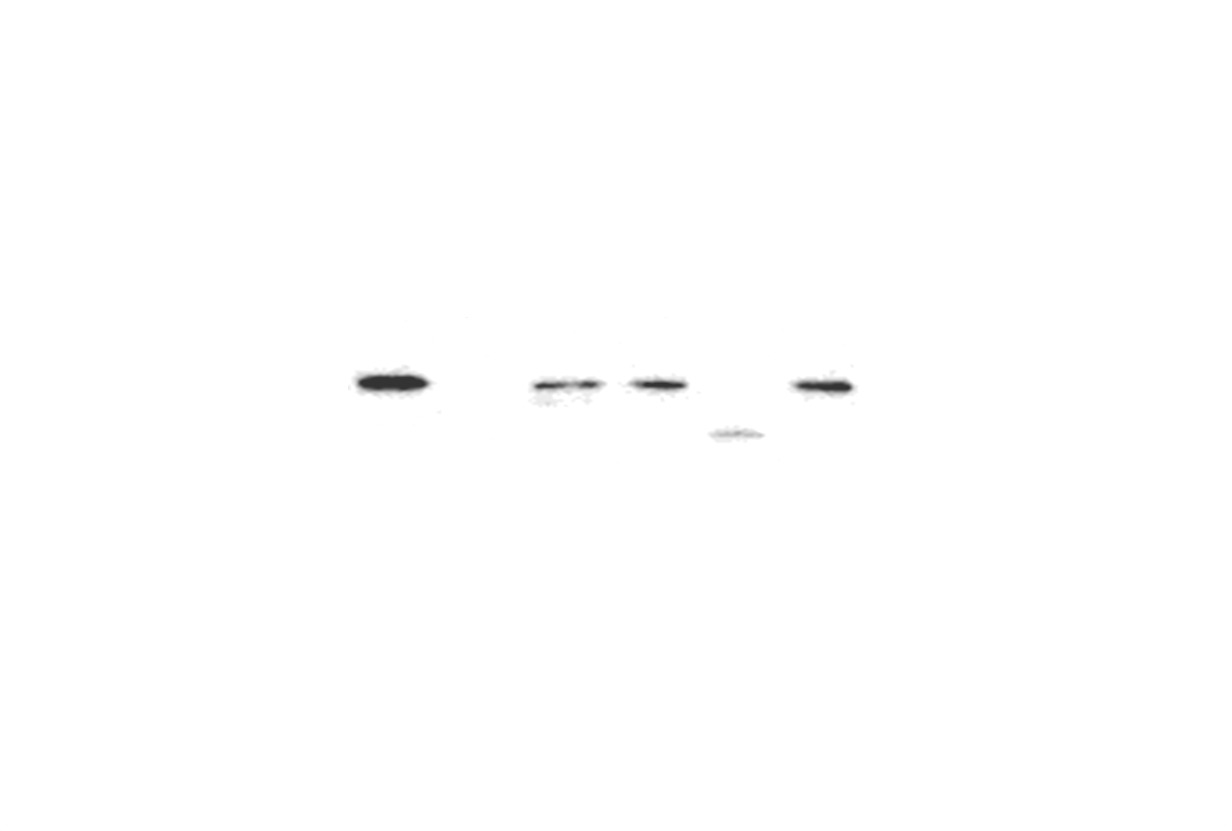

Supplement: Figure 10—source data 1. [file elife-69916-fig10-data1.zip › Figure10_Sourcedata_stress mediated sublocalization of EndoG/Figure 10A_Western blotting_after subfractionation_stress condition/Figure 10A_Representative gel_Cytochrome C localization_Menadione treatment.tif]

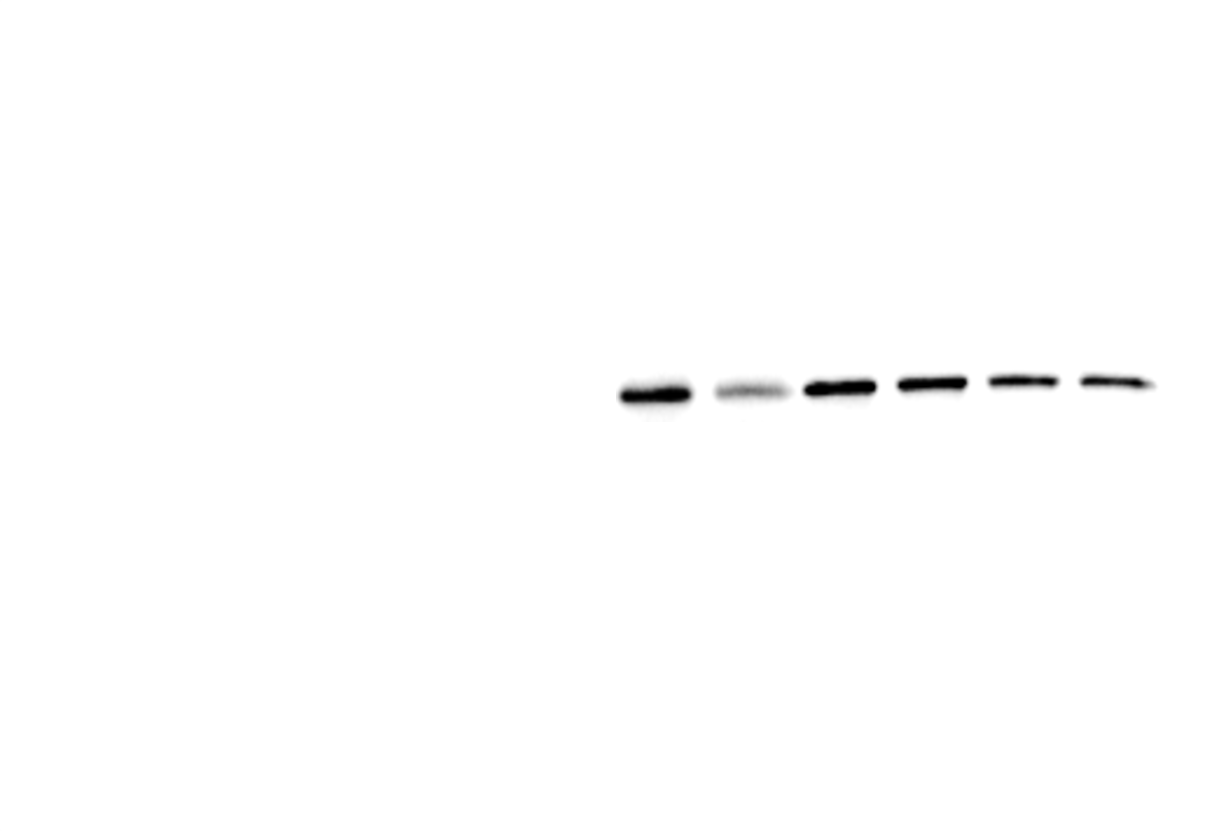

Supplement: Figure 10—source data 1. [file elife-69916-fig10-data1.zip › Figure10_Sourcedata_stress mediated sublocalization of EndoG/Figure 10A_Western blotting_after subfractionation_stress condition/Figure 10A_Representative gel_Endo G localization_Menadion treatment.tif]

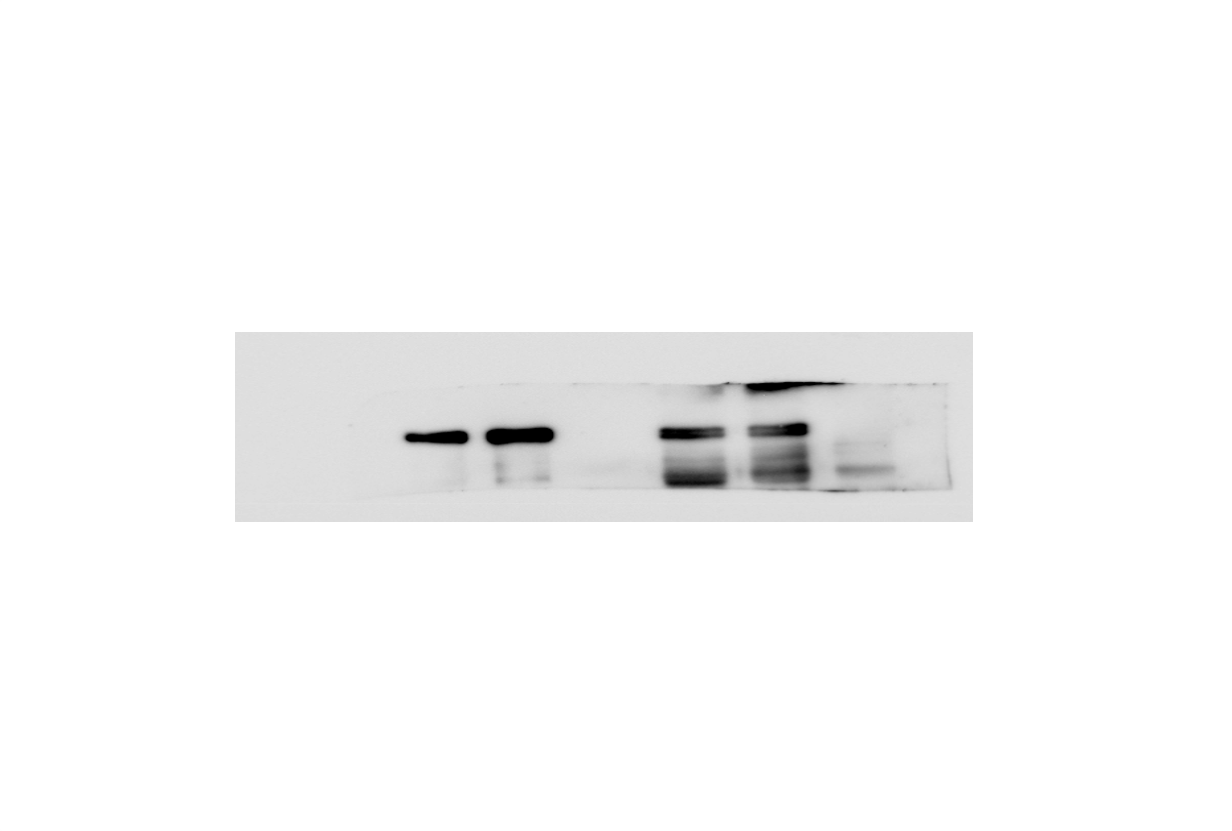

Supplement: Figure 10—source data 1. [file elife-69916-fig10-data1.zip › Figure10_Sourcedata_stress mediated sublocalization of EndoG/Figure 10A_Western blotting_after subfractionation_stress condition/Figure 10A_Representative gel_TFAM localization_Menadione treatment.tif]
