# Supplementary figures and images for: Unleashing a novel function of Endonuclease G in mitochondrial genome instability (part 3 of 4)
Source: eLife. 2022 Nov 17;11:e69916. doi: 10.7554/eLife.69916 (PMC9711528; doi:10.7554/eLife.69916)

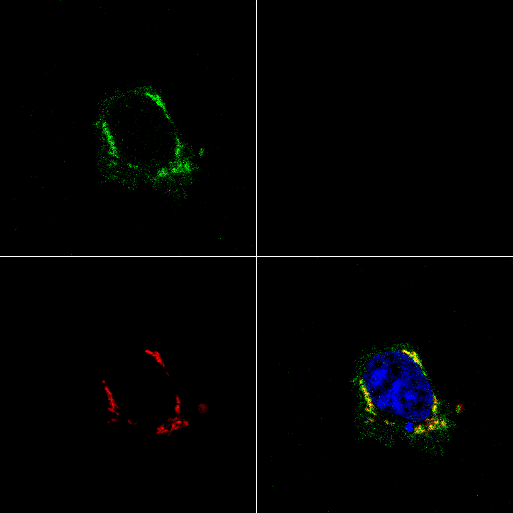

Supplement: Figure 8—source data 1. [file elife-69916-fig8-data1.zip › Figure8_Sourcedata_localization of Endonuclease G/Figure 8A_Representative images_localization of EndoG to mitochondria/Figure 8A_Source file_1_MEF/1.tif.frames(27).tif]

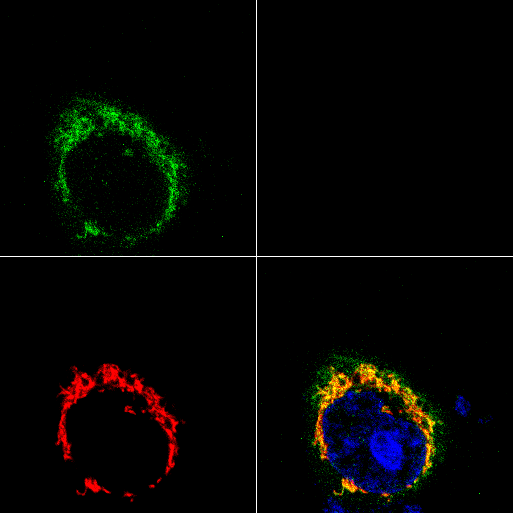

Supplement: Figure 8—source data 1. [file elife-69916-fig8-data1.zip › Figure8_Sourcedata_localization of Endonuclease G/Figure 8A_Representative images_localization of EndoG to mitochondria/Figure 8A_Source file_1_MEF/1.tif.frames.tif]

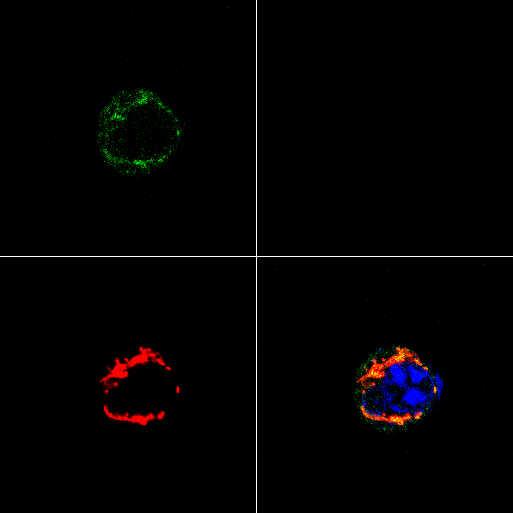

Supplement: Figure 8—source data 1. [file elife-69916-fig8-data1.zip › Figure8_Sourcedata_localization of Endonuclease G/Figure 8A_Representative images_localization of EndoG to mitochondria/Figure 8A_Source file_1_MEF/1.tif.frames(11).tif]

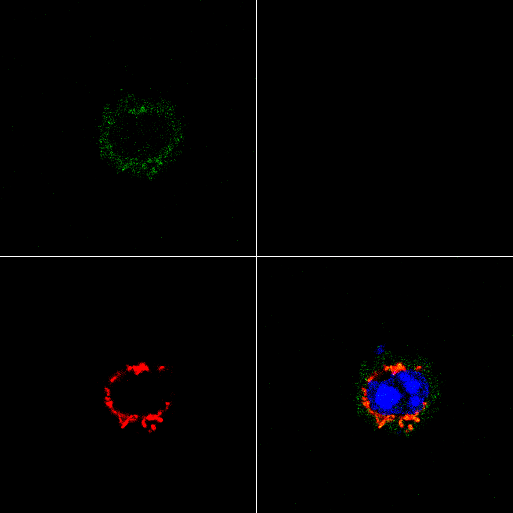

Supplement: Figure 8—source data 1. [file elife-69916-fig8-data1.zip › Figure8_Sourcedata_localization of Endonuclease G/Figure 8A_Representative images_localization of EndoG to mitochondria/Figure 8A_Source file_1_MEF/1.tif.frames(10).tif]

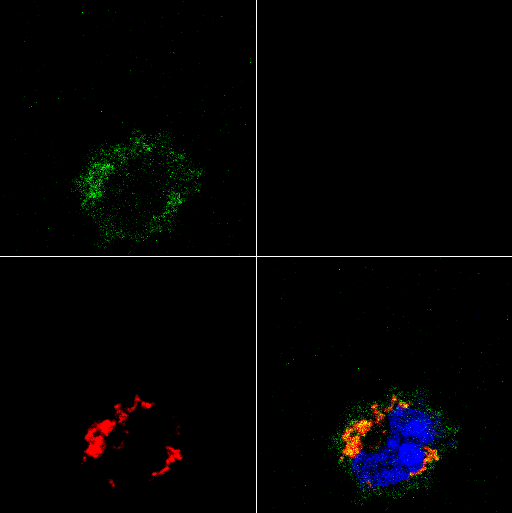

Supplement: Figure 8—source data 1. [file elife-69916-fig8-data1.zip › Figure8_Sourcedata_localization of Endonuclease G/Figure 8A_Representative images_localization of EndoG to mitochondria/Figure 8A_Source file_1_MEF/1.tif.frames(30).tif]

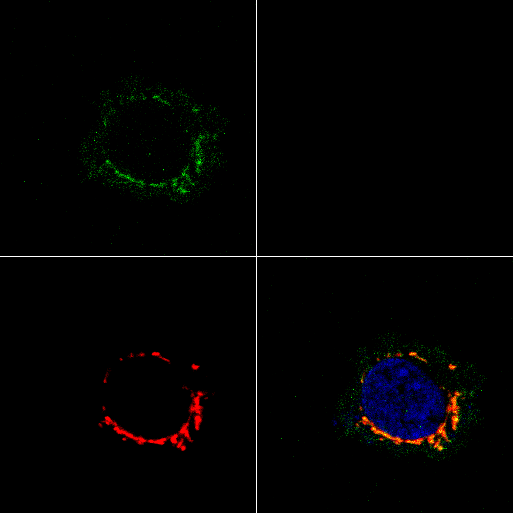

Supplement: Figure 8—source data 1. [file elife-69916-fig8-data1.zip › Figure8_Sourcedata_localization of Endonuclease G/Figure 8A_Representative images_localization of EndoG to mitochondria/Figure 8A_Source file_1_MEF/1.tif.frames(26).tif]

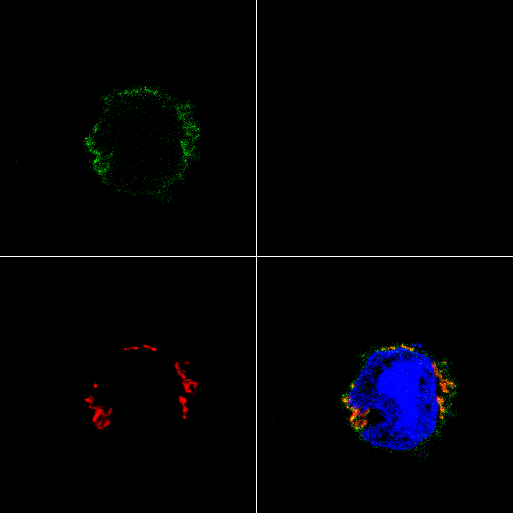

Supplement: Figure 8—source data 1. [file elife-69916-fig8-data1.zip › Figure8_Sourcedata_localization of Endonuclease G/Figure 8A_Representative images_localization of EndoG to mitochondria/Figure 8A_Source file_1_MEF/1.tif.frames(17).tif]

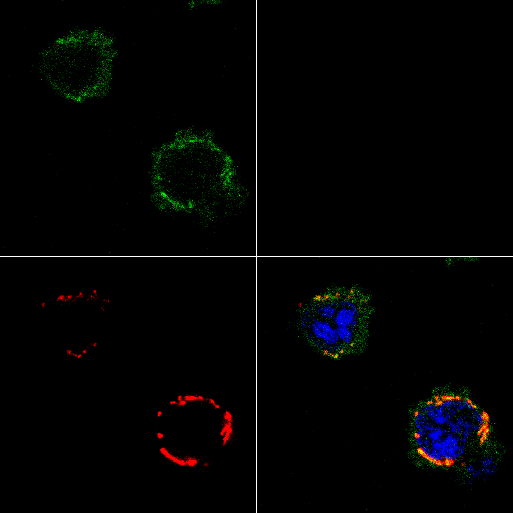

Supplement: Figure 8—source data 1. [file elife-69916-fig8-data1.zip › Figure8_Sourcedata_localization of Endonuclease G/Figure 8A_Representative images_localization of EndoG to mitochondria/Figure 8A_Source file_1_MEF/1.tif.frames(7).tif]

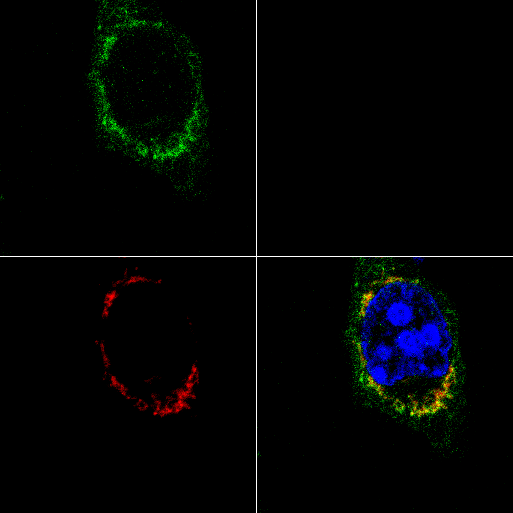

Supplement: Figure 8—source data 1. [file elife-69916-fig8-data1.zip › Figure8_Sourcedata_localization of Endonuclease G/Figure 8A_Representative images_localization of EndoG to mitochondria/Figure 8A_Source file_1_MEF/1.tif.frames(21).tif]

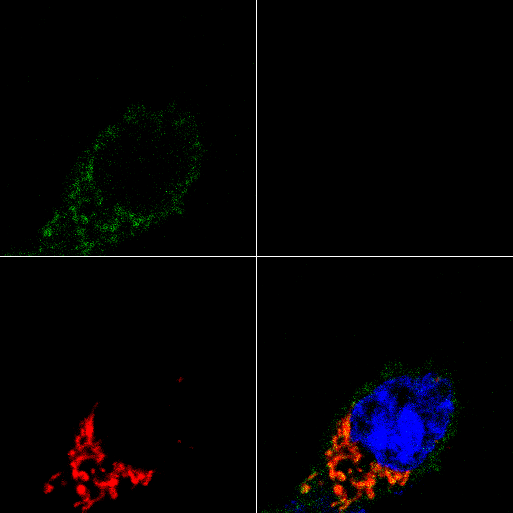

Supplement: Figure 8—source data 1. [file elife-69916-fig8-data1.zip › Figure8_Sourcedata_localization of Endonuclease G/Figure 8A_Representative images_localization of EndoG to mitochondria/Figure 8A_Source file_1_MEF/1.tif.frames(20).tif]

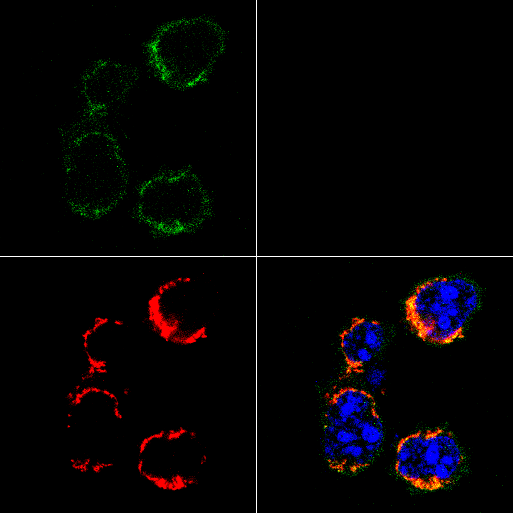

Supplement: Figure 8—source data 1. [file elife-69916-fig8-data1.zip › Figure8_Sourcedata_localization of Endonuclease G/Figure 8A_Representative images_localization of EndoG to mitochondria/Figure 8A_Source file_1_MEF/1.tif.frames(6).tif]

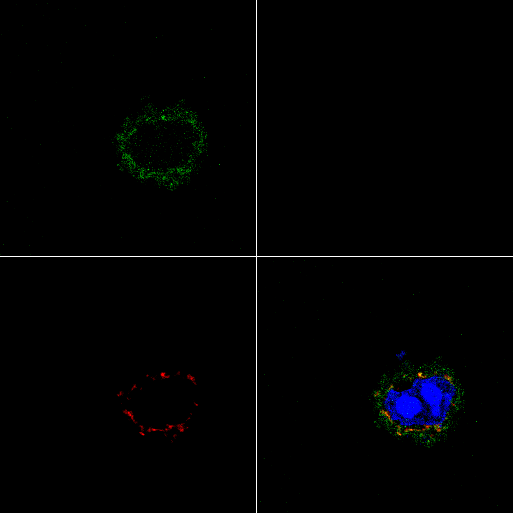

Supplement: Figure 8—source data 1. [file elife-69916-fig8-data1.zip › Figure8_Sourcedata_localization of Endonuclease G/Figure 8A_Representative images_localization of EndoG to mitochondria/Figure 8A_Source file_1_MEF/1.tif.frames(16).tif]

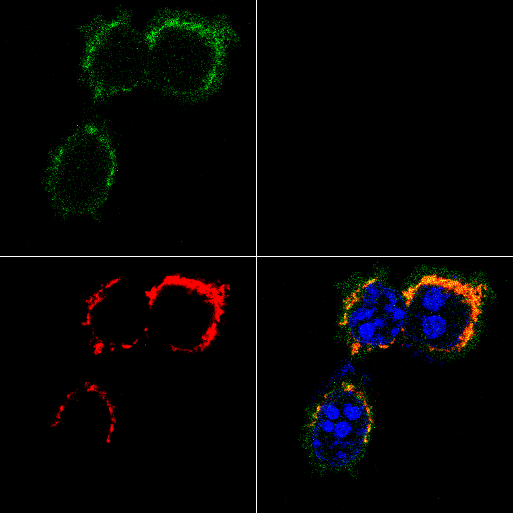

Supplement: Figure 8—source data 1. [file elife-69916-fig8-data1.zip › Figure8_Sourcedata_localization of Endonuclease G/Figure 8A_Representative images_localization of EndoG to mitochondria/Figure 8A_Source file_1_MEF/1.tif.frames(5).tif]

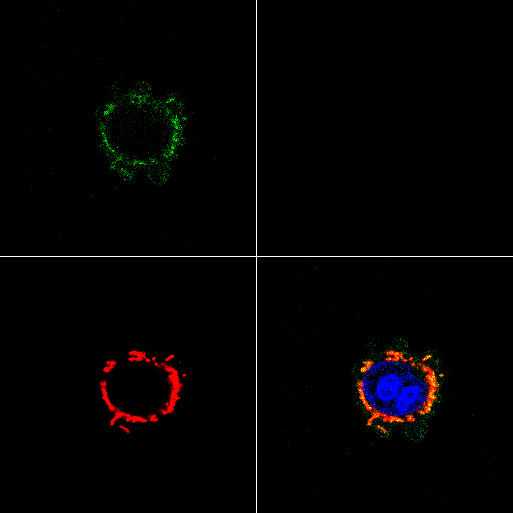

Supplement: Figure 8—source data 1. [file elife-69916-fig8-data1.zip › Figure8_Sourcedata_localization of Endonuclease G/Figure 8A_Representative images_localization of EndoG to mitochondria/Figure 8A_Source file_1_MEF/1.tif.frames(15).tif]

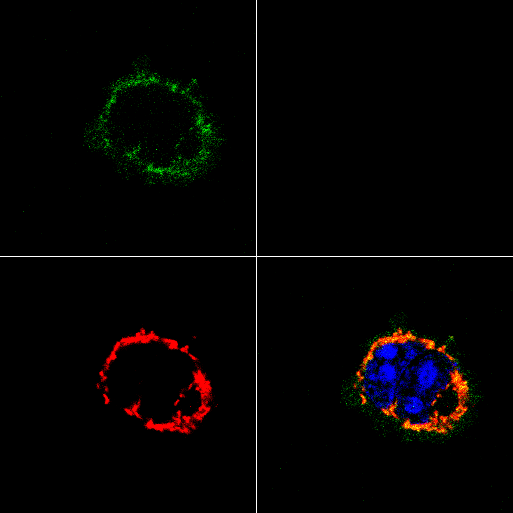

Supplement: Figure 8—source data 1. [file elife-69916-fig8-data1.zip › Figure8_Sourcedata_localization of Endonuclease G/Figure 8A_Representative images_localization of EndoG to mitochondria/Figure 8A_Source file_1_MEF/1.tif.frames(9).tif]

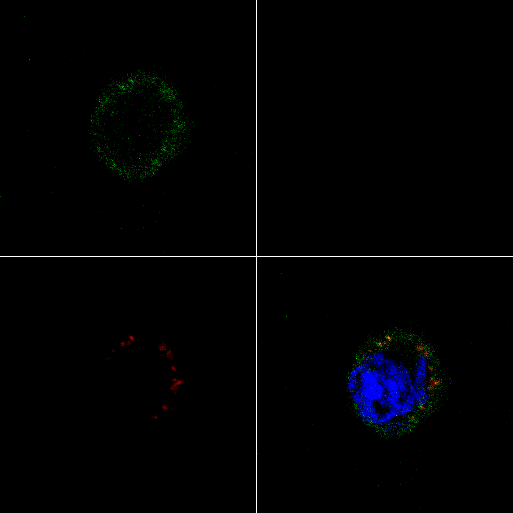

Supplement: Figure 8—source data 1. [file elife-69916-fig8-data1.zip › Figure8_Sourcedata_localization of Endonuclease G/Figure 8A_Representative images_localization of EndoG to mitochondria/Figure 8A_Source file_1_MEF/1.tif.frames(19).tif]

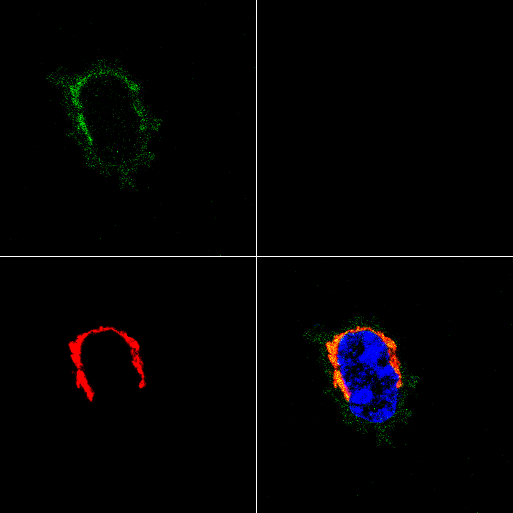

Supplement: Figure 8—source data 1. [file elife-69916-fig8-data1.zip › Figure8_Sourcedata_localization of Endonuclease G/Figure 8A_Representative images_localization of EndoG to mitochondria/Figure 8A_Source file_1_MEF/1.tif.frames(22).tif]

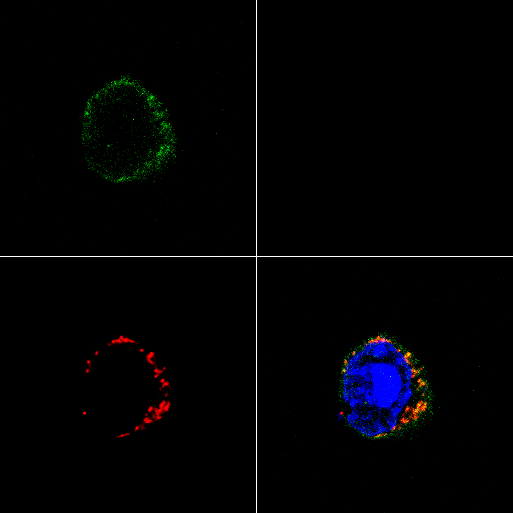

Supplement: Figure 8—source data 1. [file elife-69916-fig8-data1.zip › Figure8_Sourcedata_localization of Endonuclease G/Figure 8A_Representative images_localization of EndoG to mitochondria/Figure 8A_Source file_1_MEF/1.tif.frames(18).tif]

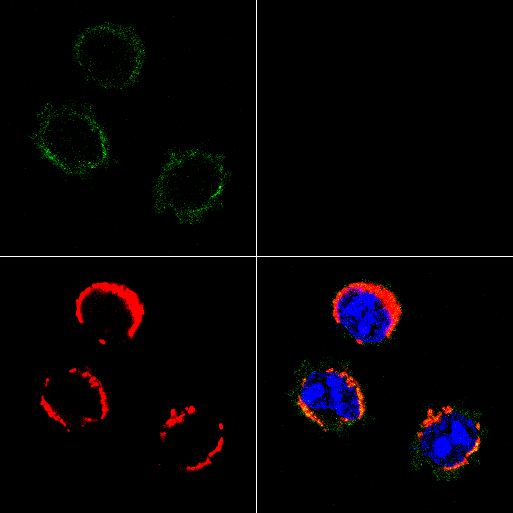

Supplement: Figure 8—source data 1. [file elife-69916-fig8-data1.zip › Figure8_Sourcedata_localization of Endonuclease G/Figure 8A_Representative images_localization of EndoG to mitochondria/Figure 8A_Source file_1_MEF/1.tif.frames(8).tif]

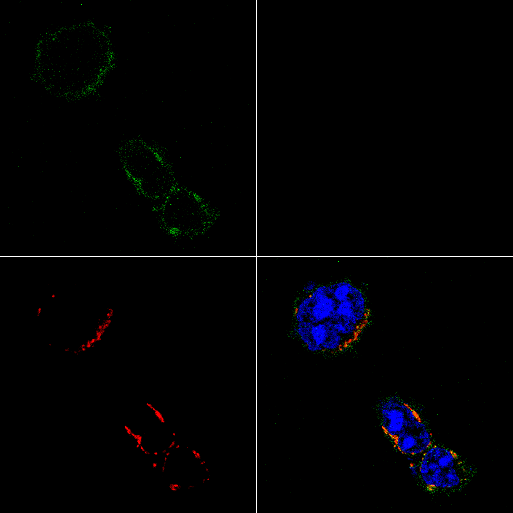

Supplement: Figure 8—source data 1. [file elife-69916-fig8-data1.zip › Figure8_Sourcedata_localization of Endonuclease G/Figure 8A_Representative images_localization of EndoG to mitochondria/Figure 8A_Source file_1_MEF/1.tif.frames(14).tif]

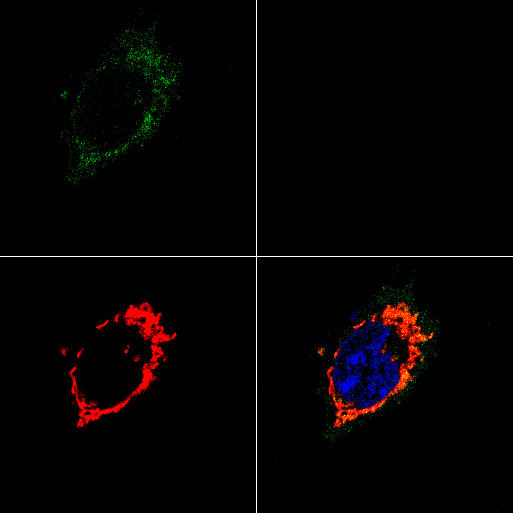

Supplement: Figure 8—source data 1. [file elife-69916-fig8-data1.zip › Figure8_Sourcedata_localization of Endonuclease G/Figure 8A_Representative images_localization of EndoG to mitochondria/Figure 8A_Source file_1_MEF/1.tif.frames(4).tif]

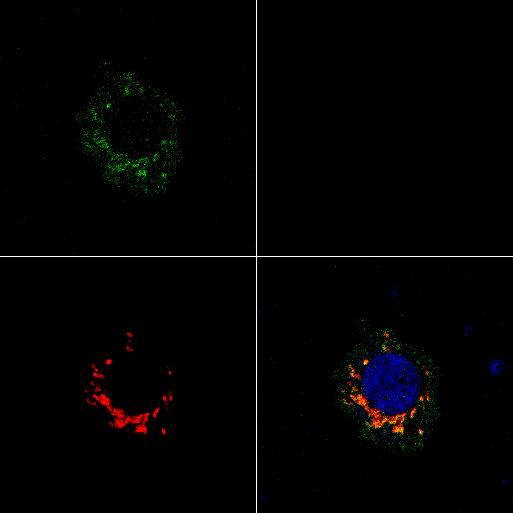

Supplement: Figure 8—source data 1. [file elife-69916-fig8-data1.zip › Figure8_Sourcedata_localization of Endonuclease G/Figure 8A_Representative images_localization of EndoG to mitochondria/Figure 8A_Source file_1_MEF/1.tif.frames(25).tif]

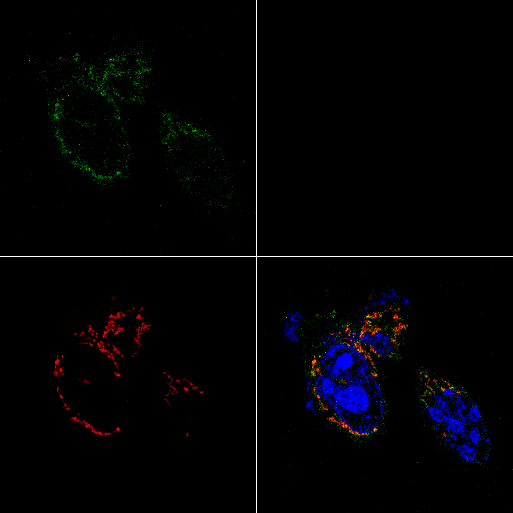

Supplement: Figure 8—source data 1. [file elife-69916-fig8-data1.zip › Figure8_Sourcedata_localization of Endonuclease G/Figure 8A_Representative images_localization of EndoG to mitochondria/Figure 8A_Source file_1_MEF/1.tif.frames(13).tif]

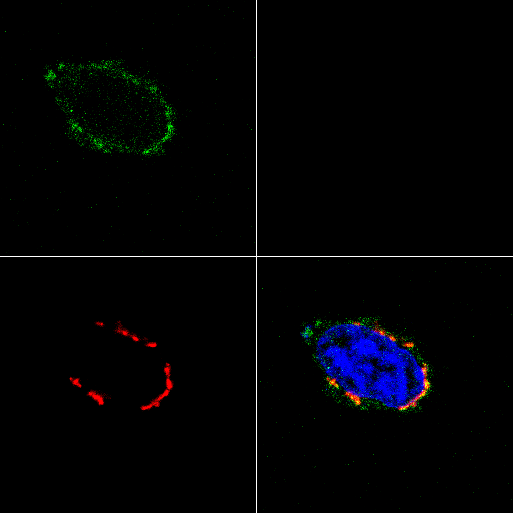

Supplement: Figure 8—source data 1. [file elife-69916-fig8-data1.zip › Figure8_Sourcedata_localization of Endonuclease G/Figure 8A_Representative images_localization of EndoG to mitochondria/Figure 8A_Source file_1_MEF/1.tif.frames(29).tif]

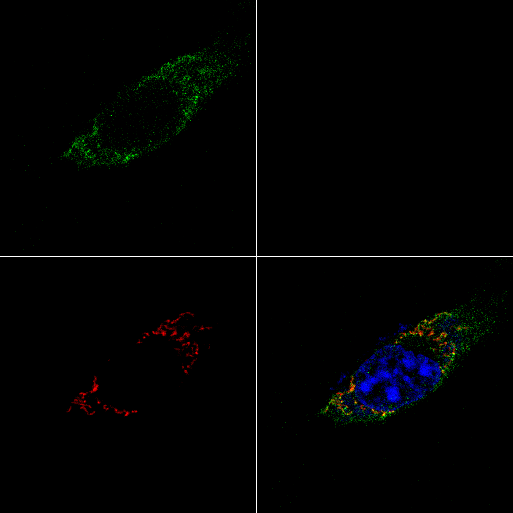

Supplement: Figure 8—source data 1. [file elife-69916-fig8-data1.zip › Figure8_Sourcedata_localization of Endonuclease G/Figure 8A_Representative images_localization of EndoG to mitochondria/Figure 8A_Source file_1_MEF/1.tif.frames(28).tif]

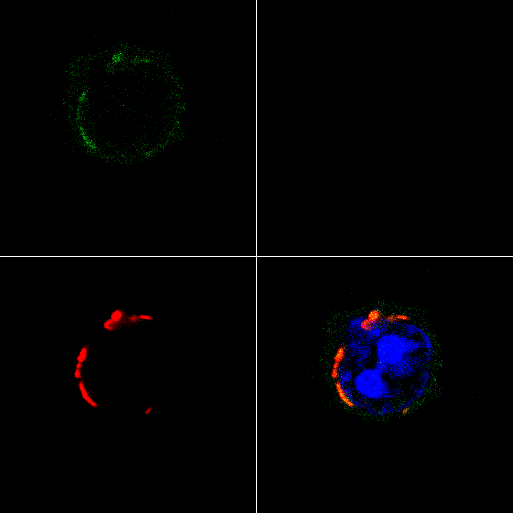

Supplement: Figure 8—source data 1. [file elife-69916-fig8-data1.zip › Figure8_Sourcedata_localization of Endonuclease G/Figure 8A_Representative images_localization of EndoG to mitochondria/Figure 8A_Source file_1_MEF/1.tif.frames(12).tif]

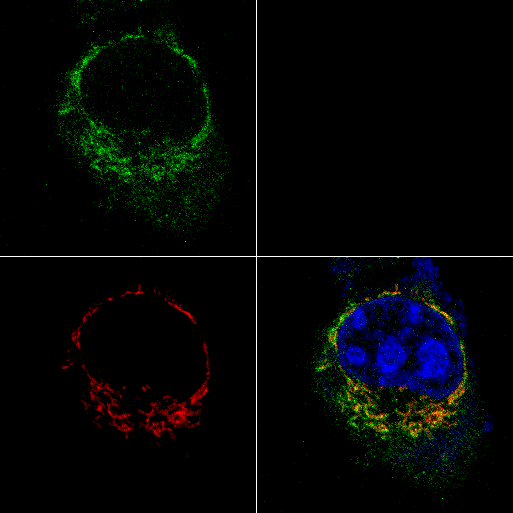

Supplement: Figure 8—source data 1. [file elife-69916-fig8-data1.zip › Figure8_Sourcedata_localization of Endonuclease G/Figure 8A_Representative images_localization of EndoG to mitochondria/Figure 8A_Source file_1_MEF/1.tif.frames(2).tif]

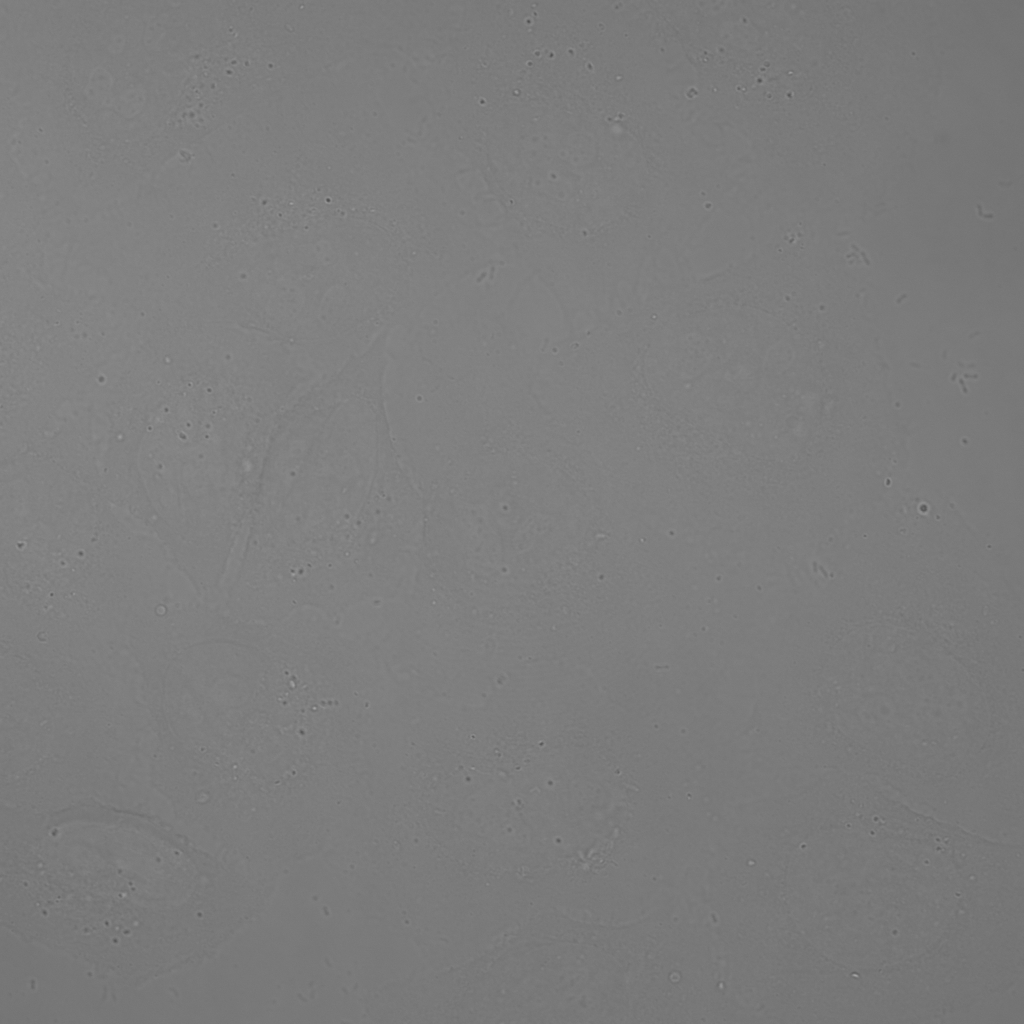

Supplement: Figure 8—source data 1. [file elife-69916-fig8-data1.zip › Figure8_Sourcedata_localization of Endonuclease G/Figure 8A_Representative images_localization of EndoG to mitochondria/Figure 8A_Source file_3-293T/1_0007.tif.frames/1_0007_C004T001.tif]

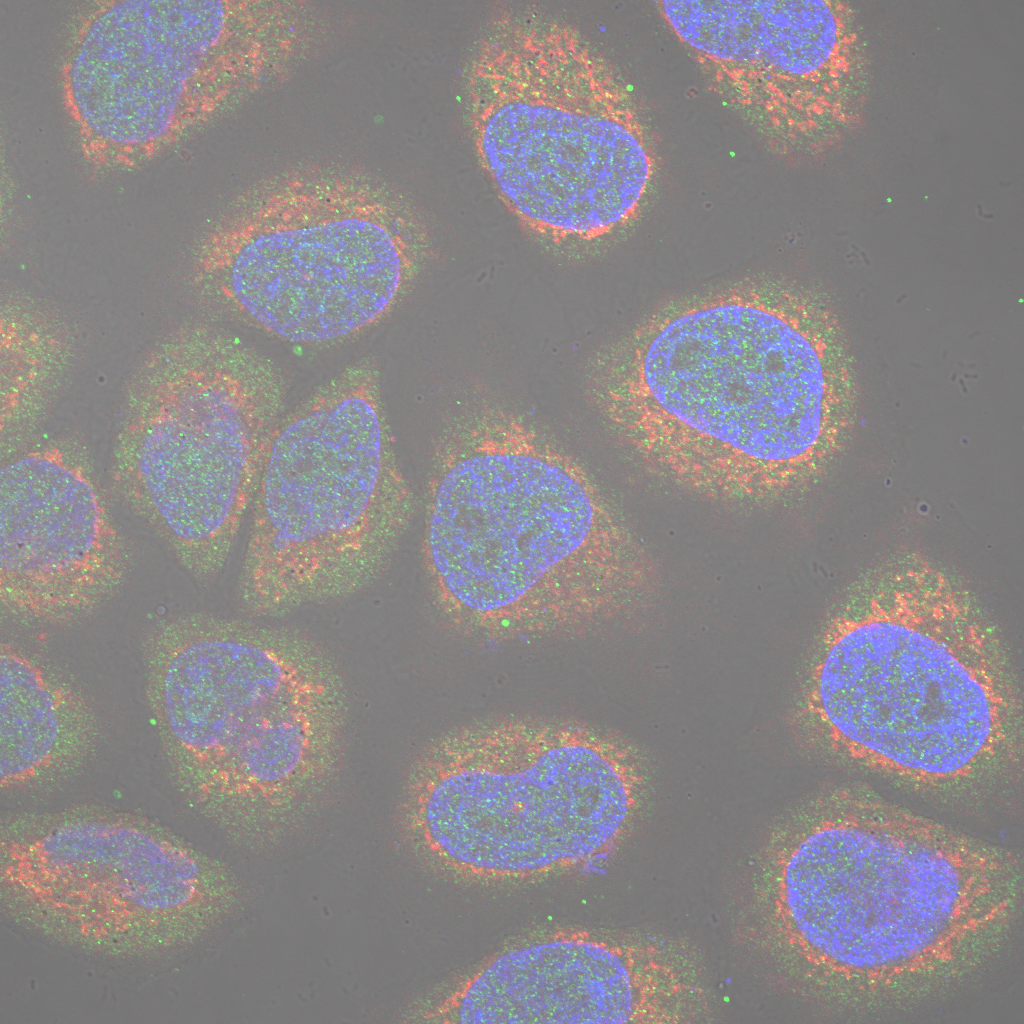

Supplement: Figure 8—source data 1. [file elife-69916-fig8-data1.zip › Figure8_Sourcedata_localization of Endonuclease G/Figure 8A_Representative images_localization of EndoG to mitochondria/Figure 8A_Source file_3-293T/1_0007.tif.frames/1_0007_T001.tif]

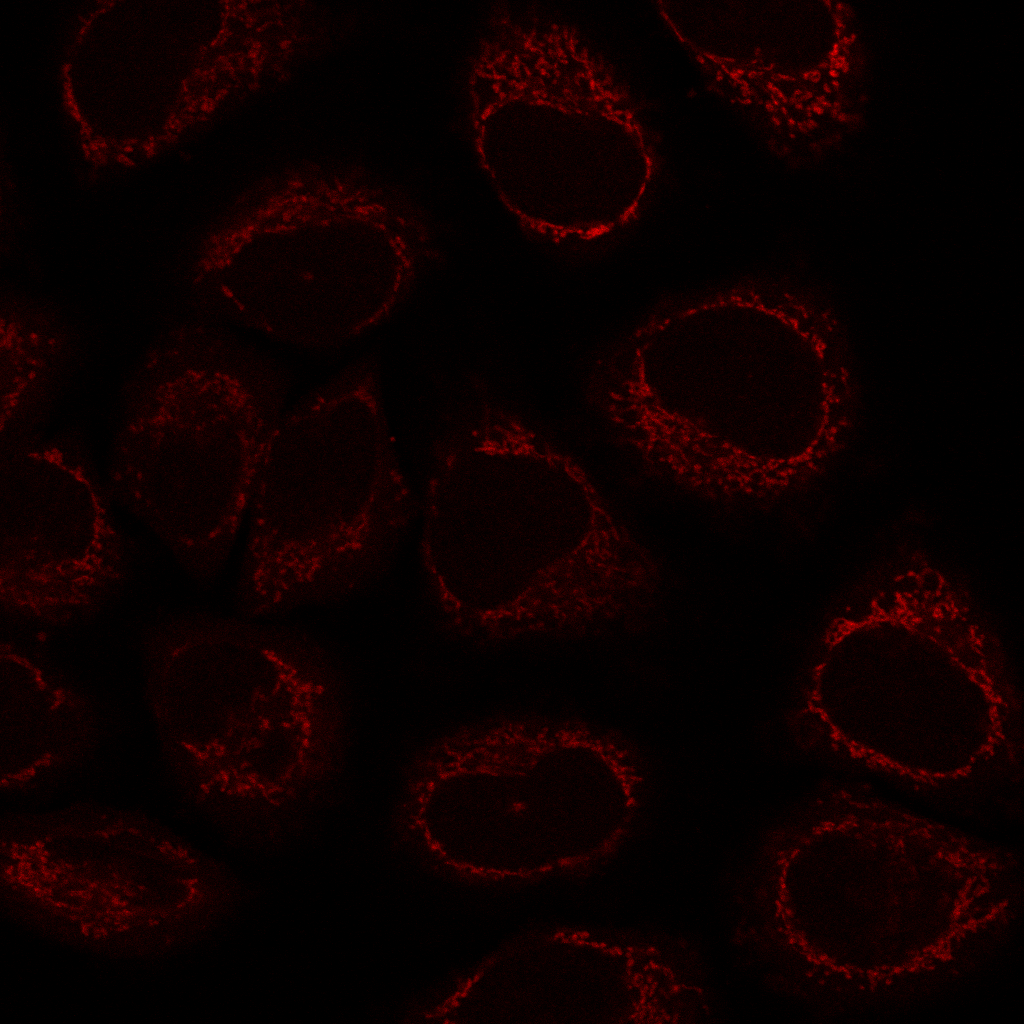

Supplement: Figure 8—source data 1. [file elife-69916-fig8-data1.zip › Figure8_Sourcedata_localization of Endonuclease G/Figure 8A_Representative images_localization of EndoG to mitochondria/Figure 8A_Source file_3-293T/1_0007.tif.frames/1_0007_C002T001.tif]

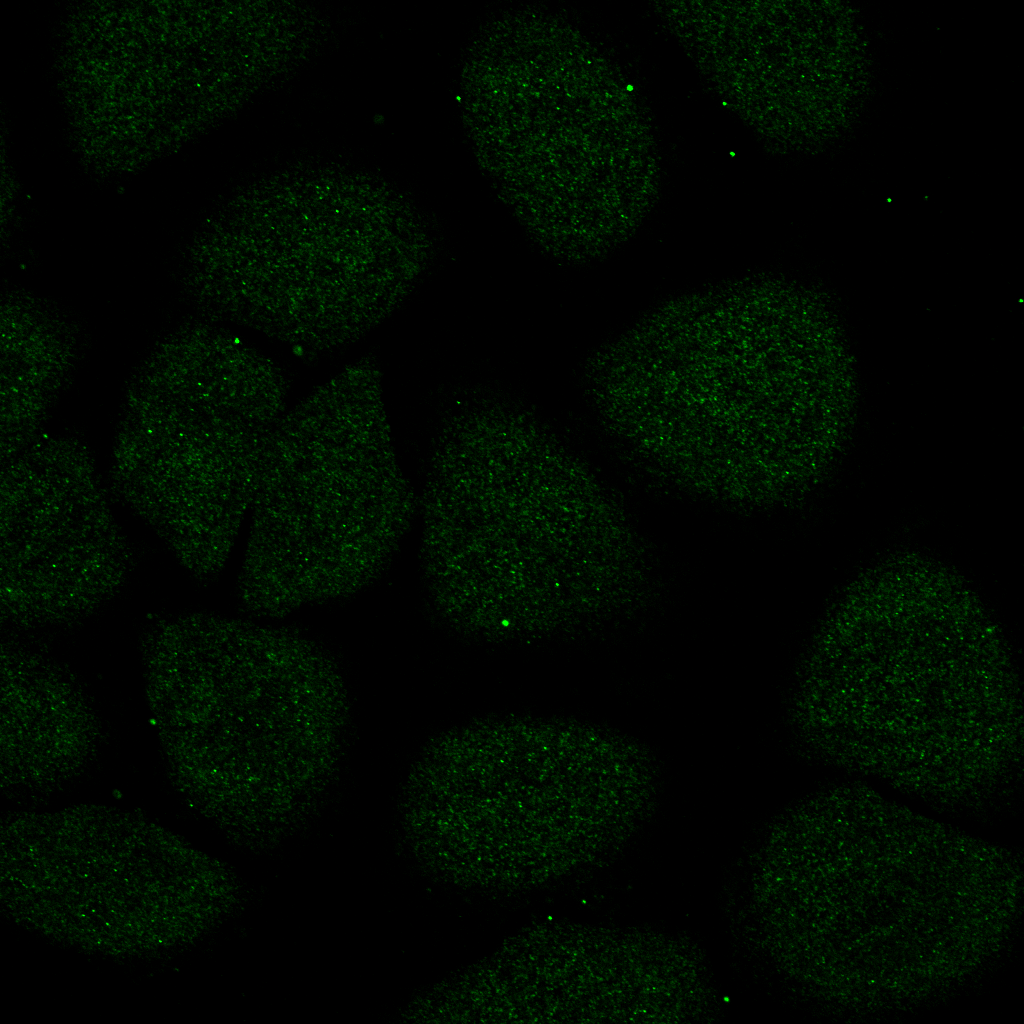

Supplement: Figure 8—source data 1. [file elife-69916-fig8-data1.zip › Figure8_Sourcedata_localization of Endonuclease G/Figure 8A_Representative images_localization of EndoG to mitochondria/Figure 8A_Source file_3-293T/1_0007.tif.frames/1_0007_C003T001.tif]

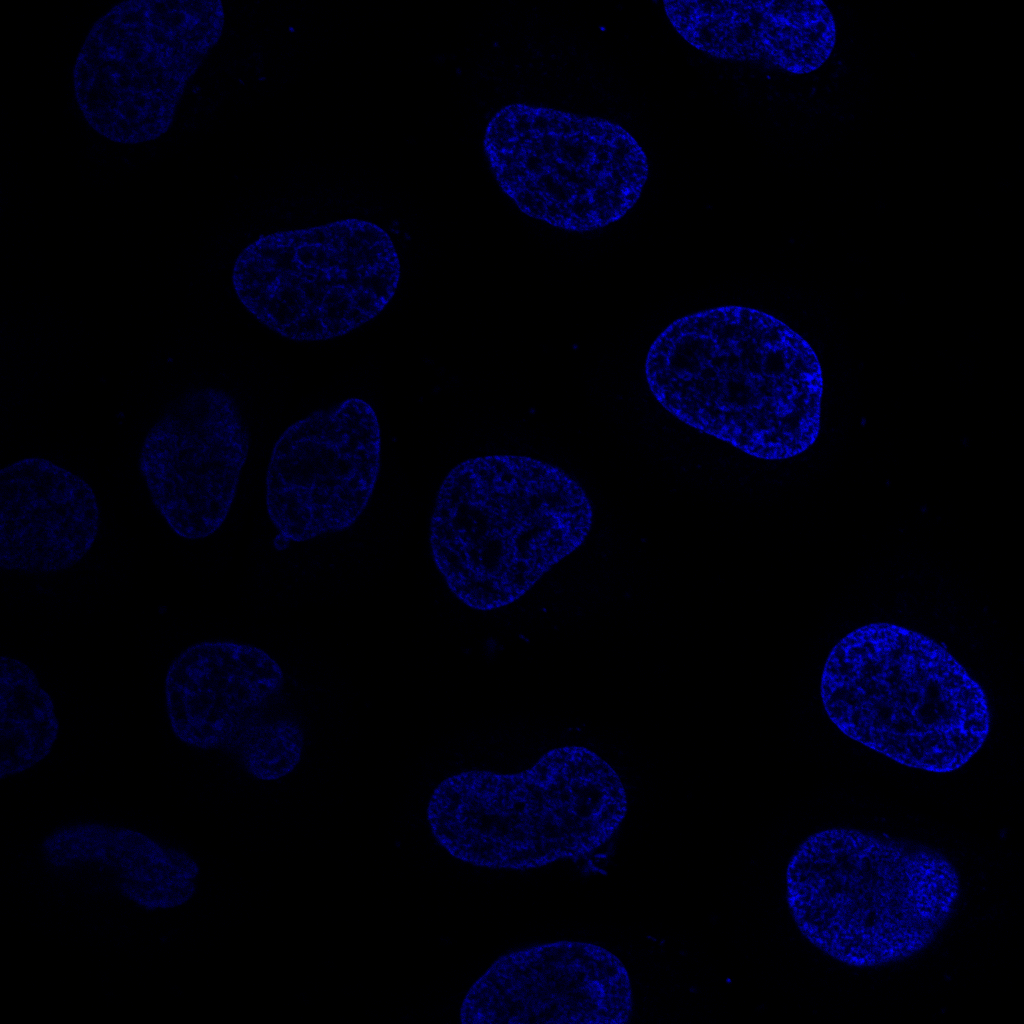

Supplement: Figure 8—source data 1. [file elife-69916-fig8-data1.zip › Figure8_Sourcedata_localization of Endonuclease G/Figure 8A_Representative images_localization of EndoG to mitochondria/Figure 8A_Source file_3-293T/1_0007.tif.frames/1_0007_C001T001.tif]

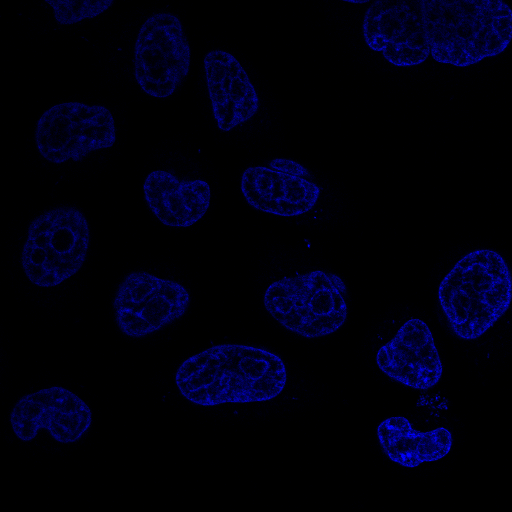

Supplement: Figure 8—source data 1. [file elife-69916-fig8-data1.zip › Figure8_Sourcedata_localization of Endonuclease G/Figure 8A_Representative images_localization of EndoG to mitochondria/Figure 8A_Source file_3-293T/1_0002.tif.frames/1_0002_C001T001.tif]

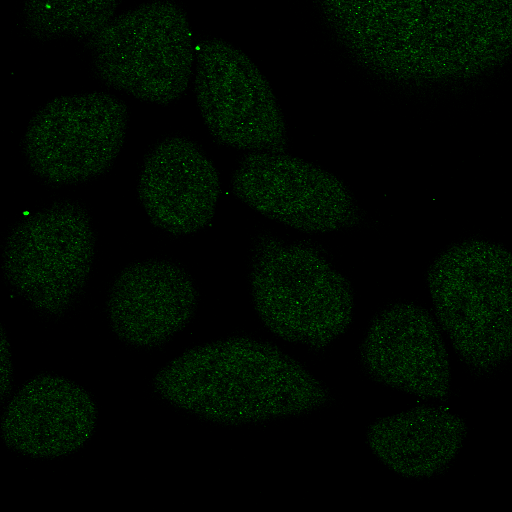

Supplement: Figure 8—source data 1. [file elife-69916-fig8-data1.zip › Figure8_Sourcedata_localization of Endonuclease G/Figure 8A_Representative images_localization of EndoG to mitochondria/Figure 8A_Source file_3-293T/1_0002.tif.frames/1_0002_C003T001.tif]

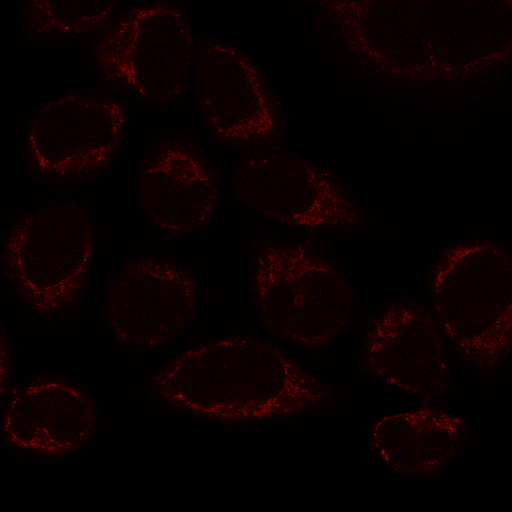

Supplement: Figure 8—source data 1. [file elife-69916-fig8-data1.zip › Figure8_Sourcedata_localization of Endonuclease G/Figure 8A_Representative images_localization of EndoG to mitochondria/Figure 8A_Source file_3-293T/1_0002.tif.frames/1_0002_C002T001.tif]

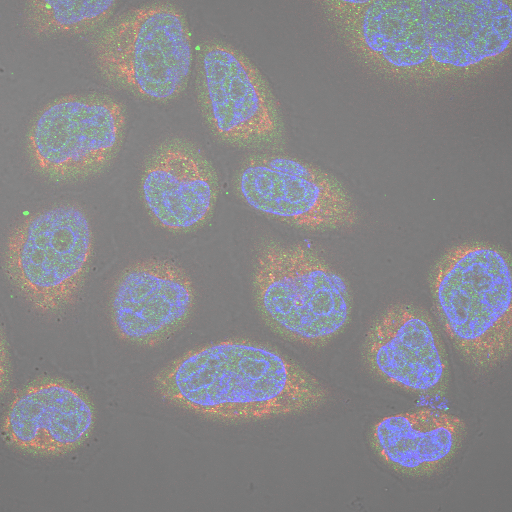

Supplement: Figure 8—source data 1. [file elife-69916-fig8-data1.zip › Figure8_Sourcedata_localization of Endonuclease G/Figure 8A_Representative images_localization of EndoG to mitochondria/Figure 8A_Source file_3-293T/1_0002.tif.frames/1_0002_T001.tif]

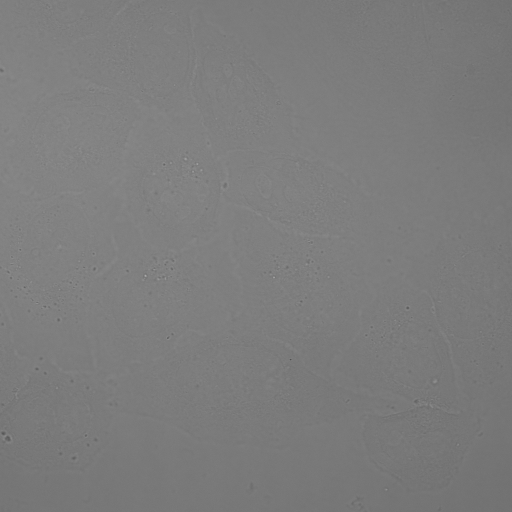

Supplement: Figure 8—source data 1. [file elife-69916-fig8-data1.zip › Figure8_Sourcedata_localization of Endonuclease G/Figure 8A_Representative images_localization of EndoG to mitochondria/Figure 8A_Source file_3-293T/1_0002.tif.frames/1_0002_C004T001.tif]

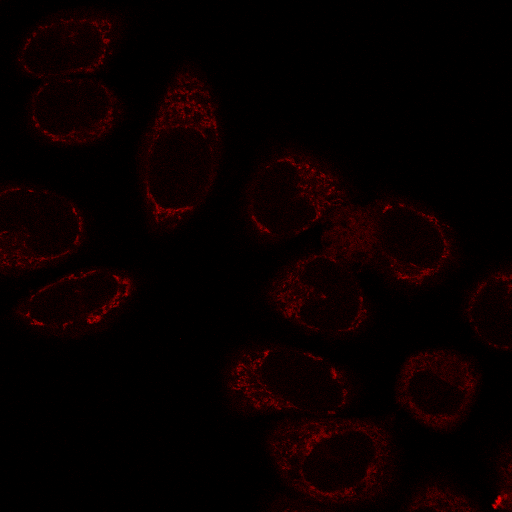

Supplement: Figure 8—source data 1. [file elife-69916-fig8-data1.zip › Figure8_Sourcedata_localization of Endonuclease G/Figure 8A_Representative images_localization of EndoG to mitochondria/Figure 8A_Source file_3-293T/1_0008.tif.frames/1_0008_C002T001.tif]

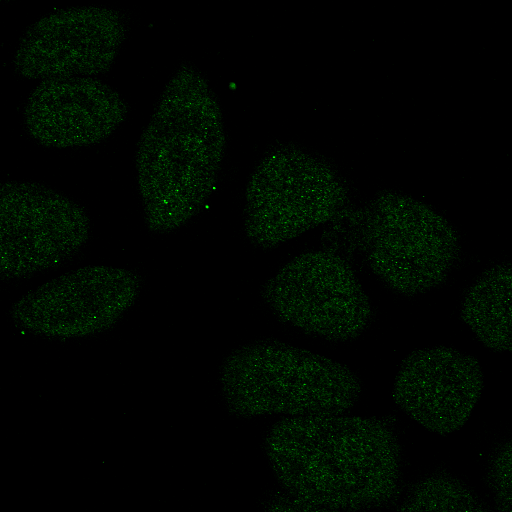

Supplement: Figure 8—source data 1. [file elife-69916-fig8-data1.zip › Figure8_Sourcedata_localization of Endonuclease G/Figure 8A_Representative images_localization of EndoG to mitochondria/Figure 8A_Source file_3-293T/1_0008.tif.frames/1_0008_C003T001.tif]

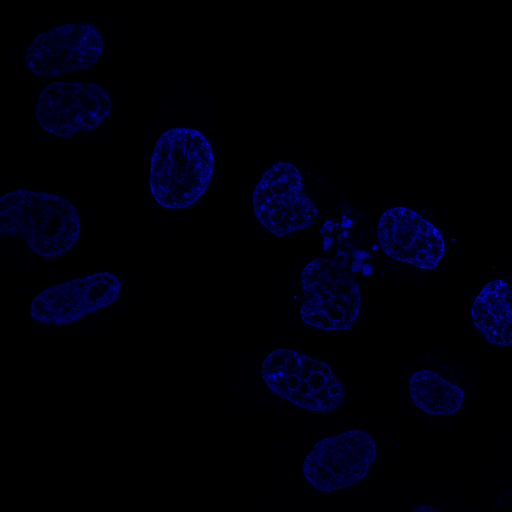

Supplement: Figure 8—source data 1. [file elife-69916-fig8-data1.zip › Figure8_Sourcedata_localization of Endonuclease G/Figure 8A_Representative images_localization of EndoG to mitochondria/Figure 8A_Source file_3-293T/1_0008.tif.frames/1_0008_C001T001.tif]

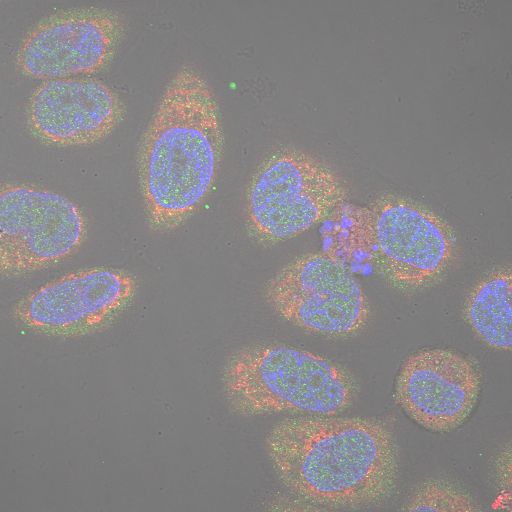

Supplement: Figure 8—source data 1. [file elife-69916-fig8-data1.zip › Figure8_Sourcedata_localization of Endonuclease G/Figure 8A_Representative images_localization of EndoG to mitochondria/Figure 8A_Source file_3-293T/1_0008.tif.frames/1_0008_T001.tif]

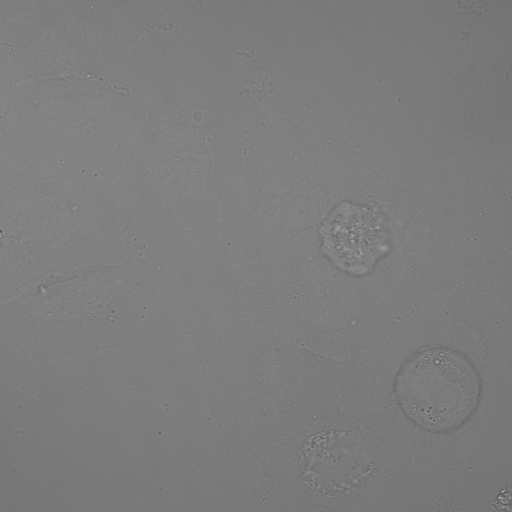

Supplement: Figure 8—source data 1. [file elife-69916-fig8-data1.zip › Figure8_Sourcedata_localization of Endonuclease G/Figure 8A_Representative images_localization of EndoG to mitochondria/Figure 8A_Source file_3-293T/1_0008.tif.frames/1_0008_C004T001.tif]

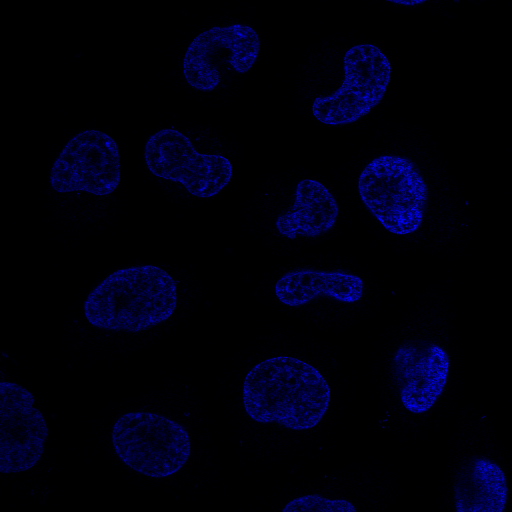

Supplement: Figure 8—source data 1. [file elife-69916-fig8-data1.zip › Figure8_Sourcedata_localization of Endonuclease G/Figure 8A_Representative images_localization of EndoG to mitochondria/Figure 8A_Source file_3-293T/1_0005.tif.frames/1_0005_C001T001.tif]

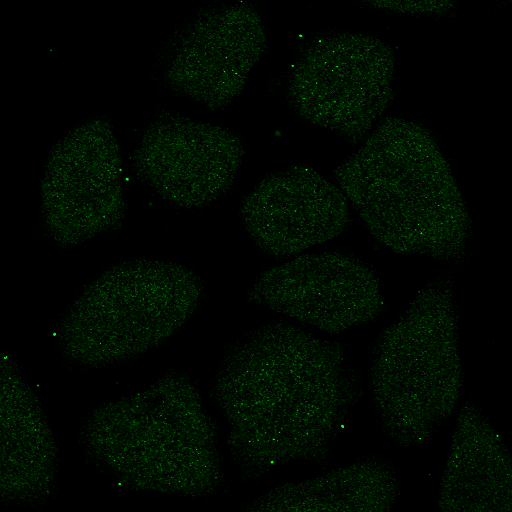

Supplement: Figure 8—source data 1. [file elife-69916-fig8-data1.zip › Figure8_Sourcedata_localization of Endonuclease G/Figure 8A_Representative images_localization of EndoG to mitochondria/Figure 8A_Source file_3-293T/1_0005.tif.frames/1_0005_C003T001.tif]

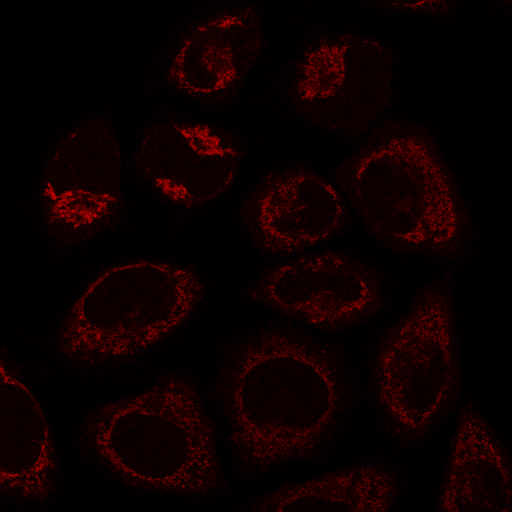

Supplement: Figure 8—source data 1. [file elife-69916-fig8-data1.zip › Figure8_Sourcedata_localization of Endonuclease G/Figure 8A_Representative images_localization of EndoG to mitochondria/Figure 8A_Source file_3-293T/1_0005.tif.frames/1_0005_C002T001.tif]

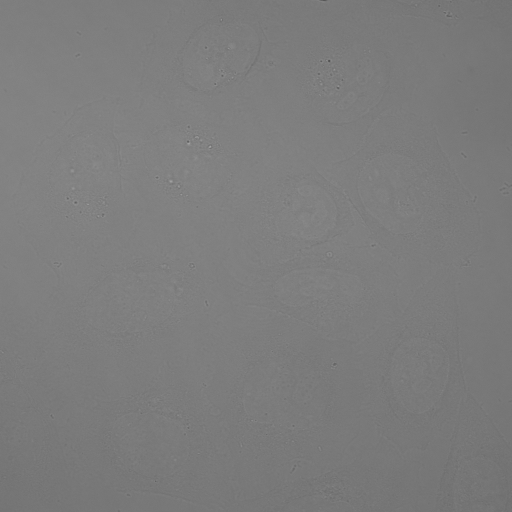

Supplement: Figure 8—source data 1. [file elife-69916-fig8-data1.zip › Figure8_Sourcedata_localization of Endonuclease G/Figure 8A_Representative images_localization of EndoG to mitochondria/Figure 8A_Source file_3-293T/1_0005.tif.frames/1_0005_C004T001.tif]

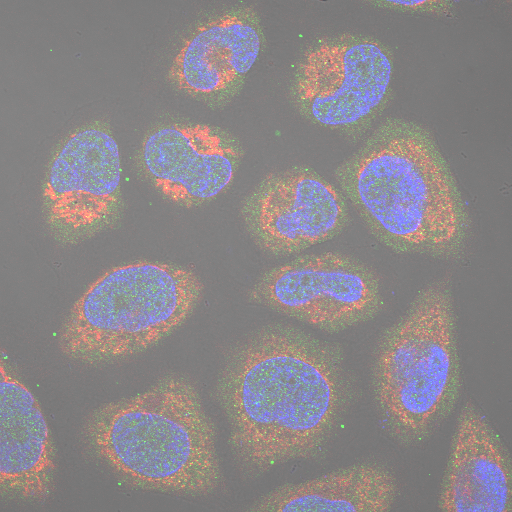

Supplement: Figure 8—source data 1. [file elife-69916-fig8-data1.zip › Figure8_Sourcedata_localization of Endonuclease G/Figure 8A_Representative images_localization of EndoG to mitochondria/Figure 8A_Source file_3-293T/1_0005.tif.frames/1_0005_T001.tif]

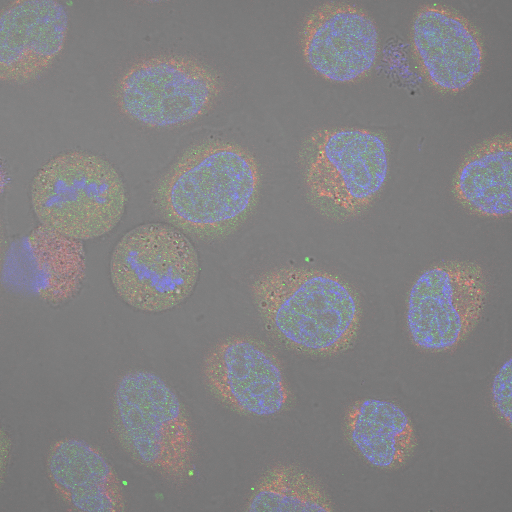

Supplement: Figure 8—source data 1. [file elife-69916-fig8-data1.zip › Figure8_Sourcedata_localization of Endonuclease G/Figure 8A_Representative images_localization of EndoG to mitochondria/Figure 8A_Source file_3-293T/1.tif.frames/1_T001.tif]

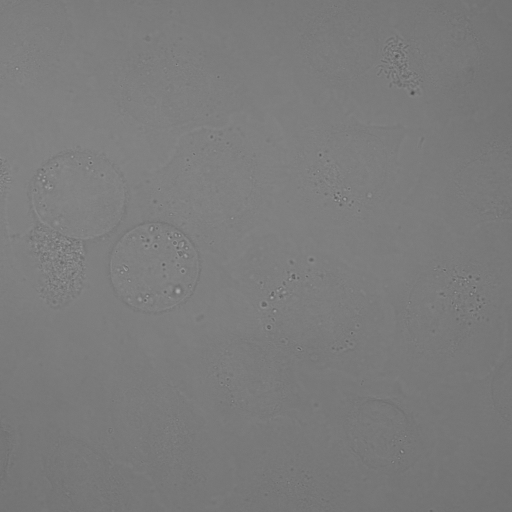

Supplement: Figure 8—source data 1. [file elife-69916-fig8-data1.zip › Figure8_Sourcedata_localization of Endonuclease G/Figure 8A_Representative images_localization of EndoG to mitochondria/Figure 8A_Source file_3-293T/1.tif.frames/1_C004T001.tif]

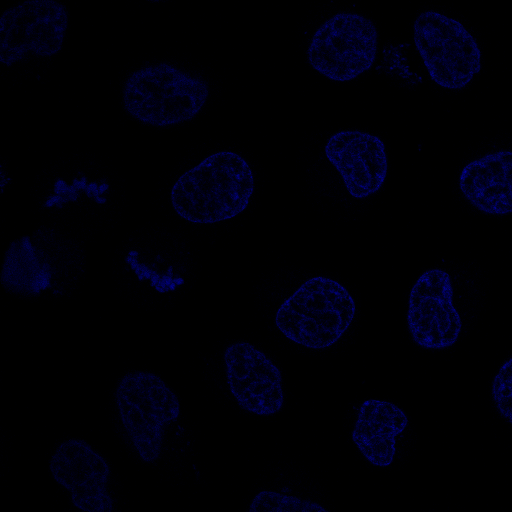

Supplement: Figure 8—source data 1. [file elife-69916-fig8-data1.zip › Figure8_Sourcedata_localization of Endonuclease G/Figure 8A_Representative images_localization of EndoG to mitochondria/Figure 8A_Source file_3-293T/1.tif.frames/1_C001T001.tif]

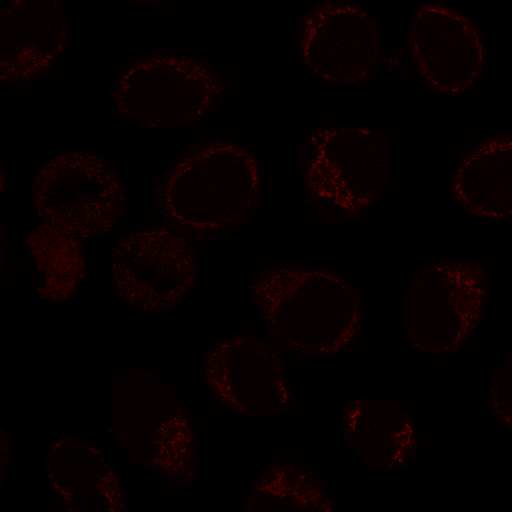

Supplement: Figure 8—source data 1. [file elife-69916-fig8-data1.zip › Figure8_Sourcedata_localization of Endonuclease G/Figure 8A_Representative images_localization of EndoG to mitochondria/Figure 8A_Source file_3-293T/1.tif.frames/1_C002T001.tif]

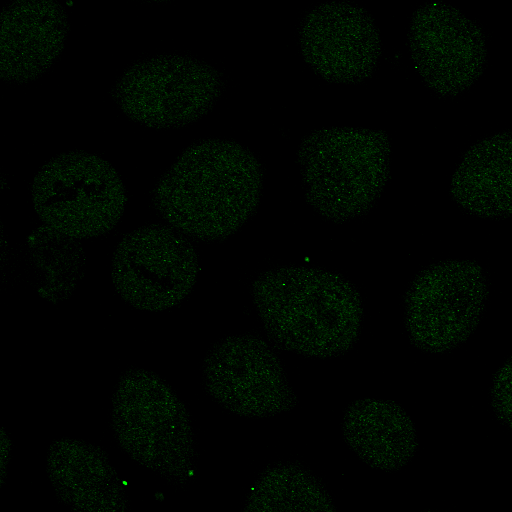

Supplement: Figure 8—source data 1. [file elife-69916-fig8-data1.zip › Figure8_Sourcedata_localization of Endonuclease G/Figure 8A_Representative images_localization of EndoG to mitochondria/Figure 8A_Source file_3-293T/1.tif.frames/1_C003T001.tif]

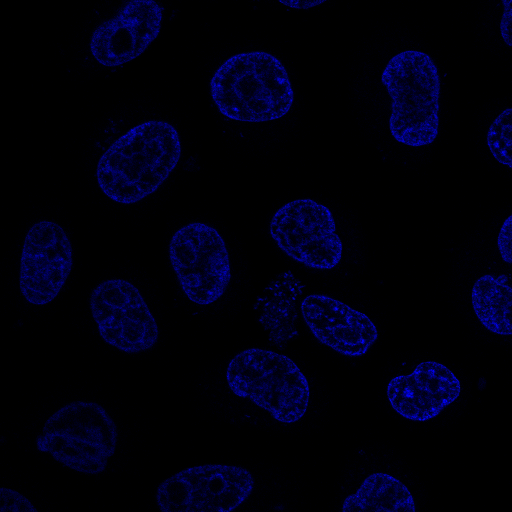

Supplement: Figure 8—source data 1. [file elife-69916-fig8-data1.zip › Figure8_Sourcedata_localization of Endonuclease G/Figure 8A_Representative images_localization of EndoG to mitochondria/Figure 8A_Source file_3-293T/1_0003.tif.frames/1_0003_C001T001.tif]

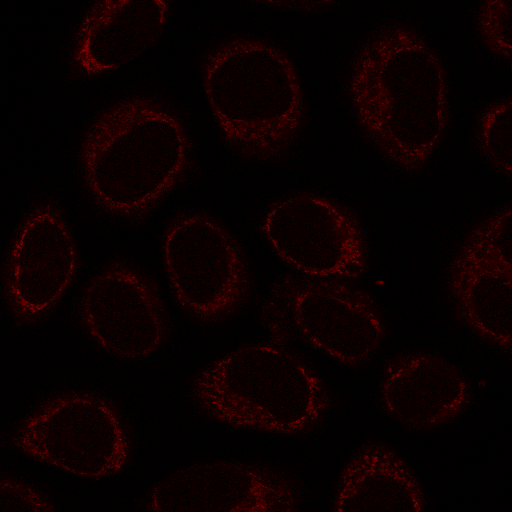

Supplement: Figure 8—source data 1. [file elife-69916-fig8-data1.zip › Figure8_Sourcedata_localization of Endonuclease G/Figure 8A_Representative images_localization of EndoG to mitochondria/Figure 8A_Source file_3-293T/1_0003.tif.frames/1_0003_C002T001.tif]

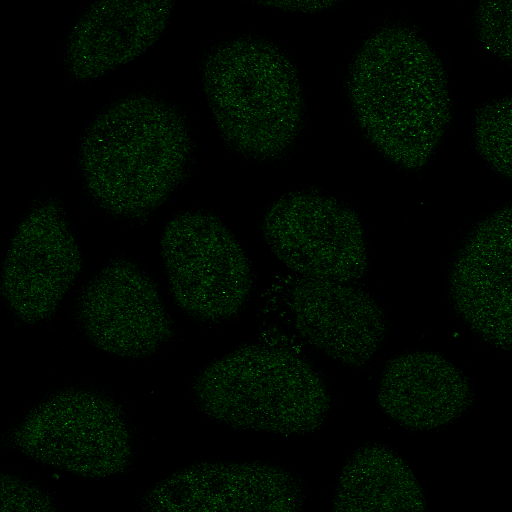

Supplement: Figure 8—source data 1. [file elife-69916-fig8-data1.zip › Figure8_Sourcedata_localization of Endonuclease G/Figure 8A_Representative images_localization of EndoG to mitochondria/Figure 8A_Source file_3-293T/1_0003.tif.frames/1_0003_C003T001.tif]

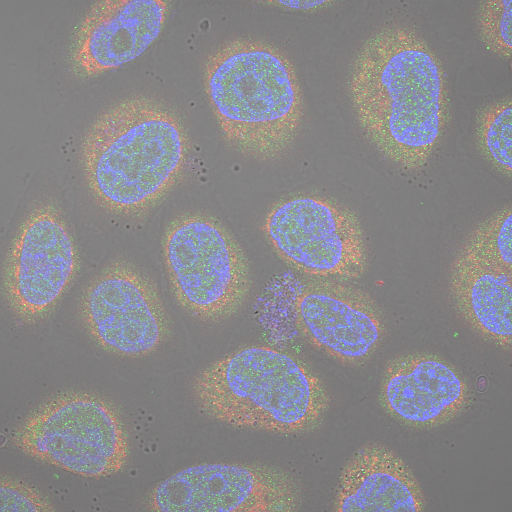

Supplement: Figure 8—source data 1. [file elife-69916-fig8-data1.zip › Figure8_Sourcedata_localization of Endonuclease G/Figure 8A_Representative images_localization of EndoG to mitochondria/Figure 8A_Source file_3-293T/1_0003.tif.frames/1_0003_T001.tif]

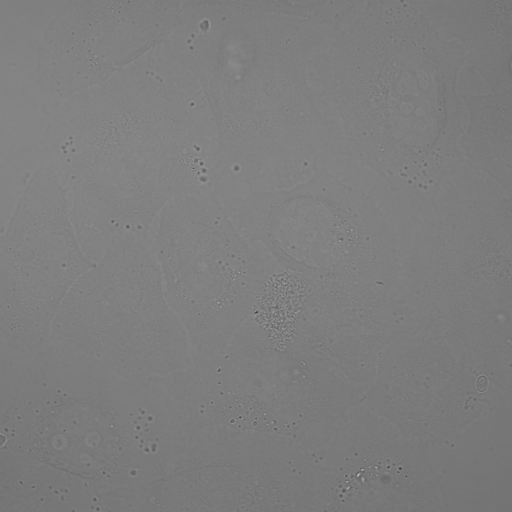

Supplement: Figure 8—source data 1. [file elife-69916-fig8-data1.zip › Figure8_Sourcedata_localization of Endonuclease G/Figure 8A_Representative images_localization of EndoG to mitochondria/Figure 8A_Source file_3-293T/1_0003.tif.frames/1_0003_C004T001.tif]

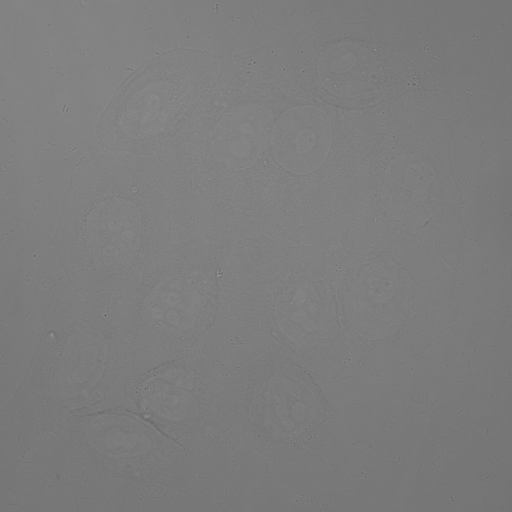

Supplement: Figure 8—source data 1. [file elife-69916-fig8-data1.zip › Figure8_Sourcedata_localization of Endonuclease G/Figure 8A_Representative images_localization of EndoG to mitochondria/Figure 8A_Source file_3-293T/1_0006.tif.frames/1_0006_C004T001.tif]

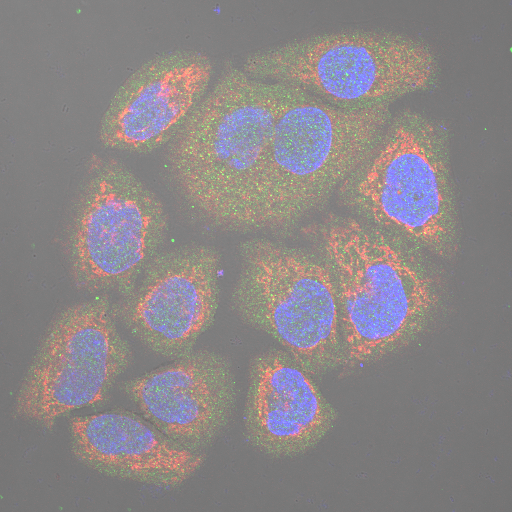

Supplement: Figure 8—source data 1. [file elife-69916-fig8-data1.zip › Figure8_Sourcedata_localization of Endonuclease G/Figure 8A_Representative images_localization of EndoG to mitochondria/Figure 8A_Source file_3-293T/1_0006.tif.frames/1_0006_T001.tif]

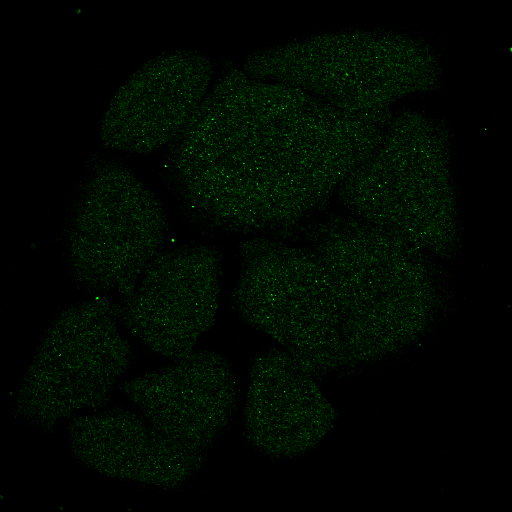

Supplement: Figure 8—source data 1. [file elife-69916-fig8-data1.zip › Figure8_Sourcedata_localization of Endonuclease G/Figure 8A_Representative images_localization of EndoG to mitochondria/Figure 8A_Source file_3-293T/1_0006.tif.frames/1_0006_C003T001.tif]

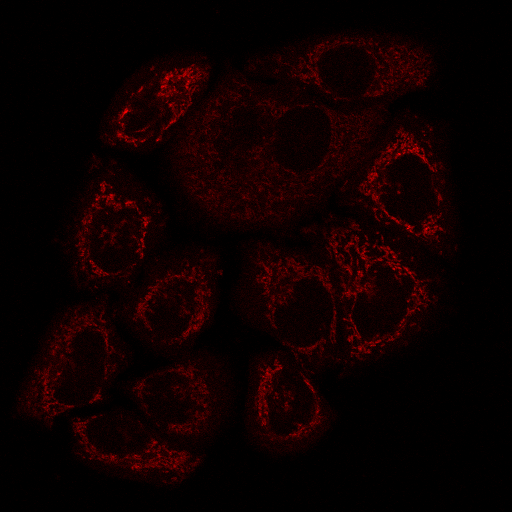

Supplement: Figure 8—source data 1. [file elife-69916-fig8-data1.zip › Figure8_Sourcedata_localization of Endonuclease G/Figure 8A_Representative images_localization of EndoG to mitochondria/Figure 8A_Source file_3-293T/1_0006.tif.frames/1_0006_C002T001.tif]

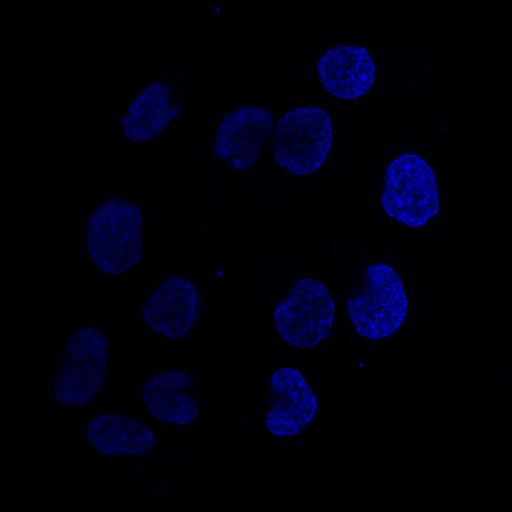

Supplement: Figure 8—source data 1. [file elife-69916-fig8-data1.zip › Figure8_Sourcedata_localization of Endonuclease G/Figure 8A_Representative images_localization of EndoG to mitochondria/Figure 8A_Source file_3-293T/1_0006.tif.frames/1_0006_C001T001.tif]

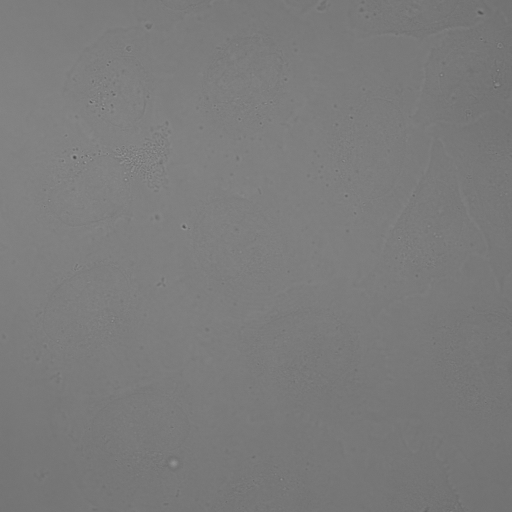

Supplement: Figure 8—source data 1. [file elife-69916-fig8-data1.zip › Figure8_Sourcedata_localization of Endonuclease G/Figure 8A_Representative images_localization of EndoG to mitochondria/Figure 8A_Source file_3-293T/1_0001.tif.frames/1_0001_C004T001.tif]

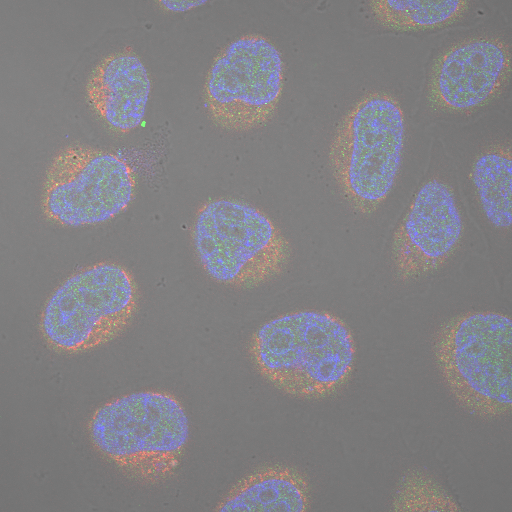

Supplement: Figure 8—source data 1. [file elife-69916-fig8-data1.zip › Figure8_Sourcedata_localization of Endonuclease G/Figure 8A_Representative images_localization of EndoG to mitochondria/Figure 8A_Source file_3-293T/1_0001.tif.frames/1_0001_T001.tif]

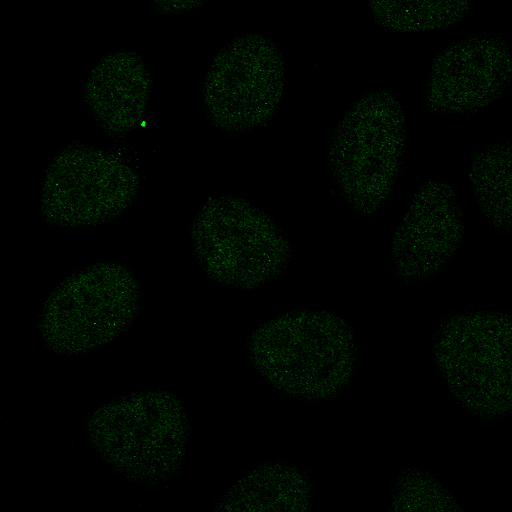

Supplement: Figure 8—source data 1. [file elife-69916-fig8-data1.zip › Figure8_Sourcedata_localization of Endonuclease G/Figure 8A_Representative images_localization of EndoG to mitochondria/Figure 8A_Source file_3-293T/1_0001.tif.frames/1_0001_C003T001.tif]

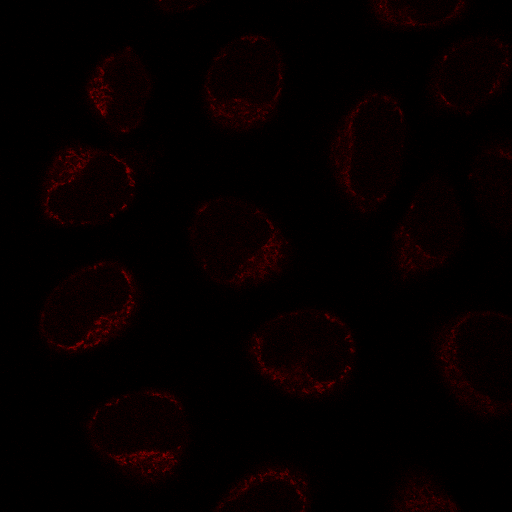

Supplement: Figure 8—source data 1. [file elife-69916-fig8-data1.zip › Figure8_Sourcedata_localization of Endonuclease G/Figure 8A_Representative images_localization of EndoG to mitochondria/Figure 8A_Source file_3-293T/1_0001.tif.frames/1_0001_C002T001.tif]

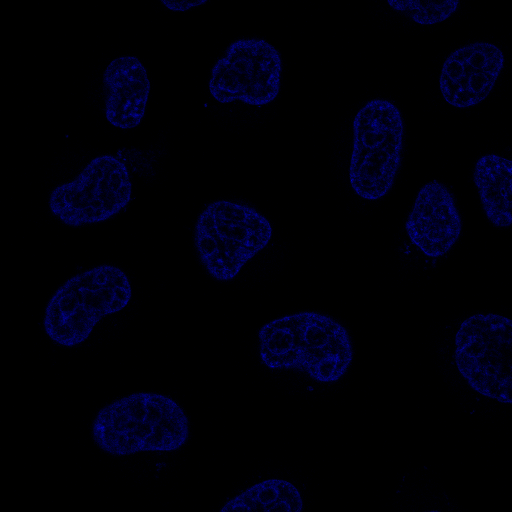

Supplement: Figure 8—source data 1. [file elife-69916-fig8-data1.zip › Figure8_Sourcedata_localization of Endonuclease G/Figure 8A_Representative images_localization of EndoG to mitochondria/Figure 8A_Source file_3-293T/1_0001.tif.frames/1_0001_C001T001.tif]

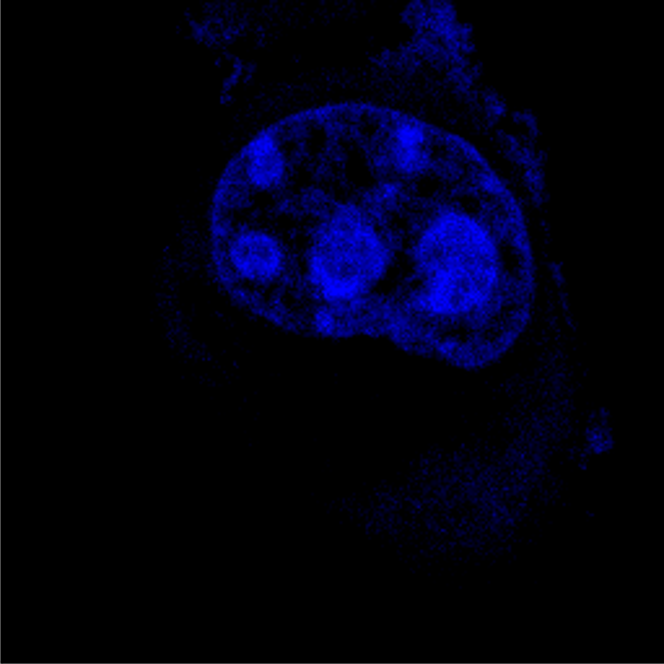

Supplement: Figure 8—source data 1. [file elife-69916-fig8-data1.zip › Figure8_Sourcedata_localization of Endonuclease G/Figure 8A_Representative images_localization of EndoG to mitochondria/Figure 8A_Source file_3-293T/1_0010.tif.frames/1_0010_C001T001.tif]

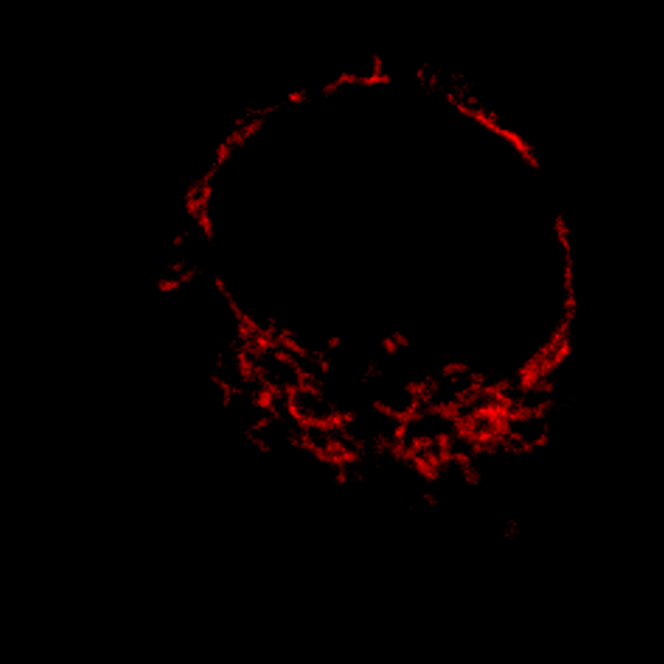

Supplement: Figure 8—source data 1. [file elife-69916-fig8-data1.zip › Figure8_Sourcedata_localization of Endonuclease G/Figure 8A_Representative images_localization of EndoG to mitochondria/Figure 8A_Source file_3-293T/1_0010.tif.frames/1_0010_C001T002.tif]

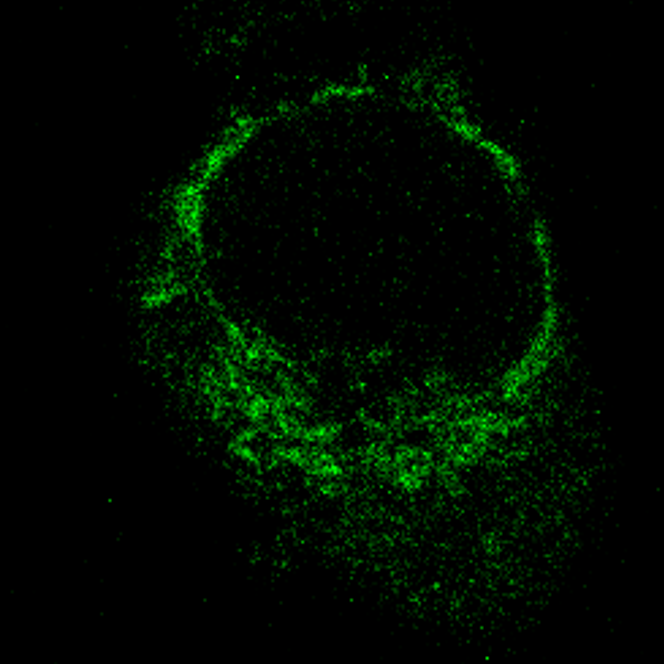

Supplement: Figure 8—source data 1. [file elife-69916-fig8-data1.zip › Figure8_Sourcedata_localization of Endonuclease G/Figure 8A_Representative images_localization of EndoG to mitochondria/Figure 8A_Source file_3-293T/1_0010.tif.frames/1_0010_C001T003.tif]

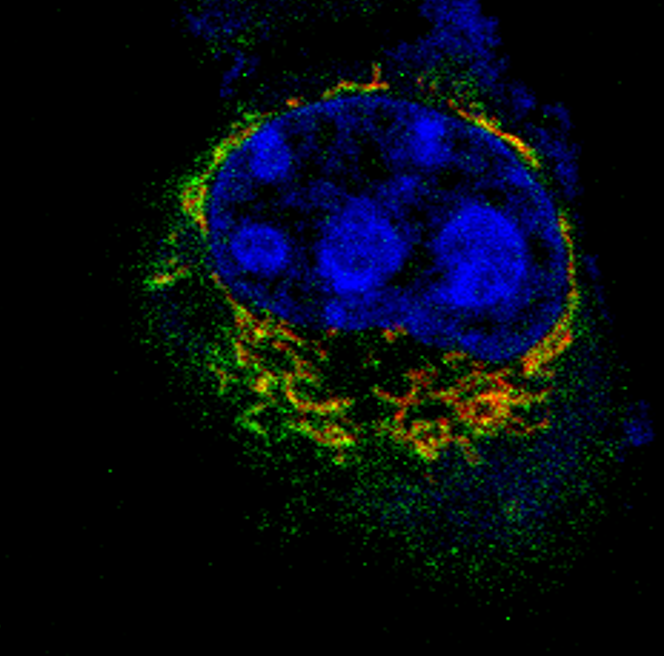

Supplement: Figure 8—source data 1. [file elife-69916-fig8-data1.zip › Figure8_Sourcedata_localization of Endonuclease G/Figure 8A_Representative images_localization of EndoG to mitochondria/Figure 8A_Source file_3-293T/1_0010.tif.frames/1_0010_T001.tif]

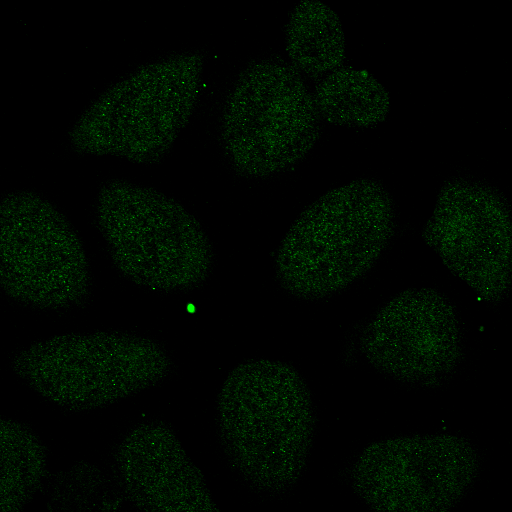

Supplement: Figure 8—source data 1. [file elife-69916-fig8-data1.zip › Figure8_Sourcedata_localization of Endonuclease G/Figure 8A_Representative images_localization of EndoG to mitochondria/Figure 8A_Source file_3-293T/1_0009.tif.frames/1_0009_C003T001.tif]

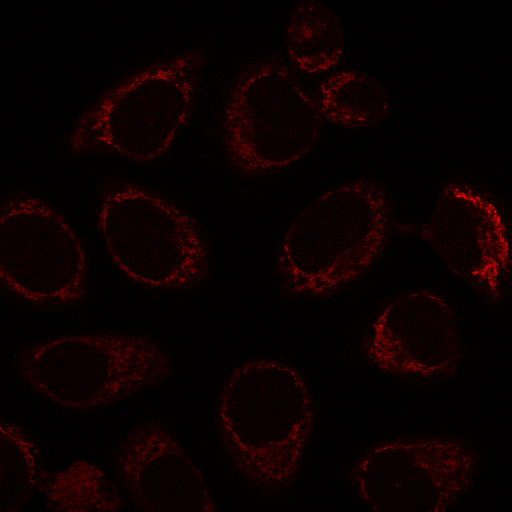

Supplement: Figure 8—source data 1. [file elife-69916-fig8-data1.zip › Figure8_Sourcedata_localization of Endonuclease G/Figure 8A_Representative images_localization of EndoG to mitochondria/Figure 8A_Source file_3-293T/1_0009.tif.frames/1_0009_C002T001.tif]

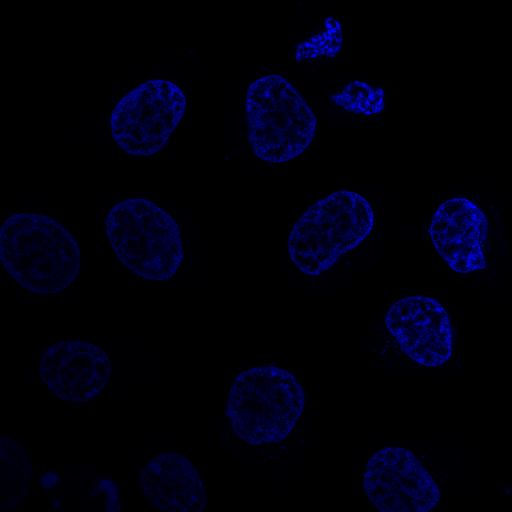

Supplement: Figure 8—source data 1. [file elife-69916-fig8-data1.zip › Figure8_Sourcedata_localization of Endonuclease G/Figure 8A_Representative images_localization of EndoG to mitochondria/Figure 8A_Source file_3-293T/1_0009.tif.frames/1_0009_C001T001.tif]

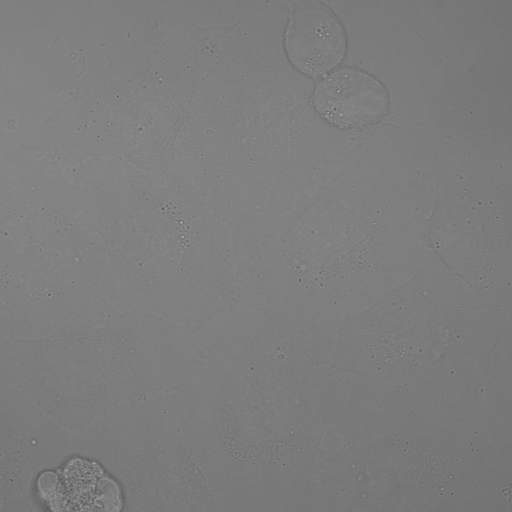

Supplement: Figure 8—source data 1. [file elife-69916-fig8-data1.zip › Figure8_Sourcedata_localization of Endonuclease G/Figure 8A_Representative images_localization of EndoG to mitochondria/Figure 8A_Source file_3-293T/1_0009.tif.frames/1_0009_C004T001.tif]

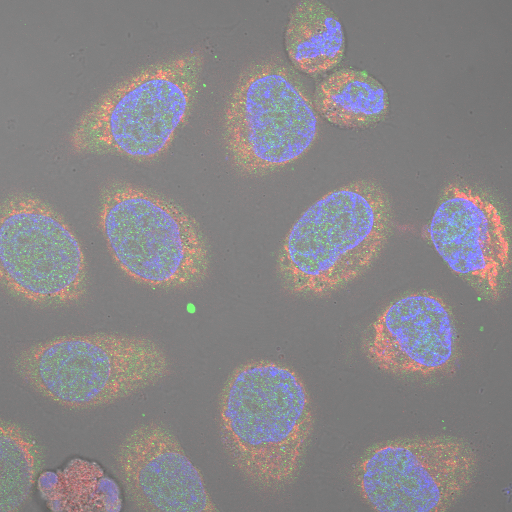

Supplement: Figure 8—source data 1. [file elife-69916-fig8-data1.zip › Figure8_Sourcedata_localization of Endonuclease G/Figure 8A_Representative images_localization of EndoG to mitochondria/Figure 8A_Source file_3-293T/1_0009.tif.frames/1_0009_T001.tif]

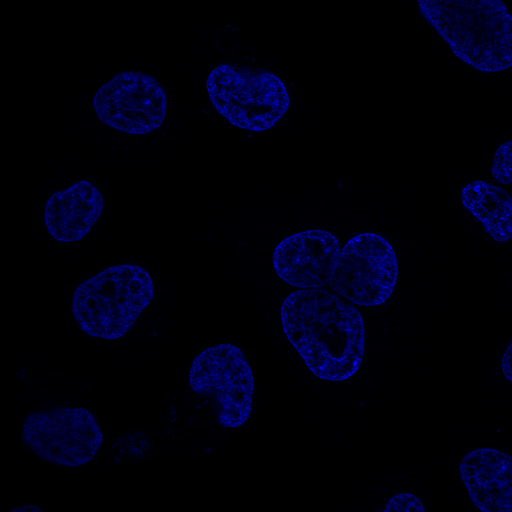

Supplement: Figure 8—source data 1. [file elife-69916-fig8-data1.zip › Figure8_Sourcedata_localization of Endonuclease G/Figure 8A_Representative images_localization of EndoG to mitochondria/Figure 8A_Source file_3-293T/1_0004.tif.frames/1_0004_C001T001.tif]

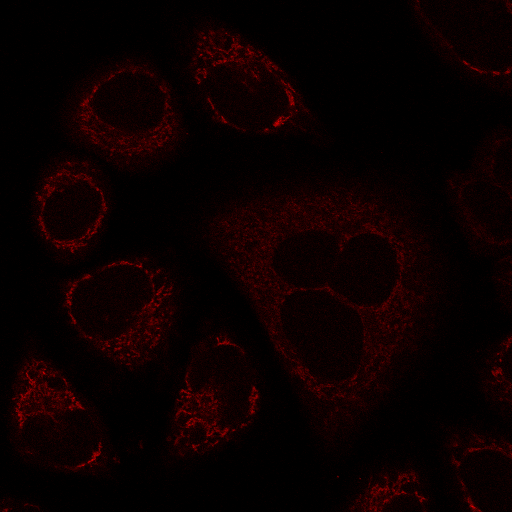

Supplement: Figure 8—source data 1. [file elife-69916-fig8-data1.zip › Figure8_Sourcedata_localization of Endonuclease G/Figure 8A_Representative images_localization of EndoG to mitochondria/Figure 8A_Source file_3-293T/1_0004.tif.frames/1_0004_C002T001.tif]

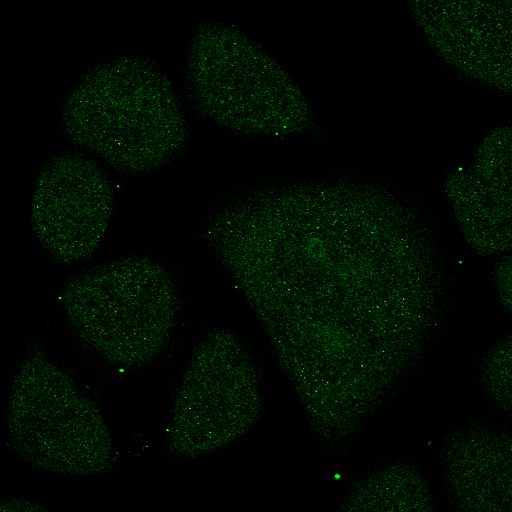

Supplement: Figure 8—source data 1. [file elife-69916-fig8-data1.zip › Figure8_Sourcedata_localization of Endonuclease G/Figure 8A_Representative images_localization of EndoG to mitochondria/Figure 8A_Source file_3-293T/1_0004.tif.frames/1_0004_C003T001.tif]

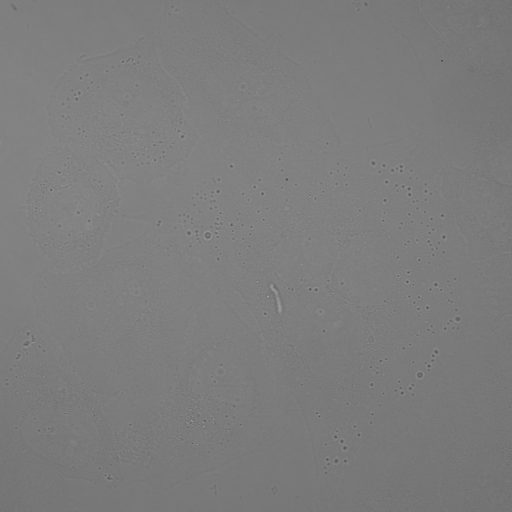

Supplement: Figure 8—source data 1. [file elife-69916-fig8-data1.zip › Figure8_Sourcedata_localization of Endonuclease G/Figure 8A_Representative images_localization of EndoG to mitochondria/Figure 8A_Source file_3-293T/1_0004.tif.frames/1_0004_C004T001.tif]

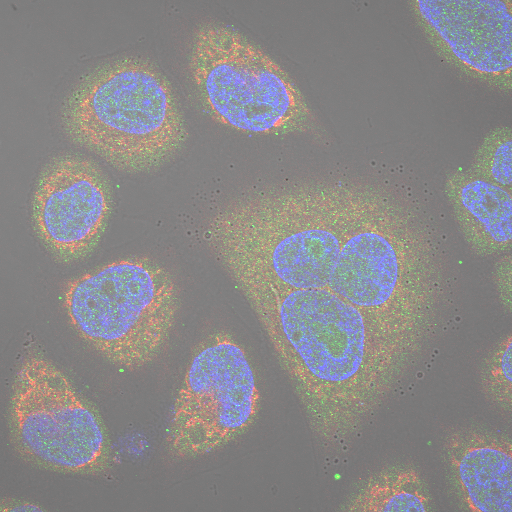

Supplement: Figure 8—source data 1. [file elife-69916-fig8-data1.zip › Figure8_Sourcedata_localization of Endonuclease G/Figure 8A_Representative images_localization of EndoG to mitochondria/Figure 8A_Source file_3-293T/1_0004.tif.frames/1_0004_T001.tif]

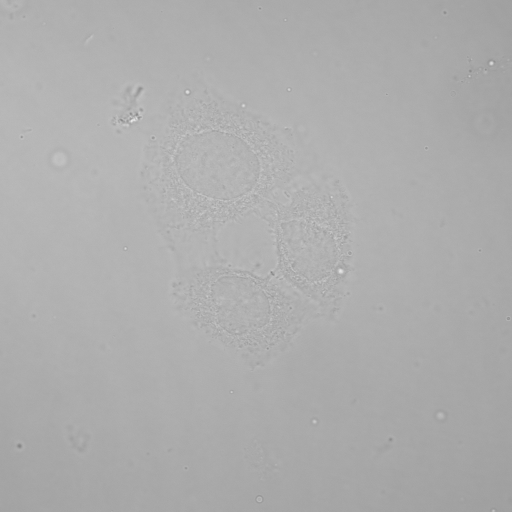

Supplement: Figure 8—source data 1. [file elife-69916-fig8-data1.zip › Figure8_Sourcedata_localization of Endonuclease G/Figure 8A_Representative images_localization of EndoG to mitochondria/Figure 8A_Source file_2_HeLa/1_0007.tif.frames/1_0007_C004T001.tif]

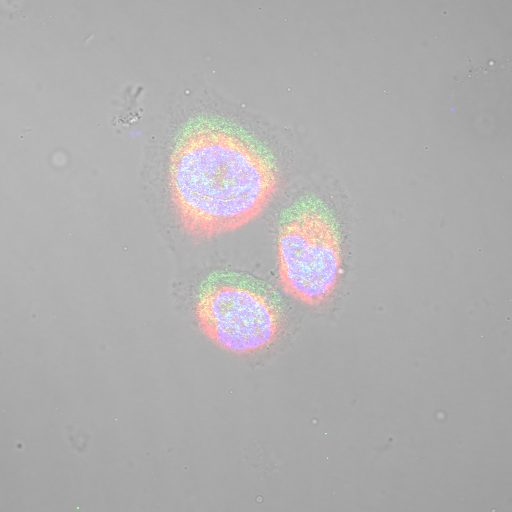

Supplement: Figure 8—source data 1. [file elife-69916-fig8-data1.zip › Figure8_Sourcedata_localization of Endonuclease G/Figure 8A_Representative images_localization of EndoG to mitochondria/Figure 8A_Source file_2_HeLa/1_0007.tif.frames/1_0007_T001.tif]

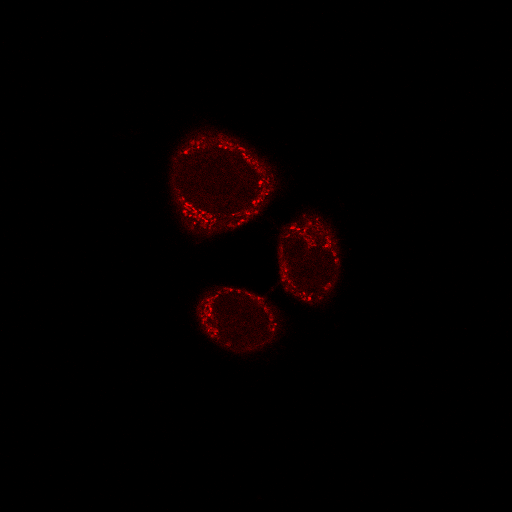

Supplement: Figure 8—source data 1. [file elife-69916-fig8-data1.zip › Figure8_Sourcedata_localization of Endonuclease G/Figure 8A_Representative images_localization of EndoG to mitochondria/Figure 8A_Source file_2_HeLa/1_0007.tif.frames/1_0007_C002T001.tif]

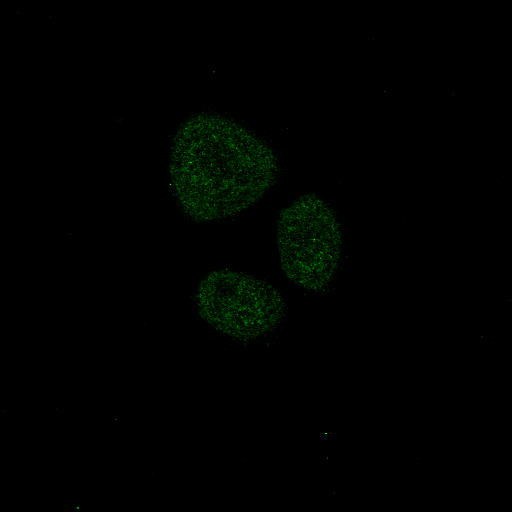

Supplement: Figure 8—source data 1. [file elife-69916-fig8-data1.zip › Figure8_Sourcedata_localization of Endonuclease G/Figure 8A_Representative images_localization of EndoG to mitochondria/Figure 8A_Source file_2_HeLa/1_0007.tif.frames/1_0007_C003T001.tif]

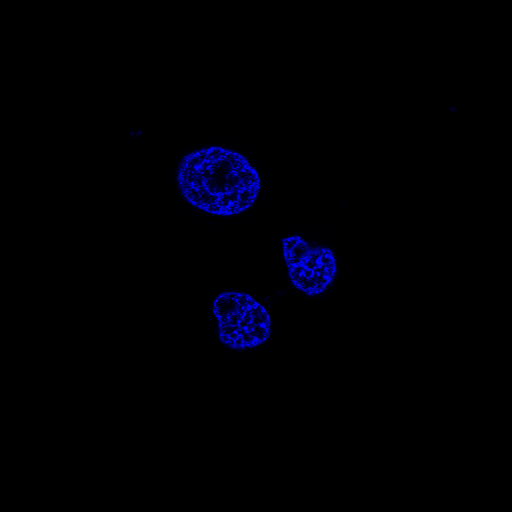

Supplement: Figure 8—source data 1. [file elife-69916-fig8-data1.zip › Figure8_Sourcedata_localization of Endonuclease G/Figure 8A_Representative images_localization of EndoG to mitochondria/Figure 8A_Source file_2_HeLa/1_0007.tif.frames/1_0007_C001T001.tif]

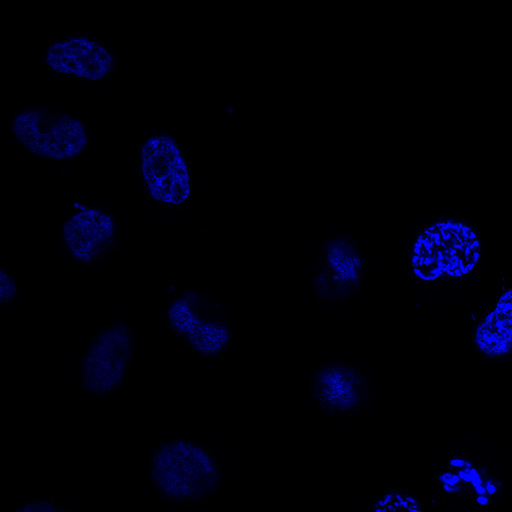

Supplement: Figure 8—source data 1. [file elife-69916-fig8-data1.zip › Figure8_Sourcedata_localization of Endonuclease G/Figure 8A_Representative images_localization of EndoG to mitochondria/Figure 8A_Source file_2_HeLa/1_0002.tif.frames/1_0002_C001T001.tif]

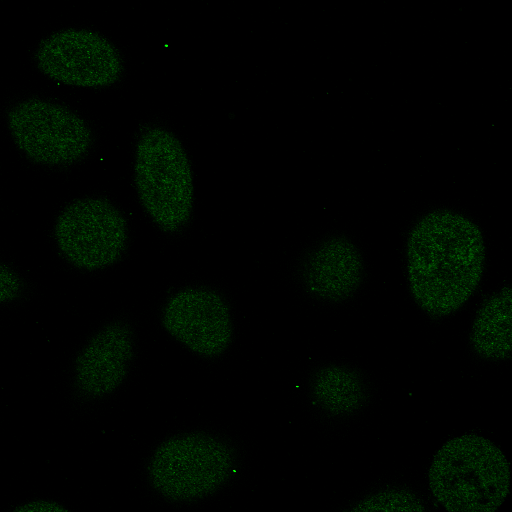

Supplement: Figure 8—source data 1. [file elife-69916-fig8-data1.zip › Figure8_Sourcedata_localization of Endonuclease G/Figure 8A_Representative images_localization of EndoG to mitochondria/Figure 8A_Source file_2_HeLa/1_0002.tif.frames/1_0002_C003T001.tif]

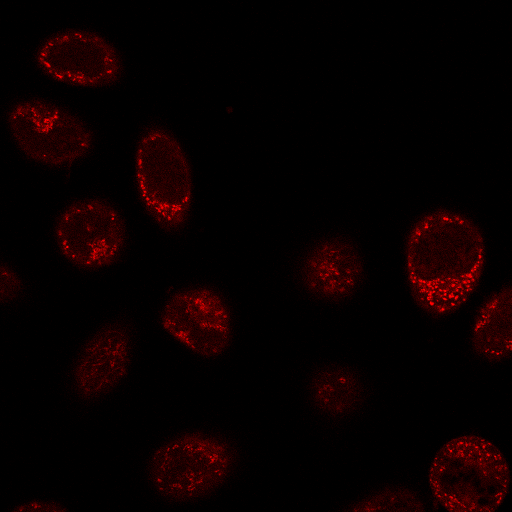

Supplement: Figure 8—source data 1. [file elife-69916-fig8-data1.zip › Figure8_Sourcedata_localization of Endonuclease G/Figure 8A_Representative images_localization of EndoG to mitochondria/Figure 8A_Source file_2_HeLa/1_0002.tif.frames/1_0002_C002T001.tif]

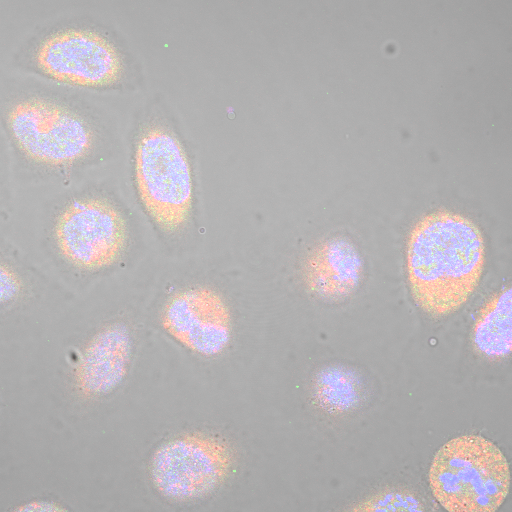

Supplement: Figure 8—source data 1. [file elife-69916-fig8-data1.zip › Figure8_Sourcedata_localization of Endonuclease G/Figure 8A_Representative images_localization of EndoG to mitochondria/Figure 8A_Source file_2_HeLa/1_0002.tif.frames/1_0002_T001.tif]

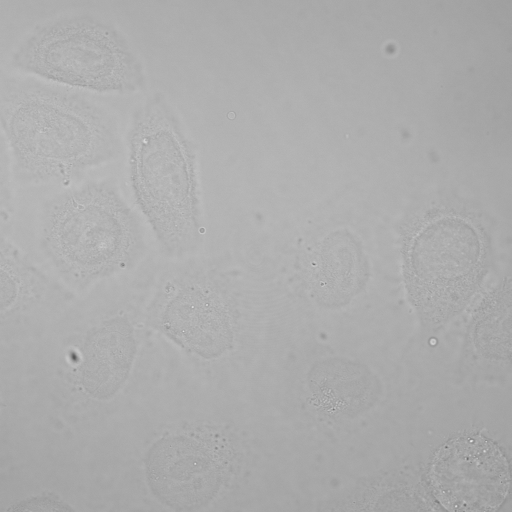

Supplement: Figure 8—source data 1. [file elife-69916-fig8-data1.zip › Figure8_Sourcedata_localization of Endonuclease G/Figure 8A_Representative images_localization of EndoG to mitochondria/Figure 8A_Source file_2_HeLa/1_0002.tif.frames/1_0002_C004T001.tif]

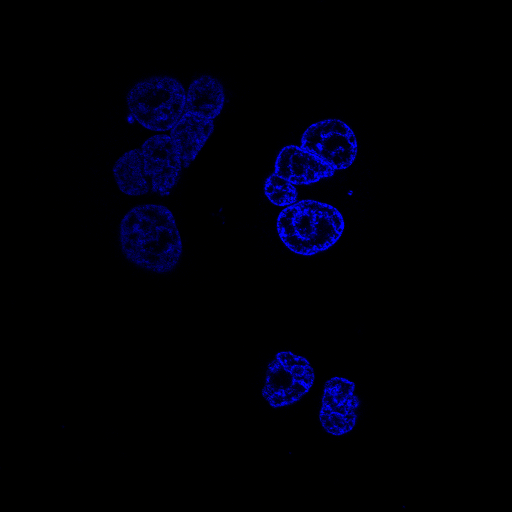

Supplement: Figure 8—source data 1. [file elife-69916-fig8-data1.zip › Figure8_Sourcedata_localization of Endonuclease G/Figure 8A_Representative images_localization of EndoG to mitochondria/Figure 8A_Source file_2_HeLa/1_0005.tif.frames/1_0005_C001T001.tif]

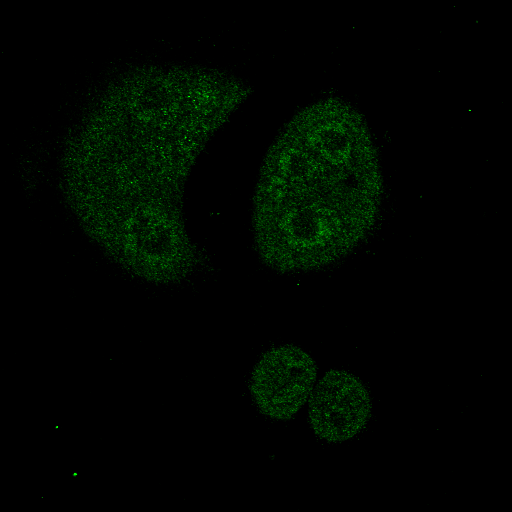

Supplement: Figure 8—source data 1. [file elife-69916-fig8-data1.zip › Figure8_Sourcedata_localization of Endonuclease G/Figure 8A_Representative images_localization of EndoG to mitochondria/Figure 8A_Source file_2_HeLa/1_0005.tif.frames/1_0005_C003T001.tif]

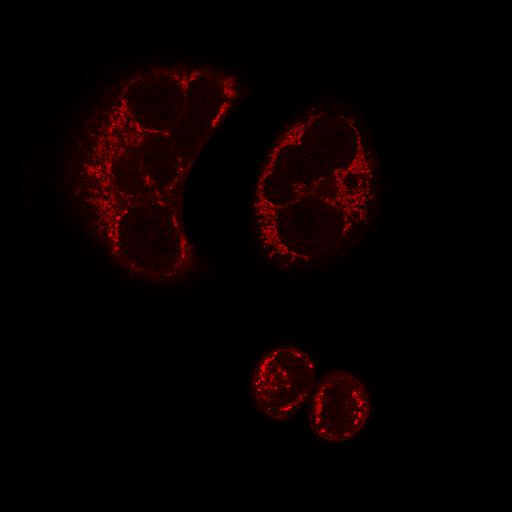

Supplement: Figure 8—source data 1. [file elife-69916-fig8-data1.zip › Figure8_Sourcedata_localization of Endonuclease G/Figure 8A_Representative images_localization of EndoG to mitochondria/Figure 8A_Source file_2_HeLa/1_0005.tif.frames/1_0005_C002T001.tif]

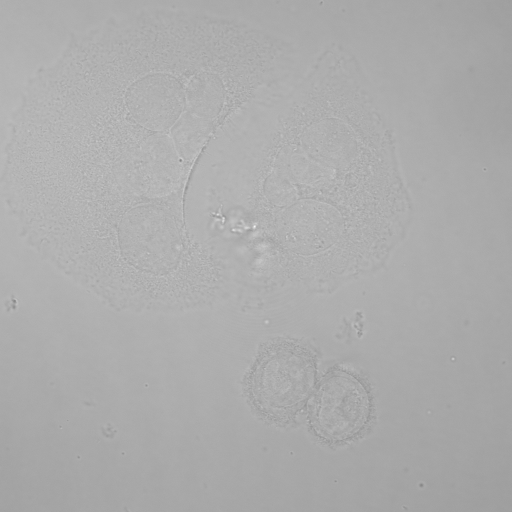

Supplement: Figure 8—source data 1. [file elife-69916-fig8-data1.zip › Figure8_Sourcedata_localization of Endonuclease G/Figure 8A_Representative images_localization of EndoG to mitochondria/Figure 8A_Source file_2_HeLa/1_0005.tif.frames/1_0005_C004T001.tif]

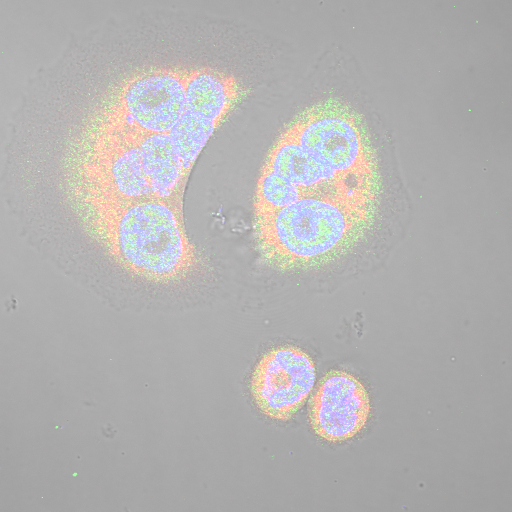

Supplement: Figure 8—source data 1. [file elife-69916-fig8-data1.zip › Figure8_Sourcedata_localization of Endonuclease G/Figure 8A_Representative images_localization of EndoG to mitochondria/Figure 8A_Source file_2_HeLa/1_0005.tif.frames/1_0005_T001.tif]

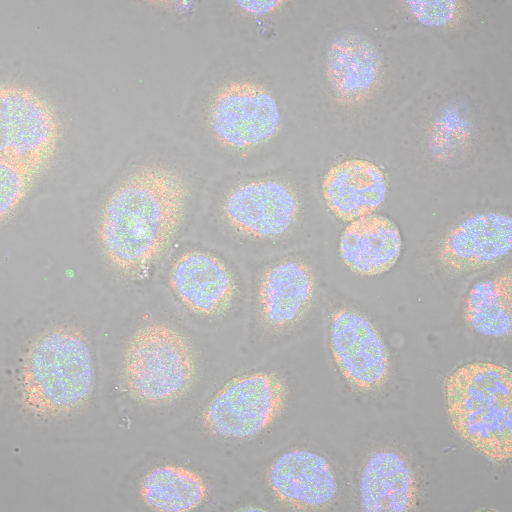

Supplement: Figure 8—source data 1. [file elife-69916-fig8-data1.zip › Figure8_Sourcedata_localization of Endonuclease G/Figure 8A_Representative images_localization of EndoG to mitochondria/Figure 8A_Source file_2_HeLa/1.tif.frames/1_T001.tif]

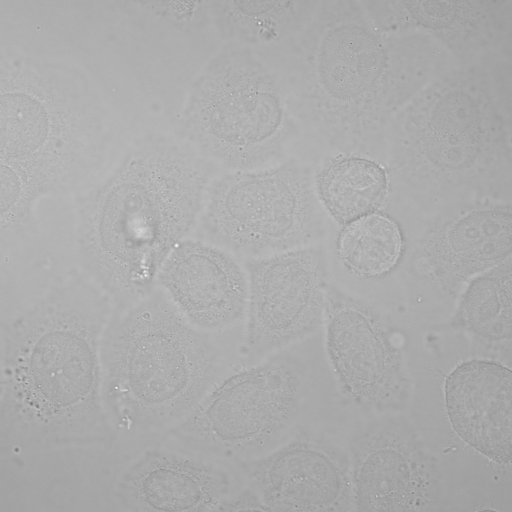

Supplement: Figure 8—source data 1. [file elife-69916-fig8-data1.zip › Figure8_Sourcedata_localization of Endonuclease G/Figure 8A_Representative images_localization of EndoG to mitochondria/Figure 8A_Source file_2_HeLa/1.tif.frames/1_C004T001.tif]

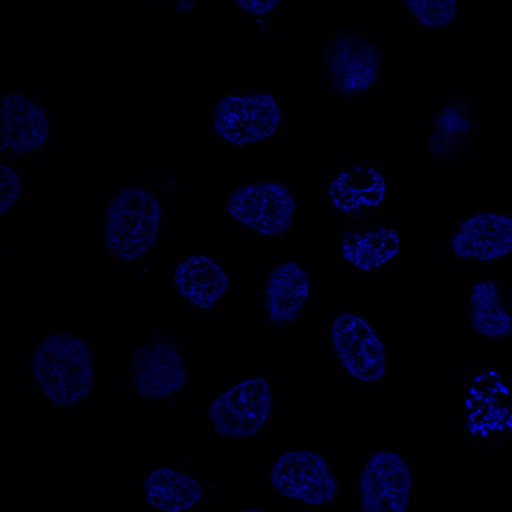

Supplement: Figure 8—source data 1. [file elife-69916-fig8-data1.zip › Figure8_Sourcedata_localization of Endonuclease G/Figure 8A_Representative images_localization of EndoG to mitochondria/Figure 8A_Source file_2_HeLa/1.tif.frames/1_C001T001.tif]

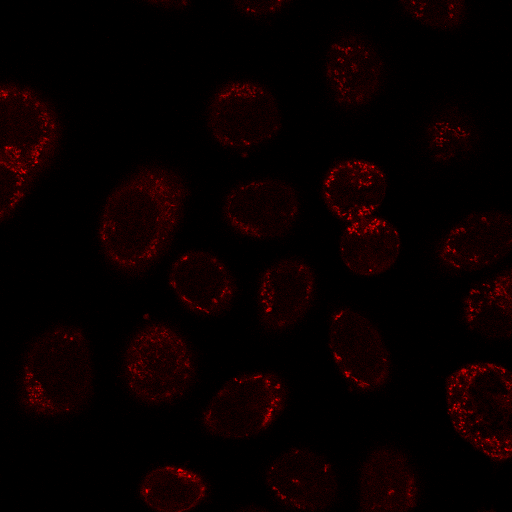

Supplement: Figure 8—source data 1. [file elife-69916-fig8-data1.zip › Figure8_Sourcedata_localization of Endonuclease G/Figure 8A_Representative images_localization of EndoG to mitochondria/Figure 8A_Source file_2_HeLa/1.tif.frames/1_C002T001.tif]
