# Supplementary material for: Unleashing a novel function of Endonuclease G in mitochondrial genome instability
Source: eLife. 2022 Nov 17;11:e69916. doi: 10.7554/eLife.69916 (PMC9711528; doi:10.7554/eLife.69916)
Supplement: Figure 9—source data 2. [file elife-69916-fig9-data2.zip › Figure 9_Source data_Supplementary/Figure S9D_Gel profile for Input and endoG pulldown_mitoChIP/Figure S5D_Gel profile.pptx]

## Slide 1
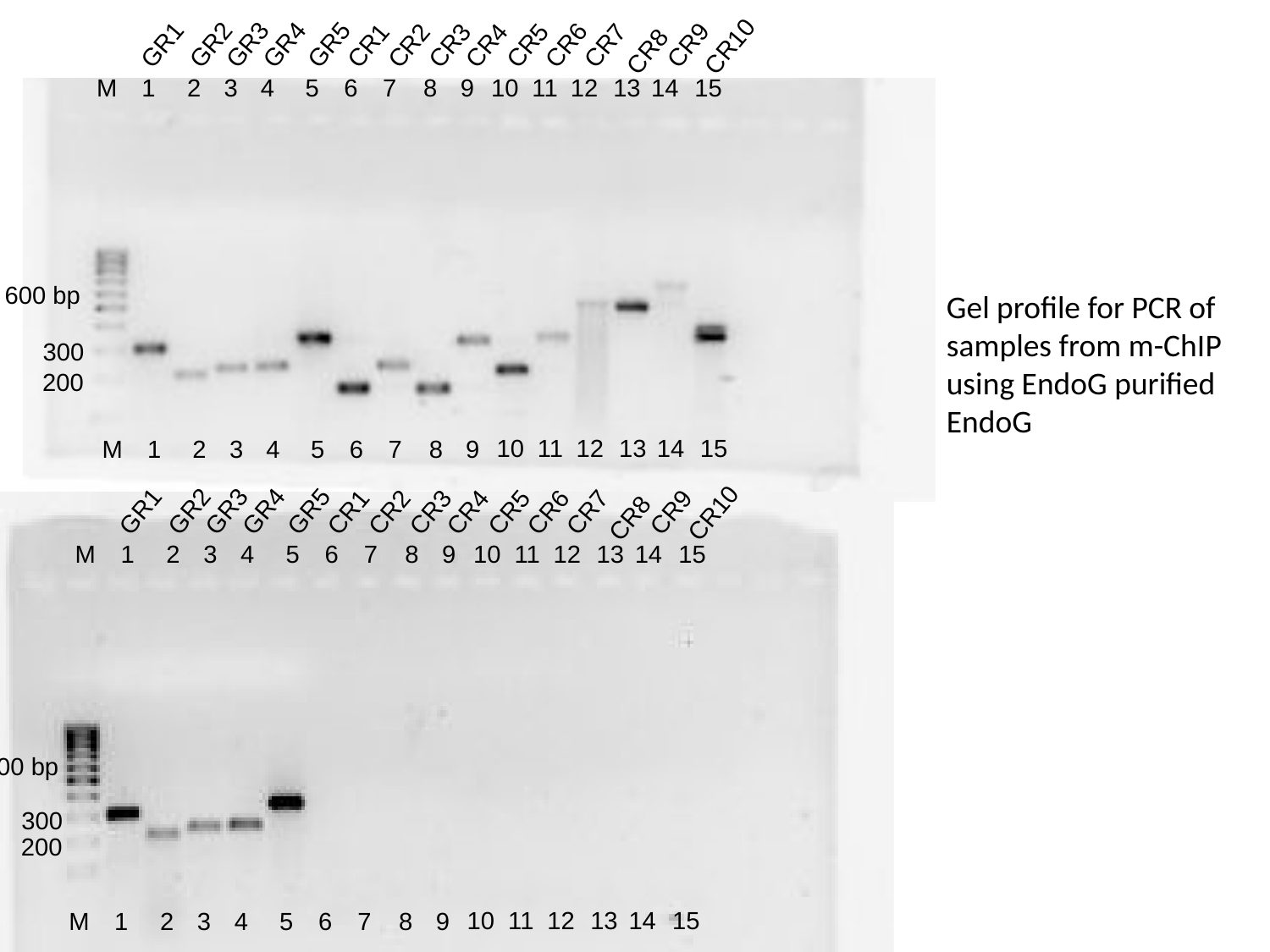

GR1
GR2
GR3
GR4
GR5
CR1
CR2
CR3
CR4
CR5
CR6
CR7
CR9
CR10
CR8
10
11
12
13
14
15
M
1
2
3
4
5
6
7
8
9
600 bp
300
200
10
11
12
13
14
15
M
1
2
3
4
5
6
7
8
9
Gel profile for PCR of samples from m-ChIP using EndoG purified EndoG
GR1
GR2
GR3
GR4
GR5
CR1
CR2
CR3
CR4
CR5
CR6
CR7
CR9
CR10
CR8
10
11
12
13
14
15
M
1
2
3
4
5
6
7
8
9
600 bp
300
200
10
11
12
13
14
15
M
1
2
3
4
5
6
7
8
9
