# Supplementary material for: Unleashing a novel function of Endonuclease G in mitochondrial genome instability
Source: eLife. 2022 Nov 17;11:e69916. doi: 10.7554/eLife.69916 (PMC9711528; doi:10.7554/eLife.69916)
Supplement: Figure 9—source data 3. [file elife-69916-fig9-data3.zip › Figure 10_Sourcedata_Supplementary/Figure S10B_Gel profile_P1 nuclease assay/Figure S5G_Source file_P1 nuclease assay.pptx]

## Slide 1
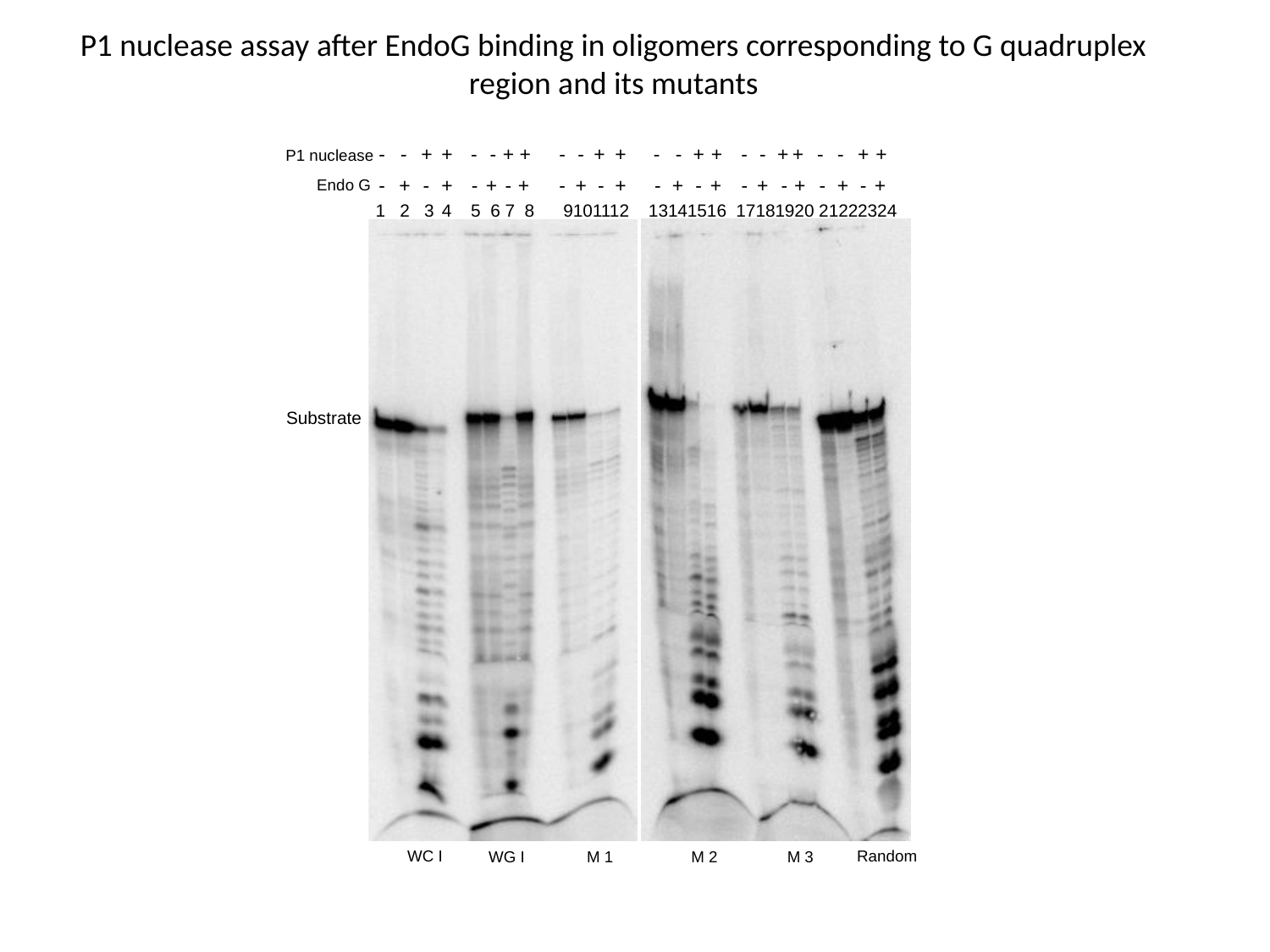

P1 nuclease assay after EndoG binding in oligomers corresponding to G quadruplex region and its mutants
-
-
+
+
-
-
+
+
-
-
+
+
-
-
+
+
-
-
+
+
-
-
+
+
P1 nuclease
-
+
-
+
-
+
-
+
-
+
-
+
-
+
-
+
-
+
-
+
-
+
-
+
Endo G
1 2 3
4 5 6 7 8 9101112 13141516 17181920 21222324
Substrate
WC I
Random
M 1
M 3
WG I
M 2
