# Supplementary material for: Unleashing a novel function of Endonuclease G in mitochondrial genome instability
Source: eLife. 2022 Nov 17;11:e69916. doi: 10.7554/eLife.69916 (PMC9711528; doi:10.7554/eLife.69916)
Supplement: Figure 9—source data 3. [file elife-69916-fig9-data3.zip › Figure 10_Sourcedata_Supplementary/Figure S10A_Gel profile after ChIP/Figure S5F_Supplementary_ Source data_.pptx]

## Slide 1
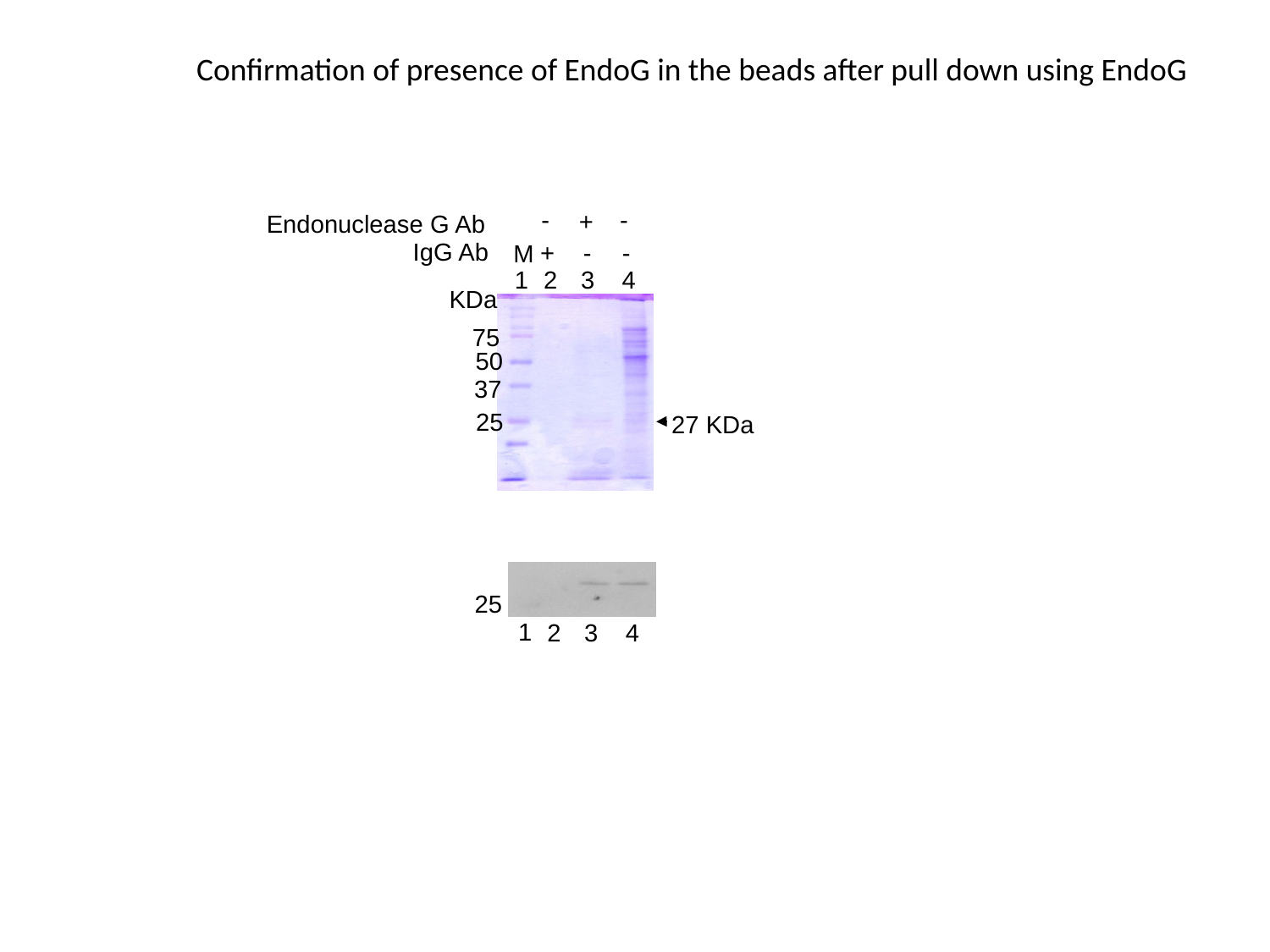

Confirmation of presence of EndoG in the beads after pull down using EndoG
-
-
+
Endonuclease G Ab
IgG Ab
-
+
-
M
2
3
4
1
KDa
75
50
37
25
27 KDa
25
1
2
3
4
