# Supplementary material for: Unleashing a novel function of Endonuclease G in mitochondrial genome instability
Source: eLife. 2022 Nov 17;11:e69916. doi: 10.7554/eLife.69916 (PMC9711528; doi:10.7554/eLife.69916)
Supplement: Figure 10—source data 1. [file elife-69916-fig10-data1.zip › Figure10_Sourcedata_stress mediated sublocalization of EndoG/Figure 10A_Western blotting_after subfractionation_stress condition/Figure 10A_Labelled blot.pptx]

## Slide 1
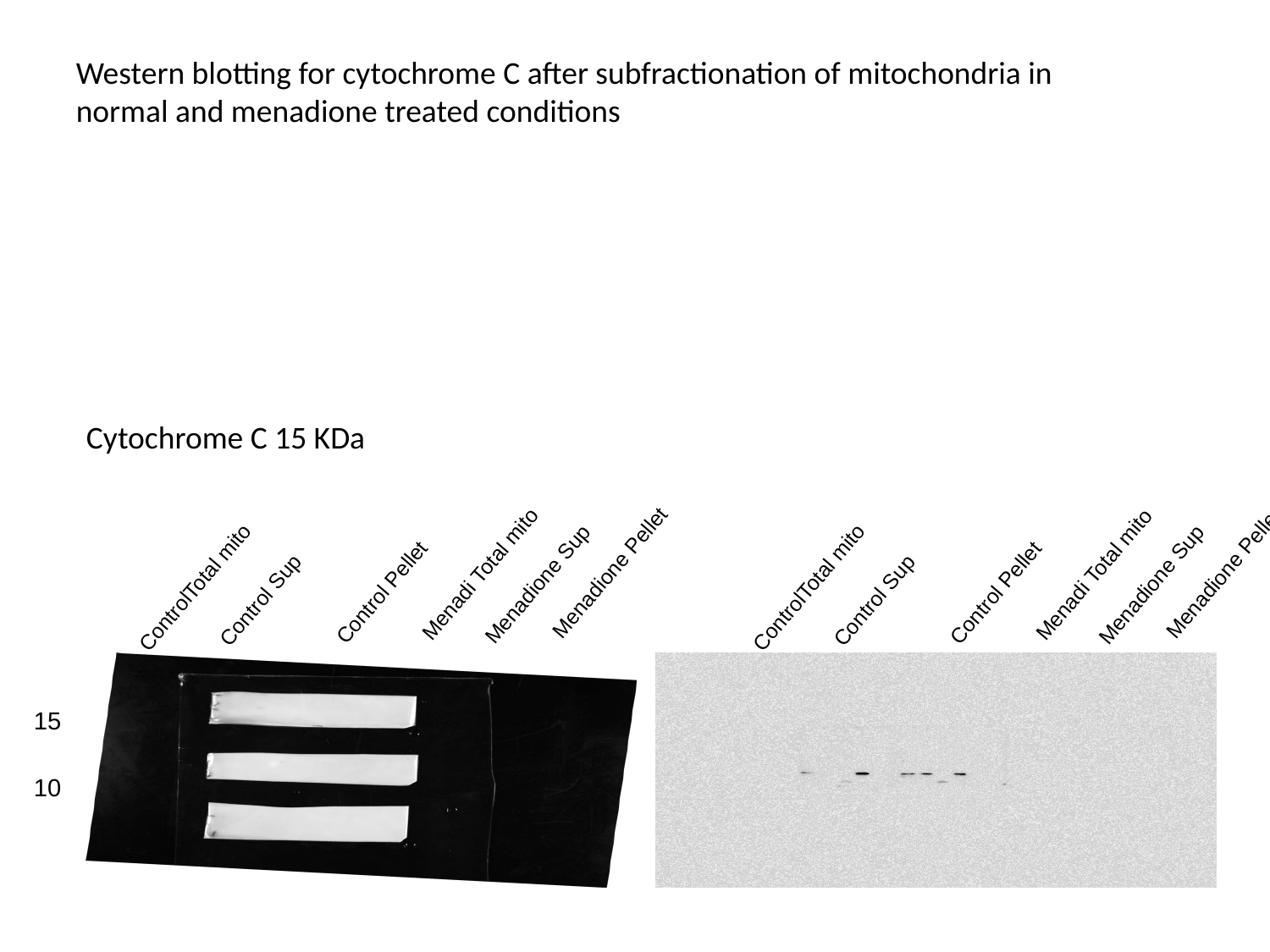

Western blotting for cytochrome C after subfractionation of mitochondria in normal and menadione treated conditions
Cytochrome C 15 KDa
Menadione Pellet
Menadione Pellet
 Menadi Total mito
 Menadi Total mito
Menadione Sup
Menadione Sup
 ControlTotal mito
 ControlTotal mito
Control Pellet
Control Pellet
Control Sup
Control Sup
15
10

## Slide 2
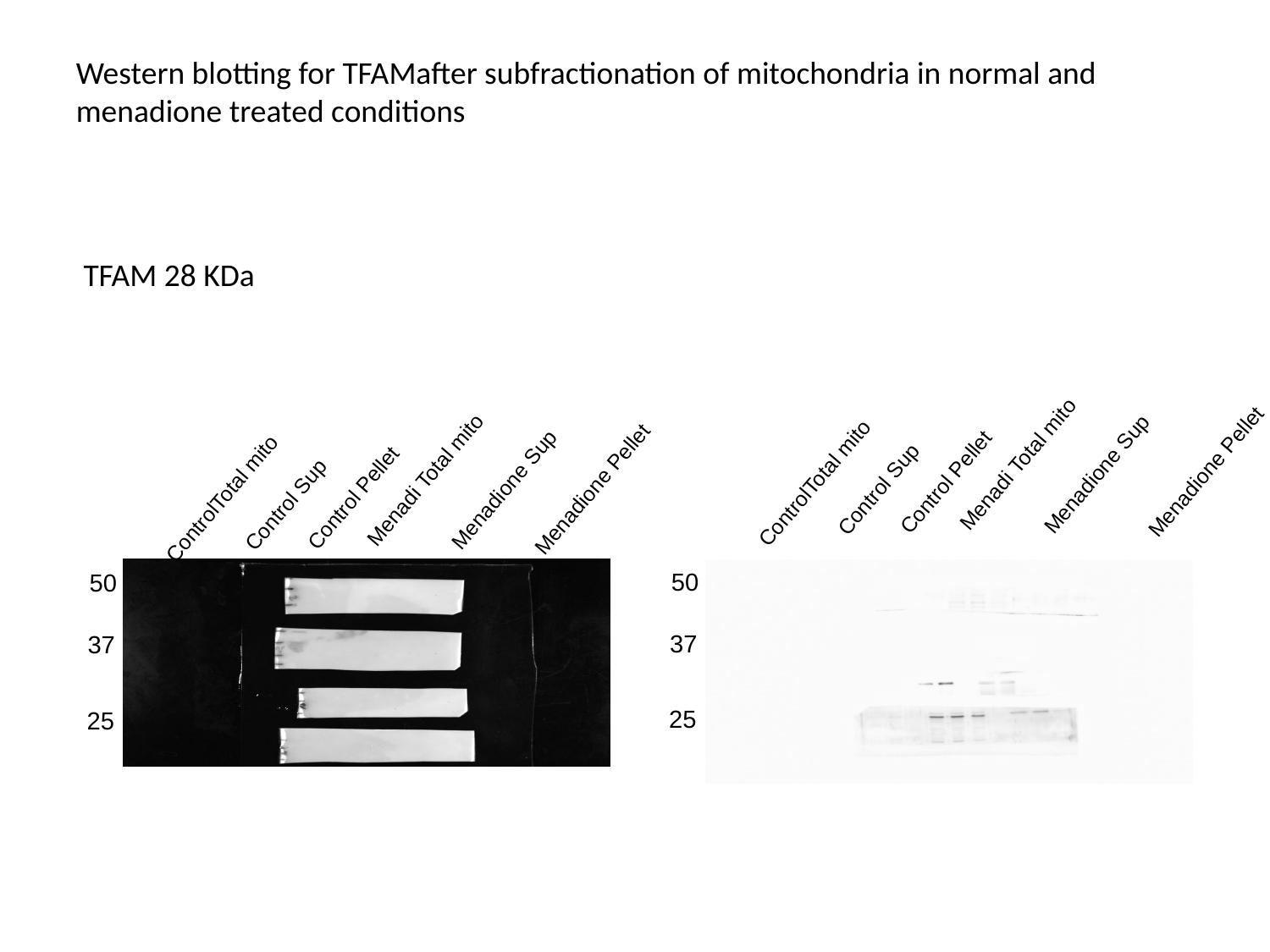

Western blotting for TFAMafter subfractionation of mitochondria in normal and menadione treated conditions
TFAM 28 KDa
 Menadi Total mito
Menadione Sup
 Menadi Total mito
 ControlTotal mito
Control Pellet
Menadione Sup
Control Sup
 ControlTotal mito
Control Pellet
Control Sup
50
50
37
37
25
25
Menadione Pellet
Menadione Pellet

## Slide 3
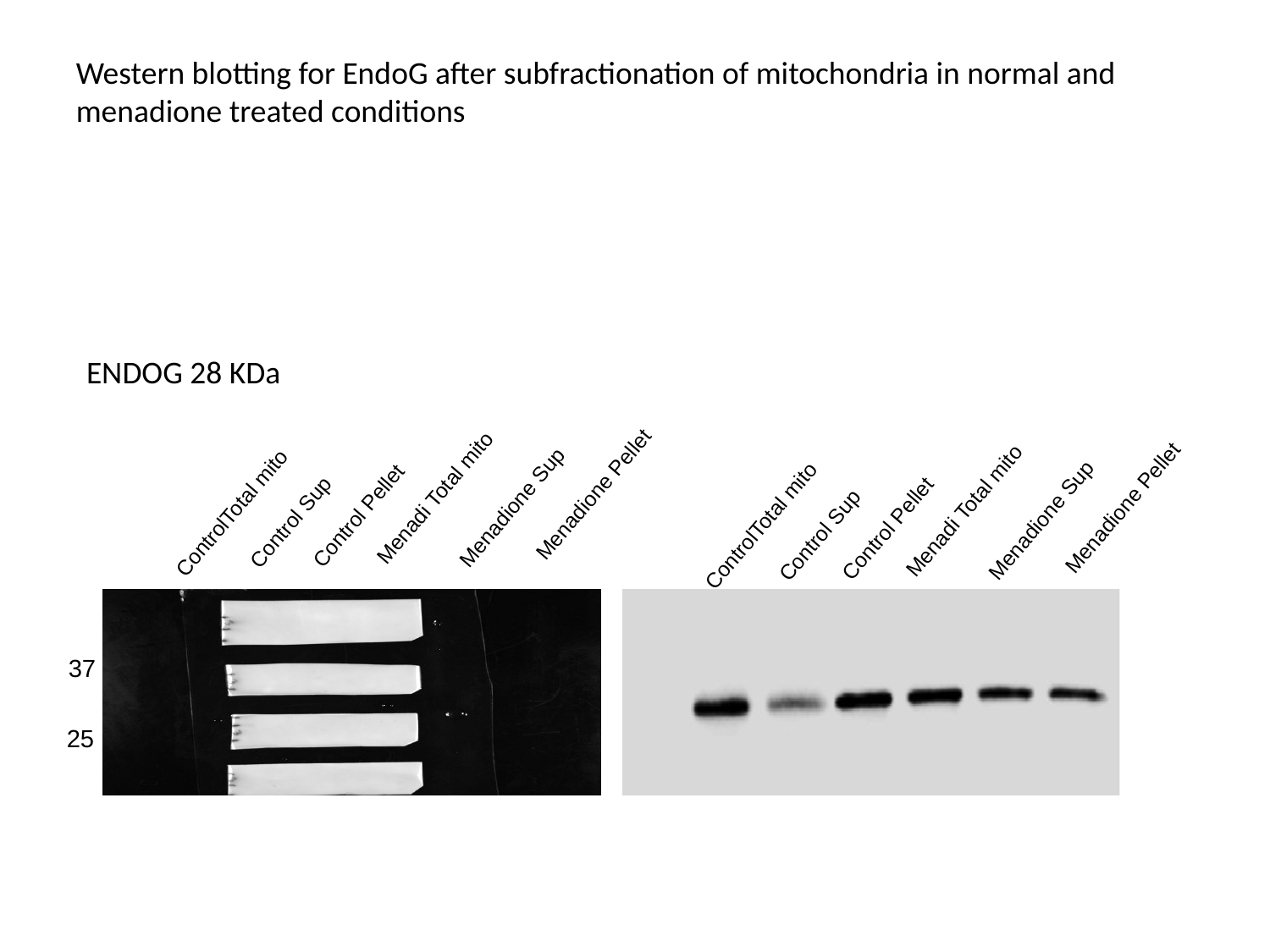

Western blotting for EndoG after subfractionation of mitochondria in normal and menadione treated conditions
Menadione Pellet
 Menadi Total mito
Menadione Pellet
Menadione Sup
 ControlTotal mito
 Menadi Total mito
Control Pellet
Menadione Sup
Control Sup
 ControlTotal mito
Control Pellet
Control Sup
37
25
ENDOG 28 KDa
